# Catalytic enantioselective synthesis of alkylidenecyclopropanes

**DOI:** 10.1038/s41586-025-09485-y

**Published:** 2025-08-11

**Authors:** Jonathan C. Golec, Dong-Hang Tan, Ken Yamazaki, Eveline H. Tiekink, Kirsten E. Christensen, Trevor A. Hamlin, Darren J. Dixon

**Affiliations:** 1 Chemistry Research Laboratory, Department of Chemistry, University of Oxford, Oxford, UK; 2 Sygnature Discovery, BioCity, Nottingham, UK; 3 Department of Chemistry and Pharmaceutical Sciences, Amsterdam Institute of Molecular and Life Sciences (AIMMS), Vrije Universiteit Amsterdam, Amsterdam, The Netherlands; 4 Division of Applied Chemistry, Okayama University, Okayama, Japan

**Keywords:** Synthetic chemistry methodology, Stereochemistry, Asymmetric catalysis, Asymmetric synthesis

## Abstract

The enantioselective construction of small-ring carbocycles provides organic chemists with an enduring challenge^1^. Despite their commercial importance, enantioselective synthetic routes towards alkylidenecyclopropanes, a class of small-ring carbocycles, remain underdeveloped^2,3^. Alkylidenecyclopropanes can be converted into cyclopropanes, a common feature in drug molecules (for example, Nirmatrelvir **1**)^4^, as well as both naturally occurring and synthetic agrochemicals (for example, permethrin **2**)^5,6^. Here we describe the facile synthesis of highly enantioenriched alkylidenecyclopropanes through the use of a bifunctional iminophosphorane catalysed, stereo-controlled, strain-relieving deconjugation. Small modifications to the basic catalyst system were used to broaden the scope of the reaction to substrates containing ester, amide, phosphine oxide and ketone functionalities. Through the design of a suitable substrate and retuning of the catalyst’s iminophosphorane moiety, the transformation was effectively applied to the synthesis of a single stereoisomer of the commonplace insecticide permethrin as well as a range of cyclopropane-based insecticide cores. State-of-the-art computational studies were performed to provide detailed insights into the mechanistic pathway and origin of both diastereoselectivities and enantioselectivities.

## Abstract (web summary)

A bifunctional iminophosphorane-catalysed, stereo-controlled deconjugation for the synthesis of highly enantioenriched alkylidenecyclopropanes is described, alongside computational studies elucidating the mechanistic pathway and origins of diastereoselectivity and enantioselectivity.

## Main

**Fig. 1:**
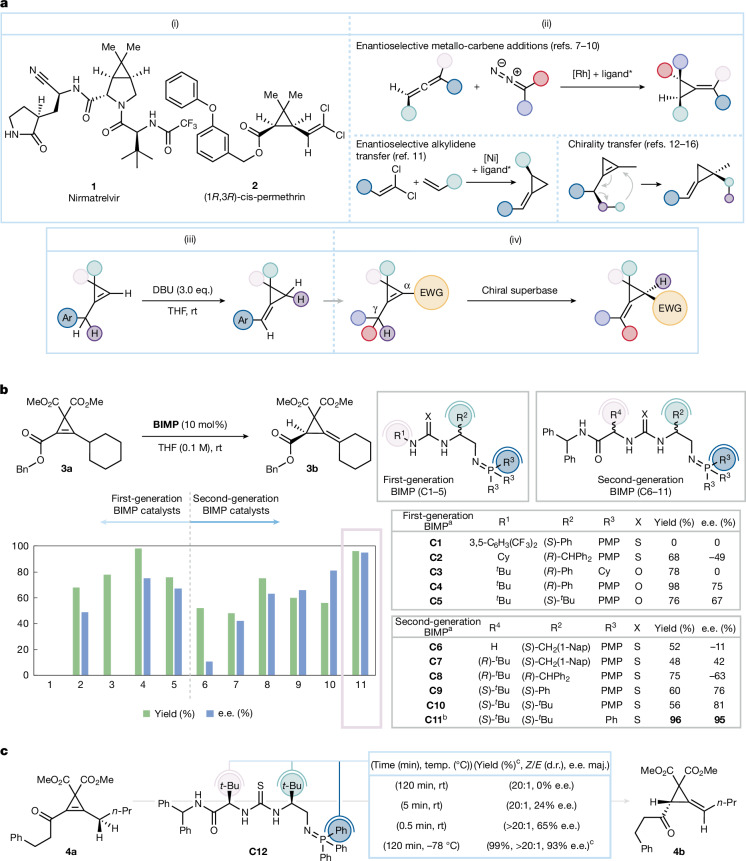
Previous art, concept and reaction optimizations. **a**, Drug molecules and agrochemicals containing cyclopropene rings, state-of-the-art synthetic approaches towards chiral ACPs and strain-relieving concept of cyclopropenes to ACPs: (i) natural products and bioactive compounds; (ii) enantioselective approaches to ACPs; (iii) conjugation approach^40^ and (iv) enantioselective deconjugation concept. **b**, Catalyst screening for the enantioselective strain-relieving deconjugation of substrate **3a**. **c**, Conditions for the enantioselective strain-relieving deconjugation of substrate **4a** with catalyst **C12**. In **b**, e.e. was determined by high-performance liquid chromatography analysis on a chiral stationary phase. Also the reactions were carried out with 0.013 mmol of **3a**. ^a^Reaction carried out in Et_2_O (0.05 M). ^b^Yield only obtained for the last entry, reactions were carried out in Et_2_O (0.05 M). ^c^Reaction was carried out with 0.10 mmol of substrate. DBU, 1,8-diazabicyclo[5.4.0]undec-7-ene; Nap, naphthalenyl; PMP, *para*-methoxyphenyl; rt, room temperature; temp., temperature; THF, tetrahydrofuran.

At present, the enantioselective synthesis of alkylidenecyclopropanes (ACPs) is limited. Asymmetric metallocarbene additions to allenes^7–10^, reductive enantioselective alkylidene transfer^11^ and chirality transfer^12–16^ represent the main pathways to these desirable products (Fig. 1a(ii)). The most used method, metallocarbene addition, fails to deliver ACPs with substituents on each cyclopropane carbon as the addition occurs at the terminal alkene, a consequence of both electronic and steric considerations. Although metallocarbenes can be added to electron poor alkenes using cobalt catalysis, this chemistry has not yet been applied to allenes^17,18^. An enantioselective transformation to overcome these limitations represents a notable advancement in the field.

We sought to use our superbasic^19–25^ bifunctional iminophosphorane (BIMP) catalyst^26–33^ to promote their efficient and enantioselective synthesis by means of a conceptually new strain-relieving, deconjugative^34,35^, prototropic shift^29,36,37^ making use of substrates that can be readily prepared from commercially available materials.

Exploitation of the cyclopropene ring’s highly strained character provided the cornerstone for the investigation^38,39^. Double bond migration from an *endo*-cyclic to an *exo*-cyclic position has previously been reported and generates a new tetrahedral-like carbon in the cyclopropane ring^40–42^. This substrate scope limited, non-enantioselective or diastereoselective transformation requires super-stoichiometric (achiral) bases, harsh reaction conditions and, with organic bases, a conjugative driving force (Fig. 1a(iii))^40^. We recognized that if the cyclopropene in Fig. 1a(iii) contains a prochiral *π*-bonded carbon atom, a stereogenic centre is generated following the migration of the double bond. Theoretically, a suitable electron withdrawing group attached to the prochiral carbon should allow a suitably strong, chiral Brønsted base to achieve enantioselective proton transfer from the external γ-position to the α-position^43–45^, yielding a more stable chiral ACP. When combined with exquisite catalyst control, this downhill thermodynamic driving force could lead to a new and broad-in-scope strategy to valuable small-ring structures; to this end, here we report our findings (Fig. 1a(iv)).

Test substrate **3a** was selected and straightforwardly synthesized for investigation (Fig. 1b)^46,47^. The first generation BIMP catalysts (**C1**–**C5**) featuring a single stereogenic centre were efficient deconjugation promoters, and the desired products were obtained in good to excellent yield (68–98%). Whereas yields were generally good, enantiocontrol pivoted around base strength. Thus, whereas catalyst **C4** imparted encouraging enantiocontrol (75% enantiomeric excess (e.e.)), the more basic PCy_3_-derived iminophosphorane (**C3**) gave none (0% e.e.). Good enantiocontrol was obtained with both thiourea catalyst **C2** (−49% e.e.) and urea derived catalysts **C4** and **C5** (75% e.e. and 67% e.e., respectively) despite the difference in hydrogen bond donor strength, yields were higher on average with urea-based catalysts.

Second-generation (**C6**–**C11**) BIMP catalysts bearing an extra α-amino amide moiety distal to the iminophosphorane group were then investigated for improved enantiocontrol. Catalyst **C6** possessing a single stereogenic centre imparted low enantioselectivity (−11% e.e.) and moderate yield (52%). However, the introduction of a second stereogenic centre (**C7**) boosted enantioselectivity (42% e.e.) while maintaining yield (48%). Further improvements to the selectivity were achieved by increasing the steric bulk of R^2^ at the proximal stereogenic centre. Use of a benzhydryl group (**C8**) increased the enantioselectivity to −63% e.e. and a moderate increase to 76% e.e. was observed with a Ph group in place (**C9**). Finally, when R^2^ was a *t*-Bu (**C10**) the enantioselectivity was elevated to 81% e.e. Generally, the stereocentre close to the iminophosphorane moiety dictated the product’s absolute stereochemical configuration, with *S* providing the *R-*configured products and vice versa. An exception was noted with catalyst **C6** that was probably due to the lack of a secondary stereocentre affecting the catalyst’s reactive conformation. Finally, we reduced the catalyst’s Brønsted basicity with a PPh_3_-derived iminophosphorane (**C11**) that, on switching the solvent to Et_2_O (0.05 M), provided **3b** in 96% yield and 95% e.e.

**Fig. 2:**
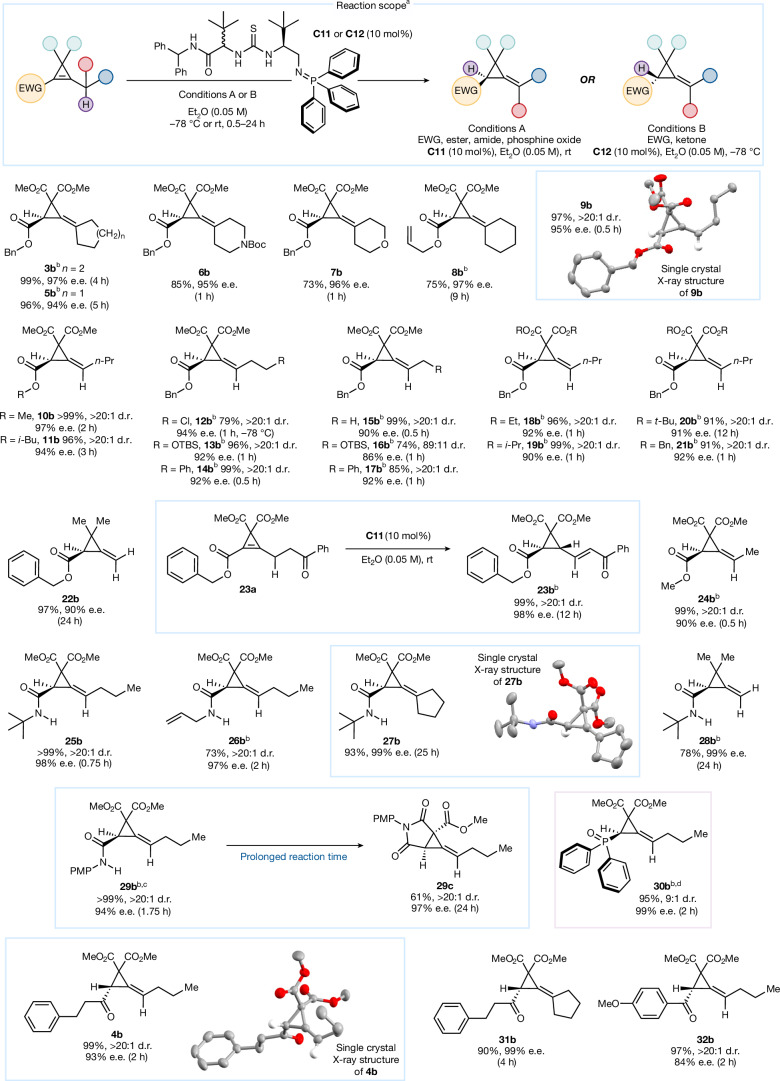
Reaction scope for the enantioselective strain-relieving deconjugation of cyclopropenes. ^a^Reactions were carried out with 0.1 mmol of substrate; all e.e. were determined by high-performance liquid chromatography or supercritical fluid chromatography analysis on chiral stationary phase. ^b^Reaction carried out with 0.05 mmol of substrate. ^c^Reaction carried out in tetrahydrofuran. ^d^−78 to 0 °C. EWG, electron-withdrawing group; TBS, *tert*-butyldimethylsilane.

α,β-Unsaturated ketones were also investigated to expand the applicable chemical space of the transformation (Fig. 1c). Substrate **4a** presented a new challenge in the control of the enantioselectivity and *E*/*Z* geometry of the product. Following extensive investigation (Supplementary Information), catalyst **C12**, the diastereomer of **C11** was identified as an efficient catalyst for the transformation. Substrate **4a** was rapidly converted to product **4b** within 120 minutes and with excellent diastereocontrol (more than 20:1 d.r.). Unfortunately, enantiocontrol was completely lost (0% e.e.) indicative of catalyst-enabled racemization of the more acidic ketonic system (Supplementary Information). Reduction of the overall reaction time (120 minutes (0% e.e.); 5 minutes (24% e.e.); 0.5 minutes (65% e.e.)) revealed that the enantioselectivity could be rescued. Consequently, we performed the reaction at the reduced temperature of −78 °C and the ACP (**4b**) rapidly formed (2 hours) in 99% yield, more than 20:1 d.r. and 93% e.e. Single-crystal X-ray diffraction analysis of **4b** confirmed the absolute stereochemical configuration as (*S*) (Fig. 2).

Next, we proceeded to explore the reaction scope (Fig. 2). Replacement of the cyclohexyl group (**3a**) with a cyclopentyl ring (**5a**) had no detrimental effect on reactivity (96%), and the product (**5b**) was obtained in 94% e.e. within 5 h. Heterocyclic rings were also well tolerated, and the products **6b** and **7b** were obtained in 95% and 96% e.e., respectively. Furthermore, substrate **8a** with an allyl ester was converted easily and with excellent selectivity (75%, 97% e.e.).

Under the control of catalyst **C11**, substrate **9a**, featuring an *n*-propyl chain and a benzyl ester, was converted to **9b** in 30 minutes with remarkable selectivity for the (*Z*)-isomer (more than 20:1 d.r.), 95% e.e. and 97% yield. Steady racemization of **9b** occurred over time under the reaction conditions after near instantaneous product formation due to the acidity of **9b**’s α-proton (Supplementary Fig. 1). Single-crystal X-ray diffraction analysis confirmed the absolute stereochemical configuration of **9b** as (*R*). Methyl-ester **10b** and *iso*-butyl ester **11b** formed rapidly (2 hours and 3 hours, respectively) with excellent levels of diastereoselectivity (more than 20:1 d.r.) and enantioselectivity (97% e.e. and 94% e.e., respectively).

Next, a selection of functionalized alkyl chains were investigated. At reduced temperature, an alkyl chloride was well tolerated, and **12b** was obtained in 79% yield and 94% e.e. as a single diastereomer. O-*tert*-butyldimethylsilane and Ph were also efficiently converted to their deconjugated products with both *n*-Pr (**13b** and **14b**, respectively) and *n*-Et (**16b** and **17b**, respectively) chains. Enantioselectivity generally exceeded 90% and most products were obtained as single diastereomers. A moderate drop in both diastereoselectivity (89:11) and enantioselectivity (86% e.e.) occurred with substrate **16a**, probably due to the proximal bulk of the *tert*-butyldimethylsilane group.

We investigated the effect of substituents attached to the cyclopropene’s bridging methylene carbon. Four ester variations, Et (**18a**), *i*-Pr (**19a**), *t*-Bu (**20a**) and Bn (**21a**) were trialled. In each case deconjugation occurred in excellent yield and, despite the increased bulk at the bridge, enantioselectivity was maintained. Removal of the bridging esters was also possible as demonstrated with substrate **22a**. The reaction progressed slowly over 24 h and despite the new *gem*-dimethyl motif, **22b** was obtained in 97% yield and 90% e.e.

We next designed a new substrate featuring a pendant ketone (**23a**). On exposure to the reaction conditions the prototropic shift proceeded smoothly, and the resulting ACP underwent further transformation to enone **23b**. All three stereocentres were formed with excellent control resulting in a single trans configured cyclopropane diastereomer (more than 20:1 d.r) and near-perfect enantiomeric excess (98% e.e.).

Finally, we synthesized substrate **24a** that represented an ideal substrate for comparison with our computational studies (vide infra) and featured a methyl-ester and ethyl chain that, under the reaction conditions, transformed into **24b** in 99% yield, more than 20:1 d.r. and 90% e.e. in 30 minutes.

Furthermore, a range of amides were tested under the optimized reaction conditions for ester substrates. *tert*-Butyl amide substrate **25a** was converted to **25b** in under 1 hour regardless of the reduced acidity of the γ-proton. Despite the amide’s hydrogen bond donating N–H group, **25b** was obtained in more than 20:1 d.r. and with complete enantiocontrol (98% e.e.). Similarly, allyl amide ACP **26b** formed rapidly (2 hours) under the reaction conditions in 73% yield, more than 20:1 d.r. and 97% e.e. Amide **27a**, featuring a cyclopentyl ring was tolerated and the ACP (**27b**) was obtained in 93% yield and 99% e.e. Single-crystal X-ray analysis of **27b** confirmed the absolute stereochemical configuration as *R* and a supplementary study showed that no racemization occurred over 80 h. Substrate **28a** converted to **28b** in good yield of 78% with complete enantiocontrol (99% e.e.) under a reduced time-frame (24 h). *para*-Methoxyphenyl amide (**29a**) was smoothly converted to **29b** in more than 99% yield, more than 20:1 d.r. and 94% e.e. in 1.75 h. With prolonged exposure to the reaction conditions, **29b** underwent intramolecular cyclization to provide fused ring system **29c** with complete diastereocontrol (more than 20:1 d.r.) and near-perfect enantioselectivity (97% e.e.). As a stand-alone example, a substrate featuring a phosphine oxide (**30a**) was successfully tested. Compound **30b** was obtained in high yield of 95% and 99% e.e. of the main diastereomer succinctly demonstrating the transformation’s broad utility.

Catalyst **C12** was then used to investigate the scope of ketone substrates. Replacement of **4a**’s *n*-Pr chain with a cyclopentane ring (**31a**) was well tolerated and **31b** was obtained in 90% yield and 99% e.e. in 4 hours. *para*-Methoxyphenyl ketone (**32a**) could also be used albeit with moderately reduced enantioselectivity of 84% e.e. but an excellent yield of 97% and complete diastereocontrol (more than 20:1 d.r.).

As of 2020, pyrethroid insecticides accounted for 25% of global insecticide sales^48^. Despite the differing biological properties of their stereoisomers, few direct enantioselective methods exist for their synthesis^48^. Current routes involve chiral auxiliaries^49^, resolution approaches^5^ or stoichiometric quantities of chiral reagents^50^. The development of catalytic enantioselective approaches have been met with limited success^51^.

**Fig. 3:**
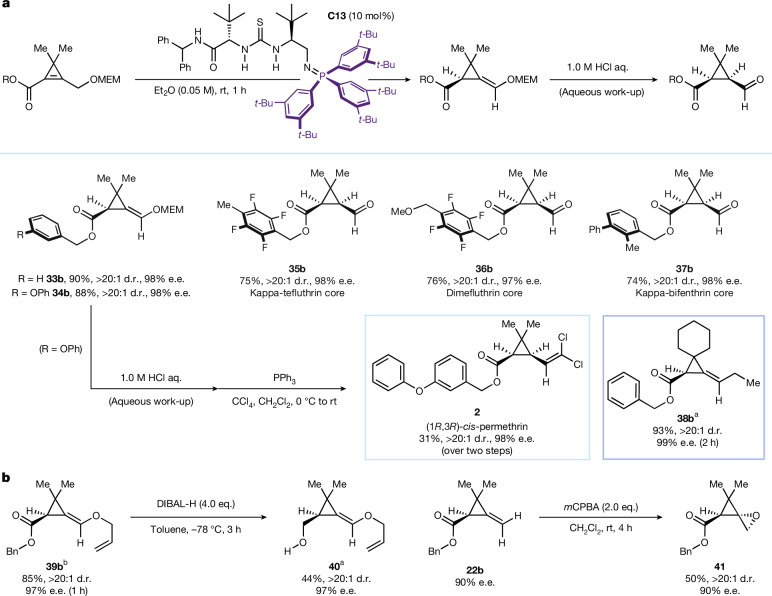
Enantioselective synthesis of insecticides and their precursors. **a**, Enantioselective synthesis of (1*R*,3*R*)-*cis*-permethrin and insecticide cores using catalyst **C13**. **b**, Derivatization of product **39b** and **22b**. In **a**, reactions were carried out with 0.1 mmol of substrate and all e.e. were determined by high-performance liquid chromatography or supercritical fluid chromatography analysis on chiral stationary phase. ^a^Reaction carried out with 0.05 mmol of substrate. ^b^Reaction carried out with 0.2 mmol of substrate. OMEM, methoxyethoxymethyl ether.

Pyrethroids have several common, recurring structural features, most notably their *gem*-dimethyl cyclopropane, alkene and carbonyl motifs (Fig. 1a). To incorporate these features into our ACP products, a modified strategy was realized. Placement of a suitably protected alcohol at the site of deprotonation would result in the formation of an enol-ether on successful migration of the double bond. Deprotection of the enol-ether would then reveal an aldehyde amenable to further functionalization while allowing two stereocentres to be set in a single step (Fig. 3a). A test substrate **33a** containing the required structural features was accordingly synthesized (Supplementary Information).

Initially, the deconjugation reaction was tested on substrate **33a** using catalyst **C11**. No conversion to the desired product was observed, however, the BIMP’s iminophosphorane could be easily tuned by variation of the triarylphosphine, and the catalyst was quickly re-optimized to suit the new substrate (Supplementary Information). The new catalyst (**C13**) featured a sterically encumbered triarylphosphine moiety and rapidly formed the desired product **33b** (90%) with excellent levels of diastereocontrol (more than 20:1 d.r.) and enantiocontrol (98% e.e.). A deuterium labelling study of **33a** was undertaken and a large kinetic isotope effect observed for the transformation (Supplementary Information). An enantioselective synthesis of permethrin was performed using the new catalyst **C13** showcasing the synthetic utility of the transformation. Following rapid formation of enol-ether **34b** (1 hour), an aqueous acidic work-up provided the crude aldehyde as a single diastereomer that, on being subjected to Corey–Fuchs reaction conditions, formed (1*R*,3*R*)-*cis*-permethrin (**2**) as a single diastereomer in 98% e.e. completing the route in six steps (longest linear sequence, Supplementary Information).

The scope was expanded to aldehyde-cores of other pyrethroid-type insecticides (Fig. 3a). In each case, **C13** performed well, and following an acid work-up, aldehyde-cores to kappa-tefluthrin (**35b**), dimefluthrin (**36b**) and kappa-bifenthrin (**37b**) could all be obtained as single diastereomers in >97% e.e. within 1 h.

**C13** was also applicable to substrates that did not feature the pendent ether chain. A spirocyclic cyclopropene with a bridging cyclohexyl group (**38a**) was synthesized. The desired deconjugated product formed rapidly (2 hours) with high levels of diastereoselectivity (more than 20:1 d.r.) and enantioselectivity (99% e.e.).

Next, to showcase the synthetic potential of the ACPs we proceeded to investigate the derivatization of **39b** and **22b** (Fig. 3b). **39b** was synthesized using **C13** in good yield (85%) and with excellent stereocontrol (more than 20:1 d.r., 97% e.e.) and was converted into alcohol **40** using DIBAL-H at reduced temperature. The product (**40**) was obtained without loss of enantioenrichment (97% e.e.) in good yield (44%). ACP **22b** was smoothly converted into spirocycle **41** by means of stereoselective epoxidation with *m*CPBA providing the desired compound as a single diastereomer (more than 20:1) while maintaining 90% e.e.

**Fig. 4:**
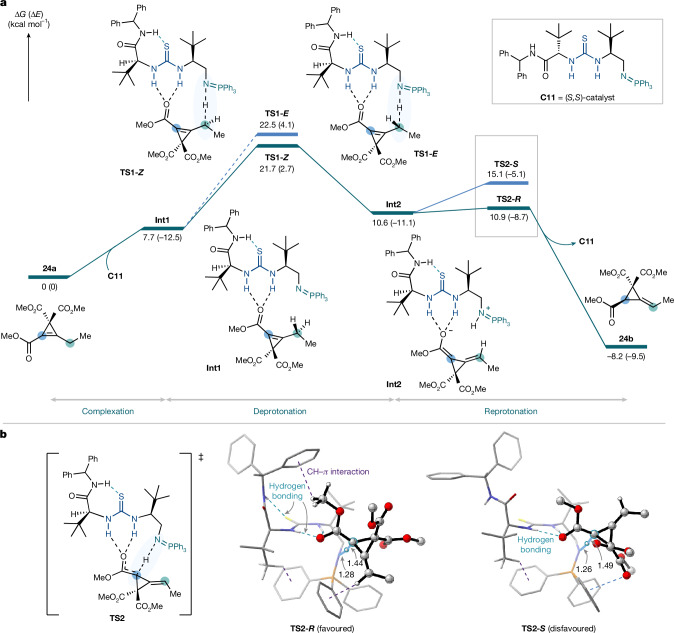
DFT study of the reaction pathway with ester-substituted cyclopropene. **a**, Potential energy surface diagram. **b**, Transition state structures for reprotonation. Computed potential energy surfaces (Δ*G* (kcal mol^−^^1^)) for the 1,3-prototropic shift of ester **24a** computed at COSMO(Et_2_O)-ZORA-M06-2X/TZ2P//COSMO(Et_2_O)-ZORA-BLYP-D3(BJ)/DZP. Bond lengths (Å) of the transition state geometries are provided in the inset. All non-essential hydrogen atoms were removed from the three-dimensional molecular geometries for clarity.

Density functional theory (DFT) calculations were performed to elucidate the reaction mechanism and origins of diastereoselectivities and enantioselectivities using the ADF program (Fig. 4)^52^. The reaction probably proceeds through a two-step catalytic sequence; γ-deprotonation, and enantioselective α-reprotonation. An alternative mechanism, involving a non-selective ring strain-relief sequence followed by ACP desymmetrization, is unlikely as the product racemizes over extended reaction time (Supplementary Fig. 1). Two diastereomeric catalysts were considered in this computational study to provide detailed insight into the transformation.

First, we explored the reaction pathway of the cyclopropene esters using **24a** as an idealized substrate (Fig. 4). Initially, the catalyst **C11** and **24a** form a hydrogen-bonded complex **Int1**. We computed and compared all the possible transition state (TS) conformations for deprotonation and reprotonation processes (Supplementary Figs. 7–10). The lowest-energy transition structure **TS1-*Z*** for the initial deprotonation leads to the diastereoselective formation of dienolate intermediate **Int2**. Although the computed Gibbs free energy difference is slightly underestimated for the second-lowest transition state, **TS1-*E***, the large difference in electronic energies supports a deprotonation process that predominantly occurs through **TS1-*Z*** in a diastereoselective manner. Reprotonation occurs through **TS2-*R*** or **TS2-*S***, and the lowest energy transition state, **TS2-*R***, forms the (*R*)-product **P-1**, in agreement with the reaction’s experimentally confirmed absolute stereochemical outcome (ΔΔ*G*^‡^ = 4.2 kcal mol^−1^). The geometry of **TS2-*R*** benefits from a complementary fit of **24a** in the binding pocket of the catalyst in which intramolecular hydrogen bonding between the S(thiourea)–H(amide) fixes the conformational freedom of the ‘left arm’ of the BIMP catalyst. This creates a three-dimensionally defined pocket that embraces **24a** to minimize steric repulsions during the reprotonation step (Supplementary Fig. 11). In addition, there is a network of many intermolecular and intramolecular stabilizing interactions, including hydrogen bonding and CH–*π* interactions ranging in strength between −0.7 and −139.7 kcal mol^−1^ as quantified by our energy decomposition analysis (Supplementary Fig. 11)^53,54^.

We also investigated the reaction pathway for ketone-substituted substrates (Supplementary Scheme 6). Conformational analysis of the transition state structures revealed a preferred pathway that leads to the experimentally confirmed absolute stereochemical outcome. Notably, the rate-determining step shifts between the ester-substituted and ketone-substituted cyclopropenes, probably due to differences in the stability and reactivity of their respective dienolate intermediates.

In conclusion, an efficient, conceptually new and broad-scope method for the synthesis of chiral ACPs and cyclopropanes has been developed. Two BIMP catalysts provided excellent reactivity and selectivity across a broad range of substrates including esters, amides, ketones and a stand-alone phosphine oxide example. Through rational substrate design and retuning of the catalyst’s basic iminophosphorane moiety, the transformation was applied in the total synthesis of (1*R*,3*R*)-*cis*-permethrin, providing a new, enantioselective route to synthetically important insecticide cores. Finally, DFT studies elucidated the mechanism and origin of diastereoselectivities and enantioselectivities with the BIMP catalyst and their respective substrate classes. This powerful BIMP-catalysed transformation has provided access to highly attractive, previously unobtainable, enantioenriched ACPs and cyclopropanes through a strain-relief strategy, whose concept will inspire investigations into further transformations of small-ring carbocycles with BIMP catalysts.

## Online content

Any methods, additional references, Nature Portfolio reporting summaries, source data, extended data, supplementary information, acknowledgements, peer review information; details of author contributions and competing interests; and statements of data and code availability are available at 10.1038/s41586-025-09485-y.

## Supplementary information

This file contains Supplementary Sections 1–12, including Supplementary Figs. 1–14, Supplementary Tables 1–9 and Supplementary Schemes 1–6; see contents for details.

## Data Availability

Crystallographic data are available free of charge from the Cambridge Crystallographic Data Centre under reference CCDC 2342557 (**4b**), CCDC 2342558 (**9b**) and CCDC 2456302 (**27b**). Further optimization data, full synthetic methods and characterization data, and Cartesian coordinates and energies of all stationary points are available in the Supplementary Information.
