# Supplementary material for: Catalytic enantioselective synthesis of alkylidenecyclopropanes
Source: Nature. 2025 Aug 11;645(8082):932–8. doi: 10.1038/s41586-025-09485-y (PMC12460155; doi:10.1038/s41586-025-09485-y)
Supplement: Supplementary file 1 — This file contains Supplementary Sections 1–12, including Supplementary Figs. 1–14, Supplementary Tables 1–9 and Supplementary Schemes 1–6; see contents for details. [file 41586_2025_9485_MOESM1_ESM.pdf]

nature portfolio

https://doi.org/10.1038/s41586-025-09485-y

Supplementary information

Catalytic enantioselective synthesis of alkylidenecyclopropanes

In the format provided by the authors and unedited

Catalytic enantioselective synthesis of alkylidenecyclopropanes

Authors: Jonathan C. Golec‡[1,2], Dong-Hang Tan‡[1], Ken Yamazaki[1,3,4], Eveline H. Tiekink[3], Kirsten E. Christensen[1], Trevor A. Hamlin[3*], Darren J. Dixon[1*]

‡These authors contributed equally to this work

Affiliation: [1]Chemistry Research Laboratory, Department of Chemistry, University of Oxford, 12 Mansfield Road, Oxford, OX1 3TA, UK

[2]Sygnature Discovery, BioCity, Pennyfoot St, Nottingham, NG1 1GR, UK

[3]Department of Chemistry and Pharmaceutical Sciences, Amsterdam Institute of Molecular and Life Sciences (AIMMS), Vrije Universiteit Amsterdam, De Boelelaan 1108, 1081 HZ Amsterdam, The Netherlands

[4]Division of Applied Chemistry, Okayama University, Tsushimanaka, Okayama, 700-8530, Japan

*e-mail: darren.dixon@chem.ox.ac.uk, t.a.hamlin@vu.nl

Table of Contents

1. General Experimental *4*

2. Optimisation of Reaction Conditions *6*

2.1 Conditions screen for substrate 3a **6**

2.2 Investigation into the racemisation of compound 9b **7**

2.3 Catalyst screen for amide substrate 26a **7**

2.4 Investigation into the racemisation of compound 27b **8**

2.5 Conditions screen for ketone substrate 4a **9**

2.6 Conditions screen for substrate 33a **12**

3. General Procedures *13*

4. Catalyst Synthesis *18*

5. Synthesis and Characterisation of Substrates used in the Enantioselective Deconjugation of Cyclopropenes *32*

5.1 Synthesis of cyclopropene-substrate precursors **32**

5.2 Synthesis of ester substrates **48**

5.3 Synthesis of amide substrates **73**

5.4 Synthesis of ketone substrates **79**

5.6 Synthesis of ether substrates **85**

6. Synthesis and Characterisation of Products Obtained in the Enantioselective Deconjugation of Cyclopropenes *100*

6.1 Synthesis of enantioenriched cyclopropane esters **100**

6.2 Synthesis of enantioenriched cyclopropane amides and phosphine oxides 126

6.3 Synthesis of enantioenriched cyclopropane ketones 136

6.4 Synthesis of enantioenriched insecticide cores 140

7. Derivatisation 151

8. Kinetic Isotope Effect Studies 153

9. NMR Spectra 164

10. HPLC and SFC Traces 280

11. Computational Studies 321

11.1 Computational Methods 321

11.2 Computational Results 322

11.3 Computational Details 330

11. Single Crystal X-Ray Diffraction Data 438

12. References 444

1. General Experimental

Atmosphere

All reactions were carried out under an atmosphere of Ar or N_2 unless otherwise stated.

Solvents

Moisture sensitive reactions were carried out using solvents obtained from the MBRAUN-SPS solvent purification system (CH_2Cl_2, THF, pentane, toluene, DMF, Et_2O) and often dried over 3Å molecular sieves. Reactions that were not deemed moisture sensitive were carried out using solvents taken from Winchester bottles. For reactions sensitive to oxygen solvents were degassed with Ar.

Chromatography

TLCs were carried out using Merck aluminium backed DC60 F254 plates (particle size 0.2 mm). UV light was used to visualise spots which were often stained with $KMnO_4$, vanillin or ninhydrin depending on the compound. Flash column chromatography was carried out using Sigma Aldrich silica gel 60 Å (particle size 43-60 μm) with the indicated solvent system.

Spectroscopy

Proton (^1H), carbon (^{13}C), phosphorus (^{31}P) and (^{19}F) spectra were recorded on Bruker DPX200 (200 MHz) Bruker AVG400 (400/101 MHz), Bruker AVH400 (400/101 MHz), Bruker AVF400 (400/101 MHz), Bruker AVC500 (500/126 MHz), Bruker AVB500 (500/126 MHz), Bruker AVX500 (500/126 MHz), Bruker NEO600 (600/151) and Bruker AV600 (600/151) NMR spectrometers. Spectra were referenced with respect to the residual solvent peak ($CHCl_3$: δ_H 7.26, δ_C 77.16 ppm; C_6D_5H: δ_H 7.16, δ_C 128.06 ppm; CD_3COCD_2H: δ_H 2.05, δ_C 29.84 ppm; CD_2HCN: δ_H 1.94, δ_C 1.32 ppm; $C_6D_4HCD_3$: δ_H 7.09, δ_C 125.13 ppm; CHD_2OD: δ_H 3.31, δ_C 49). Peak assignments were made based on chemical shifts, integrations, coupling constants,

2-D COSY and HSQC, HMBC and NOESY. Peak multiplicities are described as singlet (s), doublet (d), triplet (t), pentet (p), or a combination e.g. doublet of doublets, or as a multiplet (m) over a peak range. Some peaks are described as broad (b). Coupling constants are reported to the nearest 0.5 Hz and ^{13}C chemical shifts are given to the nearest 0.1 ppm.

Mass spectrometry

High-resolution mass spectra (ESI) were recorded using a Bruker µTOF mass spectrometer.

Melting points

Melting points (MP) were obtained using a Leica Galen III Hot-stage melting point apparatus and microscope and on a Kofler hot block and are reported uncorrected.

Infrared

Infrared spectra (IR) were recorded using a Bruker Tensor 27 FT-IR spectrometer as a film or powder sample. Selected maximum absorbances were reported in ν_{max} (cm^{-1}).

X-Ray

Low temperature single crystal X-ray diffraction data for **4b**, **9b** and **27b** were collected using a Rigaku Oxford SuperNova diffractometer. Raw frame data were reduced using CrysAlisPro. All structures were solved using 'Superflip' before refinement with CRYSTALS as per the SI (CIF).[1-3] Full refinement details are given in the Supporting Information (CIF). Crystallographic data have been deposited with the Cam-bridge Crystallographic Data Centre (CCDC 2342557-58) and can be obtained via www.ccdc.cam.ac.uk/data_request/cif

Compound naming

Compound names are those generated by ChemDraw software following the IUPAC nomenclature.

2. Optimisation of Reaction Conditions

2.1 Conditions screen for substrate 3a

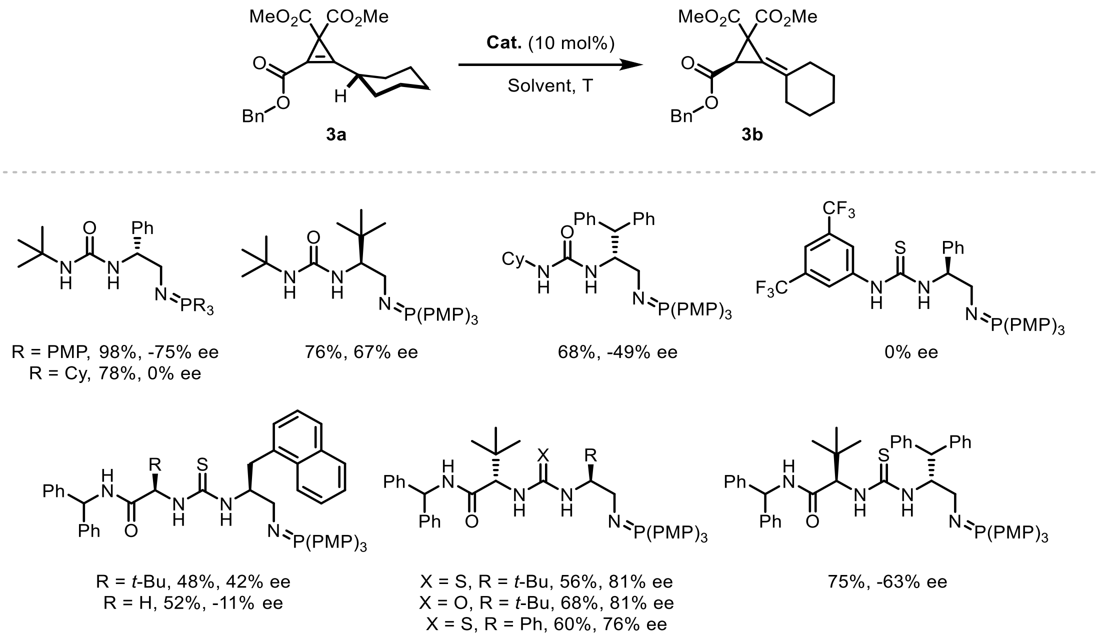

Scheme S1: Catalyst screen for the enantioselective deconjugation of model substrate **3a**.

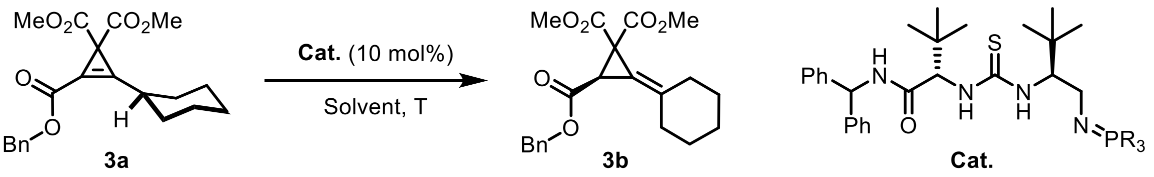

entry	*phosphine*	*solvent*	*temp / °C*	*conc / M*	*time / h*	*yield / %*	*ee / %*
**1**	$P(PMP)_3$	THF	rt	0.1	4	56	81
**2**	$P(PMP)_3$	THF	50	0.1	2	60	78
**3**	$P(PMP)_3$	Et_2O	rt	0.05	2	76	84
**4**	$P(PMP)_3$	Et_2O	-22	0.05	23	>99	84
**5**	PPh_3 (**C11**)	Et_2O	rt	0.05	4	99	97

Table S1: Conditions screen for the enantioselective deconjugation of cyclopropenes iterations of catalyst **C11** and substrate **3a**.

2.2 Investigation into the racemisation of compound 9b

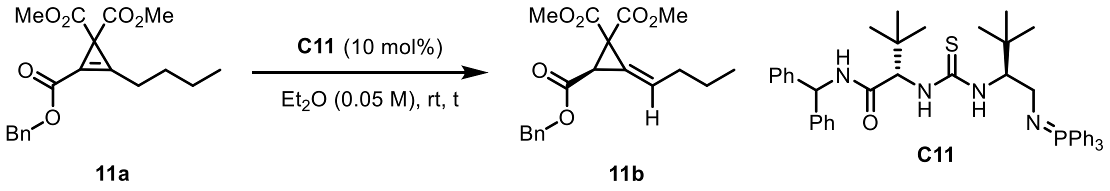

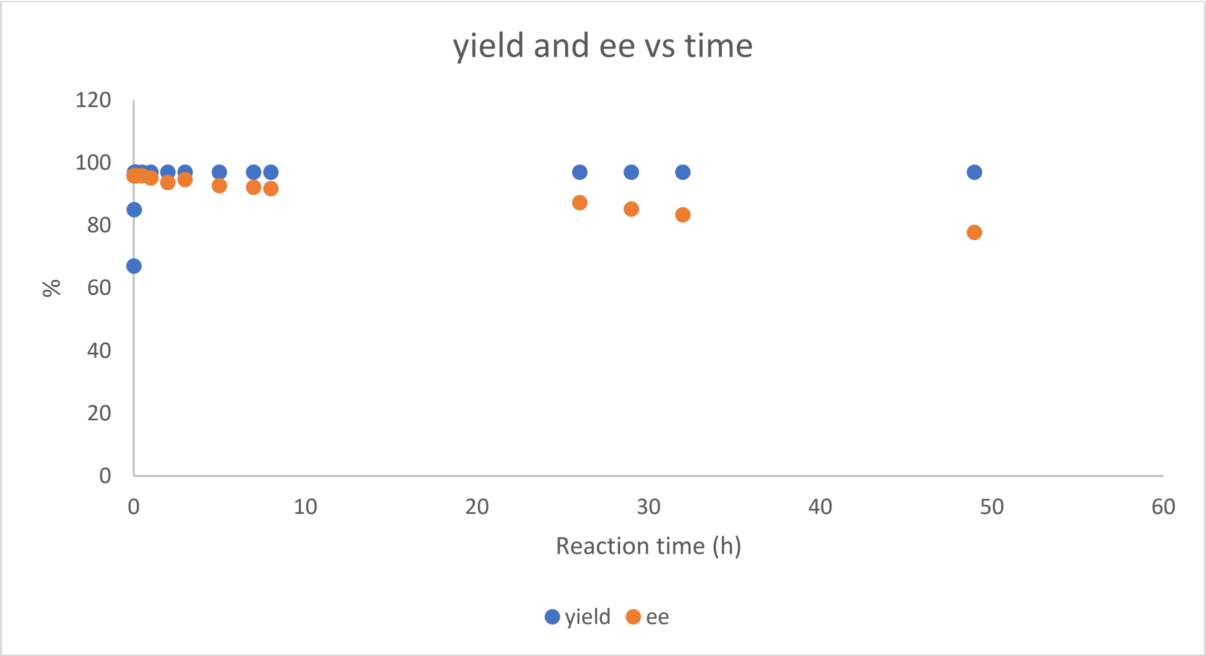

Figure S1: Plot of yield and ee vs time for the BIMP-catalysed enantioselective deconjugation of compound **9a** with catalyst **C11.**

2.3 Catalyst screen for amide substrate 26a

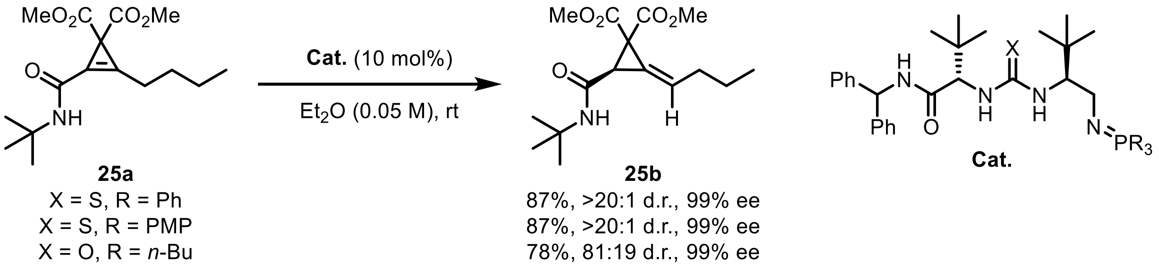

Scheme S2: Enantioselective deconjugation of amide **26a** with iterations of catalyst **C11**.

2.4 Investigation into the racemisation of compound 27b

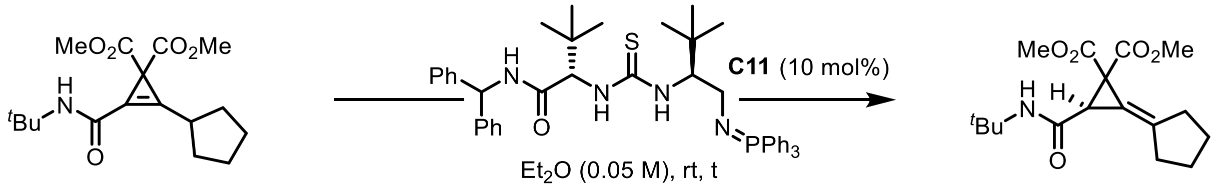

27a **27b**

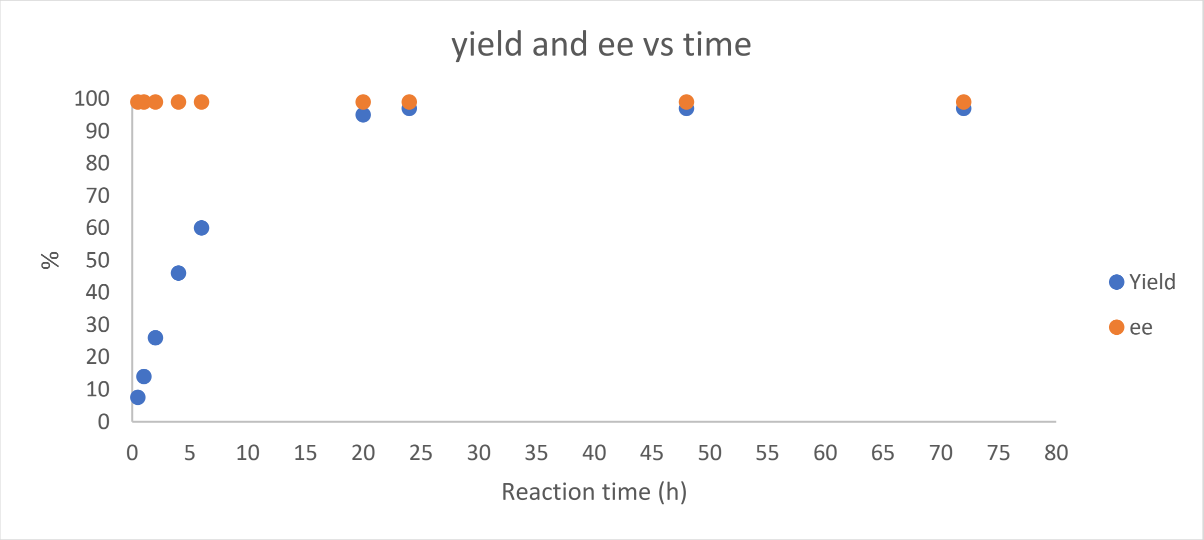

Figure S2: Plot of yield and ee vs time for the BIMP-catalysed enantioselective deconjugation of compound **27a** with catalyst **C11.**

2.5 Conditions screen for ketone substrate 4a

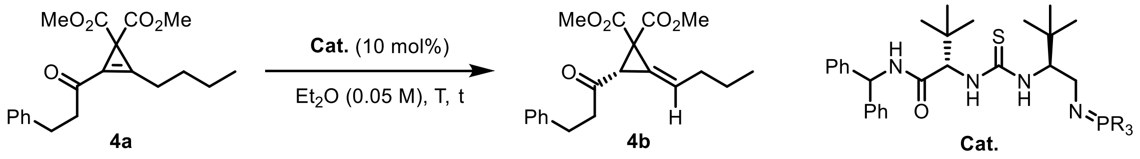

entry	*phosphine*	*time / min*	*yield / %*	*d.r. (Z:E)*	*ee (Z) / %*
**1**	PPh_3	15	84	>20:1	20
**2**	$PPh_2((p\text{-}CF_3)C_6H_4)$	10	90	>20:1	4
**3**	$PPh_2((p\text{-}CN)C_6H_4)$	10	78	>20:1	4
**4**	$PPh((p\text{-}CF_3)C_6H_4)_2$	10	85	N/D	-10
**5**	$P((p\text{-}CF_3)C_6H_4)_3$	43	84	N/D	-20

Table S2: Phosphine screen for the enantioselective deconjugation of ketone **4a**.

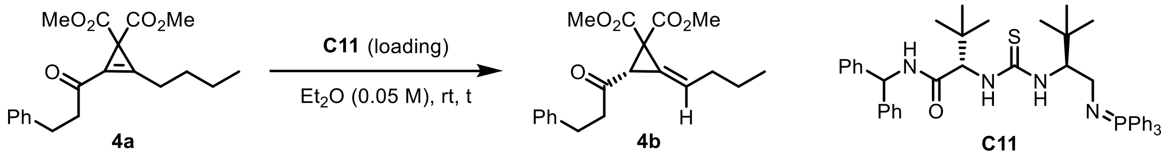

entry	*CL / mol%*	*time / min*[a]	*ee (Z) / %*
**1**	10	15	20
**2**	0.5	40	22
**3**	0.5	42	22
**4**	0.5	20	23
**5**	0.1	180	23

Table S3: Investigations into the catalyst loading for the enantioselective deconjugation of substrate **4a**. [a]Time to 100% consumption of starting material as observed by TLC.

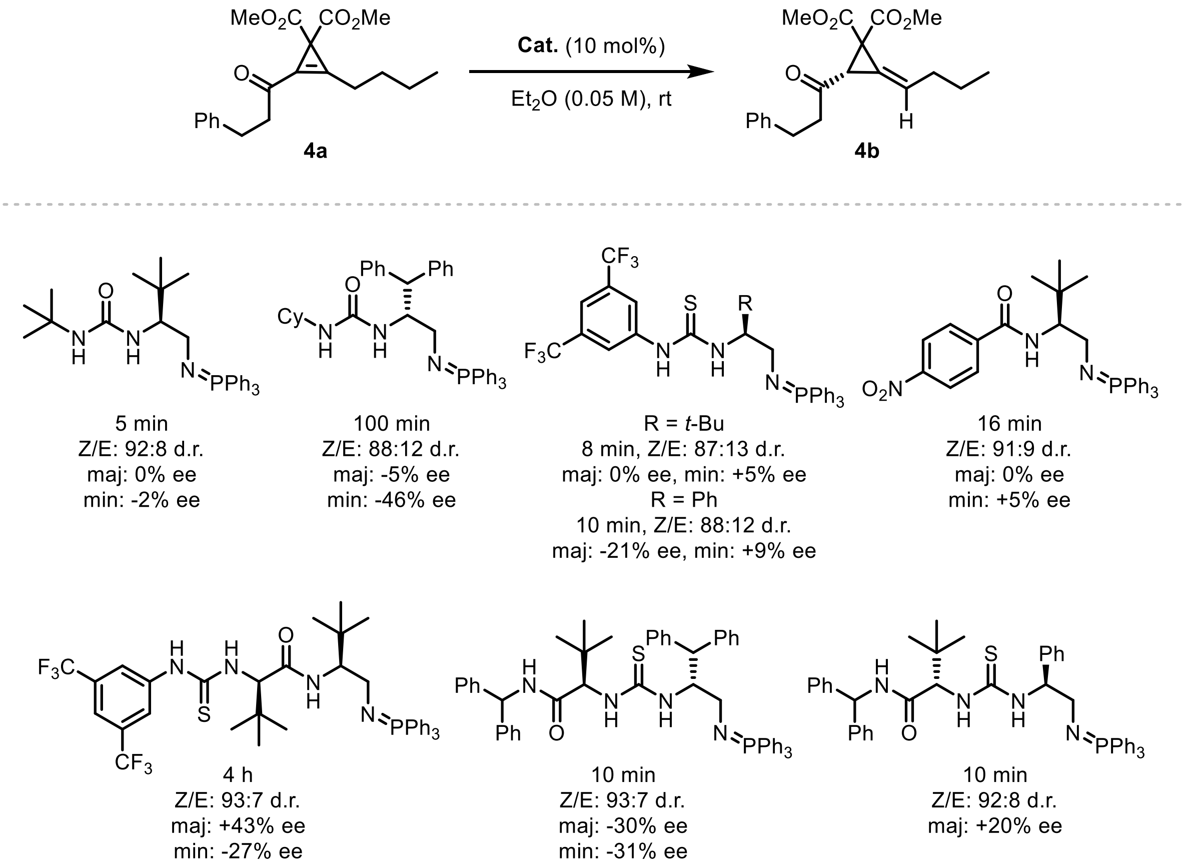

Scheme S3: Enantioselective deconjugation of substrate **4a** with catalysts. [a]Time to 100% consumption of starting material as observed by TLC.

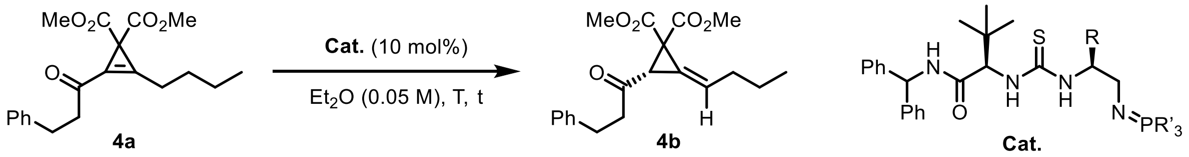

entry	*R*	*phosphine*	*temp / °C*	*time / min*[a]	*Z/E (d.r.)*	*ee maj (Z)/%*	*ee min (E)/%*
**1**	CH_2(1-Np)	PPh_3	rt	180	76:24	+39	+46
**2**	CH_2(1-Np)	PPh_2(*p*-CNC_6H_4)	rt	450	83:17	+28	+50
**3**	CH_2(1-Np)	PPh_2(*p*-$CF_3C_6H_4$)	rt	250	81:19	+24	+60
**4**	Ph	PPh_3	rt	180	92:8	+31	+33
**5**	Bn	PPh_3	rt	90	76:24	+48	+57
**6**	Cy	PPh_3	rt	90	87:13	+55	N/D
**7**	*t*-Bu	PPh_3	rt	120	20:1	0	+31
**8**	*t*-Bu	PPh_3	rt	5	20:1	+24	>+99
**9**	*t*-Bu	PPh_3	rt	0.5	>20:1	+65	>+99
**10**	*t*-Bu	PPh_3	-78	60[b]	>20:1	+94	N/D
**11**	*t*-Bu	PPh_3	-78	120[c]	>20:1	+93	N/D

Table S4: Catalyst, phosphine and conditions screen for the enantioselective deconjugation of substrate **4a**. [a]Time to 100% consumption of starting material as observed by TLC. [b]93% NMR yield. [c]99% yield.

2.6 Conditions screen for substrate 33a

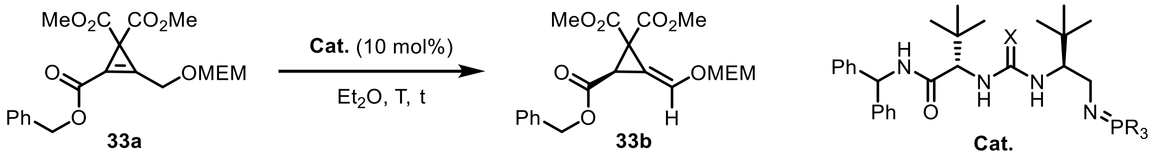

entry	*X*	*phosphine*	*T / °C*	*conc / M*	*time / h*	*conversion / %*	*d.r. (Z/E)*	*ee maj / %*	*ee min / %*
**1**	S	PPh_3	rt to 40	0.05	24	17	N/D	N/D	N/D
**2**	S	$P(PMP)_3$	rt	0.05	54	59	76:24	70	5
**3**	O	P(*n*-Bu)$_3$	rt	0.05	1.0	88[a]	11:89	0	60
**4**	O	P(*n*-Bu)$_3$	-78 to -20	0.05	24	25	12:88	0	0
**5**	S	$P(PMP)_3$	rt	0.2	2.0	100	16:84	4	32
**6**	O	$P(PMP)_3$	rt	0.05	2.0	100	64:36	75	6
**7**	O	PPh_3	rt	0.05	24	12	90:10	88	19
**8**	O	PPh_2(*p*-$CF_3C_6H_4$)	rt	0.05	24	4	90:10	87	6
**9**	O	PPh_2(*p*-CNC_6H_4)	rt	0.05	24	30	>20:1	87	12
**10**	O	P(*p*-$MeC_6H_4)_3$	rt	0.05	24	18	74:26	84	4
**11**	O	P((*m*-*t*-Bu)$_2C_6H_3)_3$	rt	0.05	0.75	100	>20:1	80	32
**12**	O	P((*m*-*t*-Bu)$_2C_6H_3)_3$	-20	0.05	34	100	>20:1	87	30
**13**	S	P((*m*-*t*-Bu)$_2C_6H_3)_3$	rt	0.05	1.0	100	>20:1	98	56

Table S5: Phosphines and conditions screen for the enantioselective deconjugation of compound **33a**.[a]Isolated yield

3. General Procedures

General procedure I for *in situ* generation of active BIMP catalysts

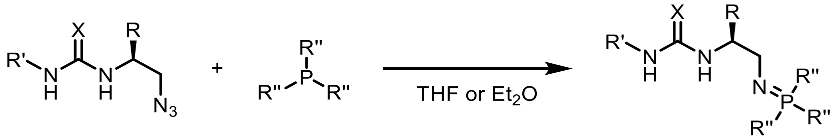

To a mass spectrometry vial was weighed the desired azide (1.00 eq) and the desired phosphine (1.00 eq) (in the case of electron poor phosphines 1.50 eq of the phosphine was used and the reaction was heated to 40 °C, this alternate procedure was also followed when reactions were carried out on small scale ≤0.025 mmol). The vial was placed under an atmosphere of Ar and THF (0.2 M) was added. The cap was replaced and sealed with parafilm® and the mixture was stirred for 18 – 24 h. The volatiles were removed under reduced pressure or under a constant stream of Ar an the crude iminophosphorane was used without further purification.

General procedure II for the enantioselective deconjugation of cyclopropenes (esters and amides)

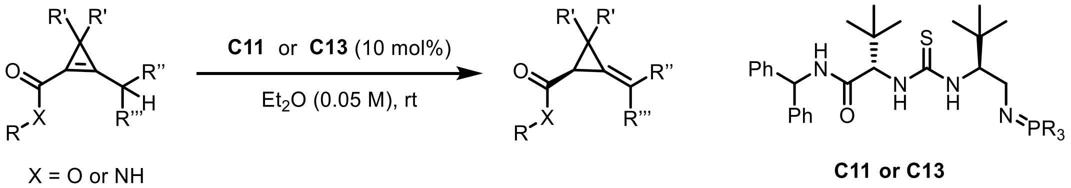

The desired cyclopropene (1.0 eq) was transferred in a solution of Et_2O (0.05 M) to preformed BIMP catalyst **C11** or **C13** (10 mol%) (see general procedure **I**) or vice versa depending on the reaction scale. The reaction was monitored by TLC and quenched with AcOH (20 mol%, stock solution in Et_2O) upon completion (care must be taken to avoid racemisation of the ACP if the reaction is left for extended time periods. Formation of tertiary ACPs appeared to form more rapidly than quaternary ACPs which in turn formed quicker than secondary ACPs). The crude reaction mixture was concentrated under a stream of N_2 and purified by silica gel column chromatography to afford the desired enantioenriched ACP. Racemic versions of the

compounds could be synthesised by addition of DBU (3.0 eq) to a stirred solution of the cyclopropene in THF (0.1 M). Racemic compounds were also synthesised using ***rac*-C11** or ***rac*-C13** (formed using an equimolar amount of the two catalyst enantiomers). AS-H and AD-H columns appear best for separating all diastereomers and starting material.

General procedure III for the enantioselective deconjugation of cyclopropenes (ketones)

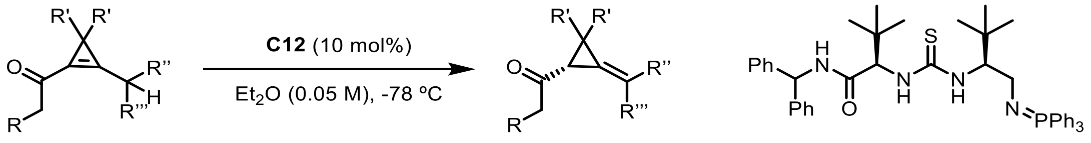

C12

A solution of preformed BIMP catalyst **C12** (10 mol%) in Et_2O (0.1 M wrt cyclopropene) was added dropwise to a stirred solution of the desired cyclopropene (1.0 eq) in Et_2O (0.1 M) at −78 °C The reaction was monitored by TLC and quenched with AcOH (20 mol%, stock solution in Et_2O) upon completion. The crude reaction mixture was concentrated under a stream of N_2 and purified by silica gel column chromatography to afford the desired enantioenriched ACP. Racemic versions of the compounds could be synthesised by addition of DBU (3.0 eq) to a stirred solution of the cyclopropene in THF (0.1 M). AS-H and AD-H columns appear best for separating all diastereomers and starting material.

General procedure IV for the $Rh_2(OAc)_4$ addition of diazonium compounds to terminal alkynes

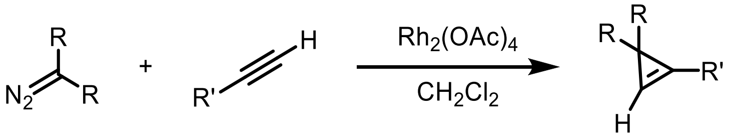

According to a literature procedure,[4] the desired diazonium compound (1.0 eq) in CH_2Cl_2 (0.5 M) was added dropwise overnight using a syringe pump to a solution of $Rh_2(OAc)_4$ (1.0 mol%) in the desired terminal alkyne (3.0 eq). Following the addition, the reaction was monitored by TLC and further equivalents of alkene and $Rh_2(OAc)_4$ were added until complete disappearance

of the diazonium compound was observed. The crude product was flushed through a plug of celite with CH_2Cl_2 and purified by silica gel column chromatography to provide the desired terminal cyclopropene.

General procedure V for the addition of terminal cyclopropenes to electrophiles

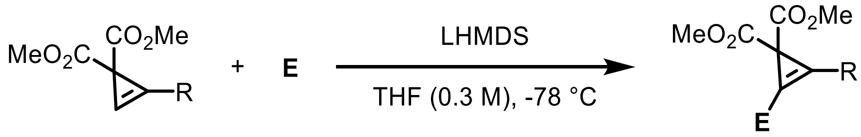

LHMDS (1.0 M in THF, 1.0 eq) was added dropwise to a stirred solution of the desired terminal cyclopropene (1.0 eq) and electrophile (1.5 eq) at −78 °C in THF (0.3 M). The solution was stirred at −78 °C until completion was observed by TLC. The reaction was quenched with sat. aq. NH_4Cl and the aqueous phase was extracted with Et_2O (3 x 20 ml) (if an isocyante was used the reaction was quenched with 1.0 M $HCl_{(aq)}$). The combined organic extracts were washed with brine then dried over Na_2SO_4, filtered and concentrated *in vacuo*. The crude material was purified by silica gel column chromatography to provide the desired cyclopropene.

General procedure VI for the addition of dibromocarbenes to alkenes

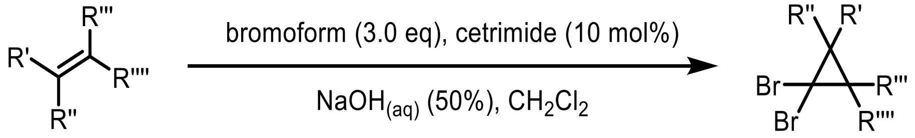

According to a literature procedure,[5] 50% NaOH (2.5 x CH_2Cl_2) was added dropwise via a syringe pump (10 ml/h) to a stirred solution of the desired alkene (1.0 eq), cetrimide (10 mol%) and bromoform (3.0 eq) in CH_2Cl_2 (10 M) at ambient temperature. The reaction was stirred and monitored by TLC until complete disappearance of the alkene was observed (1-5 days). To aid reaction progression extra equivalents of bromoform and NaOH solution were added and in some cases the reaction was warmed to 50 °C. Upon completion of the reaction H_2O was added and the organic layer separated. The aqueous layer was extracted with CH_2Cl_2 (2 x), the combined organic layers were dried over Na_2SO_4 (addition of brine is not recommended as

it results in precipitation of the phase transfer catalyst) and concentrated *in vacuo*. The crude reaction mixture was purified by silica gel column chromatography to provide the desired tribromocyclopropane. Excess bromoform may be removed under a stream of nitrogen.

General procedure VII for the addition of tribromocyclopropanes to electrophiles

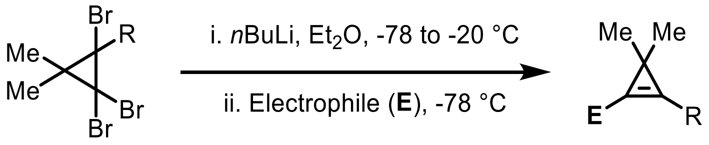

According to a modified literature procedure,[6] *n*-BuLi (2.5 M in hexanes, 2.0 eq) was added dropwise to a stirred solution of the desired tribromocyclopropane (1.0 eq) in Et_2O (0.1 M) at −78 °C. The reaction was stirred at −78 °C for 1 h at which point the temperature was raised to −20 °C and stirred for a further 2 h. The reaction mixture was then cooled to −78 °C and decanted via cannula at that temperature into a −78 °C solution of the desired electrophile (1.5 eq) (in cases where 1.2 eq of chloroformate is used, the chloroformate was added directly to the lithiated cyclopropene solution) in Et_2O (1.0 M). The reaction mixture was stirred until completion was observed by TLC at which point the reaction was quenched with sat. aq. NH_4Cl and the aqueous phase was extracted with Et_2O (3 x 20 ml) (if an isocyante was used the reaction was quenched with 1.0 M $HCl_{(aq)}$). The combined organic extracts were washed with brine then dried over Na_2SO_4, filtered and concentrated *in vacuo*. The crude material was purified by silica gel column chromatography to provide the desired cyclopropene.

General procedure VIII for the oxidation of alcohols

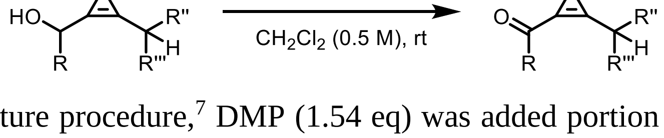

According to a literature procedure,[7] DMP (1.54 eq) was added portion wise to a solution of the desired alcohol (1.0 eq) in CH_2Cl_2 (0.5 M). The reaction was monitored by TLC until complete disappearance of the alcohol was observed (ca. 5 min). Upon completion sat. aq.

$NaHCO_3$ was added. The aqueous layer was extracted with Et_2O (3 x) and the combined organic layers were washed with brine, dried over Na_2SO_4 and concentrated *in vacuo*. The crude reaction mixture was purified by silica gel column chromatography to provide the desired ketone.

4. Catalyst Synthesis

Synthesis and characterisation of S1

(R)-N-(1-azido-3,3-dimethylbutan-2-yl)-2,2,2-trifluoroacetamide (S1)

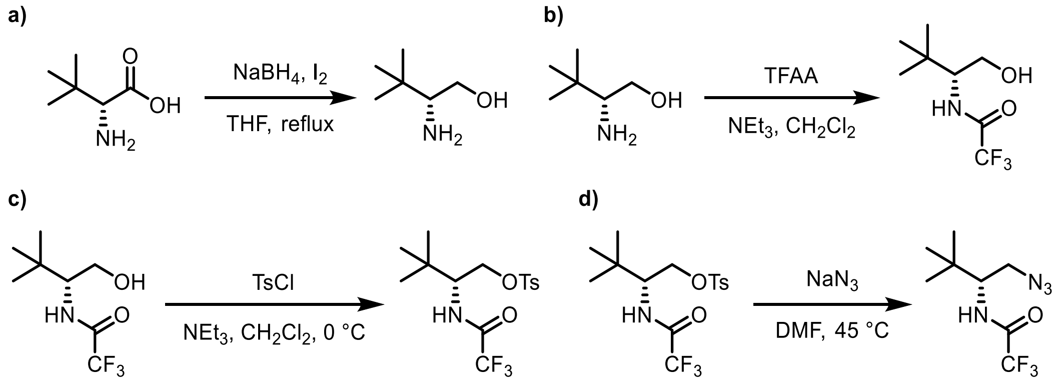

a) According to a literature procedure,[8] a solution of I_2 (9.67g, 38.1 mmol, 1.00 eq) in THF (22.4 ml) was added to a stirred solution of D-*tert*-leucine (5.00 g, 38.1 mmol, 1.00 eq) and $NaBH_4$ (3.46 g, 91.5 mmol, 2.40 eq) in THF (95.3 ml) at 0 °C. The resulting solution was warmed to ambient temperature over 15 min and then refluxed overnight. The solution was then cooled to 0 °C and MeOH was added until the solution turned colourless. The solution was then stirred for a further 30 min at which point the volatiles were removed *in vacuo*. The resulting paste was dissolved in 20% aq KOH and stirred for 3 h. The aqueous layer was extracted with CH_2Cl_2 (3 x 100 ml). The combined organic extracts were washed with brine, dried over Na_2SO_4, filtered and concentrated *in vacuo* to provide the crude amino-alcohol which was taken on to the next step without further purification.

b) According to a literature procedure,[8] trifluoroacetic anhydride (5.56 ml, 40.0 mmol, 1.05 eq) was added over 40 min to a stirred solution of the crude product obtained in step **a** and triethylamine (6.38 ml, 45.7 mmol, 1.20 eq) in CH_2Cl_2 (127 ml). The reaction was stirred for 2 h at which point H_2O (100 ml) was added. The aqueous phase was extracted with CH_2Cl_2 (3 x 100 ml). The combined organic extracts were washed with brine, dried over Na_2SO_4, filtered and concentrated *in vacuo*. The crude product was purified by silica gel column

chromatography (pentane/EtOAc = 8/2 to 6/4) to provide the product as a white solid in 55% yield (4.45 g). **^{1}H NMR** (400 MHz, $CDCl_3$) δ(ppm): 6.52 (bs, 1H, N$\underline{H}$), 3.92 – 3.82 (m, 2H, C$\underline{H}$C$(CH_3)_3$ and one of C$\underline{H_A}$$H_B$OH), 3.75 – 3.66 (m, 1H, one of C$H_A$$\underline{H_B}$OH), 1.95 (s, 1H, O$\underline{H}$), 0.98 (s, 9H, C(C$\underline{H_3}$)$_3$); **^{13}C NMR** (101 MHz, $CDCl_3$) δ(ppm): 158.3 ($\underline{C}$=O), 117.6 ($\underline{C}F_3$), 61.7 ($\underline{C}H_2$OH), 59.7 ($\underline{C}HCH_2$), 34.2 ($\underline{C}(CH_3)_3$), 26.6 (C($\underline{C}H_3$)$_3$); **^{19}F NMR** (377 MHz, $CDCl_3$) δ(ppm): -75.7 (C$\underline{F_3}$); **IR** (powder) v_{max}/cm^{-1}: 3401 (O-H), 3233 (N-H), 2972 (C-H), 1700 (C=O); **HRMS** (ES+) exact mass calculated for $[M+Na]^+$ ($C_8F_3H_{14}NNaO_2^+$) requires *m/z* 236.0716, found *m/z* 236.0719; **MP**: 96 – 98 °C; $[\boldsymbol{\alpha}]_D^{26.1}$ = +6.4 (*c* 0.39, $CHCl_3$).

c) According to a literature procedure,[8] the crude product obtained in step **b** was dissolved in CH_2Cl_2 (35 ml) and triethylamine (35 ml). The solution was cooled to 0 °C and TsCl (4.41 g, 23.1 mmol, 1.10 eq) was added. The reaction was stirred at 0 °C overnight at which point H_2O (100 ml) was added. The aqueous layer was extracted with CH_2Cl_2 (3 x 100 ml). The combined organic extracts were washed with brine, dried over Na_2SO_4, filtered and concentrated *in vacuo*. The crude product was taken on without further purification.

d) According to a literature procedure,[8] the crude product obtained in step **c** (6.64 g, 18.1 mmol, 1.00 eq) was dissolved in DMF (60 ml). Sodium azide (2.35 g, 36.2 mmol, 2.00 eq) was added and the reaction was warmed to 45 °C behind a blast shield. The reaction was stirred at 45 °C overnight at which point H_2O (100 ml) was added. The aqueous layer was extracted with Et_2O (3 x 100 ml). The combined organic extracts were washed with brine, dried over Na_2SO_4, filtered and concentrated *in vacuo*. The crude product was purified by silica gel column chromatography (pentane/EtOAc = 9/1) to provide the title compound (**S1**) as a white solid in 43% yield (1.86 g). **^{1}H NMR** (400 MHz, $CDCl_3$) δ(ppm): 6.27 (bs, 1H, N$\underline{H}$), 4.04 – 3.93 (m, 1H, C$\underline{H}$NH), 3.63 (dd, *J* = 13.0, 4.0 Hz, 1H, one of C$\underline{H_A}$$H_B$$N_3$), 3.40 (dd, *J* = 13.0, 8.0 Hz, 1H, one of C$H_A$$\underline{H_B}$$N_3$), 0.98 (s, 9H, C(C$\underline{H_3}$)$_3$); **^{13}C NMR** (101 MHz, $CDCl_3$) δ(ppm): 160.5 – 155.3 (m, $\underline{C}$=O), 118.4 – 113.6 (m, $\underline{C}F_3$), 57.3 ($\underline{C}$HNH), 51.2 (C$\underline{H_2}$$N_3$), 34.3 ($\underline{C}(CH_3)_3$), 26.6

$(C(\underline{C}H_3)_3)$; **^{19}F NMR** (376 MHz, $CDCl_3$) δ(ppm): -75.8 ($C\underline{F_3}$); **IR** (powder) v_{max}/cm^{-1}: 3283 (N-H), 2971 (C-H), 2092 (N=N=N), 1712 (C=O); $[\boldsymbol{\alpha}]_{\boldsymbol{D}}^{\boldsymbol{26.1}}$ = +46.0 (c 1.06, $CHCl_3$). Data are consistent with that published in the literature.[8]

Synthesis and characterisation of S2

(R)-2-amino-N-benzhydryl-3,3-dimethylbutanamide hydrochloride (S2)

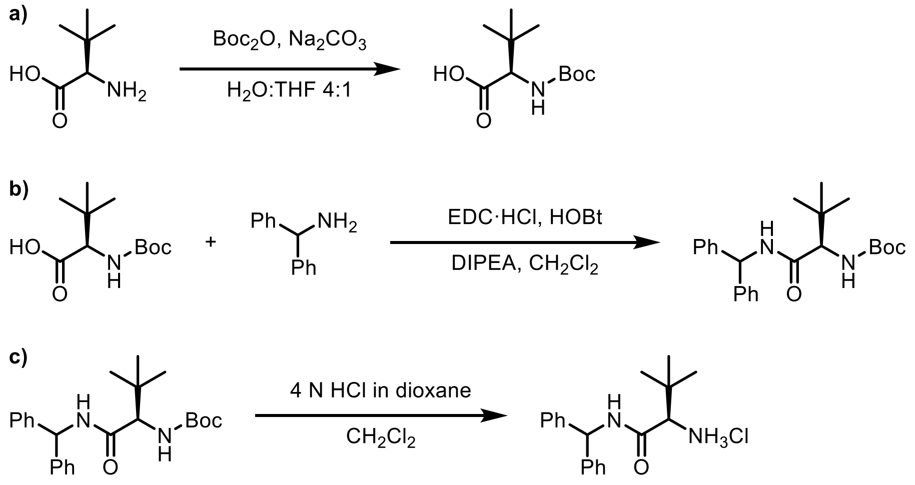

a) According to a literature procedure,[9] D-*tert*-leucine (3.00 g, 22.9 mmol, 1.00 eq), Boc_2O (5.99 g, 27.5 mmol, 1.20 eq) and Na_2CO_3 (4.85 g, 45.7 mmol, 2.00 eq) were stirred overnight in THF (11.4 ml) and H_2O (45.7 ml). The solution was brought to pH 2 by careful addition of HCl (10% aq solution). The aqueous phase was extracted with EtOAc (3 x 50 ml). The combined extracts were washed with brine, dried over Na_2SO_4, filtered, and concentrated *in vacuo* to afford the crude product which was taken on without further purification (5.21 g).

b) According to a literature procedure,[10] DIPEA (4.36 ml, 5.88 mmol, 1.50 eq) and benzhydrylamine (4.27 ml, 24.8 mmol, 1.10 eq) were added sequentially to a stirred solution of EDC hydrochloride (4.75 g, 24.8 mmol, 1.10 eq) and HOBt (3.34 g, 24.8 mmol, 1.10 eq) in CH_2Cl_2 (133 ml) under a N_2 atmosphere at room temperature. *N*-boc-D-*tert*-leucine (5.21 g, 22.5 mmol, 1.00 eq) was added in one portion and the resulting solution stirred for 20 h. The solution was diluted with Et_2O (200 ml) and washed with HCl (0.5 N, 2 x 100 ml). The aqueous phase was extracted with Et_2O (3 x 100 ml) and the combined organic phases were washed with sat. aq. $NaHCO_3$ (100 ml) and brine, dried over $MgSO_4$ and concentrated *in vacuo* to afford the crude product (7.82 g); this was taken on without further purification.

c) According to a literature procedure,[10] 4 N HCl in 1,4-dioxane (47.3 ml, 89.3 mmol, 9.60 eq) was added to a vigorously stirred solution of the crude product obtained in step **b** in CH_2Cl_2 (49.3 ml) under a N_2 atmosphere at room temperature over a period of 5 min. The resulting solution was stirred overnight and concentrated under a stream of N_2. The resulting precipitate was washed with Et_2O and filtered to afford the product crude product (6.35 g). The crude product (**S2**) was taken on without further purification.

Synthesis and characterisation of S3

(S)-2-amino-N-benzhydryl-3,3-dimethylbutanamide hydrochloride (S3)

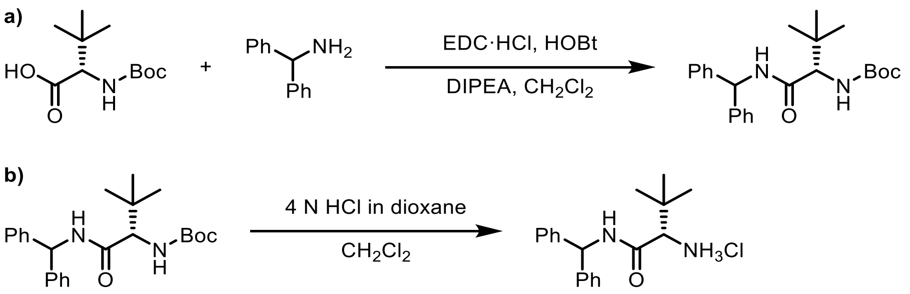

a) According to a literature procedure,[10] DIPEA (11.4 ml, 65.3 mmol, 1.50 eq) and benzhydrylamine (8.21 ml, 47.6 mmol, 1.10 eq) were added sequentially to a stirred solution of EDC hydrochloride (9.12 g, 47.6 mmol, 1.10 eq) and HOBt (6.43 g, 47.6 mmol, 1.10 eq) in CH_2Cl_2 (254 ml) at ambient temperature. *N*-boc-L-*tert*-leucine (10 g, 43.2 mmol, 1.00 eq) was added in one portion and the resulting solution stirred for 20 h. The solution was diluted with Et_2O (200 ml) and washed with HCl (0.5 N, 2 x 200 ml). The aqueous phase was extracted with Et_2O (3 x 200 ml) and the combined organic phases were washed with sat. aq. $NaHCO_3$ (200 ml), brine, dried over $MgSO_4$ and concentrated *in vacuo* to afford the crude product which was taken on without further purification.

b) According to a literature procedure,[10] 4 N HCl in 1,4-dioxane (104 ml, 415 mmol, 9.60 eq) was added to a vigorously stirred solution of the crude product obtained in step **a** in CH_2Cl_2 (108 ml) under a N_2 atmosphere at room temperature over a period of 5 min. The resulting solution was stirred overnight and concentrated under a stream of N_2 to afford the product crude product (**S3**) (12.0 g). The crude product was taken on without further purification.

Synthesis and characterisation of S4

(R)-N-benzhydryl-2-isothiocyanato-3,3-dimethylbutanamide (S4)

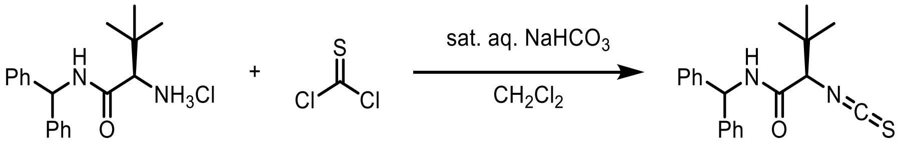

According to a literature procedure,[10] sat. aq. $NaHCO_3$ (90 ml) was added to a vigorously stirred solution of (*R*)-2-amino-N-benzhydryl-3,3-dimethylbutanamide hydrochloride (**S2**) (3.00 g, 9.01 mmol, 1.00 eq) in CH_2Cl_2 (90 ml) under a N_2 atmosphere at 0 °C and the biphasic mixture was stirred for 20 min. Stirring was stopped and thiophosgene (0.69 ml, 9.01 mmol, 1.00 eq) added to the organic layer. Vigorous stirring was recommenced immediately after addition and the mixture allowed to warm to room temperature over 30 min. Stirring was stopped, and the organic phase was extracted with CH_2Cl_2, washed with brine and dried over $MgSO_4$. The volatiles were removed *in vacuo* to give the crude product (**S4**) as a yellow solid (2.57 g) which was taken on without further purification (84%). **^{1}H NMR** (400 MHz, $CDCl_3$) δ(ppm): 7.32 – 7.19 (m, 6H, Ar<u>H</u>), 7.19 – 7.11 (m, 4H, Ar<u>H</u>), 6.54 (d, *J* = 8.0 Hz, 1H, N<u>H</u>), 6.17 (d, *J* = 8.0 Hz, 1H, C<u>H</u>Ph_2), 4.00 (s, 1H, C<u>H</u>C(=O)), 0.99 (s, 9H, C(C<u>H</u>$_3$)$_3$); **^{13}C NMR** (101 MHz, $CDCl_3$) δ(ppm): 165.5 (<u>C</u>(=O)), 140.9 (Ar<u>C</u>), 140.6 (Ar<u>C</u>), 137.3 (N=<u>C</u>=S), 128.8 (Ar<u>C</u>H), 128.8 (Ar<u>C</u>H), 127.8 (Ar<u>C</u>H), 127.8 (Ar<u>C</u>H), 127.5 (Ar<u>C</u>H), 127.3 (Ar<u>C</u>H), 71.0 (<u>C</u>HC(=O)), 57.3 (<u>C</u>H(Ph)$_2$), 37.1 (<u>C</u>H(CH_3)$_3$), 26.8 (CH(<u>C</u>H$_3$)$_3$); **IR** (powder) ν_{max}/cm^{-1}: 3295 (N-H), 2980, 2971, 2889 (C-H), 1659 (C=O); $[\alpha]_D^{26}$ = –41.8 (*c* 1.06, $CHCl_3$). Data are consistent with that published in the literature.[11]

Synthesis and characterisation of S5

(S)-N-benzhydryl-2-isothiocyanato-3,3-dimethylbutanamide (S5)

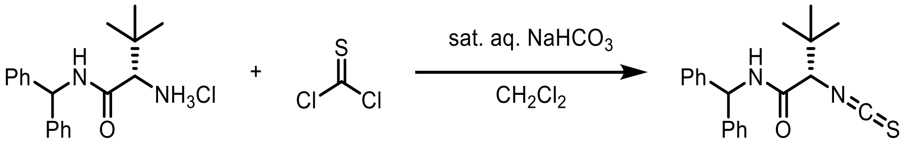

According to a literature procedure,[10] sat. aq. $NaHCO_3$ (60 ml) was added to a vigorously stirred solution of (*S*)-2-amino-N-benzhydryl-3,3-dimethylbutanamide hydrochloride (**S3**) (2.00 g, 6.01 mmol, 1.00 eq) in CH_2Cl_2 (60 ml) under a N_2 atmosphere at 0 °C and the biphasic mixture was stirred for 20 min. Stirring was stopped and thiophosgene (0.46 ml, 6.01 mmol, 1.00 eq) added to the organic layer. Vigorous stirring was recommenced immediately after addition and the mixture allowed to warm to room temperature over 30 min. Stirring was stopped, and the organic phase was extracted with CH_2Cl_2, washed with brine and dried over $MgSO_4$. The volatiles were removed *in vacuo* to give the crude product (**S5**) as a yellow solid (1.81 g) which was taken on without further purification (89%). **^{1}H NMR** (400 MHz, $CDCl_3$) δ(ppm): 7.48 – 7.22 (m, 10H, ArH), 6.65 (d, *J* = 8.0 Hz, 1H, NH), 6.28 (d, *J* = 8.0 Hz, 1H, CH(Ph)$_2$), 4.11 (s, 1H, CHC(=O)), 1.10 (s, 10H, ArH); **^{13}C NMR** (101 MHz, $CDCl_3$) δ(ppm)165.5 (C(=O)), 140.9 (ArC), 140.6 (ArC), 137.3 (N=C=S), 128.9 (ArCH), 128.8 (ArCH), 127.8 (ArCH), 127.8 (ArCH), 127.5 (ArCH), 127.3 (ArCH), 71.0 (CHC(=O)), 57.3 (CH(Ph)$_2$), 37.1 (CH(CH$_3$)$_3$), 26.8 (CH(CH$_3$)$_3$); **IR** (powder) ν_{max}/cm^{-1}: 3294 (NH), 3029, 2957 (C-H), 1658 (NHC=O); $[\alpha]_D^{26.1}$ = +42.9 (*c* 0.97, $CHCl_3$). Data are consistent with that published in the literature.[11]

Synthesis and characterisation of S6

(R)-2-(3-((S)-1-azido-3,3-dimethylbutan-2-yl)thioureido)-N-benzhydryl-3,3-dimethylbutanamide (S6)

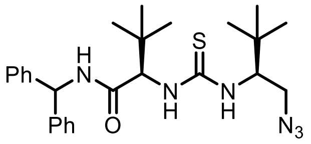

According to a literature procedure,[10] TFA (0.83 ml (1 ml/mmol)) was added to *tert*-butyl (*S*)-(1-azido-3,3-dimethylbutan-2-yl)carbamate (200 mg, 0.83 mmol, 1.00 eq). The reaction was stirred at 0 °C and allowed to warm to ambient temperature over 2 h. The reaction was then concentrated under a stream of N_2 and diluted with Et_2O (4.15 ml (5 ml/mmol)) and H_2O (2.5 ml (3 ml/mmol)). NaOH pellets were added with stirring and the biphasic mixture was brought to pH 14. The phases were partitioned, and the aqueous phase was extracted with Et_2O (2 x 20 ml), washed with brine, dried over $MgSO_4$, filtered, and concentrated *in vacuo* behind a blast shield (care must be taken as the amine is volatile). 0.71 mmol of amino azide was obtained. The amine was then diluted with THF (2.4 ml) and (*R*)-N-benzhydryl-2-isothiocyanato-3,3-dimethylbutanamide (**S4**) (264 mg, 0.78 mmol, 1.10 eq) was added. Once reaction completion was observed by TLC analysis the reaction mixture was concentrated *in vacuo* and purified by silica gel column chromatography (pentane/Et_2O = 6/4) to provide the title compound (**S6**) as a white solid in 70% yield (280 mg). **^{1}H NMR** (600 MHz, Methanol-d_4) δ(ppm): 7.39 – 7.22 (m, 10H, ArH), 6.21 (s, 1H, $CHPh_2$), 5.06 (s, 1H, $C(=O)CH(CH_3)_3$), 4.68 (d, J = 6.5 Hz, 1H, $CHCH_2N_3$), 3.57 (dd, J = 13.0, 4.0 Hz, 1H, one of $CH_AH_BN_3$), 3.39 – 3.29 (m, 1H, one of $CH_AH_BN_3$), 1.03 (s, 9H, one of $C(CH_3)_3$), 1.02 (s, 9H, one of $C(CH_3)_3$); **^{13}C NMR** (151 MHz, Methanol-d_4) δ(ppm)186.0 (C=S), 172.5 (C=O), 143.0 (ArC), 142.7 (ArC), 129.5 (ArCH), 129.3 (ArCH), 129.2 (ArCH), 128.7 (ArCH), 128.4 (ArCH), 128.1 (ArCH), 66.5 ($C(=O)CHC(CH_3)_3$), 62.9 ($CHCH_2N_3$), 58.2 (Ph_2CHNH), 53.0 ($CHCH_2N_3$), 35.9 ($C(=O)CHC(CH_3)_3$ or $NHCHC(CH_3)_3$), 35.7 ($C(=O)CHC(CH_3)_3$ or $NHCHC(CH_3)_3$), 27.3

((C(=O)CHC($\underline{C}H_3$)$_3$ or NHCHC($\underline{C}H_3$)$_3$)), 27.2 ((C(=O)CHC($\underline{C}H_3$)$_3$ or NHCHC($\underline{C}H_3$)$_3$)); $[\boldsymbol{\alpha}]_{\boldsymbol{D}}^{\mathbf{26.1}}$ = +5.9 (c 1.12, $CHCl_3$). Data are consistent with that published in the literature.[12]

Synthesis and characterisation of S7

(S)-2-(3-((S)-1-azido-3,3-dimethylbutan-2-yl)thioureido)-N-benzhydryl-3,3-dimethylbutanamide (S7)

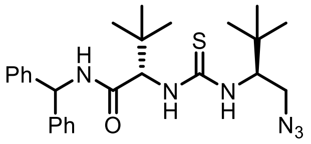

According to a literature procedure,[10] TFA (0.83 ml (1 ml/mmol)) was added to *tert*-butyl (*S*)-(1-azido-3,3-dimethylbutan-2-yl)carbamate (200 mg, 0.830 mmol, 1.00 eq). The reaction was stirred at 0 °C and allowed to warm to ambient temperature over 2h. The reaction was then concentrated under a stream of N_2 and diluted with Et_2O (4.15 ml (5 ml/mmol)) and H_2O (2.5 ml (3 ml/mmol)). NaOH pellets were added with stirring and the biphasic mixture was brought to pH 14. The phases were partitioned, and the aqueous phase was extracted with Et_2O (2 x 20 ml), washed with brine, dried over $MgSO_4$, filtered, and concentrated *in vacuo* behind a blast shield (care must be taken as the amine is volatile). 0.790 mmol of amino azide was obtained. The amine was then diluted with THF (2.8 ml) and (*S*)-N-benzhydryl-2-isothiocyanato-3,3-dimethylbutanamide (**S5**) (292 mg, 0.86 mmol, 1.10 eq) was added. Once reaction completion was observed by TLC analysis the reaction mixture was concentrated *in vacuo* and purified by silica gel column chromatography (pentane/Et_2O = 6/4) to provide the title compound (**S7**) as a white solid in 73% yield (290 mg). **^{1}H NMR** (400 MHz, Methanol-d_4) δ(ppm)(ppm): 7.43 – 7.16 (m, 10H, Ar$\underline{H}$), 6.20 (s, 1H, $Ph_2C\underline{H}NH$), 5.05 (s, 1H, $C(=O)C\underline{H}(CH_3)_3$), 4.69 (dd, J = 8.0, 4.0 Hz, 1H, $C\underline{H}CH_2N_3$), 3.55 (dd, J = 13.0, 4.0 Hz, 1H, $CHCH_A\underline{H_B}N_3$), 1.04 (s, 9H, one of $C(=O)CHC(C\underline{H_3})_3$ or $NHCHC(C\underline{H_3})_3$), 1.01 (s, 1H, one of $C(=O)CHC(C\underline{H_3})_3$ or $NHCHC(C\underline{H_3})_3$) ($CHCH_A\underline{H_B}N_3$ is beneath the solvent peak); **^{13}C NMR** (100 MHz, Methanol-d_4) δ(ppm)(ppm) 186.0 ($\underline{C}$=S), 172.8 ($\underline{C}$=O), 143.0 (Ar$\underline{C}$), 142.7 (Ar$\underline{C}$), 129.6 (Ar$\underline{C}$H), 129.3 (Ar$\underline{C}$H), 129.3 (Ar$\underline{C}$H), 128.6 (Ar$\underline{C}$H), 128.5 (Ar$\underline{C}$H), 128.0 (Ar$\underline{C}$H), 66.6 ($C(=O)\underline{C}HC(CH_3)_3$), 63.3 ($\underline{C}HCH_2N_3$), 58.3 ($Ph_2\underline{C}HNH$), 52.9 ($CH\underline{C}H_2N_3$), 35.6, 35.6 ($C(=O)CH\underline{C}(CH_3)_3$ and

NHCH$\underline{C}$(CH_3)$_3$), 27.3, 27.2 (C(=O)CHC($\underline{C}H_3$)$_3$ and NHCHC($\underline{C}H_3$)$_3$); **MP**: 204-206; $[\boldsymbol{\alpha}]_{\boldsymbol{D}}^{\mathbf{26.1}}$ = –76.8 (c 0.56, $CHCl_3$). Data are consistent with that published in the literature.[11]

Synthesis and characterisation of S8

(R)-2-(3-((R)-1-azido-3,3-dimethylbutan-2-yl)thioureido)-N-benzhydryl-3,3-dimethylbutanamide (S8)

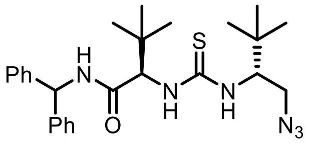

According to a literature procedure,[8,10] (*R*)-N-(1-azido-3,3-dimethylbutan-2-yl)-2,2,2-trifluoroacetamide (**S1**) (800 mg, 3.40 mmol, 1.00 eq) was dissolve in a mixture of MeOH (8.9 ml) and H_2O (4.5 ml). NaOH (1.63 g, 40.8 mmol, 12.0 eq) was added portionwise and the reaction mixture was stirred for 20 h. The aqueous phase was extracted with Et_2O and TFA (3.4 ml) was added. The solvent was removed *in vacuo*. Et_2O (17 ml) and H_2O (10.2 ml) were added. The mixture was basified to pH 14 by addition of NaOH pellets. The aqueous layer was extracted with Et_2O (3 x 10 ml). The combined organic phases were washed with brine, dried over Na_2SO_4, filtered, and concentrated *in vacuo* behind a blast shield (care must be taken as the amine is volatile). 3.16 mmol of amino azide was obtained. The amine was then diluted with THF (10.5 ml) and (*R*)-N-benzhydryl-2-isothiocyanato-3,3-dimethylbutanamide (**S4**) (1.18 g, 3.48 mmol, 1.10 eq) was added. Once reaction completion was observed by TLC analysis the reaction mixture was concentrated *in vacuo* and purified by silica gel column chromatography (pentane/Et_2O = 9/1 to 8/2 to 6/4) to provide an off-white solid which was triturated with Et_2O. The title compound (**S8**) was obtained as a white solid as in 64% yield (1.05 g). **^{1}H NMR** (600 MHz, Methanol-d_4) δ(ppm): 7.36 – 7.32 (m, 2H, Ar<u>H</u>), 7.32 – 7.25 (m, 7H, Ar<u>H</u>), 7.24 – 7.19 (m, 1H, Ar<u>H</u>), 6.18 (s, 1H, $Ph_2C\underline{H}NH$), 5.03 (s, 1H, $C(=O)C\underline{H}(CH_3)_3$), 4.67 (dd, J = 8.0, 4.0 Hz, 1H, $C\underline{H}CH_2N_3$), 3.53 (dd, J = 13.0, 4.0 Hz, 1H, $CHC\underline{H_A}H_BN_3$), 1.02 (s, 9H, one of $C(=O)CHC(C\underline{H_3})_3$ or $NHCHC(C\underline{H_3})_3$), 0.99 (s, 9H, one of $C(=O)CHC(C\underline{H_3})_3$ or $NHCHC(C\underline{H_3})_3$). ($CHCH_A\underline{H_B}N_3$ is beneath the solvent peak); **^{13}C NMR** (151 MHz, Methanol-d_4) δ(ppm): 186.0 ($\underline{C}$=S), 172.8 ($\underline{C}$=O), 143.0 (Ar$\underline{C}$), 142.7 (Ar$\underline{C}$), 129.6 (Ar$\underline{C}$H), 129.3

(Ar$\underline{C}$H), 129.3 (Ar$\underline{C}$H), 128.6 (Ar$\underline{C}$H), 128.5 (Ar$\underline{C}$H), 128.0 (Ar$\underline{C}$H), 66.6 ($C(=O)\underline{C}HC(CH_3)_3$), 63.4 ($\underline{C}HCH_2N_3$), 58.3 ($Ph_2\underline{C}HNH$), 52.9 ($CH\underline{C}H_2N_3$), 35.6 (one of $C(=O)CH\underline{C}(CH_3)_3$ or $NHCH\underline{C}(CH_3)_3$), 35.6 (one of $C(=O)CH\underline{C}(CH_3)_3$ or $NHCH\underline{C}(CH_3)_3$), 27.3 (one of $C(=O)CHC(\underline{C}H_3)_3$ or $NHCHC(\underline{C}H_3)_3$), 27.2 (one of $C(=O)CHC(\underline{C}H_3)_3$ or $NHCHC(\underline{C}H_3)_3$); $[\boldsymbol{\alpha}]_{\boldsymbol{D}}^{\mathbf{26.1}}$ = +91.4 (*c* 0.99, $CHCl_3$). Data are consistent with that published in the literature.[11]

5. Synthesis and Characterisation of Substrates used in the Enantioselective Deconjugation of Cyclopropenes

5.1 Synthesis of cyclopropene-substrate precursors

Synthesis and characterisation of S9

dimethyl 2-cyclohexylcycloprop-2-ene-1,1-dicarboxylate (S9)

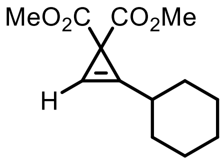

Compound **S9** was synthesised according to general procedure **IV** by the addition of dimethyl diazomalonate (605 mg, 3.83 mmol, 1.00 eq) to cyclohexylacetylene (1.50 ml, 11.48 mmol, 3.00 eq). The crude product was purified by silica gel column chromatography (pentane/EtOAc = 9/1) to provide the title compound (**S9**) as a yellow oil in 46% yield (418 mg). **^{1}H NMR** (400 MHz, $CDCl_3$) δ(ppm): 6.31 (d, *J* = 1.5 Hz, 1H, C=C$\underline{H}$), 3.70 (s, 6H, $C(CO_2C\underline{H_3})_2$), 2.69 – 2.58 (m, 1H, C$\underline{H}$C=CH), 1.98 – 1.84 (m, 2H, $C\underline{H_A}H_BCHC\underline{H_C}H_D$), 1.79 – 1.52 (m, 3H, $C\underline{H_A}\underline{H_B}CH_2CHCH_2C\underline{H_C}H_D$), 1.52 – 1.15 (m, 5H, $C\underline{H_A}H_BCHC\underline{H_C}H_D$, $CH_AH_BCH_2CHCH_2CH_C\underline{H_D}$ and $CHCH_2CH_2C\underline{H_2}$); **^{13}C NMR** (101 MHz, $CDCl_3$) δ(ppm): 172.1 ($\underline{C}$=O), 117.9 ($\underline{C}$=CH), 92.5 (C=$\underline{C}$H), 52.3 ($C(CO_2\underline{C}H_3)_2$), 34.0 ($\underline{C}$HC=CH), 32.4 ($\underline{C}(CO_2CH_3)_2$), 30.2 ($CH\underline{C}H_2$), 25.9 ($CHCH_2CH_2\underline{C}H_2$), 25.3 ($CHCH_2\underline{C}H_2CH_2$); **IR** (film) ν_{max}/cm^{-1}: 3137, 2930, 2855 (C-H), 1723 (C=O); **HRMS** (ES+) exact mass calculated for $[M+Na]^+$ ($C_{13}H_{18}NaO_4^+$) requires *m/z* 261.1097, found *m/z* 261.1096.

Synthesis and characterisation of S10

dimethyl 2-butylcycloprop-2-ene-1,1-dicarboxylate (S10)

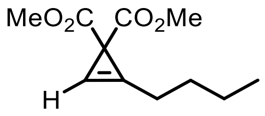

Compound **S10** was synthesised according to general procedure **IV** by the addition of dimethyl diazomalonate (1.21 g, 7.66 mmol, 1.00 eq) to 1-hexyne (2.64 ml, 23.0 mmol, 3.00 eq). After the addition extra 1-hexyne (1.30 ml, 11.5 mmol, 1.50 eq) and $Rh_2(OAc)_4$ (34.0 mg, 0.0770 mmol, 0.0100 eq). The crude product was purified by silica gel column chromatography (pentane/EtOAc = 95/5 to 9/1) to provide the title compound (**S10**) as a yellow oil in 67% yield (1.07 g). **^{1}H NMR** (400 MHz, $CDCl_3$) δ(ppm): 6.34 (t, *J* = 1.5 Hz, 1H, C=C$\underline{H}$), 3.69 (s, 6H, $C(CO_2C\underline{H_3})_2$), 2.53 (td, *J* = 7.5, 1.5 Hz, 2H, $C\underline{H_2}C{=}CH$), 1.62 – 1.51 (m, 2H, $CH_3CH_2C\underline{H_2}$), 1.43 – 1.31 (m, 2H, $CH_3C\underline{H_2}CH_2$), 0.90 (t, *J* = 7.5 Hz, 3H, $C\underline{H_3}CH_2CH_2$); **^{13}C NMR** (101 MHz, $CDCl_3$) δ(ppm): 172.0 ($C(\underline{C}O_2Me)_2$), 114.7 ($\underline{C}{=}CH$), 93.6 ($C{=}\underline{C}H$), 52.3 ($C(CO_2\underline{C}H_3)_2$), 32.5 ($\underline{C}(CO_2Me)_2$), 28.5 ($CH_2C\underline{H_2}CH_2CH_3$), 23.8 ($C\underline{H_2}CH_2CH_2CH_3$), 22.3 ($CH_2CH_2C\underline{H_2}CH_3$), 13.7 ($CH_2CH_2CH_2C\underline{H_3}$); **IR** (film) ν_{max}/cm^{-1}: 3137, 2956, 2874 (C-H), 1724 (C=O). Data are consistent with that published in the literature.[13]

Synthesis and characterisation of S11

dimethyl 2-cyclopentylcycloprop-2-ene-1,1-dicarboxylate (S11)

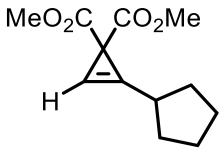

Compound **S11** was synthesised according to general procedure **IV** by the addition of dimethyl diazomalonate (1.21 g, 7.66 mmol, 1.00 eq) to cyclopentylacetylene (2.67 ml, 23.0 mmol, 3.00 eq). The crude product was purified by silica gel column chromatography (pentane/EtOAc = 95/5) to provide the title compound (**S11**) as a yellow oil in 53% yield (907 mg). **^{1}H NMR** (400 MHz, $CDCl_3$) δ(ppm): 6.29 (d, J = 1.5 Hz, 1H, C=C$\underline{H}$), 3.69 (s, 6H, $C(COC\underline{H_3})_2$), 3.10 – 2.96 (m, 1H, C$\underline{H}$C=CH), 1.98 – 1.81 (m, 2H, 2 of $CHC\underline{H_A}H_BCH_2CH_2C\underline{H_B}H_C$), 1.77 – 1.51 (m, 6H, 2 of $CHCH_A\underline{H_B}CH_2CH_2CH_B\underline{H_C}$ and $CHCH_AH_BC\underline{H_2}C\underline{H_2}CH_BH_C$); **^{13}C NMR** (101 MHz, $CDCl_3$) δ(ppm): 172.1 ($C(\underline{C}O_2Me)_2$), 118.0 ($\underline{C}$=CH), 92.4 (C=$\underline{C}$H), 52.2 ($C(CO_2\underline{C}H_3)_2$), 35.1 ($\underline{C}$HC=CH), 33.1 ($\underline{C}(CO_2CH_3)_2$), 31.0 ($CH\underline{C}H_2CH_2CH_2\underline{C}H_2$), 25.2 ($CHCH_2\underline{C}H_2\underline{C}H_2CH_2$); **IR** (film) ν_{max}/cm^{-1}: 3137, 2954, 2872 (C-H), 1722 (C=O). Data are consistent with that published in the literature.[14]

Synthesis and characterisation of S12

dimethyl 2-(1-(tert-butoxycarbonyl)piperidin-4-yl)cycloprop-2-ene-1,1-dicarboxylate (S12)

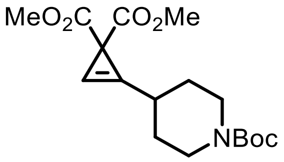

Compound **S12** was synthesised according to general procedure **IV** by the addition of dimethyl diazomalonate (632 mg, 4 mmol, 1.00 eq) to cyclohexyne (1.7 g, 8 mmol, 2.00 eq). The crude product was purified by silica gel column chromatography (pentane/EtOAc = 3/1) to provide the title compound (**S12**) as a white solid in 14% yield (188 mg). **^{1}H NMR** (400 MHz, $CDCl_3$) δ(ppm): 6.42 (d, *J* = 1.4 Hz, 1H, $C{=}C\underline{H}$), 3.97 – 3.81 (m, 2H, $C\underline{H_A}H_BN(Boc)C\underline{H_C}H_D$), 3.70 (s, 6H, $C(CO_2C\underline{H_3})_2$), 2.98 (ddd, *J* = 13.6, 10.4, 3.1 Hz, 2H, $CH_A\underline{H_B}N(Boc)CH_C\underline{H_D}$), 2.82 (ttd, *J* = 9.8, 3.9, 1.4 Hz, 1H, $HC{=}CC\underline{H}$), 1.94 – 1.78 (m, 2H, $C\underline{H_A}H_BCH_2N(Boc)\ CH_2C\underline{H_C}H_D$), 1.64 – 1.50 (m, 2H, $CH_A\underline{H_B}CH_2N(Boc)\ CH_2CH_C\underline{H_D}$), 1.44 (s, 9H, $C(C\underline{H_3})_3$); **^{13}C NMR** (101 MHz, $CDCl_3$) δ(ppm): 171.8 ($\underline{C}{=}O$), 154.8 ($\underline{C}{=}O$ of Boc), 116.5 ($\underline{C}{=}CH$), 94.2 ($C{=}\underline{C}H$), 79.8 ($\underline{C}(CH_3)_3$), 52.4 ($C(CO_2\underline{C}H_3)_2$), 42.8 ($CH_2\underline{C}H_2N(Boc)\ \underline{C}H_2CH_2$), 32.4 ($HC{=}C\underline{C}H$), 32.2 ($\underline{C}(CO_2CH_3)_2$), 29.2 ($\underline{C}H_2CH_2N(Boc)\ CH_2\underline{C}H_2$), 28.5 ($C(\underline{C}H_3)_3$); **HRMS** (ES+) exact mass calculated for $[M+Na]^+$ ($C_{17}H_{25}NO_6Na^+$) requires *m/z* 362.1574, found *m/z* 362.1562.

Synthesis and characterisation of S13

<u>dimethyl 2-(tetrahydro-2H-pyran-4-yl)cycloprop-2-ene-1,1-dicarboxylate (S13)</u>

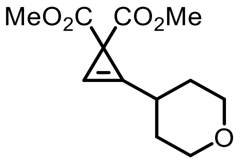

Compound **S13** was synthesised according to general procedure **IV** by the addition of dimethyl 2-diazomalonate (0.38 g, 2.5 mmol, 1.00 eq) to 4-ethynyltetrahydro-2H-pyran (550 mg, 5.0 mmol, 2.00 eq). The crude product was purified by silica gel column chromatography (pentane/EtOAc = 7/1) to provide the title compound (**S13**) as a colourless oil in 60% yield (360 mg). **^{1}H NMR** (400 MHz, $CDCl_3$) δ(ppm): 6.41 (d, *J* = 1.4 Hz, 1H, $C{=}C\underline{H}$), 3.91 (t, *J* = 4.0 Hz, 1H, one of $C\underline{H_A}H_BOCH_CH_D$), 3.88 (t, *J* = 4.0 Hz, 1H, one of $CH_AH_BOC\underline{H_C}H_D$), 3.69 (s, 6H, $C(COC\underline{H_3})_2$), 3.51 – 3.43 (m, 2H, 2 of $CH_A\underline{H_B}OCH_C\underline{H_D}$), 2.89 (ttd, *J* = 10.0, 4.1, 1.4 Hz, 1H, $C\underline{H}C{=}CH$), 1.92 – 1.79 (m, 2H, 2 of $C\underline{H_A}H_BCH_2OCH_2C\underline{H_C}H_D$), 1.69 (dtd, *J* = 14.0, 10.2, 4.1 Hz, 2H, 2 of $CH_A\underline{H_B}CH_2OCH_2CH_C\underline{H_D}$); **^{13}C NMR** (101 MHz, $CDCl_3$) δ(ppm): 171.8 ($C(\underline{C}O_2Me)_2$), 116.6 ($\underline{C}{=}CH$), 93.8 ($C{=}\underline{C}H$), 66.8 ($\underline{C}H_2O\underline{C}H_2$), 52.4 ($C(CO_2\underline{C}H_3)_2$), 32.4 ($\underline{C}(CO_2CH_3)_2$), 31.2 ($\underline{C}HC{=}CH$), 29.9 ($\underline{C}H_2CH_2OCH_2\underline{C}H_2$); **HRMS** (ES+) exact mass calculated for $[M+H]^+$ ($C_{12}H_{17}O_5^+$) requires *m/z* 241.1071, found *m/z* 241.1064.

Synthesis and characterisation of S14

dimethyl 2-(3-chloropropyl)cycloprop-2-ene-1,1-dicarboxylate (S14)

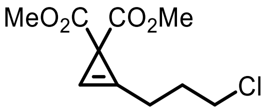

Compound **S14** was synthesised according to general procedure **IV** by the addition of dimethyl diazomalonate (790 mg, 5 mmol, 1.00 eq) to 5-chloropent-1-yne (1060 µL, 10 mmol, 2.00 eq). The crude product was purified by silica gel column chromatography (pentane/Et_2O = 5/1) to provide the title compound (**S14**) as a colourless oil in 34% yield (639 mg). **^{1}H NMR** (400 MHz, $CDCl_3$) δ(ppm): 6.44 (t, *J* = 1.4 Hz, 1H, C=C<u>H</u>), 3.71 (s, 6H, $C(CO_2C\underline{H_3})_2$), 3.59 (t, *J* = 6.4 Hz, 2H, $ClC\underline{H_2}$), 2.74 (td, *J* = 7.1, 1.4 Hz, 2H, $ClCH_2CH_2C\underline{H_2}$), 2.14 – 2.02 (m, 2H, $ClCH_2C\underline{H_2}CH_2$); **^{13}C NMR** (101 MHz, $CDCl_3$) δ(ppm): 171.7 ($C(\underline{C}O_2Me)_2$), 113.4 ($\underline{C}$=CH), 95.0 (C=$\underline{C}$H), 52.4 ($C(CO_2\underline{C}H_3)_2$), 43.7 ($CH_2CH_2\underline{C}H_2Cl$), 32.5 ($\underline{C}(CO_2Me)_2$), 29.4 ($\underline{C}H_2CH_2CH_2Cl$), 21.5 ($CH_2\underline{C}H_2CH_2Cl$); **HRMS** (ES+) exact mass calculated for $[M+ K]^+$ ($C_{10}H_{13}ClO_4K^+$) requires *m/z* 271.0134, found *m/z* 271.0139;

Synthesis and characterisation of S15

dimethyl 2-(3-((tert-butyldimethylsilyl)oxy)propyl)cycloprop-2-ene-1,1-dicarboxylate (S15)

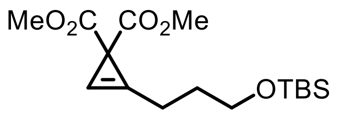

Compound **S15** was synthesised according to general procedure **IV** by the addition of dimethyl diazomalonate (1.4 g, 8.9 mmol, 1.00 eq) to tert-butyldimethyl(pent-4-yn-1-yloxy)silane (3.54 g, 17.9 mmol, 2.00 eq). The crude product was purified by silica gel column chromatography (pentane/Et_2O = 5/1) to provide the title compound (**S15**) as a colourless oil in 56% yield (1.64 g). **^{1}H NMR** (400 MHz, $CDCl_3$) δ(ppm): 6.36 (q, *J* = 1.2 Hz, 1H, C=C<u>H</u>), 3.70 (s, 6H, $C(CO_2C\underline{H_3})_2$), 3.64 (t, *J* = 6.0 Hz, 2H, $C\underline{H_2}OTBS$), 2.62 (td, *J* = 7.4, 1.4 Hz, 2H, $C\underline{H_2}C{=}CH$), 1.87 – 1.75 (m, 2H, $C\underline{H_2}CH_2OTBS$), 0.88 (s, 9H, $Si(CH_3)_2C(C\underline{H_3})_3$), 0.03 (s, 6H, $Si(C\underline{H_3})_2C(CH_3)_3$); **^{13}C NMR** (101 MHz, $CDCl_3$) δ(ppm): 171.9 ($C(\underline{C}O_2Me)_2$), 114.5 ($\underline{C}{=}CH$), 93.9 ($C{=}\underline{C}H$), 61.8 ($TBSO\underline{C}H_2$), 52.3 ($C(CO_2\underline{C}H_3)_2$), 32.5 ($\underline{C}(CO_2Me)_2$), 29.7 ($TBSOCH_2CH_2\underline{C}H_2$), 26.0 ($Si(CH_3)_2\underline{C}(CH_3)_3$), 20.7 ($TBSOCH_2\underline{C}H_2$), 18.4 ($Si(CH_3)_2C(\underline{C}H_3)_3$), -5.2 ($Si(\underline{C}H_3)_2C(CH_3)_3$); **HRMS** (ES+) exact mass calculated for $[M+ NH_4]^+$ ($C_{16}H_{32}O_5NSi^+$) requires *m/z* 346.2044, found *m/z* 346.2033.

Synthesis and characterisation of S16

dimethyl 2-(3-phenylpropyl)cycloprop-2-ene-1,1-dicarboxylate (S16)

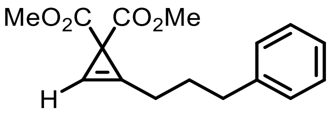

Compound **S16** was synthesised according to general procedure **IV** by the addition of dimethyl 2-diazomalonate (0.62 g, 4.1 mmol, 1.00 eq) to pent-4-yn-1-ylbenzene (1.12 mL, 8.2 mmol, 2.00 eq). The crude product was purified by silica gel column chromatography (pentane/EtOAc = 8/1) to provide the title compound (**S16**) as a colourless oil in 59% yield (1.32 g). **^{1}H NMR** (400 MHz, $CDCl_3$) δ(ppm): 7.31 – 7.26 (m, 2H, ArH), 7.22 – 7.14 (m, 3H, ArH), 6.40 (t, *J* = 1.4 Hz, 1H, C=CH), 3.71 (s, 6H, $C(CO_2CH_3)_2$), 2.73 – 2.65 (m, 2H, $PhCH_2CH_2CH_2$), 2.57 (td, *J* = 7.3, 1.5 Hz, 2H, $PhCH_2CH_2CH_2$), 1.94 (p, *J* = 7.4 Hz, 2H, $PhCH_2CH_2CH_2$); **^{13}C NMR** (101 MHz, $CDCl_3$) δ(ppm): 171.9 ($C(CO_2Me)_2$), 141.3 (ArC), 128.6 (ArCH), 128.5 (ArCH), 126.1 (ArCH), 114.3 (C=CH), 94.1 (C=CH), 52.3 ($C(CO_2CH_3)_2$), 35.1 ($PhCH_2CH_2CH_2$), 32.5 ($C(CO_2Me)_2$), 28.1 ($PhCH_2CH_2CH_2$), 23.5 ($PhCH_2CH_2CH_2$); HRMS (ES+) exact mass calculated for [M+ H]+ (C16H19O4+) requires m/z 275.1278, found m/z 275.1265.

Synthesis and characterisation of S17

dimethyl 2-(2-bromoethyl)cycloprop-2-ene-1,1-dicarboxylate (S17)

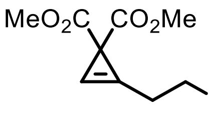

Compound **S17** was synthesised according to general procedure **IV** by the addition of dimethyl 2-diazomalonate (0.79 g, 5 mmol, 1.00 eq) to 4-bromobut-1-yne (0.92 mL, 10.0 mmol, 2.00 eq). The crude product was purified by silica gel column chromatography (pentane/EtOAc = 5/1) to provide the title compound (**S17**) as a colorless oil in 28% yield (371 mg). **^{1}H NMR** (400 MHz, $CDCl_3$) δ(ppm): 6.57 (t, *J* = 1.4 Hz, 1H, C=C<u>H</u>), 3.71 (s, 6H, $C(CO_2C\underline{H_3})_2$), 3.55 (t, *J* = 6.9 Hz, 2H, $BrCH_2$), 3.15 (td, *J* = 6.9, 1.4 Hz, 2H, $C\underline{H_2}C$=CH). **^{13}C NMR** (101 MHz, $CDCl_3$) δ(ppm): 171.4 ($C(\underline{C}O_2Me)_2$), 112.2 (<u>C</u>=CH), 96.5 (C=<u>C</u>H), 52.5 ($C(CO_2\underline{C}H_3)_2$), 32.5 ($\underline{C}(CO_2Me)_2$), 27.9 ($BrCH_2\underline{C}H_2$), 27.5 ($Br\underline{C}H_2CH_2$). **HRMS** (ES+) exact mass calculated for $[M+ K]^+$ ($C_9H_{11}BrO_4K^+$) requires *m/z* 300.9472, found *m/z* 300.9462;

Synthesis and characterisation of S18

dimethyl 2-ethylcycloprop-2-ene-1,1-dicarboxylate (S17)

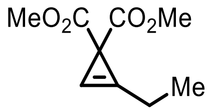

According to a modified literature procedure,[15] compound **S17** (300 mg, 1.14 mmol, 1.0 equiv.) and Et_3B (1 M in THF, 2.3 mL, 2.3 mmol, 2.0 equiv.) were added to a stirred solution of Bn_3SnH (0.61 mL, 2.3 mmol, 2.0 equiv) in toluene (3 mL) at room temperature, the reaction mixture was stirred at room temperature for 3 h, and then adding the second portion of Et_3B (1 M in THF, 2.3 mL, 2.3 mmol, 2.0 equiv.) and Bn_3SnH (0.61 mL, 2.3 mmol, 2.0 equiv). The reaction mixture was stirred at room temperature for another 3 hours. After completion, the reaction was quenched by CCl_4 (0.44 mL, 4.6 mmol, 4 equiv.) and stirred for 5 min, the resulting mixture was concentrated *in vacuo*. The crude was purified by silica gel column chromatography (pentane/EtOAc = 5/1) to provide the title compound (**S18**) as a colourless oil in 73% yield (153 mg). **^{1}H NMR** (400 MHz, $CDCl_3$) δ(ppm)6.35 (t, *J* = 1.5 Hz, 1H, C=C$\underline{H}$), 3.71 (s, 6H, $C(CO_2C\underline{H_3})_2$), 2.57 (qd, *J* = 7.5, 1.5 Hz, 2H, $C\underline{H_2}C$=CH), 1.19 (t, *J* = 7.5 Hz, 3H, $C\underline{H_3}CH_2$); **^{13}C NMR** (101 MHz, $CDCl_3$) δ(ppm)172.0 ($C(\underline{C}O_2Me)_2$), 115.7 ($\underline{C}$=CH), 93.2 (C=$\underline{C}$H), 52.3 ($C(CO_2\underline{C}H_3)_2$), 32.6 ($\underline{C}(CO_2Me)_2$), 17.8 ($CH_3C\underline{H_2}$), 11.1 ($C\underline{H_3}CH_2$); **HRMS** (ES+) exact mass calculated for $[M+ K]^+$ ($C_9H_{12}O_4K^+$) requires *m/z* 223.0367, found *m/z* 223.0376.

Synthesis and characterisation of S19

dimethyl 2-(2-((tert-butyldimethylsilyl)oxy)ethyl)cycloprop-2-ene-1,1-dicarboxylate (S19)

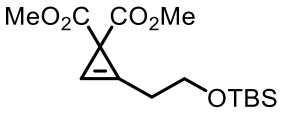

Compound **S19** was synthesised according to general procedure **IV** by the addition of dimethyl diazomalonate (790 mg, 5 mmol, 1.00 eq) to (but-3-yn-1-yloxy)(tert-butyl)dimethylsilane (1.5 mL, 7.5 mmol, 1.50 eq). The crude product was purified by silica gel column chromatography (pentane/Et_2O = 5/1) to provide the title compound (**S19**) as a colourless oil in 48% yield (750 mg). **^{1}H NMR** (400 MHz, $CDCl_3$) δ(ppm): 6.41 (q, *J* = 1.3 Hz, 1H, C=C<u>H</u>), 3.95 – 3.76 (m, 2H, C<u>H$_2$</u>OTBS), 3.70 (s, 6H, C(CO_2C<u>H$_3$</u>)$_2$), 2.76 (t, *J* = 6.9 Hz, 2H, C<u>H$_2$</u>C=CH), 0.87 (s, 9H, Si(CH_3)$_2$C(C<u>H$_3$</u>)$_3$), 0.05 (s, 6H, Si(C<u>H$_3$</u>)$_2$C(CH_3)$_3$); **^{13}C NMR** (101 MHz, $CDCl_3$) δ(ppm): 171.8 (C(<u>C</u>O_2Me)$_2$), 112.2 (<u>C</u>=CH), 94.9 (C=<u>C</u>H), 60.0 (TBSO<u>C</u>H_2), 52.3 (C(CO_2<u>C</u>H_3)$_2$), 32.2 (<u>C</u>(CO_2Me)$_2$), 28.1(TBSOCH_2<u>C</u>H_2), 26.0 (Si(CH_3)$_2$<u>C</u>(CH_3)$_3$), 18.3 (Si(CH_3)$_2$C(<u>C</u>H_3)$_3$), -5.3 (Si(<u>C</u>H_3)$_2$C(CH_3)$_3$); **HRMS** (ES+) exact mass calculated for $[M+ Na]^+$ ($C_{15}H_{26}O_5SiNa^+$) requires *m/z* 337.1442, found *m/z* 337.1433.

Synthesis and characterisation of S20

dimethyl 2-phenethylcycloprop-2-ene-1,1-dicarboxylate (S20)

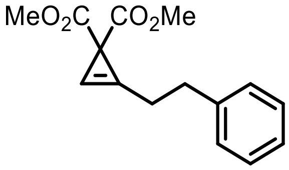

Compound **S20** was synthesised according to general procedure **IV** by the addition of dimethyl diazomalonate (316 mg, 8 mmol, 1.00 equiv.) to but-3-yn-1-ylbenzene (562 μL, 4 mmol, 2.00 equiv.). The crude product was purified by silica gel column chromatography (pentane/Et_2O = 3/1) to provide the title compound (**S20**) as a colourless oil in 56% yield (615 mg). **^{1}H NMR** (400 MHz, $CDCl_3$) δ(ppm): 7.33 – 7.26 (m, 2H, ArH), 7.24 – 7.17 (m, 3H, ArH), 6.38 (t, *J* = 1.4 Hz, 1H, C=CH), 3.69 (s, 6H, $C(CO_2CH_3)_2$), 2.96 – 2.91 (m, 2H, $PhCH_2CH_2$), 2.91 – 2.84 (m, 2H, $PhCH_2CH_2$); **^{13}C NMR** (101 MHz, $CDCl_3$) δ(ppm): 171.8 ($C(CO_2Me)_2$), 140.2 (ArC), 128.6 (ArCH), 128.3 (ArCH), 126.5 (ArCH), 114.0 (C=CH), 94.5 (C=CH), 52.3 ($C(CO_2CH_3)_2$), 32.6 ($C(CO_2Me)_2$), 25.7 ($PhCH_2CH_2$); **HRMS** (ES+) exact mass calculated for $[M+ K]^+$ ($C_{23}H_{22}O_6K^+$) requires *m/z* 433.1048, found *m/z* 433.1051.

Synthesis and characterisation of S21

diethyl 2-butylcycloprop-2-ene-1,1-dicarboxylate (S21)

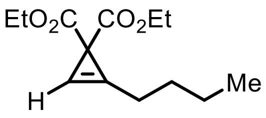

Compound **S21** was synthesised according to general procedure **IV** by the addition of diethyl diazomalonate (744 mg, 4 mmol, 1.00 eq) to hex-1-yne (688 μL, 8 mmol, 2.00 eq). The crude product was purified by silica gel column chromatography (pentane/EtOAc = 5/1) to provide the title compound (**S21**) as a yellow oil in 44% yield (402 mg). **^{1}H NMR** (400 MHz, $CDCl_3$) δ(ppm): 6.35 (t, *J* = 1.4 Hz, 1H, C=C$\underline{H}$), 4.16 (qd, *J* = 7.2, 1.2 Hz, 4H, $C(CO_2C\underline{H_2}CH_3)_2$), 2.54 (td, *J* = 7.4, 1.4 Hz, 2H, $C\underline{H_2}CH_2CH_2CH_3$), 1.63 – 1.53 (m, 2H, $CH_2C\underline{H_2}CH_2CH_3$), 1.44 – 1.33 (m, 2H, $CH_2CH_2C\underline{H_2}CH_3$), 1.24 (t, *J* = 7.1 Hz, 6H, $C(CO_2CH_2C\underline{H_3})_2$), 0.90 (t, *J* = 7.3 Hz, 3H, $CH_2CH_2CH_2C\underline{H_3}$); **^{13}C NMR** (101 MHz, $CDCl_3$) δ(ppm): 171.7 ($C(\underline{C}O_2Et)_2$), 114.9 ($\underline{C}$=CH), 93.7 (C=$\underline{C}$H), 61.0 ($\underline{C}(CO_2\underline{C}H_2CH_3)_2$), 32.9 ($\underline{C}H(CO_2Et)_2$), 28.6 ($\underline{C}H_2CH_2CH_2CH_3$), 23.8 ($CH_2\underline{C}H_2CH_2CH_3$), 22.3 ($CH_2CH_2\underline{C}H_2CH_3$), 14.3 ($C(CO_2CH_2\underline{C}H_3)_2$), 13.8 ($CH_2CH_2CH_2\underline{C}H_3$); **HRMS** (ES+) exact mass calculated for $[M+Na]^+$ ($C_{13}H_{20}O_4Na^+$) requires *m/z* 263.1254, found *m/z* 263.1244.

Synthesis and characterisation of S22

diisopropyl 2-butylcycloprop-2-ene-1,1-dicarboxylate (S22)

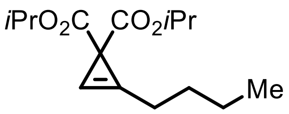

Compound **S22** was synthesised according to general procedure **IV** by the addition of diisopropyl 2-diazomalonate (1.05 g, 5.0 mmol, 1.00 eq) to hex-1-yne (1.14 mL, 10 mmol, 2.00 eq). The crude product was purified by silica gel column chromatography (pentane/Et_2O = 10/1) to provide the title compound (**S22**) as a colourless oil in 17% yield (235 mg). **^{1}H NMR** (400 MHz, $CDCl_3$) δ(ppm): 6.34 (t, *J* = 1.4 Hz, 1H, C=C$\underline{H}$), 5.02 (p, *J* = 6.3 Hz, 2H), 2.54 (td, *J* = 7.3, 1.4 Hz, 2H), 1.61 – 1.51 (m, 2H), 1.48 – 1.33 (m, 2H), 1.22 (d, *J* = 5.9 Hz, 12H), 0.90 (t, *J* = 7.3 Hz, 3H); **^{13}C NMR** (101 MHz, $CDCl_3$) δ(ppm): 171.4 (C($\underline{C}O_2{}^i Pr)_2$), 115.1 ($\underline{C}$=CH), 93.9 (C=$\underline{C}$H), 68.3 ($\underline{C}H(CH_3)_2)_{\underline{2}}$), 33.3, 28.6 ($CH_2C\underline{H_2}CH_2CH_3$), 23.8 ($C\underline{H_2}CH_2CH_2CH_3$), 22.3 ($CH_2CH_2C\underline{H_2}CH_3$), 21.9 (one of ($CH(\underline{C}H_3)_A(CH_3)_B)_{\underline{2}}$), 21.8(one of ($CH(CH_3)_A(\underline{C}H_3)_B)_{\underline{2}}$), 13.8 ($CH_2CH_2CH_2C\underline{H_3}$); **HRMS** (ES+) exact mass calculated for $[M+ Na]^+$ ($C_{15}H_{24}O_4Na^+$) requires *m/z* 291.1567, found *m/z* 291.1558.

Synthesis and characterisation of S23

di-tert-butyl 2-butylcycloprop-2-ene-1,1-dicarboxylate (S23)

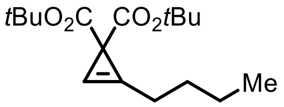

Compound **S23** was synthesised according to general procedure **IV** by the addition of di-tert-butyl 2-diazomalonate (0.97 g, 4.0 mmol, 1.00 eq) to hex-1-yne (0.69 mL, 8 mmol, 2.00 eq). The crude product was purified by silica gel column chromatography (pentane/Et_2O = 10/1) to provide the title compound (**S23**) as a colourless oil in 59% yield (700 mg). **^{1}H NMR** (400 MHz, $CDCl_3$) δ(ppm): 6.33 (s, 1H, C=C$\underline{H}$), 2.55 (t, *J* = 7.3 Hz, 2H, $C\underline{H_2}CH_2CH_2CH_3$), 1.62 – 1.51 (m, 2H, $CH_2C\underline{H_2}CH_2CH_3$), 1.45 (s, 18H, $C(CO_2C(C\underline{H_3})_3)_2$), 1.42 – 1.33 (m, 2H, $CH_2CH_2C\underline{H_2}CH_3$), 0.90 (t, *J* = 7.3 Hz, 3H, $CH_2CH_2CH_2C\underline{H_3}$); **^{13}C NMR** (101 MHz, $CDCl_3$) δ(ppm): 171.6 ($C(\underline{C}O_2C(CH_3)_3)_2$), 115.6 ($\underline{C}$=CH), 94.3 (C=$\underline{C}$H), 80.7 ($C(CO_2\underline{C}(CH_3)_3)_2$), 35.0 ($\underline{C}(CO_2{}^tBu)_2$), 28.7 ($C\underline{H_2}CH_2CH_2CH_3$), 28.2 ($C(CO_2C(\underline{C}H_3)_3)_2$), 23.8 ($CH_2C\underline{H_2}CH_2CH_3$), 22.3 ($CH_2CH_2C\underline{H_2}CH_3$), 13.8 ($CH_2CH_2CH_2C\underline{H_3}$); **HRMS** (ES+) exact mass calculated for $[M+Na]^+$ ($C_{17}H_{28}O_4Na^+$) requires *m/z* 319.1880, found *m/z* 319.1869.

Synthesis and characterisation of S24

dibenzyl 2-butylcycloprop-2-ene-1,1-dicarboxylate (S24)

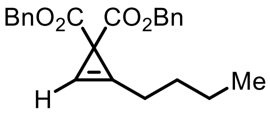

Compound **S24** was synthesised according to general procedure **IV** by the addition of dibenzyl 2-diazomalonate (0.93 g, 3.0 mmol, 1.00 eq) to hex-1-yne (0.688 mL, 6 mmol, 2.00 eq). The crude product was purified by silica gel column chromatography (pentane/Et_2O = 10/1) to provide the title compound (**S24**) as a colourless oil in 26% yield (285 mg). **^{1}H NMR** (400 MHz, $CDCl_3$) δ(ppm): 7.38 – 7.28 (m, 10H, ArH), 6.38 (s, 1H, C=CH), 5.23 – 5.07 (m, 4H, 2×PhCH_2), 2.53 (td, *J* = 7.4, 1.5 Hz, 2H, CH_2C=CH), 1.57 – 1.47 (m, 2H, $CH_3CH_2CH_2$), 1.40 – 1.22 (m, 2H, $CH_3CH_2CH_2$), 0.85 (t, *J* = 7.3 Hz, 3H, $CH_3CH_2CH_2$); **^{13}C NMR** (101 MHz, $CDCl_3$) δ(ppm): 171.4 (C($CO_2Me)_2$), 136.1 (2×ArC), 128.6 (2×ArC), 128.2 (2×ArC), 128.1 (2×ArC), 114.7 (C=CH), 93.5 (C=CH), 66.8 (2×PhCH_2), 33.0 (C($CO_2Me)_2$), 28.6 ($CH_2CH_2CH_2CH_3$), 23.8 ($CH_2CH_2CH_2CH_3$), 22.3 ($CH_2CH_2CH_2CH_3$), 13.7($CH_2CH_2CH_2CH_3$); **HRMS** (ES+) exact mass calculated for $[M+Na]^+$ ($C_{23}H_{24}O_4Na^+$) requires *m/z* 387.1567, found *m/z* 387.1568.

5.2 Synthesis of ester substrates

Synthesis and characterisation of 3a

2-benzyl 1,1-dimethyl 3-cyclohexylcycloprop-2-ene-1,1,2-tricarboxylate (3a)

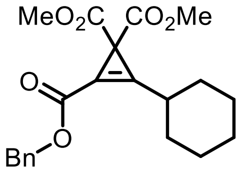

Compound **3a** was synthesised according to general procedure **V** by the addition of **S9** (400 mg, 1.68 mmol, 1.00 eq) to benzyl chloroformate (0.35 ml, 2.52 mmol, 1.50 eq). The crude product was purified by silica gel column chromatography (1st column: CH_2Cl_2, 2nd column: pentane/CH_2Cl_2 = 1/1, 3rd column: pentane/Et_2O = 95:5) to provide the title compound (**3a**) as a white solid in 44% yield (277 mg). **^{1}H NMR** (500 MHz, $CDCl_3$) δ(ppm):7.40 – 7.28 (m, 5H, $Ar\underline{H}$), 5.28 (s, 2H, $C\underline{H_2}Ph$), 3.72 (s, 6H, $C(CO_2C\underline{H_3})$), 2.80 (tt, *J* = 10.0, 4.0 Hz, 1H, $C\underline{H}C{=}CC({=}O)$), 1.97 – 1.89 (m, 2H, $C\underline{H_A}H_BCHC\underline{H_C}H_D$), 1.71 (m, 2H, $C\underline{H_A}H_B$-$CH_2CHCH_2C\underline{H_C}H_D$), 1.65 – 1.56 (m, 2H, cyclohexyl ring), 1.50 (m, 2H, $CH_A\underline{H_B}CHCH_C\underline{H_D}$), 1.42 – 1.24 (m, 2H cyclohexyl ring); **^{13}C NMR** (126 MHz, $CDCl_3$) δ(ppm): 169.7 ($C(\underline{C}O_2CH_3)$), 157.3 ($CHC{=}C\underline{C}({=}O)$), 135.3 ($Ar\underline{C}$), 128.7 ($Ar\underline{C}H$), 128.5 ($Ar\underline{C}H$), 128.2 ($\underline{C}{=}CC({=}O)$), 128.1 ($Ar\underline{C}H$), 97.7 ($C{=}\underline{C}C({=}O)$), 67.5 ($\underline{C}H_2Ph$), 52.6 ($C(CO_2\underline{C}H_3)$), 36.2 ($\underline{C}(CO_2CH_3)$), 35.1 ($\underline{C}HC{=}CC({=}O)$), 29.8 ($CH\underline{C}H_2CH_2CH_2$), 25.7 ($CHCH_2CH_2\underline{C}H_2$), 25.1 ($CHCH_2\underline{C}H_2CH_2$); **IR** (powder) ν_{max}/cm^{-1}: 2933, 2856 (C-H), 1720 (C=O); **HRMS** (ES+) exact mass calculated for $[M+H]^+$ ($C_{21}H_{25}O_6^+$) requires *m/z* 373.1646, found *m/z* 373.1646; **MP**: 34 – 36 °C.

Synthesis and characterisation of 5a

2-benzyl 1,1-dimethyl 3-cyclopentylcycloprop-2-ene-1,1,2-tricarboxylate (5a)

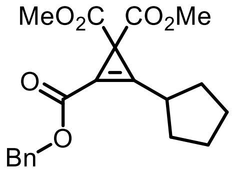

Compound **5a** was synthesised according to general procedure **V** by the addition of **S11** (250 mg, 1.11 mmol, 1.00 eq) to benzyl chloroformate (285 mg, 1.67 mmol, 1.50 eq). The crude product was purified by silica gel column chromatography (pentane/Et_2O = 9/1 (use CH_2Cl_2 TLC to check the fractions)) to provide the title compound (**5a**) as a colourless oil in 22% yield (87 mg). **^{1}H NMR** (500 MHz, $CDCl_3$) δ(ppm): 7.41 – 7.28 (m, 5H, ArH), 5.28 (s, 2H, CH_2Ph), 3.72 (s, 6H, $C(CO_2CH_3)_2$), 3.19 (tt, *J* = 8.0, 6.5 Hz, 1H, CHC=C), 2.01 – 1.91 (m, 2H, $CH_AH_BCHCH_CH_D$), 1.82 – 1.74 (m, 2H, $CH_AH_BCHCH_CH_D$), 1.74 – 1.66 (m, 2H, $CH_AH_BCH_2CHCH_2CH_CH_D$), 1.66 – 1.59 (m, 2H, $CH_AH_BCH_2CHCH_2CH_CH_D$); **^{13}C NMR** (126 MHz, $CDCl_3$) δ(ppm): 169.6 ($C(CO_2CH_3)_2$), 157.3 (CO_2Bn), 135.3 (ArC), 128.7 (ArCH), 128.7 (C=CC(=O)Bn), 128.5 (ArCH), 128.1 (ArCH), 97.3 (C=CC(=O)Bn), 67.5 (CH_2Ph), 52.6 ($C(CO_2CH_3)_2$), 36.7 ($C(CO_2Me)$), 36.1 (CHC=C), 31.1 (CH_2CHCH_2), 25.3 ($CH_2CH_2CHCH_2CH_2$); **IR** (film) v_{max}/cm^{-1}:2954 (C-H), 1739, 1732 (C=O); **HRMS** (ES+) exact mass calculated for $[M+H]^+$ ($C_{20}H_{23}O_6^+$) requires *m/z* 359.1489, found *m/z* 359.1494.

Synthesis and characterisation of 6a

2-benzyl 1,1-dimethyl 3-(1-(tert-butoxycarbonyl)piperidin-4-yl)cycloprop-2-ene-1,1,2-tricarboxylate (6a)

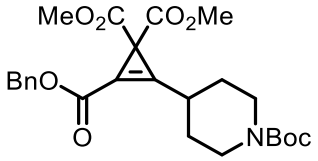

Compound **6a** was synthesised according to general procedure **V** by the addition of **S12** (188 mg, 0.55 mmol, 1.00 eq) to benzyl chloroformate (0.12 ml, 0.83 mmol, 1.50 eq). The crude product was purified by silica gel column chromatography (pentane/Et_2O = 1:1) to provide the title compound (**6a**) as a white solid in 57% yield (150 mg). **^{1}H NMR** (400 MHz, $CDCl_3$) δ(ppm)7.41 – 7.32 (m, 5H, ArH), 5.34 – 5.21 (m, 2H, CH_2Ph), 3.86 – 3.78 (m, 2H, $CH_AH_BN(Boc)CH_CH_D$, 3.73 (s, 6H, $C(CO_2CH_3)$), 3.08 (ddd, *J* = 13.4, 9.7, 3.2 Hz, 2H, $CH_AH_BN(Boc)CH_CH_D$), 2.98 (tt, *J* = 9.0, 4.1 Hz, 1H, CHC=C), 1.97 – 1.86 (m, 2H, $CH_AH_BCH_2N(Boc)CH_2CH_CH_D$), 1.74 – 1.64 (m, 2H, $CH_AH_BCH_2N(Boc)CH_2CH_CH_D$), 1.45 (s, 9H, $C(CH_3)_3$); **^{13}C NMR** (101 MHz, $CDCl_3$) δ(ppm)169.4 ($C(CO_2CH_3)$), 156.9 (CO_2Bn), 154.6 ($NCO_2C(CH_3)_3$), 135.1 (ArC), 128.7 (ArCH), 128.6 (ArCH), 128.2 ($C=CCO_2Bn$), 126.4 (ArCH), 99.3($C=CCO_2Bn$), 79.8 ($NCO_2C(CH_3)_3$), 67.7 (CH_2Ph), 52.7 ($C(CO_2CH_3)$), 42.6 (CH_2NCH_2), 36.3 ($C(CO_2CH_3)$), 33.2 (CHC=C), 28.8 ($CH_2CH_2NCH_2CH_2$), 28.5 ($NCO_2C(CH_3)_3$); **HRMS** (ES+) exact mass calculated for $[M+Na]^+$ ($C_{25}H_{31}NO_8Na^+$) requires *m/z* 496.1942, found *m/z* 496.1930.

Synthesis and characterisation of 7a

2-benzyl 1,1-dimethyl 3-(tetrahydro-2H-pyran-4-yl)cycloprop-2-ene-1,1,2-tricarboxylate (7a)

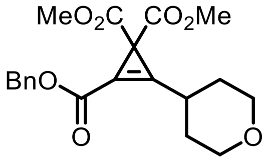

Compound **7a** was synthesised according to general procedure **V** by the addition of **S13** (340 mg, 1.42 mmol, 1.00 eq) to benzyl chloroformate (0.71 mL, 2.13 mmol, 1.50 eq, 3 M in toluene). The crude product was purified by silica gel column chromatography (pent/EtOAc = 3/1) to provide the title compound (**7a**) as a colourless oil in 54% yield (287 mg). **^{1}H NMR** (400 MHz, $CDCl_3$) δ(ppm): 7.43 – 7.30 (m, 5H, Ar$\underline{H}$), 5.28 (s, 2H, $C\underline{H_2}Ph$), 3.90 (t, *J* = 4.3 Hz, 1H, one of $C\underline{H_A}H_BOCH_CH_D$), 3.87 (t, *J* = 4.3 Hz, 1H, one of $CH_AH_BOC\underline{H_C}H_D$), 3.73 (s, 6H, $C(COC\underline{H_3})_2$), 3.52 (ddd, *J* = 12.0, 9.5, 2.8 Hz, 2H, 2 of $CH_A\underline{H_B}OCH_C\underline{H_D}$), 3.06 (tt, *J* = 9.2, 4.3 Hz, 1H, $C\underline{H}C{=}CC({=}O)$), 1.98 – 1.88 (m, 2H, 2 of $C\underline{H_A}H_BCH_2OCH_2C\underline{H_C}H_D$), 1.79 (dtd, *J* = 13.5, 9.5, 3.9 Hz, 2H, 2 of $CH_A\underline{H_B}CH_2OCH_2CH_C\underline{H_D}$); **^{13}C NMR** (101 MHz, $CDCl_3$) δ(ppm): 169.4 ($C(\underline{C}O_2CH_3)$), 157.0 ($CHC{=}C\underline{C}({=}O)$), 135.1 (Ar$\underline{C}$), 128.7 (Ar$\underline{C}$H), 128.6 (Ar$\underline{C}$H), 128.2 (Ar$\underline{C}$H), 126.5 ($\underline{C}{=}CC({=}O)$), 99.0 ($C{=}\underline{C}C({=}O)$), 67.7 ($\underline{C}H_2Ph$), 66.6 ($CH_2\underline{C}H_2O\underline{C}H_2CH_2$), 52.7 ($C(CO_2\underline{C}H_3)$), 36.3 ($\underline{C}(CO_2CH_3)$), 32.2 ($\underline{C}HC{=}CC({=}O)$), 29.5 ($\underline{C}H_2CH_2OCH_2\underline{C}H_2$); **HRMS** (ES+) exact mass calculated for $[M+H]^+$ ($C_{20}H_{23}O_7^+$) requires *m/z* 375.1438, found *m/z* 375.1441.

Synthesis and characterisation of 8a

2-allyl 1,1-dimethyl 3-cyclohexylcycloprop-2-ene-1,1,2-tricarboxylate (8a)

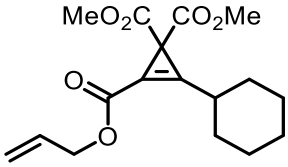

Compound **8a** was synthesised according to general procedure **V** by the addition of **S9** (200 mg, 0.84 mmol, 1.00 eq) to allyl chloroformate (0.130 ml, 1.26 mmol, 1.50 eq). The crude product was purified by silica gel column chromatography (pentane/acetone = 97/3 to 95/5) to provide the title compound (**8a**) as a colourless oil in 62% yield (168 mg). **^{1}H NMR** (500 MHz, $CDCl_3$) δ(ppm): 5.93 (ddt, *J* = 17.0, 10.5, 5.5 Hz, 1H, C<u>H</u>=CH_2), 5.35 (app-dq, *J* = 17.0, 1.5 Hz, 1H, one of C=C$\underline{H_A}H_B$), 5.27 (app-dq, *J* = 10.5, 1.5 Hz, 1H, one of C=C$\underline{H_A}H_B$), 4.74 (dt, *J* = 5.5, 1.5 Hz, 2H, C$\underline{H_2}$CH=CH_2), 3.73 (s, 6, C($CO_2C\underline{H_3}$)$_2$), 2.81 (tt, *J* = 10.0, 2.0 Hz, 1H, C<u>H</u>C=CC(=O)), 1.99 – 1.90 (m, 2H, cyclohexyl ring), 1.73 (dq, *J* = 13.5, 4.5 Hz, 2H, cyclohexyl ring), 1.65 – 1.45 (m, 3H, cyclohexyl ring), 1.41 – 1.22 (m, 3H, cyclohexyl ring); **^{13}C NMR** (126 MHz, $CDCl_3$) δ(ppm): 169.7 (C(<u>C</u>O_2CH_3)$_2$), 157.1 (<u>C</u>(=O)OCH_2CH), 131.4 (<u>C</u>H=CH_2), 128.0 (<u>C</u>=CC(=O)), 118.7 (CH=<u>C</u>H_2), 97.6 (C=<u>C</u>C(=O)), 66.4 (<u>C</u>H_2CH=CH_2), 52.6 (C(CO_2<u>C</u>H_3)$_2$), 36.2 (<u>C</u>(CO_2CH_3)$_2$), 35.1 (<u>C</u>HC=CC(=O)), 29.8 (CH<u>C</u>$H_2CH_2CH_2$), 25.7 (CHCH_2CH_2<u>C</u>H_2), 25.1 (CHCH_2<u>C</u>H_2CH_2); **IR** (film) v_{max}/cm^{-1}: 2933, 2856 (C-H), 1721 (C=O); **HRMS** (ES+) exact mass calculated for $[M+Na]^+$ ($C_{17}H_{22}NaO_6^+$) requires *m/z* 345.1309, found *m/z* 345.1305.

Synthesis and characterisation of 9a

2-benzyl 1,1-dimethyl 3-butylcycloprop-2-ene-1,1,2-tricarboxylate (9a)

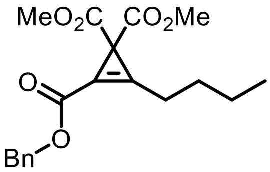

Compound **9a** was synthesised according to general procedure **V** by the addition of **S10** (250 mg, 1.18 mmol, 1.00 eq) to benzyl chloroformate (0.250 ml, 1.77 mmol, 1.50 eq). The crude product was purified by silica gel column chromatography (1st column: pentane/Et_2O = 8/2, 2nd column: pentane/Et_2O = 85/15 to 8/2) to provide the title compound (**9a**) as a yellow oil in 46% yield (187 mg). **^{1}H NMR** (500 MHz, $CDCl_3$) δ(ppm): 7.40 – 7.30 (m, 5H, Ar$\underline{H}$), 5.28 (s, 2H, C$\underline{H_2}$Ph), 3.72 (s, 6H, $C(CO_2C\underline{H_3})_2$), 2.69 (t, J = 7.5 Hz, 2H, $C\underline{H_2}CH_2CH_2CH_3$), 1.67 (app-p, J = 7.5 Hz, 2H, $CH_2C\underline{H_2}CH_2CH_3$), 1.41 (app-dq, J = 14.5, 7.5 Hz, 2H, $CH_2CH_2C\underline{H_2}CH_3$), 0.90 (t, J = 7.5 Hz, 3H, $CH_2CH_2CH_2C\underline{H_3}$); **^{13}C NMR** (126 MHz, $CDCl_3$) δ(ppm): 169.6 ($C(\underline{C}O_2CH_3)_2$), 157.3 ($\underline{C}O_2Bn$), 135.2 (Ar$\underline{C}$), 128.7 (Ar$\underline{C}$H), 128.6 (Ar$\underline{C}$H), 128.2 (Ar$\underline{C}$H), 125.4 ($\underline{C}$=CC(=O)), 98.6 (C=$\underline{C}$C(=O)), 67.7 ($\underline{C}H_2Ph$), 52.7 ($C(CO_2\underline{C}H_3)_2$), 36.4 ($\underline{C}(CO_2CH_3)_2$), 28.4 ($CH_2\underline{C}H_2CH_2CH_3$), 25.0 ($\underline{C}H_2CH_2CH_2CH_3$), 22.3 ($CH_2CH_2\underline{C}H_2CH_3$), 13.7 ($CH_2CH_2CH_2\underline{C}H_3$); **IR** (film) ν_{max}/cm^{-1}: 2956, 2874 (C-H), 1879, 1720 (C=O); **HRMS** (ES+) exact mass calculated for $[M+H]^+$ ($C_{19}H_{23}O_6^+$) requires *m/z* 347.1489, found *m/z* 347.1495.

Synthesis and characterisation of 10a

trimethyl 3-butylcycloprop-2-ene-1,1,2-tricarboxylate (10a)

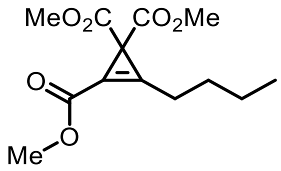

Compound **10a** was synthesised according to general procedure **V** by the addition of **S10** (250 mg, 1.18 mmol, 1.00 eq) to methyl chloroformate (0.140 ml, 1.77 mmol, 1.50 eq). The crude product was purified by silica gel column chromatography (pentane/Et_2O = 9/1 to 85/15) to provide the title compound (**10a**) as a colourless oil in 46% yield (146 mg). **^{1}H NMR** (500 MHz, $CDCl_3$) δ(ppm): 3.84 (s, 3H, C=CC(=O)OC$\underline{H_3}$), 3.73 (s, 6H, C(CO_2C$\underline{H_3}$)$_2$), 2.69 (t, *J* = 7.5 Hz, 2H, C$\underline{H_2}$$CH_2CH_2CH_3$), 1.73 – 1.63 (m, 1H, CH_2C$\underline{H_2}$$CH_2CH_3$), 1.48 – 1.37 (m, 2H, CH_2CH_2C$\underline{H_2}$$CH_3$), 0.93 (t, *J* = 7.5 Hz, 3H, $CH_2CH_2CH_2$C$\underline{H_3}$); **^{13}C NMR** (126 MHz, $CDCl_3$) δ(ppm): 169.6 (C(CO_2C$\underline{H_3}$)$_2$), 157.9 (C=C$\underline{C}$(=O)), 125.3 ($\underline{C}$=CC(=O)), 98.5 (C=$\underline{C}$C(=O)), 52.9 (C=CC(=O)O$\underline{C}H_3$), 52.7 (C($CO_2$$\underline{C}H_3$)$_2$), 36.4 ($\underline{C}$($CO_2CH_3$)$_2$), 28.4 ($CH_2$$\underline{C}H_2$$CH_2CH_3$), 24.9 ($\underline{C}H_2$$CH_2CH_2CH_3$), 22.3 ($CH_2CH_2$$\underline{C}H_2$$CH_3$), 13.7 ($CH_2CH_2CH_2$$\underline{C}H_3$); **IR** (film) v_{max}/cm^{-1}: 2958, 2875 (C-H), 1721 (C=O); **HRMS** (ES+) exact mass calculated for $[M+H]^+$ ($C_{13}H_{19}O_6^+$) requires *m/z* 271.1176, found *m/z* 271.1175.

Synthesis and characterisation of 11a

2-isobutyl 1,1-dimethyl 3-butylcycloprop-2-ene-1,1,2-tricarboxylate (11a)

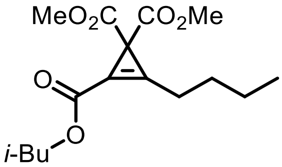

Compound **11a** was synthesised according to general procedure **V** by the addition of **S10** (250 mg, 1.18 mmol, 1.00 eq) to isobutyl chloroformate (0.230 ml, 1.77 mmol, 1.50 eq). The crude product was purified by silica gel column chromatography (pentane/Et_2O = 9/1 to 85/15) to provide the title compound (**11a**) as a colourless oil in 43% yield (159 mg). **^{1}H NMR** (500 MHz, $CDCl_3$) δ(ppm): 4.02 (d, *J* = 6.5 Hz, 2H, $OC\underline{H_2}$), 3.73 (s, 6H, $C(CO_2C\underline{H_3})_2$), 2.69 (t, *J* = 7.5 Hz, 2H, $C\underline{H_2}CH_2CH_2CH_3$), 2.00 (hep, *J* = 13.5, 6.5 Hz, 1H, $C\underline{H}(CH_3)_2$), 1.74 – 1.65 (m, 2H, $CH_2C\underline{H_2}CH_2CH_3$), 1.49 – 1.39 (m, 2H, $CH_2CH_2C\underline{H_2}CH_3$), 0.96 – 0.90 (m, 9H, $CH_2CH_2CH_2C\underline{H_3}$ and $CH(C\underline{H_3})_2$); **^{13}C NMR** (126 MHz, $CDCl_3$) δ(ppm): 169.5 ($C(\underline{C}O_2Me)_2$), 157.4 (C=C$\underline{C}$(=O)), 124.5 ($\underline{C}$=CC(=O)), 98.6 (C=$\underline{C}$C(=O)), 71.8 ($O\underline{C}H_2$), 52.5 ($C(CO_2\underline{C}H_3)_2$), 36.3 ($\underline{C}(CO_2CH_3)_2$), 28.3 ($CH_2\underline{C}H_2CH_2CH_3$), 27.7 ($\underline{C}H(CH_3)_2$), 24.8 ($\underline{C}H_2CH_2CH_2CH_3$), 22.1 ($CH_2CH_2\underline{C}H_2CH_3$), 18.9 ($CH(\underline{C}H_3)_2$), 13.6 ($CH_2CH_2CH_2\underline{C}H_3$); **IR** (film) ν_{max}/cm^{-1}: 2959, 2876 (C-H), 1719 (C=O); **HRMS** (ES+) exact mass calculated for $[M+Na]^+$ ($C_{16}H_{24}NaO_6^+$) requires *m/z* 335.1465, found *m/z* 335.1461.

Synthesis and characterisation of 12a

2-benzyl 1,1-dimethyl 3-(3-chloropropyl)cycloprop-2-ene-1,1,2-tricarboxylate (12a)

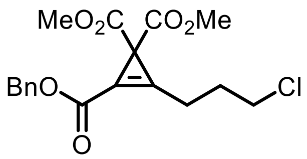

Compound **12a** was synthesised according to general procedure **V** by the addition of **S14** (535 mg, 1.46 mmol, 1.00 eq) to benzyl chloroformate (0.73 ml, 2.19 mmol, 1.50 eq, 3 M in toluene). The crude product was purified by silica gel column chromatography (CH_2Cl_2/Et_2O = 199:1) to provide the title compound (**12a**) as a colourless oil in 18% yield (96 mg). **^{1}H NMR** (400 MHz, $CDCl_3$) δ(ppm): 7.42 – 7.30 (m, 5H, ArH,), 5.28 (s, 2H C$\underline{H_2}$Ph), 3.73 (s, 6H, C($CO_2C\underline{H_3}$)$_2$), 3.64 (t, *J* = 6.3 Hz, 2H, $CH_2CH_2C\underline{H_2}Cl$), 2.88 (t, *J* = 7.0 Hz, 2H, $\underline{CH_2}CH_2CH_2Cl$), 2.25 – 2.14 (m, 2H, $CH_2C\underline{H_2}CH_2Cl$); **^{13}C NMR** (101 MHz, $CDCl_3$) δ(ppm): 169.3 (C($\underline{C}O_2CH_3$)$_2$), 157.0 ($\underline{C}O_2$Bn), 135.0 (Ar$\underline{C}$), 128.8 (Ar$\underline{C}$H), 128.6 (Ar$\underline{C}$H), 128.3 (Ar$\underline{C}$H), 124.0 ($\underline{C}$=CC(=O)), 99.8 (C=$\underline{C}$C(=O)), 67.7 ($\underline{C}H_2$Ph), 52.8 (C($CO_2\underline{C}H_3$)$_2$), 43.5 ($CH_2CH_2\underline{C}H_2Cl$), 36.5 ($\underline{C}$($CO_2CH_3$)$_2$), 29.3 ($\underline{C}H_2CH_2CH_2Cl$), 22.6 ($CH_2\underline{C}H_2CH_2Cl$); **HRMS** (ES+) exact mass calculated for $[M+H]^+$ ($C_{18}H_{20}ClO_6^+$) requires *m/z* 367.0943, found *m/z* 367.0932;

Synthesis and characterisation of 13a

2-benzyl 1,1-dimethyl 3-(3-((tert-butyldimethylsilyl)oxy)propyl)cycloprop-2-ene-1,1,2-tricarboxylate (13a)

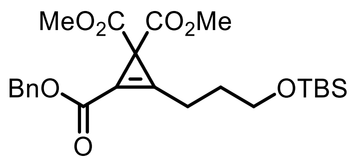

Compound **13a** was synthesised according to general procedure **V** by the addition of **S15** (1.64 g, 5.00 mmol, 1.00 eq) to benzyl chloroformate (2.5 ml, 7.5 mmol, 1.50 eq, 3 M in toluene). The crude product was purified by silica gel column chromatography (pent/EtOAc = 10/1) to provide the title compound (**13a**) as a colourless oil in 78% yield (1.8 g). **^{1}H NMR** (400 MHz, $CDCl_3$) δ(ppm): 7.39 – 7.32 (m, 5H, ArH), 5.28 (s, 2H, CH_2Ph), 3.72 (s, 6H, $C(CO_2CH_3)_2$), 3.67 (t, *J* = 5.9 Hz, 2H, $TBSOCH_2$), 2.78 (t, *J* = 7.3 Hz, 2H, $CH_2CH_2CH_2OTBS$), 1.89 (tt, *J* = 7.3, 5.8 Hz, 2H, $CH_2CH_2CH_2OTBS$), 0.87 (s, 9H, $Si(CH_3)_2C(CH_3)_3$)), 0.02 (s, 6H, $Si(CH_3)_2C(CH_3)_3$)); **^{13}C NMR** (101 MHz, $CDCl_3$) δ(ppm): 169.4 ($C(CO_2CH_3)_2$), 157.2 (CO_2Bn), 135.2 (ArC), 128.7 (ArCH), 128.6 (ArCH), 128.2 (ArCH), 125.3 (C=CC(=O)), 98.8 (C=CC(=O)), 67.6 (CH_2Ph), 61.5 ($CH_2CH_2CH_2OTBS$), 52.6 ($C(CO_2CH_3)_2$), 36.4 ($C(CO_2CH_3)_2$), 29.5 ($CH_2CH_2CH_2OTBS$), 26.0 ($Si(CH_3)_2C(CH_3)_3$), 21.8 ($CH_2CH_2CH_2OTBS$), 18.4 ($Si(CH_3)_2C(CH_3)_3$) ($Si(CH_3)_2C(CH_3)_3$), -5.3 ($Si(CH_3)_2C(CH_3)_3$); **HRMS** (ES+) exact mass calculated for $[M+H]^+$ ($C_{24}H_{35}O_7Si^+$) requires *m/z* 463.2147, found *m/z* 463.2143.

Synthesis and characterisation of 14a

2-benzyl 1,1-dimethyl 3-(3-phenylpropyl)cycloprop-2-ene-1,1,2-tricarboxylate (14a)

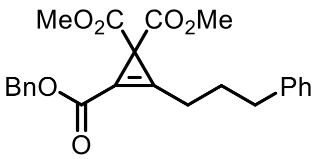

Compound **14a** was synthesised according to general procedure **V** by the addition of **S16** (208 mg, 0.76 mmol, 1.00 eq) to benzyl chloroformate (0.39 mL, 1.14 mmol, 1.50 eq, 3 M in toluene). The crude product was purified by silica gel column chromatography (pent/EtOAc = 10/1) to provide the title compound (**14a**) as a colourless oil in 65% yield (200 mg). **^{1}H NMR** (500 MHz, $CDCl_3$) δ(ppm): 7.40 – 7.34 (m, 5H, ArH of Bn), 7.30 – 7.25 (m, 2H, ArH), 7.23 – 7.18 (m, 1H, ArH), 7.18 – 7.13 (m, 2H, ArH), 5.31 (s, 2H, $C\underline{H_2}Ph$), 3.74 (s, 6H, $C(CO_2C\underline{H_3})_2$), 2.77 – 2.64 (m, 4H, $C\underline{H_2}CH_2C\underline{H_2}Ph$), 2.10 – 1.99 (m, 2H, $CH_2C\underline{H_2}CH_2Ph$); **^{13}C NMR** (126 MHz, $CDCl_3$) δ(ppm): 169.5 ($C(\underline{C}O_2CH_3)_2$), 157.2 ($\underline{C}O_2Bn$), 141.0 (ArC), 135.2 (ArC), 128.7 (ArCH), 128.6 (ArCH), 128.6 (ArCH), 128.2 (ArCH), 126.2 (ArCH), 124.9 (C=CC(=O)), 99.0 (C=CC(=O)), 67.6 ($\underline{C}H_2Ph$), 52.7 ($C(CO_2\underline{C}H_3)_2$), 36.4 ($\underline{C}(CO_2CH_3)_2$), 35.0 ($CH_2CH_2\underline{C}H_2Ph$), 28.0 ($\underline{C}H_2CH_2CH_2Ph$), 24.7 ($CH_2\underline{C}H_2CH_2Ph$); **HRMS** (ES+) exact mass calculated for $[M+H]^+$ ($C_{24}H_{25}O_6^+$) requires *m/z* 409.1646, found *m/z* 409.1640.

Synthesis and characterisation of 15a

2-benzyl 1,1-dimethyl 3-ethylcycloprop-2-ene-1,1,2-tricarboxylate (15a)

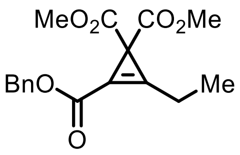

Compound **15a** was synthesised according to general procedure **V** by the addition of **S18** (153 mg, 0.83 mmol, 1.00 eq) to benzyl chloroformate (0.42 mL, 1.25 mmol, 1.50 eq, 3 M in toluene). The crude product was purified by silica gel column chromatography (pent/EtOAc = 10/1) to provide the title compound (**15a**) as a colourless oil in 15% yield (39 mg). **^{1}H NMR** (400 MHz, $CDCl_3$) δ(ppm): 7.40 – 7.28 (m, 5H, Ar$\underline{H}$), 5.28 (s, 2H, $C\underline{H_2}Ph$), 3.73 (s, 6H, $C(CO_2C\underline{H_3})_2$), 2.72 (q, *J* = 7.5 Hz, 2H, $C\underline{H_2}CH_3$), 1.28 (t, *J* = 7.4 Hz, 3H, $CH_2C\underline{H_3}$); **^{13}C NMR** (126 MHz, $CDCl_3$) δ(ppm): 169.6 ($C(\underline{C}O_2CH_3)_2$), 157.2 ($\underline{C}O_2Bn$), 135.2 (Ar$\underline{C}$), 128.7 (Ar$\underline{C}$H), 128.6 (Ar$\underline{C}$H), 128.2 (Ar$\underline{C}$H), 126.2 ($\underline{C}$=CC(=O)), 98.5 (C=$\underline{C}$C(=O)), 67.5 ($\underline{C}H_2Ph$), 52.7 ($C(CO_2\underline{C}H_3)_2$), 36.5 ($\underline{C}(CO_2CH_3)_2$), 19.0 ($\underline{C}H_2CH_3$), 10.9 ($CH_2\underline{C}H_3$); **HRMS** (ES+) exact mass calculated for $[M+ Na]^+$ ($C_{17}H_{18}O_6Na^+$) requires *m/z* 341.0996, found *m/z* 341.0984.

Synthesis and characterisation of 16a

2-benzyl 1,1-dimethyl 3-(2-((tert-butyldimethylsilyl)oxy)ethyl)cycloprop-2-ene-1,1,2-tricarboxylate (16a)

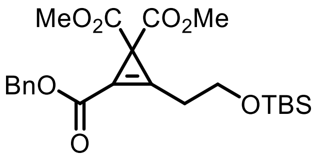

Compound **16a** was synthesised according to general procedure **V** by the addition of **S19** (645 mg, 2.05 mmol, 1.00 eq) to benzyl chloroformate (1.1 ml, 3.08 mmol, 1.50 eq, 3 M in toluene). The crude product was purified by silica gel column chromatography (pent/EtOAc = 10/1) to provide the title compound (**16a**) as a colourless oil in 48% yield (500 mg). **^{1}H NMR** (400 MHz, $CDCl_3$) δ(ppm): 7.38 – 7.30 (m, 5H, Ar$\underline{H}$), 5.27 (s, 2H, $C\underline{H_2}Ph$), 3.90 (t, *J* = 6.8 Hz, 2H, $CH_2C\underline{H_2}OTBS$), 3.72 (s, 6H, $C(CO_2C\underline{H_3})_2$), 2.90 (t, *J* = 6.8 Hz, 2H, $C\underline{H_2}CH_2OTBS$), 0.85 (s, 9H, $Si(CH_3)_2C(C\underline{H_3})_3$), 0.03 (s, 6H, $Si(C\underline{H_3})_2C(CH_3)_3$); **^{13}C NMR** (101 MHz, $CDCl_3$) δ(ppm): 169.4 ($\underline{C}O_2Bn$), 157.1 $C((\underline{C}O_2Me)_2)$, 135.1 (Ar$\underline{C}$), 128.7 (Ar$\underline{CH}$), 128.6 (Ar$\underline{CH}$), 128.2 (Ar$\underline{CH}$), 122.5 ($\underline{C}$=CC(=O)), 99.7 (C=$\underline{C}$C(=O)), 67.6 ($\underline{C}H_2Ph$), 59.6 ($CH_2\underline{C}H_2OTBS$), 52.6 ($C(CO_2\underline{C}H_3)_2$), 36.3 ($\underline{C}(CO_2CH_3)_2$), 29.0 ($\underline{C}H_2CH_2OTBS$), 25.9 ($Si(CH_3)_2\underline{C}(CH_3)_3$), 18.3 ($Si(CH_3)_2C(\underline{C}H_3)_3$), -5.4 ($Si(\underline{C}H_3)_2C(CH_3)_3$); **HRMS** (ES+) exact mass calculated for $[M+H]^+$ ($C_{23}H_{33}O_7Si^+$) requires *m/z* 449.1990, found *m/z* 449.1983.

Synthesis and characterisation of 17a

2-benzyl 1,1-dimethyl 3-phenethylcycloprop-2-ene-1,1,2-tricarboxylate (17a)

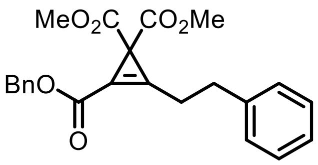

Compound **17a** was synthesised according to general procedure **V** by the addition of **S20** (580 mg, 2.23 mmol, 1.00 eq) to benzyl chloroformate (1.1 ml, 3.35 mmol, 1.50 eq, 3 M in toluene). The crude product was purified by silica gel column chromatography (1st column: CH_2Cl_2/Et_2O = 50/1, 2nd column: pent/EtOAc = 10/1) to provide the title compound (**17a**) as a colourless oil in 16% yield (143 mg). **^{1}H NMR** (400 MHz, $CDCl_3$) δ(ppm): 7.40 – 7.32 (m, 5H, ArH), 7.31 – 7.26 (m, 1H, ArH), 7.26 – 7.24 (m, 1H, ArH), 7.23 – 7.15 (m, 3H, ArH), 5.28 (s, 2H, CH_2Ph), 3.69 (s, 6H, $C(CO_2CH_3)_2$), 3.03 (s, 4H, CH_2CH_2Ph); **^{13}C NMR** (101 MHz, $CDCl_3$) δ(ppm): 169.4 ($C(CO_2CH_3)_2$), 157.1 (CO_2Me), 139.7 (ArC), 135.2 (ArC), 128.7 (ArCH), 128.7 (ArCH), 128.6 (ArCH), 128.3 (ArCH), 128.2 (ArCH), 126.6 (ArCH), 124.3 (C=CC(=O)), 99.4 (C=CC(=O)), 67.6 (CH_2Ph), 52.7 ($C(CO_2CH_3)_2$), 36.6 ($C(CO_2CH_3)_2$), 32.3 (CH_2CH_2Ph), 26.7 (CH_2CH_2Ph); **HRMS** (ES+) exact mass calculated for $[M+H]^+$ ($C_{23}H_{23}O_6^+$) requires *m/z* 395.1489, found *m/z* 395.1479.

Synthesis and characterisation of 18a

2-benzyl 1,1-diethyl 3-butylcycloprop-2-ene-1,1,2-tricarboxylate (18a)

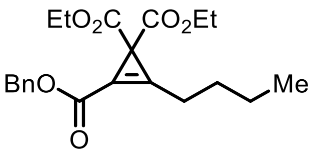

Compound **18a** was synthesised according to general procedure **V** by the addition of **S21** (402 mg, 0.55 mmol, 1.00 eq) to benzyl chloroformate (0.84 ml, 2.57 mmol, 1.50 eq, 3 M in toluene). The crude product was purified by silica gel column chromatography (pentane/Et_2O = 5:1) to provide the title compound (**18a**) as a colourless oil in 34% yield (230 mg). **^{1}H NMR** (400 MHz, $CDCl_3$) δ(ppm): 7.37 – 7.29 (m, 5H, Ar$\underline{H}$), 5.27 (s, 2H, $C\underline{H_2}Ph$), 4.18 (q, J = 7.1 Hz, 4H, $C(CO_2C\underline{H_2}CH_3)_{\underline{2}}$), 2.69 (t, J = 7.3 Hz, 2H, $C\underline{H_2}CH_2CH_2CH_3$), 1.67 (tt, J = 7.4, 6.4 Hz, 2H, $CH_2C\underline{H_2}CH_2CH_3$), 1.47 – 1.34 (m, 2H, $CH_2CH_2C\underline{H_2}CH_3$), 1.23 (t, J = 7.1 Hz, 6H, $C(CO_2CH_2C\underline{H_3})_{\underline{2}}$), 0.89 (t, J = 7.3 Hz, 3H, $CH_2CH_2CH_2C\underline{H_3}$); **^{13}C NMR** (101 MHz, $CDCl_3$) δ(ppm): 169.2 ($C(\underline{C}O_2Et)_2$), 157.4 ($\underline{C}O_2Bn$), 135.3 (Ar$\underline{C}$), 128.7 (Ar$\underline{C}$H), 128.5 (Ar$\underline{C}$H), 128.2 (Ar$\underline{C}$H), 125.6 ($\underline{C}$=CC(=O)), 98.7 (C=$\underline{C}$C(=O)), 67.4 ($\underline{C}H_2Ph$), 61.4 ($C(CO_2\underline{C}H_2CH_3)_{\underline{2}}$), 36.8 ($\underline{C}(CO_2Et)_2$), 28.4 ($\underline{C}H_2CH_2CH_2CH_3$), 24.9 ($CH_2\underline{C}H_2CH_2CH_3$), 22.2 ($CH_2CH_2\underline{C}H_2CH_3$), 14.2 ($C(CO_2CH_2\underline{C}H_3)_{\underline{2}}$), 13.6 ($CH_2CH_2CH_2\underline{C}H_3$); **HRMS** (ES+) exact mass calculated for $[M+ H]^+$ ($C_{21}H_{27}O_6^+$) requires *m/z* 375.1802, found *m/z* 375.1801.

Synthesis and characterisation of 19a

2-benzyl 1,1-diisopropyl 3-butylcycloprop-2-ene-1,1,2-tricarboxylate (19a)

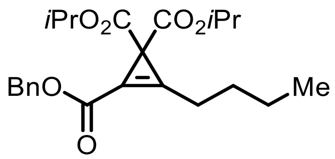

Compound **19a** was synthesised according to general procedure **V** by the addition of **S22** (204 mg, 0.76 mmol, 1.00 eq) to benzyl chloroformate (0.38 mL, 1.14 mmol, 1.50 eq, 3 M in toluene). The crude product was purified by silica gel column chromatography (pent/EtOAc = 20/1) to provide the title compound (**19a**) as a colourless oil in 44% yield (135 mg). **^{1}H NMR** (400 MHz, $CDCl_3$) δ(ppm): 7.40 – 7.30 (m, 5H, Ar$\underline{H}$), 5.27 (d, *J* = 1.7 Hz, 2H, $C\underline{H_2}Ph$), 5.11 – 4.97 (m, 2H, $C(CO_2(C\underline{H}(CH_3)_2)_{\underline{2}})$), 2.79 – 2.58 (m, 2H, $C\underline{H_2}CH_2CH_2CH_3$), 1.75 – 1.62 (m, 2H, $CH_2C\underline{H_2}CH_2CH_3$), 1.42 (dtd, *J* = 14.4, 7.6, 3.7 Hz, 2H, $CH_2CH_2C\underline{H_2}CH_3$), 1.21 (dd, *J* = 6.1, 1.6 Hz, 12H, $C(CO_2(CH(C\underline{H_3})_{\underline{2}})_{\underline{2}})$), 0.90 (td, *J* = 7.5, 1.5 Hz, 3H, $CH_2CH_2CH_2C\underline{H_3}$); **^{13}C NMR** (101 MHz, $CDCl_3$) δ(ppm): 168.9 ($C(\underline{C}O_2{}^iPr)_{\underline{2}}$), 157.6 ($\underline{C}O_2Bn$), 135.4, 128.6 (Ar$\underline{C}$H), 128.4 (Ar$\underline{C}$H), 128.2 (Ar$\underline{C}$H), 125.9 ($\underline{C}$=CC(=O)), 99.0 (C=$\underline{C}$C(=O)), 68.9 ($C(CO_2(\underline{C}H(CH_3)_2)_{\underline{2}})$), 67.3 ($\underline{C}H_2Ph$), 37.4 ($\underline{C}(CO_2CH_3)_2$), 28.4 ($\underline{C}H_2CH_2CH_2CH_3$), 24.9 ($CH_2\underline{C}H_2CH_2CH_3$), 22.2 ($CH_2CH_2\underline{C}H_2CH_3$), 21.8 ($C(CO_2(CH(\underline{C}H_3)_2)_{\underline{2}})$), 13.7 ($CH_2CH_2CH_2\underline{C}H_3$); **HRMS** (ES+) exact mass calculated for $[M+ Na]^+$ ($C_{23}H_{30}O_6Na^+$) requires *m/z* 425.1935, found *m/z* 425.1928.

Synthesis and characterisation of 20a

2-benzyl 1,1-di-tert-butyl 3-butylcycloprop-2-ene-1,1,2-tricarboxylate (20a)

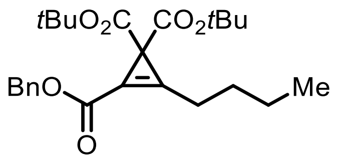

Compound **20a** was synthesised according to general procedure **V** by the addition of **S23** (680 mg, 2.38 mmol, 1.00 eq) to benzyl chloroformate (1.2 mL, 3.57 mmol, 1.50 eq, 3 M in toluene). The crude product was purified by silica gel column chromatography (pent/EtOAc = 50/1 to 25/1) to provide the title compound (**20a**) as a colourless oil in 25% yield (250 mg). **^{1}H NMR** (400 MHz, $CDCl_3$) δ(ppm): 7.39 – 7.29 (m, 5H, Ar$\underline{H}$), 5.27 (s, 2H, C$\underline{H_2}$Ph), 2.69 (t, *J* = 7.2 Hz, 2H, C$\underline{H_2}$$CH_2CH_2CH_3$)), 1.71 – 1.61 (m, 2H, CH_2C$\underline{H_2}$$CH_2CH_3$), 1.43 (s, 18H, C($CO_2$C(C$\underline{H_3}$$\underline{)_3}$$\underline{)_2}$), 1.48 – 1.35 (m, 2H, CH_2CH_2C$\underline{H_2}$$CH_3$), 0.90 (t, *J* = 7.3 Hz, 3H, $CH_2CH_2CH_2$C$\underline{H_3}$); **^{13}C NMR** (101 MHz, $CDCl_3$) δ(ppm): 168.9 (C($\underline{C}$$O_2C(CH_3)_3)_2$), 157.8 ($\underline{C}$$O_2$Bn), 135.5 (Ar$\underline{C}$), 128.6 (Ar$\underline{C}$H), 128.4 (Ar$\underline{C}$H), 128.1 (Ar$\underline{C}$H), 126.4 ($\underline{C}$=CC(=O)), 99.5 (C=$\underline{C}$C(=O)), 81.5 (C($CO_2$$\underline{C}$$(CH_3)_3)_2$), 67.1 ($\underline{C}$$H_2$Ph), 39.0 ($\underline{C}$$(CO_2CH_3)_2$), 28.5 ($CH_2$$\underline{C}$$H_2CH_2CH_3$), 28.1 (C($CO_2$C($\underline{C}$$H_3)_3)_2$), 24.9 ($\underline{C}$$H_2CH_2CH_2CH_3$), 22.3 ($CH_2CH_2$$\underline{C}$$H_2CH_3$), 13.7 ($CH_2CH_2CH_2$$\underline{C}$$H_3$); **HRMS** (ES+) exact mass calculated for $[M+ K]^+$ ($C_{25}H_{34}O_6K^+$) requires *m/z* 469.1987, found *m/z* 469.1979.

Synthesis and characterisation of 21a

tribenzyl 3-butylcycloprop-2-ene-1,1,2-tricarboxylate (21a)

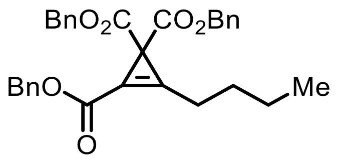

Compound **21a** was synthesised according to general procedure **V** by the addition of **S24** (270 mg, 0.74 mmol, 1.00 eq) to benzyl chloroformate (0.37 mL, 1.11 mmol, 1.50 eq, 3 M in toluene). The crude product was purified by silica gel column chromatography (pent/EtOAc = 5/1) to provide the title compound (**21a**) as a colourless oil in 36% yield (132 mg). **^{1}H NMR** (400 MHz, $CDCl_3$) δ(ppm): 7.35 – 7.32 (m, 5H, Ar$\underline{H}$), 7.32 – 7.26 (m, 10H, Ar$\underline{H}$), 5.26 (s, 2H, $C\underline{H}_2$Ph), 5.17 (d, *J* = 1.4 Hz, 4H, $C(CO_2C\underline{H}_2Ph)_2$), 2.66 (t, *J* = 7.3 Hz, 2H, $C\underline{H}_2CH_2CH_2CH_3$), 1.64 – 1.53 (m, 2H, 2H, $CH_2C\underline{H}_2CH_2CH_3$), 1.39 – 1.29 (m, 2H, $CH_2CH_2C\underline{H}_2CH_3$), 0.83 (t, *J* = 7.3 Hz, 3H, $CH_2CH_2CH_2C\underline{H}_3$); **^{13}C NMR** (101 MHz, $CDCl_3$) δ(ppm): 168.9 ($\underline{C}O_2$Bn), 157.2 ($C(CO_2C\underline{H}_2Ph)_2$), 135.8 (Ar$\underline{C}$), 135.2 (Ar$\underline{C}$), 128.7 (Ar$\underline{C}$H), 128.6 (Ar$\underline{C}$H), 128.5 (Ar$\underline{C}$H), 128.2 (Ar$\underline{C}$H), 128.2 (Ar$\underline{C}$H), 128.0 (Ar$\underline{C}$H), 125.4 ($\underline{C}$=CC(=O)), 98.5 (C=$\underline{C}$C(=O)), 67.5 ($\underline{C}H_2$Ph), 67.1 ($C(CO_2\underline{C}H_2Ph)_2$), 36.8 ($\underline{C}(CO_2CH_3)_2$), 28.3 ($\underline{C}H_2CH_2CH_2CH_3$), 25.0 ($CH_2\underline{C}H_2CH_2CH_3$), 22.2 ($CH_2CH_2\underline{C}H_2CH_3$), 13.6 ($CH_2CH_2CH_2\underline{C}H_3$); **HRMS** (ES+) exact mass calculated for $[M+H]^+$ ($C_{31}H_{31}O_6^+$) requires *m/z* 499.2115, found *m/z* 499.2111.

Synthesis and characterisation of S25

1,1,2-tribromo-2,3,3-trimethylcyclopropane (S25)

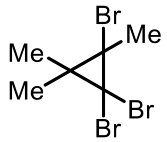

Compound **S25** was synthesised according to general procedure **VI** by addition of bromoform (11.3 ml, 129 mmol, 3.00 eq) to 2-bromo-3-methylbut-2-ene (5.00 ml, 43.1 mmol, 1.00 eq). The reaction was stirred at room temperature overnight. H_2O (50 ml) was added, the layers were separated, and the aqueous phase extracted with CH_2Cl_2 (2 x 50 ml). The combined organic layers were washed with brine, dried over Na_2SO_4, filtered and concentrated *in vacuo*. The crude product was purified by silica gel column chromatography (hexane) to provide the title compound (**S25**) as a white solid in 19% yield (2.64 g). **^{1}H NMR** (400 MHz, $CDCl_3$) δ(ppm): 2.00 (s, 3H, $C(Br)(C\underline{H_3})$), 1.51 (s, 3H, one of $C(C\underline{H_3})_A(CH_3)_B$), 1.37 (s, 3H, one of $C(CH_3)_A(C\underline{H_3})_B$); **^{13}C NMR** (101 MHz, $CDCl_3$) δ(ppm): 51.8 ($\underline{C}Br_2$ or $\underline{C}(Br)(CH_3)$), 51.2 ($\underline{C}Br_2$ or $\underline{C}(Br)(CH_3)$), 33.4 ($\underline{C}(CH_3)_2$), 27.2 (one of $C(\underline{C}H_3)_A(CH_3)_B$), 26.8 ($C(Br)(\underline{C}H_3)$), 21.0 (one of $C(CH_3)_A(\underline{C}H_3)_B$); **IR** (powder) ν_{max}/cm^{-1}: 2991, 2971, 2930, 2889 (C-H), 2161, 2016. Data are consistent with that published in the literature.[16]

Synthesis and characterisation of 22a

benzyl 2,3,3-trimethylcycloprop-1-ene-1-carboxylate (22a)

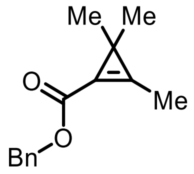

Compound **22a** was synthesised according to general procedure **VII** by the addition of **S25** (300 mg, 0.940 mmol, 1.00 eq) to benzyl chloroformate (0.200 ml, 1.40 mmol, 1.50 eq). The crude product was purified by silica gel column chromatography (pentane/Et_2O = 99/1) to provide the title compound (**22a**) as a colourless oil in 51% yield (104 mg). **^{1}H NMR** (500 MHz, $CDCl_3$) δ(ppm): 7.41 – 7.30 (m, 5H, Ar<u>H</u>), 5.23 (s, 2H, $C\underline{H_2}Ph$), 2.24 (s, 3H, $C\underline{H_3}C{=}C$), 1.22 (s, 6H, $C(C\underline{H_3})_2$); **^{13}C NMR** (126 MHz, $CDCl_3$) δ(ppm)162.3 (<u>C</u>=O), 145.7 ($CH_3\underline{C}{=}C$), 136.3 (Ar<u>C</u>), 128.7 (Ar<u>C</u>H), 128.2 (Ar<u>C</u>H), 128.2 (Ar<u>C</u>H), 117.6 ($CH_3C{=}\underline{C}$), 66.3 ($\underline{C}H_2Ph$), 25.3 ($C(\underline{C}H_3)_2$), 24.8 ($\underline{C}(CH_3)_2$), 11.1 ($\underline{C}H_3C{=}C$); **IR** (film) v_{max}/cm^{-1}: 3382, 3324 (C-H), 1753 (C=O); **HRMS** (ES+) exact mass calculated for $[M+H]^+$ ($C_{14}H_{17}O_2^+$) requires *m/z* 217.1223, found *m/z* 217.1223.

Synthesis and characterisation of 23a

2-benzyl 1,1-dimethyl 3-(3-oxo-3-phenylpropyl)cycloprop-2-ene-1,1,2-tricarboxylate (23a)

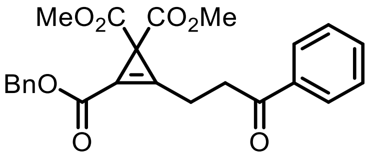

According to a literature procedure,[17] compound **14a** (408 mg, 1.0 mmol, 1.0 equiv.) was added to a stirred solution of $Co(ClO_4)_2$ (36.6 mg, 0.1 mmol, 10 mol%), Oxone (1.84 g, 6.0 mmol, 6.0 equiv.) in CH_3CN (10 ml) and water (10 ml). The reaction mixture was stirred at room temperature for 3 h. The solution was diluted with water and extracted with CH_2Cl_2 (3 x 30 ml), the combined organic phases were dried over Na_2SO_4 and concentrated in vacuo to afford the crude product, which was purified by silica gel column chromatography (pentane/EtOAc = 10/1 to 5/1) to provide the title compound (**23a**) as a colourless oil in 8% yield (34 mg). **^{1}H NMR** (500 MHz, $CDCl_3$) δ(ppm): 7.92 – 7.89 (m, 2H, ArH), 7.58 (t, J = 7.4 Hz, 1H, ArH), 7.45 (t, J = 7.7 Hz, 2H, ArH), 7.38 – 7.31 (m, 5H, ArH of Bn), 5.24 (s, 2H, $PhCH_2$), 3.71 (s, 6H, $C(CO_2CH_3)_2$), 3.44 (t, J = 7.0 Hz, 2H, $C{=}CCH_2CH_2$), 3.14 (t, J = 7.0 Hz, 2H, $C{=}CCH_2CH_2$); **^{13}C NMR** (126 MHz, $CDCl_3$) δ(ppm): 197.0 (PhC(=O)), 169.3 (C(CO2CH$_3$)$_2$), 157.1 (CO_2Bn), 136.3 (ArC of PhC(=O)), 135.1 (ArC of Bn), 133.5 (ArCH), 128.8 (ArCH), 128.8 (ArCH), 128.6 (ArCH), 128.3 (ArCH), 128.1 (ArCH), 124.8 (C=CC(=O)), 99.6 (C=CC(=O)), 67.7 (CH_2Ph), 52.7 ($C(CO_2CH_3)_2$), 36.9 ($C(CO_2CH_3)_2$), 35.4 ($C{=}CCH_2CH_2$), 19.7 ($C{=}CCH_2CH_2$); **HRMS** (ES+) exact mass calculated for $[M+H]^+$ (C24H23O7) requires m/z 423.1438, found m/z 423.1450.

Synthesis and characterisation of 24a

trimethyl 3-ethylcycloprop-2-ene-1,1,2-tricarboxylate (24a)

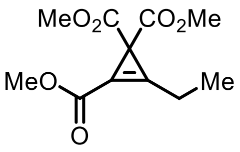

Compound **24a** was synthesised according to general procedure **V** by the addition of **S18** (124 mg, 0.67 mmol, 1.00 eq) to methyl chloroformate (78 μL, 1.01 mmol, 1.50 eq). The crude product was purified by silica gel column chromatography (pent/EtOAc = 5/1) to provide the title compound (**24a**) as a colourless oil in 24% yield (39 mg). **^{1}H NMR** (400 MHz, $CDCl_3$) δ(ppm): 3.84 (s, 3H, $C{=}CCO_2C\underline{H_3}$), 3.73 (s, 6H, $C(CO_2C\underline{H_3})$), 2.72 (q, J = 7.5 Hz, 2H, $C\underline{H_2}CH_3$), 1.28 (t, J = 7.5 Hz, 3H, $CH_2C\underline{H_3}$); **^{13}C NMR** (126 MHz, $CDCl_3$) δ(ppm): 169.6 ($C(CO_2C\underline{H_3})_2$), 157.8 ($C{=}C\underline{C}({=}O)$), 126.0 ($\underline{C}{=}CC({=}O)$), 98.3 ($C{=}\underline{C}C({=}O)$), 52.9 ($C{=}CCO_2\underline{C}H_3$), 52.6 ($C(CO_2\underline{C}H_3)_2$), 36.4 ($\underline{C}(CO_2CH_3)_2$), 18.9 ($C\underline{H_2}CH_3$), 10.9 ($CH_2C\underline{H_3}$); **HRMS** (ES+) exact mass calculated for $[M+K]^+$ ($C_{11}H_{14}O_6K^+$) requires *m/z* 281.0422, found *m/z* 281.0412.

Synthesis and characterisation of S26

1,1,2-tribromo-2-propylspiro[2.5]octane (S26)

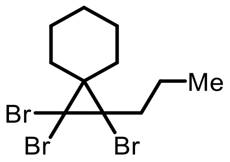

i. According to a literature procedure,[18] under argon at 0 °C, to a solution of butyltriphenylphosphonium bromide (9.6 g, 24 mmol, 1.20 equiv) in THF (25 ml) was added ^{t}BuOK (2.7 g, 24 mmol, 1.20 equiv.). The reaction was allowed to warm to rt and stirred for 30 min, and then re-cooled to 0 °C, a solution of cyclohexanone (2.4 mL, 20 mmol, 1.00 equiv.) in anhydrous THF (45 ml) was added dropwise. The reaction was then warmed to room temperature and stirred for 12 h. The reaction was quenched by addition of NH_4Cl (sat. aq.), extracted with Et_2O (3 x 60 ml), the combined organic phased were dried over Na_2SO_4 and concentrated. The residue was purified by silica gel flash chromatography to give the product as a colourless oil (0.93 g, 34% yield).

ii. According to a literature procedure,[19] the product from step i (1.0 g, 7.24 mmol, 1.00 equiv) was dissolved in CH_2Cl_2 (20 ml) in an oven-dried round-bottomed flask and the resulting solution cooled to 0 °C under argon. A solution of Br_2 (0.4 ml, 7.8 mmol, 1.08 equiv.) in hexane (3.3 ml) was added dropwise to the reaction via syringe over 1 min. The mixture was stirred at 0 °C for 5 min and then warmed to room temperature. After stirring for 30 min, the reaction mixture was concentrated under a stream of N_2. The crude reaction mixture was dissolved in THF (16 ml) and the solution was cooled to -78 ^{o}C under argon. KHMDS (22 mL, 1 M in THF, 3.00 equiv.) was added dropwise and the mixture was stirred overnight, during which the cooling bath was warmed room temperature over 14 h. The reaction was quenched with sat. aq. NH_4Cl (20 ml) and extracted with hexane (3 x 40 ml). The combined organic phase was washed with saturated aqueous $NaHCO_3$ (20 ml), brine, dried over Na_2SO_4, filtered and concentrated

in vacuo to give a brown oil, which was then filtered by a silica gel plug with pentane as eluent to provide the crude product as colourless oil (950 mg). The crude was directly used for next step without further purification.

iii. According to general procedure **VI**, bromoform (1.13 ml, 13.2 mmol, 3.00 eq) was added to the product from step ii (0.95 g). The reaction mixture was stirred at room temperature for 24 h, at which point H_2O (20 ml) was added. The layers were separated and the aqueous phase extracted with CH_2Cl_2 (3 x 10 ml). The combined organic layers were washed with brine, dried over Na_2SO_4, filtered and concentrated *in vacuo*. The crude product was purified by silica gel column chromatography using pentane as eluent to provide the title compound **S26** as a colourless oil (1.16 g, 41% over 2 steps). **^{1}H NMR** (400 MHz, $CDCl_3$) δ(ppm): 2.03 – 1.94 (m, 2H, $C\underline{H_2}CH_2CH_3$), 1.79 – 1.67 (m, 10H, cyclohexyl), 1.65 – 1.56 (m, 2H, $CH_2C\underline{H_2}CH_3$), 0.97 (t, *J* = 7.4 Hz, 3H, $CH_2CH_2C\underline{H_3}$); **^{13}C NMR** (126 MHz, $CDCl_3$) δ(ppm): 58.3 ($\underline{C}Br_2$), 50.8 ($\underline{C}(Br)(CH_2)$), 39.7 (one of $(\underline{C}H_2)_ACH_2CH_2CH_2(CH_2)_B$), 37.6 ($\underline{C}(CH_2)_2$), 37.1 (one of $(CH_2)_ACH_2CH_2CH_2(\underline{C}H_2)_B$), 31.6 ($C(Br)(\underline{C}H_2)$), 25.5 (one of $CH_2(\underline{C}H_2)_ACH_2(CH_2)_BCH_2$), 24.8 (one of $CH_2(CH_2)_ACH_2(\underline{C}H_2)_BCH_2$ and $CH_2(CH_2)_A\underline{C}H_2(CH_2)_BCH_2$), 21.4 ($\underline{C}H_2CH_3$), 13.9 ($\underline{C}H_3$).

Synthesis and characterisation of 38a

benzyl 2-propylspiro[2.5]oct-1-ene-1-carboxylate (38a)

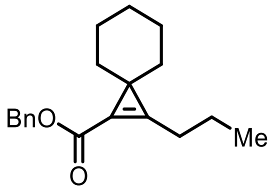

Compound **38a** was synthesised according to general procedure **VII** by the addition of **S26** (1.16 g, 2.98 mmol, 1.00 eq) to benzyl chloroformate (3 M in toluene, 1.5 mL, 4.47 mmol, 1.50 eq). The crude product was purified by silica gel column chromatography (pentane/EtOAc = 50/1) to provide the title compound (**38a**) as a colourless oil in 86% yield (811 mg). **^{1}H NMR** (400 MHz, $CDCl_3$) δ(ppm)7.47 – 7.28 (m, 5H, Ar$\underline{H}$), 5.23 (s, 2H, PhC$\underline{H_2}$), 2.61 (t, *J* = 7.2 Hz, 2H, C=CC$\underline{H_2}$), 1.79 – 1.62 (m, 4H, $C\underline{H_2}CH_2CH_2CH_2C\underline{H_2}$), 1.59 – 1.41 (m, 8H, $CH_2C\underline{H_2}C\underline{H_2}C\underline{H_2}CH_2$ and $C\underline{H_2}CH_3$), 0.99 (t, *J* = 7.4 Hz, 3H, $CH_2C\underline{H_3}$); **^{13}C NMR** (101 MHz, $CDCl_3$) δ(ppm)162.3 ($\underline{C}O_2Bn$), 150.2 ($\underline{C}$=CC(=O)), 136.4 (Ar$\underline{C}$), 128.6 (Ar$\underline{C}$H), 128.1 (Ar$\underline{C}$H), 128.0 (Ar$\underline{C}$H), 117.4 (C=$\underline{C}$C(=O)), 66.2 ($\underline{C}H_2Ph$), 37.5 ($C(\underline{C}H_2CH_2CH_2CH_2\underline{C}H_2)$), 32.5 ($\underline{C}(CH_2CH_2CH_2CH_2CH_2)$), 29.4 ($\underline{C}H_2CH_2CH_3$), 27.1 ($C(CH_2\underline{C}H_2CH_2\underline{C}H_2CH_2)$), 26.7 ($C(CH_2CH_2\underline{C}H_2CH_2CH_2)$), 20.7($CH_2\underline{C}H_2CH_3$), 14.1 ($CH_2CH_2\underline{C}H_3$); **HRMS** (ES+) exact mass calculated for $[M+ Na]^+$ ($C_{19}H_{24}O_2Na^+$) requires *m/z* 307.1669, found *m/z* 307.1654.

5.3 Synthesis of amide substrates

Synthesis and characterisation of 25a

dimethyl 2-butyl-3-(tert-butylcarbamoyl)cycloprop-2-ene-1,1-dicarboxylate (25a)

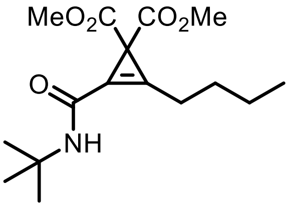

Compound **25a** was synthesised according to general procedure **V** by the addition of **S10** (500 mg, 2.40 mmol, 1.00 eq) to *tert*-butyl isocyanate (0.410 ml, 3.60 mmol, 1.50 eq). The crude product was purified by silica gel column chromatography (1st column: pentane/EtOAc = 8/2, 2nd column: pentane/EtOAc = 85/15) to provide the title compound (**25a**) as an amorphous yellow solid in 50% yield (377 mg). **^{1}H NMR** (400 MHz, $CDCl_3$) δ(ppm): 6.28 (s, 1H, $N\underline{H}$), 3.72 (s, 6H, $C(CO_2C\underline{H_3})_2$), 2.60 (t, J = 7.5 Hz, 2H, $C\underline{H_2}CH_2CH_2CH_3$), 1.68 – 1.56 (m, 2H, $CH_2C\underline{H_2}CH_2CH_3$), 1.44 – 1.37 (m, 2H, $CH_2CH_2C\underline{H_2}CH_3$), 1.36 (s, 9H, $C(C\underline{H_3})_3$), 0.89 (t, J = 7.5 Hz, 3H, $CH_2CH_2CH_2C\underline{H_3}$); **^{13}C NMR** (101 MHz, $CDCl_3$) δ(ppm): 170.6 ($C(\underline{C}O_2CH_3)_2$), 155.9 ($\underline{C}$(=O)NH), 119.2 ($\underline{C}$=CC(=O)N), 101.6 (C=$\underline{C}$C(=O)N), 52.6 ($C(CO_2\underline{C}H_3)_2$), 52.1 ($N\underline{C}(CH_3)_3$), 37.3 ($\underline{C}(CO_2CH_3)_2$), 28.7 ($NC(\underline{C}H_3)_3$), 28.5 ($CH_2\underline{C}H_2CH_2CH_3$), 24.5 ($\underline{C}H_2CH_2CH_2CH_3$), 22.2 ($CH_2CH_2\underline{C}H_2CH_3$), 13.7 ($CH_2CH_2CH_2\underline{C}H_3$); **IR** (film) ν_{max}/cm^{-1}: 3237 (N-H), 2962, 2875 (C-H), 2210, 1732 (C=O ester), 1651 (C=O amide); **HRMS** (ES+) exact mass calculated for $[M+Na]^+$ ($C_{16}H_{25}NaO_5^+$) requires *m/z* 334.1625, found *m/z* 334.1624.

Synthesis and characterisation of 26a

dimethyl 2-(allylcarbamoyl)-3-butylcycloprop-2-ene-1,1-dicarboxylate (26a)

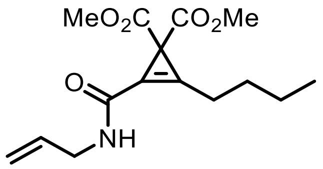

Compound **26a** was synthesised according to general procedure **V** by the addition of **S10** (250 mg, 1.18 mmol, 1.00 eq) to allyl isocyanate (0.156 ml, 1.77 mmol, 1.50 eq). The crude product was purified by silica gel column chromatography (pentane/Et_2O = 1/1) to provide the title compound (**26a**) as a yellow oil in 10% yield (37.2 mg). **^{1}H NMR** (500 MHz, $CDCl_3$) δ(ppm): 6.50 (bs, 1H, NH), 5.85 (ddt, *J* = 17.0, 10.5, 5.5 Hz, 1H, CH=CH_2), 5.24 – 5.19 (m, one of C=CH_AH_B), 5.17 (app-dq, *J* = 10.5, 1.5 Hz, 1H, one of C=CH_AH_B), 3.97 (tt, *J* = 5.5, 1.5 Hz, 2H, $NHCH_2$), 3.75 (s, 6H, $C(CO_2CH_3)_2$), 2.67 (t, *J* = 7.5 Hz, 2H, $CH_2CH_2CH_2CH_3$), 1.67 (tt, *J* = 7.5, 6.5 Hz, 2H, $CH_2CH_2CH_2CH_3$), 1.47 – 1.36 (m, 2H, $CH_2CH_2CH_2CH_3$), 0.93 (t, *J* = 7.5 Hz, 3H, $CH_2CH_2CH_2CH_3$); **^{13}C NMR** (126 MHz, $CDCl_3$) δ(ppm): 170.5 ($C(CO_2Me)_2$), 156.5 (C(=O)NH), 133.3 (CH=CH_2), 120.6 (C=CC(=O)), 117.0 (CH=CH_2), 100.6 (C=CC(=O)), 52.8 ($C(CO_2CH_3)_2$), 42.0 (CH_2NH), 37.0 ($C(CO_2CH_3)_2$), 28.5 ($CH_2CH_2CH_2CH_3$), 24.7 ($CH_2CH_2CH_2CH_3$), 22.3 ($CH_2CH_2CH_2CH_3$), 13.7 ($CH_2CH_2CH_2CH_3$); **IR** (film) ν_{max}/cm^{-1}: 3342 (N-H), 2957, 2874 (C-H), 1729, 1657 (C=O); **HRMS** (ES+) exact mass calculated for $[M+H]^+$ ($C_{15}H_{22}NO_5^+$) requires *m/z* 296.1492, found *m/z* 296.1494.

Synthesis and characterisation of 27a

dimethyl 2-(tert-butylcarbamoyl)-3-cyclopentylcycloprop-2-ene-1,1-dicarboxylate (27a)

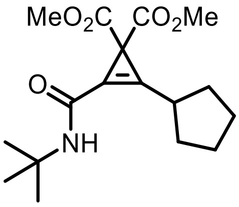

Compound **27a** was synthesised according to general procedure **V** by the addition of **S11** (250 mg, 1.11 mmol, 1.00 eq) to *tert*-butyl isocyanate (0.190 ml, 1.67 mmol, 1.50 eq). The crude product was purified by silica gel column chromatography (pentane/Et_2O = 8/2 to 7/3) to provide the title compound (**27a**) as a yellow solid in 74% yield (266 mg). **¹H NMR** (500 MHz, $CDCl_3$) δ(ppm): 6.26 (s, 1H, N$\underline{H}$), 3.74 (s, 6H, $C(CO_2C\underline{H_3})_2$), 3.13 (tt, *J* = 8.0, 6.5 Hz, 1H, C$\underline{H}$C=C), 2.01 – 1.89 (m, 2H, $C\underline{H_A}H_BCHC\underline{H_C}H_D$), 1.82 – 1.67 (m, 3H, $C\underline{H_A}H_BCH_2CHCH_2C\underline{H_C}H_D$ and $CH_A\underline{H_B}CHCH_CH_D$), 1.67 – 1.54 (m, 3H, $CH_A\underline{H_B}CH_2CHCH_2CH_C\underline{H_D}$ and $CH_AH_BCHC\underline{H_C}H_D$), 1.39 (s, 9H, $C(C\underline{H_3})_3$); **¹³C NMR** (126 MHz, $CDCl_3$) δ(ppm): 170.7 ($\underline{C}O_2Me$), 156.0 ($\underline{C}$ONH), 122.9 (CH$\underline{C}$=C), 100.5 (CHC=$\underline{C}$), 52.7 ($C(CO_2\underline{C}H_3)_2$), 52.2 ($N\underline{C}(CH_3)_3$), 37.7 ($\underline{C}(CO_2CH_3)_2$), 35.8 ($\underline{C}$HC=C), 31.2 ($CH_2\underline{C}H_2CH\underline{C}H_2CH_2$), 28.8 ($NC(\underline{C}H_3)_3$), 25.4 ($\underline{C}H_2CH_2CHCH_2\underline{C}H_2$); **IR** (film) ν_{max}/cm^{-1}: 1736 (C=O ester), 1667 (C=O amide); **HRMS** (ES+) exact mass calculated for $[M+Na]^+$ ($C_{17}H_{25}NNaO_5^+$) requires *m/z* 346.1625, found *m/z* 346.1624.

Synthesis and characterisation of 28a

N-(tert-butyl)-2,3,3-trimethylcycloprop-1-ene-1-carboxamide (28a)

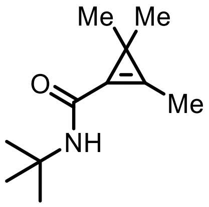

Compound **28a** was synthesised according to general procedure **VII** by the addition of tribromocyclopropane **S12** (300 mg, 0.940 mmol, 1.00 eq) to *tert*-butyl isocyanate (0.160 ml, 1.41 mmol, 1.50 eq). The crude product was purified by silica gel column chromatography (pentane/EtOAc = 7/3) to provide the title compound (**28a**) as a white solid in 34% yield (57.5 mg). **^{1}H NMR** (500 MHz, $CDCl_3$) δ(ppm): 5.34 (bs, 1H, NH), 2.18 (s, 3H, CH_3C=C), 1.40 (s, 9H, $C(CH_3)_3$), 1.20 (s, 6H, $C(CH_3)_2$); **^{13}C NMR** (126 MHz, $CDCl_3$) δ(ppm): 161.0 (C=O), 137.8 (C=CC(=O)), 120.2 (C=CC(=O)), 51.6 ($C(CH_3)_3$), 29.1 ($C(CH_3)$), 25.1 ($C(CH_3)_2$), 23.7 ($C(CH_3)_2$), 10.4 (CH_3C=C); **IR** (film) ν_{max}/cm^{-1}: 3222, 3046 (C-H), 1624 (C=O); **HRMS** (ES+) exact mass calculated for $[M+H]^+$ ($C_{11}H_{20}NO^+$) requires *m/z* 182.1539, found *m/z* 182.1541; **MP**: 92 °C (sublimation).

Synthesis and characterisation of 29a

dimethyl 2-butyl-3-((4-methoxyphenyl)carbamoyl)cycloprop-2-ene-1,1-dicarboxylate (29a)

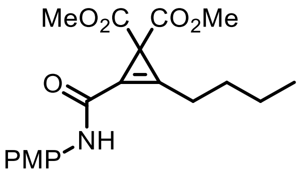

Compound **29a** was synthesised according to general procedure **V** by the addition of **S10** (250 mg, 1.18 mmol, 1.00 eq) to 4-Methoxyphenyl isocyanate (0.230 ml, 1.77 mmol, 1.50 eq). The crude product was purified by silica gel column chromatography (pentane/EtOAc = 7/3) and the isolated product was washed with Et_2O to provide the title compound (**29a**) as a white solid in 48% yield (206 mg). **^{1}H NMR** (400 MHz, $CDCl_3$) δ(ppm): 8.31 (s, 1H, $N\underline{H}$), 7.55 – 7.46 (m, 2H, $Ar\underline{H}$), 6.92 – 6.84 (m, 2H, $Ar\underline{H}$), 3.80 (s, 3H, $ArOC\underline{H_3}$), 3.78 (s, 6H, $C(CO_2C\underline{H_3})_2$), 2.70 (t, J = 7.5 Hz, 2H, $C\underline{H_2}CH_2CH_2CH_3$), 1.70 (tt, J = 7.5, 6.5 Hz, 2H, $CH_2C\underline{H_2}CH_2CH_3$), 1.51 – 1.38 (m, 2H, $CH_2CH_2C\underline{H_2}CH_3$), 0.94 (t, J = 7.5 Hz, 3H, $CH_2CH_2CH_2C\underline{H_3}$); **^{13}C NMR** (126 MHz, $CDCl_3$) δ(ppm): 170.7 ($C(\underline{C}O_2Me)_2$), 157.0 ($Ar\underline{C}$), 154.4 ($\underline{C}$(=O)NH), 130.4 ($Ar\underline{C}$), 121.5 ($Ar\underline{C}H$), 121.1 ($\underline{C}$=CC(=O)NH), 114.4 ($Ar\underline{C}H$), 101.4 (C=$\underline{C}$C(=O)NH), 55.6 ($ArO\underline{C}H_3$), 52.9 ($C(CO_2\underline{C}H_3)_2$), 37.5 ($\underline{C}(CO_2CH_3)_2$), 28.5 ($CH_2\underline{C}H_2CH_2CH_3$), 24.8 ($\underline{C}H_2CH_2CH_2CH_3$), 22.3 ($CH_2CH_2\underline{C}H_2CH_3$), 13.7 ($CH_2CH_2CH_2\underline{C}H_3$); **IR** (film) v_{max}/cm^{-1}: 3314 (N-H), 2981, 2886 (C-H), 1745 (C=O ester), 1653 (C=O amide); **HRMS** (ES+) exact mass calculated for $[M+Na]^+$ ($C_{19}H_{23}NNaO_6^+$) requires *m/z* 384.1418, found *m/z* 384.1416; **MP**: 116 – 118 °C.

Synthesis and characterisation of 30a

dimethyl 2-butyl-3-(diphenylphosphoryl)cycloprop-2-ene-1,1-dicarboxylate (30a)

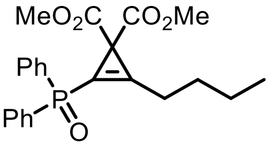

Compound **30a** was synthesised according to general procedure **V** by the addition of **S10** (250 mg, 1.18 mmol, 1.00 eq) to chlorodiphenylphosphine (196 µl, 1.06 mmol, 0.900 eq). The crude product was purified by silica gel column chromatography (pentane/EtOAc = 6/4) to provide the title compound (**30a**) as a yellow solid in 33% yield (161 mg). **^{1}H NMR** (600 MHz, $CDCl_3$) δ(ppm): 7.84 – 7.75 (m, 4H, Ar<u>H</u>), 7.59 – 7.54 (m, 2H, Ar<u>H</u>), 7.52 – 7.45 (m, 4H, Ar<u>H</u>), 3.52 (s, 6H, $C(CO_2C\underline{H_3})_2$), 2.70 (td, J = 7.5, 1.0 Hz, 2H, $C\underline{H_2}CH_2CH_2CH_3$), 1.56 (p, J = 7.5 Hz, 2H, $CH_2C\underline{H_2}CH_2CH_3$), 1.38 – 1.29 (m, 2H, $CH_2CH_2C\underline{H_2}CH_3$), 0.85 (t, J = 7.5 Hz, 3H, $CH_2CH_2CH_2C\underline{H_3}$); **^{13}C NMR** (151 MHz, $CDCl_3$) δ(ppm): 169.7 (d, J_{CP} = 2.5 Hz, $C(\underline{C}O_2Me)_2$), 132.6 (d, J_{CP} = 3.0 Hz, Ar<u>C</u>H), 132.0 (d, J_{CP} = 10.0 Hz, Ar<u>C</u>), 131.5 (d, J_{CP} = 11.0 Hz, Ar<u>C</u>H), 130.59 (d, J_{CP} = 60.0 Hz, $CH_2\underline{C}$=CP(=O)), 128.7 (d, J = 13.0 Hz, Ar<u>C</u>H), 100.3 (d, J_{CP} = 119.5 Hz, CH_2C=<u>C</u>P(=O)), 52.4 ($C(CO_2\underline{C}H_3)_2$), 37.2 ($\underline{C}(CO_2CH_3)_2$), 28.5 ($CH_2\underline{C}H_2CH_2CH_3$), 25.1 ($\underline{C}H_2CH_2CH_2CH_3$), 22.4 ($CH_2CH_2\underline{C}H_2CH_3$), 13.7 ($CH_2CH_2CH_2\underline{C}H_3$); **^{31}P NMR** (162 MHz, $CDCl_3$) δ(ppm): 15.6 (<u>P</u>=O); **IR** (film) ν_{max}/cm^{-1}: 2967, 2953, 2932, 2871 (C-H), 1840, 1742, 1711; **HRMS** (ES+) exact mass calculated for $[M+H]^+$ ($C_{23}H_{26}O_5P^+$) requires *m/z* 413.1512, found *m/z* 413.1516; **MP**: 49-50 °C.

5.4 Synthesis of ketone substrates

Synthesis and characterisation of S27

<u>dimethyl 2-butyl-3-(1-hydroxy-3-phenylpropyl)cycloprop-2-ene-1,1-dicarboxylate (S27)</u>

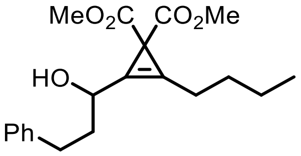

Compound **S27** was synthesised according to general procedure **V** by the addition of **S10** (408 mg, 1.92 mmol, 1.00 eq) to 3-phenylpropanal (0.760 ml, 5.76 mmol, 3.00 eq). The crude product was purified by silica gel column chromatography (pentane/Et_2O = 6/4) to provide the title compound (**S27**) as a colourless oil in 86% yield (570 mg). **^{1}H NMR** (600 MHz, $CDCl_3$) δ(ppm): 7.32 – 7.27 (m, 2H, Ar$\underline{H}$), 7.24 – 7.17 (m, 3H, Ar$\underline{H}$), 4.67 (t, *J* = 6.5 Hz, 1H, $C\underline{H}OH$), 3.73 (s, 3H, one of $C(CO_2C\underline{H_3})_A(CO_2CH_3)_B$), 3.73 (s, 3H, one of $C(CO_2CH_3)_A(CO_2C\underline{H_3})_B$), 2.87 (s, 1H, $O\underline{H}$), 2.75 (bt, *J* = 7.5 Hz, 2H, $C\underline{H_2}Ph$), 2.52 (ddt, *J* = 9.5, 7.5, 1.0 Hz, 2H, $C\underline{H_2}CH_2CH_2CH_3$), 2.10 – 1.95 (m, 2H, $C\underline{H_2}CH_2Ph$), 1.62 – 1.53 (m, 2H, $CH_2C\underline{H_2}CH_2CH_3$), 1.41 – 1.32 (m, 2H, $CH_2CH_2C\underline{H_2}CH_3$), 0.91 (t, *J* = 7.5 Hz, 3H, $CH_2CH_2CH_2C\underline{H_3}$); **^{13}C NMR** (151 MHz, $CDCl_3$) δ(ppm): 172.4 (one of $C(\underline{C}O_2CH_3)_A(CO_2CH_3)_B$), 172.3 (one of $C(CO_2CH_3)_A(\underline{C}O_2CH_3)_B$), 141.4 (Ar$\underline{C}$), 128.8 (Ar$\underline{C}$H), 126.2 (Ar$\underline{C}$H), 108.7 ($\underline{C}$=CCOH), 106.5 (C=$\underline{C}$COH), 65.1 ($\underline{C}$OH), 52.67 – 52.38 (m, $C(CO_2\underline{C}H_3)_2$), 37.3 ($\underline{C}H_2CHOH$), 35.6 ($\underline{C}(CO_2\underline{C}H_3)_2$), 31.4 ($\underline{C}H_2Ph$), 28.8 ($CH_2\underline{C}H_2CH_2CH_3$), 23.6 ($\underline{C}H_2CH_2CH_2CH_3$), 22.4 ($CH_2CH_2\underline{C}H_2CH_3$), 13.8 ($CH_2CH_2CH_2\underline{C}H_3$); **IR** (film) ν_{max}/cm^{-1}: 3468 (O-H), 2954, 2872 (C-H), 1726 (C=O), 1278, 1242, 1063; **HRMS** (ES+) exact mass calculated for $[M+Na]^+$ ($C_{20}H_{26}NaO_5^+$) requires *m/z* 369.1672, found *m/z* 369.1669.

Synthesis and characterisation of 4a

dimethyl 2-butyl-3-(3-phenylpropanoyl)cycloprop-2-ene-1,1-dicarboxylate (4a)

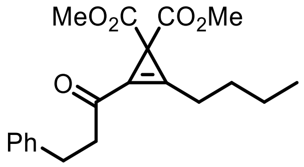

Compound **4a** was synthesised according to general procedure **VIII** by oxidation of compound **S27** (535 mg, 1.54 mmol, 1.00 eq). The crude product was purified by silica gel column chromatography (pentane/Et_2O = 8/2) to provide the title compound (**4a**) as a colourless oil in 89% yield (472 mg). **^{1}H NMR** (400 MHz, $CDCl_3$) δ(ppm): 7.27 – 7.18 (m, 2H, Ar<u>H</u>), 7.18 – 7.07 (m, 3H, Ar<u>H</u>), 3.65 (s, 6H, $C(CO_2C\underline{H_3})_2$), 3.04 – 2.95 (m, 2H, $C\underline{H_2}CH_2Ph$), 2.95 – 2.86 (m, 2H, $CH_2C\underline{H_2}Ph$), 2.61 (t, *J* = 7.5 Hz, 2H, $C\underline{H_2}CH_2CH_2CH_3$), 1.67 – 1.53 (m, 2H, $CH_2C\underline{H_2}CH_2CH_3$), 1.40 – 1.26 (m, 3H, $CH_2CH_2C\underline{H_2}CH_3$), 0.85 (t, *J* = 7.5 Hz, 3H, $CH_2CH_2CH_2C\underline{H_3}$); **^{13}C NMR** (101 MHz, $CDCl_3$) δ(ppm): 188.8 (C=C<u>C</u>(=O)), 169.8 ($C(\underline{C}O_2CH_3)_2$), 140.5 (Ar<u>C</u>), 128.8 (Ar<u>C</u>H), 128.6 (Ar<u>C</u>H), 126.5 (Ar<u>C</u>H), 123.2 (<u>C</u>=CC(=O)), 103.6 (C=<u>C</u>C(=O)), 52.8 ($C(CO_2\underline{C}H_3)_2$), 45.3 ($\underline{C}H_2CH_2Ph$), 36.6 ($\underline{C}(CO_2CH_3)_2$), 29.7 ($CH_2\underline{C}H_2Ph$), 28.6 ($CH_2\underline{C}H_2CH_2CH_3$), 25.2 ($\underline{C}H_2CH_2CH_2CH_3$), 22.5 ($CH_2CH_2\underline{C}H_2CH_3$), 13.8 ($CH_2CH_2CH_2\underline{C}H_3$); **IR** (film) ν_{max}/cm^{-1}: 2956, 2873 (C-H), 1857, 1727, 1686 (C=O); **HRMS** (ES+) exact mass calculated for $[M+H]^+$ ($C_{20}H_{25}O_5^+$) requires *m/z* 345.1697, found *m/z* 345.1695.

Synthesis and characterisation of S28

dimethyl 2-cyclopentyl-3-(1-hydroxy-3-phenylpropyl)cycloprop-2-ene-1,1-dicarboxylate (S28)

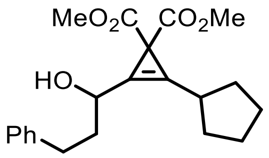

Compound **S28** was synthesised according to general procedure **V** by the addition of **S11** (250 mg, 1.11 mmol, 1.00 eq) to 3-phenylpropanal (0.220 ml, 1.67 mmol, 1.50 eq). The crude product was purified by silica gel column chromatography (pentane/Et_2O = 1:1) to provide the title compound (**S28**) as a colourless oil in 64% yield (254 mg). **^{1}H NMR** (500 MHz, $CDCl_3$) δ(ppm): 7.33 – 7.26 (m, 2H, ArH), 7.22 – 7.15 (m, 3H, ArH), 4.67 (t, *J* = 6.5 Hz, 1H, CHOH), 3.73 (s, 3H, one of $C(CO_2CH_3)_A(CO_2CH_3)_B$), 3.73 (s, 3H, one of $C(CO_2CH_3)_A(CO_2CH_3)_B$), 3.08 – 2.97 (m, 1H, CHC=CCHOH), 2.91 (bs, 1H, OH), 2.75 (t, *J* = 8.0 Hz, 2H, CH_2CH_2Ph), 2.11 – 1.96 (m, 2H, CH_2CH_2Ph), 1.96 – 1.86 (m, 2H, CH_2 - cyclopentane ring), 1.74 – 1.66 (m, 2H, CH_2 - cyclopentane ring), 1.62 (m, 4H, 2 x CH_2 - cyclopentane ring); **^{13}C NMR** (126 MHz, $CDCl_3$) δ(ppm): 172.5 (one of $C(CO_2CH_3)_A(CO_2CH_3)_B$), 172.4 (one of $C(CO_2CH_3)_A(CO_2CH_3)_B$), 141.5 (ArC), 128.6 (ArCH), 128.6 (ArCH), 126.1 (ArCH), 112.1 (CHC=CCHOH), 105.3 (CHC=CCHOH), 65.0 (CHOH), 52.6 (one of $C(CO_2CH_3)_A(CO_2CH_3)_B$), 52.5 (one of $C(CO_2CH_3)_A(CO_2CH_3)_B$), 37.4 ($CH_2CH_2Ph$), 35.9 ($C(CO_2Me)_2$), 35.0 (CHC=CCHOH), 31.4 (CH_2CH_2Ph), 31.2 (CH_2 – cyclopentane ring), 25.2 (CH_2 - cyclopentane ring); **IR** (film) ν_{max}/cm^{-1}: Unlabelled broad peak around 3400 (O-H), 1731 (C=O); **HRMS** (ES+) exact mass calculated for $[M+Na]^+$ ($C_{21}H_{26}NaO_5^+$) requires *m/z* 381.1672, found *m/z* 381.1671.

Synthesis and characterisation of 31a

dimethyl 2-cyclopentyl-3-(3-phenylpropanoyl)cycloprop-2-ene-1,1-dicarboxylate (31a)

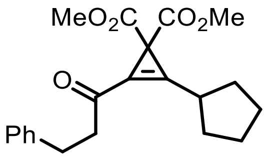

Compound **31a** was synthesised according to general procedure **VIII** by oxidation of compound **S28** (239 mg, 0.670 mmol, 1.00 eq). The crude product was purified by silica gel column chromatography (pentane/Et_2O = 7/3) to provide the title compound (**31a**) as a colourless oil in 75% yield (178 mg). **^{1}H NMR** (500 MHz, $CDCl_3$) δ(ppm): 7.33 – 7.27 (m, 2H, Ar$\underline{H}$), 7.20 (tt, *J* = 8.0, 1.5 Hz, 3H, Ar$\underline{H}$), 3.72 (s, 6H, $C(CO_2C\underline{H_3})_2$), 3.23 – 3.13 (m, 1H, C$\underline{H}$C=CC(=O)), 3.05 (ddd, *J* = 8.5, 6.5, 1.5 Hz, 2H, $C\underline{H_2}CH_2Ph$), 2.98 (ddd, *J* = 8.5, 6.5, 1.5 Hz, 2H, $CH_2C\underline{H_2}Ph$), 2.03 – 1.93 (m, 2H two of $C\underline{H_A}H_BCH_2CH_2C\underline{H_C}H_D$), 1.78 – 1.60 (m, 6H, two of $CH_A\underline{H_B}CH_2CH_2CH_C\underline{H_D}$ and $CH_2C\underline{H_2}C\underline{H_2}CH_2$); **^{13}C NMR** (126 MHz, $CDCl_3$) δ(ppm): 188.7 (C=C$\underline{C}$(=O)), 169.8 ($C(\underline{C}O_2Me)_2$), 140.5 (Ar$\underline{C}$), 128.7 (Ar$\underline{C}$H), 128.5 (Ar$\underline{C}$H), 126.4 ($\underline{C}$=CC(=O)), 126.3 (Ar$\underline{C}$H), 102.2 (C=$\underline{C}$C(=O)), 52.7 ($C(CO_2\underline{C}H_3)_2$), 45.2 ($\underline{C}H_2CH_2Ph$), 36.7 ($\underline{C}(CO_2CH_3)_2$), 36.3 ($\underline{C}$HC=CC(=O)), 31.3 ($C{=}CCH\underline{C}H_2CH_2$), 29.6 ($CH_2\underline{C}H_2Ph$), 25.4 ($C{=}CCHCH_2\underline{C}H_2$); **IR** (film) v_{max}/cm^{-1}: 3028, 2953, 2872 (C-H), 1726 (C=O ester), 1685 (C=O ketone); **HRMS** (ES+) exact mass calculated for $[M+Na]^+$ ($C_{21}H_{24}NaO_5^+$) requires *m/z* 379.1516, found *m/z* 379.1511.

Synthesis and characterisation of S29

dimethyl 2-butyl-3-(hydroxy(4-methoxyphenyl)methyl)cycloprop-2-ene-1,1-dicarboxylate (S29)

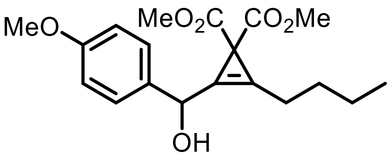

Compound **S29** was synthesised according to general procedure **V** by the addition of **S10** (250 mg, 1.18 mmol, 1.00 eq) to 4-anisaldehyde (0.220 ml, 1.77 mmol, 1.50 eq). The crude product was purified by silica gel column chromatography (pentane/Et_2O = 6/4) to provide the title compound (**S29**) as a colourless oil in 80% yield (329 mg). **^{1}H NMR** (500 MHz, $CDCl_3$) δ(ppm): 7.36 – 7.29 (m, 2H, Ar<u>H</u>), 6.93 – 6.85 (m, 2H, Ar<u>H</u>), 5.65 (d, *J* = 4.5 Hz, 1H, C<u>H</u>OH), 3.80 (s, 3H, ArOC<u>H₃</u>), 3.70 (s, 3H, one of $C(CO_2C\underline{H_3})_A(CO_2CH_3)_B$), 3.62 (s, 3H, one of $C(CO_2CH_3)_A(CO_2C\underline{H_3})_B$), 3.43 (d, *J* = 5.0 Hz, 1H, O<u>H</u>), 2.44 (app-tt, *J* = 7.0, 1.0 Hz, 2H, $C\underline{H_2}C=C$), 1.44 (tt, *J* = 7.5, 6.5 Hz, 2H, $CH_2C\underline{H_2}CH_2CH_3$), 1.32 – 1.22 (m, 2H, $CH_2CH_2C\underline{H_2}CH_3$), 0.84 (t, *J* = 7.5 Hz, 3H, $CH_2CH_2CH_2C\underline{H_3}$); **^{13}C NMR** (126 MHz, $CDCl_3$) δ(ppm): 172.2 (one of $C(\underline{C}O_2Me)_A(CO_2Me)_B$), 172.2 (one of $C(CO_2Me)_A(\underline{C}O_2Me)_B$), 159.7 (Ar<u>C</u>), 132.2 (Ar<u>C</u>), 127.8 (Ar<u>C</u>H), 114.0 (Ar<u>C</u>H), 108.7 ($CH_2\underline{C}=C$), 106.7 ($CH_2C=\underline{C}$), 67.4 (<u>C</u>HOH), 55.5 ($ArO\underline{C}H_3$), 52.6 (one of $C(CO_2\underline{C}H_3)_A(CO_2CH_3)_B$), 52.4 (one of $C(CO_2CH_3)_A(CO_2\underline{C}H_3)_B$), 36.2 ($\underline{C}(CO_2Me)_2$), 28.6 ($CH_2\underline{C}H_2CH_2CH_3$), 23.5 ($\underline{C}H_2CH_2CH_2CH_3$), 22.3 ($CH_2CH_2\underline{C}H_2CH_3$), 13.7 ($CH_2CH_2CH_2\underline{C}H_3$); **IR** (film) ν_{max}/cm^{-1}: Unlabelled broad peak around 3500 (O-H), 1731 (C=O); **HRMS** (ES+) exact mass calculated for $[M+Na]^+$ ($C_{19}H_{24}NaO_6^+$) requires *m/z* 371.1465, found *m/z* 371.1466.

Synthesis and characterisation of 32a

dimethyl 2-butyl-3-(4-methoxybenzoyl)cycloprop-2-ene-1,1-dicarboxylate (32a)

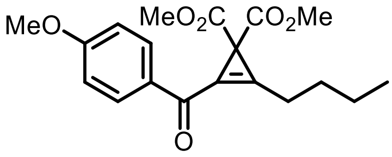

Compound **32a** was synthesised according to general procedure **VIII** by oxidation of compound **S29** (314 mg, 0.9 mmol, 1.00 eq). The crude product was purified by silica gel column chromatography (1st column: pentane/Et_2O = 7/3 to 6/4, 2nd column: pentane/EtOAc = 9/1 to 8/2, 3rd column: toluene/EtOAc = 95/5) to provide the title compound (**32a**) as a colourless oil in 52% yield (162 mg). **^{1}H NMR** (500 MHz, $CDCl_3$) δ(ppm): 8.08 – 8.01 (m, 2H, Ar$\underline{H}$), 7.02 – 6.95 (m, 2H, Ar$\underline{H}$), 3.90 (s, 3H, ArO$\underline{C}H_3$), 3.74 (s, 6H, $C(CO_2C\underline{H_3})_2$), 2.83 (t, J = 7.5 Hz, 2H, $C\underline{H_2}CH_2CH_2CH_3$), 1.69 (tt, J = 7.5, 6.5 Hz, 2H, $CH_2C\underline{H_2}CH_2CH_3$), 1.48 – 1.37 (m, 2H, $CH_2CH_2C\underline{H_2}CH_3$), 0.93 (t, J = 7.5 Hz, 3H, $CH_2CH_2CH_2C\underline{H_3}$); **^{13}C NMR** (126 MHz, $CDCl_3$) δ(ppm): 178.7 (Ar$\underline{C}$=O), 170.0 ($C(\underline{C}O_2Me)_2$), 164.6 (Ar$\underline{C}$), 131.7 (Ar$\underline{C}$H), 129.7 (Ar$\underline{C}$), 121.6 ($\underline{C}$=CC(=O)), 114.32(Ar$\underline{C}$H), 102.9 (C=$\underline{C}$C(=O)), 55.8 (PhO$\underline{C}H_3$), 52.7 ($C(CO_2\underline{C}H_3)_2$), 36.3 ($\underline{C}(CO_2CH_3)_2$), 28.4 ($CH_2\underline{C}H_2CH_2CH_3$), 25.2 ($\underline{C}H_2CH_2CH_2CH_3$), 22.5 ($CH_2CH_2CH_2CH_3$), 13.8 ($CH_2CH_2CH_2\underline{C}H_3$); **IR** (film) v_{max}/cm^{-1}: 2956, 2873 (C-H), 1725 (C=O ester), 1644 (C=O ketone); **HRMS** (ES+) exact mass calculated for $[M+H]^+$ ($C_{19}H_{23}O_6^+$) requires *m/z* 347.1493, found *m/z* 347.1489.

5.6 Synthesis of ether substrates

Synthesis and characterisation of S30

2-bromo-3-methylbut-2-en-1-ol (S30)

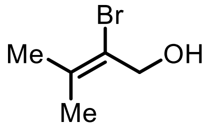

According to a literature procedure,[20] bromine (15.4 ml, 305 mmol, 1.05 eq) was added dropwise to a stirred solution of 3-methyl-but-2-en-1-ol (25.0 ml, 290 mmol, 1.00 eq) in CH_2Cl_2 (484 ml) at 0 °C over 2 h using a syringe pump. The solution was allowed to warm to room temperature overnight at which point DBU (68.6 ml, 459 mmol, 1.58 eq) was added. The solution was heated to reflux for 4 h. The reaction mixture was allowed too cool to room temperature and sat. aq. Sodium thiosulfate was added (200 ml). The phases were separated, and the aqueous layer was extracted with Et_2O (3 x 300 ml). The combined organic layers were washed with 1.0 M $HCl_{(aq)}$ (3 x 200 ml), sat. aq. $NaHCO_3$ (200 ml) and brine then dried over Na_2SO_4, filtered and concentrated *in vacuo*. The crude product (**S30**) was taken on without further purification (31.5 g). **^{1}H NMR** (400 MHz, $CDCl_3$) δ(ppm): 4.36 (s, 2H, C$\underline{H_2}$OH), 1.97 (d, *J* = 3.5 Hz, 1H, O$\underline{H}$), 1.90 (s, 3H, C$\underline{H_3}$), 1.86 (s, 3H, C$\underline{H_3}$); **^{13}C NMR** (101 MHz, $CDCl_3$) δ(ppm): 134.8 (one of $\underline{C}$=C), 121.2 (one of C=$\underline{C}$), 64.8 ($\underline{C}H_2$OH), 25.5 ($\underline{C}H_3$), 20.6 ($\underline{C}H_3$). Data are consistent with that published in the literature.[20]

Synthesis and characterisation of S31

2-bromo-1-((2-methoxyethoxy)methoxy)-3-methylbut-2-ene (S31)

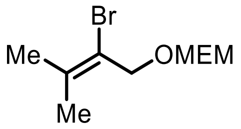

DIPEA (41.5 ml, 238 mmol, 1.25 eq) was added to a stirred solution of 2-bromo-3-methylbut-2-en-1-ol (**S30**) (31.5 g, 191 mmol, 1.00 eq) and MEMCl (32.7 ml, 286 mmol, 1.50 eq) in CH_2Cl_2 (636 ml) at 0 °C. The reaction was allowed to warm to room temperature overnight at which point H_2O was added (200 ml) and the layers separated. The aqueous layer was extracted with CH_2Cl_2 (2 x 300 ml). The combined organic layers were washed with brine, dried over Na_2SO_4, filtered and concentrated *in vacuo*. The crude product was purified by silica gel column chromatography (pentane/EtOAc = 8/2) to provide the title compound (**S31**) as a yellow oil in 58% yield (42.9 g). **^{1}H NMR** (400 MHz, $CDCl_3$) δ(ppm): 4.74 (s, 2H, $OC\underline{H_2}O$), 4.41 – 4.35 (m, 2H, $C{=}CC\underline{H_2}O$), 3.79 – 3.68 (m, 2H, $CH_2OCH_2OC\underline{H_2}CH_2$), 3.59 – 3.54 (m, 2H, $CH_2OCH_2OCH_2C\underline{H_2}$), 3.39 (s, 3H, $OC\underline{H_3}$), 1.91 (s, 3H, one of $C{=}C(C\underline{H_3})_A(CH_3)_B$), 1.87 (s, 3H, one of $C{=}C(CH_3)_A(C\underline{H_3})_B$); **^{13}C NMR** (101 MHz, $CDCl_3$) δ(ppm): 137.4 ($\underline{C}{=}CBr$), 116.8 ($C{=}\underline{C}Br$), 94.2 ($O\underline{C}H_2O$), 71.9 ($CH_2OCH_2OCH_2\underline{C}H_2$), 68.7 ($C{=}C\underline{C}H_2O$), 67.1 ($CH_2OCH_2O\underline{C}H_2CH_2$), 59.1 ($O\underline{C}H_3$), 25.6 (one of $C{=}C(\underline{C}H_3)_A(CH_3)_B$), 20.8 (one of $C{=}C(CH_3)_A(\underline{C}H_3)_B$); **IR** (film) ν_{max}/cm^{-1}: 1998, 2981 (C-H), 1655 (C=C); **HRMS** (ES+) exact mass calculated for $[M+H]^+$ ($BrC_9H_{18}O_3^+$) requires *m/z* 253.0434, found *m/z* 253.0435.

Synthesis and characterisation of S32

<u>1,1,2-tribromo-2-(((2-methoxyethoxy)methoxy)methyl)-3,3-dimethylcyclopropane (S32)</u>

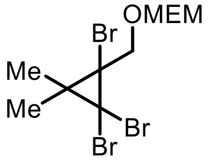

Compound **S32** was synthesised according to general procedure **VI** by addition of bromoform (45.5 ml, 510 mmol, 3.00 eq) to **S31** (42.9 g, 170 mmol, 1.00 eq). The reaction mixture was warmed to 50 °C and after 5 days more bromoform (45.5 ml, 510 mmol, 3.00 eq) and 50% aq. NaOH (42.5 ml) was added. The reaction was stirred for a further 5 days at which point H_2O (300 ml) was added, the layers were separated and the aqueous phase extracted with CH_2Cl_2 (2 x 300 ml). The combined organic layers were washed with brine, dried over Na_2SO_4, filtered and concentrated *in vacuo*. The crude product was purified by silica gel column chromatography (1st column: pentane/EtOAc = 8/2, 2nd column: pentane/EtOAc = 1/0 to 95/5 to 9/1 to 1/1) to provide the title compound (**S32**) as a yellow oil in 84% yield (60.6 g). **^{1}H NMR** (500 MHz, $CDCl_3$) δ(ppm): 4.84 (d, *J* = 7.0 Hz, 1H, one of $OC\underline{H_A}H_BO$), 4.82 (d, *J* = 7.0 Hz, 1H, one of $OCH_A\underline{H_B}O$), 4.06 (d, *J* = 11.5 Hz, 1H, one of $C\underline{H_A}H_BCBr$), 4.02 (d, *J* = 11.5 Hz, 1H, one of $CH_A\underline{H_B}CBr$), 3.86 – 3.71 (m, 2H, $C\underline{H_2}CH_2OCH_3$), 3.58 – 3.55 (m, 2H, $CH_2C\underline{H_2}OCH_3$), 3.40 (s, 3H, $CH_2CH_2OC\underline{H_3}$), 1.52 (s, 3H, one of $C(C\underline{H_3})_A(CH_3)_B$), 1.45 (s, 3H, one of $C(CH_3)_A(C\underline{H_3})_B$); **^{13}C NMR** (126 MHz, $CDCl_3$) δ(ppm): 95.8 ($O\underline{C}H_2O$), 73.2 ($O\underline{C}H_2CBr$), 71.8 ($\underline{C}H_2OCH_3$), 67.4 ($\underline{C}H_2CH_2OCH_3$), 59.2 ($O\underline{C}H_3$), 53.5 ($CH_2\underline{C}Br$ or $\underline{C}Br_2$), 48.12 ($CH_2\underline{C}Br$ or $\underline{C}Br_2$), 33.9 ($\underline{C}(CH_3)_3$), 27.0 (one of $C(\underline{C}H_3)_A(CH_3)_B$), 21.1 ($C(CH_3)_A(\underline{C}H_3)_B$); **IR** (film) ν_{max}/cm^{-1}: 2990, 2930, 2884 (C-H), 1456, 1370, 1043; **HRMS** (MSS+) exact mass calculated for $[M+Na]^+$ ($C_{10}Br_3H_{17}Na^+$) requires *m/z* 444.8620, found *m/z* 444.8620.

Synthesis and characterisation of 33a

benzyl 2-(((2-methoxyethoxy)methoxy)methyl)-3,3-dimethylcycloprop-1-ene-1-carboxylate (33a)

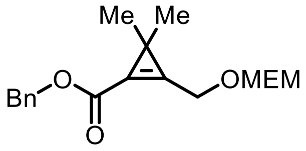

Compound **33a** was synthesised according to general procedure **VII** by the addition of tribromocyclopropane **S32** (3.00 g, 7.11 mmol, 1.00 eq) to benzyl chloroformate (1.53 ml, 10.7 mmol, 1.50 eq). The crude product was purified by silica gel column chromatography (1st column: pentane/EtOAc = 9/1, 2nd column: pentane/Et_2O = 9/1) to provide the title compound (**33a**) as a colourless oil in 32% yield (739 mg). **^{1}H NMR** (500 MHz, $CDCl_3$) δ(ppm): 7.44 – 7.29 (m, 5H, Ar$\underline{H}$), 5.23 (s, 2H, $C\underline{H}_2Ph$), 4.79 (s, 2H, $OC\underline{H}_2OCH_2C{=}C$), 4.72 (s, 2H, $OCH_2OC\underline{H}_2C{=}C$), 3.73 – 3.67 (m, 2H, $C\underline{H}_2OCH_2OCH_2C{=}C$), 3.58 – 3.51 (m, 2H, $C\underline{H}_2CH_2OCH_2OCH_2C{=}C$), 3.38 (s, 3H, $OC\underline{H}_3$), 1.29 (s, 6H, $C(C\underline{H}_3)_2$); **^{13}C NMR** (126 MHz, $CDCl_3$) δ(ppm): 161.6 ($\underline{C}{=}O$), 144.2 ($\underline{C}{=}CC({=}O)$), 136.0 (Ar$\underline{C}$), 128.7 (Ar$\underline{C}$H), 128.3 (Ar$\underline{C}$H), 128.2 (Ar$\underline{C}$H), 119.2 ($C{=}\underline{C}C({=}O)$), 94.6 ($\underline{C}H_2C{=}C$), 71.9 ($\underline{C}H_2OCH_3$), 67.3 ($\underline{C}H_2CH_2OCH_3$), 66.6 ($\underline{C}H_2Ph$), 61.9 ($O\underline{C}H_2O$), 59.2 ($O\underline{C}H_3$), 26.7 ($\underline{C}(CH_3)_2$), 25.7 ($C(\underline{C}H_3)_2$); **IR** (film) v_{max}/cm^{-1}: 3035, 2941, 2887 (C-H), 1703 (C=O); **HRMS** (ES+) exact mass calculated for $[M+H]^+$ ($C_{18}H_{25}O_5^+$) requires *m/z* 321.1697, found *m/z* 321.1697.

Synthesis and characterisation of S33

3-phenoxybenzyl carbonochloridate (S33)

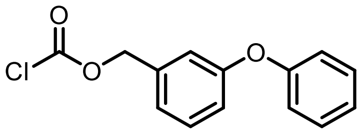

According to a literature procedure,[21] (3-phenoxyphenyl)methanol (1.74 ml, 10.0 mmol, 1.00 eq) in toluene (20 ml) was added dropwise over 30 min to a stirred mixture of triphosgene (1.54 g, 5.20 mmol, 0.52 eq), Na_2CO_3 (1.06 g, 10.0 mmol, 1.00 eq) and DMF (0.210 ml) in toluene (20 ml) at 0 °C. The reaction was stirred overnight and allowed to reach room temperature at which point completion was observed by TLC (pentane/EtOAc = 7/3). The reaction mixture was filtered through a pad of celite and concentrated *in vacuo*. The crude reaction mixture was flushed through a pad of silica (pentane/Et_2O = 95/5) to provide the title compound (**S33**) as a colourless oil in 85% yield (2.24 g) which was taken on without further purification. **^{1}H NMR** (400 MHz, $CDCl_3$) δ(ppm): 7.41 – 7.32 (m, 3H, ArH), 7.18 – 7.09 (m, 2H, ArH), 7.06 – 6.97 (m, 4H, ArH), 5.26 (s, 2H).

Synthesis and characterisation of 34a

3-phenoxybenzyl 2-(((2-methoxyethoxy)methoxy)methyl)-3,3-dimethylcycloprop-1-ene-1-carboxylate (34a)

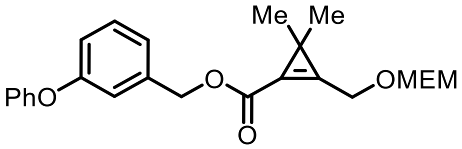

Compound **34a** was synthesised according to general procedure **VII** by the addition of tribromocyclopropane **S32** (3.00 g, 7.11 mmol, 1.00 eq) to **S33** (2.24 g, 8.53 mmol, 1.20 eq). The crude product was purified by silica gel column chromatography (1st column: pentane/Et_2O = 8/2 to 6/4, 2nd column: pentane/Et_2O = 9/1 to 8/2, 3rd column: CH_2Cl_2/Et_2O = 1/0 to 95/5) to provide the title compound (**34a**) as a colourless oil in 30% yield (879 mg). **^{1}H NMR** (500 MHz, $CDCl_3$) δ(ppm): 7.39 – 7.28 (m, 3H, ArH), 7.15 – 7.05 (m, 2H, ArH), 7.05 – 6.98 (m, 3H, ArH), 6.95 (m, 1H, ArH), 5.20 (s, 2H, CH_2Ph), 4.79 (s, 2H, OCH_2OCH_2C=C), 4.71 (s, 2H, OCH_2OCH_2C=C), 3.75 – 3.67 (m, 2H, $CH_2OCH_2OCH_2C$=C), 3.58 – 3.49 (m, 2H, $CH_2CH_2OCH_2OCH_2C$=C), 3.38 (s, 3H, OCH_3), 1.27 (s, 6H, $C(CH_3)_2$); **^{13}C NMR** (126 MHz, $CDCl_3$) δ(ppm): 161.4 (C=O), 157.7 (ArC), 157.0 (ArC), 144.5 (C=CC(=O)), 138.0 (ArC), 130.0 (ArCH), 130.0 (ArCH), 123.7 (ArCH), 122.6 (ArCH), 119.3 (C=CC(=O)), 119.1 (ArCH), 118.4 (ArCH), 118.1 (ArCH), 94.6 (CH_2C=C), 71.9 (CH_2OCH_3), 67.3 ($CH_2CH_2OCH_3$), 66.1 (CH_2Ar), 61.9 (OCH_2O), 59.2 (OCH_3), 26.8 ($C(CH_3)_2$), 25.6 ($C(CH_3)_2$); **IR** (film) ν_{max}/cm^{-1}: 2942, 2887 (C-H), 1750 (C=O); **HRMS** (ES+) exact mass calculated for $[M+Na]^+$ ($C_{24}H_{28}NaO_6^+$) requires *m/z* 435.1778, found *m/z* 435.1774.

Synthesis and characterisation of S34

2,3,5,6-tetrafluoro-4-methylbenzyl carbonochloridate (S34)

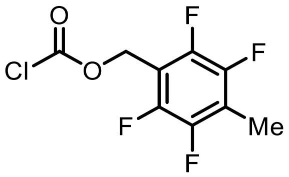

According to a literature procedure,[21] (2,3,5,6-tetrafluoro-4-methylphenyl)methanol (1.94 ml, 10.0 mmol, 1.00 eq) in toluene (20 ml) was added dropwise over 30 min to a stirred mixture of triphosgene (1.54 g, 5.20 mmol, 0.52 eq), Na_2CO_3 (1.06 g, 10.0 mmol, 1.00 eq) and DMF (0.21 ml) in toluene (20 ml) at 0 °C. The reaction was stirred overnight and allowed to reach room temperature at which point completion was observed by TLC (pentane/EtOAc = 7/3). The reaction mixture was filtered through a pad of celite and concentrated *in vacuo*. The crude reaction mixture was flushed through a pad of silica (pentane/Et_2O = 95/5) to provide the title compound (**S34**) as a colourless oil in >99% yield (2.61 g) which was taken on without further purification. **^{1}H NMR** (400 MHz, $CDCl_3$) δ(ppm): 5.41 (t, J_{HF} = 1.5 Hz, 2H, C<u>H</u>$_2$OC(=O)), 2.31 (t, J_{HF} = 2.0 Hz, 3H, C<u>H</u>$_3$).

Synthesis and characterisation of 35a

2,3,5,6-tetrafluoro-4-methylbenzyl 2-(((2-methoxyethoxy)methoxy)methyl)-3,3-dimethylcycloprop-1-ene-1-carboxylate (35a)

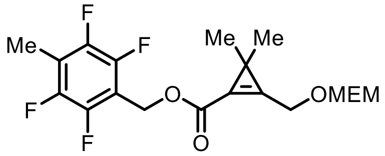

Compound **35a** was synthesised according to general procedure **VII** by the addition of tribromocyclopropane **S32** (1.00 g, 2.37 mmol, 1.00 eq) to **S34** (0.727 g, 2.84 mmol, 1.20 eq). The crude product was purified by silica gel column chromatography (pentane/Et_2O = 9/1 to 8/2) to provide the title compound (**35a**) as a colourless oil in 41% yield (399 mg). **^{1}H NMR** (500 MHz, $CDCl_3$) δ(ppm): 5.31 (t, J_{HF} = 1.5 Hz, 2H, C$\underline{H_2}$Ar), 4.79 (s, 2H, C$\underline{H_2}$C=CCO$_2$), 4.70 (s, 2H, O$\underline{C}$H$_2$O), 3.74 – 3.69 (m, 2H, CH$_2$C$\underline{H_2}$OCH$_2$O), 3.58 – 3.52 (m, 2H, C$\underline{H_2}$CH$_2$OCH$_2$O), 3.38 (s, 3H, OC$\underline{H_3}$), 2.29 (t, J = 2.0 Hz, 3H, C$\underline{H_3}$Ar), 1.27 (s, 6H, C(C$\underline{H_3}$)$_2$); **^{13}C NMR** (126 MHz, $CDCl_3$) δ(ppm): 161.1 ($\underline{C}$O$_2$CH$_2$), 146.5 – 145.8 (m, Ar$\underline{C}$), 145.3 (Ar$\underline{C}$), 144.1 ($\underline{C}$=CCO$_2$), 118.7 (C=$\underline{C}$CO$_2$), 117.5 (t, J_{CF} = 19.0 Hz, Ar$\underline{C}$), 111.46 (t, J_{CF} = 17.0 Hz, Ar$\underline{C}$), 94.5 (O$\underline{C}$H$_2$O), 71.9 ($\underline{C}$H$_2$OCH$_3$), 67.3 ($\underline{C}$H$_2$CH$_2$OCH$_3$), 61.8 ($\underline{C}$H$_2$C=CCO$_2$), 59.2 (O$\underline{C}$H$_3$), 54.2 (d, J_{CF} = 3.5 Hz, $\underline{C}$H$_2$Ph), 26.9 ($\underline{C}$Me$_2$), 25.6 (C($\underline{C}$H$_3$)$_2$), 7.9 ($\underline{C}$H$_3$Me); **^{19}F NMR** (471 MHz, $CDCl_3$) δ(ppm): -143.5 – -143.6 (m, 2 x ArC$\underline{F}$), -144.18 – -144.30 (m, 2 x ArC$\underline{F}$);); **IR** (film) ν_{max}/cm^{-1}: 2981, 2888 (C-H), 1832, 1712 (C=O), 1490; **HRMS** (ES+) exact mass calculated for [M+Na]$^+$ ($C_{19}F_4H_{22}NaO_5^+$) requires *m/z* 429.1296, found *m/z* 429.1295.

Synthesis and characterisation of S35

<u>2,3,5,6-tetrafluoro-4-(methoxymethyl)benzyl carbonochloridate (S35)</u>

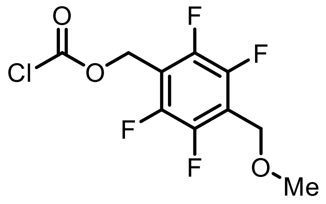

According to a literature procedure,[21] (2,3,5,6-tetrafluoro-4-(methoxymethyl)phenyl)methanol (2.24 ml, 10.0 mmol, 1.00 eq) in toluene (20 ml) was added dropwise over 30 min to a stirred mixture of triphosgene (1.54 g, 5.20 mmol, 0.52 eq), Na_2CO_3 (1.06 g, 10.0 mmol, 1.00 eq) and DMF (0.21 ml) in toluene (20 ml) at 0 °C. The reaction was stirred overnight and allowed to reach room temperature at which point completion was observed by TLC (pentane/EtOAc = 7/3). The reaction mixture was filtered through a pad of celite and concentrated *in vacuo*. The crude reaction mixture was flushed through a pad of silica (pentane/Et_2O = 95/5) to provide the title compound (**S35**) as a colourless oil in >97% yield (2.78 g) which was taken on without further purification. **^{1}H NMR** (400 MHz, $CDCl_3$) δ(ppm): 5.43 (t, J_{HF} = 1.5 Hz, 3H, one of $OC\underline{H_3}$), 4.60 (t, J_{HF} = 2.0 Hz, 3H, one of $OC\underline{H_3}$), 3.41 (s, 4H, $C\underline{H_2}$OC(=O) and $C\underline{H_2}OCH_3$).

Synthesis and characterisation of 36a

2,3,5,6-tetrafluoro-4-(methoxymethyl)benzyl 2-(((2-methoxyethoxy)methoxy)methyl)-3,3-dimethylcycloprop-1-ene-1-carboxylate (36a)

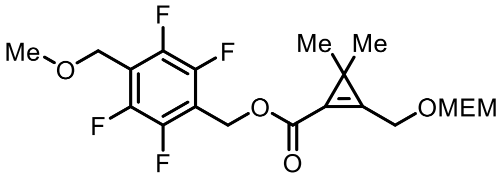

Compound **36a** was synthesised according to general procedure **VII** by the addition of tribromocyclopropane **S32** (1.00 g, 2.37 mmol, 1.00 eq) to **S35** (0.814 g, 2.84 mmol, 1.20 eq). The crude product was purified by silica gel column chromatography (pentane/Et_2O = 8/2 to 7/3) to provide the title compound (**36a**) as a colourless oil in 44% yield (451 mg). **^{1}H NMR** (500 MHz, $CDCl_3$) δ(ppm): 5.33 (t, J_{HF} = 1.5 Hz, 2H, C$\underline{H_2}$Ar), 4.79 (s, 2H, OC$\underline{H_2}$O), 4.70 (s, 2H, $OCH_2O\underline{C}H_2$), 4.58 (t, J_{HF} = 2.0 Hz, 2H, ArC$\underline{H_2}$$OCH_3$), 3.74 – 3.68 (m, 2H, $CH_3OCH_2C\underline{H_2}O$), 3.57 – 3.52 (m, 2H, $CH_3OC\underline{H_2}CH_2O$), 3.41 (s, 3H, $C\underline{H_3}OCH_2Ar$), 3.38 (s, 3H, $C\underline{H_3}OCH_2CH_2O$), 1.27 (s, 6H, $C(C\underline{H_3})_2$); **^{13}C NMR** (126 MHz, $CDCl_3$) δ(ppm): 161.0 ($\underline{C}O_2CH_2$), 146.4 – 146.0 (m, Ar$\underline{C}$), 145.7 ($\underline{C}$=CCO_2), 144.5 – 144.1 (m, Ar$\underline{C}$), 118.6 (C=$\underline{C}CO_2$), 117.8 (t, J_{CF} = 18.0 Hz, Ar$\underline{C}$), 114.6 (t, J_{CF} = 17.0 Hz, Ar$\underline{C}$), 94.5 ($O\underline{C}H_2O$), 71.9 ($CH_3O\underline{C}H_2CH_2O$), 67.3 ($CH_3OCH_2\underline{C}H_2O$), 61.8 (C=$C\underline{C}H_2OCH_2O$), 61.6 (t, J_{CF} = 2.5 Hz, Ar$\underline{C}H_2OCH_3$), 59.1 ($\underline{C}H_3OCH_2CH_2O$), 58.7 ($ArCH_2O\underline{C}H_3$), 54.2 – 54.0 (m, $\underline{C}H_2Ar$), 26.9 ($\underline{C}(CH_3)_2$), 25.5 ($C(\underline{C}H_3)_2$); **^{19}F NMR** (471 MHz, $CDCl_3$) δ(ppm): -142.6 – -142.7 (m, 2 x ArC$\underline{F}$), -143.36 – -143.51 (m, 2 x ArC$\underline{F}$); **IR** (film) ν_{max}/cm^{-1}: 2928, 2888 (C-H), 1832, 1712 (C=O), 1489; **HRMS** (ES+) exact mass calculated for $[M+Na]^+$ ($C_{20}F_4H_{24}NaO_6^+$) requires *m/z* 459.1401, found *m/z* 459.1400.

Synthesis and characterisation of S36

(2-methyl-[1,1'-biphenyl]-3-yl)methyl carbonochloridate (S36)

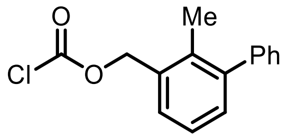

According to a literature procedure,[21] (2-methyl-[1,1'-biphenyl]-3-yl)methanol (1.98 ml, 10.0 mmol, 1.00 eq) in toluene (20 ml) was added dropwise over 30 min to a stirred mixture of triphosgene (1.54 g, 5.20 mmol, 0.52 eq), Na_2CO_3 (1.06 g, 10.0 mmol, 1.00 eq) and DMF (0.21 ml) in toluene (20 ml) at 0 °C. The reaction was stirred overnight and allowed to reach room temperature at which point completion was observed by TLC (pentane/EtOAc = 7/3). The reaction mixture was filtered through a pad of celite and concentrated *in vacuo*. The crude reaction mixture was flushed through a pad of silica (pentane/Et_2O = 95/5) to provide the title compound (**S36**) as a colourless oil in >98% yield (2.56 g) which was taken on without further purification. **^{1}H NMR** (400 MHz, $CDCl_3$) δ(ppm): 7.46 – 7.40 (m, 2H, Ar<u>H</u>), 7.40 – 7.33 (m, 2H, Ar<u>H</u>), 7.33 – 7.22 (m, 4H, Ar<u>H</u>), 5.42 (s, 2H, C<u>H</u>$_2$), 2.26 (s, 3H, C<u>H</u>$_3$).

Synthesis and characterisation of 37a

(2-methyl-[1,1'-biphenyl]-3-yl)methyl 2-(((2-methoxyethoxy)methoxy)methyl)-3,3-dimethylcycloprop-1-ene-1-carboxylate (37a)

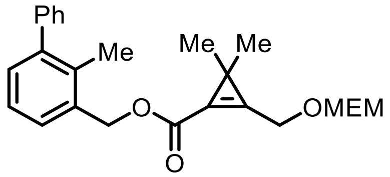

Compound **37a** was synthesised according to general procedure **VII** by the addition of tribromocyclopropane **S32** (1.00 g, 2.37 mmol, 1.00 eq) to **S36** (0.740 g, 2.84 mmol, 1.20 eq). The crude product was purified by silica gel column chromatography (pentane/Et_2O = 9/1) to provide the title compound (**37a**) as a colourless oil in 35% yield (343 mg). **^{1}H NMR** (500 MHz, $CDCl_3$) δ(ppm): 7.44 – 7.33 (m, 4H, ArC$\underline{H}$), 7.32 – 7.27 (m, 2H, ArC$\underline{H}$), 7.26 – 7.18 (m, 2H, ArC$\underline{H}$), 5.31 (s, 2H, C$\underline{H_2}$Ar), 4.80 (s, 2H, OC$\underline{H_2}$O), 4.72 (s, 2H, $OCH_2O\underline{C}H_2C{=}C$), 3.73 – 3.68 (m, 2H, $CH_3OCH_2C\underline{H_2}O$), 3.56 – 3.51 (m, 2H, $CH_3OC\underline{H_2}CH_2O$), 3.38 (s, 3H, $C\underline{H_3}OCH_2CH_2O$), 2.23 (s, 3H, ArC$\underline{H_3}$), 1.30 (s, 6H, $C(C\underline{H_3})_2$); **^{13}C NMR** (126 MHz, $CDCl_3$) δ(ppm): 161.6 ($\underline{C}O_2CH_2$), 144.2 ($\underline{C}{=}CCO_2$), 143.0 (Ar$\underline{C}$), 142.0 (Ar$\underline{C}$), 134.6 (Ar$\underline{C}$), 134.5 (Ar$\underline{C}$), 130.4 (Ar$\underline{C}$H), 129.5 (Ar$\underline{C}$H), 128.4 (Ar$\underline{C}$H), 128.2 (Ar$\underline{C}$H), 127.0 (Ar$\underline{C}$H), 125.7 (Ar$\underline{C}$H), 119.2 ($C{=}\underline{C}CO_2$), 94.6 ($O\underline{C}H_2O$), 71.9 ($CH_3O\underline{C}H_2CH_2O$), 67.3 ($CH_3OCH_2\underline{C}H_2O$), 65.6 (Ar$\underline{C}H_2$), 61.9 ($C{=}C\underline{C}H_2OCH_2O$), 59.2 ($\underline{C}H_3OCH_2CH_2O$), 26.7 ($\underline{C}(CH_3)_2$), 25.7 ($C(\underline{C}H_3)_2$), 16.3 (Ar$\underline{C}H_3$); **IR** (film) ν_{max}/cm^{-1}: 2970, 2887 (C-H), 1832, 1703 (C=O); **HRMS** (ES+) exact mass calculated for $[M+Na]^+$ ($C_{25}H_{30}NaO_5^+$) requires *m/z* 433.1985, found *m/z* 433.1985.

Synthesis and characterisation of S37

1-((allyloxy)methyl)-1,2,2-tribromo-3,3-dimethylcyclopropanec (S37)

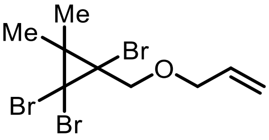

i. To a solution of compound **S32** (1.00 g, 2.35 mmol, 1.0 equiv.) in MeOH/ CH_2Cl_2 (5/1 ml) was added *conc.* HCl (5 ml), the reaction mixture was stirred at room temperature overnight. The aqueous layer was extracted with CH_2Cl_2 (3 x 10 ml). The combined organic layers were washed with brine, dried over Na_2SO_4, filtered and concentrated *in vacuo* to give the crude product as a white solid (800 mg), which was taken on without further purification.

ii. At 0 °C, to the crude from step i was added NaI (35 mg, 0.235 mmol, 10 mol%), allyl bromide (0.41 ml, 4.70 mmol, 2.0 equiv.) and DMF (40 ml), to the resulting solution was added NaH (60% dispersion in mineral oil, 141 mg, 3.50 mmol, 1.5 equiv.) portionwise. The reaction mixture was stirred at room temperature overnight. After completion as determined by TLC, the reaction was cooled to 0 °C, H_2O (2 ml) and was added dropwise. The solution was diluted with Et_2O (10 mL) and water (20 mL), the layers were separated and the aqueous phase extracted with Et_2O (2 x 10 ml). The combined organic layers were washed with brine, dried over Na_2SO_4, filtered and concentrated *in vacuo*. The crude product was purified by silica gel column chromatography (pentane/EtOAc = 20/1) to provide the title compound (**S37**) as a yellow oil in 81% yield (730 mg). **^{1}H NMR** (400 MHz, $CDCl_3$) δ(ppm) 5.94 (ddt, *J* = 17.2, 10.3, 5.7 Hz, 1H, $OCH_2C\underline{H}{=}CH_2$), 5.32 (dq, *J* = 17.2, 1.6 Hz, 1H, $OCH_2CH{=}C\underline{H_A}H_B$), 5.22 (dq, *J* = 10.4, 1.4 Hz, 1H, $OCH_2CH{=}CH_A\underline{H_B}$), 4.17 – 4.01 (m, 2H, $OC\underline{H_2}CH{=}CH_2$), 3.93 (d, *J* = 11.4 Hz, 1H, $C\underline{H_A}H_BOCH_2C\underline{H}{=}CH_2$), 3.87 (d, *J* = 11.5 Hz, 1H, $CH_A\underline{H_B}OCH_2C\underline{H}{=}CH_2$), 1.51 (s, 3H, $C(C\underline{H_3})_A(CH_3)_B$), 1.43 (s, 3H, $C(CH_3)_A(C\underline{H_3})_B$); **^{13}C NMR** (101 MHz, $CDCl_3$) δ(ppm): 134.4 ($OCH_2\underline{C}H{=}CH_2$), 118.0 ($OCH_2CH{=}\underline{C}H_2$), 74.8 ($CH_2O\underline{C}H_2CH{=}\underline{C}H_2$), 72.4

($\underline{C}H_2OCH_2CH=\underline{C}H_2$), 53.6 ($\underline{C}(CH_2)(Br)$), 48.6 ($\underline{C}Br_2$), 33.7 ($\underline{C}(CH_3)_2$), 27.0 ($C(\underline{C}H_3)_A(CH_3)_B$), 21.1 ($C(CH_3)_A(\underline{C}H_3)_B$); **HRMS (ES+)** exact mass calculated for [M+ H]+ (C9H14Br3O+) requires m/z 374.8589, found m/z 374.8606.

Synthesis and characterisation of 39a

benzyl 2-((allyloxy)methyl)-3,3-dimethylcycloprop-1-ene-1-carboxylate (39a)

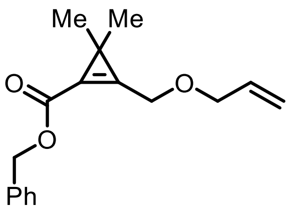

According to a modified literature procedure,[3] *n*-BuLi (2.5 M in hexanes, 1.55 mL, 2.0 eq) was added dropwise to a stirred solution of the desired tribromocyclopropane **S37** (730 mg, 1.94 mmol, 1.00 eq) in Et_2O (20 mL) at −78 °C. The reaction was stirred at −78 °C for 1 h at which point benzyl chloroformate (3 M in toluene, 0.97 mL, 2.90 mmol, 1.50 eq) was added dropwise. The temperature was raised to −20 °C and stirred for a further 2 h. The reaction mixture was stirred until completion was observed by TLC at which point the reaction was quenched with sat. aq. NH_4Cl and the aqueous phase was extracted with Et_2O (3 x 20 ml). The combined organic extracts were washed with brine then dried over Na_2SO_4, filtered and concentrated *in vacuo*. The crude product was purified by silica gel column chromatography (pentane/EtOAc = 40/1) to provide the title compound (**39a**) as a colourless oil in 36% yield (185 mg)**^{1}H NMR** (400 MHz, $CDCl_3$) δ(ppm): 7.42 – 7.32 (m, 5H, Ar$\underline{H}$), 5.89 (ddt, *J* = 17.2, 10.4, 5.7 Hz, 1H, C$\underline{H}$=CH_AH_B), 5.30 – 5.23 (m, 1H, CH=C$\underline{H_A}H_B$), 5.25 (s, 2H, C$\underline{H_2}$Ph), 5.18 (dq, *J* = 10.4, 1.4 Hz, 1H, CH=C$H_A\underline{H_B}$), 4.62 (s, 2H, C=C C$\underline{H_2}$O), 4.09 (t, *J* = 1.4 Hz, 1H, OC$\underline{H_A}H_B$CH=CH_2), 4.08 (t, *J* = 1.4 Hz, 1H, OC$H_A\underline{H_B}$CH=CH_2), 1.30 (s, 6H, C(C$\underline{H_3}$)$_2$); **^{13}C NMR** (101 MHz, $CDCl_3$) δ(ppm)161.6 ($\underline{C}$=O), 144.3 ($\underline{C}$=CC(=O)), 135.9 (Ar$\underline{C}$), 134.2 (OCH_2CH=$\underline{C}H_2$), 128.6 (Ar$\underline{C}$H), 128.3 (Ar$\underline{C}$H), 128.3 (Ar$\underline{C}$H), 119.2 (C=$\underline{C}$C(=O)), 117.7 ($OCH_2\underline{C}H$=CH_2), 71.5 (O$\underline{C}H_2$CH=CH_2), 66.6 ($\underline{C}H_2$Ph), 65.0 (C=C$\underline{C}H_2$O), 26.5 ($\underline{C}(CH_3)_2$), 25.6 (C($\underline{C}H_3)_2$); **HRMS** (ES+) exact mass calculated for $[M+K]^+$ ($C_{17}H_{20}O_3K^+$) requires *m/z* 311.1044, found *m/z* 311.1031.

6. Synthesis and Characterisation of Products Obtained in the Enantioselective Deconjugation of Cyclopropenes

6.1 Synthesis of enantioenriched cyclopropane esters

Synthesis and characterisation of 3b

2-benzyl 1,1-dimethyl (R)-3-cyclohexylidenecyclopropane-1,1,2-tricarboxylate (3b)

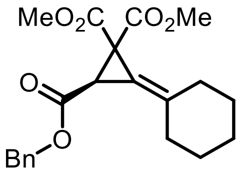

The title compound was prepared according to general procedure **II** from 2-benzyl 1,1-dimethyl 3-cyclohexylcycloprop-2-ene-1,1,2-tricarboxylate (**3a**) (18.6 mg, 0.0500 mmol, 1.00 eq) with catalyst **C11**. After 4 h the crude reaction mixture was purified by silica gel column chromatography (pentane/Et_2O = 8/2) to provide the title compound (**3b**) as a colourless oil in 99% yield (18.4 mg) and 97% ee. [determined by HPLC chiralpak OG, hexane/isopropanol = 98/2, 1 ml/min, λ = 220 nm, t(minor) = 14.04 min, t(major) = 17.93 min]. **^{1}H NMR** (500 MHz, $CDCl_3$) δ(ppm): 7.41 – 7.28 (m, 5H, Ar$\underline{H}$), 5.14 (d, *J* = 12.5 Hz, 1H, one of $C\underline{H_A}H_BPh$), 5.10 (d, *J* = 12.5 Hz, 1H, one of $CH_A\underline{H_B}Ph$), 3.74 (s, 3H, one of $C(CO_2C\underline{H_3})_A(CO_2CH_3)_B$), 3.60 (s, 3H, one of $C(CO_2CH_3)_A(CO_2C\underline{H_3})_B$), 3.32 – 3.26 (bm, 1H, $C\underline{H}C(=O)$)), 2.49 (dt, *J* = 12.0, 5.5 Hz, 1H, one of $C{=}CC\underline{H_A}H_BCH_2CH_2CH_2CH_CH_D$), 2.25 (qdd, *J* = 19.5, 17.5, 10.0, 4.5 Hz, 3H, $C{=}CCH_A\underline{H_B}CH_2CH_2CH_2C\underline{H_C}\underline{H_D}$), 1.71 – 1.46 (m, 6H, $C{=}CCH_2C\underline{H_2}C\underline{H_2}C\underline{H_2}CH_2$); **^{13}C NMR** (126 MHz, $CDCl_3$) δ(ppm): 168.1 ($\underline{C}O_2Bn$), 167.9 (one of $C(\underline{C}O_2CH_3)_A(CO_2CH_3)_B$), 165.8 (one of $C(CO_2CH_3)_A(\underline{C}O_2CH_3)_B$), 136.6 ($\underline{C}{=}CCHCO_2Bn$), 135.6 (Ar$\underline{C}$), 128.67 (Ar$\underline{C}$H) 128.5 (Ar$\underline{C}$H), 128.4 (Ar$\underline{C}$H), 111.6 ($C{=}\underline{C}CHCO_2Bn$), 67.2 ($\underline{C}H_2Ph$), 53.3 (one of $C(CO_2\underline{C}H_3)_A(CO_2CH_3)_B$), 52.9 (one of $C(CO_2CH_3)_A(CO_2\underline{C}H_3)_B$), 38.9 ($\underline{C}(CO_2Me)_2$), 33.4 ($C{=}C\underline{C}H_2CH_2CH_2CH_2CH_2$), 33.4 ($C{=}CCH_2CH_2CH_2CH_2\underline{C}H_2$), 30.5 ($\underline{C}HCO_2Bn$), 27.5 ($C{=}CCH_2\underline{C}H_2CH_2\underline{C}H_2CH_2$), 26.2 ($C{=}CCH_2CH_2\underline{C}H_2CH_2CH_2$); **IR** (film) ν_{max}/cm^{-1}: 3033, 2934,

2854 (C-H), 1734 (C=O), 1435, 1250; **HRMS** (ES+) exact mass calculated for $[M+H]^+$ ($C_{21}H_{25}O_6^+$) requires *m/z* 373.1646, found *m/z* 373.1644; $[\boldsymbol{\alpha}]_{\boldsymbol{D}}^{\mathbf{25}}$ = –59.63 (*c* 0.35, $CHCl_3$).

Synthesis and characterisation of 5b

2-benzyl 1,1-dimethyl (R)-3-cyclopentylidenecyclopropane-1,1,2-tricarboxylate (5b)

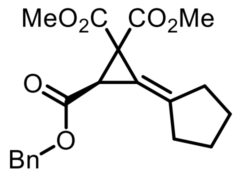

The title compound was prepared according to general procedure **II** from **5a** (17.9 mg, 0.0500 mmol, 1.00 eq) with catalyst **C11**. After 5 h the reaction was quenched and the crude reaction mixture was purified by silica gel column chromatography (pentane/Et_2O = 8/2) to provide the title compound (**5b**) as a colourless oil in 96% yield (17.2 mg) and 94% ee. [determined by HPLC chiralpak AD-H, hexane/isopropanol = 98/2, 1 ml/min, λ = 220 nm, t(major) = 18.85 min, t(minor) = 20.59 min]. **^{1}H NMR** (500 MHz, $CDCl_3$) δ(ppm): 7.40 – 7.28 (m, 5H, Ar$\underline{H}$), 5.15 (d, *J* = 12.5 Hz, 1H, one of $C\underline{H_A}H_BPh$), 5.10 (d, *J* = 12.5 Hz, 1H, , one of $CH_A\underline{H_B}Ph$), 3.75 (s, 3H, one of $C(CO_2C\underline{H_3})_A(CO_2CH_3)_B$), 3.62 (s, 3H, one of $C(CO_2CH_3)_A(CO_2C\underline{H_3})_B$), 3.25 (p, *J* = 2.5 Hz, 1H, $C\underline{H}C(=O)$), 2.80 – 2.62 (m, 1H, one of $C\underline{H_A}H_BCCH_CH_D$), 2.49 – 2.30 (m, 3H, three of $CH_A\underline{H_B}CC\underline{H_C}\underline{H_D}$), 1.75 (m, 4H, $C\underline{H_2}CH_2CCH_2C\underline{H_2}$); **^{13}C NMR** (126 MHz, $CDCl_3$) δ(ppm): 167.9 ($\underline{C}O_2Bn$), 167.8 (one of $C(\underline{C}O_2CH_3)_A(CO_2CH_3)_B$), 165.9 (one of $C(CO_2CH_3)_A(\underline{C}O_2CH_3)_B$), 140.4 ($CH_2\underline{C}=C$), 135.7 (Ar$\underline{C}$), 128.7 (Ar$\underline{C}$H), 128.5 (Ar$\underline{C}$H), 128.4 (Ar$\underline{C}$H), 109.7 ($CH_2C=\underline{C}$), 67.2 ($\underline{C}H_2Ph$), 53.3 (one of $C(CO_2\underline{C}H_3)_A(CO_2CH_3)_B$), 52.8 (one of $C(CO_2CH_3)_A(CO_2\underline{C}H_3)_B$), 39.0 ($\underline{C}(CO_2Me)_2$), 32.5 (one of $(\underline{C}H_2)_AC(CH_2)_B$), 32.3 (one of $(CH_2)_AC(\underline{C}H_2)_B$), 30.8 ($\underline{C}HC(=O)$), 26.6 (one of $(\underline{C}H_2)_ACH_2CCH_2(CH_2)_B$), 26.6 (one of $(CH_2)_ACH_2CCH_2(\underline{C}H_2)_B$); **IR** (film) ν_{max}/cm^{-1}: 2952 (C-H), 1732 (C=O), 1249; **HRMS** (ES+) exact mass calculated for $[M+H]^+$ ($C_{20}H_{23}O_6^+$) requires *m/z* 359.1489, found *m/z* 359.1493; $[\alpha]_D^{25}$ = –56.4 (*c* 0.17, $CHCl_3$).

Synthesis and characterisation of 6b

2-benzyl 1,1-dimethyl (R)-3-(1-(tert-butoxycarbonyl)piperidin-4-ylidene)cyclopropane-1,1,2-tricarboxylate (6b)

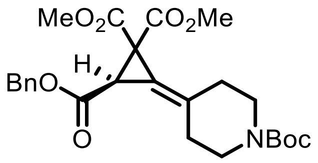

The title compound was prepared according to general procedure **II** from 2-benzyl 1,1-dimethyl 3-(1-(tert-butoxycarbonyl)piperidin-4-yl)cycloprop-2-ene-1,1,2-tricarboxylate (**6a**) (47.3 mg, 0.1000 mmol, 1.00 eq) with catalyst **C11**. After 1 h the crude reaction mixture was purified by silica gel column chromatography (pentane/Et_2O = 1/1) to provide the title compound (**6b**) as a white solid in 85% yield (40.2 mg) and 95% ee. [determined by HPLC chiralpak AD-H, hexane/isopropanol = 99/5, 1 ml/min, λ = 220 nm, t(major) = 18.52 min, t(minor) = 21.87 min]. **^{1}H NMR** (400 MHz, $CDCl_3$) δ(ppm)7.39 – 7.28 (m, 5H, Ar$\underline{H}$), 5.14 (d, *J* = 12.2 Hz, 1H, one of $C\underline{H_A}H_BPh$), 5.09 (d, *J* = 12.2 Hz, 1H, one of $CH_A\underline{H_B}Ph$), 3.75 (s, 3H, one of $(CO_2C\underline{H_3})_A(CO_2CH_3)_B$), 3.71 – 3.62 (m, 2H, $C\underline{H_A}H_BN(Boc)C\underline{H_C}H_D$), 3.61 (s, 3H, one of $(CO_2CH_3)_A(CO_2C\underline{H_3})_B$), 3.39 – 3.26 (m, 2H, $CH_A\underline{H_B}N(Boc)CH_C\underline{H_D}$), 3.18 (dt, *J* = 12.8, 6.3 Hz, 1H, $C\underline{H}CO_2Bn$), 2.56 (m, 1H, $C\underline{H_A}H_BCH_2N(Boc)CH_2CH_CH_D$), 2.38 – 2.31 (m, 1H, $CH_AH_BCH_2N(Boc)CH_2C\underline{H_C}H_D$), 2.32 – 2.24 (m, 2H, $CH_A\underline{H_B}CH_2N(Boc)CH_2CH_C\underline{H_D}$), 1.46 (s, 9H, $C(C\underline{H_3})_3$); **^{13}C NMR** (101 MHz, $CDCl_3$) δ(ppm)167.5 ($\underline{C}O_2Bn$), 167.4 (one of $(\underline{C}O_2CH_3)_A(CO_2CH_3)_B$), 165.3(one of $(CO_2CH_3)_A(\underline{C}O_2CH_3)_B$), 154.8 ($N\underline{C}O_2C(CH_3)_3$), 135.4 (Ar$\underline{C}$), 132.3 ($\underline{C}$=CCHCO$_2$Bn), 128.7 (Ar$\underline{C}$H), 128.6 (Ar$\underline{C}$H), 128.5 (Ar$\underline{C}$H), 113.9 (C=$\underline{C}$CHCO$_2$Bn), 79.9, 67.4 ($\underline{C}H_2Ph$), 53.4 (one of $C(CO_2\underline{C}H_3)_A(CO_2CH_3)_B$), 52.9 (one of $C(CO_2CH_3)_A(CO_2\underline{C}H_3)_B$), 44.6 ($\underline{C}H_2N\underline{C}H_2$), 38.8 ($\underline{C}(CO_2Me)_2$), 32.6 ($\underline{C}H_2CH_2NCH_2\underline{C}H_2$), 30.4 ($\underline{C}HCO_2Bn$), 28.5 ($NCO_2C(\underline{C}H_3)_3$); **HRMS** (ES+) exact mass calculated for $[M+Na]^+$ ($C_{25}H_{31}NO_8Na^+$) requires *m/z* 496.1942, found *m/z* 496.1932; $[\alpha]_D^{20}$ = –43.5 (*c* 2.52, $CHCl_3$).

Synthesis and characterisation of 7b

2-benzyl 1,1-dimethyl (R)-3-(tetrahydro-4H-pyran-4-ylidene)cyclopropane-1,1,2-tricarboxylate (7b)

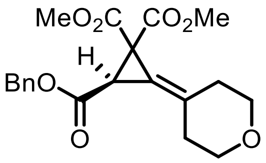

The title compound was prepared according to general procedure **II** from 2-benzyl 1,1-dimethyl 3-(tetrahydro-2H-pyran-4-yl)cycloprop-2-ene-1,1,2-tricarboxylate (**7a**) (37.4 mg, 0.100 mmol, 1.00 eq) with catalyst **C11**. After 1 h the reaction was quenched with was quenched with AcOH to give the crude product. The crude reaction mixture was purified by silica gel column chromatography (pentane/EtOAc = 2/1) to provide the title compound (**7b**) as a colourless oil in 73% yield (27.3 mg) and 96% ee. [determined by HPLC chiralcel OD, hexane/isopropanol = 95/5, 1 ml/min, λ = 220 nm, t(major) = 27.51 min, t(minor) = 8.75 min]. **^{1}H NMR** (400 MHz, $CDCl_3$) δ(ppm)7.41 – 7.28 (m, 5H, Ar$\underline{H}$), 5.14 (d, *J* = 12.2 Hz, 1H, one of $C\underline{H_A}H_BPh$), 5.09 (d, *J* = 12.2 Hz, 1H, one of $CH_A\underline{H_B}Ph$), 3.86 – 3.76 (m, 2H, one of $C{=}CCH_2C\underline{H_A}H_BOC\underline{H_C}H_DCH_2$), 3.75 (s, 3H, one of $C(CO_2C\underline{H_3})_A(CO_2CH_3)_B$), 3.68 (ddd, *J* = 11.3, 8.1, 3.8 Hz, 1H, one of $C{=}CCH_2CH_A\underline{H_B}OCH_CH_DCH_2$), 3.61 (s, 3H, one of $C(CO_2CH_3)_A(CO_2C\underline{H_3})_B$), 3.56 (ddd, *J* = 11.0, 8.0, 4.0 Hz, 1H, one of $C{=}CCH_2CH_AH_BOCH_CH_DCH_2$), 3.32 (dd, *J* = 2.2, 1.2 Hz, 1H, $C\underline{H}CO_2Bn$), 2.66 – 2.51 (m, 1H, one of $C{=}CC\underline{H_A}H_BCH_2OCH_2CH_CH_D$), 2.48 – 2.35 (m, 2H, one of $C{=}CCH_A\underline{H_B}CH_2OCH_2C\underline{H_C}H_D$), 2.35 – 2.27 (m, 1H, one of $C{=}CCH_AH_BCH_2OCH_2CH_C\underline{H_D}$); **^{13}C NMR** (101 MHz, $CDCl_3$) δ(ppm)167.6 ($\underline{C}O_2Bn$), 167.4 (one of $C(\underline{C}O_2CH_3)_A(CO_2CH_3)_B$), 165.4(one of $C(CO_2CH_3)_A(\underline{C}O_2CH_3)_B$), 135.4 ($\underline{C}{=}CCHCO_2Bn$), 131.6 (Ar$\underline{C}$), 128.7 (Ar$\underline{C}$H), 128.6 (Ar$\underline{C}$H), 128.5 (Ar$\underline{C}$H), 113.2($C{=}\underline{C}CHCO_2Bn$), 68.6 ($\underline{C}H_2Ph$), 68.4 (one of $\underline{C}H_2OCH_2$), 67.4 (one of $CH_2O\underline{C}H_2$), 53.4 (one of $C(CO_2\underline{C}H_3)_A(CO_2CH_3)_B$), 53.0 (one of $C(CO_2CH_3)_A(CO_2\underline{C}H_3)_B$), 38.8 ($\underline{C}(CO_2Me)_2$), 33.6($C{=}C\underline{C}H_2CH_2OCH_2\underline{C}H_2$), 30.4 ($\underline{C}HCO_2Bn$);

HRMS (ES+) exact mass calculated for $[M+H]^+$ ($C_{20}H_{23}O_7^+$) requires *m/z* 375.1438, found *m/z* 375.1436; $[\boldsymbol{\alpha}]_{\boldsymbol{D}}^{\mathbf{20}}$ = –64.2 (*c* 1.49, $CHCl_3$).

Synthesis and characterisation of 8b

2-allyl 1,1-dimethyl (R)-3-cyclohexylidenecyclopropane-1,1,2-tricarboxylate (8b)

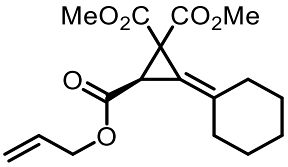

The title compound was prepared according to general procedure **II** from 2-allyl 1,1-dimethyl 3-cyclohexylcycloprop-2-ene-1,1,2-tricarboxylate (**8a**) (16.1 mg, 0.0500 mmol, 1.00 eq) with catalyst **C11**. After 9 h the reaction was quenched and the crude reaction mixture was purified by silica gel column chromatography (pentane/Et_2O = 8/2 then toluene/Et_2O = 97.5:2.5) to provide the title compound (**8b**) as a colourless oil in 75% yield (12.1 mg) and 97% ee. [determined by HPLC chiralpak OG, hexane/isopropanol = 98/2, 1 ml/min, λ = 220 nm, t(minor) = 9.66 min, t(major) = 11.97 min]. **^{1}H NMR** (400 MHz, $CDCl_3$) δ(ppm): 5.87 (ddt, *J* = 17.0, 10.5, 5.5 Hz, 1H, $C\underline{H}{=}CH_2$), 5.30 (app-dq, *J* = 17.0, 1.5 Hz, 1H, one of $CH{=}C\underline{H_A}H_B$), 5.22 (app-dq, *J* = 10.5, 1.5 Hz, 1H, one of $CH{=}CH_A\underline{H_B}$), 4.66 – 4.49 (m, 2H, $OC\underline{H_2}$), 3.74 (s, 3H, one of $OC\underline{H_3}$), 3.72 (s, 3H, one of $OC\underline{H_3}$), 3.26 – 3.23 (m, 1H, $C\underline{H}C({=}O)$), 2.53 – 2.43 (m, 1H, one of $C{=}C(C\underline{H_A}H_B)(CH_CH_D)$), 2.37 – 2.13 (m, 3H, three of $C{=}C(CH_A\underline{H_B})(C\underline{H_C}\underline{H_D})$), 1.71 – 1.49 (m, 6H, $CH_2C\underline{H_2}C\underline{H_2}C\underline{H_2}CH_2$); **^{13}C NMR** (101 MHz, $CDCl_3$) δ(ppm): 167.9 (one of $\underline{C}O_2Me$), 167.8 (one of $\underline{C}O_2Me$), 165.8 ($\underline{C}O_2CH_2$), 136.6 ($\underline{C}{=}C(CH_2)_2$ or $C{=}\underline{C}(CH_2)_2$), 131.8 ($\underline{C}H{=}CH_2$), 118.5 ($CH{=}\underline{C}H_2$), 111.6 ($\underline{C}{=}C(CH_2)_2$ or $C{=}\underline{C}(CH_2)_2$), 66.0 ($O\underline{C}H_2$), 53.2 (one of $O\underline{C}H_3$), 52.9 (one of $O\underline{C}H_3$), 38.9 ($\underline{C}(CO_2Me)$), 33.4 ($\underline{C}H_2CH_2CH_2CH_2CH_2$ or $CH_2CH_2CH_2CH_2\underline{C}H_2$), 33.3 ($\underline{C}H_2CH_2CH_2CH_2CH_2$ or $CH_2CH_2CH_2CH_2\underline{C}H_2$), 30.4 ($\underline{C}HC({=}O)$), 27.5 ($CH_2\underline{C}H_2CH_2CH_2CH_2$ or $CH_2CH_2CH_2\underline{C}H_2CH_2$), 27.5 ($CH_2\underline{C}H_2CH_2CH_2CH_2$ or $CH_2CH_2CH_2\underline{C}H_2CH_2$), 26.2 ($CH_2CH_2\underline{C}H_2CH_2CH_2$); **IR** (film) v_{max}/cm^{-1}: 2933, 2856 (C-H), 1731 (C=O); **HRMS** (ES+) exact mass calculated for $[M+H]^+$ ($C_{17}H_{23}O_6^+$) requires *m/z* 323.1489, found *m/z* 323.1489; $[\alpha]_D^{25}$ = –73.00 (*c* 0.34, $CHCl_3$).

Synthesis and characterisation of 9b

2-benzyl 1,1-dimethyl (R,Z)-3-butylidenecyclopropane-1,1,2-tricarboxylate (9b)

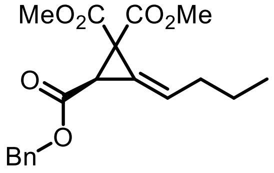

The title compound was prepared according to general procedure **II** from 2-benzyl 1,1-dimethyl 3-butylcycloprop-2-ene-1,1,2-tricarboxylate (**9a**) (34.6 mg, 0.100 mmol, 1.00 eq) with catalyst **C11**. After 30 min the reaction was quenched with sat. aq. NH_4Cl (5 ml) and extracted with Et_2O (3 x 10 ml). The combined organic layers were washed with brine, dried over Na_2SO_4, filtered and concentrated *in vacuo* providing the crude compound in >20:1 d.r. The crude reaction mixture was purified by silica gel column chromatography (pentane/Et_2O = 8/2) to provide the title compound (**9b**) as a colourless solid in 97% yield (32.5 mg), 95% ee. [determined by HPLC chiralpak AS-H, hexane/isopropanol = 90/10, 1 ml/min, λ = 210 nm, t(minor) = 7.31 min, t(major) = 10.12 min]; **^{1}H NMR** (500 MHz, $CDCl_3$) δ(ppm)7.40 – 7.29 (m, 5H, Ar$\underline{H}$), 6.10 (td, *J* = 7.5, 2.0 Hz, 1H, $CH_2C\underline{H}$=C), 5.14 (d, *J* = 12.5 Hz, 1H, one of $C\underline{H_A}H_BPh$), 5.10 (d, *J* = 12.5 Hz, 1H, one of $CH_A\underline{H_B}Ph$), 3.75 (s, 3H, one of $(CO_2C\underline{H_3})_A(CO_2CH_3)_B$), 3.61 (s, 3H, one of $(CO_2CH_3)_A(CO_2C\underline{H_3})_B$), 3.30 (app-q, *J* = 2.0 Hz, 1H, $C\underline{H}CO_2Bn$), 2.41 – 2.24 (m, 2H, $CHC\underline{H_2}$), 1.50 (h, *J* = 7.5 Hz, 2H, $CHCH_2C\underline{H_2}$), 0.90 (t, *J* = 7.4 Hz, 3H, $CH_2C\underline{H_3}$); **^{13}C NMR** (126 MHz, $CDCl_3$) δ(ppm)167.8 ($\underline{C}O_2Bn$), 167.4 (one of $(\underline{C}O_2CH_3)_A(CO_2CH_3)_B$), 165.3 (one of $(CO_2CH_3)_A(\underline{C}O_2CH_3)_B$), 135.4 (Ar$\underline{C}$), 128.7 (Ar$\underline{C}$H), 128.5 (Ar$\underline{C}$H) (one Ar$\underline{C}$H is hidden), 124.8 ($CH_2\underline{C}H$=C), 119.8 ($CH_2CH$=$\underline{C}$), 67.4 ($\underline{C}H_2Ph$), 53.4 (one of $(CO_2\underline{C}H_3)_A(CO_2CH_3)_B$), 52.9 (one of $(CO_2CH_3)_A(CO_2\underline{C}H_3)_B$), 38.5 ($\underline{C}(CO_2Me)_2$), 33.4 ($\underline{C}H_2CH$=C), 30.5 ($\underline{C}HCO_2Bn$), 21.9 ($CH_2\underline{C}H_2CH_3$), 13.7 ($CH_2CH_2\underline{C}H_3$); **IR** (film) ν_{max}/cm^{-1}: 3027, 2956, 2873 (C-H), 1733 (C=O), 1713 (C=O), 1604 (C=C); **HRMS** (ES+) exact

mass calculated for $[M+H]^+$ ($C_{19}H_{23}O_6^+$) requires *m/z* 347.1489, found *m/z* 347.1494; **MP**: 32 – 34 °C; $[\boldsymbol{\alpha}]_{\boldsymbol{D}}^{\mathbf{25}}$ = –109.06 (*c* 3.25, $CHCl_3$).

Synthesis and characterisation of 10b

trimethyl (R,Z)-3-butylidenecyclopropane-1,1,2-tricarboxylate (10b)

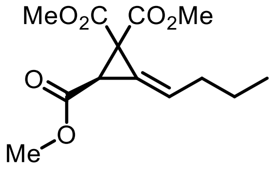

The title compound was prepared according to general procedure **II** from trimethyl 3-butylcycloprop-2-ene-1,1,2-tricarboxylate (**10a**) (27.0 mg, 0.100 mmol, 1.00 eq) with catalyst **C11**. After 2 h the crude mixture was quenched with AcOH to give the crude product in >20:1 d.r. The crude product was purified by silica gel column chromatography (pentane/Et_2O = 7/3) to provide the title compound (**10b**) as a colourless oil in quantitative yield (27.2 mg) and 97% ee. [determined by SFC Chiralcel IC, 1500 psi, 1% to 30% MeOH over 5 min, then from 30% to 50% MeOH in 0.5 min, then hold 50% MeOH for 1.5 min, 1.5 mL/min, t(major) = 2.27 min, t(minor) = 1.91 min]. **^{1}H NMR** (500 MHz, $CDCl_3$) δ(ppm): 6.10 (td, *J* = 7.5, 2.0 Hz, 1H, C=C<u>H</u>), 3.77 (s, 3H, one of CO_2C<u>H_3</u>), 3.76 (s, 3H, one of CO_2C<u>H_3</u>), 3.70 (s, 3H, one of CO_2C<u>H_3</u>), 3.25 (q, *J* = 2.0 Hz, 1H, C<u>H</u>CO_2Me), 2.41 – 2.24 (m, 2H, C<u>H_2</u>CH_2CH_3), 1.50 (h, *J* = 7.5 Hz, 2H, CH_2C<u>H_2</u>CH_3), 0.90 (t, *J* = 7.5 Hz, 3H, CH_2CH_2C<u>H_3</u>); **^{13}C NMR** (126 MHz, $CDCl_3$) δ(ppm): 168.3 (one of <u>C</u>=O), 167.5 (one of <u>C</u>=O), 165.4 (one of <u>C</u>=O), 124.8 (C=<u>C</u>H), 119.8 (<u>C</u>=CH), 53.4 (one of CO_2<u>C</u>H_3), 53.1 (one of CO_2<u>C</u>H_3), 52.7 (one of CO_2<u>C</u>H_3), 38.4 (<u>C</u>(CO_2Me)), 33.4 (<u>C</u>$H_2CH_2CH_3$), 30.4 (C<u>H</u>CO_2Me), 21.9 (CH_2<u>C</u>H_2CH_3), 13.8 (CH_2CH_2<u>C</u>H_3); **IR** (film) ν_{max}/cm^{-1}: 2957 (C-H), 1736 (C=O), 1436, 1283, 1250; **HRMS** (ES+) exact mass calculated for $[M+H]^+$ ($C_{13}H_{19}O_6^+$) requires *m/z* 271.1176, found *m/z* 271.1177; $[\alpha]_D^{25}$ = –129.1 (*c* 0.54, $CHCl_3$).

Synthesis and characterisation of 11b

2-isobutyl 1,1-dimethyl (R,Z)-3-butylidenecyclopropane-1,1,2-tricarboxylate (11b)

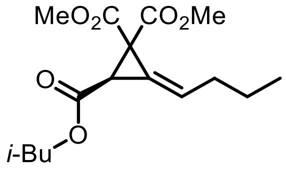

The title compound was prepared according to general procedure **II** from 2-isobutyl 1,1-dimethyl 3-butylcycloprop-2-ene-1,1,2-tricarboxylate (**11a**) (31.2 mg, 0.100 mmol, 1.00 eq) with catalyst **C11**. After 3 h the reaction mixture was quenched with AcOH to give the crude product in >20:1 d.r. The crude reaction mixture was purified by silica gel column chromatography (pentane/Et_2O = 8/2) to provide the title compound (**11b**) as a colourless oil in 96% yield (29.8 mg) and 94% ee. [determined by HPLC chiralpak AS-H, hexane/isopropanol = 99/1, 1 ml/min, λ = 220 nm, t(minor) = 15.56 min, t(major) = 23.75 min]. **^{1}H NMR** (500 MHz, $CDCl_3$) δ(ppm): 6.09 (td, *J* = 7.5, 2.0 Hz, 1H, C=C$\underline{H}$), 3.92 (dd, *J* = 10.5, 7.0 Hz, 1H, one of OC$\underline{H_A}$$H_B$), 3.81 (dd, *J* = 10.5, 6.5 Hz, 1H, one of OC$H_A$$\underline{H_B}$), 3.76 (s, 3H, one of $C(CO_2C\underline{H_3})_A(CO_2CH_3)_B$), 3.75 (s, 3H, one of $C(CO_2CH_3)_A(CO_2C\underline{H_3})_B$), 3.26 (q, *J* = 2.0 Hz, 1H, C$\underline{H}$C(=O)), 2.40 – 2.25 (m, 2H, $C\underline{H_2}CH_2CH_3$), 1.91 (dp, *J* = 13.5, 6.5 Hz, 1H, $C\underline{H}(CH_3)_2$), 1.50 (h, *J* = 7.5 Hz, 2H, $CH_2C\underline{H_2}CH_3$), 0.94 – 0.87 (m, 9H, $CH_2CH_2C\underline{H_3}$ and $CH(C\underline{H_3})_2$); **^{13}C NMR** (126 MHz, $CDCl_3$) δ(ppm): 167.9 ($\underline{C}O_2$*i*-Bu), 167.6 (one of $C(\underline{C}O_2CH_3)_A(CO_2CH_3)_B$), 165.4 (one of $C(CO_2CH_3)_A(\underline{C}O_2CH_3)_B$), 124.5 (C=$\underline{C}$H), 120.0 ($\underline{C}$=CH), 71.7 ($CO_2\underline{C}H_2$), 53.4 (one of $C(CO_2\underline{C}H_3)_A(CO_2CH_3)_B$), 53.0 (one of $C(CO_2CH_3)_A(CO_2\underline{C}H_3)_B$), 38.3 ($\underline{C}(CO_2Me)$), 33.4 ($\underline{C}H_2CH_2CH_3$), 30.6 (C$\underline{H}$$CO_2$*i*-Bu), 27.8 ($\underline{C}H(CH_3)_2$), 22.0 ($CH_2\underline{C}H_2CH_3$), 19.1 ($CH(\underline{C}H_3)_2$), 13.8 ($CH_2CH_2\underline{C}H_3$); **IR** (film) v_{max}/cm^{-1}: 2960, 2875 (C-H), 1735 (C=O), 1435, 1281, 1248; **HRMS** (ES+) exact mass calculated for $[M+H]^+$ ($C_{16}H_{25}O_6^+$) requires *m/z* 313.1646, found *m/z* 313.1645; $[\alpha]_D^{25}$ = –121.7 (*c* 0.53, $CHCl_3$).

Synthesis and characterisation of 12b

2-benzyl 1,1-dimethyl (R,Z)-3-(3-chloropropylidene)cyclopropane-1,1,2-tricarboxylate (12b)

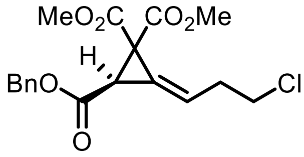

The title compound was prepared according to general procedure **II** from 2-benzyl 1,1-dimethyl 3-(3-chloropropyl)cycloprop-2-ene-1,1,2-tricarboxylate (**12a**) (18.3 mg, 0.0500 mmol, 1.00 eq) in Et_2O (0.5 mL) with dropwise addition of catalyst **C11** in Et_2O (0.5 mL) at -78 ^oC under Ar. After 1 h the reaction was quenched with was quenched with AcOH to give the crude product in >20:1 d.r. The crude reaction mixture was purified by silica gel column chromatography (pentane/Et_2O = 5/1) to provide the title compound (**12b**) as a colourless oil in 96% yield (18.0 mg) and 92% ee. [determined by HPLC chiralcel OD, hexane/isopropanol = 95/5, 1 ml/min, λ = 220 nm, t(major) = 24.89 min, t(minor) = 16.05 min]. **^{1}H NMR** (400 MHz, $CDCl_3$) δ(ppm): 7.40 – 7.30 (m, 5H, Ar$\underline{H}$), 6.18 (td, *J* = 7.0, 2.2 Hz, 1H, $CH_2C\underline{H}$=C), 5.21 – 5.05 (m, 2H, $C\underline{H_2}Ph$), 3.76 (s, 3H, one of $(CO_2C\underline{H_3})_A(CO_2CH_3)_B$), 3.73 – 3.62 (m, 2H, $CHCH_2\underline{CH_2}Cl$), 3.61 (s, 3H, one of $(CO_2CH_3)_A(CO_2C\underline{H_3})_B$), 3.36 (q, *J* = 1.9 Hz, 1H, $C\underline{H}CO_2Bn$), 2.90 – 2.73 (m, 2H, $CHC\underline{H_2}CH_2Cl$); **^{13}C NMR** (101 MHz, $CDCl_3$) δ(ppm): 167.3 ($\underline{C}O_2Bn$), 166.8 (one of $(\underline{C}O_2CH_3)_A(CO_2CH_3)_B$), 164.9 (one of $(CO_2CH_3)_A(\underline{C}O_2CH_3)_B$), 135.3 (Ar$\underline{C}$), 128.7 (Ar$\underline{C}$H), 128.6 (Ar$\underline{C}$H), 128.6 (Ar$\underline{C}$H), 122.4 ($CH_2\underline{C}H$=C), 120.4 ($CH_2CH$=$\underline{C}$), 67.5 ($\underline{C}H_2Ph$), 53.5 (one of $(CO_2\underline{C}H_3)_A(CO_2CH_3)_B$), 53.1(one of $(CO_2CH_3)_A(CO_2\underline{C}H_3)_B$), 43.2 ($Cl\underline{C}H_2$), 38.5 ($\underline{C}(CO_2Me)_2$), 34.5 ($\underline{C}H_2CH$=C), 30.4 ($\underline{C}HCO_2Bn$); **HRMS** (ES+) exact mass calculated for $[M+Na]^+$ ($C_{18}H_{19}ClO_6Na^+$) requires *m/z* 389.0762, found *m/z* 389.0762; $[\alpha]_D^{20}$ = –74.4 (*c* 0.87, $CHCl_3$).

Synthesis and characterisation of 13b

2-benzyl 1,1-dimethyl (R,Z)-3-(3-((tert-butyldimethylsilyl)oxy)propylidene)cyclopropane-1,1,2-tricarboxylate (13b)

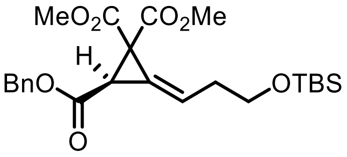

The title compound was prepared according to general procedure **II** from 2-benzyl 1,1-dimethyl 3-(3-((tert-butyldimethylsilyl)oxy)propyl)cycloprop-2-ene-1,1,2-tricarboxylate (**13a**) (23.1 mg, 0.0500 mmol, 1.00 eq) with catalyst **C11**. After 1 h the reaction was quenched with was quenched with AcOH to give the crude product >20:1 d.r. The crude reaction mixture was purified by silica gel column chromatography (pentane/EtOAc = 10/1) to provide the title compound (**13b**) as a colourless oil in 96% yield (22.2 mg) and 92% ee. [determined by HPLC chiralcel OD, hexane/isopropanol = 99/1, 1 ml/min, λ = 220 nm, t(major) = 26.22 min, t(minor) = 17.39 min]. **^{1}H NMR** (400 MHz, $CDCl_3$) δ(ppm): 7.39 – 7.29 (m, 5H, Ar$\underline{H}$), 6.18 (td, *J* = 7.2, 2.2 Hz, 1H, $CH_2C\underline{H}=C$), 5.12 (s, 2H, $PhC\underline{H_2}$), 3.75 (s, 3H, one of $(CO_2C\underline{H_3})_A(CO_2CH_3)_B$), 3.74 – 3.68 (m, 2H, $C\underline{H_2}OTBS$), 3.61 (s, 3H, one of $Si(C\underline{H_3})_A(CH_3)_BC(CH_3)_3$), 3.31 (q, *J* = 1.9 Hz, 1H, $C\underline{H}CO_2Bn$), 2.63 – 2.48 (m, 2H, $CHC\underline{H_2}$), 0.89 (s, 9H, $Si(CH_3)_2C(C\underline{H_3})_3$), 0.05 (s, 6H, $Si(C\underline{H_3})_2C(CH_3)_3$); **^{13}C NMR** (101 MHz, $CDCl_3$) δ(ppm): 167.6 ($\underline{C}O_2Bn$), 167.2 (one of $(\underline{C}O_2CH_3)_A(CO_2CH_3)_B$), 165.1(one of $(CO_2CH_3)_A(\underline{C}O_2CH_3)_B$), 135.4 (Ar$\underline{C}$), 128.7 (Ar$\underline{C}$), 128.6 (2 × Ar$\underline{C}$), 121.6 ($CH_2\underline{C}H=C$), 121.0 ($CH_2CH=\underline{C}$), 67.4 ($\underline{C}H_2Ph$), 62.2 ($TBSO\underline{C}H_2CH=C$), 53.4 (one of $(CO_2\underline{C}H_3)_A(CO_2CH_3)_B$), 53.0 (one of $(CO_2CH_3)_A(CO_2\underline{C}H_3)_B$), 38.4 ($\underline{C}(CO_2Me)_2$), 35.0 ($CH\underline{C}H_2$), 30.6 ($\underline{C}HCO_2Bn$), 26.0 ($Si(CH_3)_A(CH_3)_B\underline{C}(CH_3)_3$), 18.5 ($Si(CH_3)_A(CH_3)_BC(\underline{C}H_3)_3$), -5.2 ($Si(\underline{C}H_3)_2C(CH_3)_3$); **HRMS** (ES+) exact mass calculated for $[M+NH_4]^+$ ($C_{24}H_{38}NO_7Si^+$) requires *m/z* 480.2412, found *m/z* 480.2423; $[\alpha]_D^{20}$ = –74.3 (*c* 1.35, $CHCl_3$).

Synthesis and characterisation of 14b

2-benzyl 1,1-dimethyl (R,Z)-3-(3-phenylpropylidene)cyclopropane-1,1,2-tricarboxylate (14b)

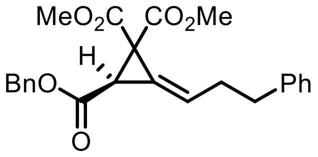

The title compound was prepared according to general procedure **II** from 2-benzyl 1,1-dimethyl 3-(3-phenylpropyl)cycloprop-2-ene-1,1,2-tricarboxylate (**14a**) (20.4 mg, 0.0500 mmol, 1.00 eq) with catalyst **C11**. After 0.5 h the reaction was quenched with was quenched with AcOH to give the crude product >20:1 d.r. The crude reaction mixture was purified by silica gel column chromatography (pentane/EtOAc = 10/1) to provide the title compound (**14b**) as a colourless oil in 99% yield (20.2 mg) and 92% ee. [determined by HPLC chiralpak AD-H, hexane/isopropanol = 95/5, 1 ml/min, λ = 220 nm, t(major) = 26.45 min, t(minor) = 20.30 min]. **^{1}H NMR** (400 MHz, $CDCl_3$) δ(ppm): 7.32 – 7.24 (m, 5H, Ar$\underline{H}$ of Bn), 7.23 – 7.17 (m, 2H, Ar$\underline{H}$), 7.14 – 7.07 (m, 3H, Ar$\underline{H}$), 6.06 (td, *J* = 7.1, 2.2 Hz, 1H, $CH_2C\underline{H}$=C), 5.24 – 4.85 (m, 2H, $C\underline{H_2}Ph$), 3.64 (s, 3H, one of $(CO_2C\underline{H_3})_A(CO_2CH_3)_B$), 3.54 (s, 3H, one of $(CO_2CH_3)_A(CO_2C\underline{H_3})$-$_B$), 3.23 (q, *J* = 1.9 Hz, 1H, $C\underline{H}CO_2Bn$), 2.78 – 2.68 (m, 2H, $CH_2C\underline{H_2}Ph$), 2.69 – 2.51 (m, 2H, $C\underline{H_2}CH_2Ph$); **^{13}C NMR** (126 MHz, $CDCl_3$) δ(ppm): 167.6 ($\underline{C}O_2Bn$), 167.2 (one of $(\underline{C}O_2CH_3)_A(CO_2CH_3)_B$), 165.2 (one of $(CO_2CH_3)_A(\underline{C}O_2CH_3)_B$), 141.5 (Ar$\underline{C}$), 135.4 (Ar$\underline{C}$ of Bn), 128.7 (Ar$\underline{C}$H), 128.6 (Ar$\underline{C}$H), 128.5 (Ar$\underline{C}$H), 126.1 (Ar$\underline{C}$H), 123.9 ($CH_2\underline{C}H$=C), 120.3 (119.8 (CH_2CH=$\underline{C}$), 67.4 ($\underline{C}H_2Ph$), 53.4 (one of $(CO_2\underline{C}H_3)_A(CO_2CH_3)_B$), 53.0 (one of $(CO_2CH_3)_A(CO_2\underline{C}H_3)_B$), 38.4 ($\underline{C}(CO_2Me)_2$), 35.0 ($CH_2\underline{C}H_2Ph$), 33.3 ($\underline{C}H_2CH$=C), 30.5 ($\underline{C}HCO_2Bn$); **HRMS** (ES+) exact mass calculated for $[M+H]^+$ ($C_{24}H_{25}O_6^+$) requires *m/z* 409.1646, found *m/z* 409.1640; $[\alpha]_D^{20}$ = –88.4 (*c* 1.34, $CHCl_3$).

Synthesis and characterisation of 15b

2-benzyl 1,1-dimethyl (R,Z)-3-ethylidenecyclopropane-1,1,2-tricarboxylate (15b)

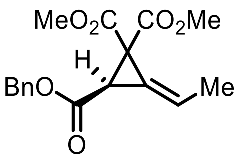

The title compound was prepared according to general procedure **II** from 2-benzyl 1,1-dimethyl 3-ethylcycloprop-2-ene-1,1,2-tricarboxylate (**15a**) (15.9 mg, 0.0500 mmol, 1.00 eq) with catalyst **C11**. After 0.5 h the reaction was quenched with was quenched with AcOH to give the crude product >20:1 d.r. The crude reaction mixture was purified by silica gel column chromatography (pentane/EtOAc = 4/1) to provide the title compound (**15b**) as a colourless oil in 99% yield (15.7 mg) and 90% ee. [determined by HPLC chiralcel OD, hexane/isopropanol = 95/5, 1 ml/min, λ = 220 nm, t(major) = 18.04 min, t(minor) = 11.43 min]. **^{1}H NMR** (400 MHz, $CDCl_3$) δ(ppm): 7.43 – 7.28 (m, 5H, ArH), 6.15 (qd, *J* = 6.8, 2.2 Hz, 1H, $CH_3C\underline{H}$=C), 5.13 (s, 2H, $C\underline{H_2}$Ph), 3.76 (s, 3H, one of $(CO_2C\underline{H_3})_A(CO_2CH_3)_B$), 3.63 (s, 3H, one of $(CO_2CH_3)_A(CO_2C\underline{H_3})_B$), 3.30 (p, *J* = 2.3 Hz, 1H, $C\underline{H}CO_2$Bn), 1.97 (dd, *J* = 6.8, 2.3 Hz, 3H, $C\underline{H_3}$CH=C); **^{13}C NMR** (101 MHz, $CDCl_3$) δ(ppm): 167.7 ($\underline{C}O_2$Bn), 167.4 (one of $(\underline{C}O_2CH_3)_A(CO_2CH_3)_B$), 165.3 (one of $(CO_2CH_3)_A(\underline{C}O_2CH_3)_B$), 135.4 (Ar$\underline{C}$), 128.7 (Ar$\underline{C}$H), 128.6 (Ar$\underline{C}$H), 128.6 (Ar$\underline{C}$H), 120.5 ($CH_3\underline{C}$H=C), 120.0 ($CH_3$CH=$\underline{C}$), 67.4 ($\underline{C}H_2$Ph), 53.4 (one of $(CO_2\underline{C}H_3)_A(CO_2CH_3)_B$), 53.0 (one of $(CO_2CH_3)_A(CO_2\underline{C}H_3)_B$), 38.3 ($\underline{C}(CO_2Me)_2$), 30.8 ($\underline{C}HCO_2$Bn), 16.7($\underline{C}H_3$CH=C); **HRMS** (ES+) exact mass calculated for $[M+H]^+$ ($C_{17}H_{19}O_6^+$) requires *m/z* 319.1176, found *m/z* 319.1171; $[\alpha]_D^{20}$ = –57.6 (*c* 0.39, $CHCl_3$).

Synthesis and characterisation of 16b

2-benzyl 1,1-dimethyl (R,Z)-3-(2-((tert-butyldimethylsilyl)oxy)ethylidene)cyclopropane-1,1,2-tricarboxylate (16b)

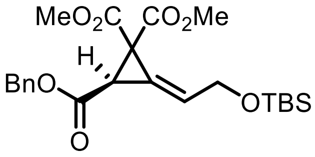

The title compound was prepared according to general procedure **II** from 2-benzyl 1,1-dimethyl 3-(2-((tert-butyldimethylsilyl)oxy)ethyl)cycloprop-2-ene-1,1,2-tricarboxylate (**16a**) (22.4 mg, 0.0500 mmol, 1.00 eq) with catalyst **C11**. After 1 h the reaction was quenched with was quenched with AcOH to give the crude product in 89:11 d.r. The crude reaction mixture was purified by silica gel column chromatography (pentane/EtOAc = 10/1) to provide the title compound (**16b**) as a colourless oil in 74% yield (16.7 mg) and 86% ee. [determined by HPLC chiralpak AD-H, hexane/isopropanol = 99/1, 1 ml/min, λ = 220 nm, t(major) = 13.61 min, t(minor) = 8.22 min]. **^{1}H NMR** (400 MHz, $CDCl_3$) δ(ppm): 7.40 – 7.30 (m, 5H, $Ar\underline{H}$), 6.21 (ddd, J = 6.9, 4.8, 2.2 Hz, 1H, $CH_2C\underline{H}$=C), 5.21 – 5.06 (m, 2H, $PhC\underline{H_2}$), 4.50 (ddd, J = 13.8, 4.8, 2.1 Hz, 1H, $C\underline{H_A}H_BOTBS$), 4.40 (ddd, J = 13.8, 6.7, 1.8 Hz, 1H, $CH_A\underline{H_B}OTBS$), 3.75 (s, 3H, one of $(CO_2C\underline{H_3})_A(CO_2CH_3)_B$), 3.60 (s, 3H, one of $(CO_2CH_3)_A(CO_2C\underline{H_3})_B$), 3.32 (q, J = 2.0 Hz, 1H, $C\underline{H}CO_2Bn$), 0.89 (s, 9H, $Si(CH_3)_A(CH_3)_BC(C\underline{H_3})_3$), 0.05 (s, 3H, one of $Si(C\underline{H_3})_A(CH_3)_BC(CH_3)_3$), 0.05 (s, 3H, one of $Si(CH_3)_A(C\underline{H_3})_BC(CH_3)_3$); **^{13}C NMR** (101 MHz, $CDCl_3$) δ(ppm): 167.4 ($\underline{C}O_2Bn$), 166.8 (one of $(\underline{C}O_2CH_3)_A(CO_2CH_3)_B$), 164.9 (one of $(CO_2CH_3)_A(\underline{C}O_2CH_3)_B$), 135.3 ($Ar\underline{C}$), 128.7 ($Ar\underline{C}$), 128.6 (2 × $Ar\underline{C}$), 124.1 ($CH_2\underline{C}H$=C), 119.4 (CH_2CH=$\underline{C}$), 67.5 ($\underline{C}H_2Ph$), 62.6 ($TBSO\underline{C}H_2CH$=C), 53.4 (one of $(CO_2\underline{C}H_3)_A(CO_2CH_3)_B$), 53.0 (one of $(CO_2CH_3)_A(CO_2\underline{C}H_3)_B$), 38.4 ($\underline{C}(CO_2Me)_2$), 29.9 ($\underline{C}HCO_2Bn$), 26.0 ($Si(CH_3)_A(CH_3)_B\underline{C}(CH_3)_3$), 18.5 ($Si(CH_3)_A(CH_3)_BC(\underline{C}H_3)_3$), -5.2 (one of $Si(\underline{C}H_3)_A(CH_3)_BC(CH_3)_3$), -5.2 (one of $Si(CH_3)_A(\underline{C}H_3)_BC(CH_3)_3$); **HRMS** (ES+) exact mass

calculated for $[M+H]^+$ ($C_{23}H_{33}O_7Si^+$) requires *m/z* 449.1990, found *m/z* 449.1988; $[\boldsymbol{\alpha}]_{\boldsymbol{D}}^{\mathbf{20}}$ = –84.9 (*c* 0.71, $CHCl_3$).

Synthesis and characterisation of 17b

2-benzyl 1,1-dimethyl (R,Z)-3-(2-phenylethylidene)cyclopropane-1,1,2-tricarboxylate (17b)

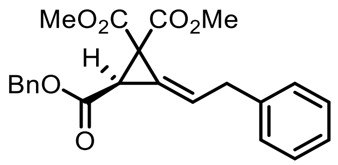

The title compound was prepared according to general procedure **II** from 2-benzyl 1,1-dimethyl 3-phenethylcycloprop-2-ene-1,1,2-tricarboxylate (**17a**) (19.7 mg, 0.0500 mmol, 1.00 eq) with catalyst **C11**. After 1 h the reaction was quenched with was quenched with AcOH to give the crude product in >20:1 d.r. The crude reaction mixture was purified by silica gel column chromatography (pentane/Et_2O = 3/1) to provide the title compound (**17b**) as a colourless oil in 85% yield (16.7 mg) and 92% ee. [determined by HPLC chiralcel OD, hexane/isopropanol = 95/5, 1 ml/min, λ = 220 nm, t(major) = 24.75 min, t(minor) = 17.08 min]. **^{1}H NMR** (400 MHz, $CDCl_3$) δ(ppm): 7.40 – 7.31 (m, 5H, ArH of Bn), 7.31 – 7.27 (m, 2H, ArH of Bn), 7.24 – 7.14 (m, 3H, ArH), 6.25 (td, *J* = 7.4, 2.1 Hz, 1H, $PhCH_2CH$), 5.20 – 5.02 (m, 2H, CH_2Ph), 3.74 (s, 3H, one of $(CO_2CH_3)_A(CO_2CH_3)_B$), 3.78 – 3.64 (m, 2H, $PhCH_2CH$), 3.61 (s, 3H, one of $(CO_2CH_3)_A(CO_2CH_3)_B$), 3.36 (q, *J* = 1.9 Hz, 1H, $CHCO_2Bn$); **^{13}C NMR** (101 MHz, $CDCl_3$) δ(ppm): 167.5(one of $(CO_2CH_3)_A(CO_2CH_3)_B$), 167.2(CO_2Bn), 165.0(one of $(CO_2CH_3)_A(CO_2CH_3)_B$), 139.0 (ArC), 135.3 (ArC), 128.7 (ArC), 128.7 (ArC), 128.6 (ArC), 128.6 (ArC), 126.5 (ArC), 123.2 (CH_2CH=C), 121.0 (CH_2CH=C), 67.5 (CO2CH_2Ph), 53.4 (one of $(CO_2CH_3)_A(CO_2CH_3)_B$), 53.0 (one of $(CO_2CH_3)_A(CO_2CH_3)_B$), 38.4 ($C(CO_2Me)_2$), 37.6 (CH_2CH=C), 30.8 ($CHCO_2Bn$); **HRMS** (ES+) exact mass calculated for $[M+H]^+$ ($C_{23}H_{23}O_6^+$) requires *m/z* 395.1489, found *m/z* 395.1499; $[\alpha]_D^{20}$ = –83.4 (*c* 0.98, $CHCl_3$).

Synthesis and characterisation of 18b

2-benzyl 1,1-diethyl (R,Z)-3-butylidenecyclopropane-1,1,2-tricarboxylate (18b)

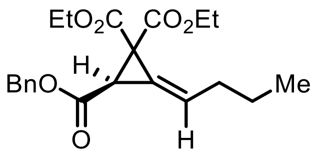

The title compound was prepared according to general procedure **II** from 2-benzyl 1,1-diethyl 3-butylcycloprop-2-ene-1,1,2-tricarboxylate (**18a**) (18.7 mg, 0.0500 mmol, 1.00 eq) with catalyst **C11**. After 1 h the reaction was quenched with was quenched with AcOH to give the crude product in >20:1 d.r. The crude reaction mixture was purified by silica gel column chromatography (pentane/Et_2O = 5/1) to provide the title compound (**18b**) as a colourless oil in 96% yield (18.0 mg) and 92% ee. [determined by HPLC chiralcel OD, hexane/isopropanol = 99/1, 1 ml/min, λ = 220 nm, t(major) = 20.00 min, t(minor) = 15.87 min]. **^{1}H NMR** (400 MHz, $CDCl_3$) δ(ppm): 7.40 – 7.29 (m, 5H, Ar<u>H</u>), 6.09 (td, *J* = 7.3, 2.2 Hz, 1H, , $CH_2C\underline{H}$=C), 5.18 – 5.07 (m, 2H, $C\underline{H_2}Ph$), 4.20 (q, *J* = 7.1 Hz, 2H, one of $(CO_2C\underline{H_2}CH_3)_A(CO_2CH_2CH_3)_B$), 4.09 (qd, *J* = 7.1, 1.1 Hz, 2H, one of $(CO_2CH_2CH_3)_A(CO_2C\underline{H_2}CH_3)_B$), 3.28 (q, *J* = 1.9 Hz, 1H, $C\underline{H}CO_2Bn$), 2.43 – 2.24 (m, 2H, $CHC\underline{H_2}$), 1.51 (h, *J* = 7.3 Hz, 2H, $CHCH_2C\underline{H_2}$), 1.25 (t, *J* = 7.1 Hz, 3H, $CH_2C\underline{H_3}$), 1.20 (t, *J* = 7.1 Hz, 3H, one of $(CO_2CH_2C\underline{H_3})_A(CO_2CH_2CH_3)_B$), 0.91 (t, *J* = 7.4 Hz, 3H, one of $(CO_2CH_2CH_3)_A(CO_2CH_2C\underline{H_3})_B$); **^{13}C NMR** (101 MHz, $CDCl_3$) δ(ppm): 167.8 ($C(\underline{C}O_2Et)_2$), 167.0 (one of $(\underline{C}O_2CH_2CH_3)_A(CO_2CH_2CH_3)_B$), 164.7(one of $(CO_2CH_3)_A(\underline{C}O_2CH_3)_B$), 135.5 (Ar<u>C</u>), 128.7 (Ar<u>C</u>H), 128.5 (Ar<u>C</u>H), 128.4 (Ar<u>C</u>H), 124.5 ($CH_2\underline{C}H$=C), 120.0 ($CH_2CH=\underline{C}$), 67.3 ($\underline{C}H_2Ph$), 62.4 (one of $(CO_2\underline{C}H_2CH_3)_A(CO_2CH_2CH_3)_B$), 61.9 (one of $(CO_2CH_2CH_3)_A(CO_2\underline{C}H_2CH_3)_B$), 38.9 ($\underline{C}(CO_2Me)_2$), 33.4 ($\underline{C}H_2CH$=C), 30.3 ($\underline{C}HCO_2Bn$), 22.0($CH_2\underline{C}H_2CH_3$), 14.1 (one of $(CO_2CH_2\underline{C}H_3)_A(CO_2CH_2CH_3)_B$), 14.0 (one of $(CO_2CH_2CH_3)_A(CO_2CH_2\underline{C}H_3)_B$), 13.8 ($CH_2CH_2\underline{C}H_3$); **HRMS** (ES+) exact mass calculated for $[M+H]^+$ ($C_{21}H_{27}O_6^+$) requires *m/z* 375.1802, found *m/z* 375.1800; $[\boldsymbol{\alpha}]_{\boldsymbol{D}}^{\mathbf{20}}$ = –109.2 (*c* 0.83, $CHCl_3$).

Synthesis and characterisation of 19b

2-benzyl 1,1-diisopropyl (R,Z)-3-butylidenecyclopropane-1,1,2-tricarboxylate (19b)

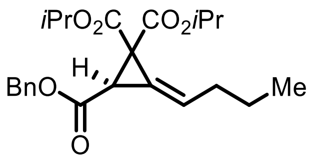

The title compound was prepared according to general procedure **II** from 2-benzyl 1,1-diisopropyl 3-butylcycloprop-2-ene-1,1,2-tricarboxylate (**19a**) (20.1 mg, 0.0500 mmol, 1.00 eq) with catalyst **C11**. After 1 h the reaction was quenched with was quenched with AcOH to give the crude product >20:1 d.r. The crude reaction mixture was purified by silica gel column chromatography (pentane/EtOAc = 10/1) to provide the title compound (**19b**) as a colourless oil in 99% yield (19.9 mg) and 90% ee. [determined by HPLC chiralpak AD-H, hexane/isopropanol = 98/2, 1 ml/min, λ = 220 nm, t(major) = 13.03 min, t(minor) = 10.03 min]. **^{1}H NMR** (400 MHz, $CDCl_3$) δ(ppm): 7.39 – 7.29 (m, 5H, Ar$\underline{H}$), 6.08 (td, *J* = 7.2, 2.1 Hz, 1H, $CH_2C\underline{H}$=C), 5.17 – 5.07 (m, 2H, $C\underline{H_2}$Ph), 5.02 (dq, *J* = 18.7, 6.2 Hz, 2H, $(C\underline{H}(CH_3)_2)_{\underline{2}}$), 3.25 (q, *J* = 1.9 Hz, 1H, $C\underline{H}CO_2Bn$), 2.43 – 2.20 (m, 2H, $CHC\underline{H_2}$), 1.57 – 1.45 (m, 2H, $CHCH_2C\underline{H_2}$), 1.24 (d, *J* = 6.3 Hz, 6H, one of $(CH(CH_{\underline{3}})_{\underline{2}})$, 1.21 (d, *J* = 6.3 Hz, 3H, one of $(CH(C\underline{H_3})_A(CH_3)_B)$, 1.19 (d, *J* = 6.3 Hz, 3H, $(CH(C\underline{H_3})_A(CH_3)_B)$), 0.91 (t, *J* = 7.4 Hz, 3H, $CH_2C\underline{H_3}$); **^{13}C NMR** (101 MHz, $CDCl_3$) δ(ppm): 167.9 ($\underline{C}O_2Bn$), 166.6 (one of $(\underline{C}O_2{}^iPr)_A(CO_2{}^iPr)_B$), 164.1(one of $(CO_2{}^iPr)_A(\underline{C}O_2{}^iPr)_B$), 135.6 (Ar$\underline{C}$), 128.7 (Ar$\underline{C}$H), 128.4 (Ar$\underline{C}$H), 128.4 (Ar$\underline{C}$H), 124.3 ($CH_2\underline{C}H$=C), 120.2 ($CH_2CH$=$\underline{C}$), 70.1 (one of $(\underline{C}H(CH_3)_2)_{\underline{A}B}$), 69.4(one of $(\underline{C}H(CH_3)_2)_{A\underline{B}}$), 67.1 ($\underline{C}H_2Ph$), 39.3 ($\underline{C}(CO_2Me)_2$), 33.4 ($\underline{C}H_2CH$=C), 30.0 ($\underline{C}HCO_2Bn$), 22.1 ($CH_2\underline{C}H_2CH_3$), 21.7 (one of $CH(\underline{C}H_3)_A(CH_3)_B$), 21.7 (one of $CH(CH_3)_A(\underline{C}H_3)_B$), 21.6 (one of $CH(\underline{C}H_3)_C(CH_3)_D$), 21.6 (one of $CH(CH_3)_C(\underline{C}H_3)_D$), 13.9 ($CH_2CH_2\underline{C}H_3$); **HRMS** (ES+) exact mass calculated for $[M+H]^+$ ($C_{23}H_{31}O_6{}^+$) requires *m/z* 403.2115, found *m/z* 403.2131; $[\boldsymbol{\alpha}]_{\boldsymbol{D}}^{\mathbf{20}}$ = –108.3 (*c* 0.33, $CHCl_3$).

Synthesis and characterisation of 20b

2-benzyl 1,1-di-tert-butyl (R,Z)-3-butylidenecyclopropane-1,1,2-tricarboxylate (20b)

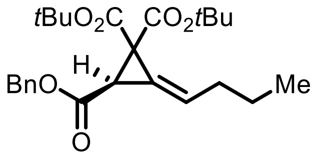

The title compound was prepared according to general procedure **II** from 2-benzyl 1,1-dimethyl 3-(3-oxo-3-phenylpropyl)cycloprop-2-ene-1,1,2-tricarboxylate (**20a**) (21.5 mg, 0.0500 mmol, 1.00 eq) with catalyst **C11**. After 12 h the reaction was quenched with was quenched with AcOH to give the crude product >20:1 d.r.. The crude reaction mixture was purified by silica gel column chromatography (pentane/EtOAc = 4/1) to provide the title compound (**20b**) as a colourless oil in 87% yield (18.7 mg) and 92% ee. [determined by SFC Chiralcel IC, 1500 psi, from 1% to 30% IPA in 5 min, 1.5 mL/min, t(major) = 3.06 min, t(minor) = 2.70 min]. **^{1}H NMR** (400 MHz, $CDCl_3$) δ(ppm): 7.50 – 7.28 (m, 5H, Ar$\underline{H}$), 6.04 (ddd, *J* = 7.6, 6.8, 2.1 Hz, 1H, $CH_2C\underline{H}{=}C$), 5.19 (d, *J* = 12.4 Hz, 1H, $C\underline{H_A}H_BPh$), 5.08 (d, *J* = 12.4 Hz, 1H, $CH_A\underline{H_B}Ph$), 3.16 – 3.14 (m, 1H, $C\underline{H}CO_2Bn$), 2.42 – 2.25 (m, 2H, $CHC\underline{H_2}$), 1.51 (q, *J* = 7.4 Hz, 2H, $CHCH_2C\underline{H_2}$), 1.46 (s, 9H, one of $C(CO_2C(C\underline{H_3)_3})_A(CO_2C(CH_3)_3)_B$), 1.43 (s, 9H, one of $C(CO_2C(C\underline{H_3)_3})_A(CO_2C(CH_3)_3)_B$), 0.92 (t, *J* = 7.4 Hz, 3H, $CH_2C\underline{H_3}$); **^{13}C NMR** (101 MHz, $CDCl_3$) δ(ppm): 168.2 ($\underline{C}O_2Bn$), 166.2 (one of $C(\underline{C}O_2{}^tBu)_A(CO_2{}^tBu)_B$), 163.6 (one of $C(\underline{C}O_2{}^tBu)_A(CO_2{}^tBu)_B$), 135.8 (Ar$\underline{C}$), 128.6 (Ar$\underline{C}$H), 128.4 (Ar$\underline{C}$H), 128.3 (Ar$\underline{C}$H), 123.6 ($CH_2\underline{C}H{=}C$), 120.7 ($CH_2CH{=}\underline{C}$), 83.0 (one of $C(CO_2\underline{C}(CH_3)_3)_A(CO_2C(CH_3)_3)_B$), 82.0 (one of $C(CO_2C(CH_3)_3)_A(CO_2\underline{C}(CH_3)_3)_B$), 67.0 ($\underline{C}H_2Ph$), 41.0 ($\underline{C}(CO_2{}^tBu)_A(CO_2{}^tBu)_B$), 33.4 ($\underline{C}H_2CH{=}C$), 29.7 ($\underline{C}HCO_2Bn$), 28.0 (one of $C(CO_2C(\underline{C}H_3)_3)_A(CO_2C(CH_3)_3)_B$), 27.9 (one of $C(CO_2C(\underline{C}H_3)_3)_A(CO_2C(C\underline{H_3)_3})_B$), 22.2 ($CH_2\underline{C}H_2CH_3$), 13.9 ($CH_2CH_2\underline{C}H_3$); **HRMS** (ES+) exact mass calculated for $[M+Na]^+$ ($C_{25}H_{34}O_6Na^+$) requires *m/z* 453.2248, found *m/z* 453.2260; $[\boldsymbol{\alpha}]_{\boldsymbol{D}}^{\mathbf{20}}$ = –99.9 (*c* 0.77, $CHCl_3$).

Synthesis and characterisation of 21b

tribenzyl (R,Z)-3-butylidenecyclopropane-1,1,2-tricarboxylate (21b)

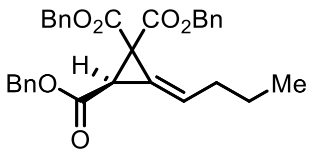

The title compound was prepared according to general procedure **II** from tribenzyl 3-butylcycloprop-2-ene-1,1,2-tricarboxylate (**21a**) (29.9 mg, 0.0500 mmol, 1.00 eq) with catalyst **C11**. After 1 h the reaction was quenched with was quenched with AcOH to give the crude product >20:1 d.r. The crude reaction mixture was purified by silica gel column chromatography (pentane/EtOAc = 10/1) to provide the title compound (**21b**) as a colourless oil in 91% yield (27.2 mg) and 92% ee. [determined by HPLC chiralpak IA, hexane/isopropanol = 98/2, 1 ml/min, λ = 220 nm, t(major) = 35.57 min, t(minor) = 33.77 min]. **^{1}H NMR** (400 MHz, $CDCl_3$) δ(ppm): 7.42 – 7.09 (m, 15H, Ar<u>H</u>), 6.10 (td, *J* = 7.2, 2.2 Hz, 1H, $CH_2C\underline{H}$=C), 5.20 – 5.13 (m, 2H, CH_2Ph), 5.08 – 5.00 (m, 4H, 2×CH_2Ph), 3.33 (q, *J* = 1.9 Hz, 1H, $C\underline{H}CO_2Bn$), 2.34 – 2.15 (m, 2H, $CHC\underline{H_2}$), 1.44 – 1.32 (m, 2H, $CHCH_2C\underline{H_2}$), 0.78 (t, *J* = 7.4 Hz, 3H, $CH_2C\underline{H_3}$); **^{13}C NMR** (101 MHz, $CDCl_3$) δ(ppm): 167.6 ($\underline{C}O_2Bn$), 166.7 (one of $(\underline{C}O_2Bn)_A(CO_2Bn)_B$), 164.5 (one of $(CO_2Bn)_A(\underline{C}O_2Bn)_B$), 135.4 (Ar<u>C</u>), 135.4 (Ar<u>C</u>), 135.2 (Ar<u>C</u>), 128.7 (Ar<u>C</u>H), 128.7 (Ar<u>C</u>H), 128.5 (Ar<u>C</u>H), 128.5 (Ar<u>C</u>H), 128.5 (Ar<u>C</u>H), 128.4 (Ar<u>C</u>H), 128.3 (Ar<u>C</u>H), 128.1 (Ar<u>C</u>H), 124.9 ($CH_2\underline{C}H$=C), 119.7 ($CH_2CH=\underline{C}$), 67.9 ($\underline{C}H_2Ph$), 67.7 ($\underline{C}H_2Ph$), 67.3 ($\underline{C}H_2Ph$), 38.7 ($\underline{C}(CO_2Bn)_2$), 33.5 ($\underline{C}H_2CH$=C), 30.5 ($\underline{C}HCO_2Bn$), 21.9 ($CH_2\underline{C}H_2CH_3$), 13.7 ($CH_2CH_2\underline{C}H_3$); **HRMS** (ES+) exact mass calculated for $[M+H]^+$ ($C_{31}H_{31}O_6^+$) requires *m/z* 499.2115, found *m/z* 499.2115; $[\alpha]_D^{20}$ = –82.2 (*c* 0.89, $CHCl_3$).

Synthesis and characterisation of 22b

benzyl (S)-2,2-dimethyl-3-methylenecyclopropane-1-carboxylate (22b)

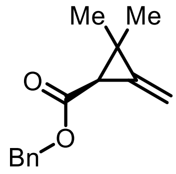

The title compound was prepared according to general procedure **II** from benzyl 2,3,3-trimethylcycloprop-1-ene-1-carboxylate (**22a**) (21.6 mg, 0.100 mmol, 1.00 eq) with catalyst **C11**. After 24 h the reaction was quenched, and the crude reaction mixture was purified by silica gel column chromatography (pntane/Et_2O = 99/1) to provide the title compound (**22b**) as a colourless oil in 97% yield (20.9 mg) and 90% ee. [determined by HPLC chiralpak IA, hexane, 1 ml/min, λ = 220 nm, t(minor) = 19.08 min, t(major) = 20.18 min]. **^{1}H NMR** (400 MHz, $CDCl_3$) δ(ppm): 7.41 – 7.30 (m, 5H, ArH), 5.45 (d, *J* = 2.3 Hz, 1H, one of C=CH_AH_B), 5.41 (d, *J* = 1.7 Hz, , one of C=CH_AH_B), 5.15 (d, *J* = 12.4 Hz, 1H, one of CH_AH_BPh), 5.11 (d, *J* = 12.4 Hz, 1H, one of CH_AH_BPh), 2.12 (dd, *J* = 2.3, 1.7 Hz, 1H, CH(C=O)), 1.32 (s, 3H, one of C$(CH_3)_A(CH_3)_B$), 1.30 (s, 3H, one of C$(CH_3)_A(CH_3)_B$); **^{13}C NMR** (101 MHz, $CDCl_3$) δ(ppm): 170.8 (C=O), 141.4 (C=CH_2), 136.3 (ArC), 128.7 (ArCH), 128.4 (ArCH), 128.3 (ArCH), 103.3 (C=CH_2), 66.4 (CH_2O), 30.1 (CHC(=O)), 26.9 (CMe_2), 26.0 (one of C$(CH_3)_A(CH_3)_B$), 18.3 (one of C$(CH_3)_A(CH_3)_B$); **IR** (film) ν_{max}/cm^{-1}: 2955, 2927 (C-H), 1729 (C=O), 1331, 1150, 900 (C=C); **HRMS** (ES+) exact mass calculated for $[M+H]^+$ ($C_{14}H_{17}O_2^+$) requires *m/z* 217.1223, found *m/z* 217.1223; $[\alpha]_D^{25}$ = –62.0 (*c* 0.25, $CHCl_3$).

Synthesis and characterisation of 23b

2-benzyl 1,1-dimethyl (2R,3R)-3-((E)-3-oxo-3-phenylprop-1-en-1-yl)cyclopropane-1,1,2-tricarboxylate (23b)

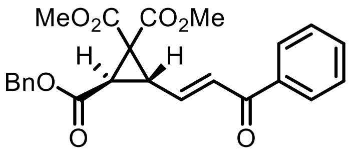

The title compound was prepared according to general procedure **II** from 2-benzyl 1,1-dimethyl 3-(3-oxo-3-phenylpropyl)cycloprop-2-ene-1,1,2-tricarboxylate (**23a**) (20.4 mg, 0.0500 mmol, 1.00 eq) with catalyst **C11**. After 12 h the reaction was quenched with was quenched with AcOH to give the crude product >20:1 d.r.. The crude reaction mixture was purified by silica gel column chromatography (pentane/EtOAc = 4/1) to provide the title compound (**23b**) as a colourless oil in 99% yield (20.2 mg) and 98% ee. [determined by HPLC chiralcel OD, hexane/isopropanol = 95/5, 1 ml/min, λ = 220 nm, t(major) = 12.93 min, t(minor) = 17.93 min]. **^{1}H NMR** (500 MHz, $CDCl_3$) δ(ppm): 7.97 – 7.85 (m, 2H, ArH of C(O)Ph), 7.61 – 7.53 (m, 1H, ArH of C(O)Ph), 7.51 – 7.44 (m, 2H, ArH of C(O)Ph), 7.42 – 7.31 (m, 5H, ArH of Bn), 7.25 (d, *J* = 15.0 Hz, 1H, C(O)CH=CH), 6.74 (dd, *J* = 15.3, 9.7 Hz, 1H, C(O)CH=CH), 5.23 – 5.08 (m, 2H, $PhCH_2$), 3.77 (s, 3H, one of $C(CO_2CH_3)_A(CO_2CH_3)_B$), 3.67 (s, 3H, one of $C(CO_2CH_3)_A(CO_2CH_3)_B$), 3.21 (dd, *J* = 9.7, 6.8 Hz, 1H, $CHCHCO_2Bn$), 3.08 (d, *J* = 6.9 Hz, 1H, $CHCO_2Bn$); **^{13}C NMR** (126 MHz, $CDCl_3$) δ(ppm): 189.3 (PhC(O)), 168.2 (CO_2Bn), 166.0 (one of $(CO_2CH_3)_A(CO_2CH_3)_B$), 165.6 (one of $(CO_2CH_3)_A(CO_2CH_3)_B$), 139.9 (C(O)CH=CH), 137.4 (ArC), 135.2 (ArC), 133.2, 130.3, 128.8, 128.8, 128.8, 128.7, 128.7, 67.8 (CH_2Ph), 53.6 (one of $C(CO_2CH_3)_A(CO_2CH_3)_B$), 53.3 (one of $C(CO_2CH_3)_A(CO_2CH_3)_B$), 43.7, 34.4, 33.6 ($CHCO_2Bn$); **HRMS** (ES+) exact mass calculated for $[M+H]^+$ ($C_{24}H_{20}O_7Na^+$) requires *m/z* 445.1258, found *m/z* 445.1272; $[\alpha]_D^{20}$ = –17.3(*c* 0.35, $CHCl_3$).

Synthesis and characterisation of 24b

trimethyl (R,Z)-3-ethylidenecyclopropane-1,1,2-tricarboxylate (24b)

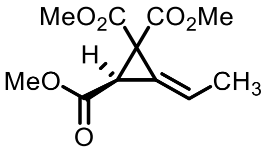

The title compound was prepared according to general procedure **II** from trimethyl 3-ethylcycloprop-2-ene-1,1,2-tricarboxylate (**24a**) (12.1 mg, 0.0500 mmol, 1.00 eq) with catalyst **C11**. After 0.5 h the reaction was quenched with was quenched with AcOH to give the crude product >20:1 d.r.. The crude reaction mixture was purified by silica gel column chromatography (CH_2Cl_2/CH_3CN = 20/1) to provide the title compound (**24b**) as a colourless oil in 85% yield (10.2 mg) and 90% ee. [determined by HPLC chiralpak IB, hexane/isopropanol = 99.5/0.5-70/30, 1 ml/min, λ = 220 nm, t(major) = 18.60 min, t(minor) = 15.96 min]. **^{1}H NMR** (400 MHz, $CDCl_3$) δ(ppm): 6.14 (qd, *J* = 6.8, 2.2 Hz, 1H, $CH_3C\underline{H}$=C), 3.77 (s, 6H, one of $(CO_2C\underline{H_3})_A(CO_2CH_3)_B$ and $CHCO_2C\underline{H_3}$), 3.69 (s, 3H, one of $(CO_2CH_3)_A(CO_2C\underline{H_3})_B$), 3.24 (p, *J* = 2.2 Hz, 1H, $C\underline{H}CO_2CH_3$), 1.96 (dd, *J* = 6.8, 2.3 Hz, 3H, $C\underline{H_3}CH$=C); **^{13}C NMR** (101 MHz, $CDCl_3$) δ(ppm): 168.3 ($\underline{C}O_2CH_3$), 167.4 (one of $(\underline{C}O_2CH_3)_A(CO_2CH_3)_B$), 165.5 (one of $(CO_2CH_3)_A(\underline{C}O_2CH_3)_B$), 120.4 ($CH_3\underline{C}H$=C), 120.0 ($CH_3CH$=$\underline{C}$), 53.4 (one of $(CO_2\underline{C}H_3)_A(CO_2CH_3)_B$), 53.1 (one of $(CO_2CH_3)_A(CO_2\underline{C}H_3)_B$), 52.7 ($CHCO_2\underline{C}H_3$), 38.2 ($\underline{C}(CO_2Me)_2$), 30.7 ($CHCO_2CH_3$), 16.7 ($\underline{C}H_3CH$=C); **HRMS** (ES+) exact mass calculated for $[M+H]^+$ ($C_{11}H_{15}O_6^+$) requires *m/z* 265.0683, found *m/z* 265.0670; $[\boldsymbol{\alpha}]_D^{20}$ = –105.4 (*c* 0.43, $CHCl_3$).

Synthesis and characterisation of 38b

benzyl (S,Z)-2-propylidenespiro[2.5]octane-1-carboxylate (38b)

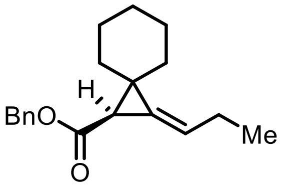

The title compound was prepared according to general procedure **II** from benzyl 2-propylspiro[2.5]oct-1-ene-1-carboxylate (**38a**) (14.2 mg, 0.0500 mmol, 1.00 eq) with catalyst **C13**. After 2 h the reaction was quenched with was quenched with AcOH to give the crude product >20:1 d.r. The crude reaction mixture was purified by silica gel column chromatography (pentane/EtOAc = 99/1) to provide the title compound (**38b**) as a colourless oil in 93% yield (13.2 mg) and 99% ee. [determined by SFC Chiralcel IG, 1500 psi, from 1% to 30% IPA in 5 min, 1.5 mL/min, t(major) = 2.60 min, t(minor) = 3.08 min]. **^{1}H NMR** (400 MHz, $CDCl_3$) δ(ppm)7.41 – 7.29 (m, 5H, Ar$\underline{H}$), 5.81 (td, *J* = 6.4, 1.7 Hz, 1H, $CH_2C\underline{H}$=C), 5.22 – 5.02 (m, 2H, $PhC\underline{H_2}$), 2.22 (qdd, *J* = 7.5, 6.3, 1.7 Hz, 2H, $CHC\underline{H_2}$), 2.01 (q, *J* = 1.7 Hz, 1H, $C\underline{H}CO_2Bn$), 1.84 – 1.75 (m, 1H, one of $C(C\underline{H_A}H_B)(CH_CH_D)$), 1.74 – 1.68 (m, 1H, one of $C(CH_AH_B)(C\underline{H_C}H_D)$), 1.68 – 1.64 (m, 1H, one of $C(CH_ACH_BC\underline{H_C}CH_D)$ $(CH_ECH_FCH_GCH_H)$), 1.65 – 1.51 (m, 4H, $CH_AH_BC\underline{H_C}H_DC\underline{H_E}H_FC\underline{H_G}H_HC\underline{H_I}H_J$), 1.46 – 1.22 (m, 3H, $CH_AH_BCH_C\underline{H_D}CH_E\underline{H_F}CH_G\underline{H_H}CH_IH_J$), 1.05 (t, *J* = 7.5 Hz, 3H, $CH_2C\underline{H_3}$); **^{13}C NMR** (101 MHz, $CDCl_3$) δ(ppm)171.6($\underline{C}O_2Bn$), 136.5 (Ar$\underline{C}$), 130.8 (Ar$\underline{C}$), 128.6 (Ar$\underline{C}$), 128.3 (Ar$\underline{C}$), 128.2 ($CH_2CH=\underline{C}$), 121.7($CH_2\underline{C}H=C$), 66.2 ($\underline{C}H_2Ph$), 36.8 ($\underline{C}(CH_2)_2$), 34.7($\underline{C}H_2CH=C$), 29.2 ($\underline{C}HCO_2Bn$), 28.7 ($\underline{C}H_2CH_2CH_2CH_2CH_2$), 26.2 ($CH_2CH_2CH_2CH_2\underline{C}H_2$), 25.8 ($CH_2\underline{C}H_2CH_2CH_2CH_2$), 25.5 ($CH_2CH_2CH_2\underline{C}H_2CH_2$), 24.9 ($CH_2CH_2\underline{C}H_2CH_2CH_2$), 14.0 ($CH_2\underline{C}H_3$); **HRMS** (ES+) exact mass calculated for $[M+H]^+$ ($C_{19}H_{25}O_2^+$) requires *m/z* 285.1849, found *m/z* 285.1841; $[\alpha]_D^{20}$ = –98.7 (*c* 0.35, $CHCl_3$).

6.2 Synthesis of enantioenriched cyclopropane amides and phosphine oxides

Synthesis and characterisation of 25b

dimethyl (R,Z)-2-(tert-butylcarbamoyl)-3-butylidenecyclopropane-1,1-dicarboxylate (25b)

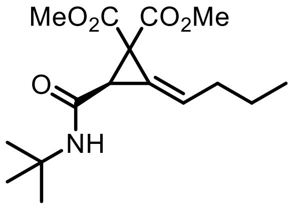

The title compound was prepared according to general procedure **II** from dimethyl 2-butyl-3-(*tert*-butylcarbamoyl)cycloprop-2-ene-1,1-dicarboxylate (**25a**) (31.1 mg, 1.00 mmol, 1.00 eq) with catalyst **C11**. After 45 min the reaction mixture was quenched with AcOH to give the crude product in >20:1 d.r. The crude reaction mixture was purified by silica gel column chromatography (pentane/Et_2O = 7/3 to 6/4) to provide the title compound (**25b**) as a yellow oil in >99% yield (31.0 mg) and 98% ee. [determined by HPLC chiralpak AD, hexane/isopropanol = 99.5/0.5-70/30, 1 ml/min, λ = 220 nm, t(major) = 22.42 min, t(minor) = 25.22 min]. **^{1}H NMR** (500 MHz, $CDCl_3$) δ(ppm): 6.11 (td, *J* = 7.2, 2.2 Hz, 1H, C=C<u>H</u>), 5.47 (s, 1H, N<u>H</u>), 3.75 (s, 3H, one of $C(CO_2C\underline{H_3})_A(CO_2CH_3)_B$), 3.74 (s, 3H, one of $C(CO_2CH_3)_A(CO_2C\underline{H_3})_B$), 3.11 (q, *J* = 1.9 Hz, 1H, C<u>H</u>CONH), 2.40 – 2.24 (m, 2H, $C\underline{H_2}CH_2CH_3$), 1.55 – 1.46 (m, 2H, $CH_2C\underline{H_2}CH_3$), 1.30 (s, 9H, $C(C\underline{H_3})_3$), 0.90 (t, *J* = 7.4 Hz, 3H, $CH_2CH_2C\underline{H_3}$); **^{13}C NMR** (126 MHz, $CDCl_3$) δ(ppm): 167.8 (one of $\underline{C}O_2Me$), 165.9 (one of $\underline{C}O_2Me$), 164.6 (<u>C</u>ONH), 124.9 (C=<u>C</u>H), 120.6 (<u>C</u>=CH), 53.2 (one of $C(CO_2\underline{C}H_3)_A(CO_2CH_3)_B$), 53.0 (one of $C(CO_2CH_3)_A(CO_2\underline{C}H_3)_B$), 51.7 ($N\underline{C}(CH_3)_3$), 37.9 ($\underline{C}(CO_2Me)$), 33.8 (<u>C</u>HCONH), 33.5 ($\underline{C}H_2CH_2CH_3$), 28.7 ($NC(\underline{C}H_3)_3$), 22.0 ($CH_2\underline{C}H_2CH_3$), 13.8 ($CH_2CH_2\underline{C}H_3$); **IR** (film) ν_{max}/cm^{-1}: 3314 (N-H), 2969 (C-H), 1734 (C=O ester), 1656 (C=O amide); **HRMS** (ES+) exact mass calculated for $[M+H]^+$ ($C_{16}H_{26}NO_5^+$) requires *m/z* 312.1805, found *m/z* 312.1805; $[\alpha]_D^{26.1}$ = –75.5 (*c* 0.48, $CHCl_3$).

NOESY of compound **25b**: Cross-peaks are observed between $N\underline{H}$ and $C{=}C\underline{H}$.

Synthesis and characterisation of 26b

dimethyl (R,Z)-2-(allylcarbamoyl)-3-butylidenecyclopropane-1,1-dicarboxylate (26b)

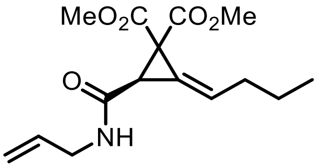

The title compound was prepared according to general procedure **II** from **26a** (14.8 mg, 0.0500 mmol, 1.00 eq) with catalyst **C11**. After 2 h the reaction mixture was quenched with AcOH to give the crude product in >20:1 d.r. The crude reaction mixture was purified by silica gel column chromatography (pentane/EtOAc = 7/3) to provide the title compound (**26b**) as a yellow oil in 73% yield (10.8 mg) and 97% ee. [determined by HPLC chiralpak AS-H, hexane/isopropanol = 90/10 to 70/30, 1 ml/min, λ = 220 nm, t(minor) = 11.67 min, t(major) = 27.68 min]. **^{1}H NMR** (500 MHz, $CDCl_3$) δ(ppm): 6.13 (td, J = 7.0, 2.0 Hz, 1H, $C{=}C\underline{H}CH_2CH_2$), 5.79 (ddt, J = 17.0, 10.0, 6.0 Hz, 1H, $C\underline{H}{=}CH_2$), 5.72 (bs, 1H, $N\underline{H}$), 5.22 – 5.07 (m, 2H, $CH{=}C\underline{H_2}$), 3.85 (tq, J = 5.5, 1.5 Hz, 2H, $NHC\underline{H_2}$), 3.76 (s, 3H, one of $C(CO_2C\underline{H_3})_A(CO_2CH_3)_B$), 3.75 (s, 3H, one of $C(CO_2CH_3)_A(CO_2C\underline{H_3})_B$), 3.22 (q, J = 2.0 Hz, 1H, $C\underline{H}C({=}O)$), 2.41 – 2.21 (m, 2H, $C\underline{H_2}CH_2CH_3$), 1.50 (hd, J = 7.5, 2.0 Hz, 2H, $CH_2C\underline{H_2}CH_3$), 0.90 (t, J = 7.5 Hz, 3H, $CH_2CH_2C\underline{H_3}$); **^{13}C NMR** (126 MHz, $CDCl_3$) δ(ppm): 167.7 (one of $C(\underline{C}O_2CH_3)_A(CO_2CH_3)_B$), 166.0 (one of $C(CO_2CH_3)_A(\underline{C}O_2CH_3)_B$), 165.6 ($\underline{C}({=}O)NH$), 133.8 ($\underline{C}H{=}CH_2$), 125.1 ($C{=}\underline{C}HCH_2CH_2$), 120.4 ($\underline{C}{=}CHCH_2CH_2$), 117.0 ($CH{=}\underline{C}H_2$), 53.3 (one of $C(CO_2\underline{C}H_3)_A(CO_2CH_3)_B$), 53.2 (one of $C(CO_2CH_3)_A(CO_2\underline{C}H_3)_B$), 42.3 ($NH\underline{C}H_2$), 38.1 ($\underline{C}(CO_2Me)_2$), 33.5 ($\underline{C}H_2CH_2CH_3$), 32.8 ($\underline{C}HC({=}O)NH$), 22.0 ($CH_2\underline{C}H_2CH_3$), 13.8 ($CH_2CH_2\underline{C}H_3$); **IR** (film) v_{max}/cm^{-1}: 3307 (N-H), 2957, 2932 (C-H), 1736 (C=O ester), 1655 (C=O amide), 1543, 1281, 1254; **HRMS** (ES+) exact mass calculated for $[M+Na]^+$ ($C_{15}H_{21}NNaO_5^+$) requires m/z 318.1312, found m/z 318.1313; $[\alpha]_D^{25}$ = –92.1 (c 0.33, $CHCl_3$).

NOESY of compound **12b**: Cross-peaks are observed between N<u>H</u> and C=C<u>H</u>$CH_2CH_2CH_3$.

Synthesis and characterisation of 27b

<u>dimethyl (R)-2-(tert-butylcarbamoyl)-3-cyclopentylidenecyclopropane-1,1-dicarboxylate (27b)</u>

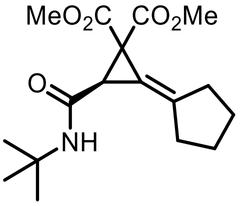

The title compound was prepared according to general procedure **II** from dimethyl 2-(*tert*-butylcarbamoyl)-3-cyclopentylcycloprop-2-ene-1,1-dicarboxylate (**27a**) (32.3 mg, 0.100 mmol, 1.00 eq) with catalyst **C11**. After being deemed complete by TLC (pentane/EtOAC = 7/3) the reaction was quenched and the crude reaction mixture was purified by silica gel column chromatography (pentane/Et_2O = 8/2 then pentane/EtOAc = 9/1) to provide the title compound (**27b**) as a white solid in 93% yield (30.1 mg) and 99% ee. [determined by HPLC chiralpak AD-H, hexane/isopropanol = 98/2, 1 ml/min, λ = 220 nm, t(major) = 31.20 min, t(minor) = 44.53 min]. **^{1}H NMR** (500 MHz, $CDCl_3$) δ(ppm): 5.41 (s, 1H, N<u>H</u>), 3.74 (s, 3H, one of $C(CO_2C\underline{H_3})_A(CO_2CH_3)_B$), 3.73 (s, 3H, one of $C(CO_2CH_3)_A(CO_2C\underline{H_3})_B$), 3.08 (p, *J* = 3.0 Hz, 1H, C<u>H</u>C(=O)), 2.74 – 2.63 (m, 1H, one of $C\underline{H_A}H_BCCH_CH_D$), 2.44 (m, two of one of $CH_A\underline{H_B}CC\underline{H_C}H_D$ 2H), 2.42 – 2.30 (m, 1H, one of one of $CH_AH_BCCH_C\underline{H_D}$), 1.84 – 1.69 (m, 4H, $C\underline{H_2}CH_2CCH_2C\underline{H_2}$), 1.29 (s, 9H, $C(C\underline{H_3})_3$); **^{13}C NMR** (126 MHz, $CDCl_3$) δ(ppm): 168.0 (one of $C(\underline{C}O_2CH_3)_A(CO_2CH_3)_B$), 166.5 (one of $C(CO_2CH_3)_A(\underline{C}O_2CH_3)_B$), 164.9 (<u>C</u>ONH), 140.4 ($CH_2\underline{C}$=C), 110.5 (CH_2C=<u>C</u>), 53.1 (one of $C(CO_2\underline{C}H_3)_A(CO_2CH_3)_B$), 52.9 (one of $C(CO_2CH_3)_A(CO_2\underline{C}H_3)_B$), 51.6 ($\underline{C}(CH_3)_3$), 38.5 ($\underline{C}(CO_2Me)_2$), 34.2 (<u>C</u>HC(=O)), 32.4 (one of $(\underline{C}H_2)_AC(CH_2)_B$), 32.3 (one of $(CH_2)_AC(\underline{C}H_2)_B$), 28.7 ($C(\underline{C}H_3)_3$), 26.8 (one of $(\underline{C}H_2)_ACH_2CCH_2(CH_2)_B$), 26.5 (one of $(CH_2)_ACH_2CCH_2(\underline{C}H_2)_B$); **IR** (film) ν_{max}/cm^{-1}: 3325 (N-H), 2956 (C-H), 1734 (C=O ester), 1680 (C=C), 1660 (C=O amide); **HRMS** (ES+) exact mass

calculated for $[M+H]^+$ ($C_{17}H_{26}O_5N^+$) requires *m/z* 324.1805, found *m/z* 324.1807; **MP**: 122 – 124 °C; $[\boldsymbol{\alpha}]_{\boldsymbol{D}}^{\boldsymbol{25}}$ = –42.4 (*c* 0.43, $CHCl_3$).

Synthesis and characterisation of 28b

(S)-N-(tert-butyl)-2,2-dimethyl-3-methylenecyclopropane-1-carboxamide (28b)

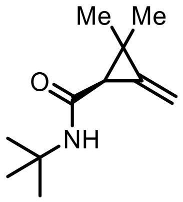

The title compound was prepared according to general procedure **II** from N-(*tert*-butyl)-2,3,3-trimethylcycloprop-1-ene-1-carboxamide (**28a**) (9.1 mg, 0.050 mmol, 1.00 eq) with catalyst **C11**. After 24 h the reaction was quenched and the crude reaction mixture was purified by silica gel column chromatography (pentane/Et_2O = 7/3) to provide the title compound (**28b**) as a white solid in 78% yield (7.1 mg) and 99% ee. [determined by HPLC chiralpak AS-H, hexane/isopropanol = 98/2, 1 ml/min, λ = 210 nm, t(minor) = 5.66 min, t(major) = 9.24 min]. **^{1}H NMR** (500 MHz, $CDCl_3$) δ(ppm): 5.47 (d, J = 2.0 Hz, 1H, one of C=C$\underline{H_A}$$H_B$), 5.45 (d, J = 2.0 Hz, 1H, one of C=C$H_A$$\underline{H_B}$), 5.40 (bs, 1H, N$\underline{H}$), 1.95 (t, J = 2.0 Hz, 1H, C$\underline{H}$C(=O)), 1.32 (s, 9H, C(C$\underline{H_3}$)$_3$), 1.26 (s, 3H, one of C(C$\underline{H_3}$)$_A$(CH_3)$_B$), 1.22 (s, 3H, one of C(CH_3)$_A$(C$\underline{H_3}$)$_B$); **^{13}C NMR** (126 MHz, $CDCl_3$) δ(ppm): 168.2 ($\underline{C}$=O), 143.3 ($\underline{C}$=CH_2), 103.5 (C=$\underline{C}H_2$), 51.2 ($\underline{C}$(CH_3)$_3$), 33.5 ($\underline{C}$HC(=O)), 28.9 (C($\underline{C}H_3$)$_3$), 25.9 (one of C($\underline{C}H_3$)$_A$(CH_3)$_B$), 24.0 ($\underline{C}$(CH_3)$_2$), 18.7 (one of C(CH_3)$_A$($\underline{C}H_3$)$_B$); **IR** (film) ν_{max}/cm^{-1}: 3287 (N-H), 2966, 2923 (C-H), 1632 (C=O), 1555; **HRMS** (ES+) exact mass calculated for $[M+H]^+$ ($C_{11}H_{20}NO^+$) requires *m/z* 182.1539, found *m/z* 182.1542; **MP**: 72 °C (sublimation); $[\boldsymbol{\alpha}]_D^{25}$ = +42.9 (*c* 0.12, $CHCl_3$).

Synthesis and characterisation of 29b

dimethyl (R,Z)-2-butylidene-3-((4-methoxyphenyl)carbamoyl)cyclopropane-1,1-dicarboxylate (29b)

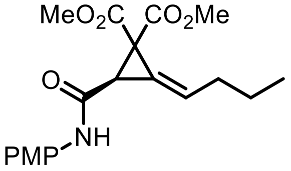

The title compound was prepared according to general procedure **II** from dimethyl 2-butyl-3-((4-methoxyphenyl)carbamoyl)cycloprop-2-ene-1,1-dicarboxylate (**29b**) (18.1 mg, 0.0500 mmol, 1.00 eq) with catalyst **C11** in THF (1 ml). After 1.75 h the reaction mixture was quenched with AcOH to give the crude product in >20:1 d.r. The crude reaction mixture was purified by silica gel column chromatography (pentane/EtOAc = 8/2 to 7/3) to provide the title compound (**29b**) as a colourless oil in >99% yield (18.1 mg) and 94% ee. [determined by HPLC chiralpak AD-H, hexane/isopropanol = 90/10, 1 ml/min, λ = 240 nm, t(minor) = 19.35 min, t(major) = 23.19 min]. **^{1}H NMR** (600 MHz, $CDCl_3$) δ(ppm): 7.38 – 7.33 (m, 2H, Ar$\underline{H}$), 7.31 (s, 1H, N$\underline{H}$), 6.87 – 6.81 (m, 2H, Ar$\underline{H}$), 6.23 (td, *J* = 7.0, 2.0 Hz, 1H, C=C$\underline{H}$$CH_2$), 3.78 (s, 3H, one of $C(CO_2C\underline{H_3})_A(CO_2CH_3)_B$), 3.78 (s, 3H, $C\underline{H_3}OAr$), 3.72 (s, 3H, one of $C(CO_2CH_3)_A(CO_2C\underline{H_3})_B$), 3.35 (q, *J* = 2.0 Hz, 1H, C$\underline{H}$C(=O)), 2.45 – 2.29 (m, 2H, $CH_2CH_2CH_3$), 1.62 – 1.51 (m, 2H, $CH_2C\underline{H_2}CH_3$), 0.94 (t, *J* = 7.5 Hz, 3H, $CH_2CH_2C\underline{H_3}$); **^{13}C NMR** (151 MHz, $CDCl_3$) δ(ppm): 167.4 (one of $C(\underline{C}O_2CH_3)_A(CO_2CH_3)_B$), 166.0 (one of $C(CO_2CH_3)_A(\underline{C}O_2CH_3)_B$), 163.7 ($\underline{C}$(=O)NH), 156.9 (Ar$\underline{C}$), 130.5 (Ar$\underline{C}$), 125.7 (C=$\underline{C}HCH_2$), 121.9 (Ar$\underline{C}$H), 120.4 ($\underline{C}$=CH$CH_2$), 114.3 (Ar$\underline{C}$H), 55.6 (ArO$\underline{C}H_3$), 53.4 (one of $C(CO_2\underline{C}H_3)_A(CO_2CH_3)_B$), 53.3 (one of $C(CO_2CH_3)_A(CO_2\underline{C}H_3)_B$), 38.4 ($\underline{C}(CO_2Me)_2$), 33.7 ($\underline{C}H_2CH_2CH_3$), 33.6 ($\underline{C}$HC(=O)), 22.0 ($CH_2\underline{C}H_2CH_3$), 13.8 ($CH_2CH_2\underline{C}H_3$); **IR** (film) ν_{max}/cm^{-1}: 3299 (N-H), 2958, 2839 (C-H), 1733, 1686 (C=O); **HRMS** (ES+) exact mass calculated for

$[M+H]^+$ ($C_{19}H_{24}O_6N^+$) requires *m/z* 362.1598, found *m/z* 362.1601; $[\boldsymbol{\alpha}]_{\boldsymbol{D}}^{\mathbf{26.1}}$ = –76.8 (*c* 0.38, $CHCl_3$).

Synthesis and characterisation of 29c

dimethyl (R,Z)-2-butylidene-3-((4-hydroxyphenyl)carbamoyl)cyclopropane-1,1-dicarboxylate (29c)

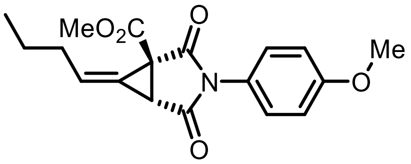

The title compound was prepared according to general procedure **II** from dimethyl 2-butyl-3-((4-methoxyphenyl)carbamoyl)cycloprop-2-ene-1,1-dicarboxylate (**29a**) (36.1 mg, 0.100 mmol, 1.00 eq) with catalyst **C11** (the substrate is sparingly soluble however enters solution overnight). After 24 h the crude reaction mixture was quenched with AcOH to give the crude product in >20:1 d.r. The crude reaction mixture was purified by silica gel column chromatography (pentane/EtOAc = 8/2 to 7/3) to provide the title compound (**29c**) as a colourless oil in 61% yield (20.0 mg) and 97% ee. [determined by HPLC chiralpak AS-H, hexane/isopropanol = 90/10 to 70/30, 1 ml/min, λ = 230 nm, t(major) = 23.73 min, t(minor) = 28.31 min]. **^{1}H NMR** (500 MHz, $CDCl_3$) δ(ppm): 7.12 – 7.07 (m, 2H, ArH), 6.98 – 6.92 (m, 2H, ArH), 6.22 (td, J = 7.0, 2.0 Hz, 1H, C=CHCH$_2$), 3.85 (s, 3H, CO_2CH_3), 3.81 (s, 3H, ArOCH$_3$), 3.50 (q, J = 1.5 Hz, 1H, CHC(=O)), 2.29 (dddd, J = 17.0, 14.5, 7.5, 6.0 Hz, 2H, $CH_2CH_2CH_3$), 1.53 – 1.44 (m, 2H, $CH_2CH_2CH_3$), 0.90 (t, J = 7.5 Hz, 3H, $CH_2CH_2CH_3$); **^{13}C NMR** (126 MHz, $CDCl_3$) δ(ppm): 169.8 (one of C(=O)NC(=O)), 167.5 (one of C(=O)NC(=O)), 164.7 (CO_2Me), 159.8 (ArC), 128.0 (ArCH), 124.2 (ArC), 123.0 (C=CHCH$_2$), 122.7 (C=CHCH$_2$), 114.7 (ArCH), 55.7 (ArOCH$_3$), 53.3 (CO_2CH_3), 35.9 (CCO$_2$Me), 33.2 ($CH_2CH_2CH_3$), 31.7 (CHC(=O)), 21.7 ($CH_2CH_2CH_3$), 13.7 ($CH_2CH_2CH_3$); **IR** (film) ν_{max}/cm^{-1}: 3052, 2959, 2934, 2873 (C-H), 1786, 1714 (has a shoulder) (C=O); **HRMS** (ES+) exact mass calculated for $[M+H]^+$ ($C_{18}H_{20}O_5N$) requires m/z 330.1336, found m/z 330.1336; $[\boldsymbol{\alpha}]_{\boldsymbol{D}}^{\mathbf{26.1}}$ = –148.7 (c 0.32, $CHCl_3$).

Synthesis and characterisation of 30b

dimethyl (R,E)-2-butylidene-3-(diphenylphosphoryl)cyclopropane-1,1-dicarboxylate (30b)

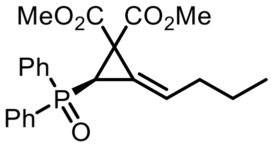

The title compound was prepared according to general procedure **II** from **30a** (20.6 mg, 0.0500 mmol) with catalyst **C11**. The reaction was initiated at −78 °C and allowed to stir for 1 h at which point the temperature was raised to −20 °C. The reaction was stirred for a further 10 min at which point the temperature was raised to 0 °C. The reaction was allowed to progress at this temperature fo 1 h at which point the reaction was quenched with AcOH to give the crude product in 9:1 d.r.. The crude reaction mixture was purified by silica gel column chromatography (pentane/EtOAc = 1/1) to provide the title compound (**30b**) as a colourless oil in 95% yield (18.9 mg) and 99% ee. [determined by HPLC chiralpak AS-H, hexane/isopropanol = 80/20, 1 ml/min, λ = 220 nm, t(major) = 7.16 min, t(minor) = 15.06 min].

^{1}H NMR (500 MHz, $CDCl_3$) δ(ppm): 7.83 – 7.69 (m, 4H, Ar$\underline{H}$), 7.61 – 7.42 (m, 6H, Ar$\underline{H}$), 5.83 (tt, *J* = 6.9, 2.8 Hz, 1H, $C{=}C\underline{H}CH_2$), 3.77 (s, 3H, one of $C(CO_2C\underline{H_3})_A(CO_2CH_3)_B$), 3.70 (s, 3H, one of $C(CO_2CH_3)_A(CO_2C\underline{H_3})_B$), 3.20 (dq, *J* = 9.2, 2.2 Hz, 1H, $C\underline{H}P(=O)$), 2.40 – 2.24 (m, 2H, $C\underline{H_2}CH_2CH_3$), 1.53 – 1.38 (m, 2H, $CH_2C\underline{H_2}CH_3$), 0.85 (t, *J* = 7.4 Hz, 3H, $CH_2CH_2C\underline{H_3}$); **^{13}C NMR** (126 MHz, $CDCl_3$) δ(ppm): 168.2 (one of $C(\underline{C}O_2CH_3)_A(CO_2CH_3)_B$), 165.5 (d, *J* = 2.4 Hz (one of $C(CO_2CH_3)_A(\underline{C}O_2CH_3)_B$)), 132.9 (d, *J* = 105.6 Hz, Ar$\underline{C}$), 132.2 (d, *J* = 2.8 Hz, Ar$\underline{C}$H), 131.4 (dd, *J* = 24.0, 9.9 Hz, Ar$\underline{C}$H), 128.7 (dd, *J* = 12.3, 8.3 Hz, Ar$\underline{C}$H), 123.8 (d, *J* = 4.9 Hz, $C{=}\underline{C}HCH_2$), 118.1 (d, *J* = 8.2 Hz, $\underline{C}{=}CHCH_2$), 53.3 (one of $C(CO_2\underline{C}H_3)_A(CO_2CH_3)_B$), 53.1 (one of $C(CO_2CH_3)_A(CO_2\underline{C}H_3)_B$), 36.3 (d, *J* = 3.2 Hz, $\underline{C}H_2CH_2CH_3$), 33.5 (d, *J* = 1.5 Hz, $\underline{C}(CO_2Me)_2$), 26.5 (d, *J* = 88.5 Hz, $\underline{C}HP(=O)$), 22.0 (d, *J* = 2.2 Hz, $CH_2\underline{C}H_2CH_3$), 13.8 ($CH_2CH_2\underline{C}H_3$); **^{31}P NMR** (162 MHz, $CDCl_3$) δ(ppm): 24.8 ($\underline{P}{=}O$, major diastereomer), 23.2 ($\underline{P}{=}O$, minor diastereomer); **IR** (film) ν_{max}/cm^{-1}: 2958, 2924, 2852 (C-H), 1733 (C=O), 1458;

HRMS (ES+) exact mass calculated for $[M+H]^+$ ($C_{23}H_{26}O_5P^+$) requires *m/z* 413.1512, found *m/z* 413.1511; $[\boldsymbol{\alpha}]_{\boldsymbol{D}}^{\mathbf{26.1}}$ = −33.5 (*c* 0.41, $CHCl_3$)

6.3 Synthesis of enantioenriched cyclopropane ketones

Synthesis and characterisation of 4b

dimethyl (S,Z)-2-butylidene-3-(3-phenylpropanoyl)cyclopropane-1,1-dicarboxylate (4b)

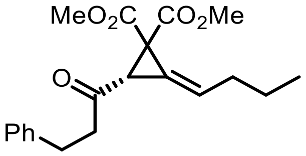

The title compound was prepared according to general procedure **III** from dimethyl 2-butyl-3-(3-phenylpropanoyl)cycloprop-2-ene-1,1-dicarboxylate (**4a**) (34.4 mg, 0.100 mmol, 1.00 eq) with catalyst **C12**. After 2 h the reaction mixture was quenched with AcOH to give the crude product in >20:1 d.r. The crude reaction mixture was purified by silica gel column chromatography (pentane/Et_2O = 8/2) to provide the title compound (**4b**) as a white solid in 99% yield (34.2 mg) and 93% ee. [determined by HPLC chiralpak AD-H, hexane/isopropanol = 220, 1 ml/min, λ = 20.74 nm, t(minor) = 20.74 min, t(major) = 24.47 min]. The sample was recrystallised by evaporation of pentane to provide a sample suitable for single crystal x-ray diffraction in 92% ee. **^{1}H NMR** (500 MHz, $CDCl_3$) δ(ppm): 7.33 – 7.24 (m, 2H, Ar$\underline{H}$), 7.23 – 7.16 (m, 3H, Ar$\underline{H}$), 5.94 (td, *J* = 7.5, 2.0 Hz, 1H, C=C$\underline{H}$), 3.76 (s, 3H, one of $C(CO_2C\underline{H_3})_A(CO_2CH_3)_B$), 3.71 (s, 3H, one of $C(CO_2CH_3)_A(CO_2C\underline{H_3})_B$), 3.43 (q, *J* = 2.0 Hz, 1H, C$\underline{H}$C(=O)), 2.98 – 2.83 (m, 4H, $C\underline{H_2}C\underline{H_2}Ph$), 2.29 (m, 2H, $C\underline{H_2}CH_2CH_3$), 1.52 – 1.44 (m, 2H, $CH_2C\underline{H_2}CH_3$), 0.89 (t, *J* = 7.5 Hz, 3H, $CH_2CH_2C\underline{H_3}$); **^{13}C NMR** (126 MHz, $CDCl_3$) δ(ppm): 202.8 ($CH_2\underline{C}$(=O)), 167.7 (one of $C(\underline{C}O_2CH_3)_A(CO_2CH_3)_B$), 165.7 (one of $C(CO_2CH_3)_A(\underline{C}O_2CH_3)_B$), 140.8 (Ar$\underline{C}$), 128.7 (Ar$\underline{C}$H), 128.5 (Ar$\underline{C}$H), 126.3 (Ar$\underline{C}$H), 123.9 (CHC=$\underline{C}$H), 121.2 (CH$\underline{C}$=CH), 53.4 (one of $C(CO_2\underline{C}H_3)_A(CO_2CH_3)_B$), 53.1 (one of $C(CO_2CH_3)_A(CO_2\underline{C}H_3)_B$), 45.5 ($\underline{C}H_2$C(=O)), 39.7 ($\underline{C}(CO_2Me)$), 36.9 ($CH_2$C(=O)$\underline{C}$H), 33.4 ($\underline{C}H_2CH_2CH_3$), 29.7 ($\underline{C}H_2Ph$), 22.0 ($CH_2\underline{C}H_2CH_3$), 13.8 ($CH_2CH_2\underline{C}H_3$); **IR** (film) ν_{max}/cm^{-1}: 2957, 2873 (C-H), 1736 (C=O ester), 1714 (C=O ketone), 1497, 1454, 1435, 1277, 1247;

HRMS (ES+) exact mass calculated for $[M+H]^+$ ($C_{20}H_{25}O_5^+$) requires *m/z* 345.1697, found *m/z* 345.1694; **MP**: 58 – 60 °C; $[\boldsymbol{\alpha}]_{\boldsymbol{D}}^{\mathbf{25}}$ = +130.63 (*c* 0.65, $CHCl_3$).

NOESY of compound **4b**: Cross-peaks are observed between C=C<u>H</u>CH_2 and CHC(=O).

Synthesis and characterisation of 31b

dimethyl (S)-2-cyclopentylidene-3-(3-phenylpropanoyl)cyclopropane-1,1-dicarboxylate (31b)

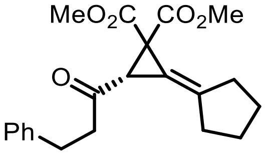

The title compound was prepared according to general procedure **III** from dimethyl 2-cyclopentyl-3-(3-phenylpropanoyl)cycloprop-2-ene-1,1-dicarboxylate (**31a**) (35.6 mg, 0.100 mmol, 1.00 eq) with catalyst **C12**. After 4 h the reaction was quenched, and the crude reaction mixture was purified by silica gel column chromatography (pentane/Et_2O = 9/1) to provide the title compound (**31b**) as a colourless oil in 90% yield (32.3 mg) and 99% ee. [determined by HPLC chiralpak AD-H, hexane/isopropanol = 98/2, 1 ml/min, λ = 220 nm, t(minor) = 20.74 min, t(major) = 24.47 min]. **^{1}H NMR** (500 MHz, Benzene-d_6) δ(ppm): 7.13 – 7.07 (m, 2H, Ar$\underline{H}$), 7.05 – 6.96 (m, 3H, Ar$\underline{H}$), 3.52 (p, *J* = 2.5 Hz, 1H, C$\underline{H}$C(=O)), 3.45 (s, 3H, one of $C(CO_2C\underline{H_3})_A(CO_2CH_3)_B$), 3.31 (s, 3H, one of $C(CO_2CH_3)_A(CO_2C\underline{H_3})_B$), 2.90 – 2.77 (m, 3H, $C\underline{H_2}$Ph and one of $C\underline{H_A}H_BCH_2CH_2CH_CH_D$), 2.63 – 2.45 (m, 2H, $C\underline{H_2}CH_2$Ph), 2.46 – 2.33 (m, 1H, one of $CH_A\underline{H_B}CH_2CH_2CH_CH_D$), 2.21 – 2.11 (m, 2H, two of $CH_AH_BCH_2CH_2C\underline{H_C}\underline{H_D}$), 1.54 – 1.33 (m, 4H, $CH_2C\underline{H_2}C\underline{H_2}CH_2$); **^{13}C NMR** (126 MHz, Benzene-d_6) δ(ppm): 201.4 ($\underline{C}$=OCH_2), 167.7 (one of $C(\underline{C}O_2Me)_A(CO_2Me)_B$), 165.6 (one of $C(CO_2Me)_A(\underline{C}O_2Me)_B$), 141.0 (Ar$\underline{C}$), 138.2 ($CH_2\underline{C}$=CCH), 128.4 (Ar$\underline{C}$H), 128.3 (Ar$\underline{C}$H), 125.9 (Ar$\underline{C}$H), 111.8 ($CH_2C$=$\underline{C}$CH), 52.2 (one of $C(CO_2\underline{C}H_3)_A(CO_2CH_3)_B$), 52.1 (one of $C(CO_2CH_3)_A(CO_2\underline{C}H_3)_B$), 45.1 ($\underline{C}H_2CH_2$Ph), 40.5 ($\underline{C}(CO_2Me)_2$), 37.1 ($\underline{C}$HC(=O)), 32.1 (one of $CH_2(\underline{C}H_2)_A(CH_2)_BCH_2$), 32.0 (one of $CH_2(CH_2)_A(\underline{C}H_2)_BCH_2$), 29.5 ($CH_2\underline{C}H_2$Ph), 26.3 (one of C=C$(\underline{C}H_2)_A(CH_2)_B$), 26.3 (one of C=C$(CH_2)_A(\underline{C}H_2)_B$); **IR** (film) ν_{max}/cm^{-1}: 2954 (C-H), 1732, 1711 (C=O); $[\alpha]_D^{26}$ = +87.5 (*c* 0.26, $CHCl_3$).

Synthesis and characterisation of 32b

dimethyl (S,Z)-2-butylidene-3-(4-methoxybenzoyl)cyclopropane-1,1-dicarboxylate (32b)

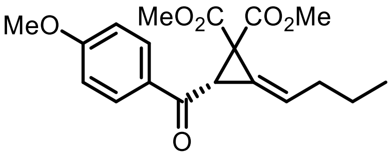

The title compound was prepared according to general procedure **III** from dimethyl 2-butyl-3-(4-methoxybenzoyl)cycloprop-2-ene-1,1-dicarboxylate (**32a**) (34.6 mg, 0.100 mmol, 1.00 eq) with catalyst **C12**. After 2 h the reaction mixture was quenched with AcOH to give the crude product in >20:1 d.r. The crude reaction mixture was purified by silica gel column chromatography (toluene/EtOAc = 95/5 then pentane/Et_2O = 7/3) to provide the title compound (**32b**) as a colourless oil in 97% yield (33.7 mg) and 84% ee. (If the reaction is left for 1 h the product is obtained in 91% yield and 88% ee) [determined by HPLC chiralpak AD-H, hexane/isopropanol = 95/5, 1 ml/min, λ = 220 nm, t(minor) = 36.13 min, t(major) = 35.01 min]. **^{1}H NMR** (500 MHz, $CDCl_3$) δ(ppm): 8.05 – 7.97 (m, 2H, Ar<u>H</u>), 7.01 – 6.92 (m, 2H, Ar<u>H</u>), 6.07 (td, *J* = 7.5, 2.5 Hz, 1H, C=C<u>H</u>CH_2), 4.10 (q, *J* = 2.0 Hz, 1H, C<u>H</u>C=O), 3.88 (s, 3H, ArOC<u>H$_3$</u>), 3.82 (s, 3H, one of $C(CO_2C\underline{H_3})_A(CO_2CH_3)_B$), 3.68 (s, 3H, one of $C(CO_2CH_3)_A(CO_2C\underline{H_3})_B$), 2.41 – 2.23 (m, 2H, $C\underline{H_2}CH_2CH_3$), 1.54 – 1.45 (m, 2H, $CH_2C\underline{H_2}CH_3$), 0.91 (t, *J* = 7.5 Hz, 3H, $CH_2CH_2C\underline{H_3}$); **^{13}C NMR** (126 MHz, $CDCl_3$) δ(ppm): 190.7 (<u>C</u>=OAr), 167.9 (one of $C(\underline{C}O_2CH_3)_A(CO_2CH_3)_B$), 166.2 (one of $C(CO_2CH_3)_A(\underline{C}O_2CH_3)_B$), 164.1 (Ar<u>C</u>), 131.1 (Ar<u>C</u>H), 129.8 (Ar<u>C</u>), 123.6 (C=<u>C</u>HCH_2), 121.5 (<u>C</u>=CHCH_2), 114.1 (Ar<u>C</u>H), 55.7 (ArO<u>C</u>H_3), 53.4 (one of $C(CO_2\underline{C}H_3)_A(CO_2CH_3)_B$), 52.9 (one of $C(CO_2CH_3)_A(CO_2\underline{C}H_3)_B$), 39.1 ($\underline{C}(CO_2CH_3)_A(CO_2CH_3)_B$), 34.5 (C<u>H</u>C=O), 33.4 ($\underline{C}H_2CH_2CH_3$), 22.1 ($CH_2\underline{C}H_2CH_3$), 13.8 ($CH_2CH_2\underline{C}H_3$); **IR** (film) v_{max}/cm^{-1}: 2958, 2873, 2843 (C-H), 1732 (C=O ester), 1673 (C=O ketone), 1248 (C-O); **HRMS** (ES+) exact mass calculated for $[M+H]^+$ ($C_{19}H_{23}O_6^+$) requires *m/z* 347.1489, found *m/z* 347.1484; $[\boldsymbol{\alpha}]_{\boldsymbol{D}}^{\mathbf{26.1}}$ = –93.1 (*c* 0.45, $CHCl_3$).

6.4 Synthesis of enantioenriched insecticide cores

Synthesis and characterisation of 33b

benzyl (S,Z)-3-(((2-methoxyethoxy)methoxy)methylene)-2,2-dimethylcyclopropane-1-carboxylate (33b)

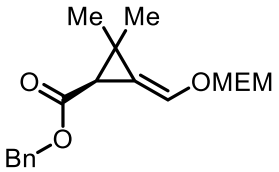

The title compound was prepared according to general procedure **II** from benzyl 2-(((2-methoxyethoxy)methoxy)methyl)-3,3-dimethylcycloprop-1-ene-1-carboxylate (**33a**) (32.0 mg, 0.100 mmol) with catalyst **C13**. After 1 h the reaction mixture was quenched with AcOH to give the crude product in >20:1 d.r. The crude reaction mixture was purified by silica gel column chromatography (pentane/EtOAc = 9/1) to provide the title compound (**33b**) as a colourless oil in 90% yield (28.9 mg) and 98% ee. [determined by HPLC chiralpak AD-H, hexane/isopropanol = 98/2, 1 ml/min, λ = 220 nm, t(major) = 14.80 min, t(minor) = 22.53 min].

^{1}H NMR (400 MHz, Benzene-d_6) δ(ppm): 7.26 – 7.20 (m, 2H, Ar$\underline{H}$), 7.12 – 7.01 (m, 3H, Ar$\underline{H}$), 6.58 (d, *J* = 1.7 Hz, 1H, C=C$\underline{H}$(OMEM)), 5.07 (d, *J* = 12.4 Hz, 1H, C$\underline{H_A}$H$_B$Ph), 5.07 (d, *J* = 12.4 Hz, 1H, CH$_A$$\underline{H_B}$Ph), 4.78 (d, *J* = 6.3 Hz, 1H, OC$\underline{H_A}$H$_B$O), 4.72 (d, *J* = 6.3 Hz, 1H, OCH$_A$$\underline{H_B}$O), 3.58 – 3.52 (m, 2H, OC$\underline{H_2}$CH$_2$OCH$_3$), 3.26 (dd, *J* = 5.4, 4.6 Hz, 2H, OCH$_2$C$\underline{H_2}$OCH$_3$), 3.07 (s, 3H, OC$\underline{H_3}$), 2.20 (d, *J* = 1.7 Hz, 1H, C$\underline{H}$CO$_2$Bn), 1.46 (s, 3H, one of C(C$\underline{H_3}$)$_A$(CH$_3$)$_B$), 1.15 (s, 3H, one of C(CH$_3$)$_A$(C$\underline{H_3}$)$_B$); **^{13}C NMR** (101 MHz, Benzene-d_6) δ(ppm): 170.5 ($\underline{C}$=O), 137.0 (Ar$\underline{C}$), 136.0 (C=$\underline{C}$H(OMEM)), 128.7 (ArC$\underline{H}$), 128.6 (ArC$\underline{H}$), 128.2 (ArC$\underline{H}$), 109.9 ($\underline{C}$=CH(OMEM)), 94.7 (O$\underline{C}$H$_2$O), 71.9 ($\underline{C}$H$_2$OCH$_3$), 68.1 ($\underline{C}$H$_2$CH$_2$OCH$_3$), 66.3 ($\underline{C}$H$_2$Ph), 58.6 (O$\underline{C}$H$_3$), 29.6 ($\underline{C}$HCO$_2$Bn), 28.3 ($\underline{C}$(CH$_3$)$_2$), 26.2 (one of C($\underline{C}$H$_3$)$_A$(CH$_3$)$_B$), 19.2 (one of C(CH$_3$)$_A$($\underline{C}$H$_3$)$_B$); **HRMS** (ES+) exact mass calculated for

$[M+Na]^+$ ($C_{18}H_{24}NaO_5^+$) requires *m/z* 343.1516, found *m/z* 343.1517; $[\boldsymbol{\alpha}]_{\boldsymbol{D}}^{\mathbf{25}}$ = –51.1 (*c* 0.31, $CHCl_3$).

Synthesis and characterisation of 34b

3-phenoxybenzyl (S,Z)-3-(((2-methoxyethoxy)methoxy)methylene)-2,2-dimethylcyclopropane-1-carboxylate (34b)

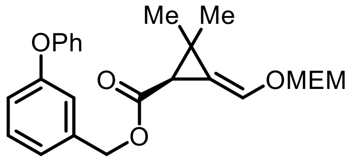

The title compound was prepared according to general procedure **II** from 3-phenoxybenzyl 2-(((2-methoxyethoxy)methoxy)methyl)-3,3-dimethylcycloprop-1-ene-1-carboxylate (**34a**) (41.2 mg, 0.100 mmol) with catalyst **C13**. After 1 h the reaction mixture was quenched with AcOH to give the crude product in >20:1 d.r. The crude reaction mixture was purified by silica gel column chromatography (pentane/EtOAc = 9/1) to provide the title compound (**34b**) as a colourless oil in 88% yield (36.2 mg) and 98% ee. [determined by HPLC chiralpak AS-H, hexane/isopropanol = 95/5, 1 ml/min, λ = 220 nm, t(major) = 10.76 min, t(minor) = 21.54 min]. **^{1}H NMR** (400 MHz, $CDCl_3$) δ(ppm): 7.40 – 7.27 (m, 3H, ArH), 7.15 – 7.06 (m, 2H, ArH), 7.04 – 6.99 (m, 3H, ArH), 6.95 (ddd, *J* = 8.2, 2.5, 1.0 Hz, 1H, ArH), 6.60 (d, *J* = 1.7 Hz, 1H, C=CH(OMEM)), 5.09 (s, 2H, CO_2CH_2Ph), 4.99 (d, *J* = 6.1 Hz, 1H, one of OCH_AH_BO), 4.97 (d, *J* = 6.1 Hz, 1H, one of OCH_AH_BO), 3.79 – 3.71 (m, 2H, $CH_2CH_2OCH_3$), 3.65 – 3.48 (m, 2H, $CH_2CH_2OCH_3$), 3.38 (s, 3H, OCH_3), 2.17 (d, *J* = 1.7 Hz, 1H, $CHCO_2CH_2Ar$), 1.33 (s, 3H, one of $C(CH_3)_A(CH_3)_B$), 1.33 (s, 3H, one of $C(CH_3)_A(CH_3)_B$); **^{13}C NMR** (101 MHz, $CDCl_3$) δ(ppm): 171.0 (C=O), 157.6 (ArC), 157.1 (ArC), 138.3 (ArC), 135.4 (C=CH(OMEM)), 130.0 (ArCH), 129.9 (ArCH), 123.6 (ArCH), 122.8 (ArCH), 119.2 (ArCH), 118.5 (ArCH), 118.3 (ArCH), 109.7 (C=CH(OMEM)), 94.6 (OCH_2O), 71.7 (CH_2OCH_3), 67.9 ($CH_2CH_2OCH_3$), 65.9 (CH_2Ph), 59.2 (OCH_3), 29.4 ($CHCO_2CH_2Ar$), 28.5 ($C(CH_3)_2$), 26.4 (one of $C(CH_3)_A(CH_3)_B$), 19.3 (one of $C(CH_3)_A(CH_3)_B$); **IR** (film) ν_{max}/cm^{-1}: 2929, 1729 (C=O), 1584, 1487; **HRMS** (ES+) exact mass calculated for $[M+Na]^+$ ($C_{24}H_{28}NaO_6^+$) requires *m/z* 435.1778, found *m/z* 435.1777; $[\alpha]_D^{25}$ = –50.6 (*c* 0.81, $CHCl_3$).

Synthesis and characterisation of 2

(1R, 3R)-cis-Permethrin (2)

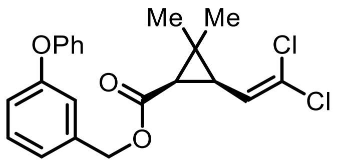

The title compound was prepared according to general procedure **II** from 3-phenoxybenzyl 2-(((2-methoxyethoxy)methoxy)methyl)-3,3-dimethylcycloprop-1-ene-1-carboxylate (**34a**) (41.2 mg, 0.100 mmol, 1.00 eq) with catalyst **C13**. After 1 h $HCl_{(aq)}$ (1.0 M, 2.0 ml) was added, and the reaction was stirred vigorously overnight. The aqueous layer was extracted with Et_2O (3 x 10 ml). The combined organic layers were washed with brine, dried over Na_2SO_4, filtered and concentrated *in vacuo*. Triphenyl phosphine (105 mg, 0.400 mmol, 4.00 eq) was dissolved in CH_2Cl_2 (0.1 ml) and CCl_4 (0.2 ml) was added at 0 °C The crude product was dissolved in CH_2Cl_2 (0.24 ml) and added to the solution of triphenyl phosphine. The reaction was warmed to room temperature. H_2O (5 ml) was added, and the aqueous phase was extracted with CH_2Cl_2 (3 x 10 ml). The combined organic layers were washed with brine, dried over Na_2SO_4, filtered and concentrated *in vacuo*. The crude reaction mixture was purified by silica gel column chromatography (pentane/Et_2O = 99/1) to provide the title compound (**2**) as a white solid in 31% yield (12.2 mg) and 98% ee. [determined by HPLC chiralpak IB, hexane/isopropanol = 99/1, 1 ml/min, λ = 230 nm, t(major) = 7.51 min, t(minor) = 8.08 min]. **^{1}H NMR** (500 MHz, $CDCl_3$) δ(ppm): 7.38 – 7.29 (m, 3H, Ar$\underline{H}$), 7.14 – 7.06 (m, 2H, Ar$\underline{H}$), 7.04 – 7.00 (m, 3H, Ar$\underline{H}$), 6.98 – 6.91 (m, 1H, Ar$\underline{H}$), 6.26 (d, *J* = 9.0 Hz, 1H, C$\underline{H}$=CCl_2), 5.09 (d, *J* = 12.5 Hz, 1H, one of C$\underline{H_A}H_B$Ar), 5.05 (d, *J* = 12.5 Hz, 1H, one of C$H_A\underline{H_B}$Ar), 2.04 (app-t, *J* = 8.5 Hz, 1H, C$\underline{H}$CH=CCl_2), 1.89 (d, *J* = 8.5 Hz, 1H, C$\underline{H}$C(=O)OCH_2), 1.24 (s, 3H, one of C(C$\underline{H_3}$)$_A$(CH_3)$_B$), 1.24 (s, 3H, one of C(CH_3)$_A$(C$\underline{H_3}$)$_B$); **^{13}C NMR** (126 MHz, $CDCl_3$) δ(ppm): 170.4 ($\underline{C}$=O), 157.7 (Ar$\underline{C}$), 157.1 (Ar$\underline{C}$), 138.1 (Ar$\underline{C}$), 130.1 (Ar$\underline{C}$H), 130.0 (Ar$\underline{C}$H), 124.9 ($\underline{C}$=CCl_2), 123.6 (Ar$\underline{C}$H), 122.8 (Ar$\underline{C}$H), 121.0 (C=$\underline{C}Cl_2$), 119.2 (Ar$\underline{C}$H), 118.6 (Ar$\underline{C}$H), 118.4 (Ar$\underline{C}$H), 65.9 (O$\underline{C}H_2$), 32.9

($\underline{C}$HCH=CCl_2), 32.0 ($\underline{C}$HC(=O)CH_2), 28.5 (one of C($\underline{C}H_3$)$_A$(CH_3)$_B$), 27.8 ($\underline{C}$(CH_3)$_2$), 15.1 (one of C($\underline{C}H_3$)$_A$(CH_3)$_B$); **IR** (film) v_{max}/cm^{-1}: 3067, 2957 (C-H), 1726 (C=O), 1585, 1488, 1258, 1215, 1180, 1135, 1084; $[\boldsymbol{\alpha}]_{\boldsymbol{D}}^{\mathbf{25}}$ = +1.81 (c 1.43, CH_2Cl_2). Lit: $[\boldsymbol{\alpha}]_{\boldsymbol{D}}^{\mathbf{20}}$ = +2.6 (c 1.5, CH_2Cl_2).[22] Data are consistent with that published in the literature.[22]

Synthesis and characterisation of 35b

2,3,5,6-tetrafluoro-4-methylbenzyl (1R,3S)-3-formyl-2,2-dimethylcyclopropane-1-carboxylate (35b)

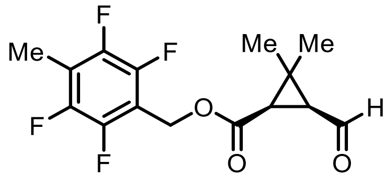

The title compound was prepared according to general procedure **II** from **35a** (40.6 mg, 0.100 mmol, 1.00 eq) with catalyst **C13**. After 60 min $HCl_{(aq)}$ (1.0 M, 2 ml) was added, and the reaction was stirred vigorously overnight. The aqueous layer was extracted with Et_2O (3 x 10 ml). The combined organic layers were washed with brine, dried over Na_2SO_4, filtered and concentrated *in vacuo* which provided the crude product in >20:1 d.r.. The crude reaction mixture was purified by silica gel column chromatography (pentane/Et_2O = 9/1) to provide the title compound (**35b**) as a colourless oil in 75% yield (23.9 mg) and 98% ee. [determined by HPLC chiralpak AS-H, hexane/isopropanol = 98/2, 1 ml/min, λ = 210 nm, t(major) = 18.91 min, t(minor) = 25.58 min]. **^{1}H NMR** (600 MHz, $CDCl_3$) δ(ppm): 9.75 (d, *J* = 6.5 Hz, 1H, C<u>H</u>O), 5.27 – 5.21 (m, 2H, C$\underline{H_A}\underline{H_B}$Ar), 2.29 (t, J_{HF} = 2.0 Hz, 3H, C$\underline{H_3}$Ar), 2.12 (d, *J* = 8.5 Hz, 1H, C<u>H</u>CHO), 1.87 (dd, *J* = 8.5, 6.5 Hz, 1H, C<u>H</u>CO_2CH_2), 1.56 (s, 3H, one of C(C$\underline{H_3}$)$_A$(CH_3)$_B$), 1.27 (s, 3H, one of C(CH_3)$_A$(C$\underline{H_3}$)$_B$); **^{13}C NMR** (151 MHz, $CDCl_3$) δ(ppm): 200.1 (<u>C</u>HO), 169.4 (<u>C</u>O_2CH_2), 146.2 – 145.5 (m, Ar<u>C</u>), 144.7 – 143.9 (m, Ar<u>C</u>), 117.7 (t, J_{CF} = 19.0 Hz, Ar<u>C</u>), 111.3 (t, J_{CF} = 17.0 Hz, Ar<u>C</u>), 54.5 – 54.26 (m, <u>C</u>H_2Ar), 41.1 (<u>C</u>HCO_2CH_2), 35.8 (<u>C</u>HCHO), 30.3 (<u>C</u>Me_2), 28.3 (one of C(<u>C</u>H_3)$_A$(CH_3)$_B$), 15.0 (one of C(CH_3)$_A$(<u>C</u>H_3)$_B$), 8.1 – 7.7 (m, <u>C</u>H_3Ar); **^{19}F NMR** (565 MHz, $CDCl_3$) δ(ppm): -143.3 (dd, *J* = 21.5, 13.0 Hz, 2 x ArC<u>F</u>), -144.4 (dd, *J* = 21.5, 13.0 Hz, 2 x ArC<u>F</u>); **IR** (film) ν_{max}/cm^{-1}: 3056, 2960 (C-H) 1729 (C=O ester), 1693 (C=O aldehyde) 1660; **HRMS** (ES+) exact mass calculated for $[M+Na]^+$

($C_{15}F_4H_{14}NaO_3^+$) requires *m/z* 341.0771, found *m/z* 341.0772; **MP**: 72 – 73 °C; $[\boldsymbol{\alpha}]_{\boldsymbol{D}}^{\mathbf{26.1}}$ = −47.4 (*c* 0.56, $CHCl_3$).

Synthesis and characterisation of 36b

2,3,5,6-tetrafluoro-4-(methoxymethyl)benzyl (1R,3S)-3-formyl-2,2-dimethylcyclopropane-1-carboxylate (36b)

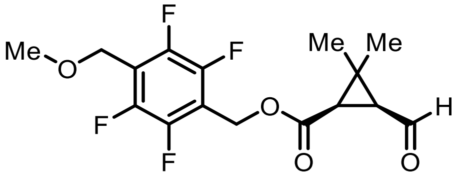

The title compound was prepared according to general procedure **II** from **36a** (43.6 mg, 0.100 mmol, 1.00 eq) with catalyst **C13**. After 60 min $HCl_{(aq)}$ (1.0 M, 2 ml) was added, and the reaction was stirred vigorously overnight. The aqueous layer was extracted with Et_2O (3 x 10 ml). The combined organic layers were washed with brine, dried over Na_2SO_4, filtered and concentrated *in vacuo* which provided the crude product in >20:1 d.r.. The crude reaction mixture was purified by silica gel column chromatography (pentane/Et_2O = 8/2) to provide the title compound (**36b**) as a white solid in 76% yield (26.3 mg) and 97% ee. [determined by HPLC chiralpak AS-H, hexane/isopropanol = 97/3, 1 ml/min, λ = 210 nm, t(major) = 28.46 min, t(minor) = 36.97 min]. **^{1}H NMR** (600 MHz, $CDCl_3$) δ(ppm): 9.75 (d, *J* = 6.5 Hz, 1H, C<u>H</u>O), 5.29 (dt, *J* = 12.0, 1.5 Hz, 1H, one of C<u>H$_A$</u>H$_B$Ar), 5.25 (dt, *J* = 12.0, 1.5 Hz, 1H, one of CH$_A$H$_B$Ar), 4.59 (t, J_{HF} = 2.0 Hz, 2H, C<u>H$_2$</u>OCH$_3$), 3.41 (s, 3H, OC<u>H$_3$</u>), 2.12 (d, *J* = 8.5 Hz, 1H, C<u>H</u>CO$_2$CH$_2$), 1.88 (dd, *J* = 8.5, 6.5 Hz, 1H, C<u>H</u>CHCO$_2$), 1.56 (s, 3H, one of C(C<u>H$_3$</u>)$_A$(CH$_3$)$_B$), 1.27 (s, 3H, one of C(CH$_3$)$_A$(C<u>H$_3$</u>)$_B$); **^{13}C NMR** (151 MHz, $CDCl_3$) δ(ppm): 201.9 – 198.1 (m, <u>C</u>HO), 169.4 (<u>C</u>O$_2$CH$_2$), 146.3 – 145.9 (m, Ar<u>C</u>), 144.7 – 144.2 (m, Ar<u>C</u>), 117.4 (t, J_{CF} = 18.0 Hz, Ar<u>C</u>), 114.4 (t, J_{CF} = 17.0 Hz, Ar<u>C</u>), 61.6 (t, J_{CF} = 36.0 Hz, <u>C</u>H$_2$OCH$_3$), 58.9 (t, J_{CF} = 37.5 Hz, CH$_2$O<u>C</u>H$_3$), 54.7 – 53.6 (m, CO$_2$<u>C</u>H$_2$), 41.6 – 40.7 (m, <u>C</u>HCHO), 35.7 (d, J_{CF} = 72.0 Hz, <u>C</u>HCO$_2$CH$_2$), 30.3 (<u>C</u>(CH$_3$)$_2$), 28.28 (q, J_{CF} = 16.5 Hz, one of C(<u>C</u>H$_3$)$_A$(CH$_3$)$_B$), 14.99 (t, J_{CF} = 38.0 Hz, one of C(CH$_3$)$_A$(<u>C</u>H$_3$)$_B$); **^{19}F NMR** (565 MHz, $CDCl_3$) δ(ppm): -142.8 (dd, *J* = 22.0, 13.5 Hz, 2 x ArC<u>F</u>), -143.2 (dd, *J* = 22.0, 14.0 Hz, 2 x ArC<u>F</u>); **IR** (film) ν_{max}/cm^{-1}: 2970 (C-H),

1733 (C=O ester), 1700 (C=O aldehyde); **HRMS** (ES+) exact mass calculated for $[M+Na]^+$ ($C_{16}F_4H_{16}NaO_4^+$) requires *m/z* 371.0877, found *m/z* 371.0877; $[\boldsymbol{\alpha}]_{\boldsymbol{D}}^{\mathbf{26.1}}$ = −37.1 (*c* 0.67, $CHCl_3$).

Synthesis and characterisation of 37b

(2-methyl-[1,1'-biphenyl]-3-yl)methyl (1R,3S)-3-formyl-2,2-dimethylcyclopropane-1-carboxylate (37b)

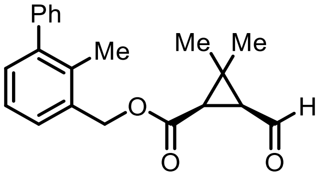

The title compound was prepared according to general procedure **II** from **37a** (41.5 mg, 0.100 mmol, 1.00 eq) with catalyst **C13**. After 60 min $HCl_{(aq)}$ (1.0 M, 2 ml) was added and the reaction was stirred vigorously overnight. The aqueous layer was extracted with Et_2O (3 x 10 ml). The combined organic layers were washed with brine, dried over Na_2SO_4, filtered and concentrated *in vacuo* which provided the crude product in >20:1 d.r.. The crude reaction mixture was purified by silica gel column chromatography (pentane/Et_2O = 9/1) to provide the title compound (**37b**) as a colourless oil in 74% yield (23.7 mg) and 98% ee. [determined by HPLC chiralpak AS-H, hexane/isopropanol = 95/5, 1 ml/min, λ = 210 nm, t(major) = 19.53 min, t(minor) = 24.47 min]. **^{1}H NMR** (600 MHz, $CDCl_3$) δ(ppm): 9.79 (d, *J* = 6.5 Hz, 1H, C<u>H</u>O), 7.44 – 7.40 (m, 2H, Ar<u>H</u>), 7.38 – 7.33 (m, 2H, Ar<u>H</u>), 7.32 – 7.27 (m, 2H, Ar<u>H</u>), 7.25 (d, *J* = 2.5 Hz, 2H, Ar<u>H</u>), 5.24 (s, 2H, C$\underline{H_A H_B}$Ar), 2.23 (s, 3H, C$\underline{H_3}$Ar), 2.20 (d, *J* = 8.5 Hz, 1H, C<u>H</u>CHO), 1.88 (dd, *J* = 8.5, 6.5 Hz, 1H, C<u>H</u>CO_2CH_2), 1.58 (s, 3H, one of $C(C\underline{H_3})_A(CH_3)_B$), 1.28 (s, 3H, one of $C(CH_3)_A(C\underline{H_3})_B$); **^{13}C NMR** (151 MHz, $CDCl_3$) δ(ppm): 200.5 (C<u>H</u>O), 170.0 ($\underline{C}O_2CH_2$), 143.2 (Ar<u>C</u>), 141.9 (Ar<u>C</u>), 134.7 (Ar<u>C</u>), 134.2 (Ar<u>C</u>), 130.7 (Ar<u>C</u>H), 129.5 (Ar<u>C</u>H), 128.7 (Ar<u>C</u>H), 128.3 (Ar<u>C</u>H), 127.1 (Ar<u>C</u>H), 125.8 (Ar<u>C</u>H), 66.1 ($CO_2\underline{C}H_2$), 41.1 ($\underline{C}HCO_2CH_2$), 36.4 ($\underline{C}HCHCO_2CH_2$), 30.1 ($\underline{C}Me_2$), 28.4 (one of $C(\underline{C}H_3)_A(CH_3)_B$), 16.4 ($\underline{C}H_3Ar$), 15.1 (one of $C(CH_3)_A(\underline{C}H_3)_B$); **IR** (film) ν_{max}/cm^{-1}: 2980, 2888 (C-H) 1723 (C=O ester), 1698 (C=O aldehyde); **HRMS** (ES+) exact mass calculated for $[M+Na]^+$ ($C_{21}H_{22}NaO_3^+$) requires *m/z* 345.1461, found *m/z* 345.1462; $[\alpha]_D^{26.1}$ = −38.8 (*c* 0.67, $CHCl_3$).

Synthesis and characterisation of 39b

benzyl (S,Z)-3-((allyloxy)methylene)-2,2-dimethylcyclopropane-1-carboxylate (39b)

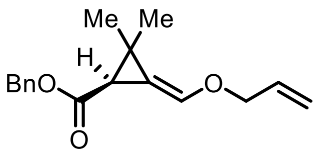

The title compound was prepared according to general procedure **II** from benzyl 2-((allyloxy)methyl)-3,3-dimethylcycloprop-1-ene-1-carboxylate (**39a**) (57.4 mg, 0.200 mmol, 1.00 eq) with catalyst **C11**. After 1 h the reaction was quenched with was quenched with AcOH to give the crude product >20:1 d.r. The crude reaction mixture was purified by silica gel column chromatography (pentane/Et_2O = 10/1) to provide the title compound (**39b**) as a colourless oil in 85% yield (49.0 mg) and 97% ee. [determined by HPLC chiralpak AD, hexane/isopropanol = 95/5, 1 ml/min, λ = 230 nm, t(major) = 5.54 min, t(minor) = 6.20 min]. **^{1}H NMR** (400 MHz, Benzene-d_6) δ(ppm): 7.30 – 7.19 (m, 2H, ArH), 7.13 – 7.02 (m, 3H, ArH), 6.64 (d, J = 1.6 Hz, 1H, $C{=}C\underline{H}OCH_2$), 5.79 (ddt, J = 17.2, 10.4, 5.2 Hz, 1H, $OCH_2C\underline{H}{=}CH_2$), 5.22 (dq, J = 17.2, 1.7 Hz, 1H, $OCH_2{=}CHC\underline{H_A}H_B$), 5.07 (d, J = 1.6 Hz, 2H, CH_2Ph), 5.01 (dq, J = 10.6, 1.5 Hz, 1H, $OCH_2CH{=}CH_A\underline{H_B}$), 4.11 (qdt, J = 12.9, 5.3, 1.6 Hz, 2H, $OC\underline{H_2}CH{=}CH_AH_B$), 2.16 (d, J = 1.7 Hz, 1H, $C\underline{H}CO_2Bn$), 1.36 (s, 3H, one of $C(C\underline{H_3})_A(CH_3)_B$), 1.02 (s, 3H, one of $C(CH_3)_A(C\underline{H_3})_B$); **^{13}C NMR** (101 MHz, Benzene-d_6) δ(ppm): 170.6 ($\underline{C}{=}O$), 138.2 (Ar$\underline{C}$), 137.0 ($C{=}\underline{C}HOCH_2$), 133.8 ($OCH_2\underline{C}H{=}CH_2$), 128.7 (ArC$\underline{H}$), 128.7 (ArC$\underline{H}$), 116.8 ($OCH_2CH{=}\underline{C}H_2$), 107.3 ($\underline{C}{=}CHOCH_2$), 69.9 ($O\underline{C}H_2CH{=}CH_2$), 66.3 ($\underline{C}H_2Ph$), 29.1 ($\underline{C}HCO_2Bn$), 28.0 ($\underline{C}(CH_3)_2$), 27.3 (one of $C(\underline{C}H_3)_A(CH_3)_B$), 20.2 (one of $C(CH_3)_A(\underline{C}H_3)_B$); **HRMS** (ES+) exact mass calculated for $[M+H]^+$ ($C_{17}H_{21}O_3^+$) requires *m/z* 273.1485, found *m/z* 273.1472; $[\boldsymbol{\alpha}]_{\boldsymbol{D}}^{\mathbf{20}}$ = –95.0 (*c* 0.81, CH_2Cl_2).

7. Derivatisation

Synthesis and characterisation of 40

(S,Z)-(3-((allyloxy)methylene)-2,2-dimethylcyclopropyl)methanol (40)

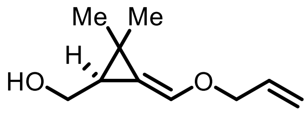

Under Ar, DIBAL-H in toluene (0.2 ml, 1.0 M, 4.0 equiv.) was added dropwise to a stirred solution of **39b (**13.6 mg, 0.05 mmol, with 97% ee) in toluene (0.5 ml) at −78 °C. The solution was stirred at −78 °C for 3 h. The reaction was quenched by addition of MeOH (0.5 ml) dropwise. The mixture was warmed to room temperature and diluted with EtOAc (5 ml) and water (5 ml). The aqueous phase was extracted with EtOAc (3 x 5 ml). The combined organic extracts were washed with brine, dried over Na_2SO_4, filtered and concentrated *in vacuo* to provide the crude product in >20:1 d.r.. The crude material was purified by silica gel column chromatography (pentane/Et_2O=2/1) to provide **40** as colourless oil (4.4 mg, 52%) and 97% ee. [determined by HPLC chiralpak AD-H, hexane/isopropanol = 99/1, 1 ml/min, λ = 210 nm, t(major) = 27.38 min, t(minor) = 32.37 min]. **^{1}H NMR** (400 MHz, Benzene-d_6) δ(ppm): 6.57 (d, *J* = 1.7 Hz, 1H, C=C<u>H</u>OCH$_2$), 5.92 – 5.80 (m, 1H, OCH$_2$C<u>H</u>=CH$_2$), 5.29 (dq, *J* = 17.2, 1.8 Hz, 1H, OCH$_2$=CHC<u>H$_A$</u>H$_B$), 5.05 (dq, *J* = 10.5, 1.5 Hz, 1H, OCH$_2$CH=CH$_A$<u>H$_B$</u>), 4.19 – 4.09 (m, 2H, OC<u>H$_2$</u>CH=CH$_A$H$_B$), 3.52 (dd, *J* = 11.4, 5.8 Hz, 1H, HOC<u>H$_A$</u>H$_B$), 3.35 (dd, *J* = 11.4, 8.6 Hz, 1H, HOCH$_A$<u>H$_B$</u>), 1.42 (ddd, *J* = 8.6, 5.8, 1.7 Hz, 1H, HOCH$_2$C<u>H</u>), 1.10 (s, 3H, one of C(C<u>H$_3$</u>)$_A$(CH$_3$)$_B$), 1.08 (s, 3H, one of C(CH$_3$)$_A$(C<u>H$_3$</u>)$_B$); **^{13}C NMR** (101 MHz, Benzene-d_6) δ(ppm): 136.3 (C=<u>C</u>HOCH$_2$), 134.4 (OCH$_2$<u>C</u>H=CH$_2$), 116.4 (OCH$_2$CH=<u>C</u>H$_2$), 108.5 (<u>C</u>=CHOCH$_2$), 69.5 (O<u>C</u>H$_2$CH=CH$_2$), 62.6 (HO<u>C</u>H$_2$CH), 28.6 (<u>C</u>(CH$_3$)$_2$), 27.7 (one of C(<u>C</u>H$_3$)$_A$(CH$_3$)$_B$), 22.3 (HOCH$_2$<u>C</u>H), 20.3 (one of C(CH$_3$)$_A$(<u>C</u>H$_3$)$_B$); **HRMS** (ES+) exact mass calculated for $[M+Na]^+$ ($C_{10}H_{16}O_2Na^+$) requires *m/z* 191.1043, found *m/z* 191.1035.; $[\boldsymbol{\alpha}]_{\boldsymbol{D}}^{\mathbf{20}}$ = –62.9 (*c* 0.27, $CHCl_3$).

Synthesis and characterisation of 41

benzyl (3R,4S)-5,5-dimethyl-1-oxaspiro[2.2]pentane-4-carboxylate (41)

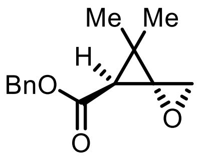

*m*CPBA (77%, 31 mg, 0.15 mmol, 1.5 equiv.) was added to a solution of **22b** (21.6 mg, 0.1 mmol, with 90% ee) in CH_2Cl_2 (2 ml). The reaction mixture was stirred at room temperature for 2 hours, at which point the second portion of *m*CPBA (77%, 31 mg, 0.15 mmol, 1.5 eq.) was added and the reaction mixture allowed to stir at room temperature for an additional 2 hours. Upon completion, sat. aq. $NaHCO_3$ (5 mL) was added. The aqueous layer was extracted with CH_2Cl_2 (3 x 5 ml) and the combined organic layers were washed with brine, dried over Na_2SO_4 and concentrated *in vacuo* to give the crude product in >20:1 d.r.. The crude reaction mixture was purified by silica gel column chromatography (pentane/EtOAc = 20/1) to provide **41** as colourless oil in 50% yield (11.6 mg) and 90% ee. [determined by HPLC chiralpak IA, hexane/isopropanol = 98/2, 1 ml/min, λ = 220 nm, t(major) = 6.72 min, t(minor) = 12.89 min]. **^{1}H NMR** (400 MHz, $CDCl_3$) δ(ppm): 7.41 – 7.30 (m, 5H, ArH), 5.11 (s, 2H, PhCH_2), 3.31 (s, 2H, C(O)CH_2), 2.08 (s, 1H, C$H$$CO_2$Bn), 1.35 (s, 3H, C$(CH_3)_A(CH_3)_B$), 1.30 (s, 3H, C$(CH_3)_A(CH_3)_B$); **^{13}C NMR** (101 MHz, $CDCl_3$) δ(ppm): 170.2 (C=O), 136.1 (ArC), 128.7 (ArCH), 128.4 (ArCH), 128.4 (ArCH), 66.5 (PhCH_2O), 65.7 (C(O)CH_2), 49.0 (C(O)CH_2), 29.9 (CHC(=O)), 26.4 (CMe_2), 21.7(one of C$(CH_3)_A(CH_3)_B$), 16.7 (one of C$(CH_3)_A(CH_3)_B$); **HRMS** (ES+) exact mass calculated for $[M+Na]^+$ ($C_{14}H_{17}O_3^+$) requires *m/z* 233.1172, found *m/z* 233.1175. ; $[\alpha]_D^{20}$ = –18.9 (*c* 0.44, $CHCl_3$).

8. Kinetic Isotope Effect Studies

Synthesis and characterisation of S30-d_2

2-bromo-1-((2-methoxyethoxy)methoxy)-3-methylbut-2-ene-1,1-d_2 (S30-d_2)

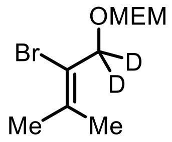

i. To a solution of 3-methylbut-2-enoic acid (1.0 g, 10 mol, 1.0 equiv.) in THF (50 mL) was added $LiAlD_4$ (760 mg, 20 mmol, 2.0 equiv.) in an ice bath portionwise. The reaction mixture was stirred at 0 °C overnight. The reaction was quenched at 0 °C with water (0.76 mL)/NaOH (15%, 0.76 mL)/water (2.28 mL), and then warmed to room temperature. Anhydrous $MgSO_4$ was added and the mixture filtered. The filtrate was concentrated under reduced pressure in an ice bath to give the crude as colourless oil (792 mg), which was used in the next step without further purification.

ii. Bromine (0.49 mL, 9.45 mmol, 1.05 eq) was added dropwise to a stirred solution of crude material from step ii in CH_2Cl_2 (15 ml). The solution was allowed to warm to room temperature overnight at which point DBU (2.12 ml, 14.2 mmol, 1.58 eq) was added. The solution was heated to reflux for 4 h. The reaction mixture was allowed to cool to room temperature and sat. aq. sodium thiosulfate was added (20 ml). The phases were separated, and the aqueous layer was extracted with Et_2O (3 x 10 ml). The combined organic layers were washed with 1.0 M $HCl_{(aq)}$ (3 x 10 ml), sat. aq. $NaHCO_3$ (10 ml) and brine then dried over Na_2SO_4, filtered and concentrated *in vacuo*. The crude product (901 mg) was taken on without further purification.

iii. DIPEA (1.0 ml, 11.25 mmol, 1.25 eq) was added to a stirred solution of the crude product from step ii and MEMCl (0.72 ml, 13.5 mmol, 1.50 eq) in CH_2Cl_2 (5 ml) at 0 °C. The reaction was allowed to warm to room temperature overnight at which point H_2O was added (10 ml) and the layers separated. The aqueous layer was extracted with CH_2Cl_2 (2 x 10 ml). The

combined organic layers were washed with brine, dried over Na_2SO_4, filtered and concentrated *in vacuo*. The crude product was purified by silica gel column chromatography (pentane/Et_2O = 5/1) to provide the title compound (**S30-*d*$_2$**) as a colourless oil (1.12 g, 44% over 3 steps). **^{1}H NMR** (400 MHz, $CDCl_3$) δ(ppm): 4.74 (s, 2H, $OC\underline{H_2}O$), 3.78 – 3.69 (m, 2H, $CD_2OCH_2OC\underline{H_2}CH_2$), 3.62 – 3.54 (m, 2H, $CD_2OCH_2OCH_2C\underline{H_2}$), 3.39 (s, 3H, $OC\underline{H_3}$), 1.91 (s, 3H, one of $C{=}C(C\underline{H_3})_A(CH_3)_B$), 1.87 (s, 3H, one of $C{=}C(CH_3)_A(C\underline{H_3})_B$); **^{13}C NMR** (101 MHz, $CDCl_3$) δ(ppm): 137.5 ($\underline{C}{=}CBr$), 116.7 ($C{=}\underline{C}Br$), 94.1 ($O\underline{C}H_2O$), 71.9 ($CD_2OCH_2OCH_2\underline{C}H_2$), 67.1 ($CD_2OCH_2O\underline{C}H_2CH_2$), 59.1 ($O\underline{C}H_3$), 25.6 (one of $C{=}C(\underline{C}H_3)_A(CH_3)_B$), 20.8 (one of $C{=}C(CH_3)_A(\underline{C}H_3)_B$); **HRMS** (ES+) exact mass calculated for $[M+ Na]^+$ ($C_9H_{15}D_2BrO_3Na^+$) requires *m/z* 277.0379, found *m/z* 277.0375.

Synthesis and characterisation of S31-d_2

1,1,2-tribromo-2-(((2-methoxyethoxy)methoxy)methyl-d$_2$)-3,3-dimethylcyclopropane (S31-d$_2$)

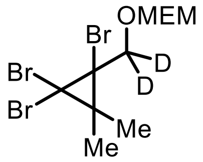

Compound **S31-*d*$_2$** was synthesised according to general procedure **VI** by addition of bromoform (1.15 ml, 13.2 mmol, 3.00 eq) to **S30-*d*$_2$** (1.12 g, 4.4 mmol, 1.00 eq). The reaction mixture was warmed to 50 °C and after 5 days more bromoform (1.15 ml, 13.2 mmol, 3.00 eq) and 50% aq. NaOH (1.15 mL) was added. The reaction was stirred for a further 5 days at which point H_2O (20 ml) was added, the layers were separated and the aqueous phase extracted with CH_2Cl_2 (2 x 10 ml). The combined organic layers were washed with brine, dried over Na_2SO_4, filtered and concentrated *in vacuo*. The crude product was purified by silica gel column chromatography (pentane/Et_2O = 5/1) to provide the title compound (**S31-*d*$_2$**) as a yellow oil in 78% yield (1.46 g). **^{1}H NMR** (400 MHz, $CDCl_3$) δ(ppm): 4.82 (d, *J* = 7.0 Hz, 1H, one of OC$\underline{H_A}$H$_B$O), 4.80 (d, *J* = 7.0 Hz, 1H, one of OCH$_A\underline{H_B}$O), 3.84 – 3.69 (m, 2H, C$\underline{H_2}$CH$_2$OCH$_3$), 3.59 – 3.52 (m, 2H, CH$_2$C$\underline{H_2}$OCH$_3$), 3.38 (s, 3H, CH$_2$CH$_2$OC$\underline{H_3}$), 1.51 (s, 3H, one of C(C$\underline{H_3}$)$_A$(CH$_3$)$_B$), 1.44 (s, 3H, one of C(CH$_3$)$_A$(C$\underline{H_3}$)$_B$); **^{13}C NMR** (101 MHz, $CDCl_3$) δ(ppm): 95.7 (O$\underline{C}$H$_2$O), 71.8 ($\underline{C}$H$_2$OCH$_3$), 67.4 ($\underline{C}$H$_2$CH$_2$OCH$_3$), 59.2 (O$\underline{C}$H$_3$), 53.3 ($\underline{C}$Br$_2$), 48.1 (CD$_2\underline{C}$Br), 33.8 ($\underline{C}$(CH$_3$)$_3$), 26.9 (one of C($\underline{C}$H$_3$)$_A$(CH$_3$)$_B$), 21.1(C(CH$_3$)$_A$($\underline{C}$H$_3$)$_B$); **HRMS** (ES+) exact mass calculated for $[M+Na]^+$ ($C_{10}H_{15}D_2Br_3O_3Na^+$) requires *m/z* 446.8746, found *m/z* 446.8760.

Synthesis and characterisation of 33a-*d*$_2$

benzyl 2-(((2-methoxyethoxy)methoxy)methyl-d$_2$)-3,3-dimethylcycloprop-1-ene-1-carboxylate (33a-d$_2$)

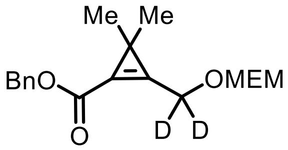

Compound **33a-*d*$_2$** was synthesised according to general procedure **VII** by the addition of tribromocyclopropane **S31-*d*$_2$** (1.46 g, 3.42 mmol, 1.00 eq) to benzyl chloroformate (3 M in toluene, 1.7 ml, 5.13 mmol, 1.50 eq). The crude product was purified by silica gel column chromatography (pentane/EtOAc = 5/1) to provide the title compound (**33a-*d*$_2$**) as a colourless oil in 80% yield (882 mg). **^{1}H NMR** (400 MHz, $CDCl_3$) δ(ppm): 7.44 – 7.28 (m, 5H, Ar$\underline{H}$), 5.23 (s, 2H, PhC$\underline{H_2}$), 4.79 (s, 2H, OC$\underline{H_2}$OCD$_2$C=C), 3.73 – 3.68 (m, 2H, C$\underline{H_2}$OCH$_2$OCD$_2$C=C), 3.55 – 3.51 (m, 2H, C$\underline{H_2}$CH$_2$OCH$_2$OCD$_2$C=C), 3.38 (s, 3H, OC$\underline{H_3}$), 1.29 (s, 6H, C(C$\underline{H_3}$)$_2$); **^{13}C NMR** (101 MHz, $CDCl_3$) δ(ppm): 161.5 ($\underline{C}$=O), 144.1 ($\underline{C}$=CC(=O)), 135.9 (Ar$\underline{C}$), 128.6 (Ar$\underline{C}$H), 128.3 (Ar$\underline{C}$H), 128.2 (Ar$\underline{C}$H), 119.4 (C=$\underline{C}$C(=O)), 94.5 (O$\underline{C}$H$_2$O), 71.8 ($\underline{C}$H$_2$OCH$_3$), 67.2 ($\underline{C}$H$_2$CH$_2$OCH$_3$), 66.6 ($\underline{C}$H$_2$Ph), 59.1 (O$\underline{C}$H$_3$), 26.7 ($\underline{C}$(CH$_3$)$_2$), 25.6 (C($\underline{C}$H$_3$)$_2$); **HRMS** (ES+) exact mass calculated for $[M+Na]^+$ ($C_{18}H_{22}D_2O_5Na^+$) requires *m/z* 345.1641, found *m/z* 345.1640.

Synthesis and characterisation of S30-*d*

2-bromo-1-((2-methoxyethoxy)methoxy)-3-methylbut-2-ene-1-d (S30-d)

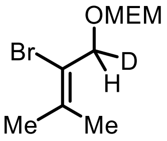

i. To a solution of 3-methylbut-2-enal (0.95 ml, 10 mmol, 1.0 equiv.) in THF/water (10/2 mL) was added $NaBD_4$ (460 mg, 11 mmol, 1.1 equiv.) in an ice bath portionwise. The reaction mixture was stirred at rt for 3 hours. Then anhydrous $MgSO_4$ was added and the mixture filtered. The filtrate was concentrated under reduced pressure in an ice bath to give the crude material as a colourless oil (783 mg), which was used in the next step without further purification.

ii. Bromine (0.54 ml, 10.5 mmol, 1.05 eq) was added dropwise to a stirred solution of crude material from step i in CH_2Cl_2 (17 ml) dropwise at 0 °C. The solution was allowed to warm to room temperature overnight at which point DBU (2.36 ml, 15.8 mmol, 1.58 eq) was added. The solution was heated to reflux for 4 h. The reaction mixture was allowed to cool to room temperature and sat. aq. Sodium thiosulfate was added (20 ml). The phases were separated, and the aqueous layer was extracted with Et_2O (3 x 10 ml). The combined organic layers were washed with 1.0 M $HCl_{(aq)}$ (3 x 10 ml), sat. aq. $NaHCO_3$ (10 ml), brine, dried over Na_2SO_4, filtered and concentrated *in vacuo*. The crude product (896 mg) was taken on without further purification.

iii. DIPEA (1.00 ml, 12.5 mmol, 1.25 eq) was added to a stirred solution crude material from step ii and MEMCl (0.800 ml, 15.0 mmol, 1.50 eq) in CH_2Cl_2 (5.00 ml) at 0 °C. The reaction was allowed to warm to room temperature overnight at which point H_2O was added (10 ml) and the layers separated. The aqueous layer was extracted with CH_2Cl_2 (2 x 10 ml). The combined organic layers were washed with brine, dried over Na_2SO_4, filtered and concentrated *in vacuo*. The crude product was purified by silica gel column chromatography (pentane/Et_2O = 5/1) to provide the title compound (**S30-*d***) as a colourless oil (978 mg, 38% over 3 steps).

^{1}**H NMR** (400 MHz, $CDCl_3$) δ(ppm): 4.74 (s, 2H, $OC\underline{H_2}O$), 4.36 (s, 1H, $C{=}CC\underline{H}DO$), 3.81 – 3.70 (m, 2H, $CHDOCH_2OC\underline{H_2}CH_2$), 3.58 – 3.51 (m, 2H, $CHDOCH_2OCH_2C\underline{H_2}$), 3.39 (s, 3H, $OC\underline{H_3}$), 1.92 (s, 3H, one of $C{=}C(C\underline{H_3})_A(CH_3)_B$), 1.87 (s, 3H, one of $C{=}C(CH_3)_A(C\underline{H_3})_B$).

Synthesis and characterisation of S31-*d*

1,1,2-tribromo-2-(((2-methoxyethoxy)methoxy)methyl-d)-3,3-dimethylcyclopropane (S31-d)

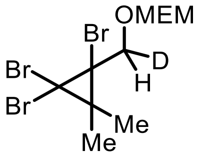

Compound **S31-*d*** was synthesised according to general procedure **VI** by addition of bromoform (1.00 ml, 11.6 mmol, 3.00 eq) to **S30-*d*** (978 mg, 3.87 mmol, 1.00 eq). The reaction mixture was warmed to 50 °C and after 5 days more bromoform (1.00 ml, 11.6 mmol, 3.00 eq) and 50% aq. NaOH (1.00 ml) was added. The reaction was stirred for a further 5 days at which point H_2O (20 ml) was added, the layers were separated and the aqueous phase extracted with CH_2Cl_2 (2 x 10 ml). The combined organic layers were washed with brine, dried over Na_2SO_4, filtered and concentrated *in vacuo*. The crude product was purified by silica gel column chromatography (pentane/Et_2O = 5/1) to provide the title compound (**S31-*d***) as a yellow oil in 73% yield (1.20 g). **^{1}H NMR** (400 MHz, $CDCl_3$) δ(ppm): 4.90 – 4.75 (m, 2H, $OC\underline{H_2}O$), 4.01 (d, *J* = 14.6 Hz, 1H, CHDCBr), 3.84 – 3.70 (m, 2H, $C\underline{H_2}CH_2OCH_3$), 3.56 (t, *J* = 4.6 Hz, 2H, $CH_2C\underline{H_2}OCH_3$), 3.38 (s, 3H, $CH_2CH_2OC\underline{H_3}$), 1.51 (s, 3H, one of $C(C\underline{H_3})_A(CH_3)_B$), 1.44 (s, 3H, one of $C(CH_3)_A(C\underline{H_3})_B$); **^{13}C NMR** (101 MHz, $CDCl_3$) δ(ppm): 95.7 ($O\underline{C}H_2O$), 72.9 (td, *J* = 22.7, 3.15 Hz, O$\underline{C}$HDCBr), 71.8 ($\underline{C}H_2OCH_3$), 67.4 ($\underline{C}H_2CH_2OCH_3$), 59.2 ($O\underline{C}H_3$), 53.4 ($\underline{C}Br_2$), 48.1 (d, *J* = 1.9 Hz, CHD$\underline{C}$Br), 33.8 (d, *J* = 3.2 Hz, $\underline{C}(CH_3)_3$), 27.0 (one of $C(\underline{C}H_3)_A(CH_3)_B$), 21.1 ($C(CH_3)_A(\underline{C}H_3)_B$); **HRMS** (ES+) exact mass calculated for $[M+ K]^+$ ($C_{10}H_{16}DBr_3O_3K^+$) requires *m/z* 461.8422, found *m/z* 461.8415.

Synthesis and characterisation of 33a-*d*

benzyl 2-(((2-methoxyethoxy)methoxy)methyl-d)-3,3-dimethylcycloprop-1-ene-1-carboxylate (33a-d)

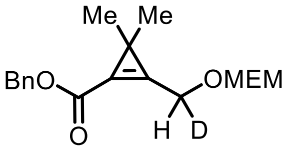

Compound **33a-*d*** was synthesised according to general procedure **VII** by the addition of tribromocyclopropane **S31-*d*** (1.20 g, 2.81 mmol, 1.00 eq) to benzyl chloroformate (3.0 M in toluene, 1.40 ml, 4.20 mmol, 1.50 eq). The crude product was purified by silica gel column chromatography (pentane/EtOAc = 5/1) to provide the title compound (**33a-*d***) as a colourless oil in 45% yield (407 mg). **^{1}H NMR** (400 MHz, $CDCl_3$) δ(ppm): 7.43 – 7.30 (m, 5H, Ar<u>H</u>), 5.23 (s, 2H, PhC<u>H$_2$</u>), 4.79 (s, 2H, OC<u>H$_2$</u>OCH_2C=C), 4.69 (t, J = 2.5 Hz, 1H, OCH_2OC<u>H</u>DC=C), 3.77 – 3.65 (m, 2H, C<u>H$_2$</u>OCH_2OCHDC=C), 3.62 – 3.49 (m, 2H, C<u>H$_2$</u>CH_2OCH_2OCHDC=C), 3.38 (s, 3H, OC<u>H$_3$</u>), 1.29 (s, 6H, C(C<u>H$_3$</u>)$_2$); **^{13}C NMR** (101 MHz, $CDCl_3$) δ(ppm): 161.6 (<u>C</u>=O), 144.1 (<u>C</u>=CC(=O)), 136.0 (Ar<u>C</u>), 128.7 (Ar<u>C</u>H), 128.3 (Ar<u>C</u>H), 128.2 (Ar<u>C</u>H), 119.3 (C=<u>C</u>C(=O)), 94.6 (O<u>C</u>H$_2$O), 71.9 (<u>C</u>H$_2$OCH_3), 67.3 (<u>C</u>H$_2$CH_2OCH_3), 66.6 (<u>C</u>H$_2$Ph), 61.8 (t, J = 2.5 Hz, <u>C</u>HDC=C), 59.1 (O<u>C</u>H$_3$), 26.7 (<u>C</u>(CH_3)$_2$), 25.6 (C(<u>C</u>H$_3$)$_2$); **HRMS** (ES+) exact mass calculated for $[M+ Na]^+$ ($C_{18}H_{23}DO_5Na^+$) requires *m/z* 322.1759, found *m/z* 322.1745.

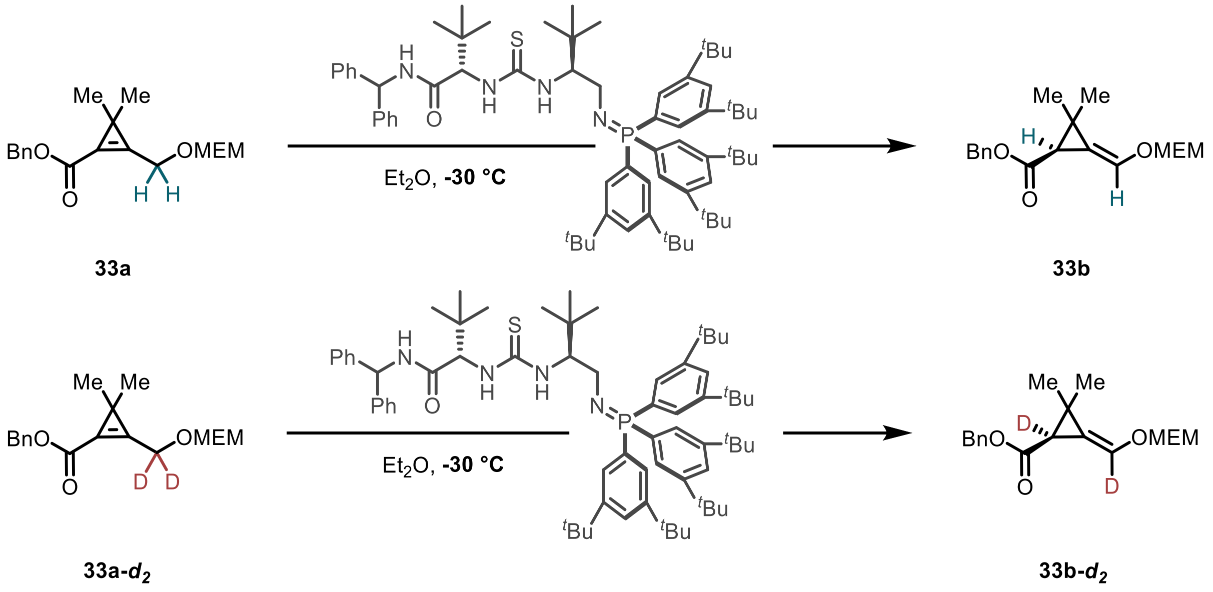

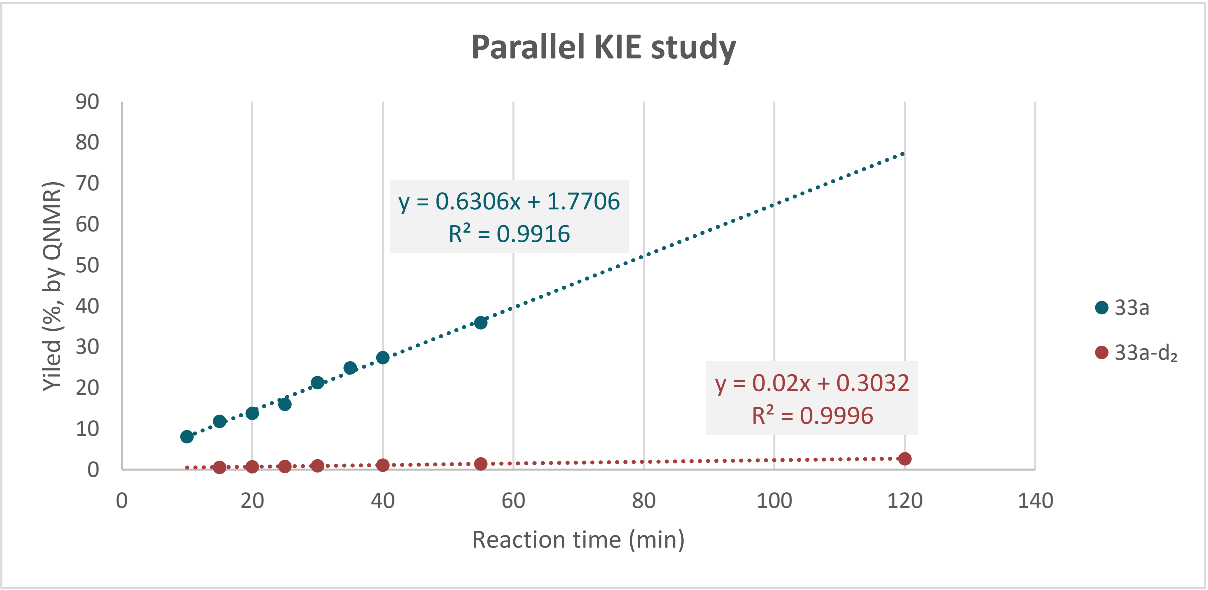

Figure S3: Plot of yield vs. reaction time for substrate **33a** and **33a-*d*$_2$**. Observed KIE = 31.6.

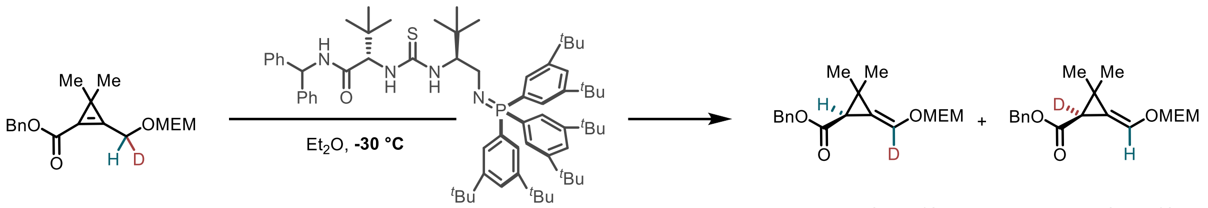

33a-*d* **33b-*d*-1 (H-shift)** **33b-*d*-2 (D-shift)**

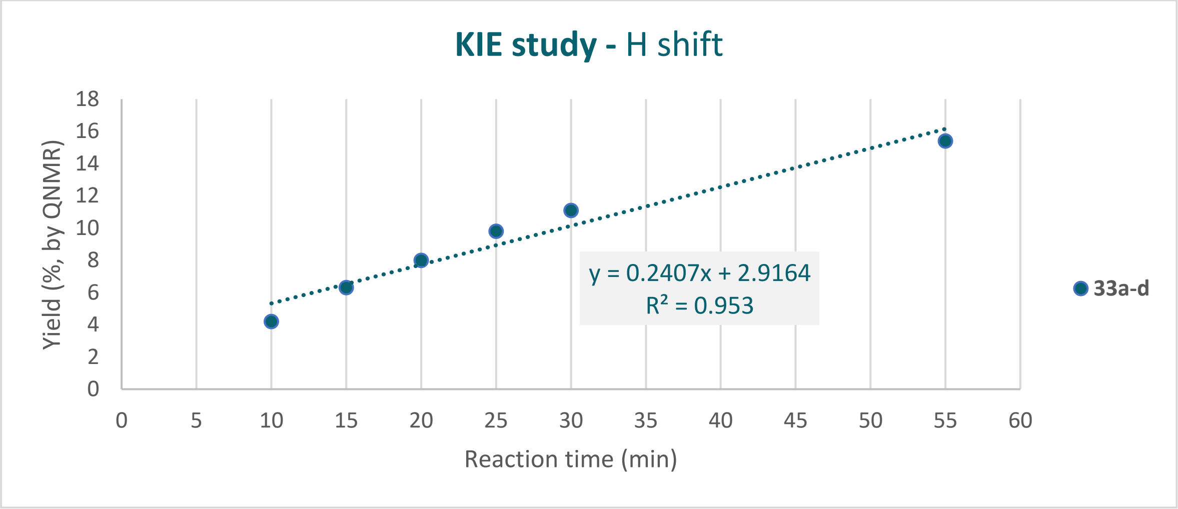

Figure S4: Plot of yield of α-protonated **33b-*d*** (H-shift) vs. reaction time for substrate **33a-*d***.

Observed intramolecular KIE = 7.4

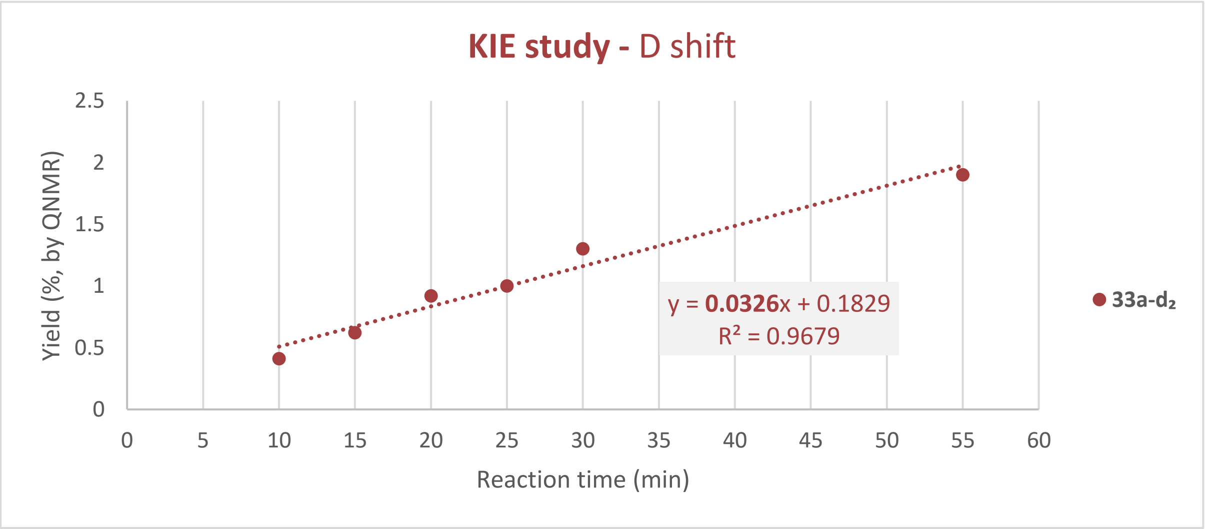

Figure S5: Plot of yield of α-deuterated **33b-*d*** (D-shift) vs. reaction time for substrate **33a-*d***.

Observed intramolecular KIE = 7.4

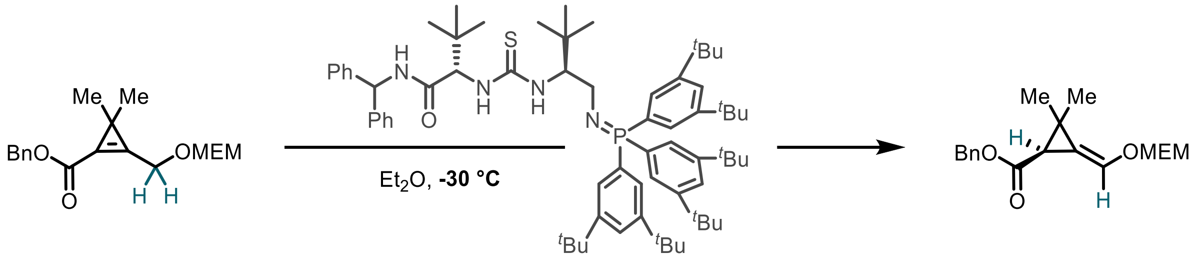

33a **33b**

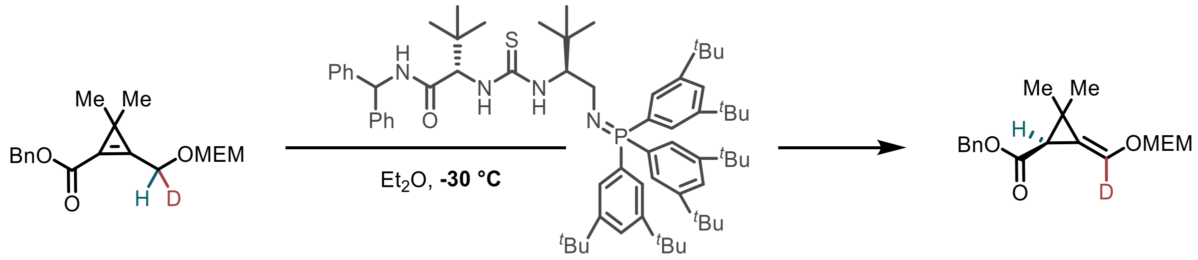

33a-*d* **33b-*d*-1**

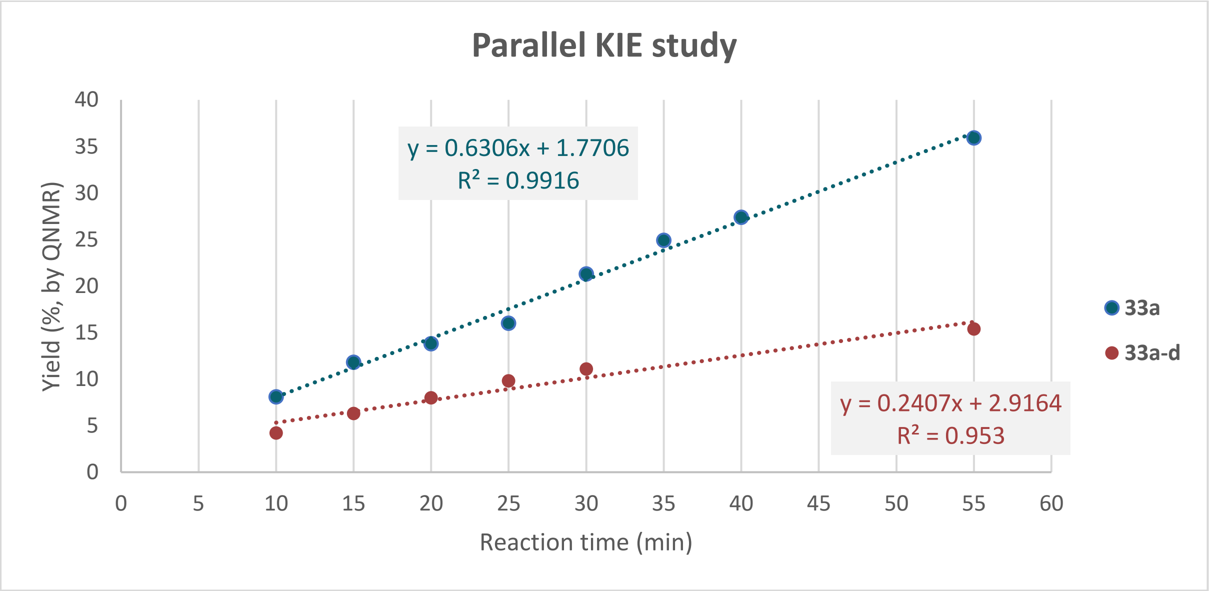

Figure S6: Plot of yield vs. reaction time for substrate **33a** and **33a-*d*** (α-protonated **33b-*d*** product). Observed KIE = 2.6.

9. NMR Spectra

^{1}H NMR (400 MHz, $CDCl_3$) of **S9**

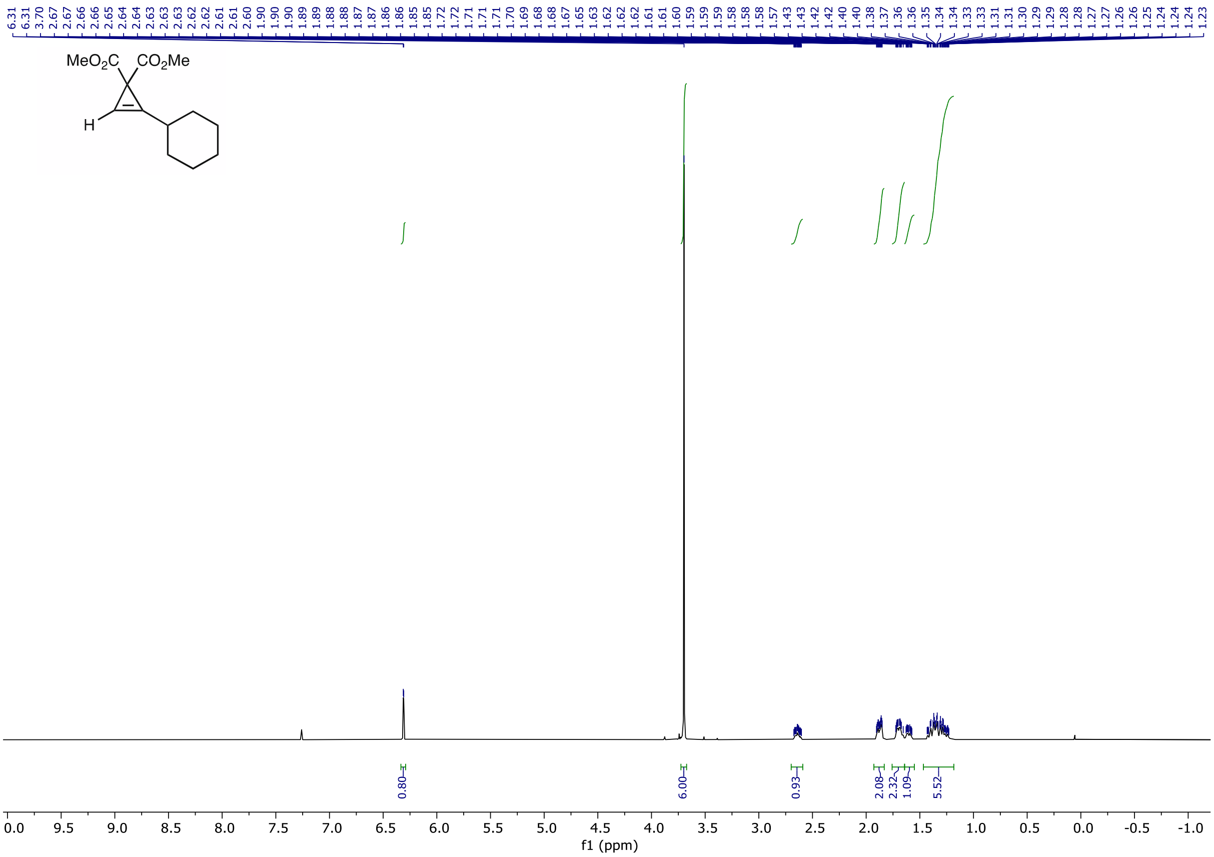

^{13}C NMR (101 MHz, $CDCl_3$) of **S9**

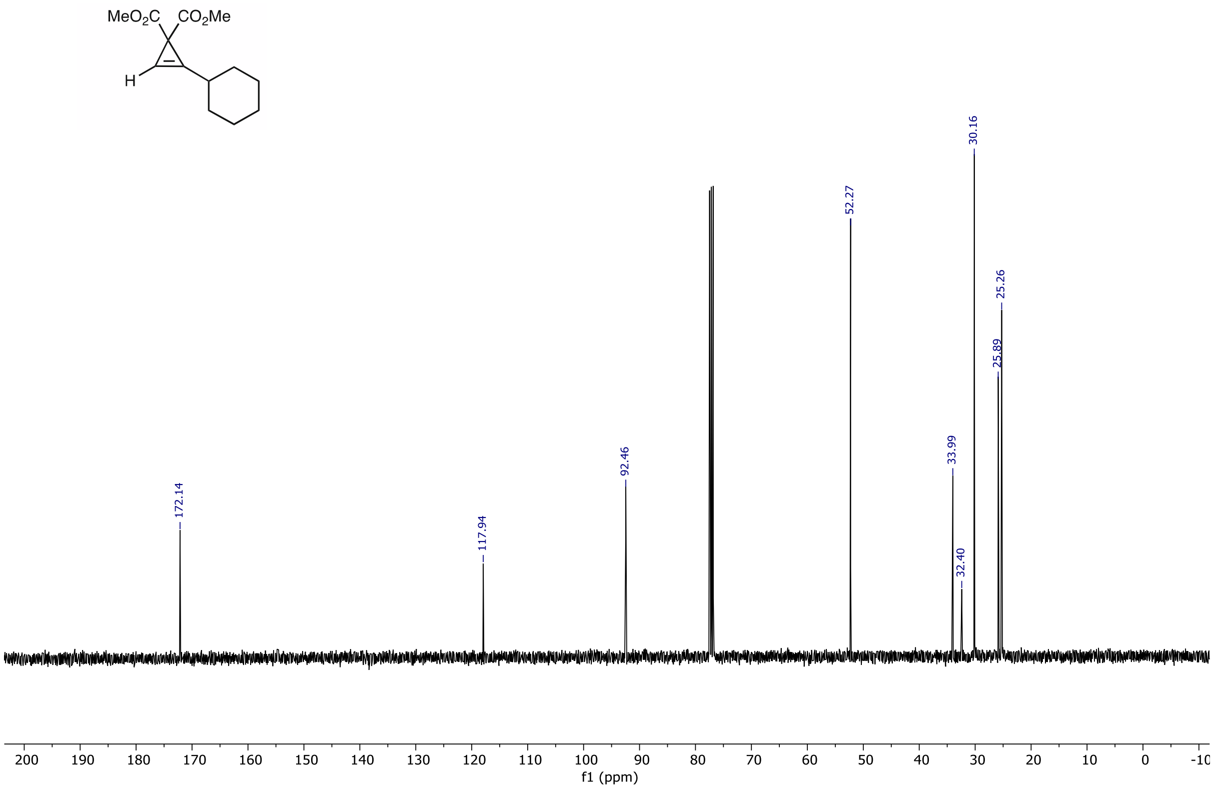

^{1}H NMR (400 MHz, $CDCl_3$) of **S10**

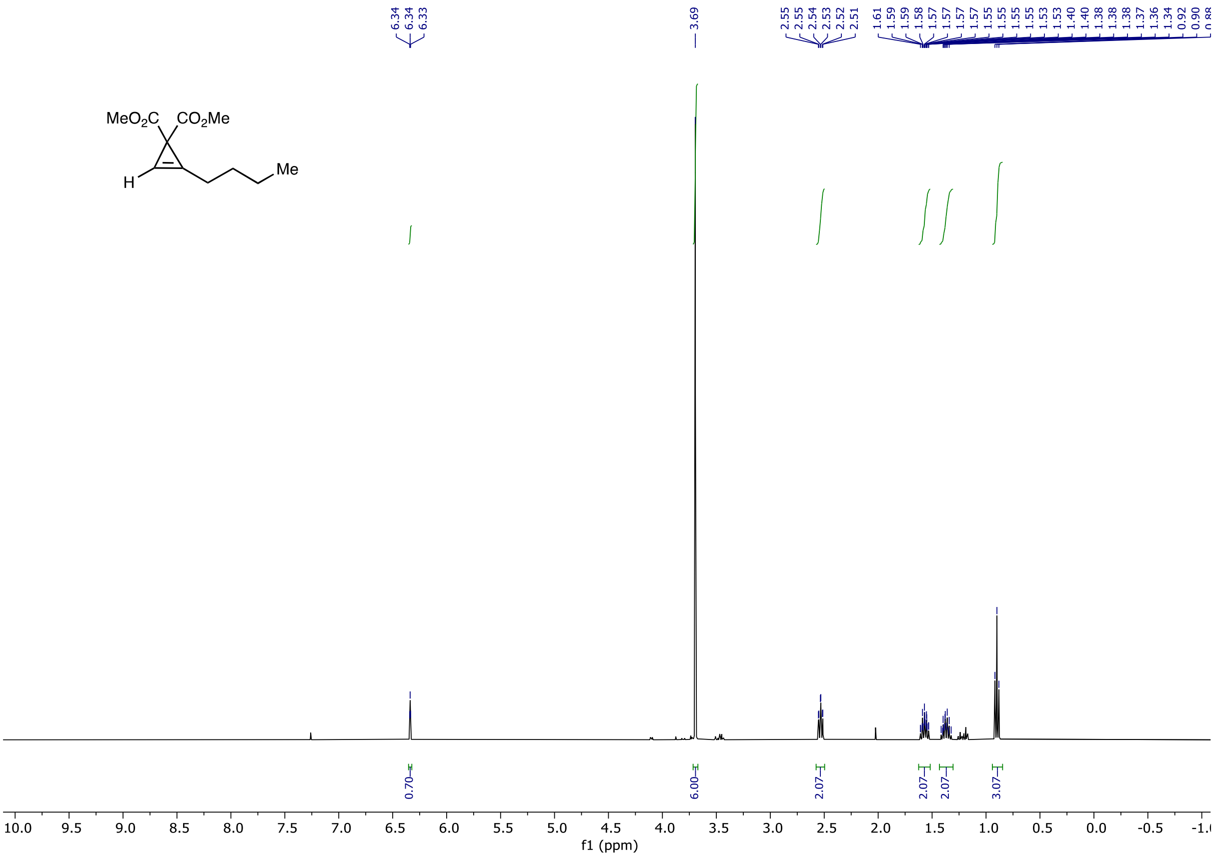

^{13}C NMR (101 MHz, $CDCl_3$) of **S10**

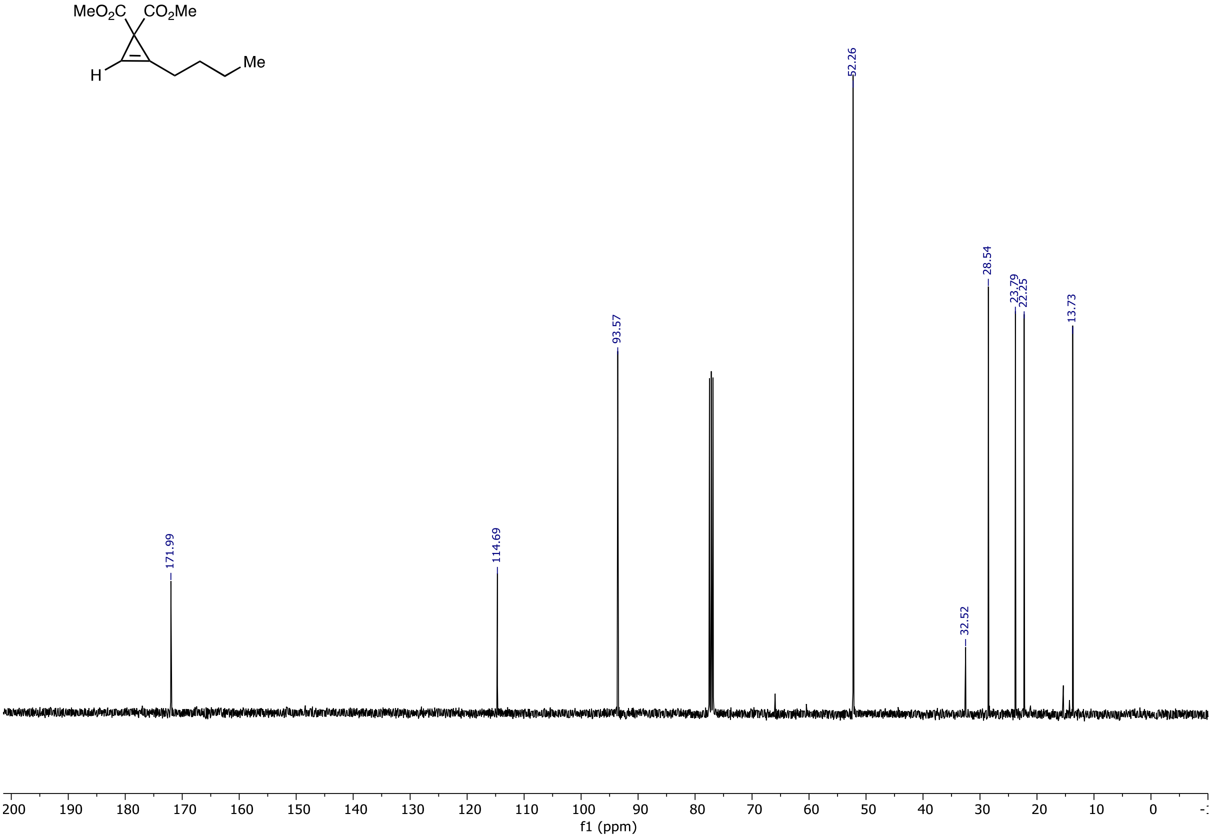

^{1}H NMR (400 MHz, $CDCl_3$) of **S11**

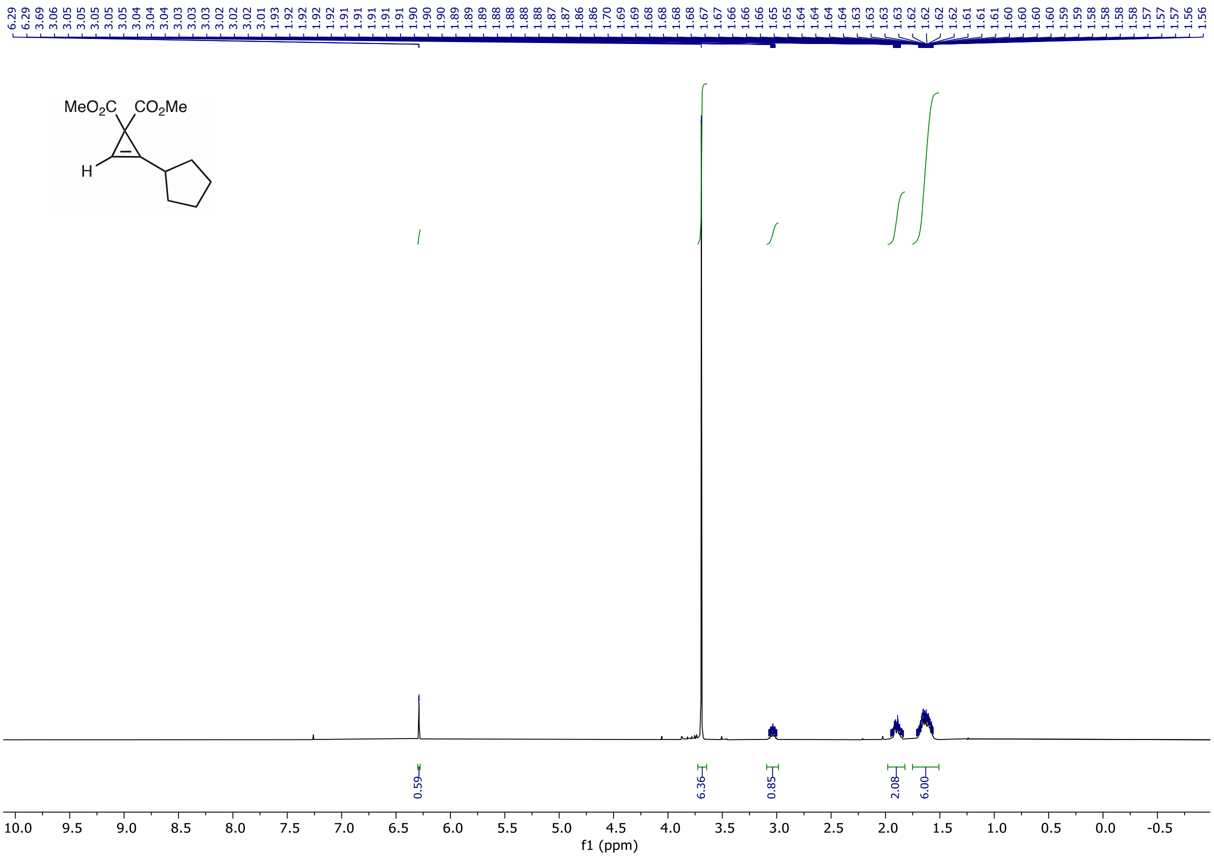

^{13}C NMR (101 MHz, $CDCl_3$) of **S11**

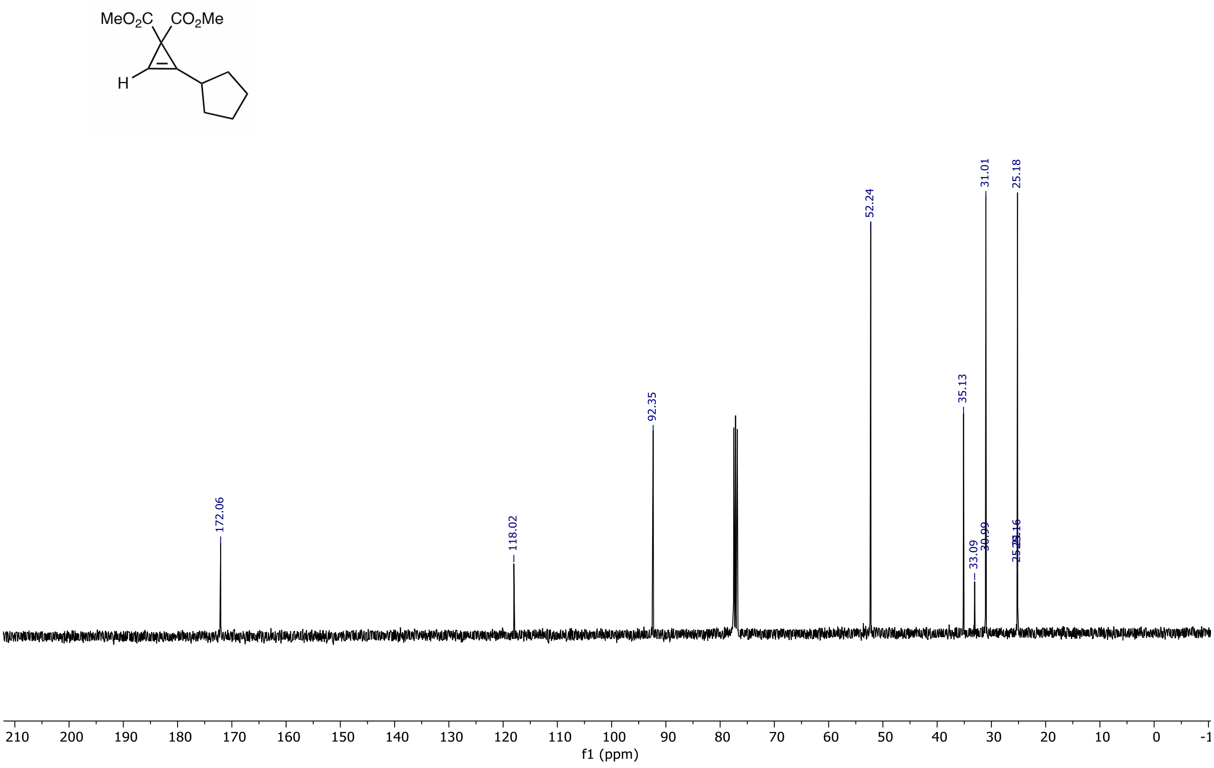

^{1}H NMR (400 MHz, $CDCl_3$) of **S12**

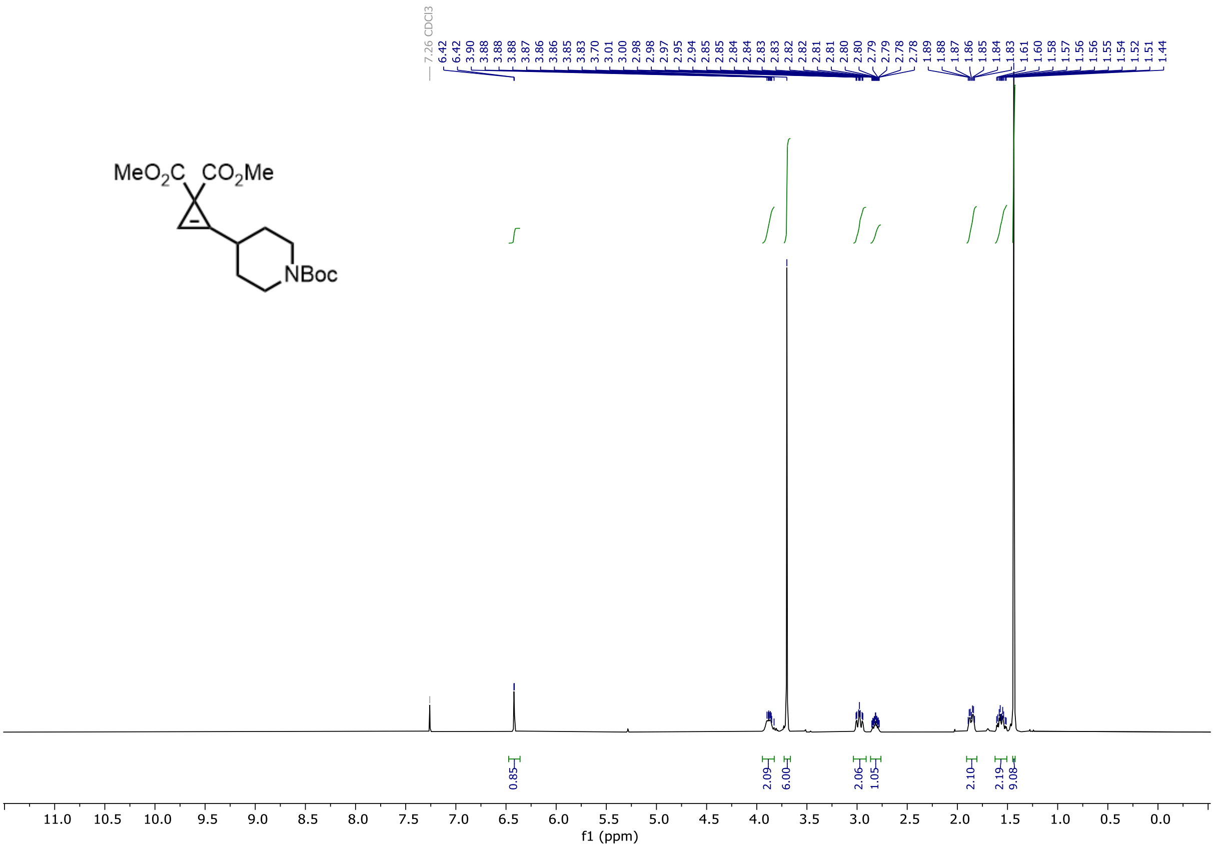

^{13}C NMR (101 MHz, $CDCl_3$) of **S12**

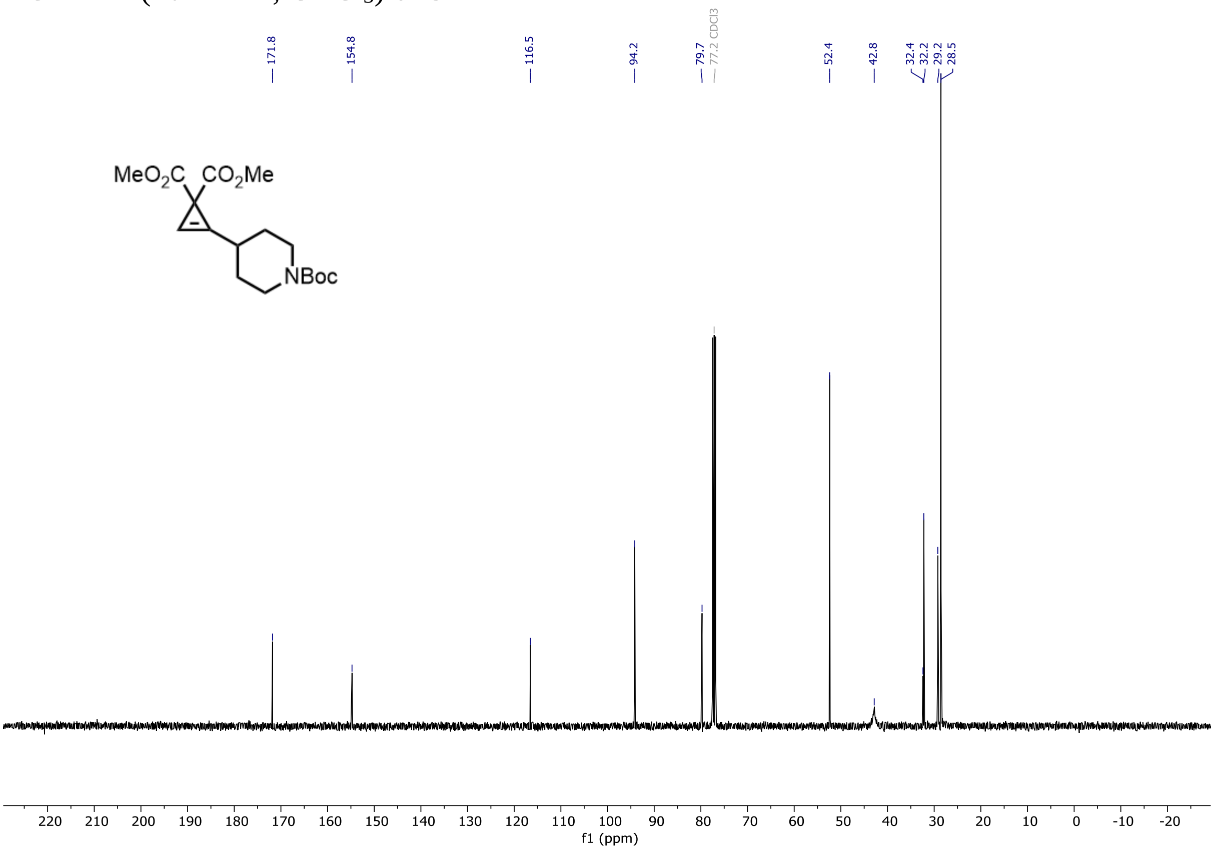

^{1}H NMR (400 MHz, $CDCl_3$) of **S13**

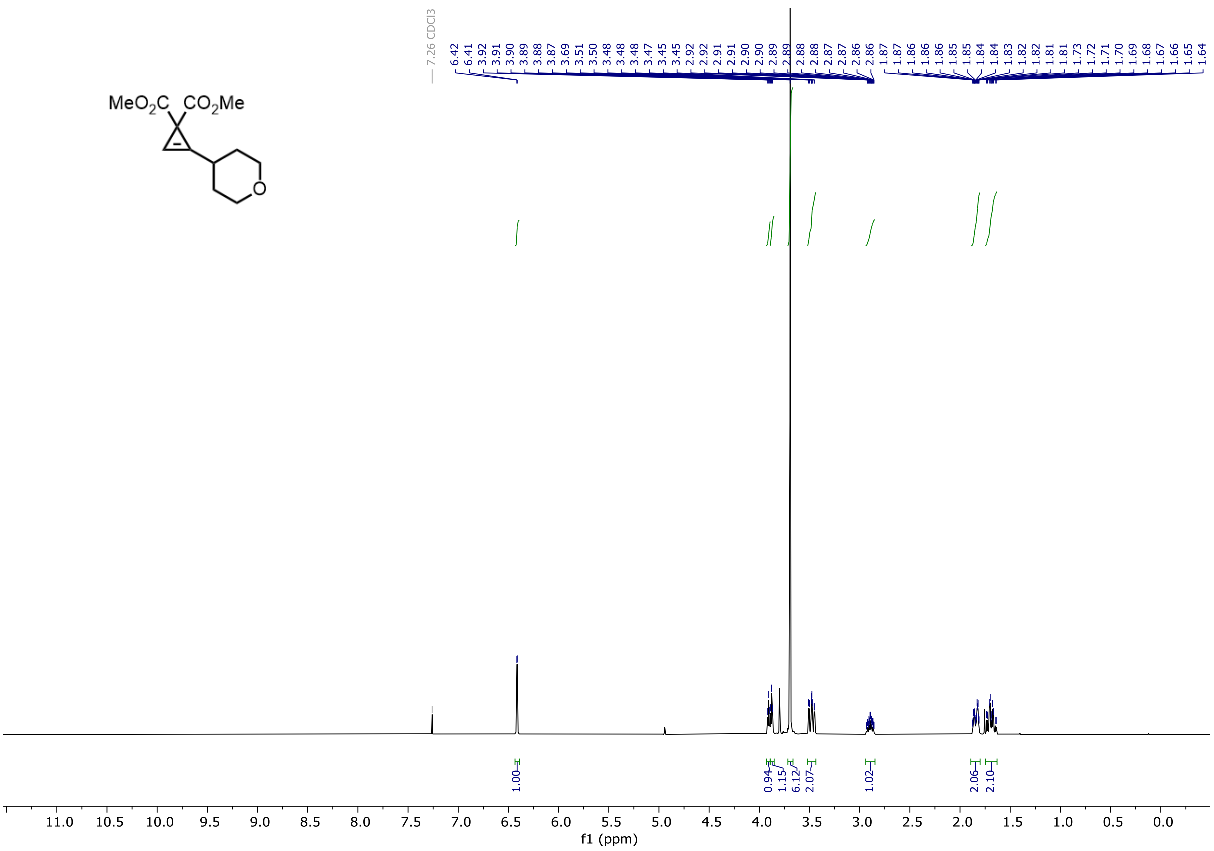

^{13}C NMR (101 MHz, $CDCl_3$) of **S13**

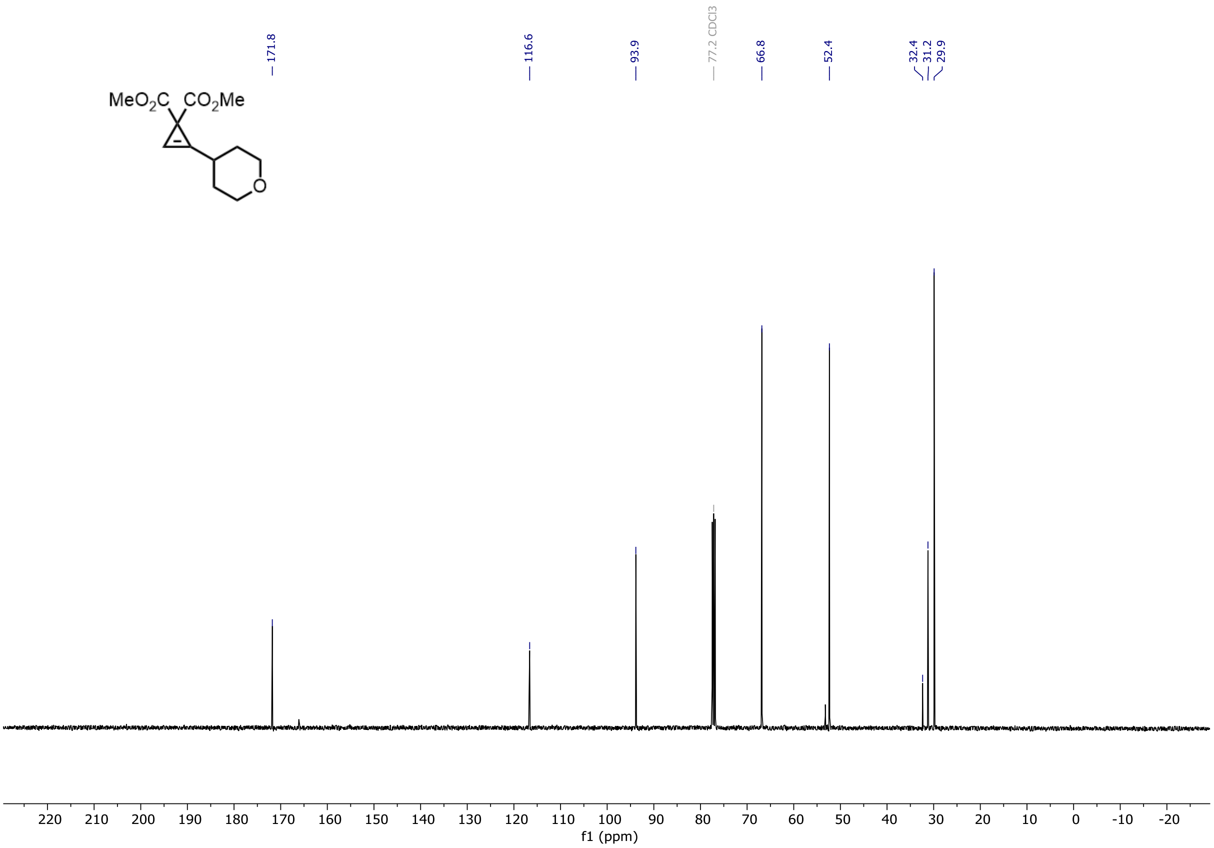

^{1}H NMR (400 MHz, $CDCl_3$) of **S14**

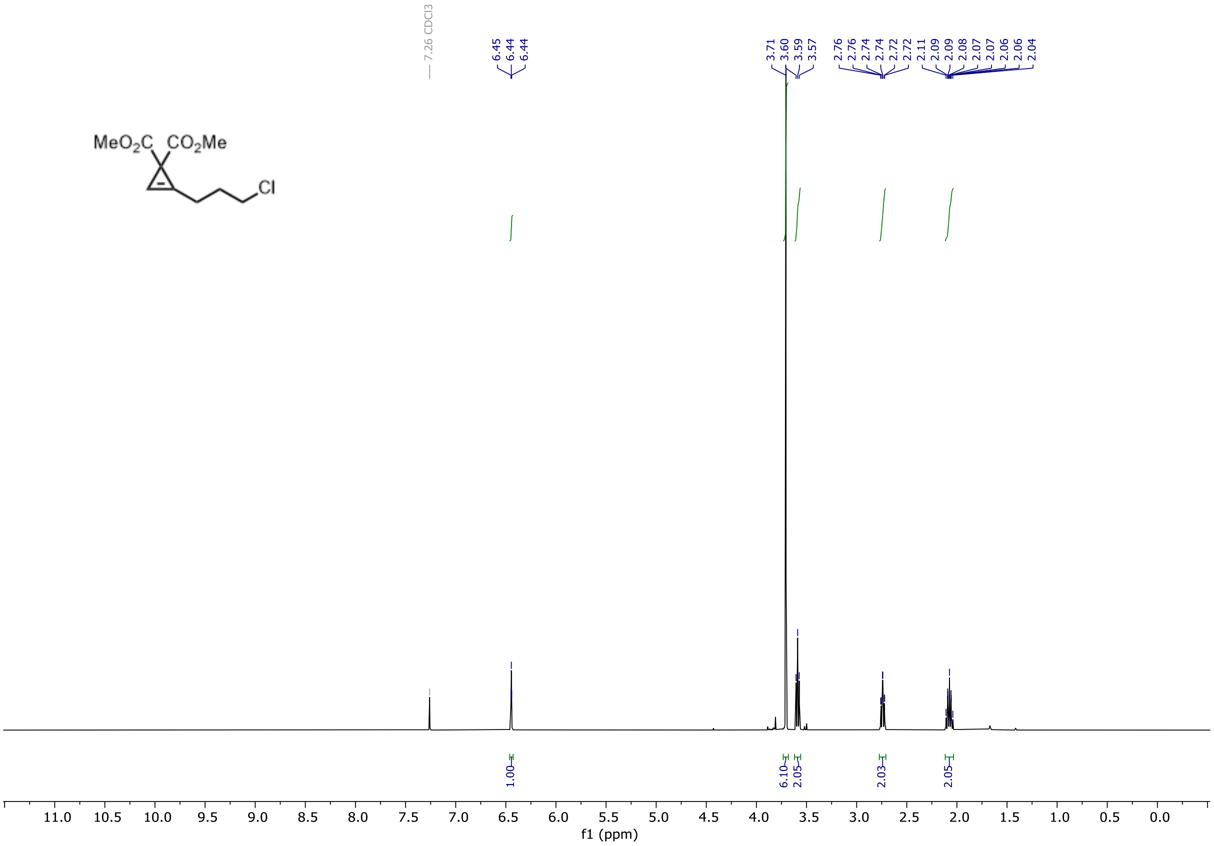

^{13}C NMR (101 MHz, $CDCl_3$) of **S14**

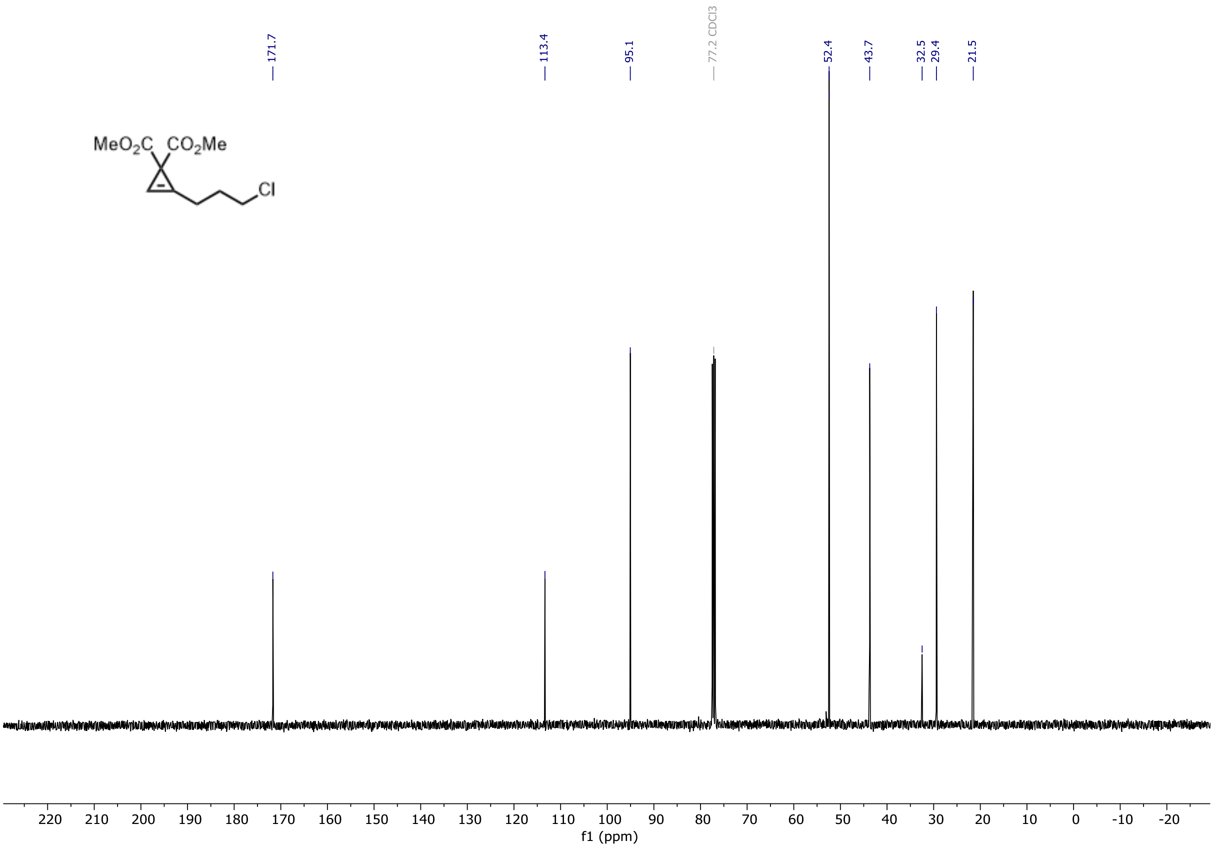

^{1}H NMR (400 MHz, $CDCl_3$) of **S15**

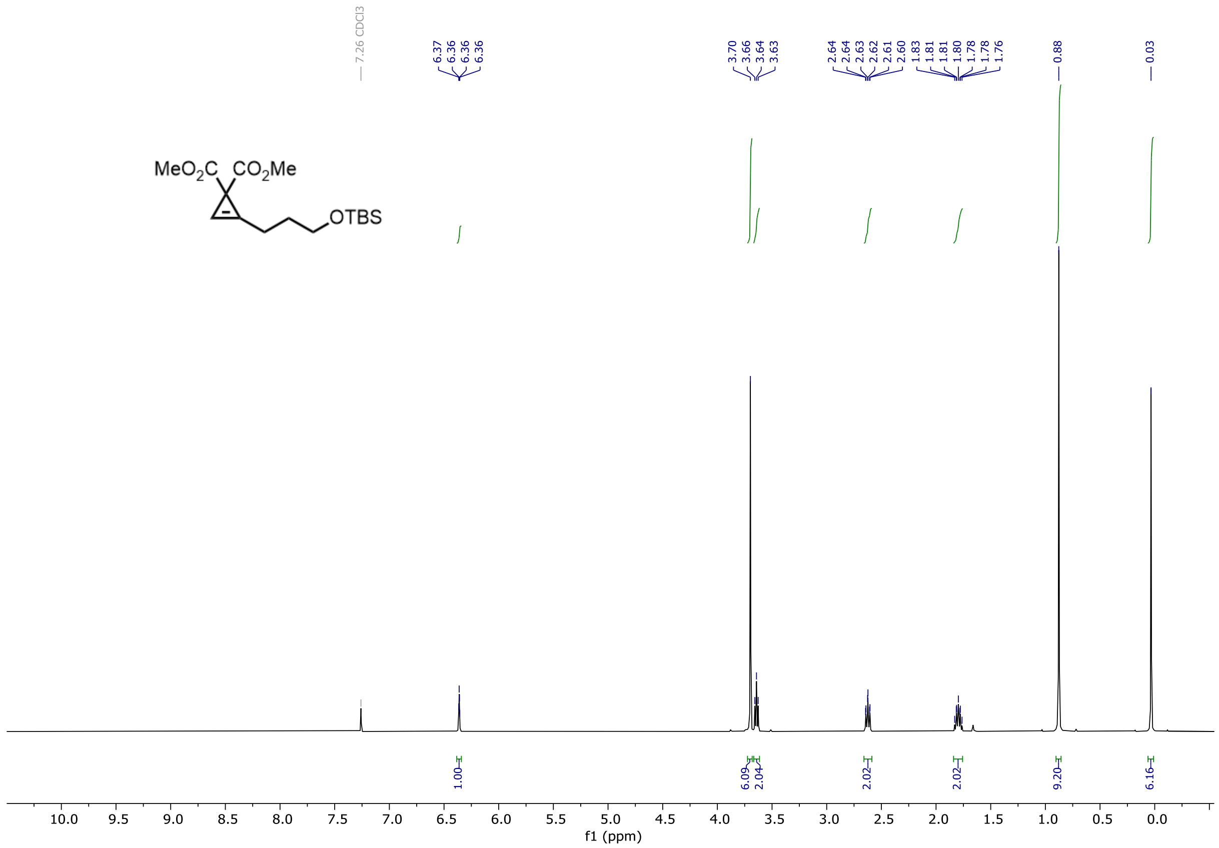

^{13}C NMR (101 MHz, $CDCl_3$) of **S15**

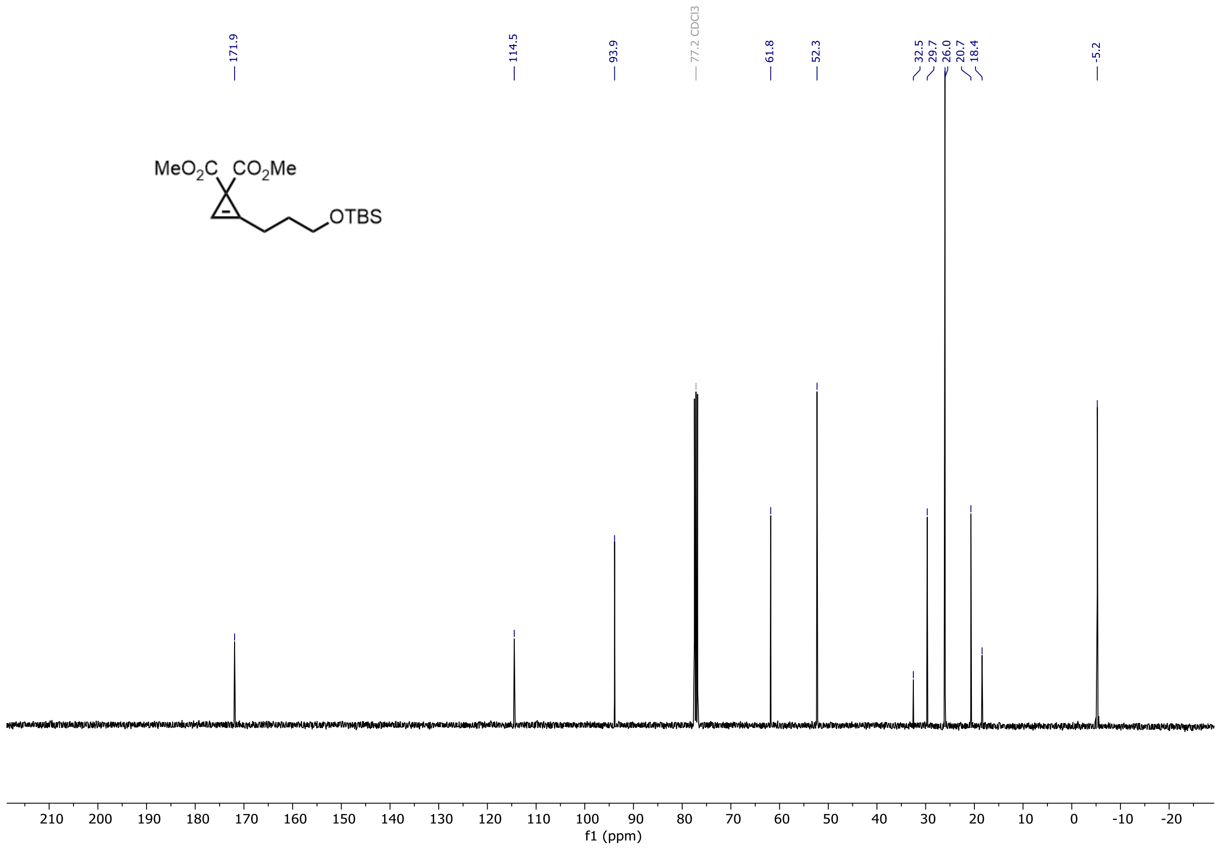

^{1}H NMR (400 MHz, $CDCl_3$) of **S16**

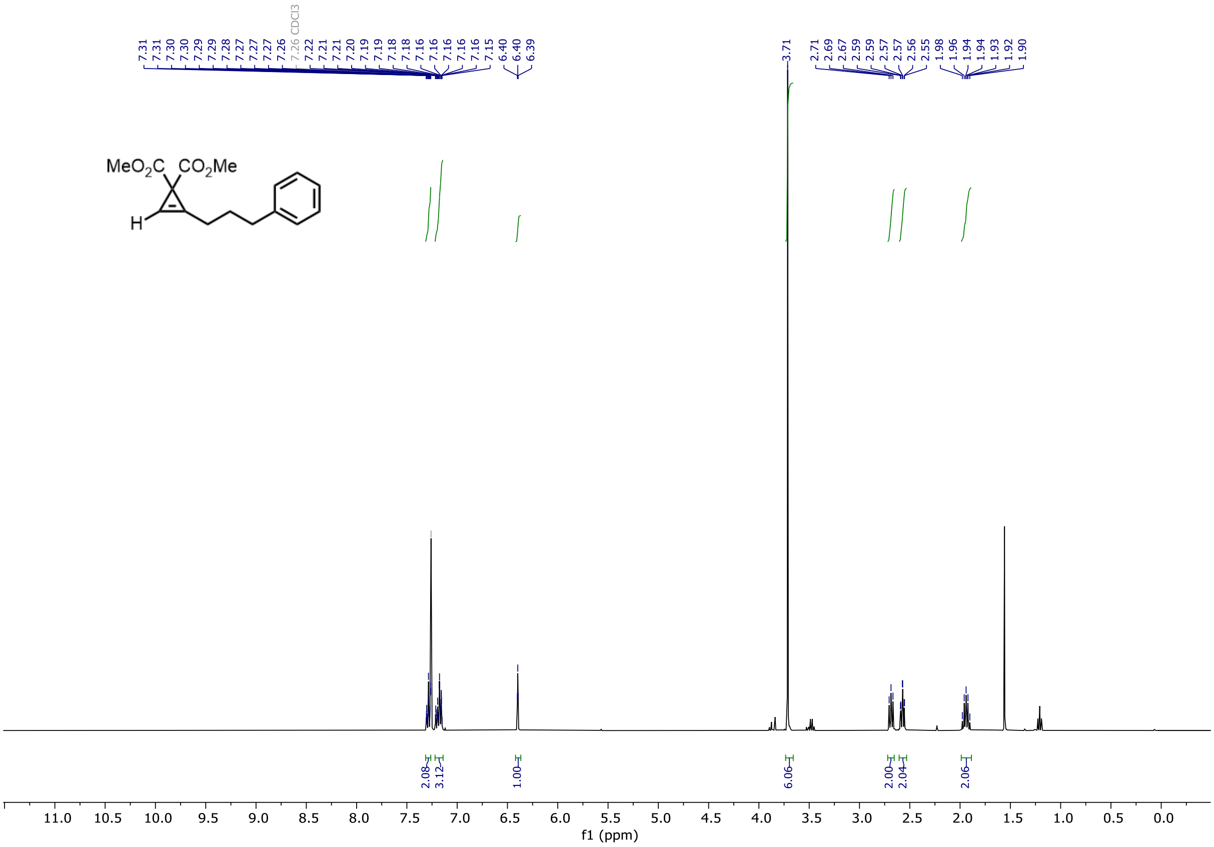

^{13}C NMR (101 MHz, $CDCl_3$) of **S16**

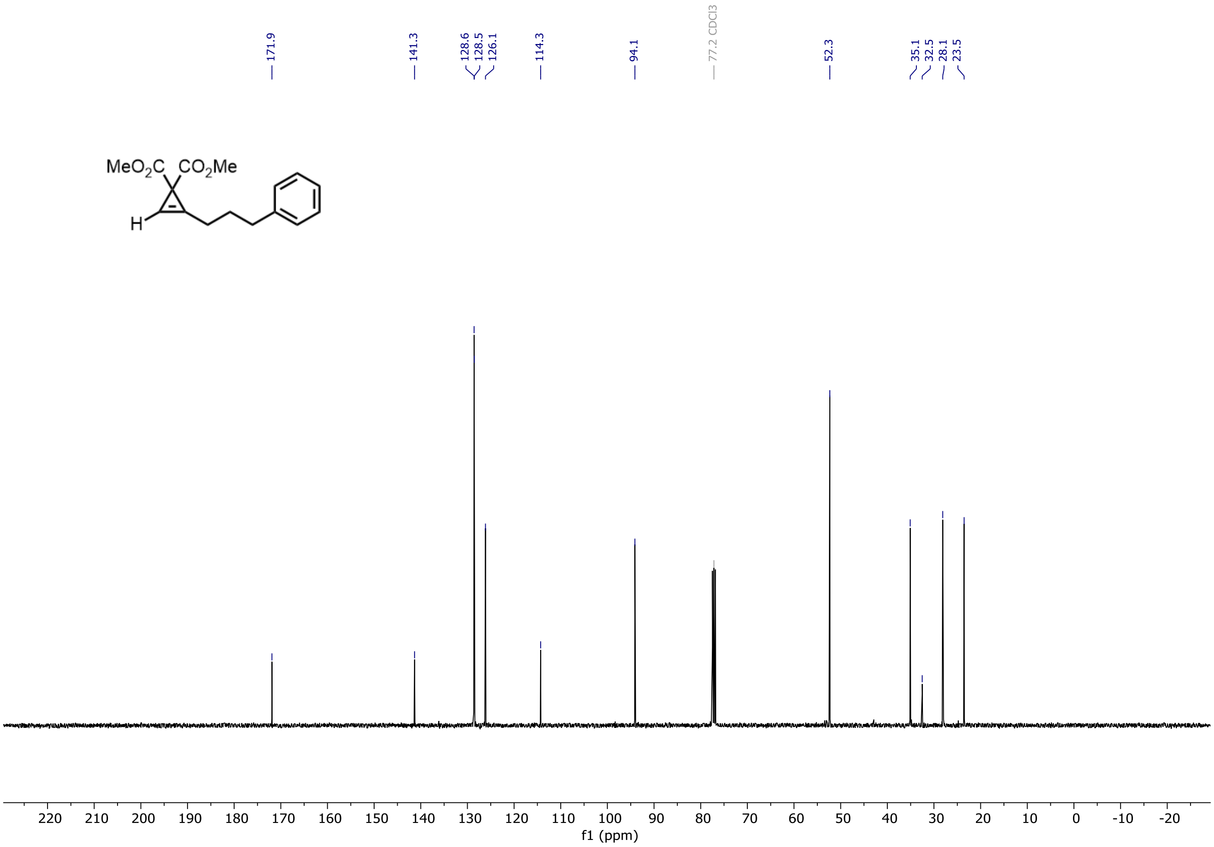

^{1}H NMR (400 MHz, $CDCl_3$) of **S17**

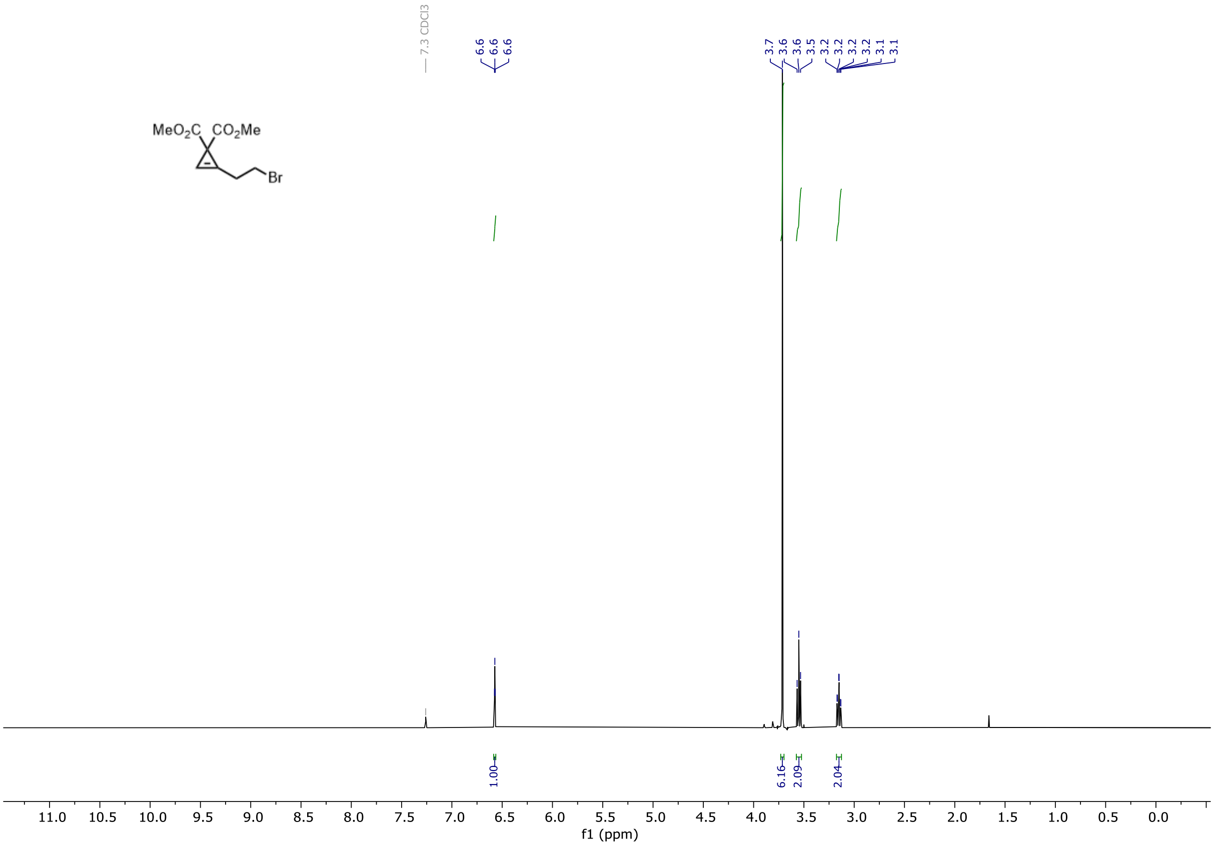

^{13}C NMR (101 MHz, $CDCl_3$) of **S17**

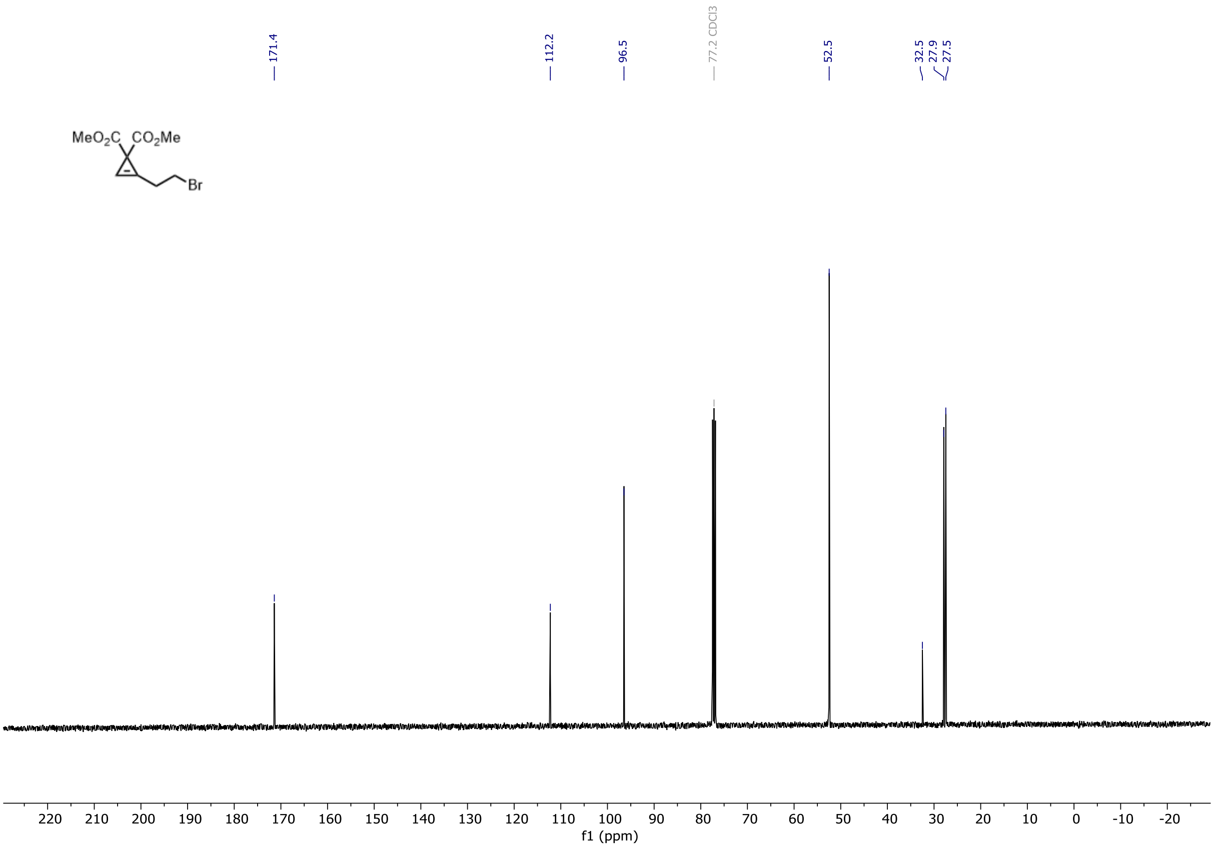

^{1}H NMR (400 MHz, $CDCl_3$) of **S18**

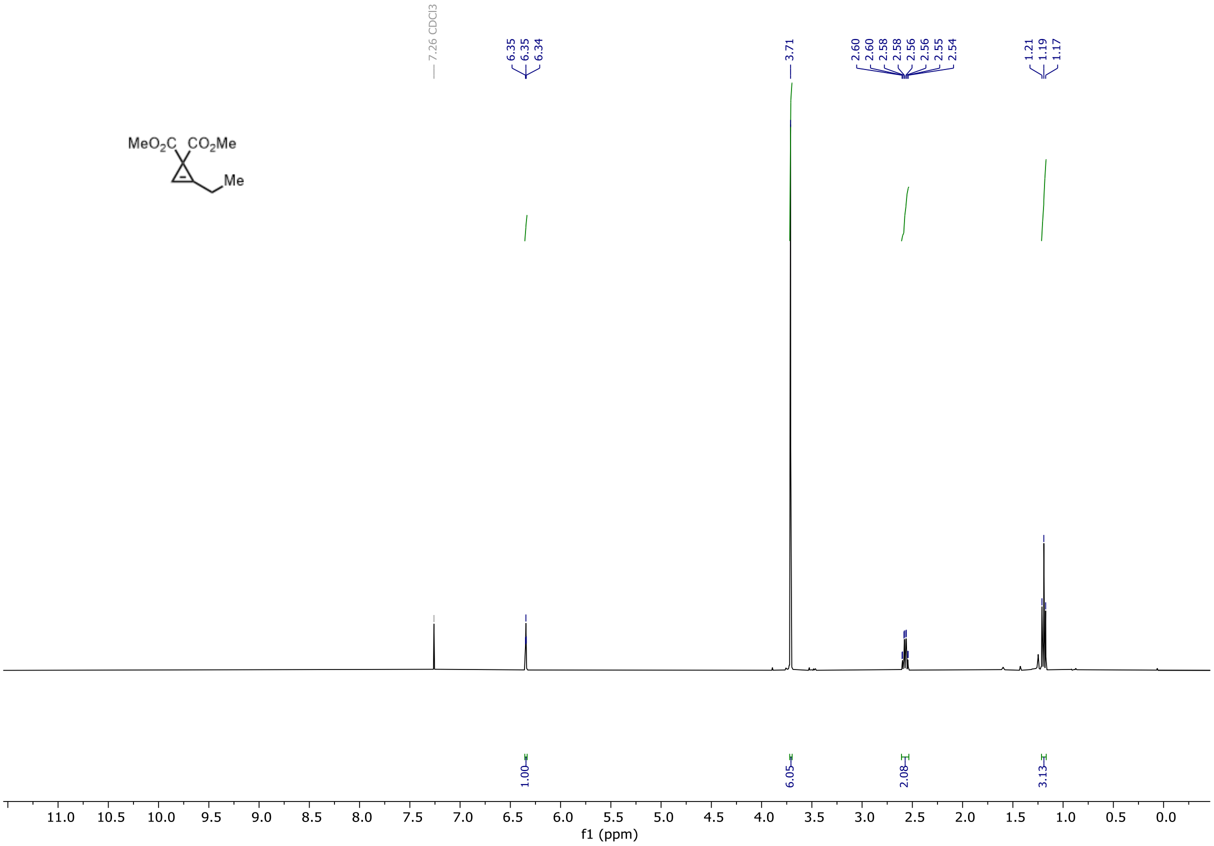

^{13}C NMR (101 MHz, $CDCl_3$) of **S18**

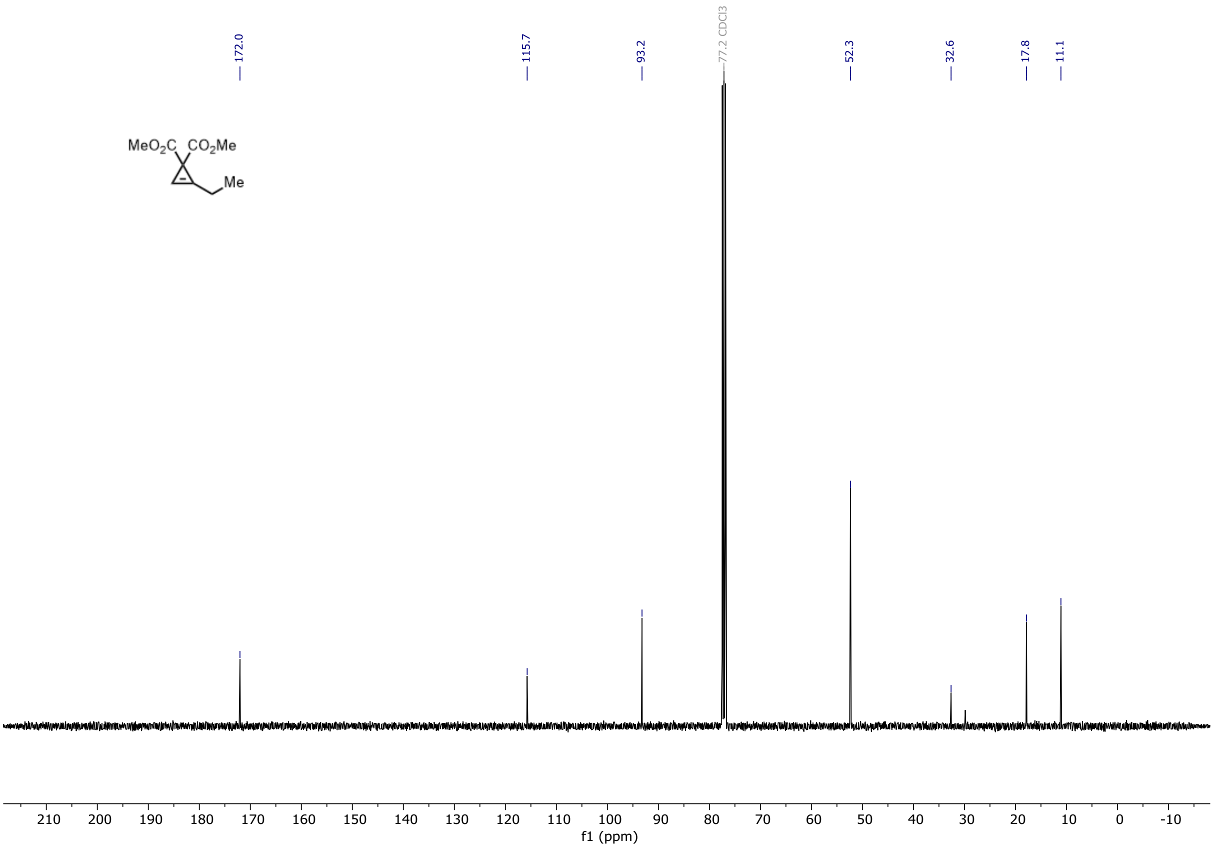

^{1}H NMR (400 MHz, $CDCl_3$) of **S19**

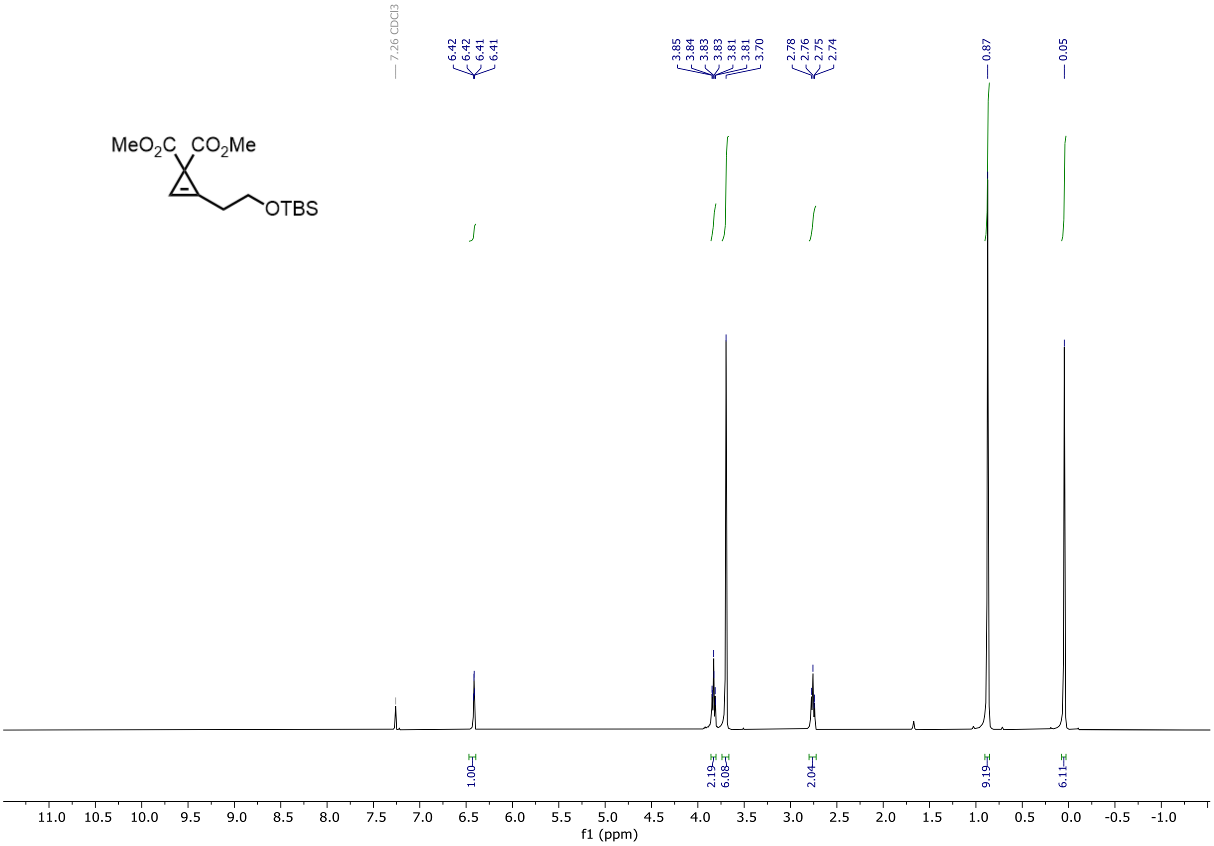

^{13}C NMR (101 MHz, $CDCl_3$) of **S19**

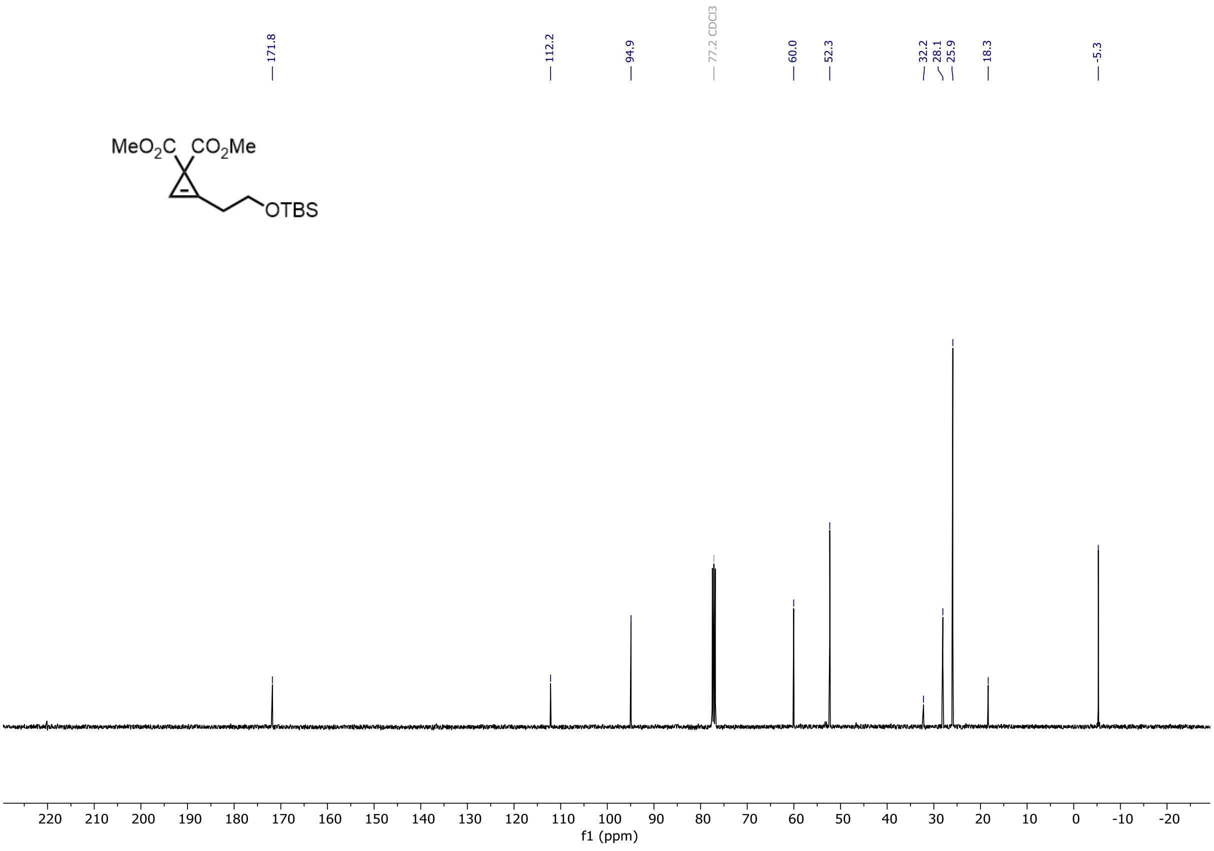

^{1}**H NMR** (400 MHz, $CDCl_3$) of **S20**

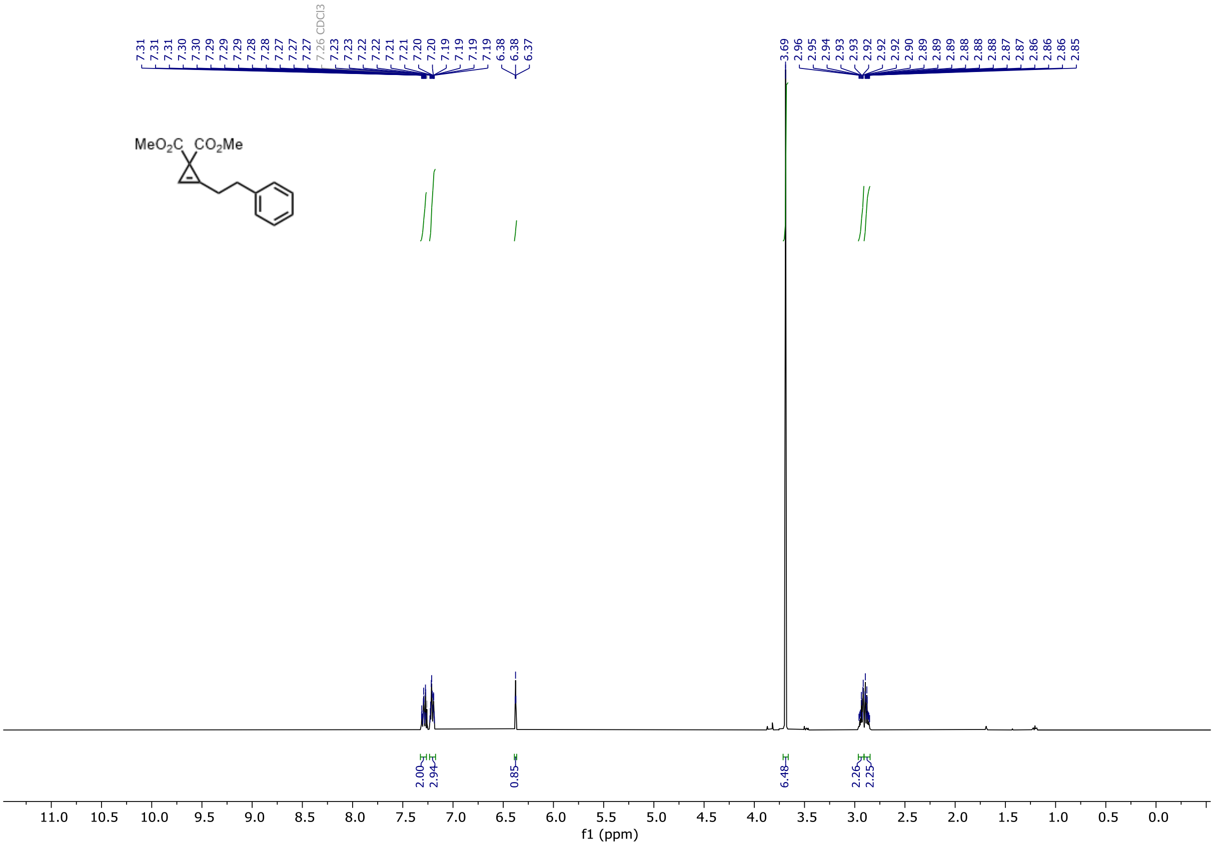

^{13}**C NMR** (101 MHz, $CDCl_3$) of **S20**

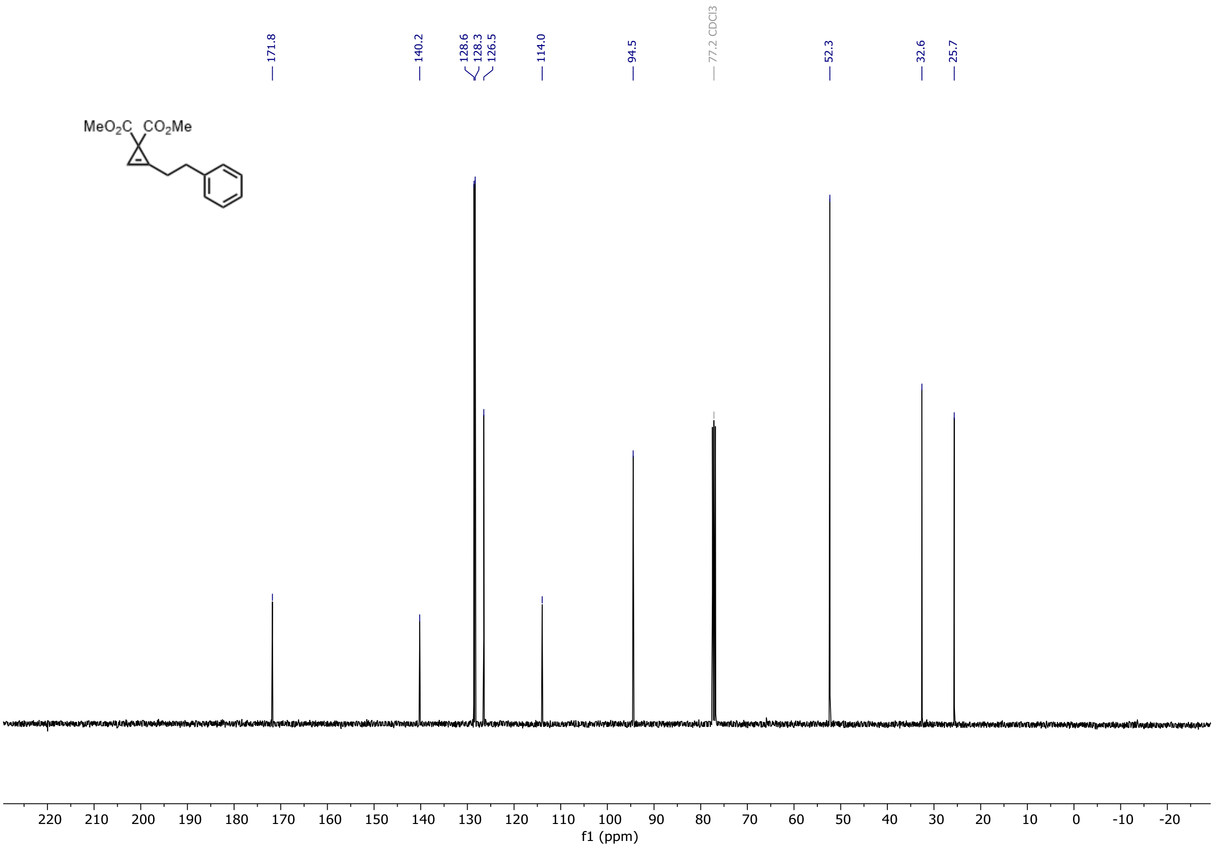

^{1}H NMR (400 MHz, $CDCl_3$) of **S21**

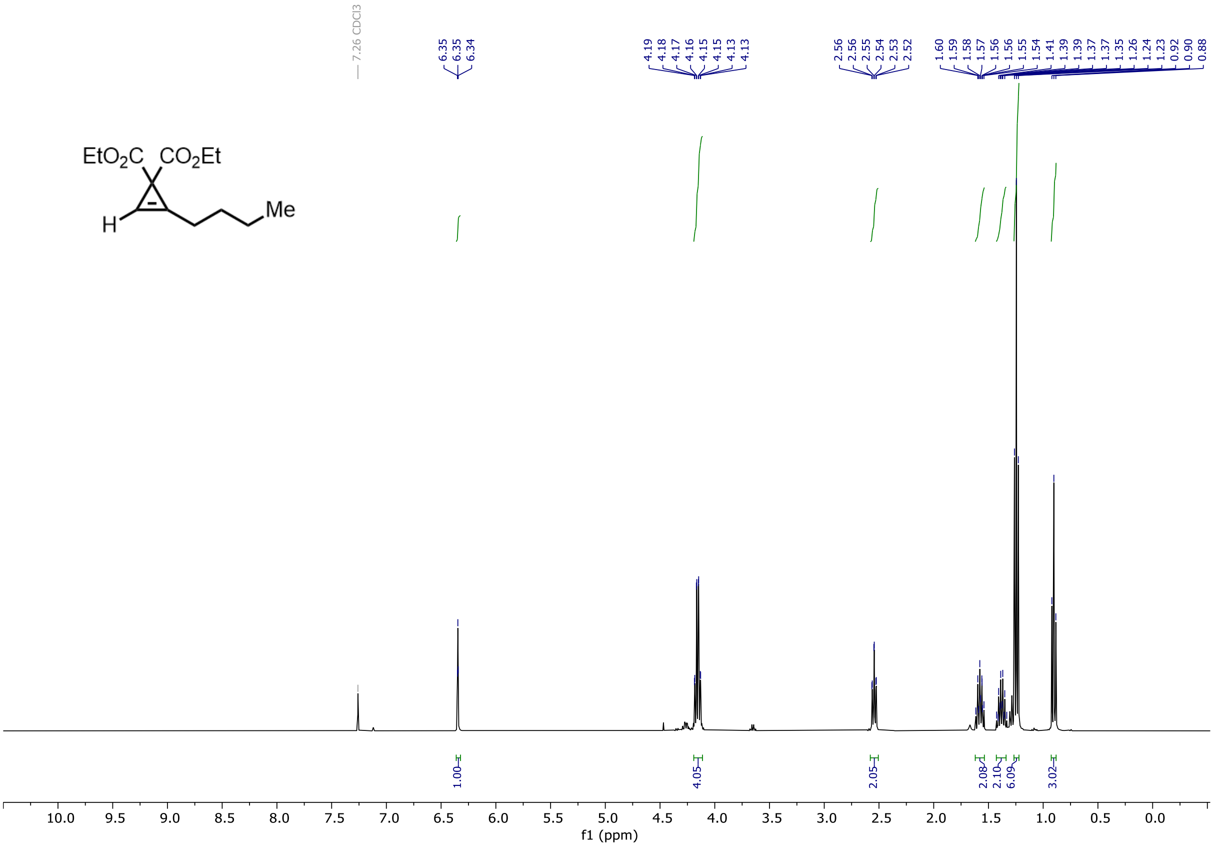

^{13}C NMR (101 MHz, $CDCl_3$) of **S21**

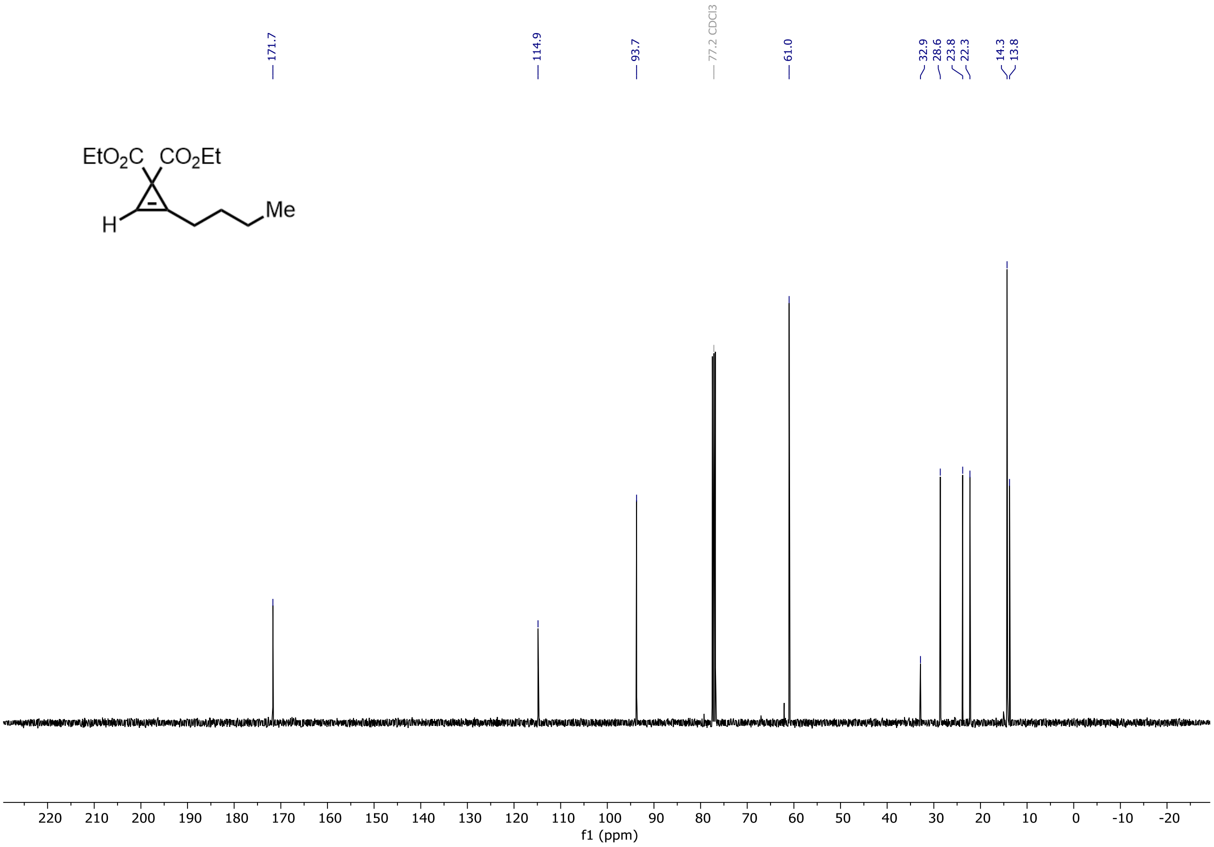

^{1}H NMR (400 MHz, $CDCl_3$) of **S22**

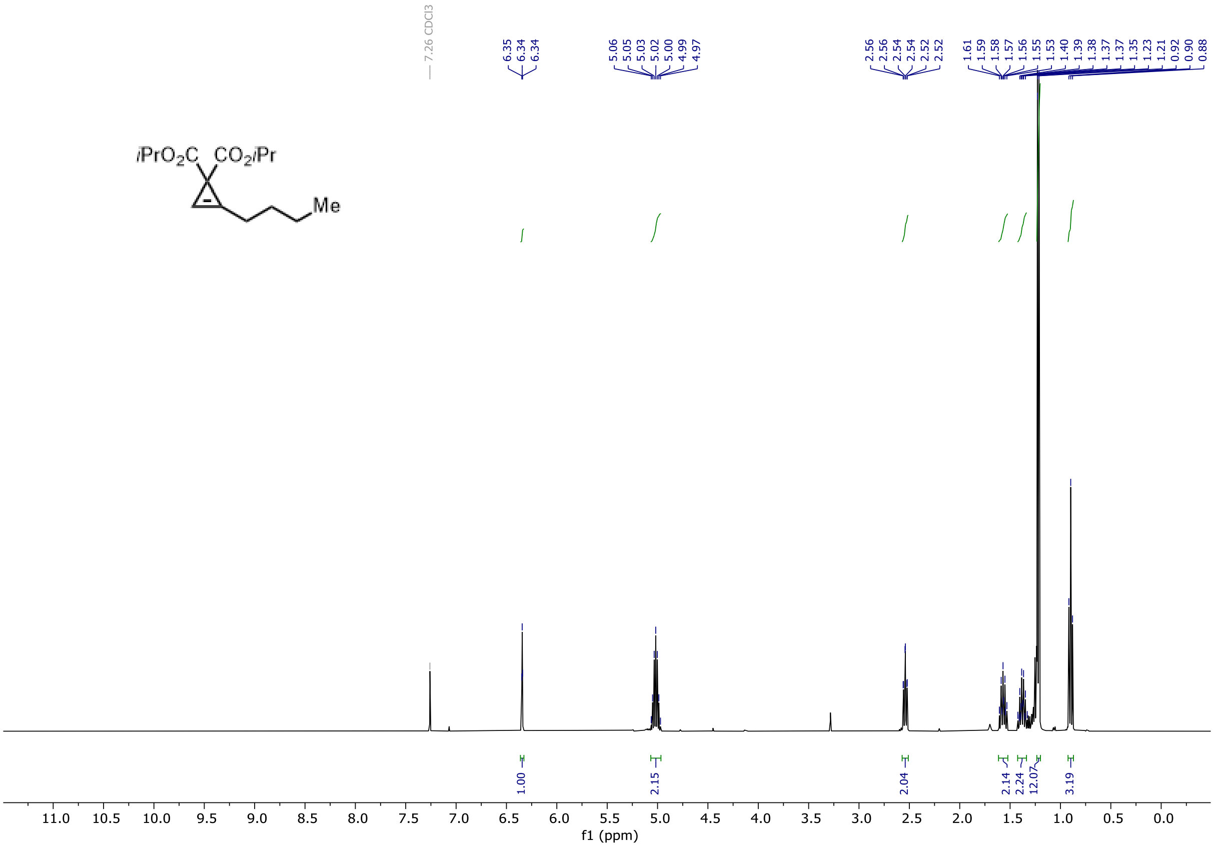

^{13}C NMR (101 MHz, $CDCl_3$) of **S22**

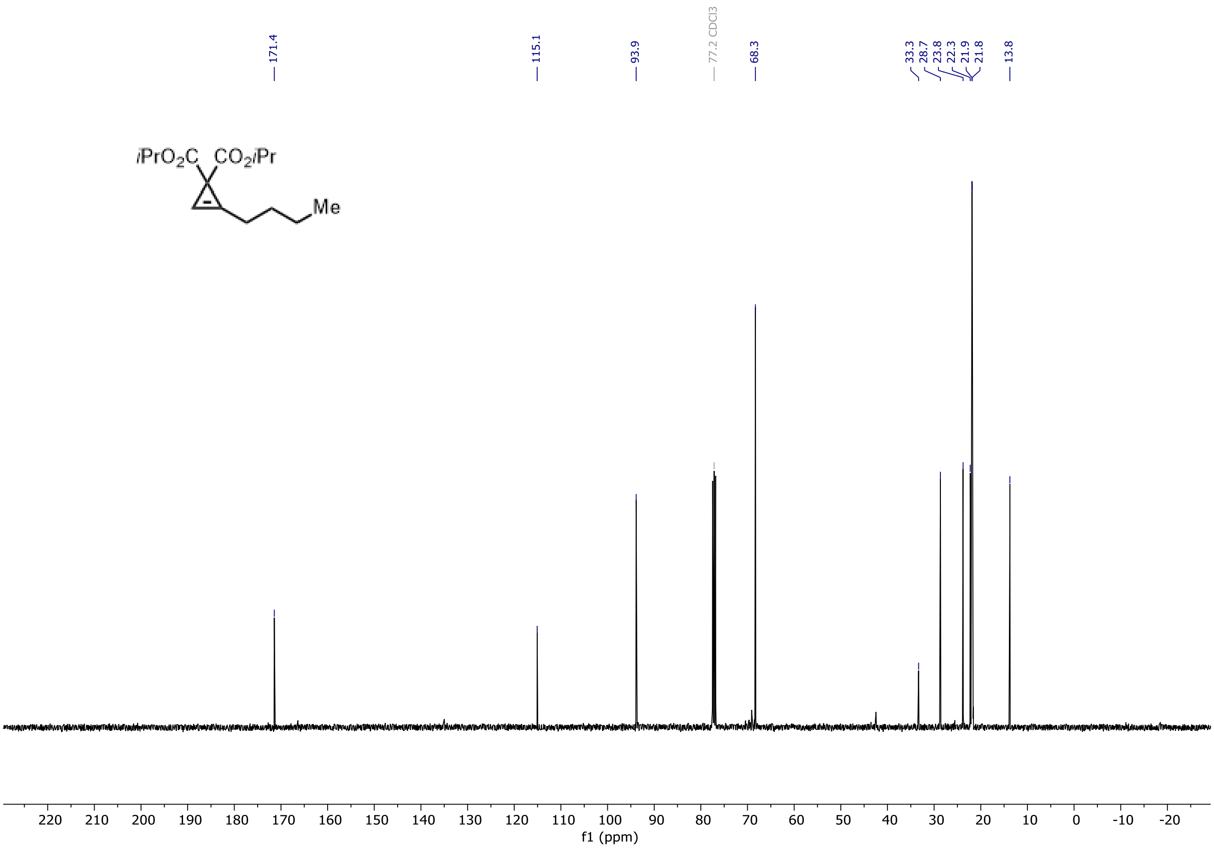

^{1}H NMR (400 MHz, $CDCl_3$) of **S23**

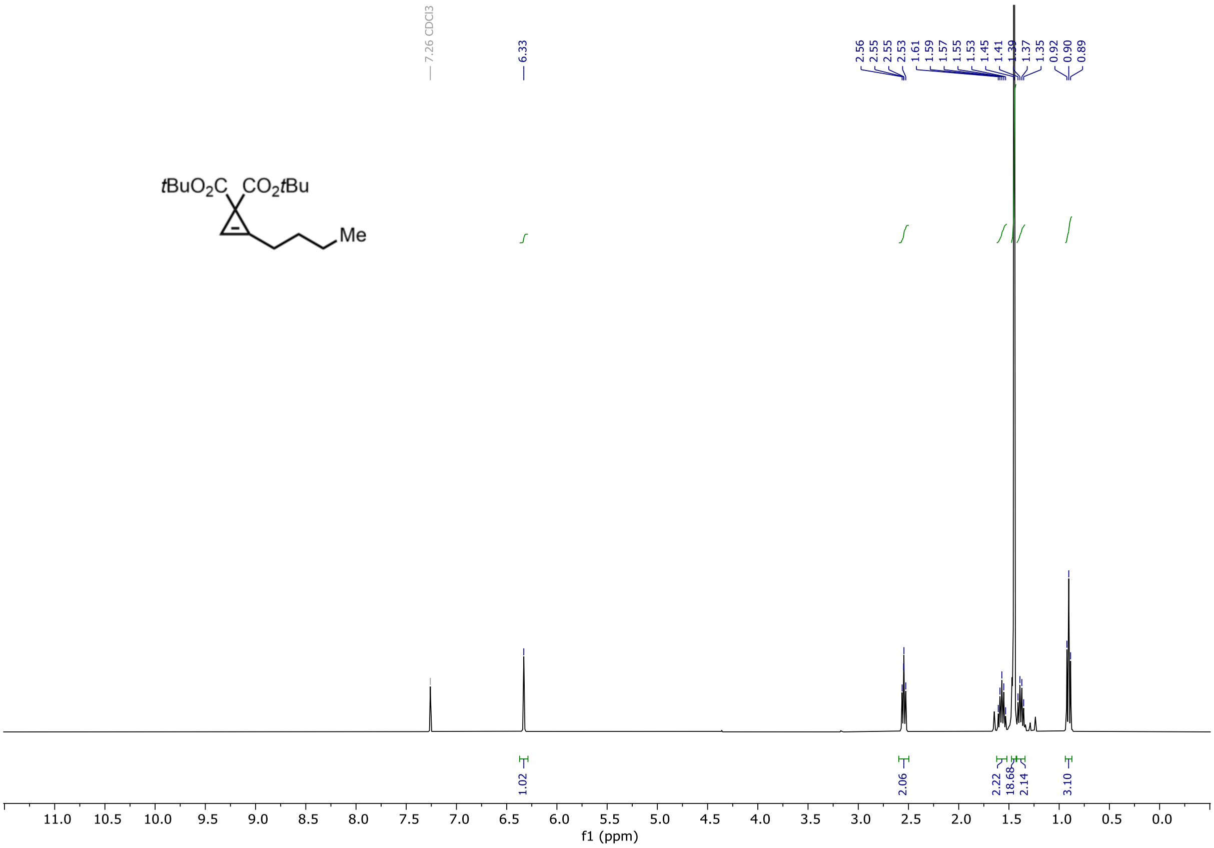

^{13}C NMR (101 MHz, $CDCl_3$) of **S23**

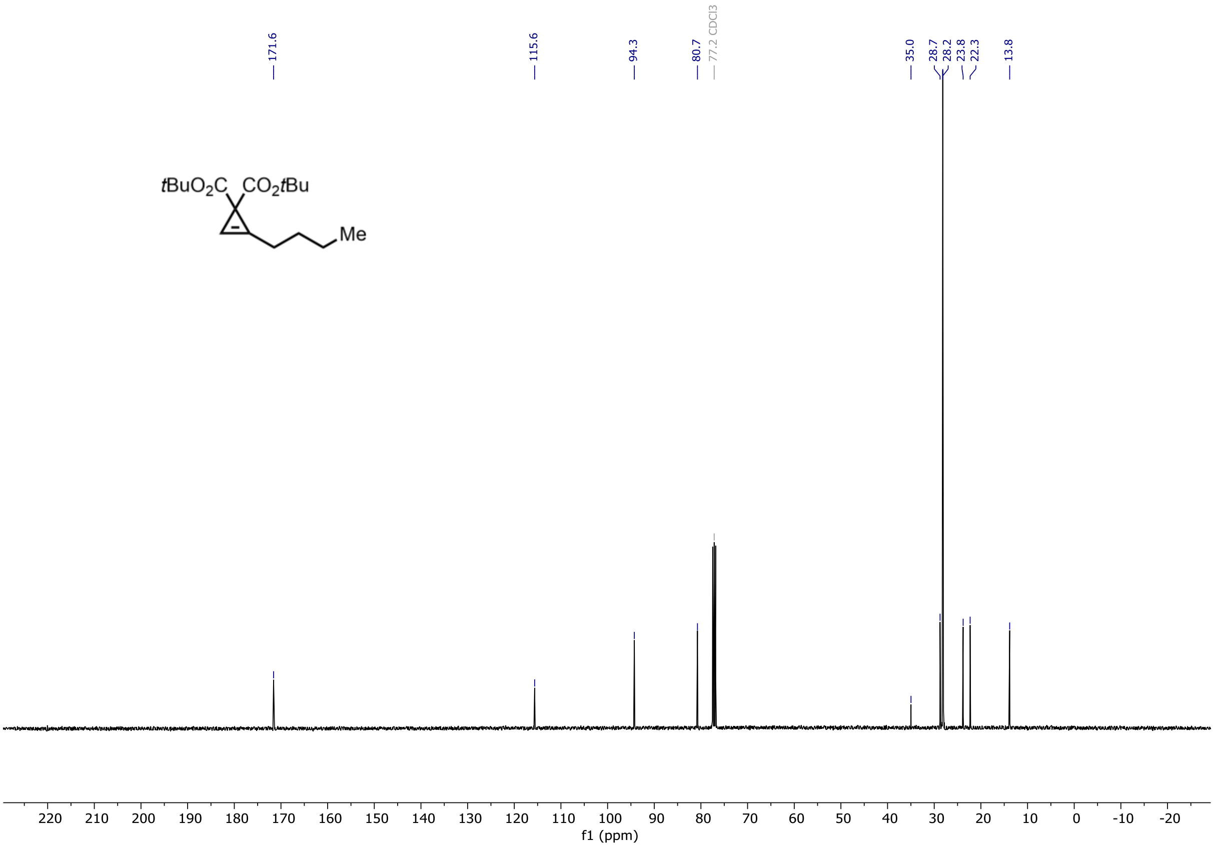

^{1}H NMR (400 MHz, $CDCl_3$) of **S24**

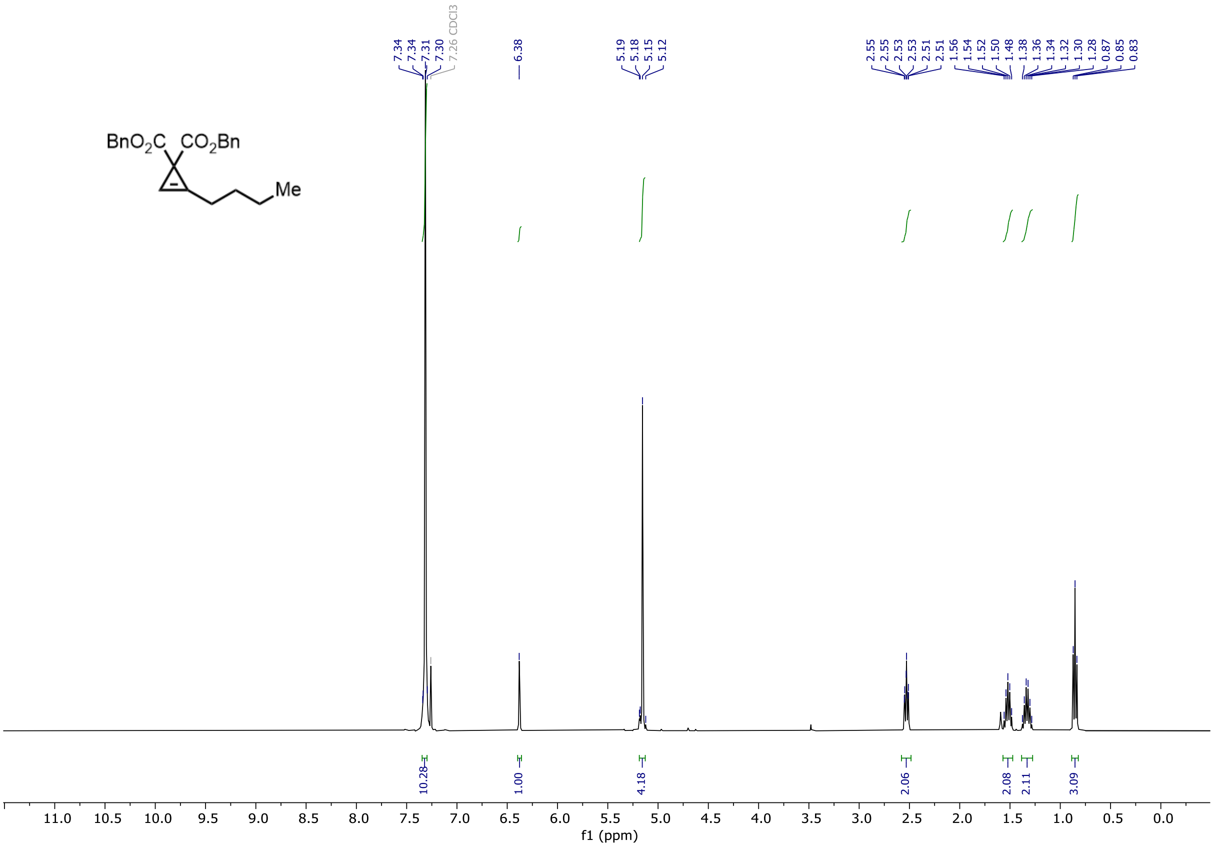

^{13}C NMR (101 MHz, $CDCl_3$) of **S24**

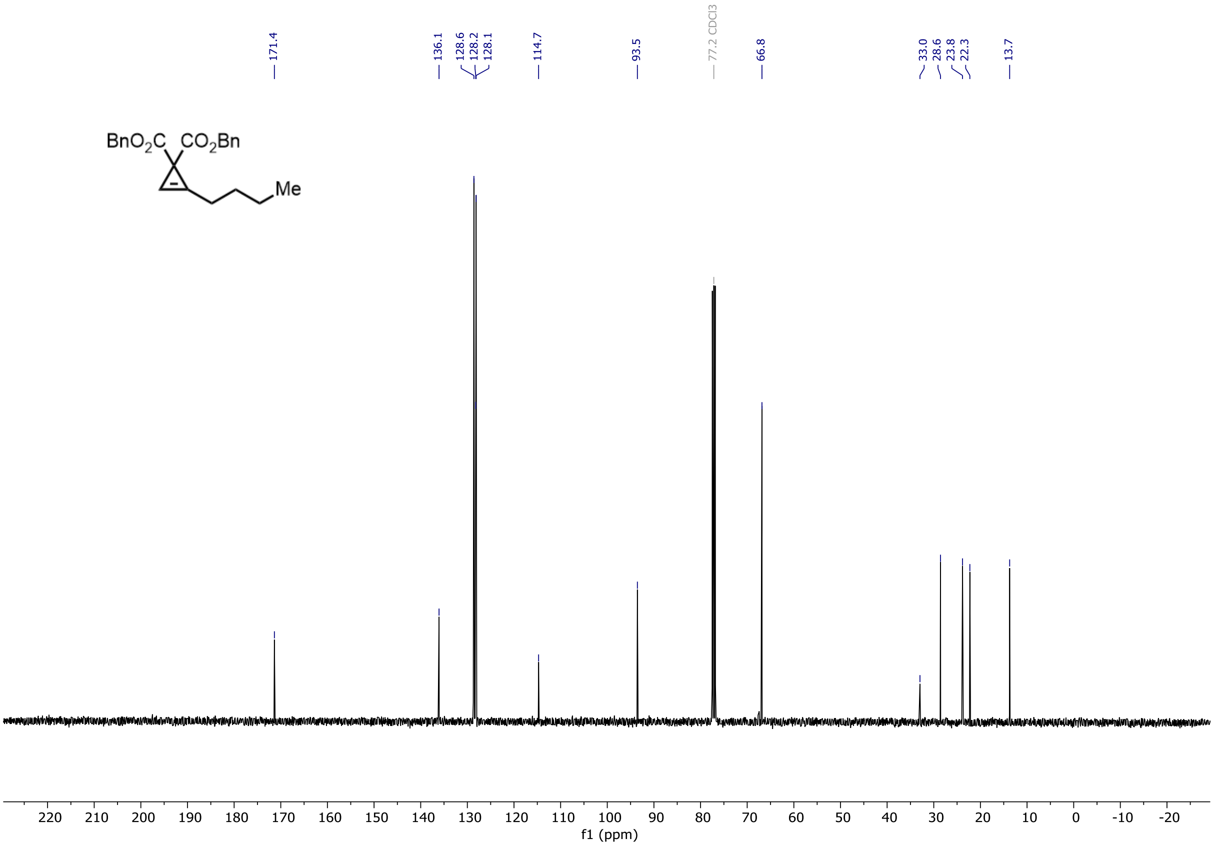

^{1}H NMR (500 MHz, $CDCl_3$) of **3a**

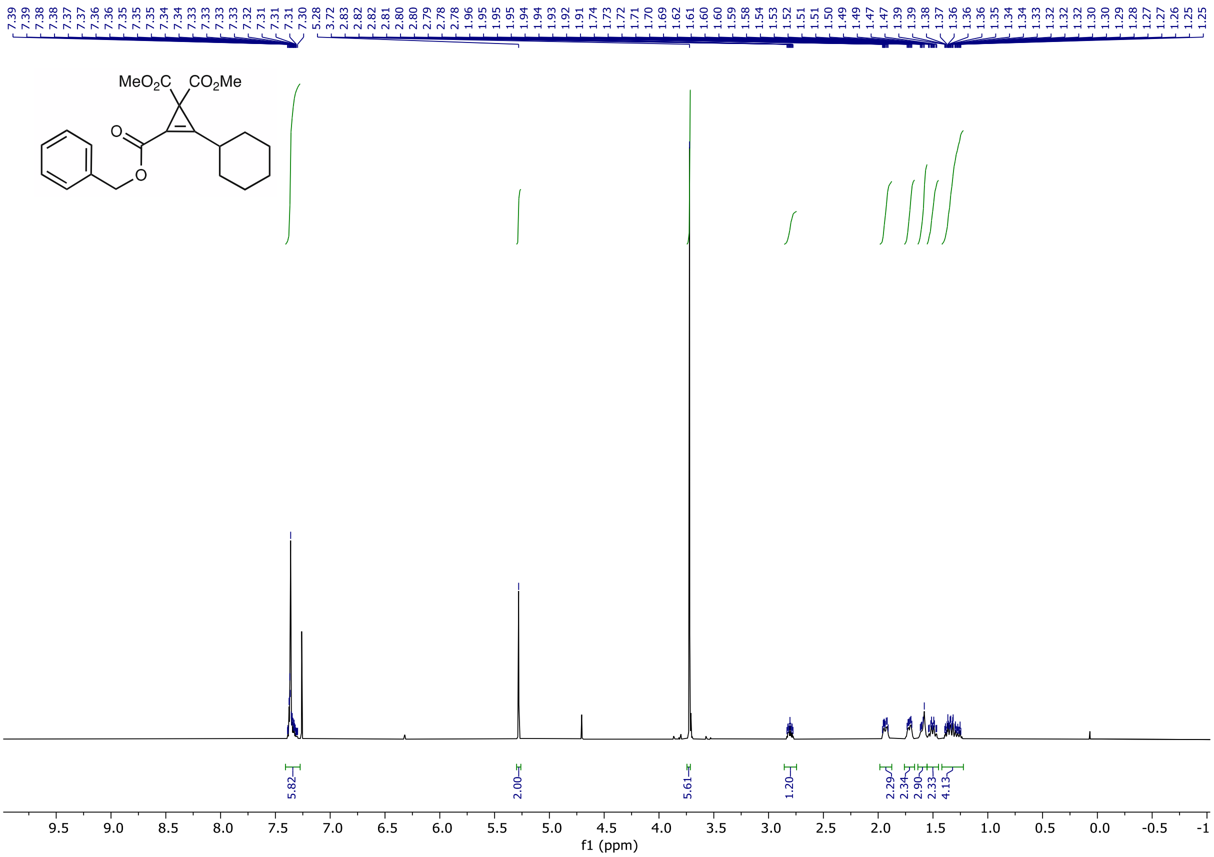

^{13}C NMR (126 MHz, $CDCl_3$) of **3a**

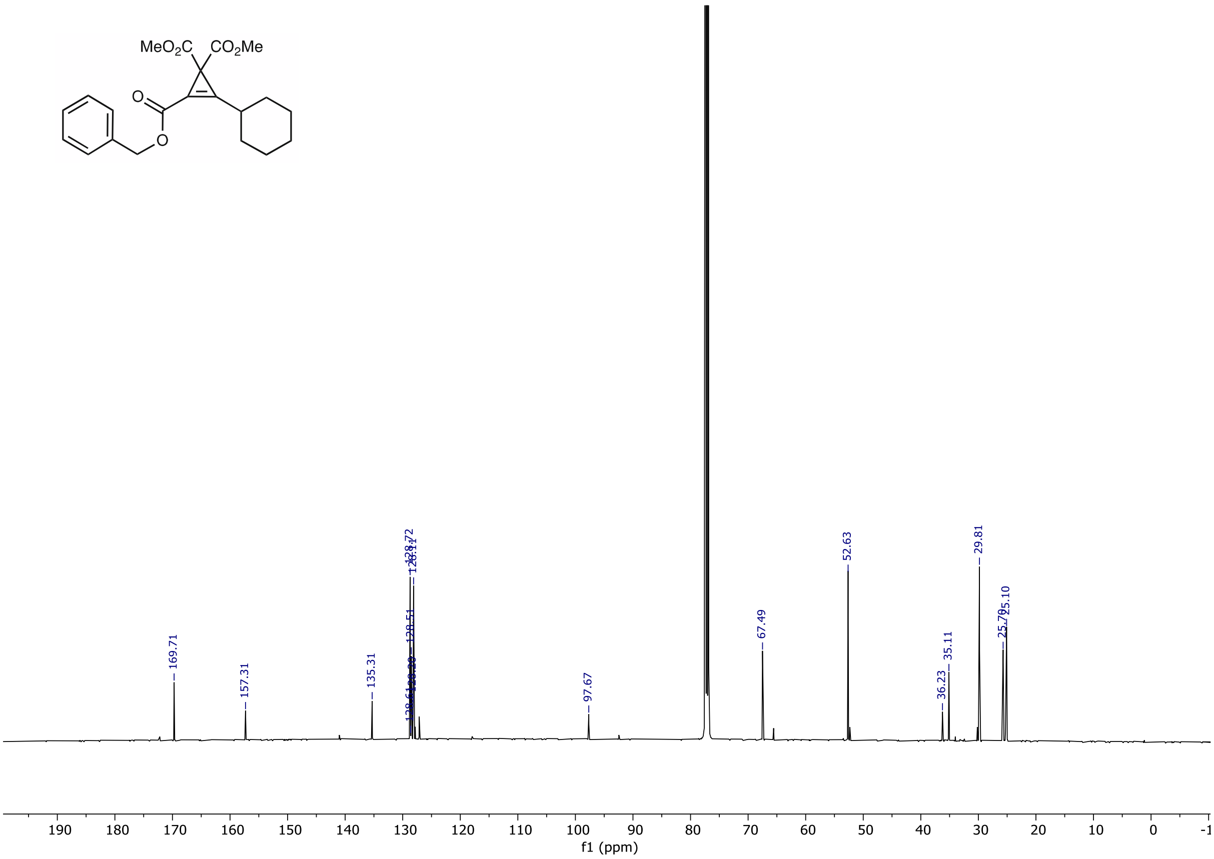

^{1}H NMR (500 MHz, $CDCl_3$) of **5a**

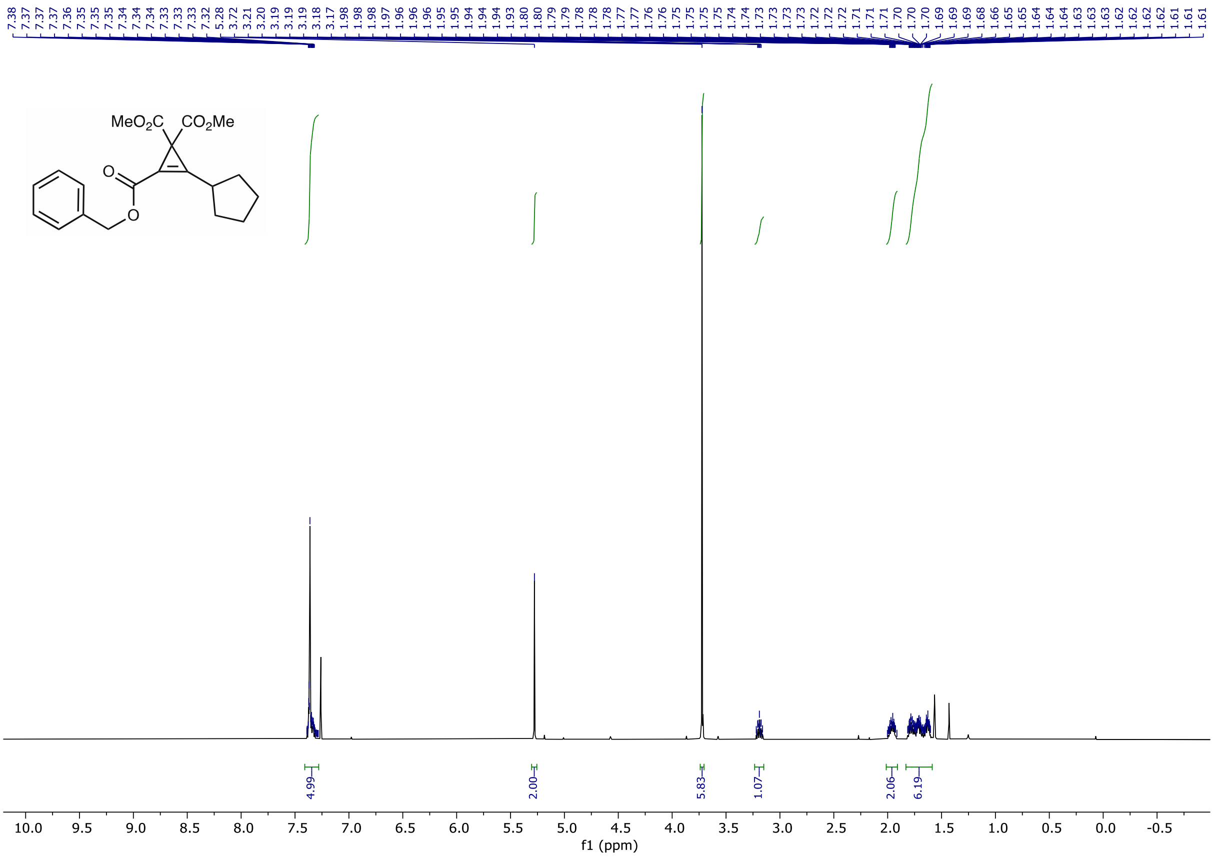

^{13}C NMR (126 MHz, $CDCl_3$) of **5a**

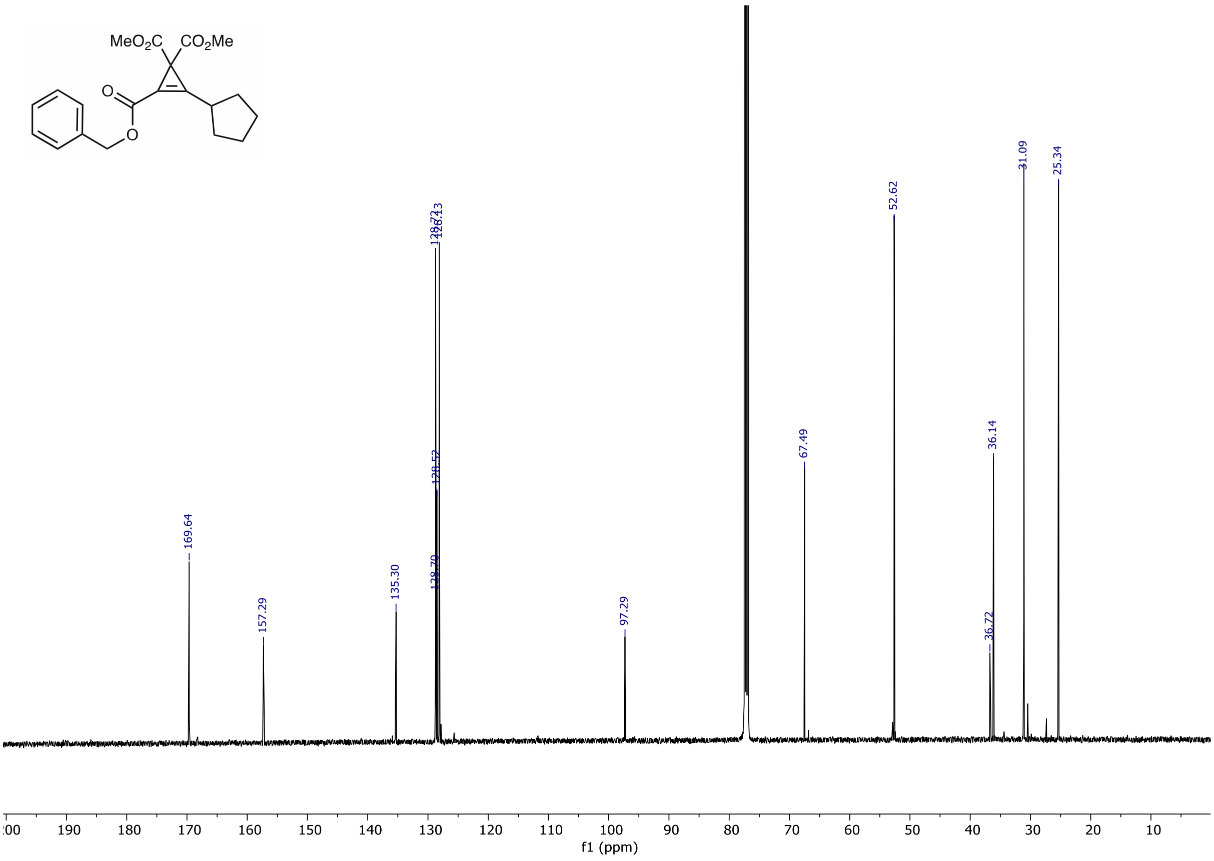

^{1}H NMR (400 MHz, $CDCl_3$) of **6a**

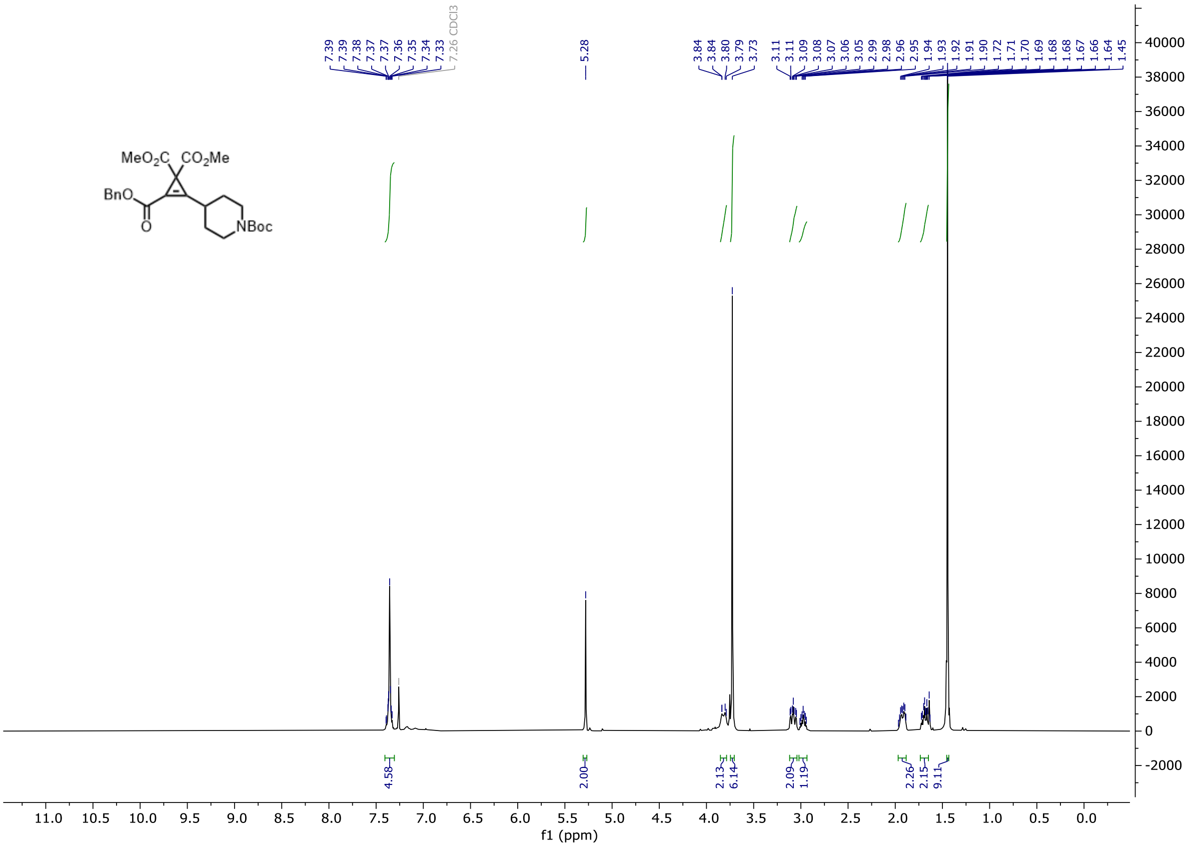

^{13}C NMR (101 MHz, $CDCl_3$) of **6a**

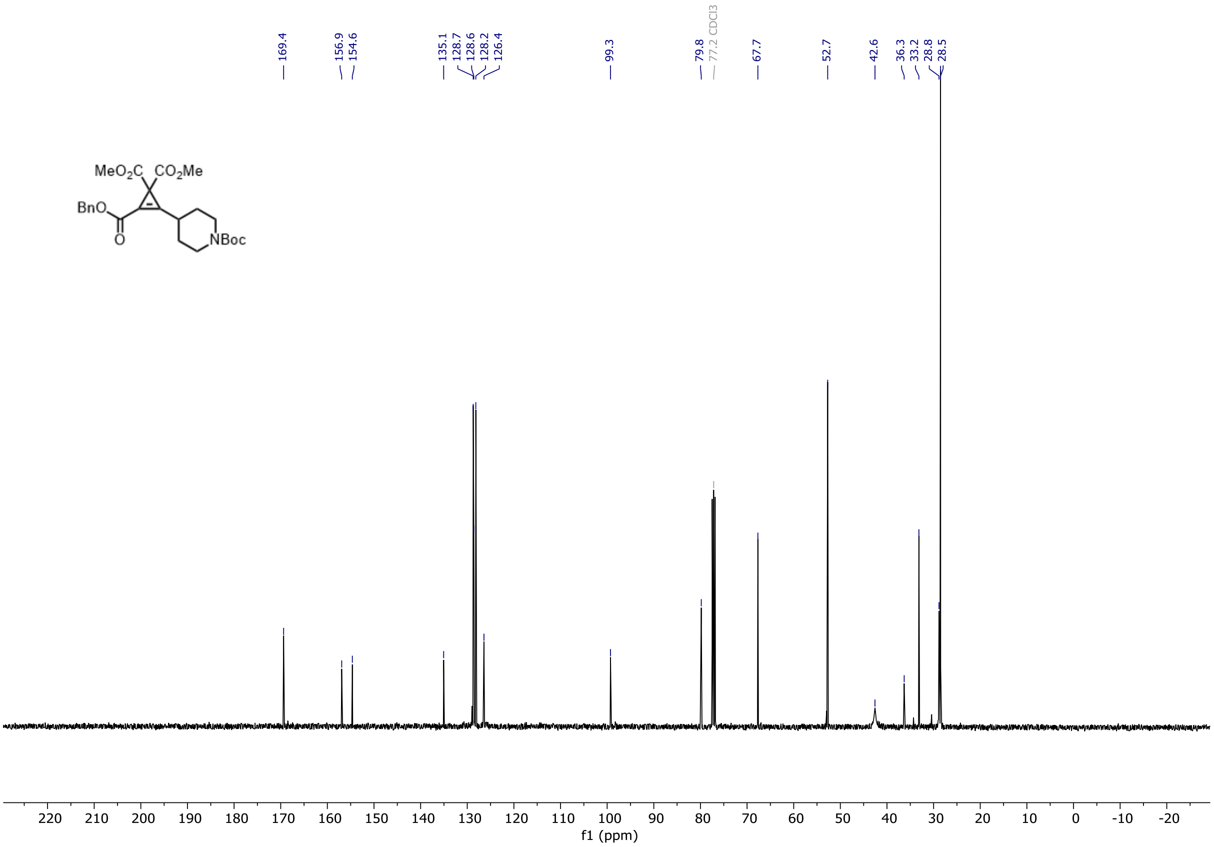

^{1}H NMR (400 MHz, $CDCl_3$) of **7a**

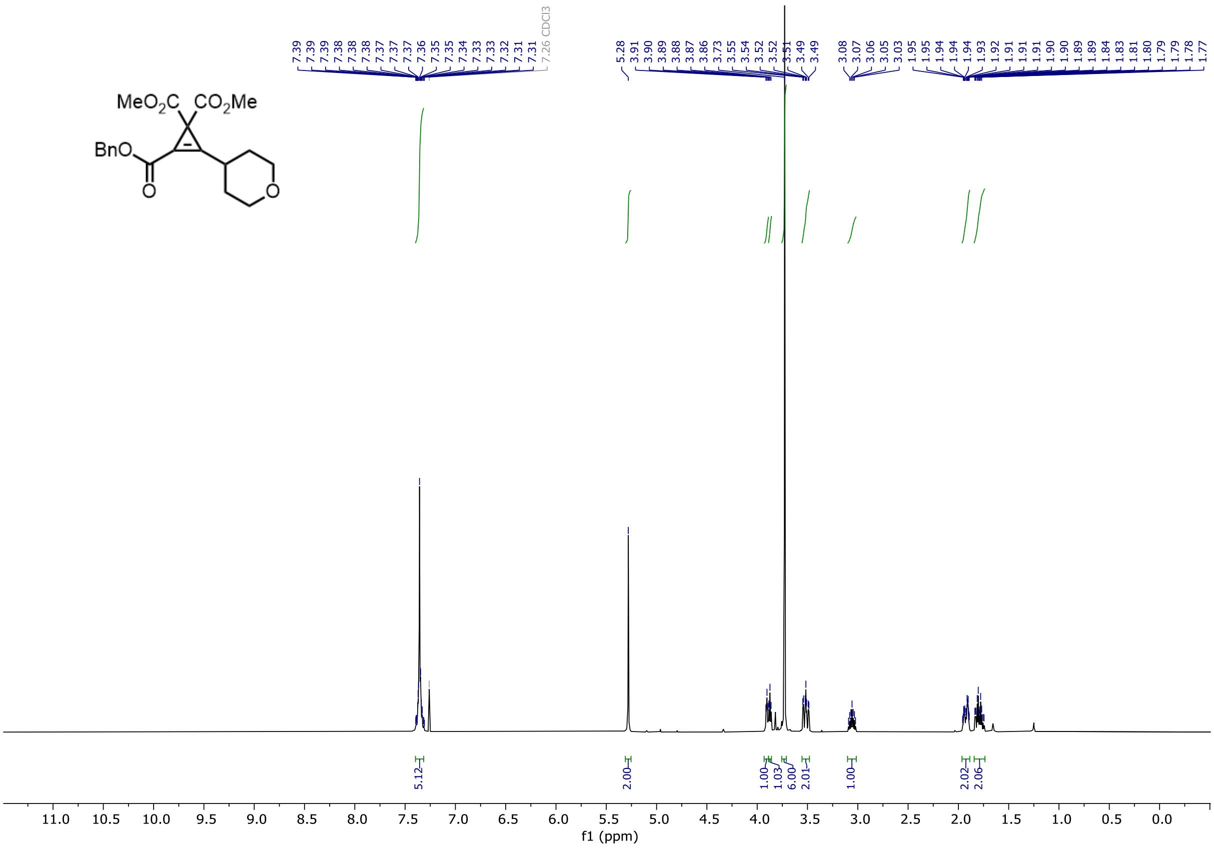

^{13}C NMR (101 MHz, $CDCl_3$) of **7a**

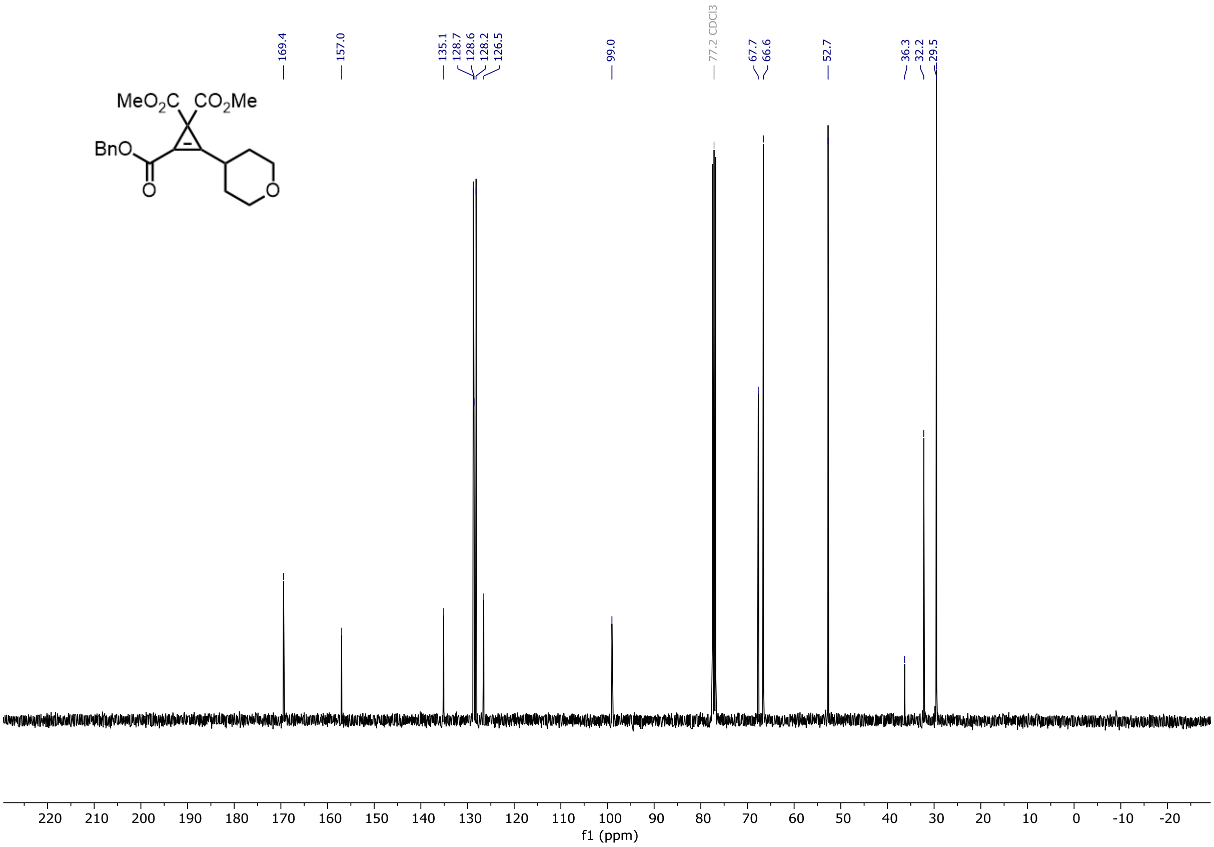

^{1}H NMR (500 MHz, $CDCl_3$) of **8a**

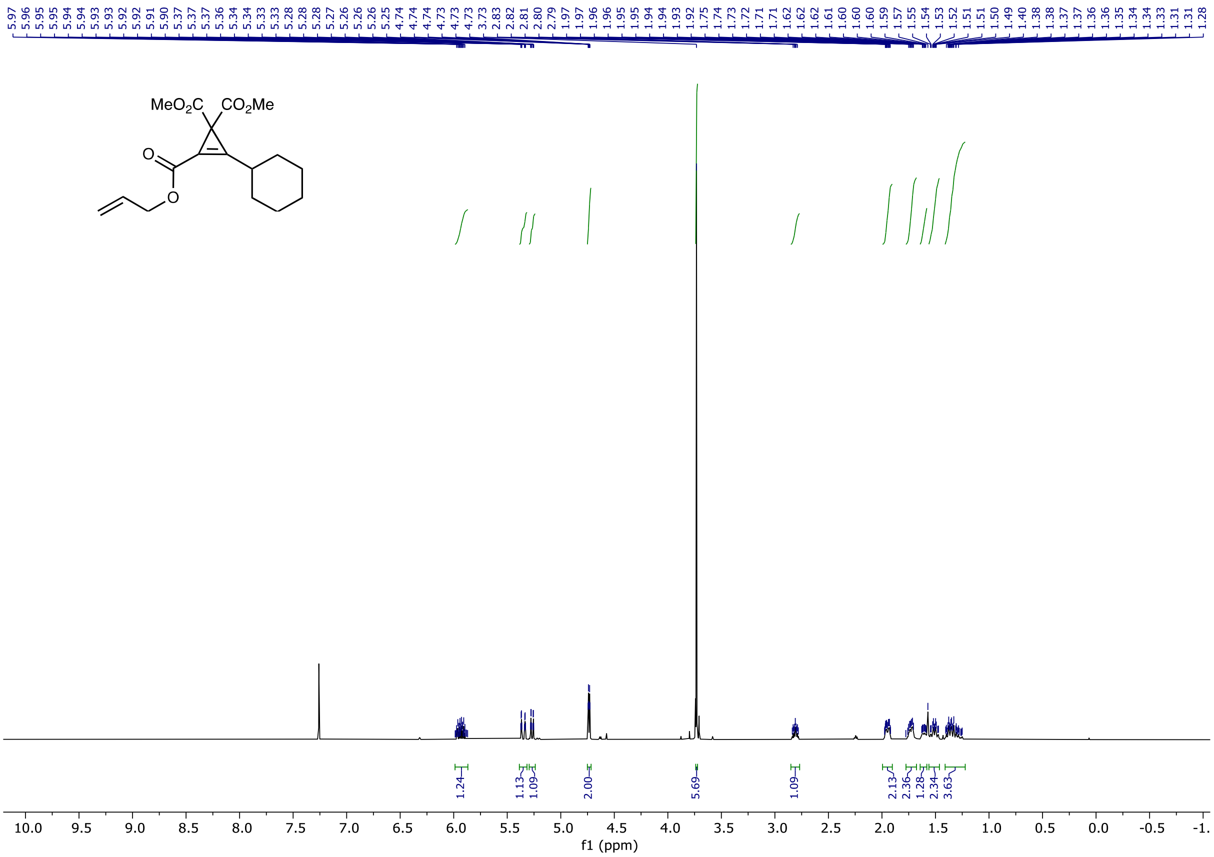

^{13}C NMR (126 MHz, $CDCl_3$) of 8**a**

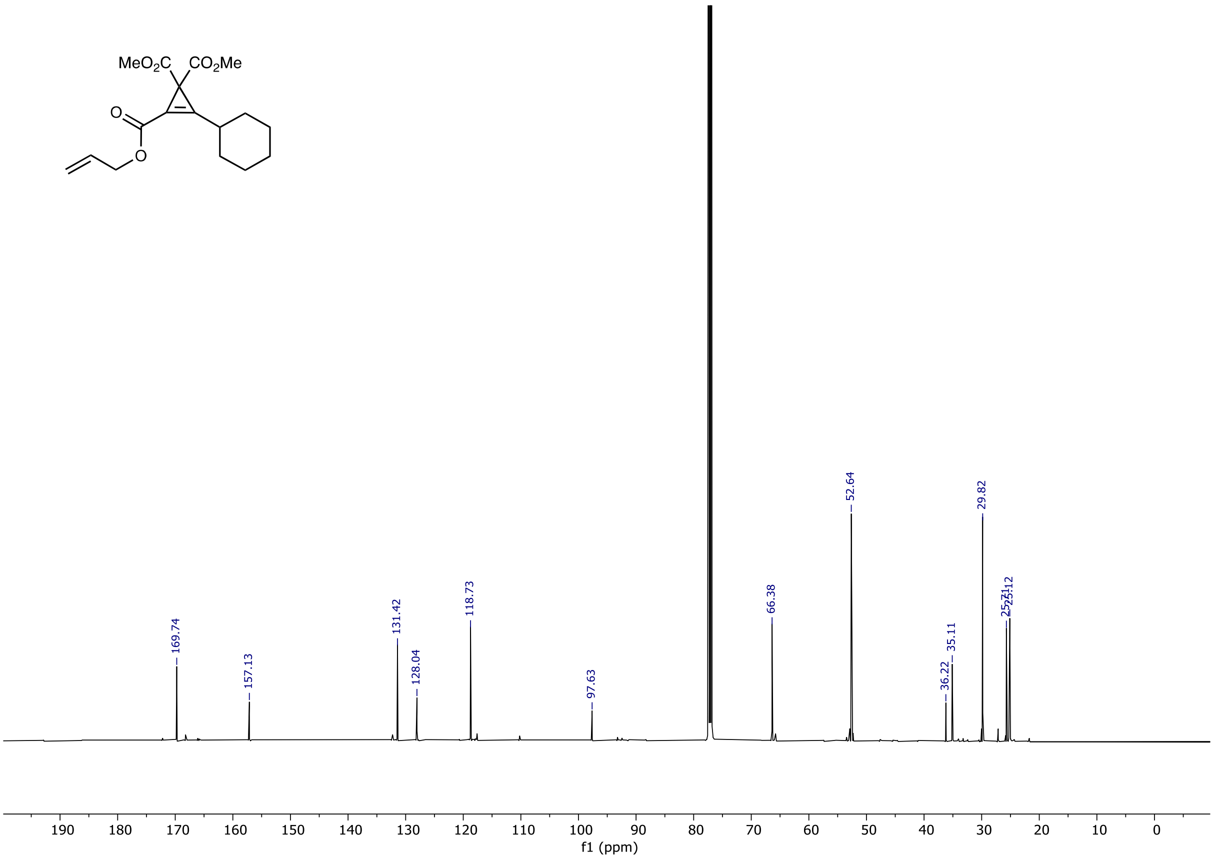

^{1}H NMR (500 MHz, $CDCl_3$) of **9a**

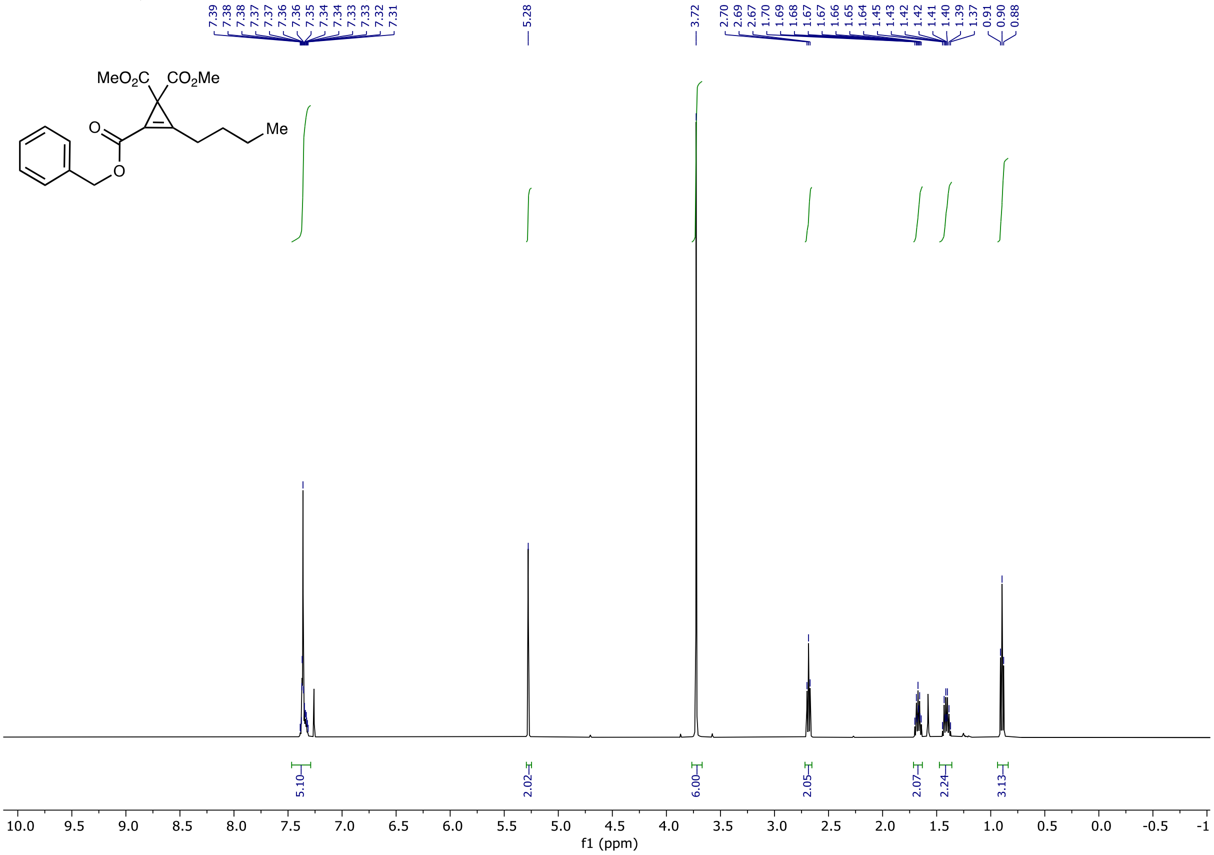

^{13}C NMR (126 MHz, $CDCl_3$) of **9a**

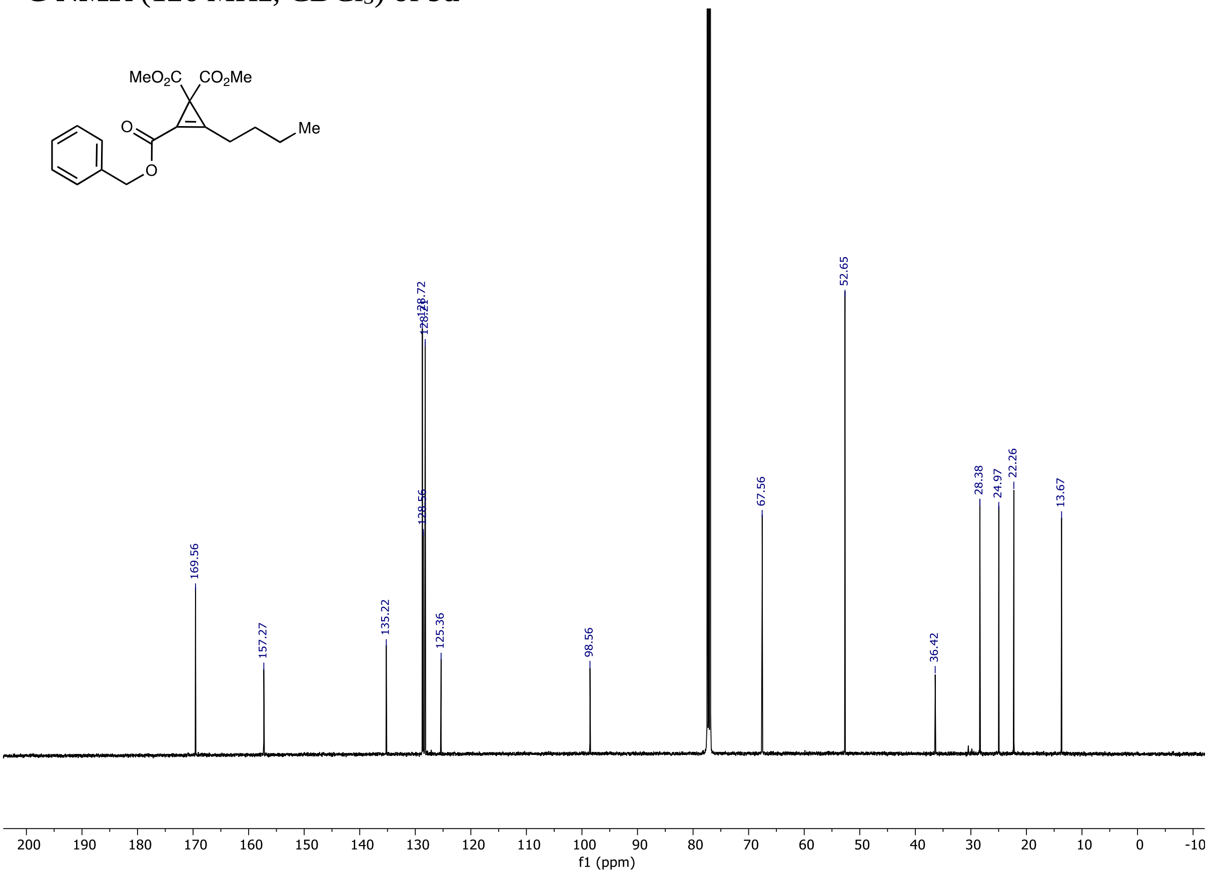

^{1}H NMR (500 MHz, $CDCl_3$) of **10a**

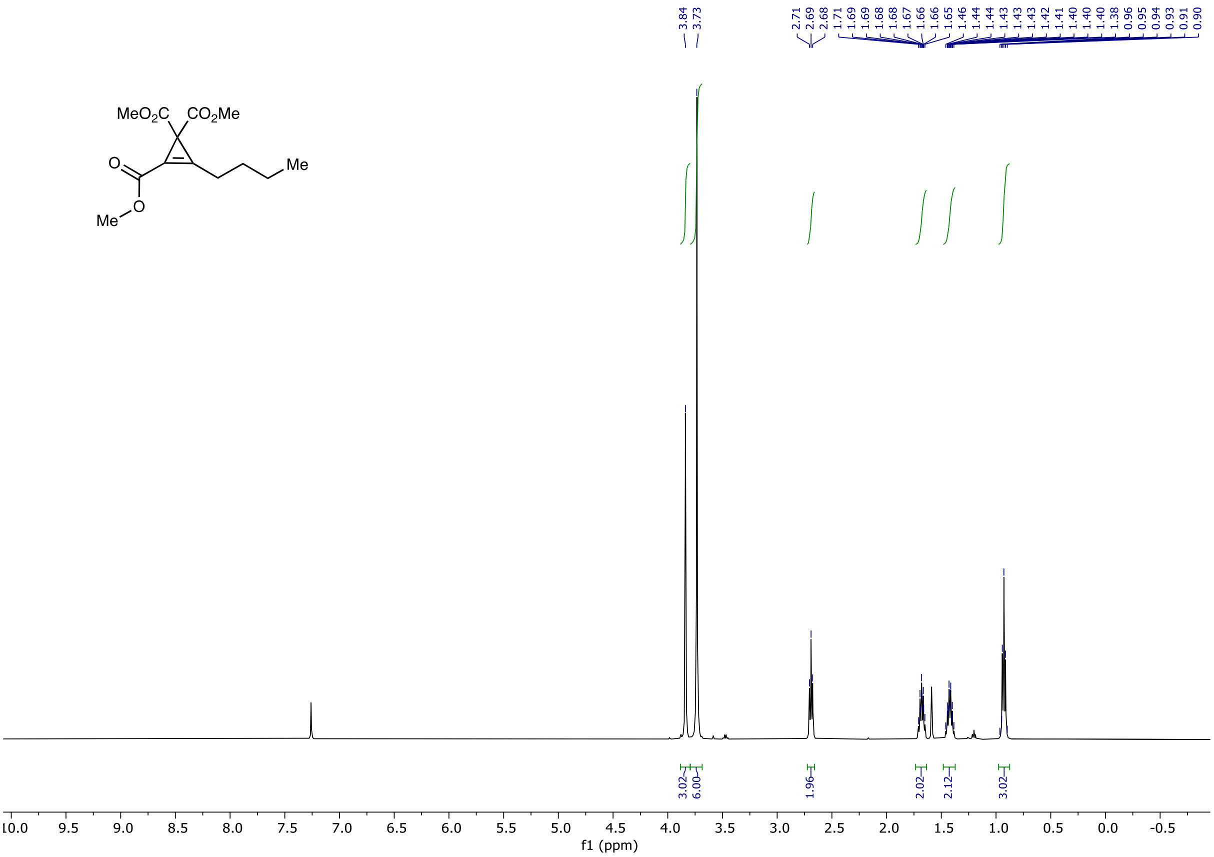

^{13}C NMR (126 MHz, $CDCl_3$) of **10a**

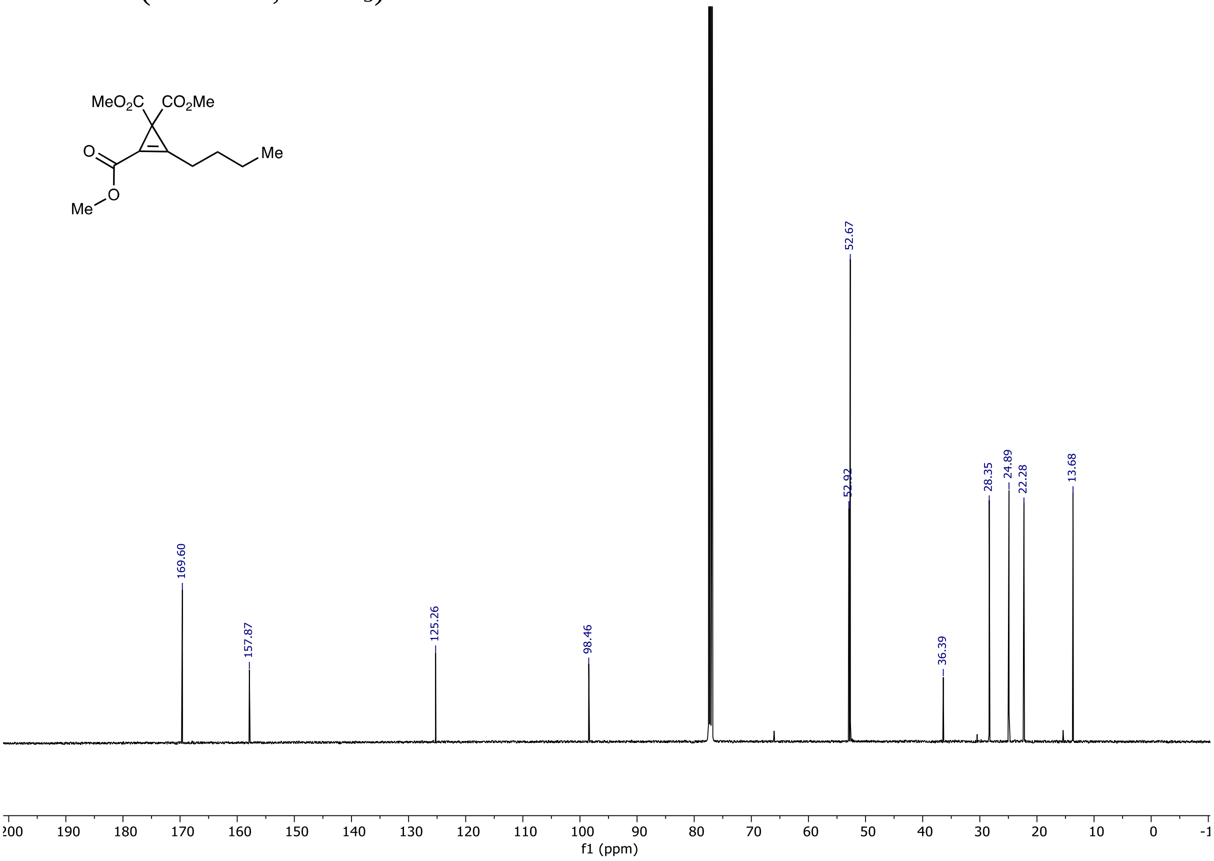

^{1}H NMR (500 MHz, $CDCl_3$) of **11a**

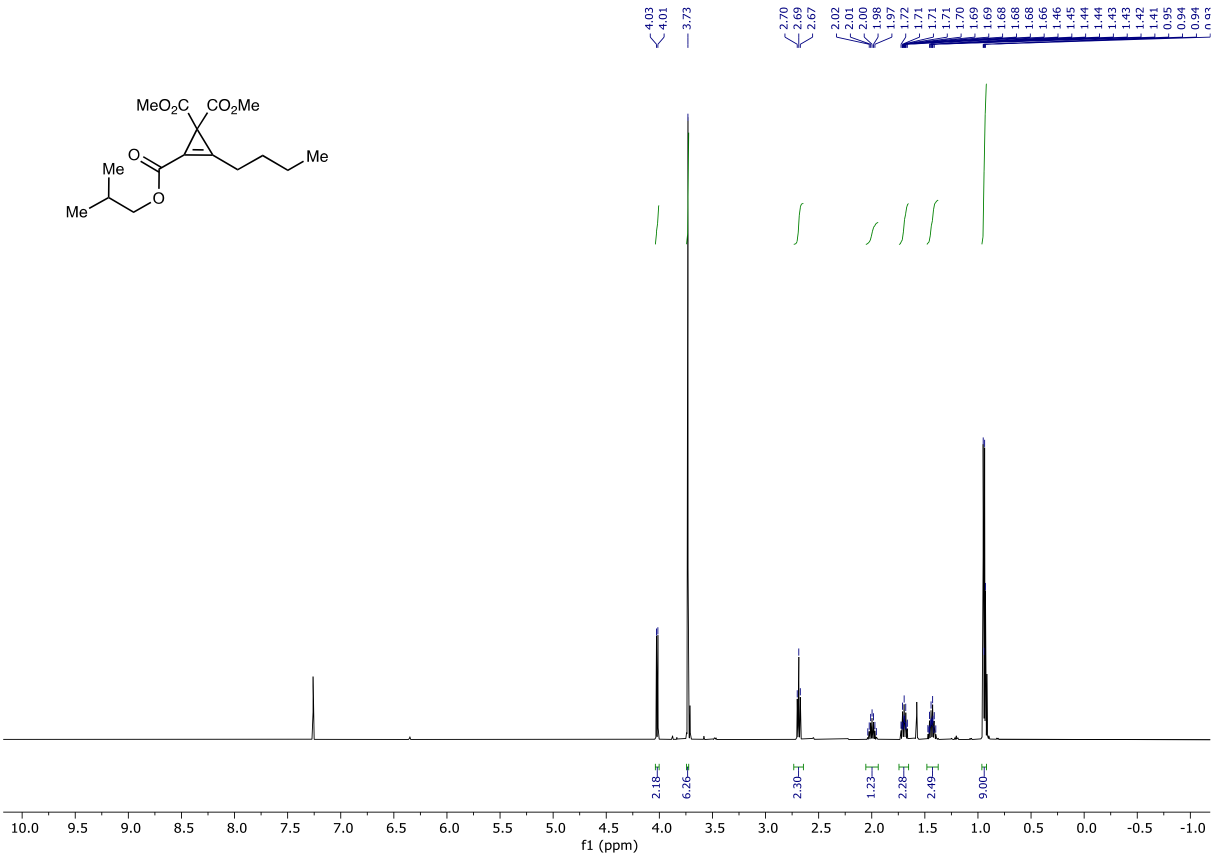

^{13}C NMR (126 MHz, $CDCl_3$) of **11a**

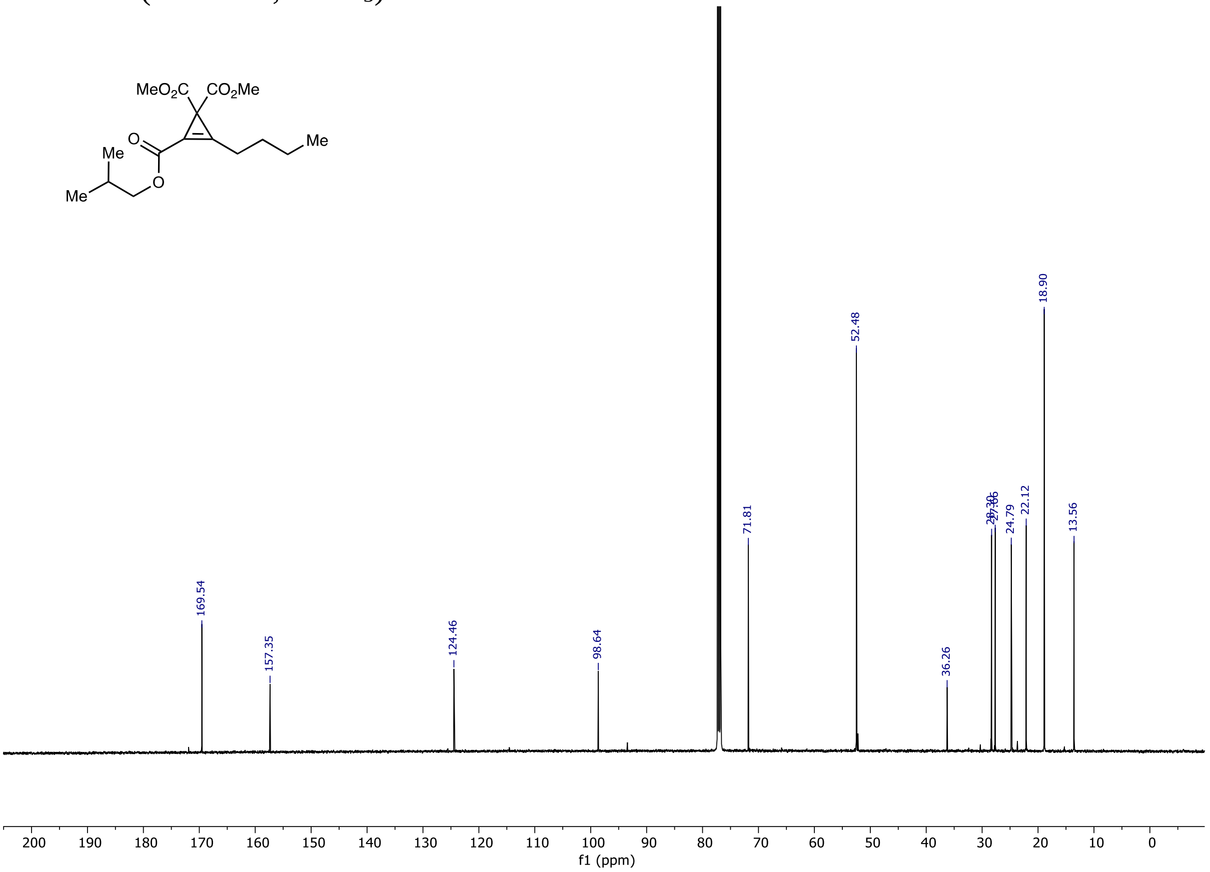

^{1}**H NMR** (400 MHz, $CDCl_3$) of **12a**

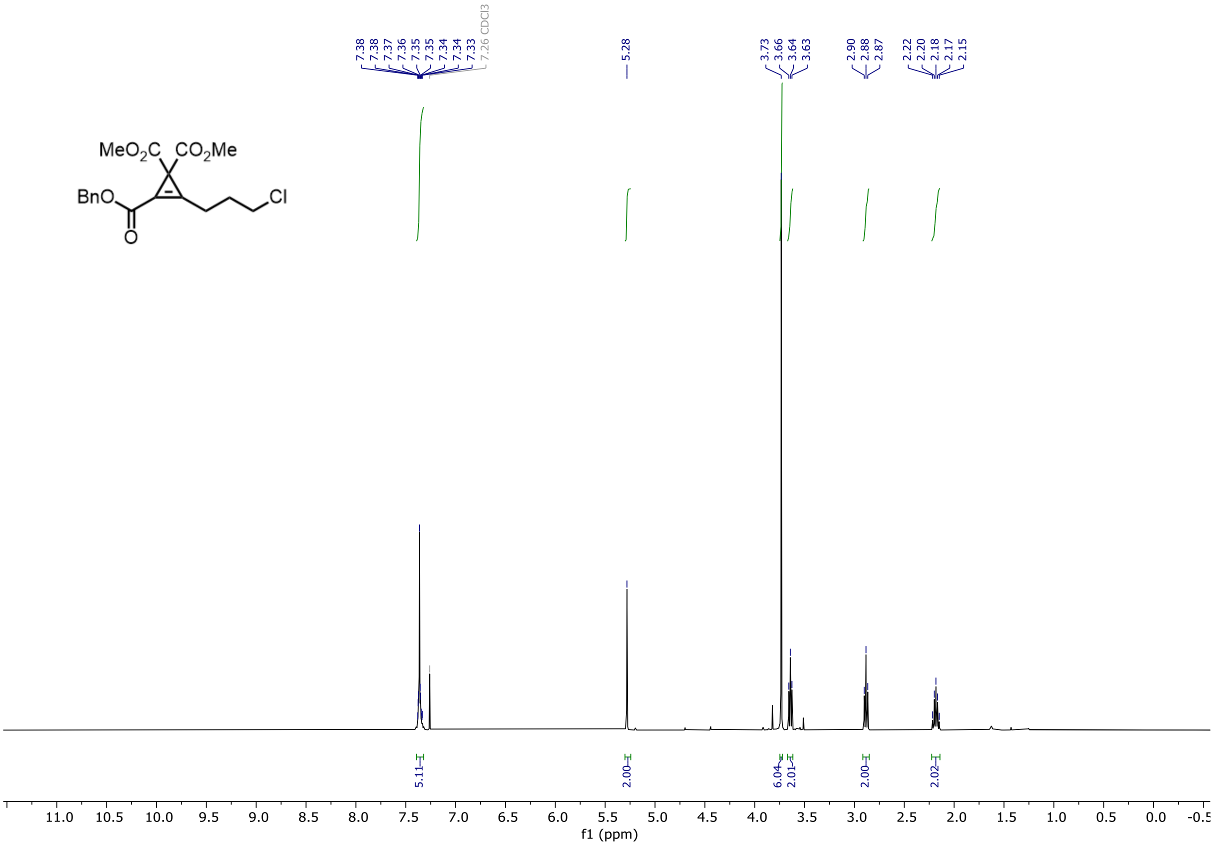

^{13}**C NMR** (101 MHz, $CDCl_3$) of **12a**

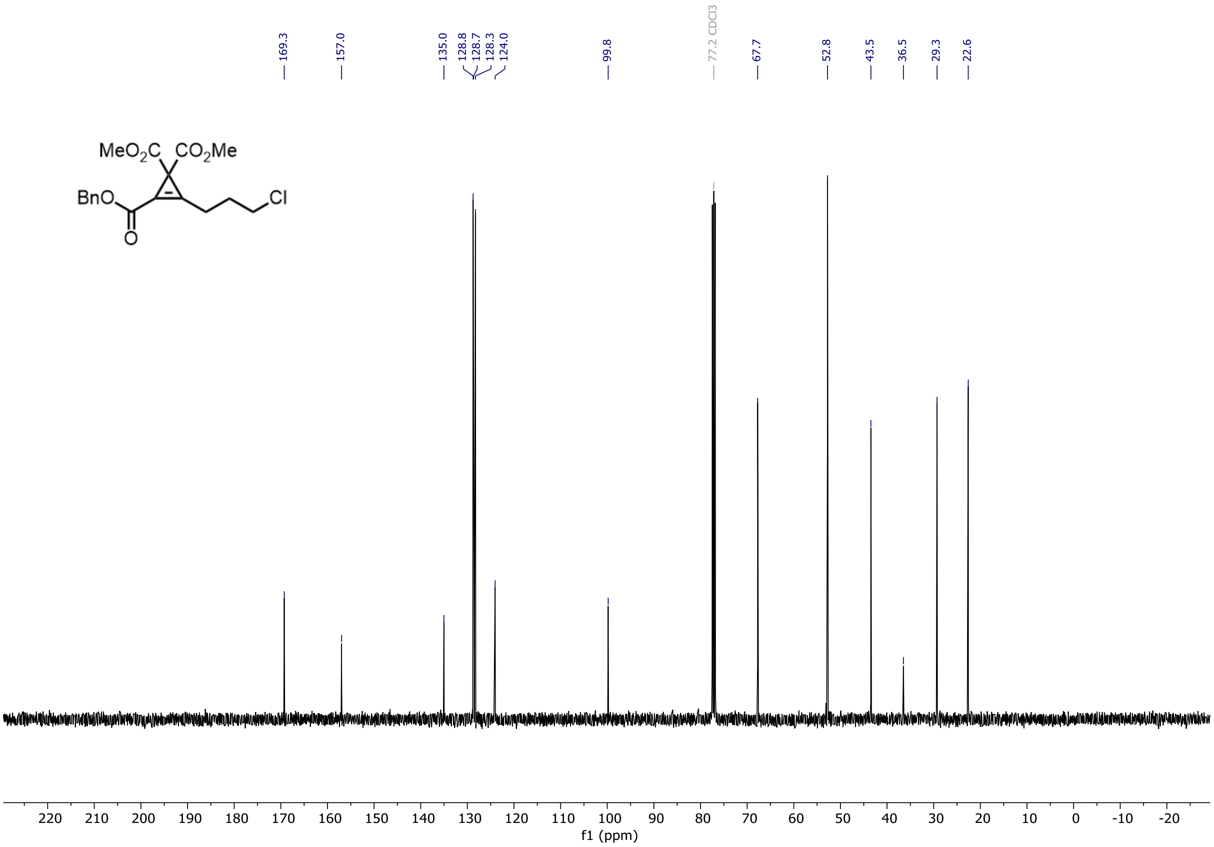

^{1}H NMR (400 MHz, $CDCl_3$) of **13a**

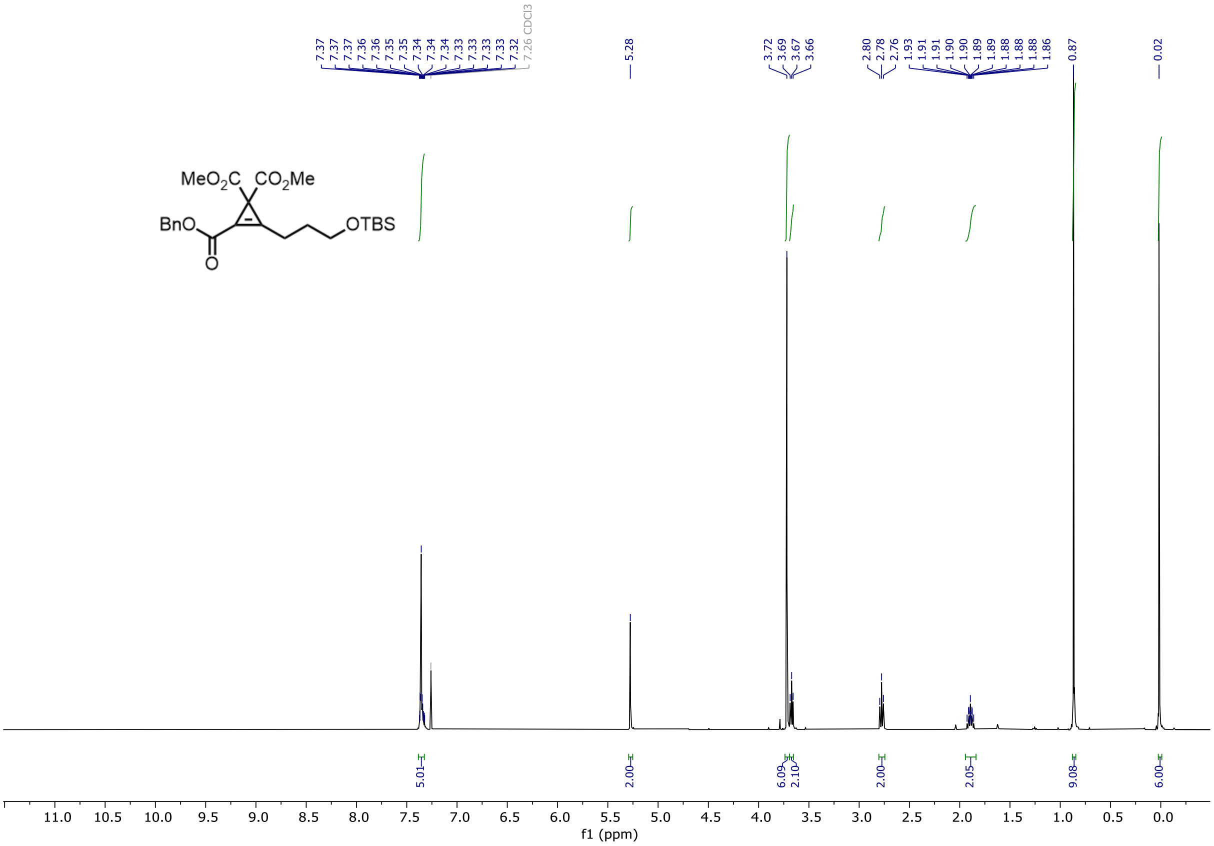

^{13}C NMR (101 MHz, $CDCl_3$) of **13a**

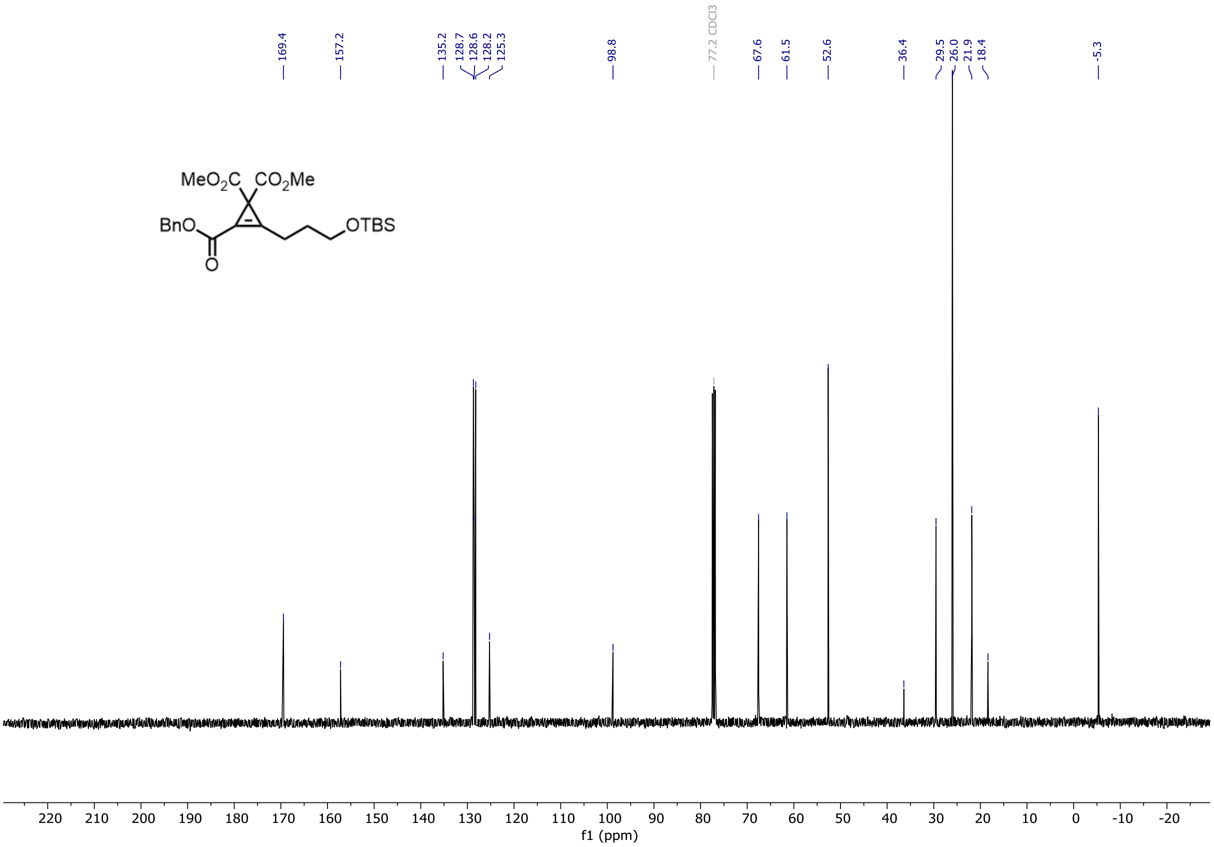

^{1}H NMR (500 MHz, $CDCl_3$) of **14a**

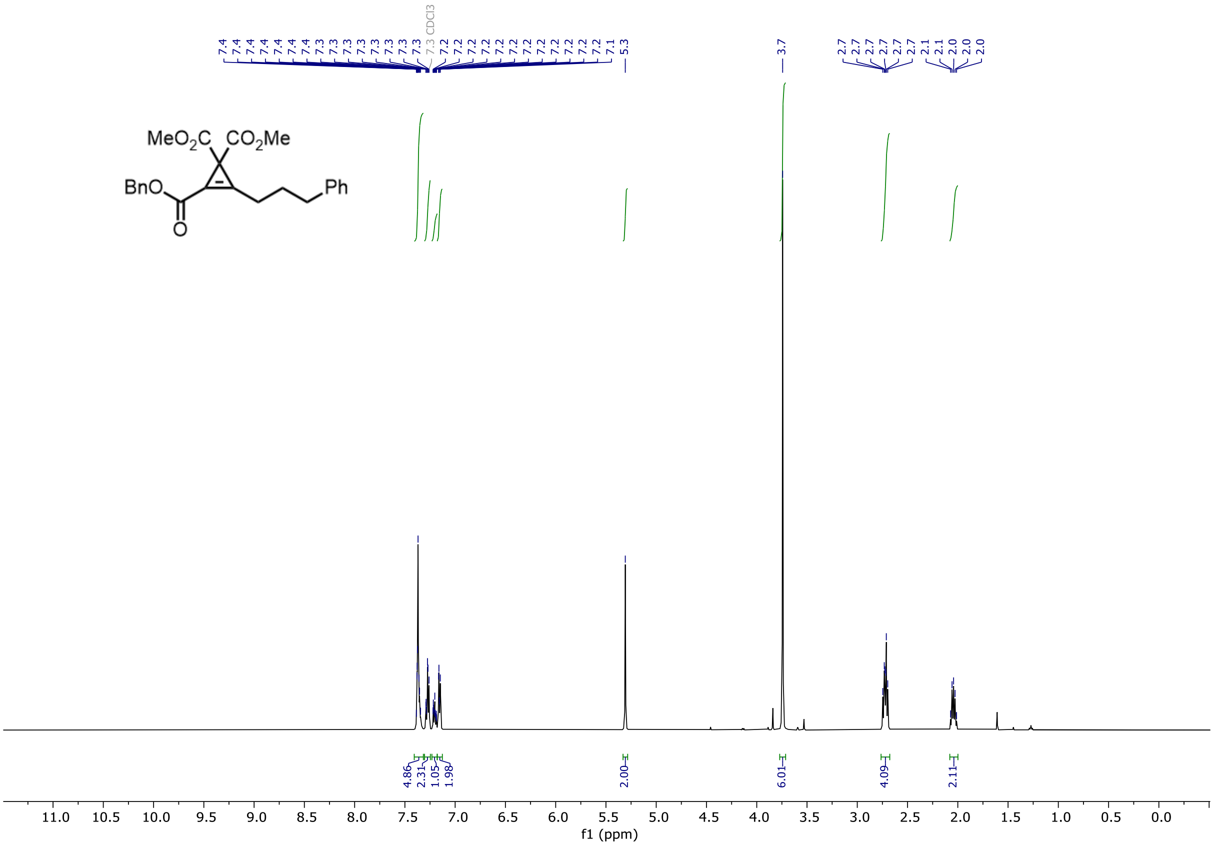

^{13}C NMR (126 MHz, $CDCl_3$) of **14a**

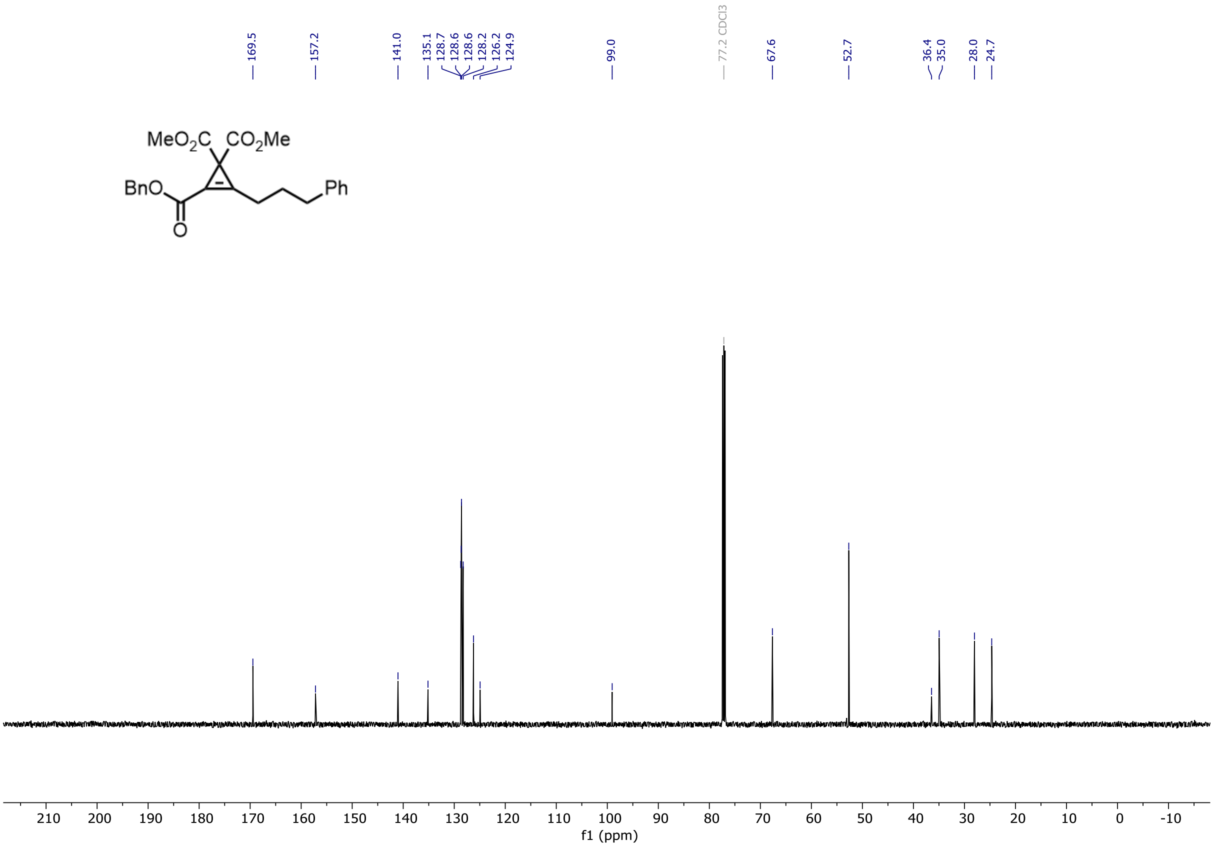

^{1}H NMR (400 MHz, $CDCl_3$) of **15a**

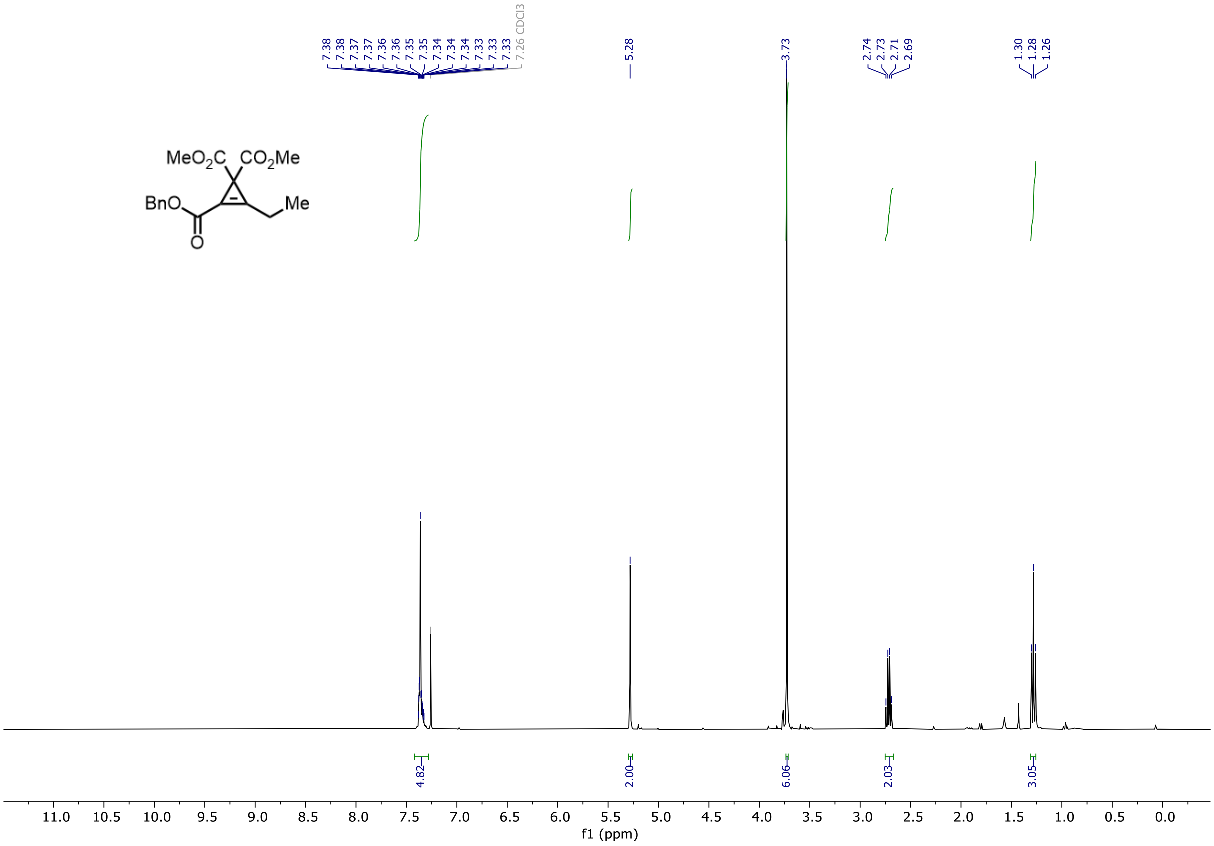

^{13}C NMR (126 MHz, $CDCl_3$) of **15a**

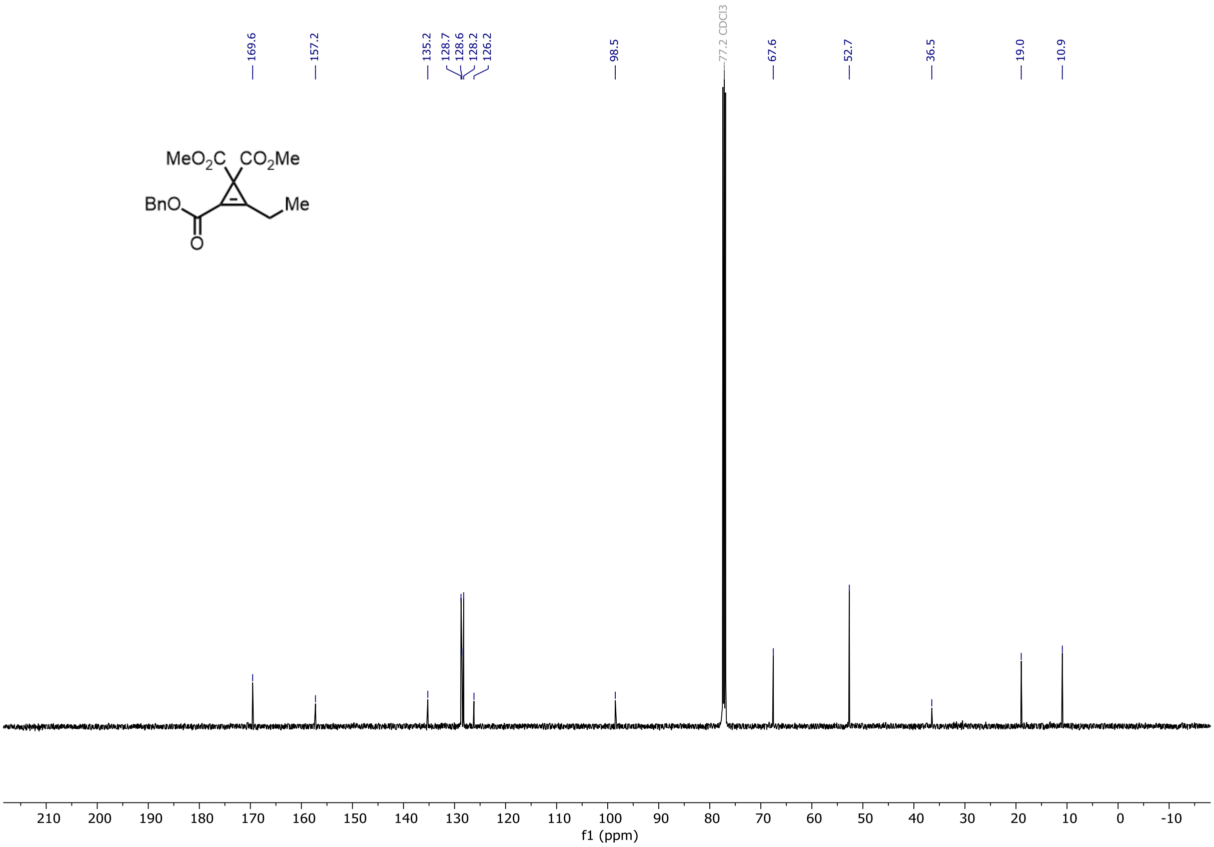

^{1}H NMR (400 MHz, $CDCl_3$) of **16a**

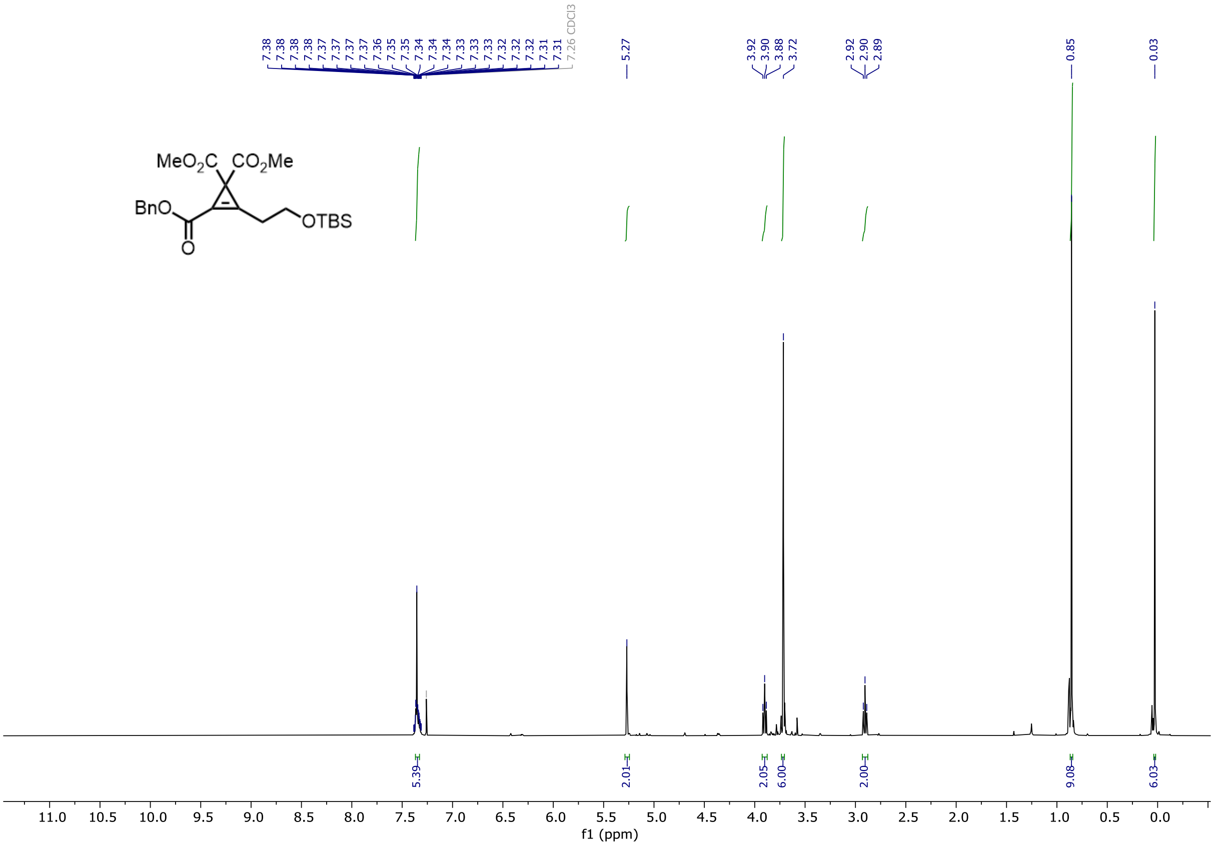

^{13}C NMR (101 MHz, $CDCl_3$) of **16a**

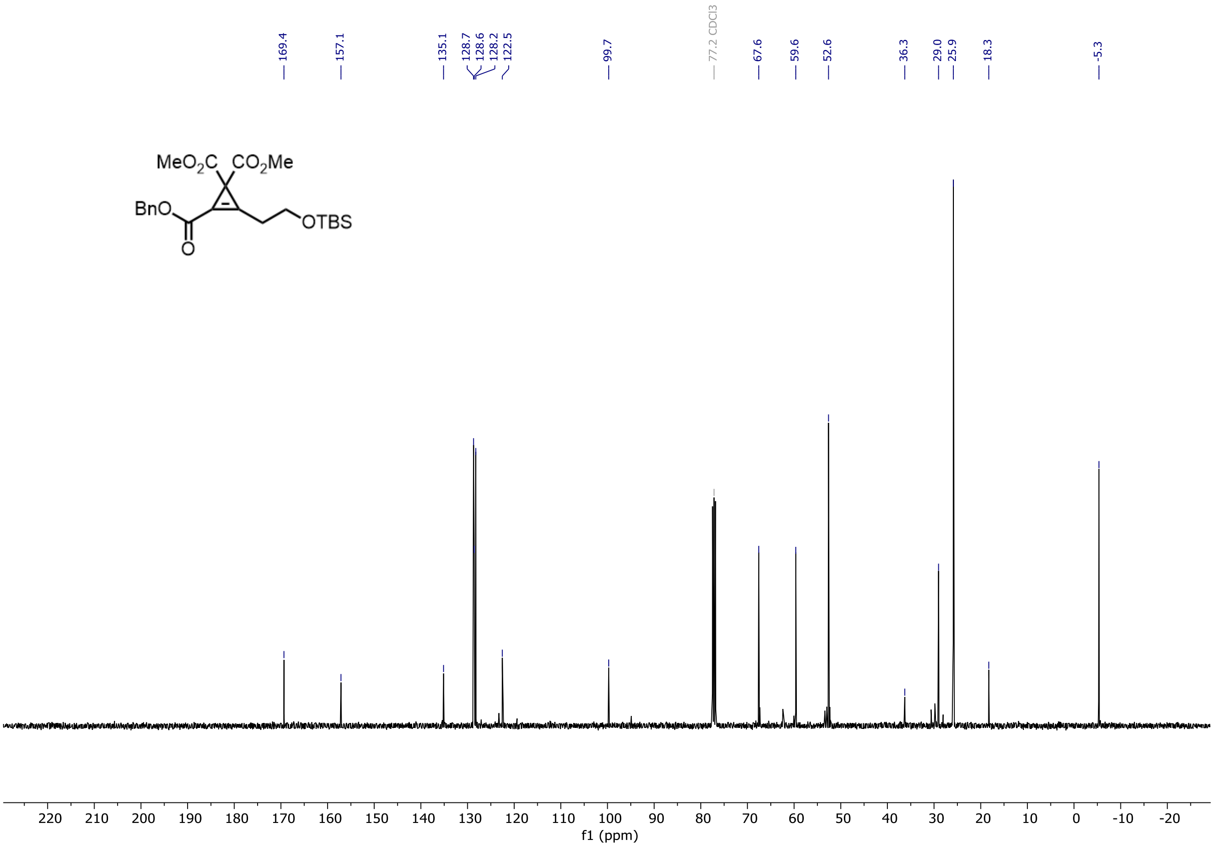

^{1}H NMR (400 MHz, $CDCl_3$) of **17a**

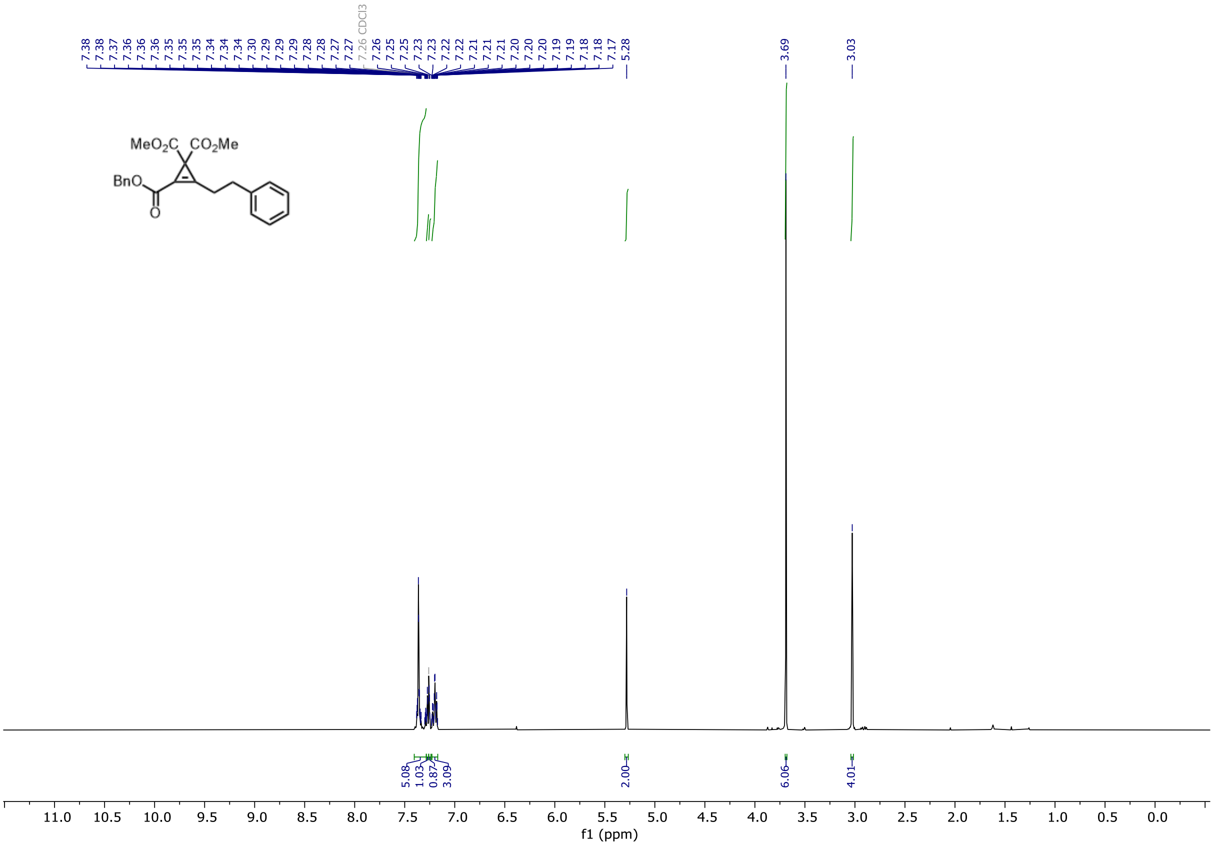

^{13}C NMR (101 MHz, $CDCl_3$) of **17a**

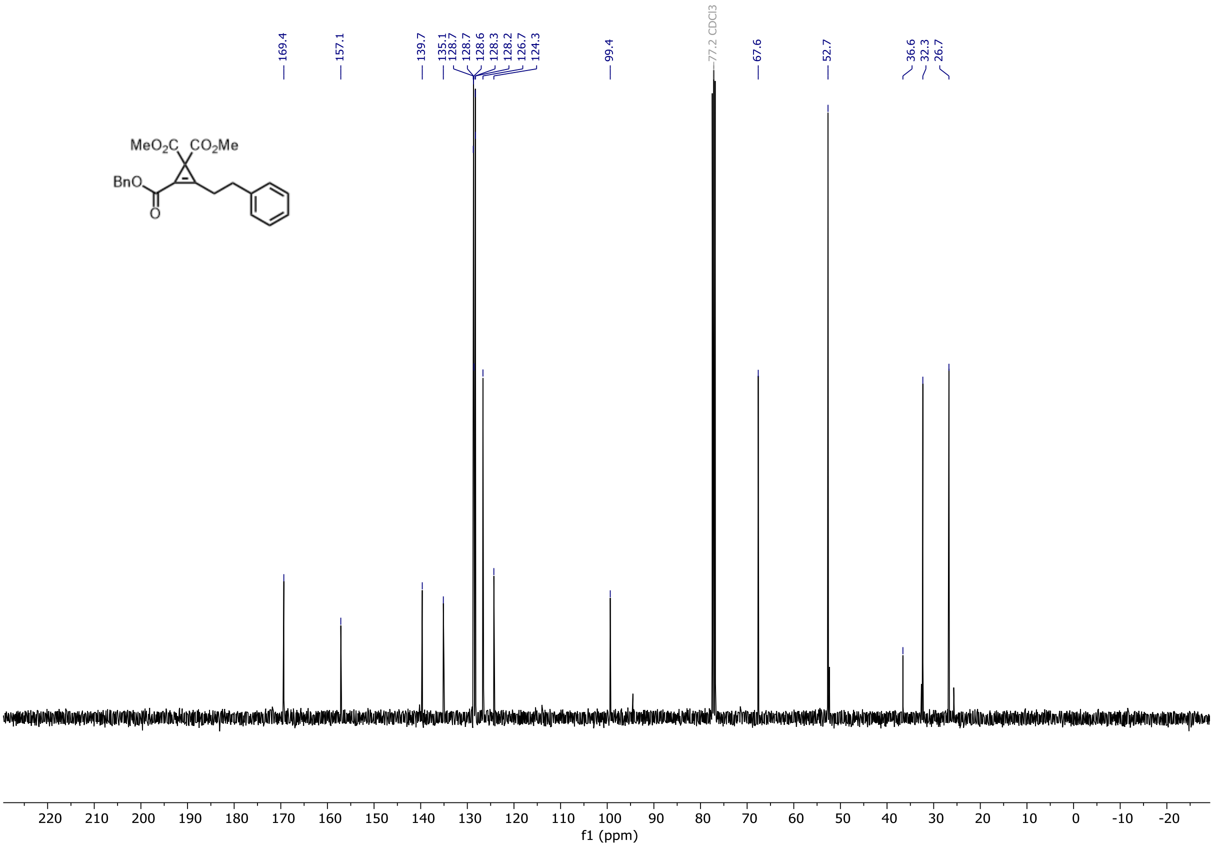

^{1}H NMR (400 MHz, $CDCl_3$) of **18a**

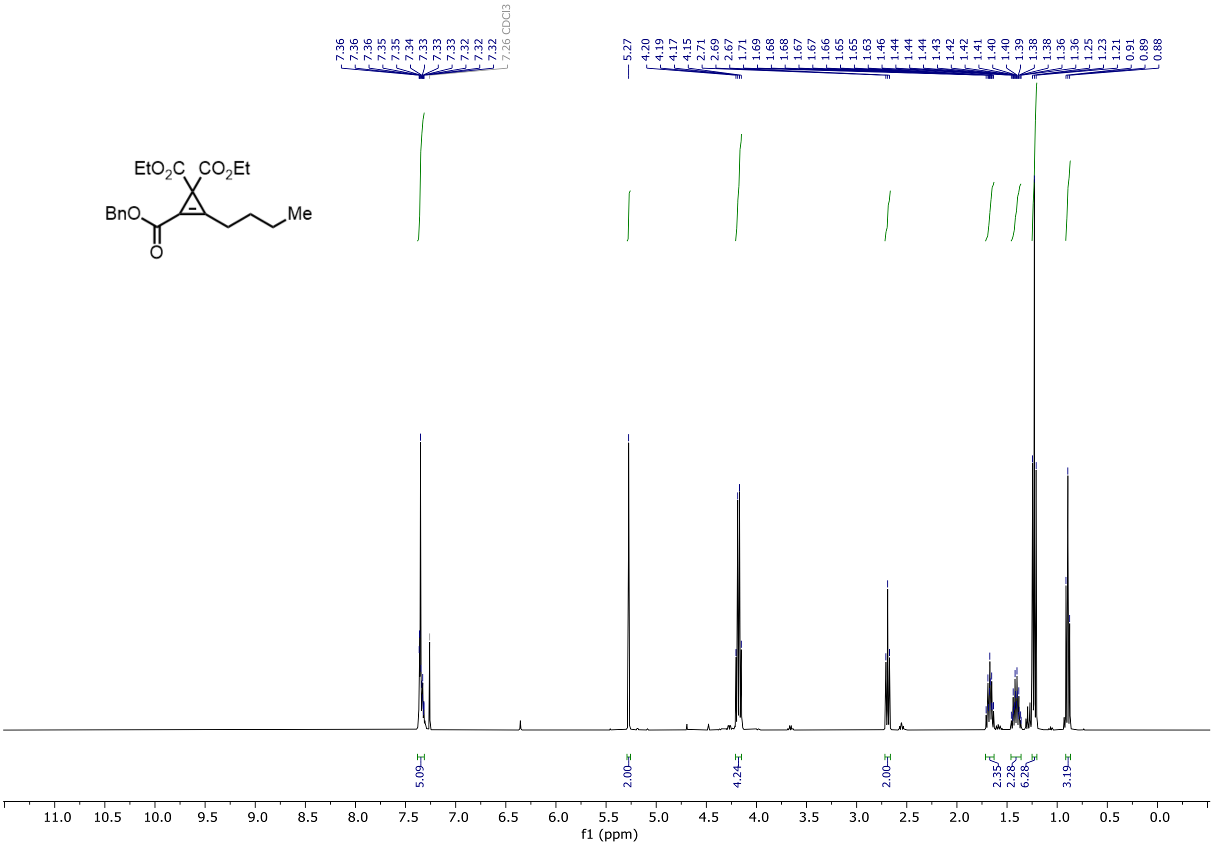

^{13}C NMR (101 MHz, $CDCl_3$) of **18a**

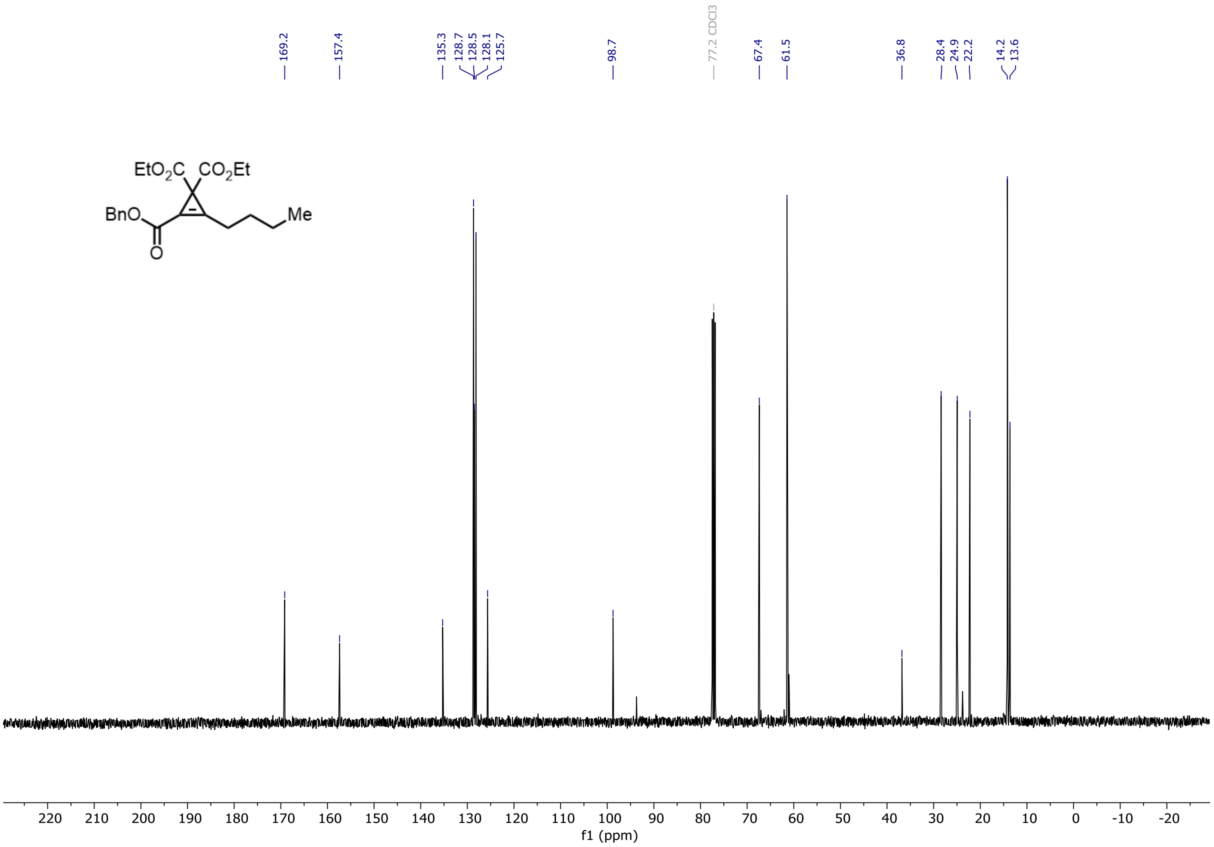

^{1}H NMR (400 MHz, $CDCl_3$) of **19a**

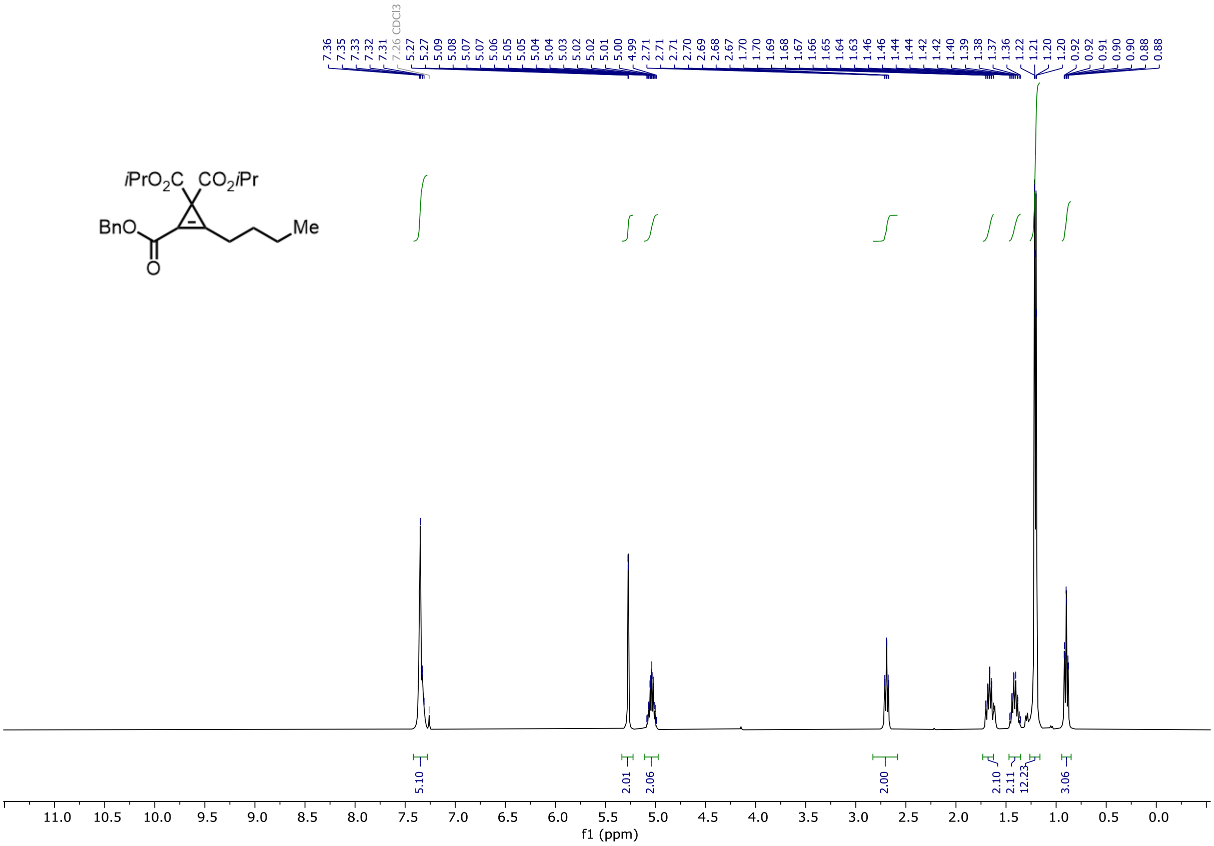

^{13}C NMR (101 MHz, $CDCl_3$) of **19a**

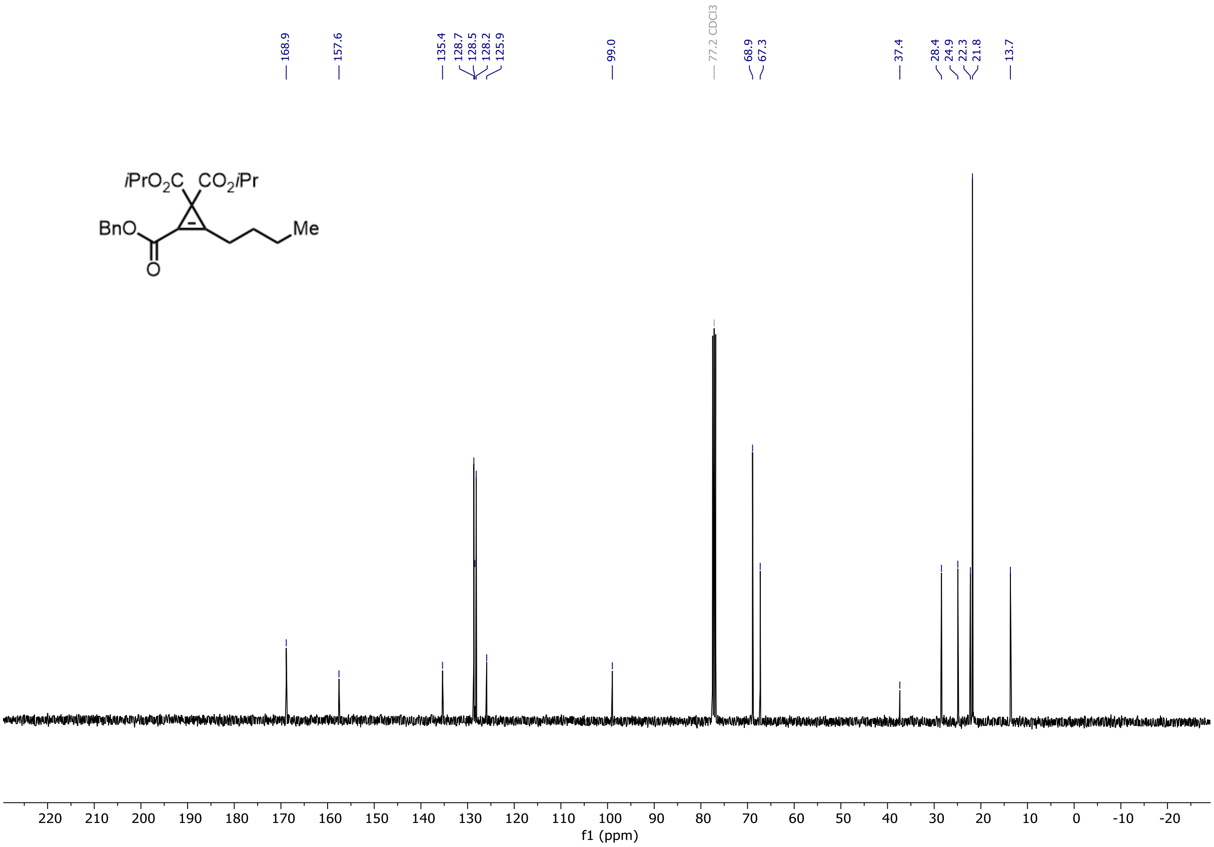

^{1}**H NMR** (400 MHz, $CDCl_3$) of **20a**

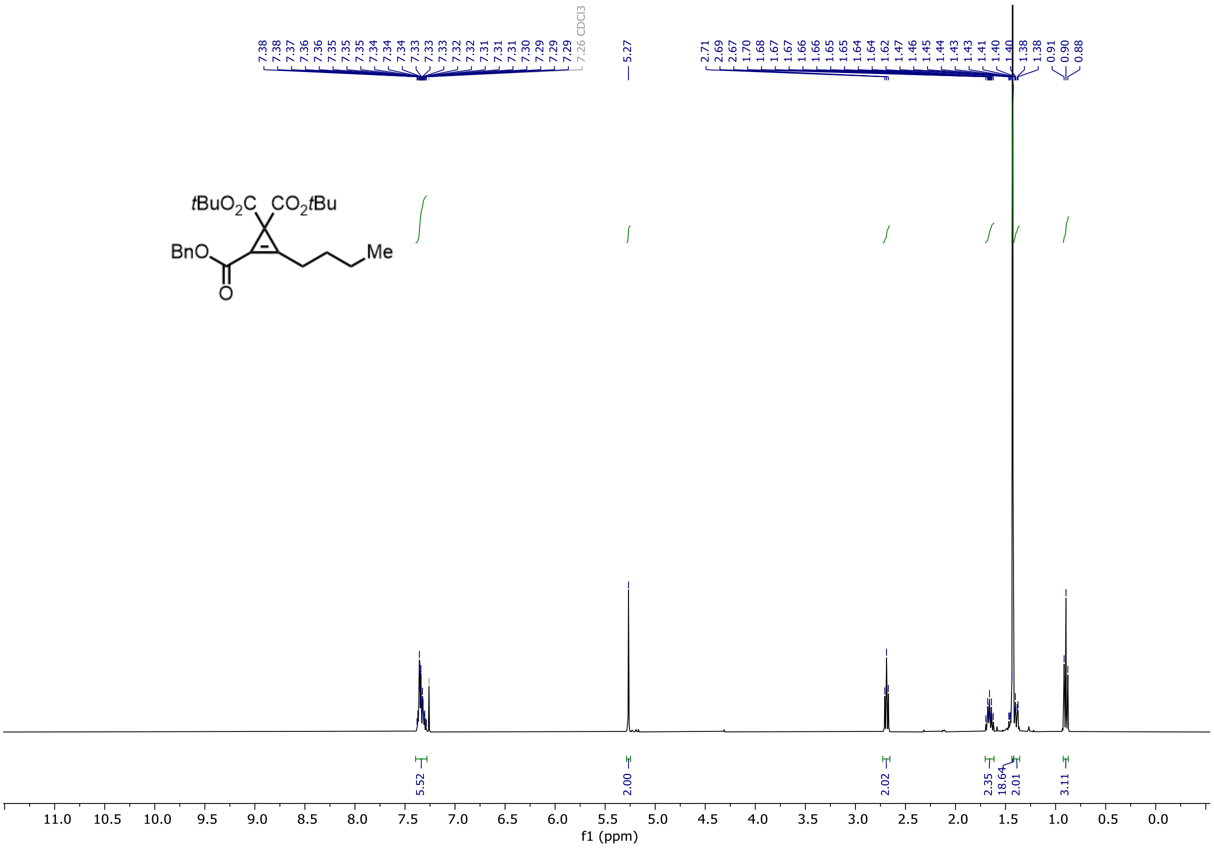

^{13}**C NMR** (101 MHz, $CDCl_3$) of **20a**

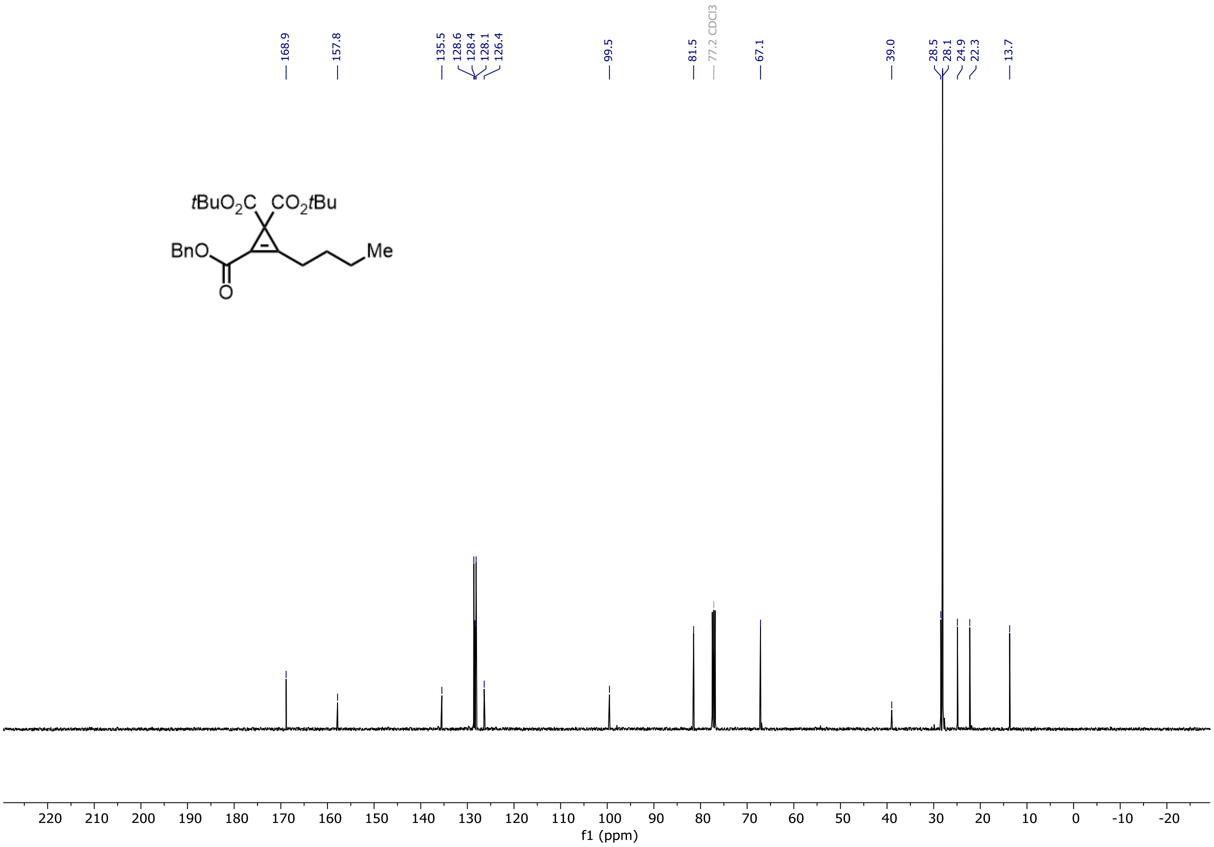

^{1}H NMR (400 MHz, $CDCl_3$) of **21a**

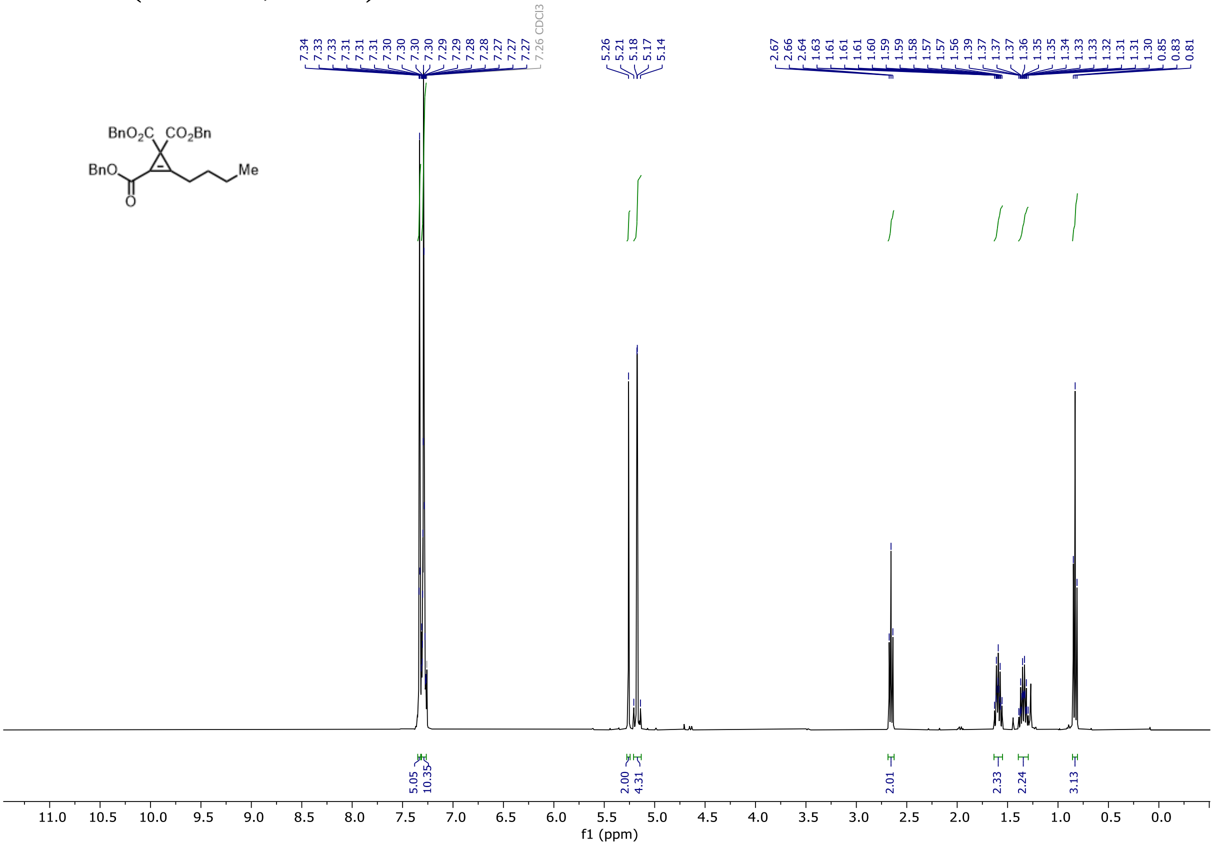

^{13}C NMR (101 MHz, $CDCl_3$) of **21a**

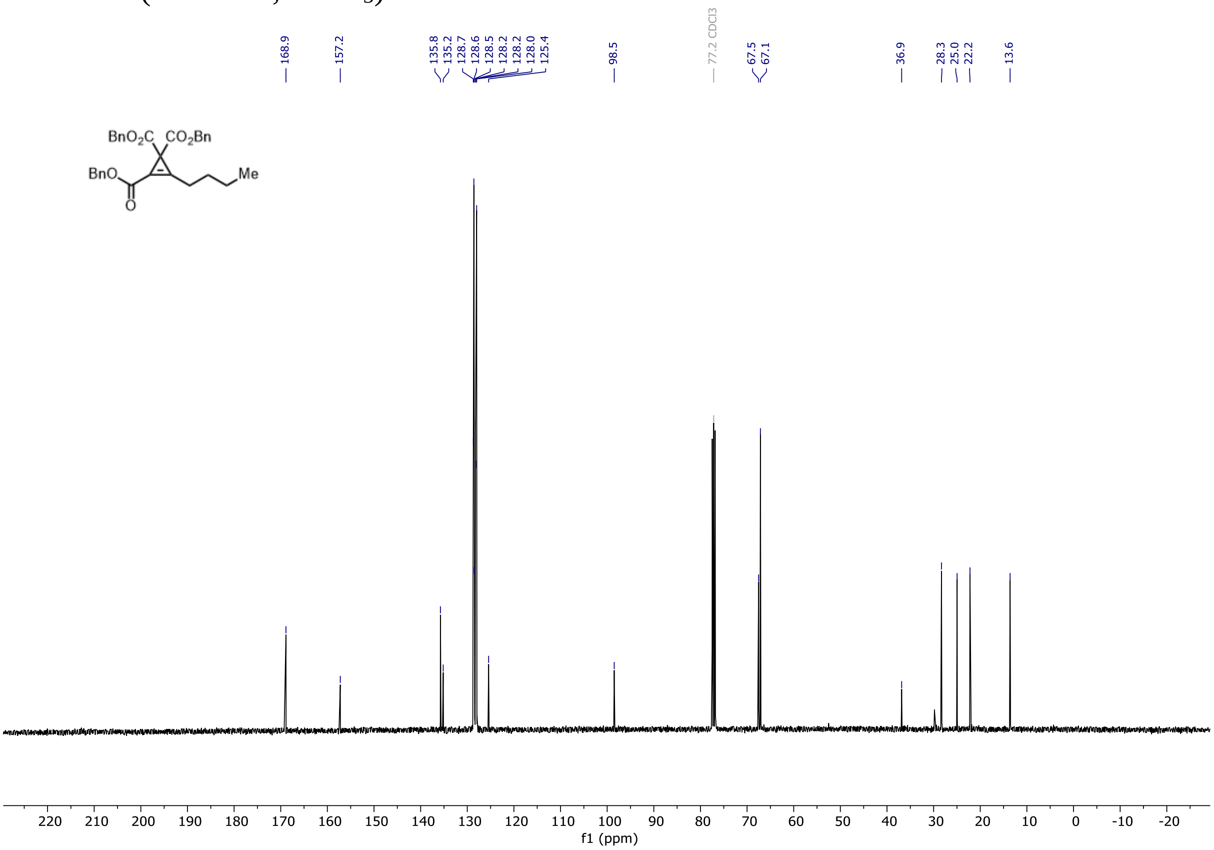

^{1}H NMR (400 MHz, $CDCl_3$) of **S25**

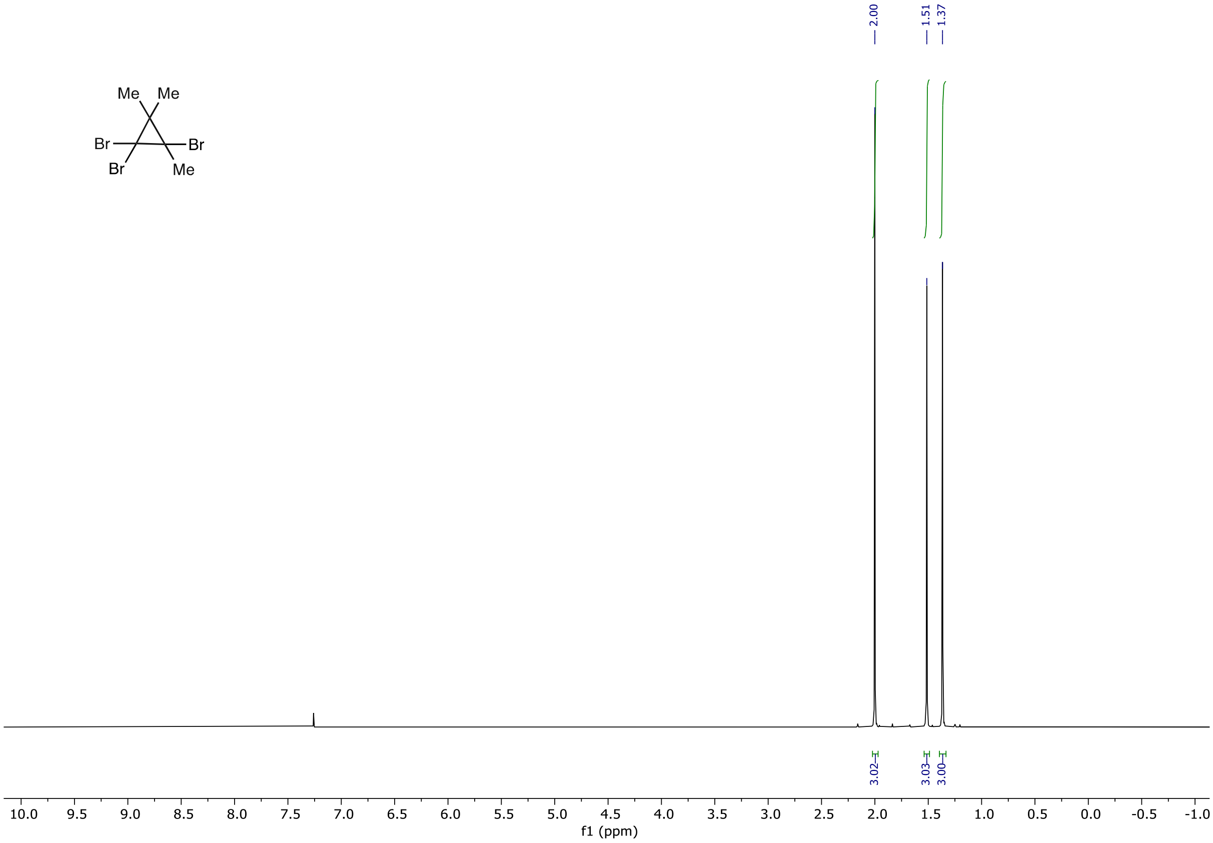

^{13}C NMR (101 MHz, $CDCl_3$) of **S25**

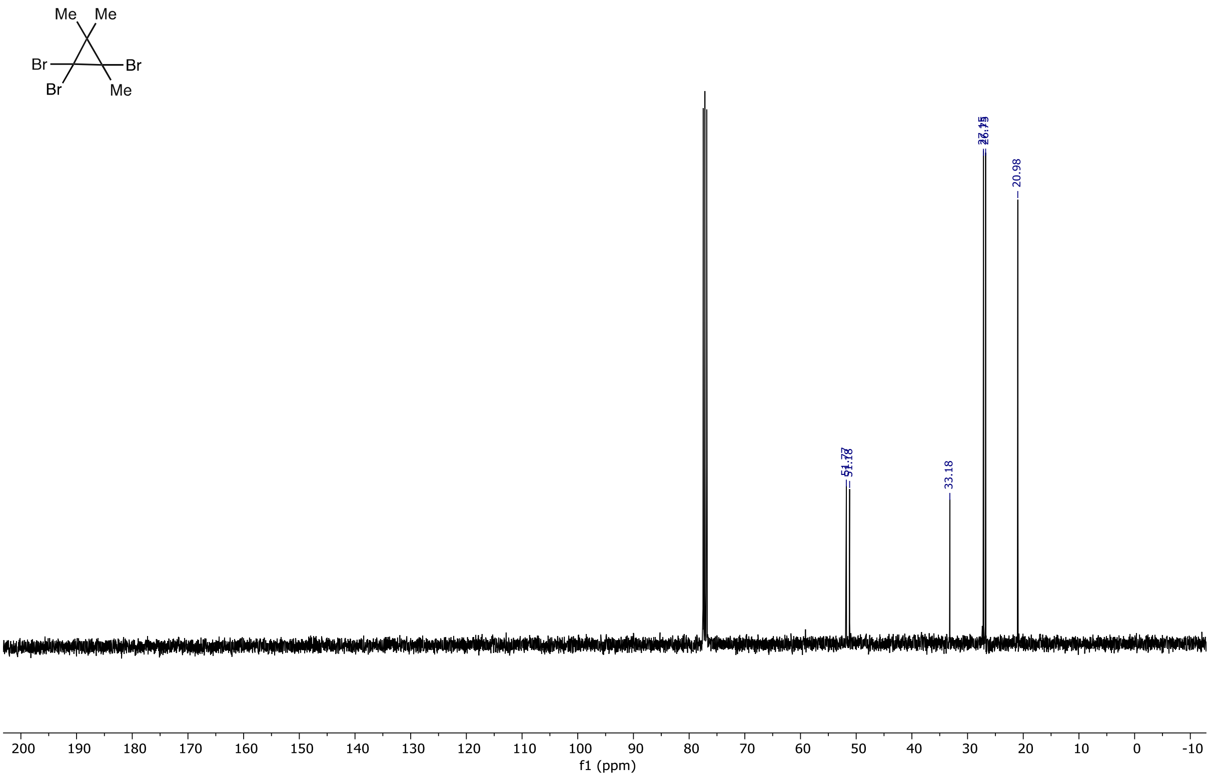

^{1}H NMR (500 MHz, $CDCl_3$) of **22a**

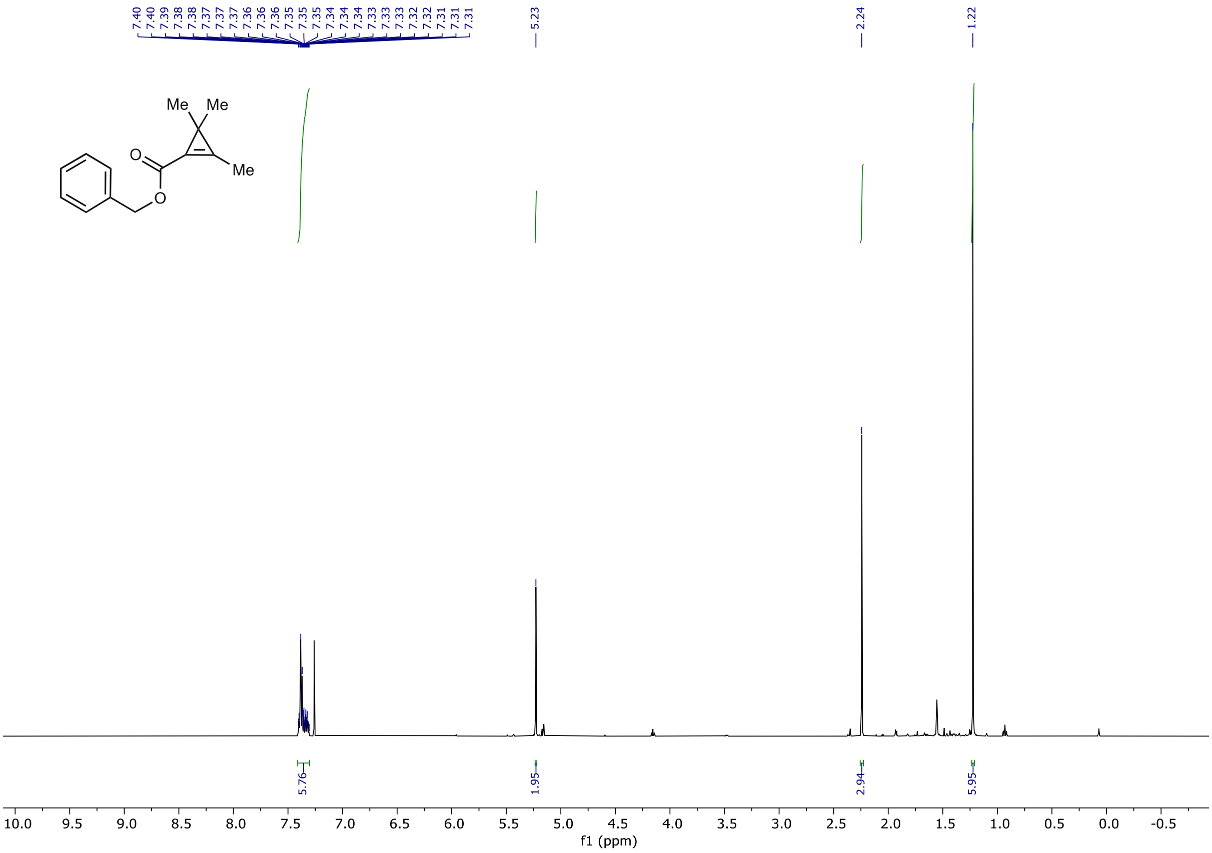

^{13}C NMR (126 MHz, $CDCl_3$) of **22a**

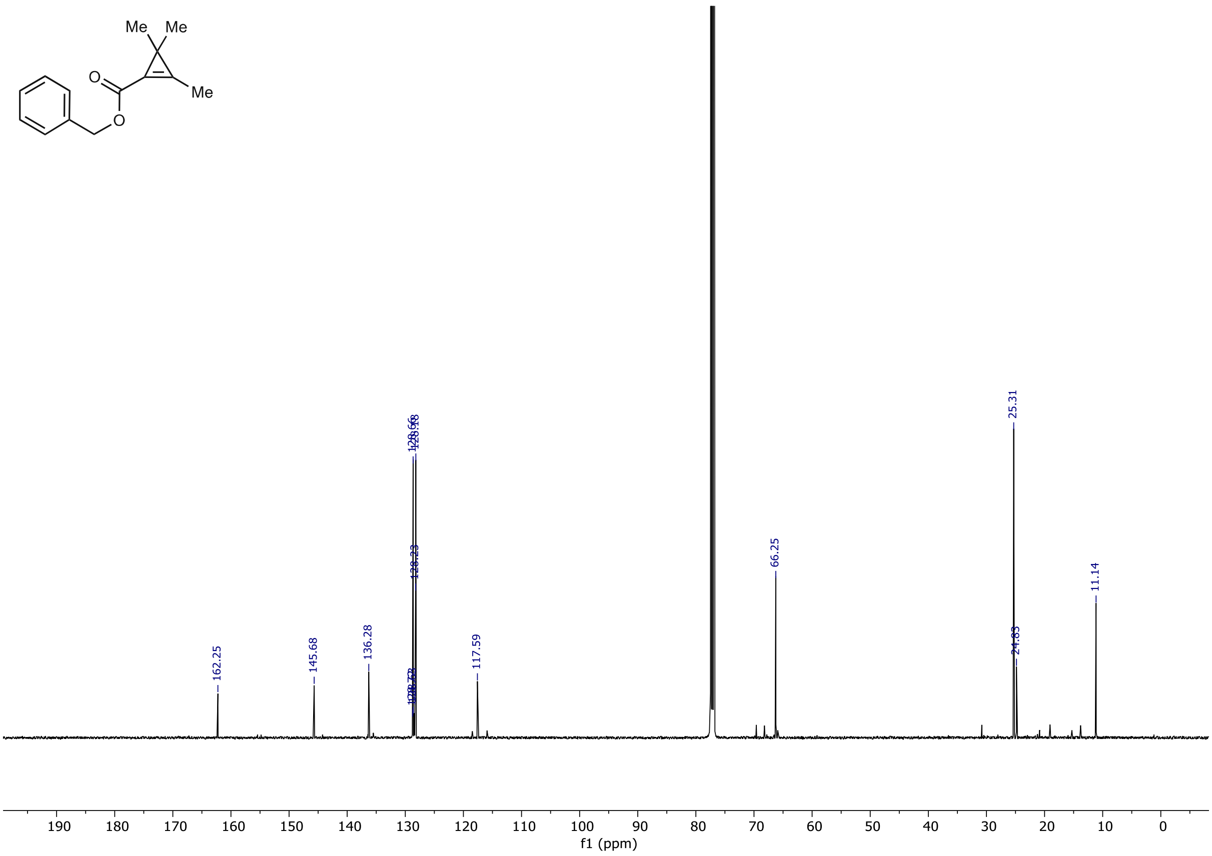

^{1}**H NMR** (500 MHz, $CDCl_3$) of **23a**

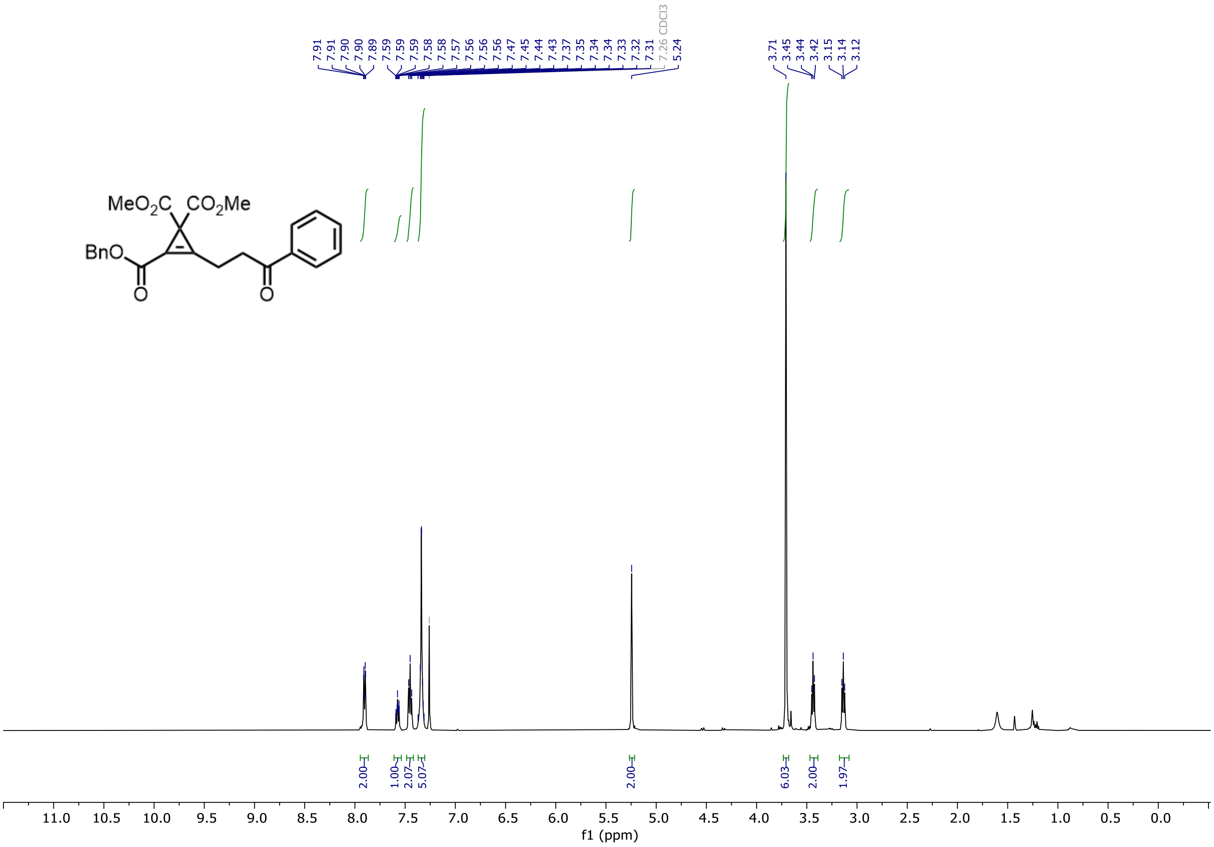

^{13}**C NMR** (126 MHz, $CDCl_3$) of **23a**

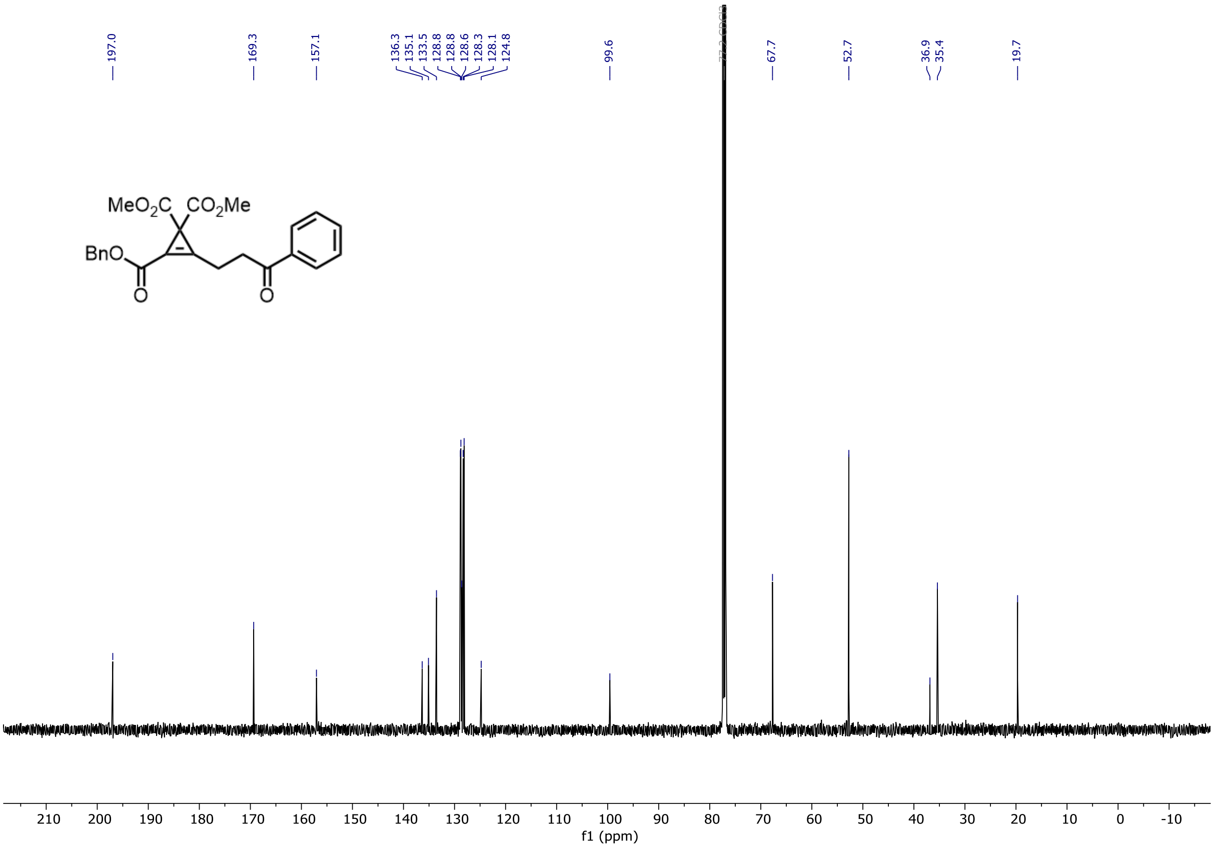

^{1}H NMR (400 MHz, $CDCl_3$) of **24a**

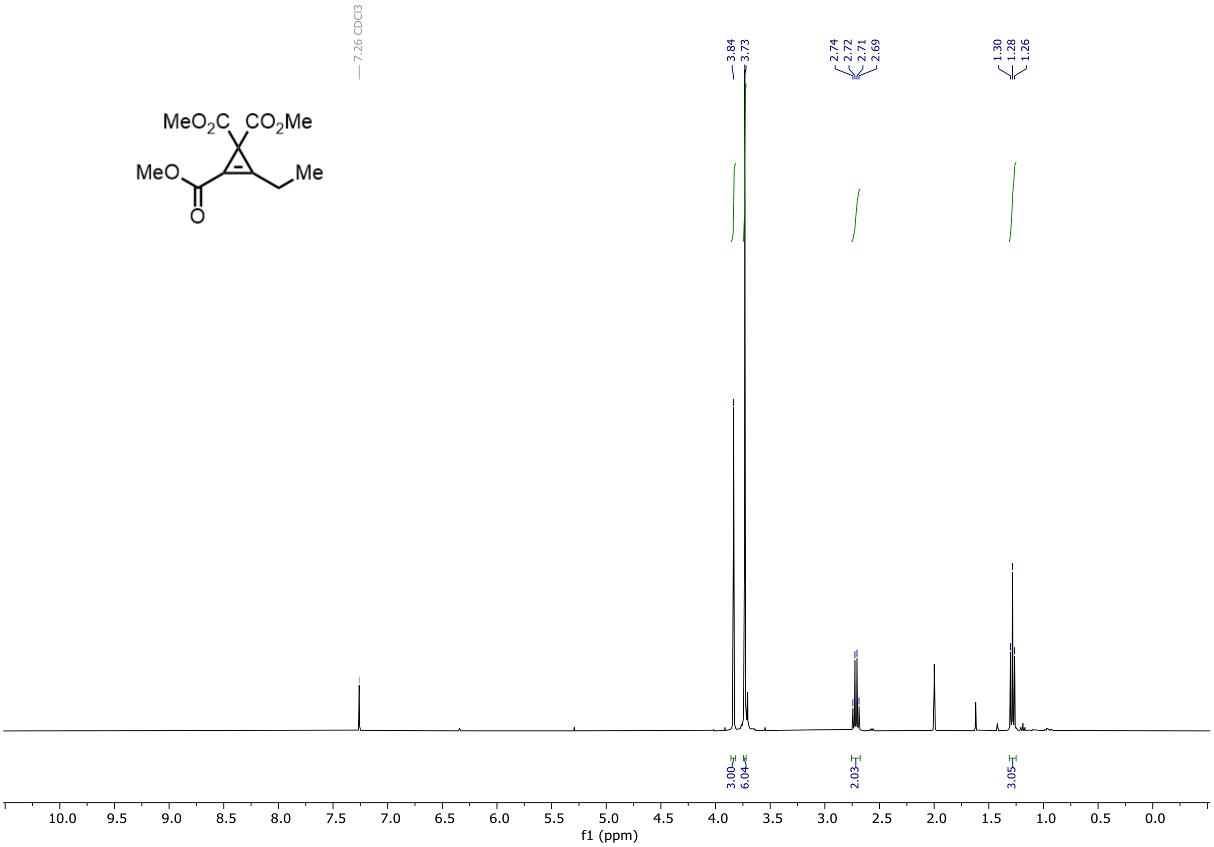

^{13}C NMR (126 MHz, $CDCl_3$) of **24a**

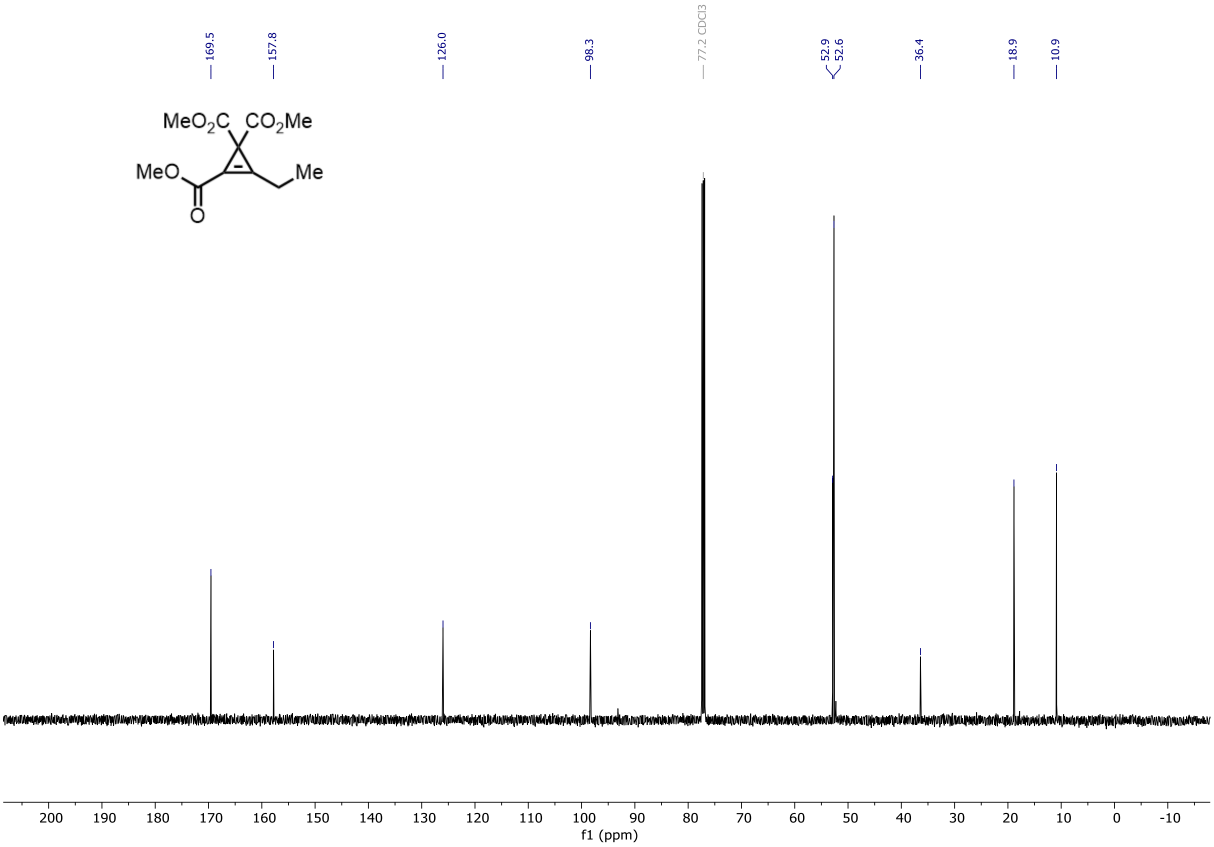

^{1}H NMR (400 MHz, $CDCl_3$) of **S26**

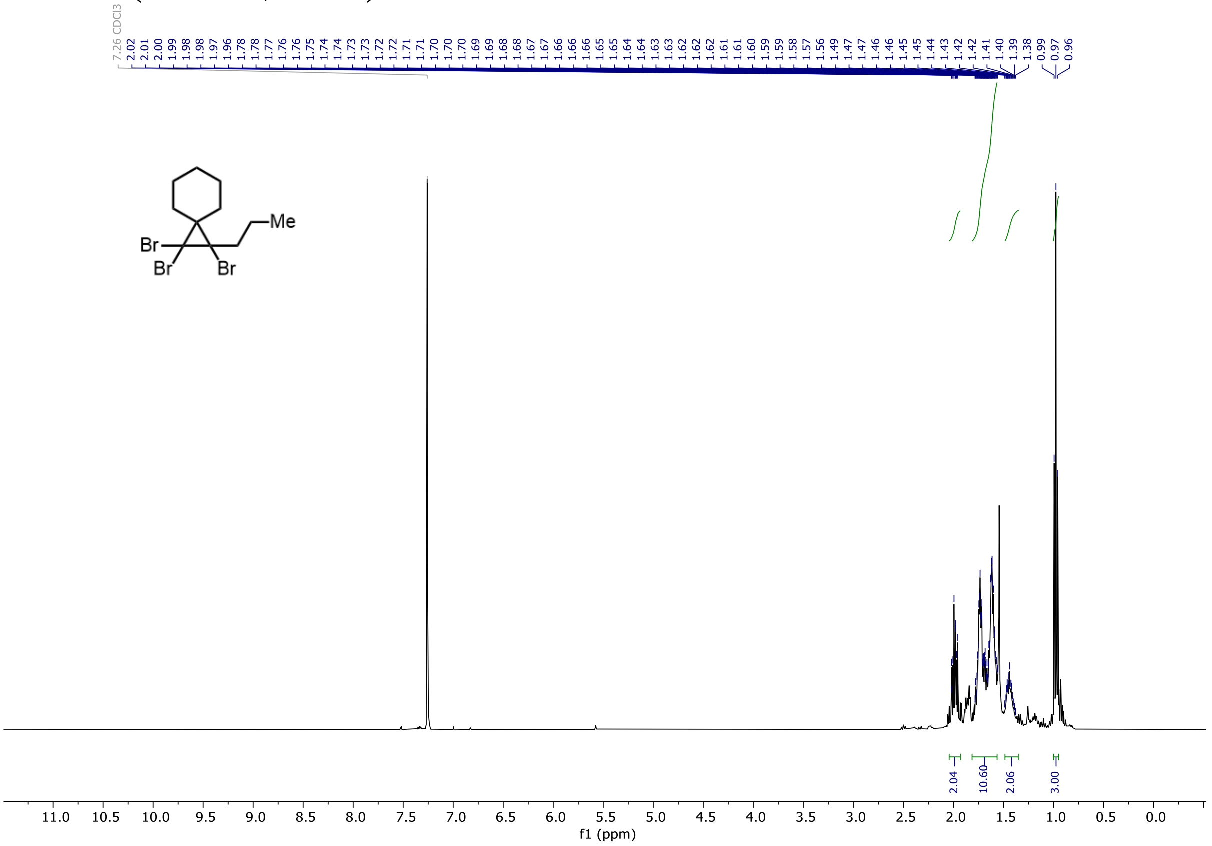

^{13}C NMR (126 MHz, $CDCl_3$) of **S26**

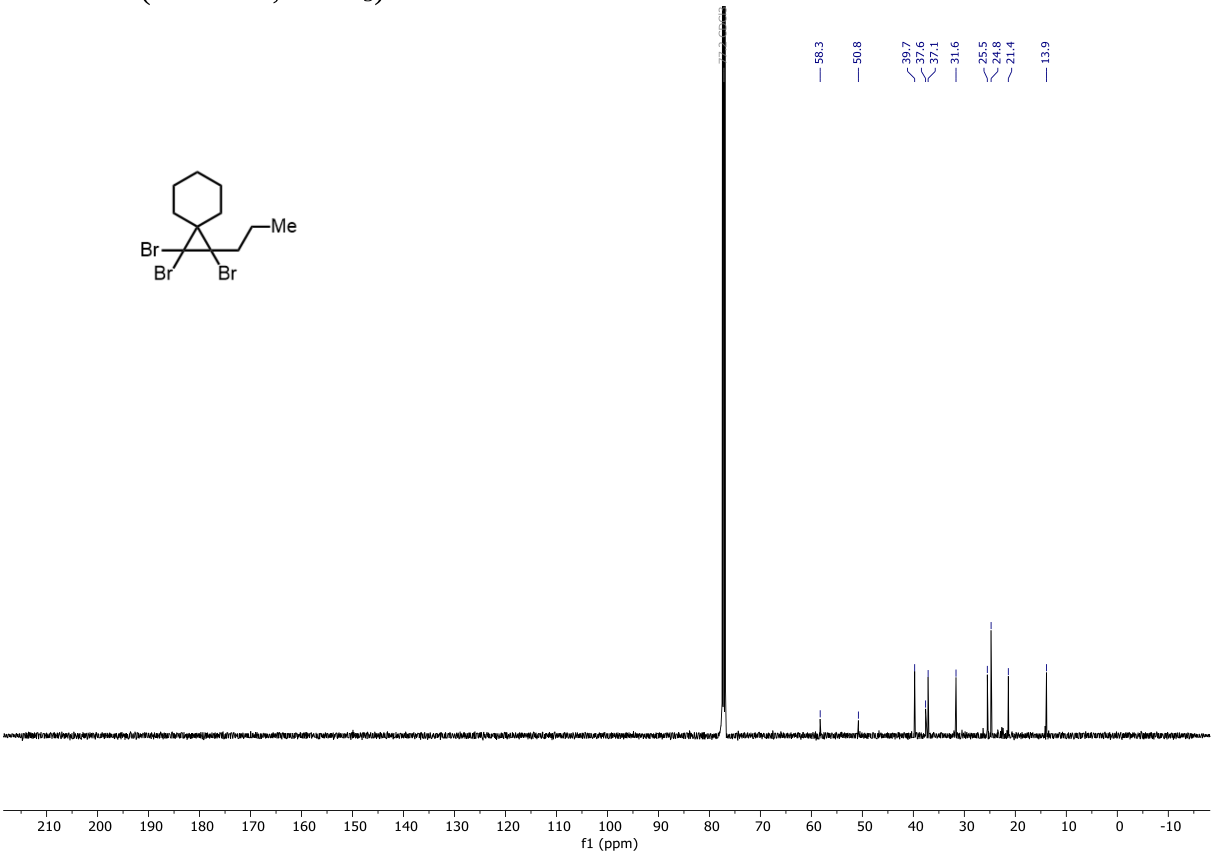

^{1}H NMR (400 MHz, $CDCl_3$) of **38a**

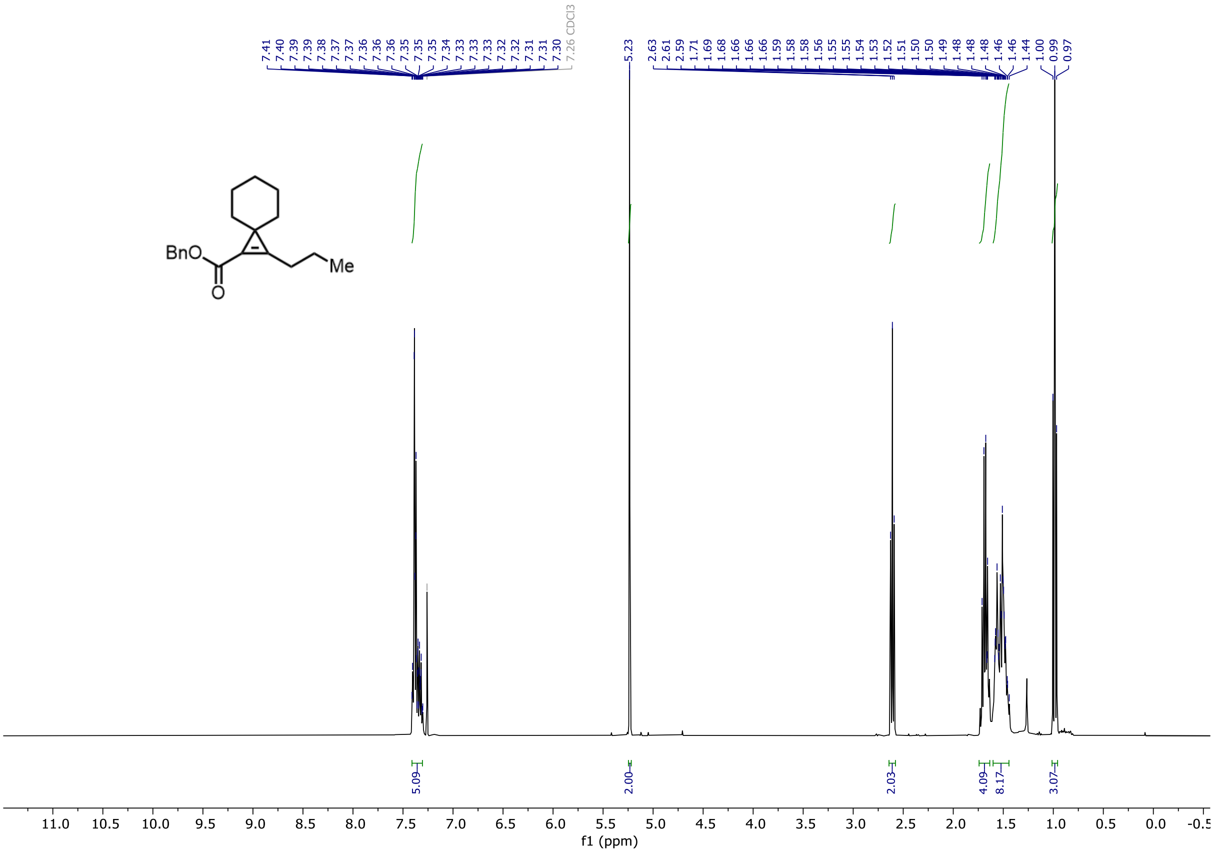

^{13}C NMR (101 MHz, $CDCl_3$) of **38a**

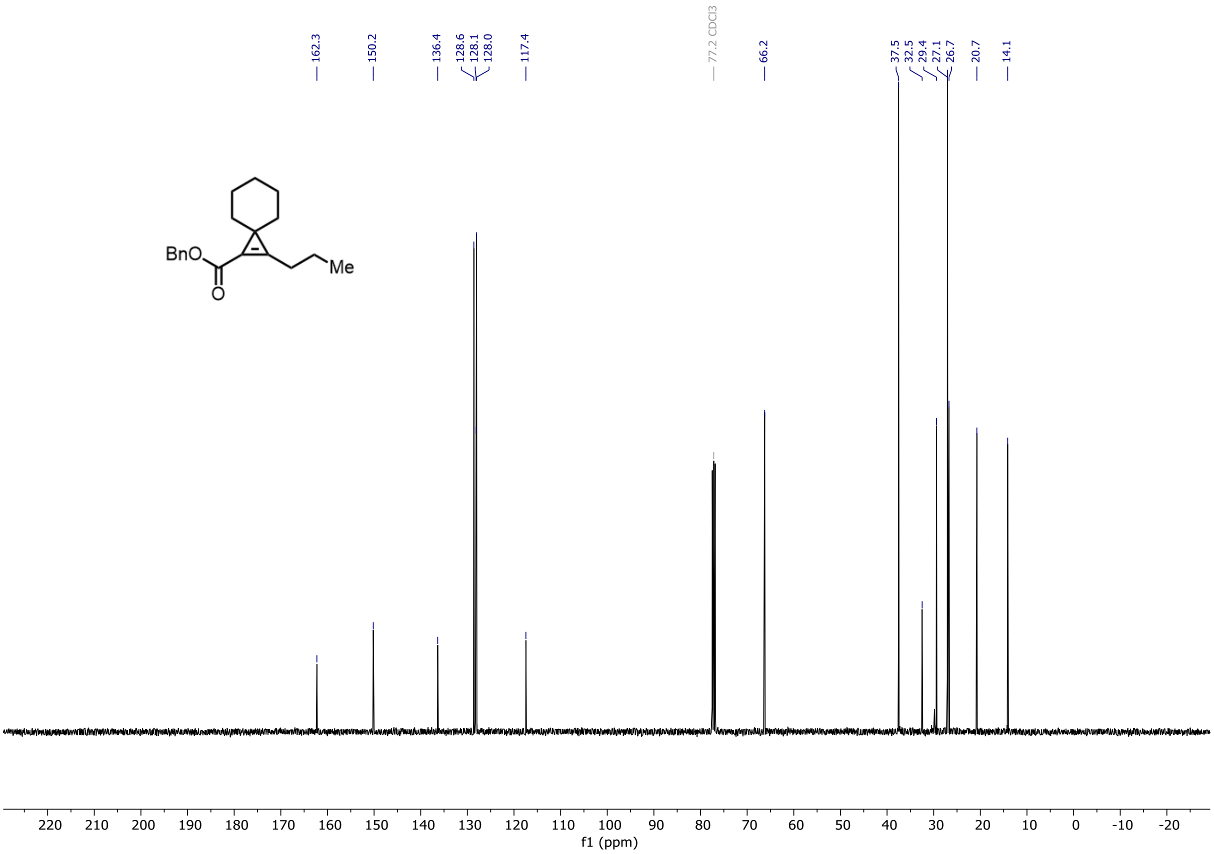

¹H NMR (400 MHz, $CDCl_3$) of **25a**

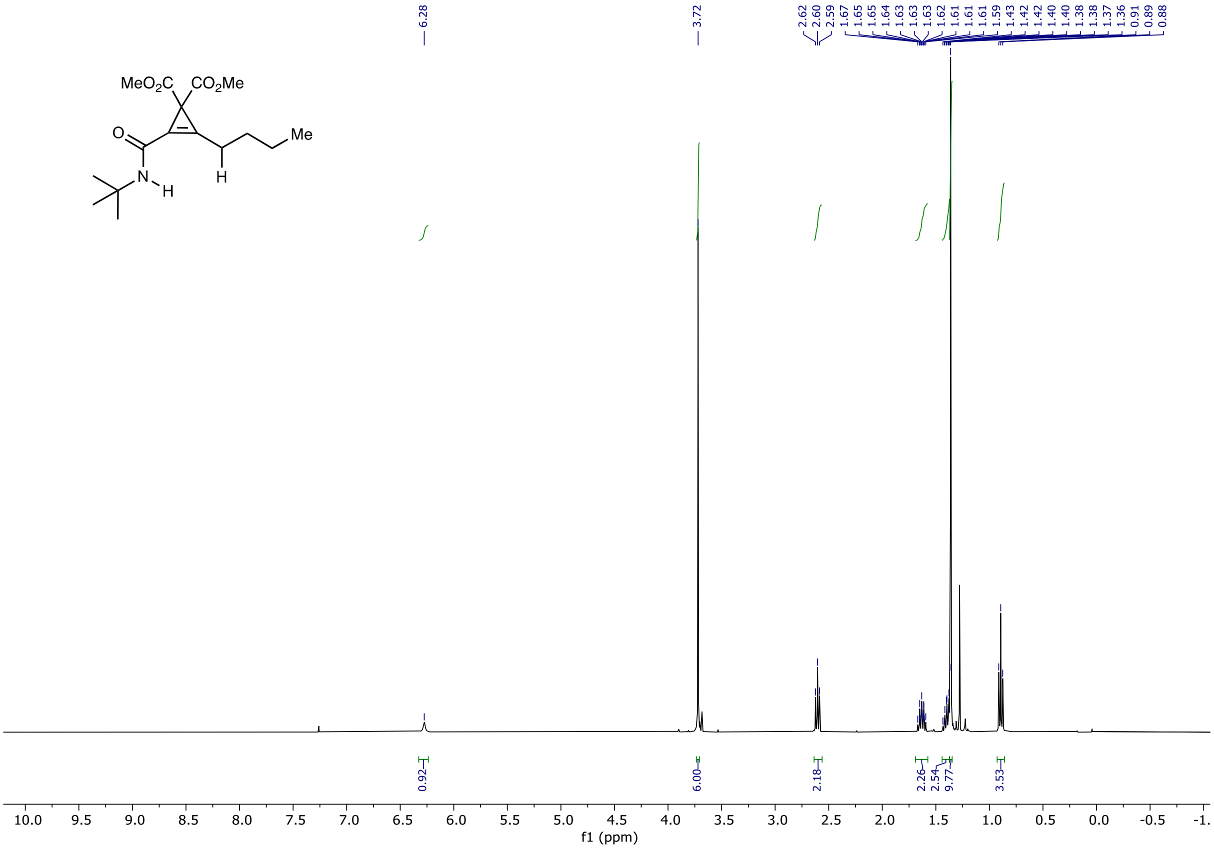

¹³C NMR (101 MHz, $CDCl_3$) of **25a**

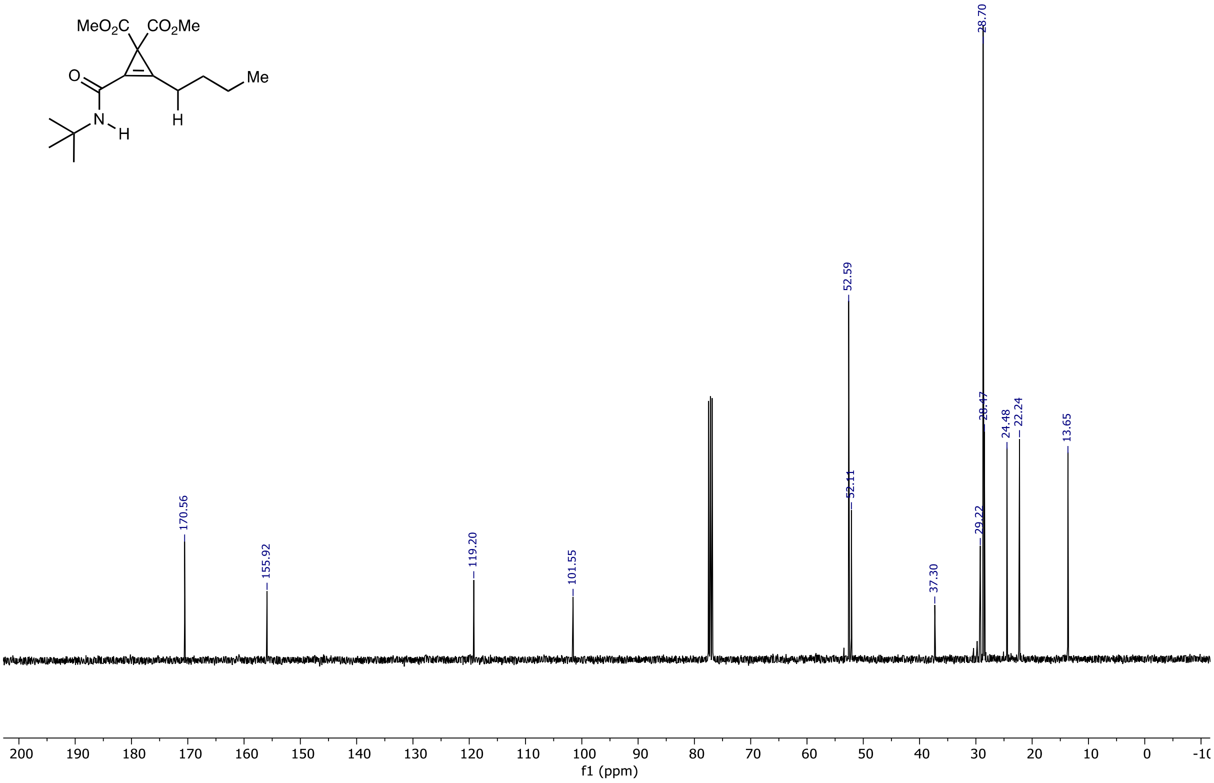

^{1}H NMR (500 MHz, $CDCl_3$) of **26a**

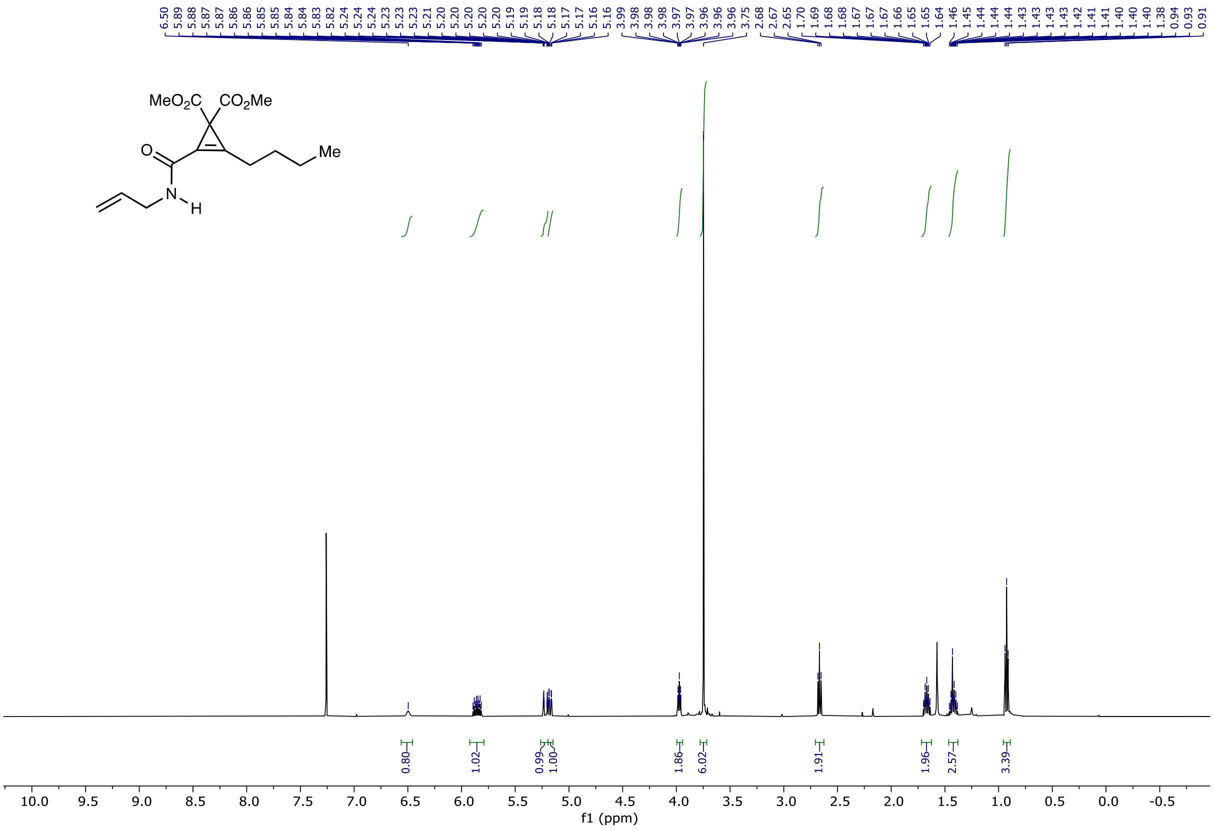

^{13}C NMR (126 MHz, $CDCl_3$) of **26a**

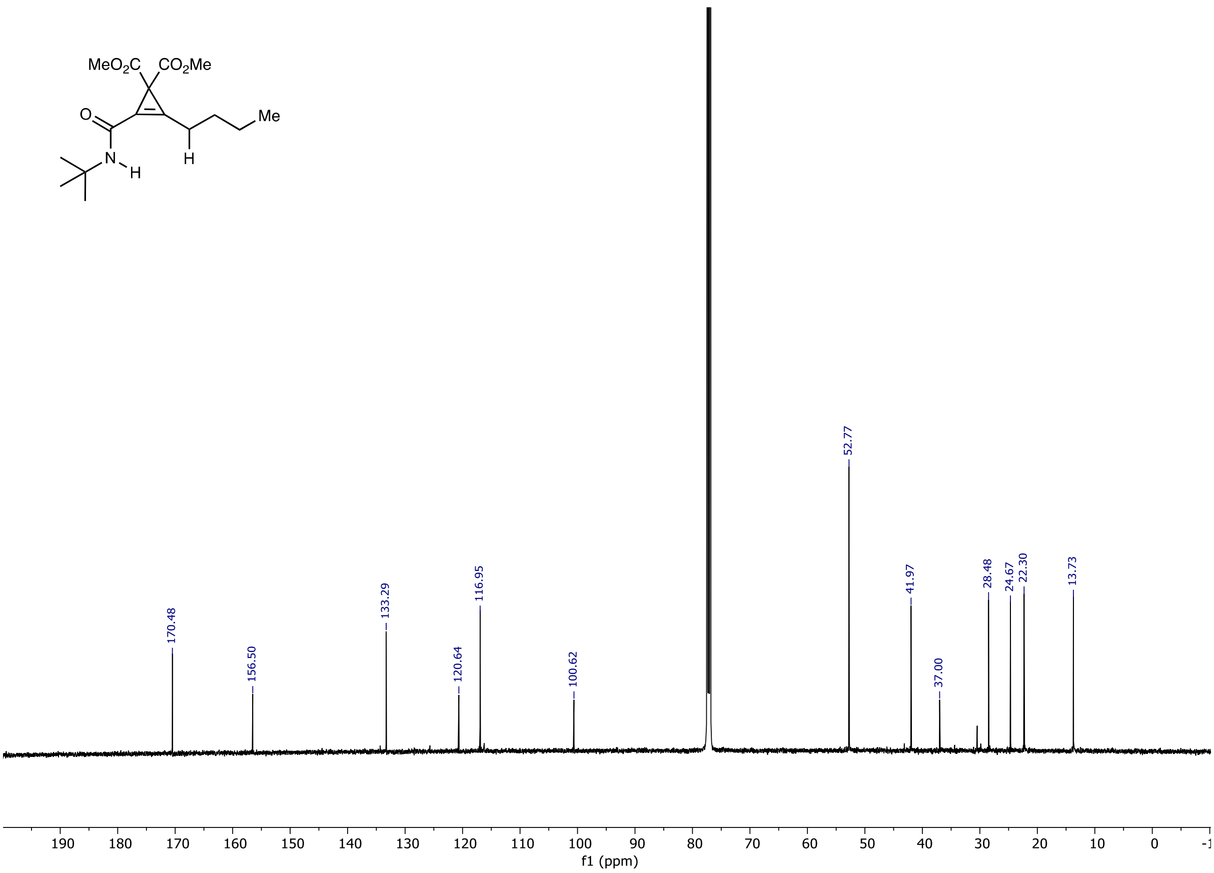

^{1}H NMR (500 MHz, $CDCl_3$) of **27a**

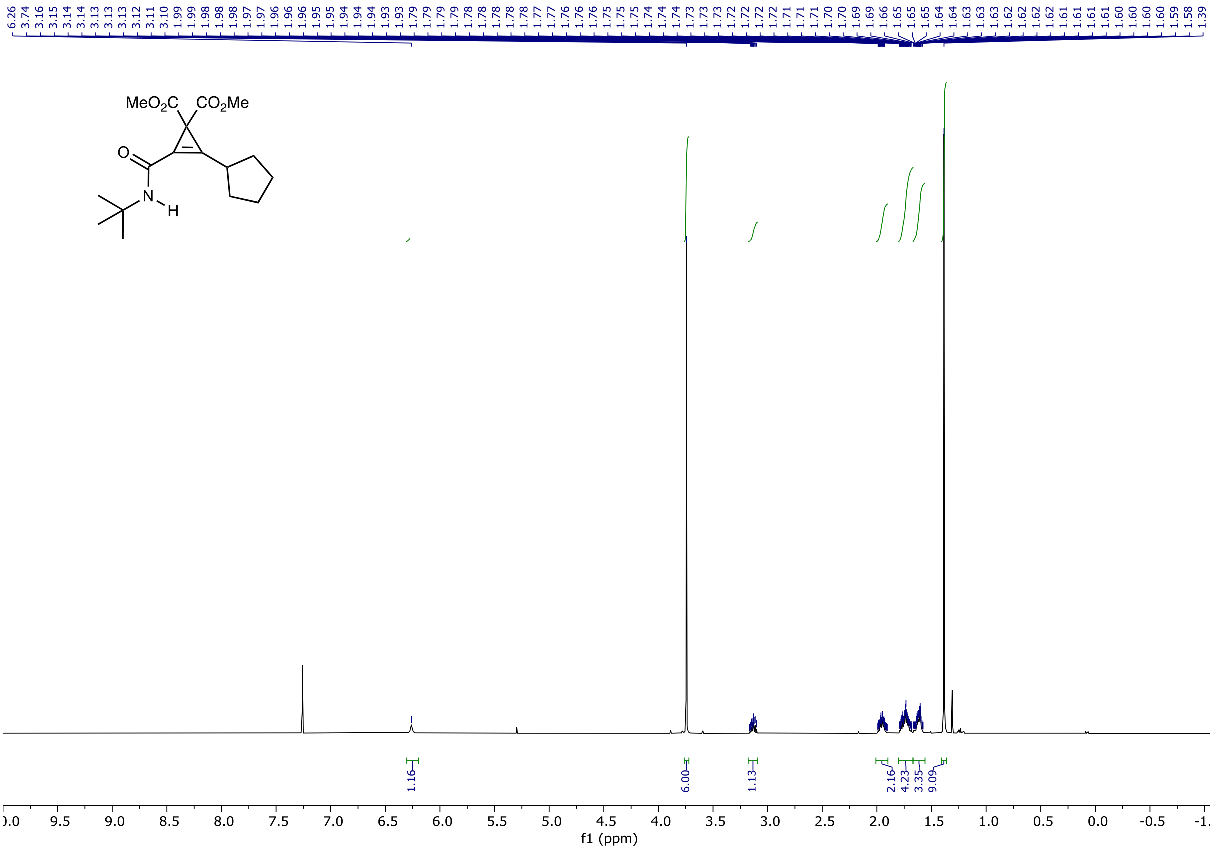

^{13}C NMR (126 MHz, $CDCl_3$) of **27a**

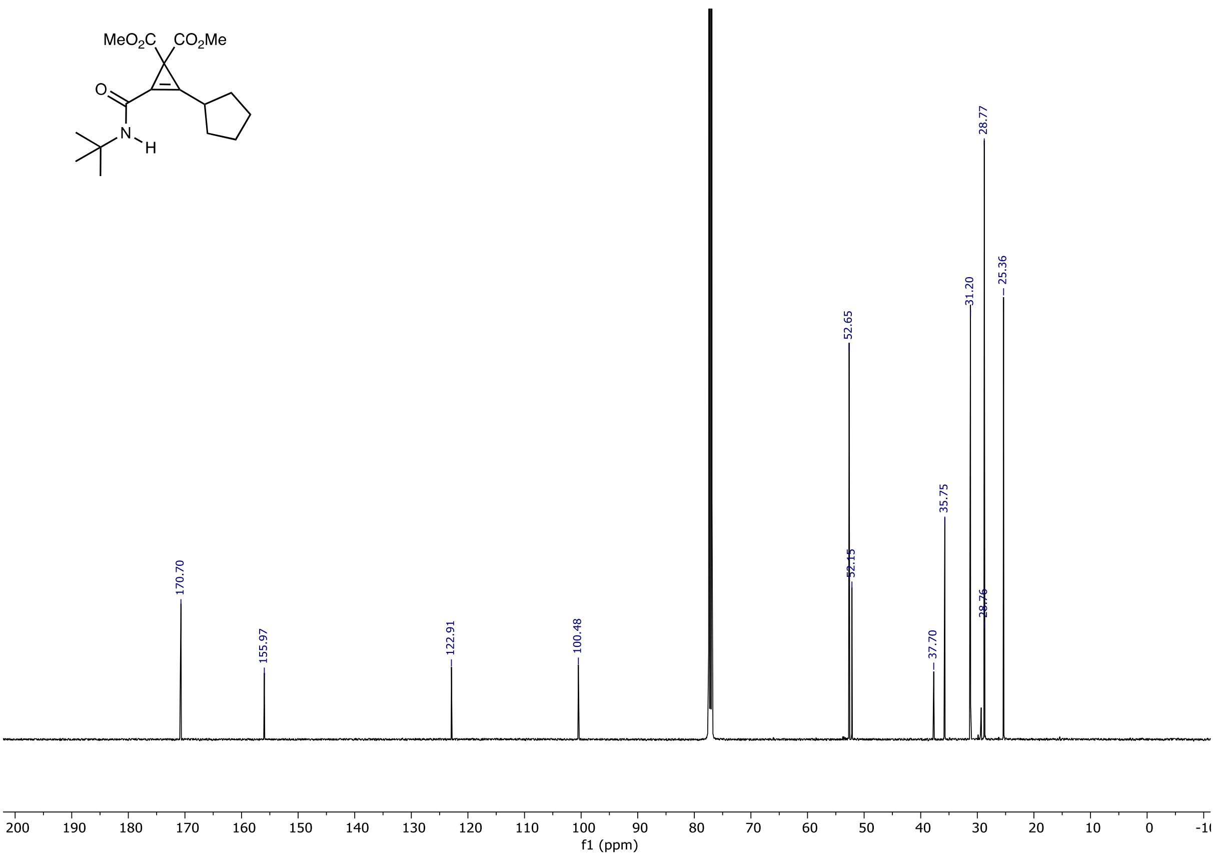

^{1}H NMR (500 MHz, $CDCl_3$) of **28a**

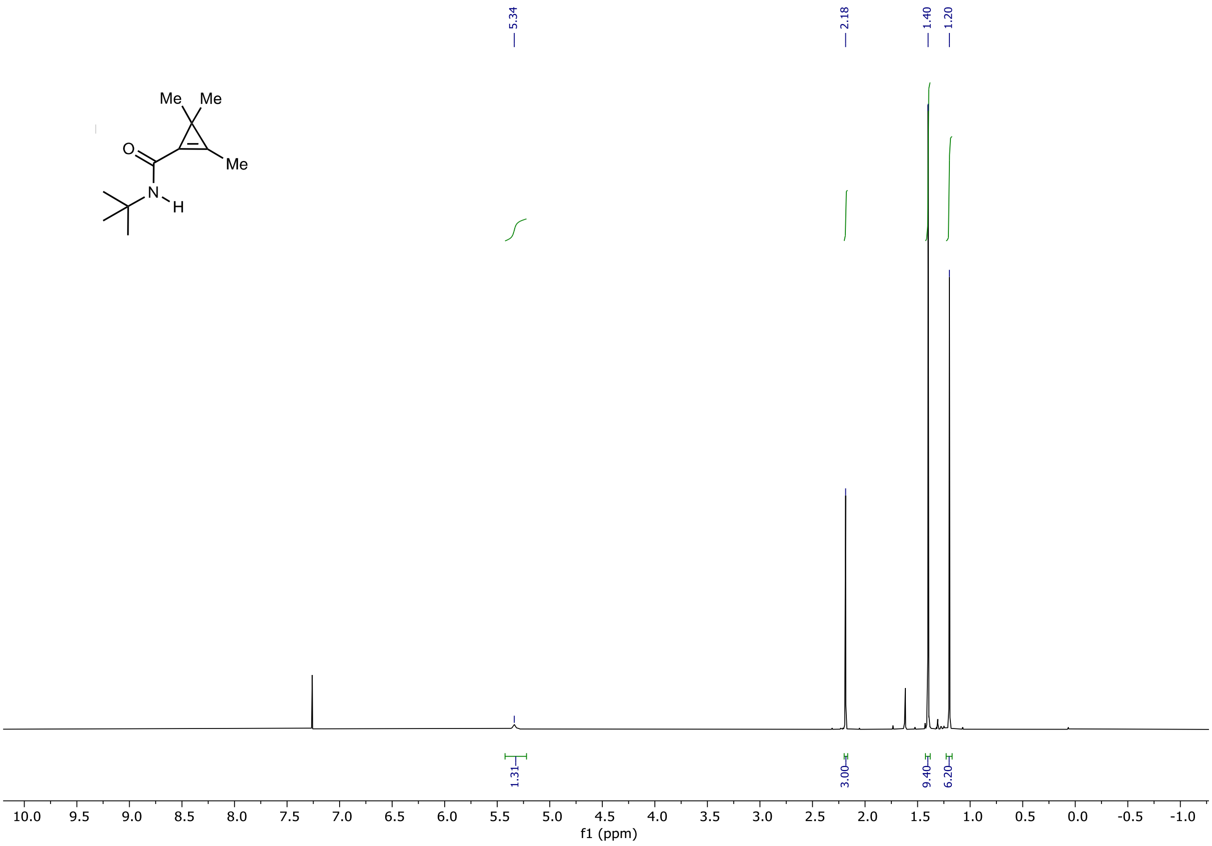

^{13}C NMR (126 MHz, $CDCl_3$) of **28a**

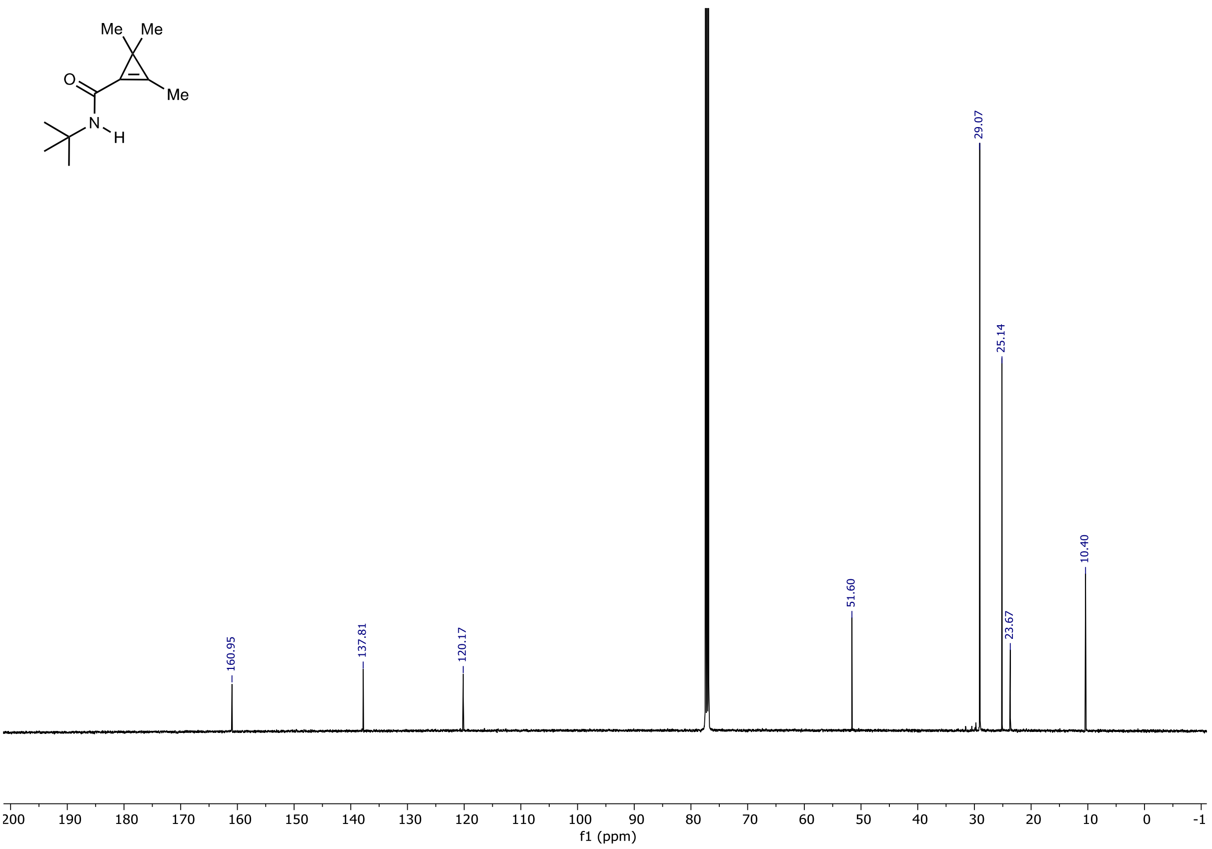

^{1}H NMR (400 MHz, $CDCl_3$) of **29a**

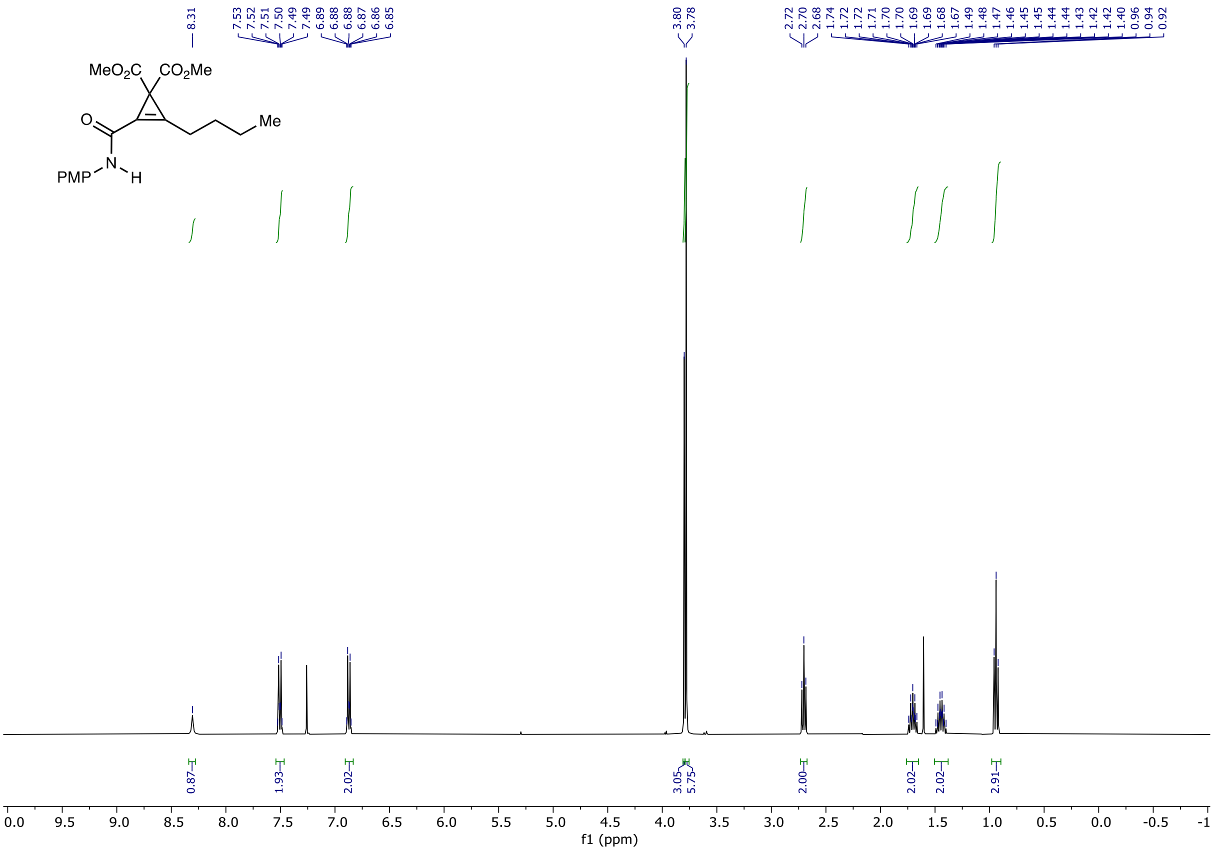

^{13}C NMR (126 MHz, $CDCl_3$) of **29a**

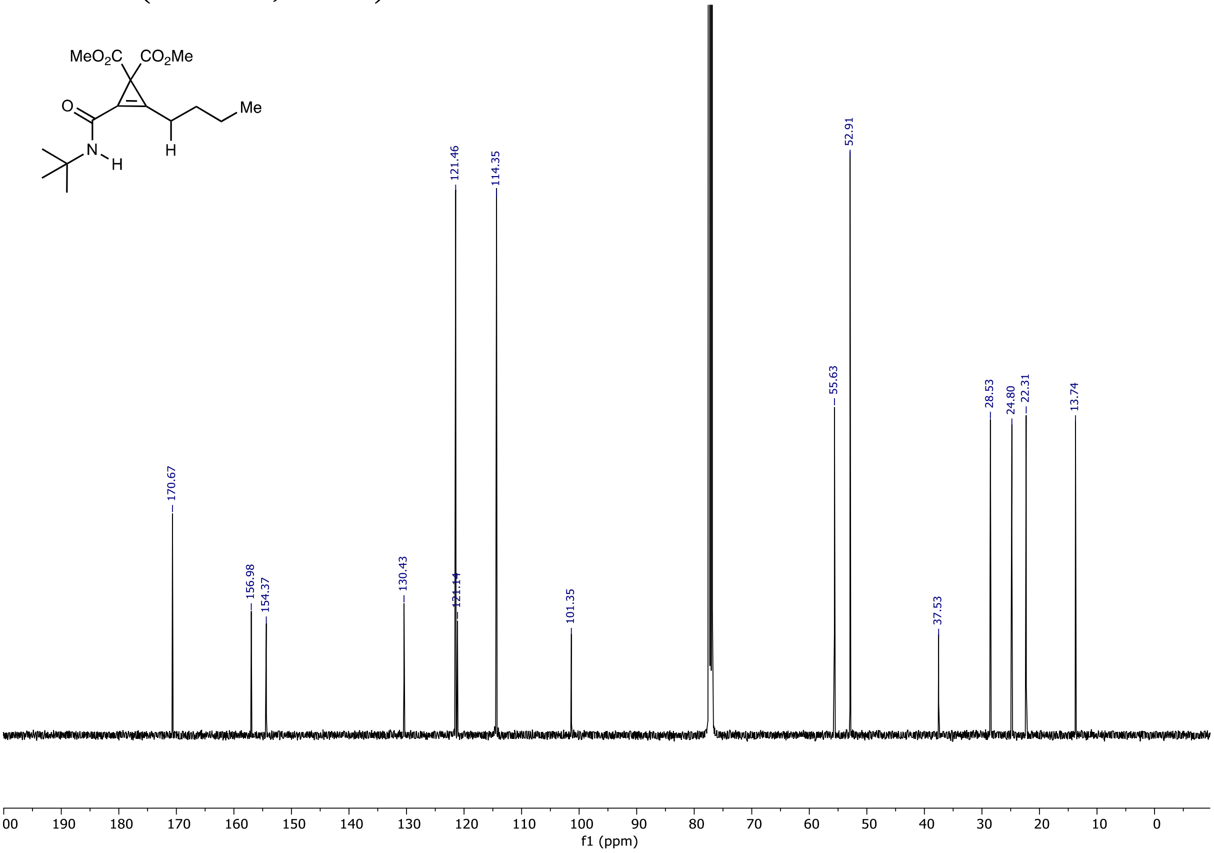

^{1}H NMR (600 MHz, $CDCl_3$) of **30a**

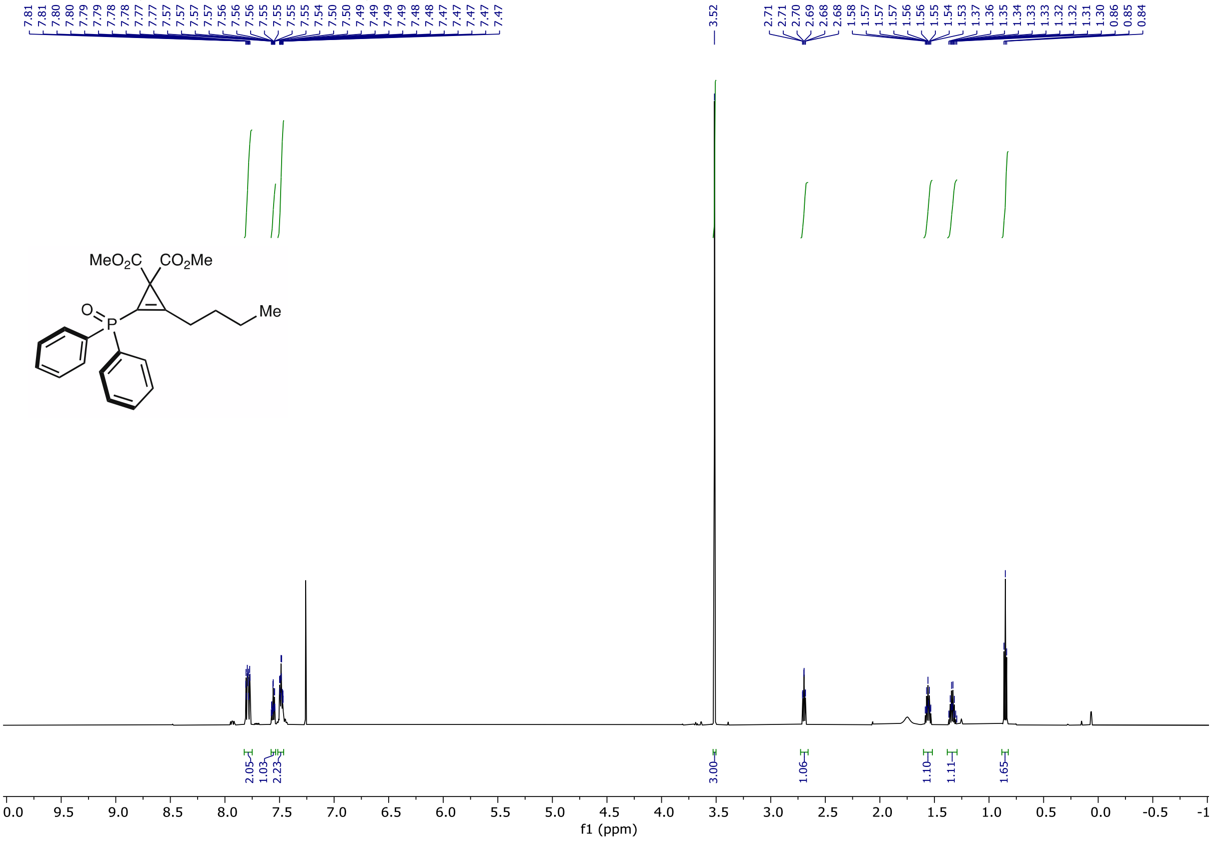

^{13}C NMR (151 MHz, $CDCl_3$) of **30a**

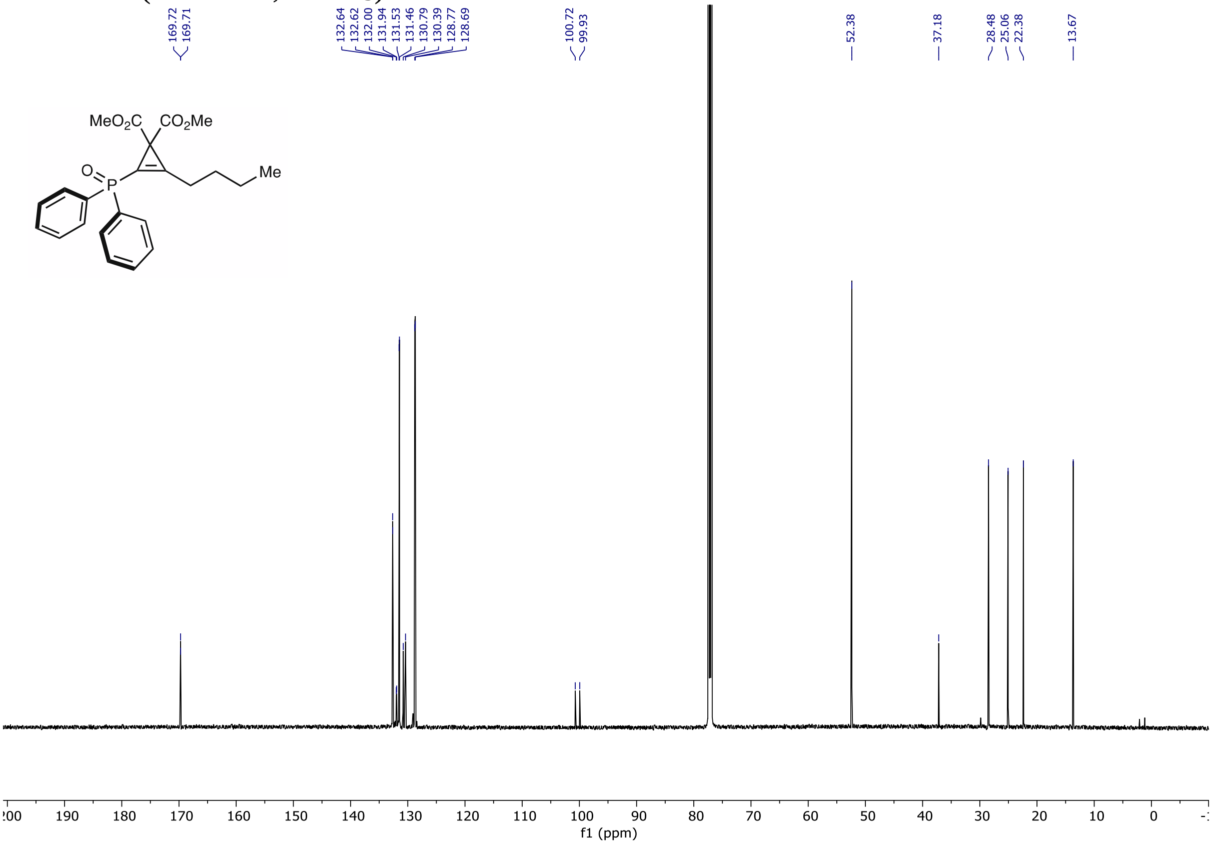

^{31}P NMR (162 MHz, $CDCl_3$) of **30a**

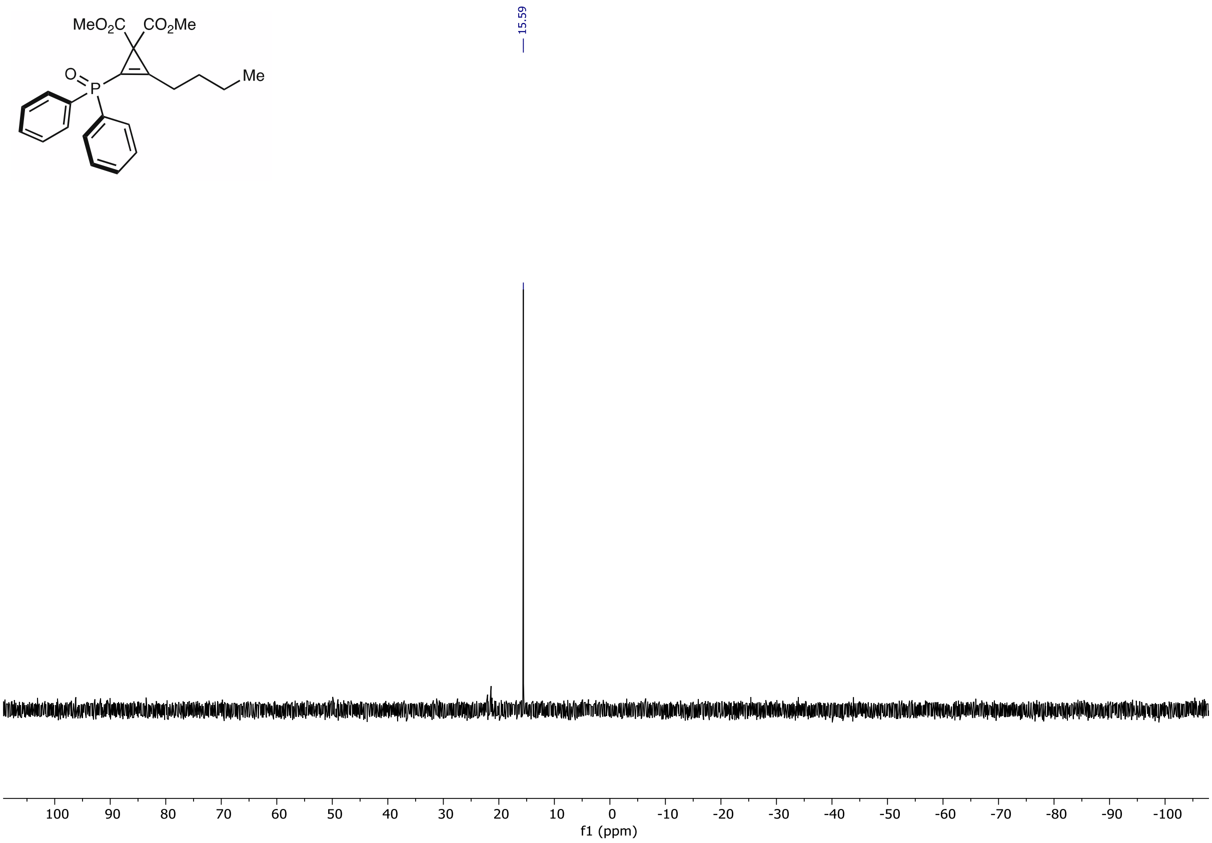

^{1}H NMR (600 MHz, $CDCl_3$) of **S27**

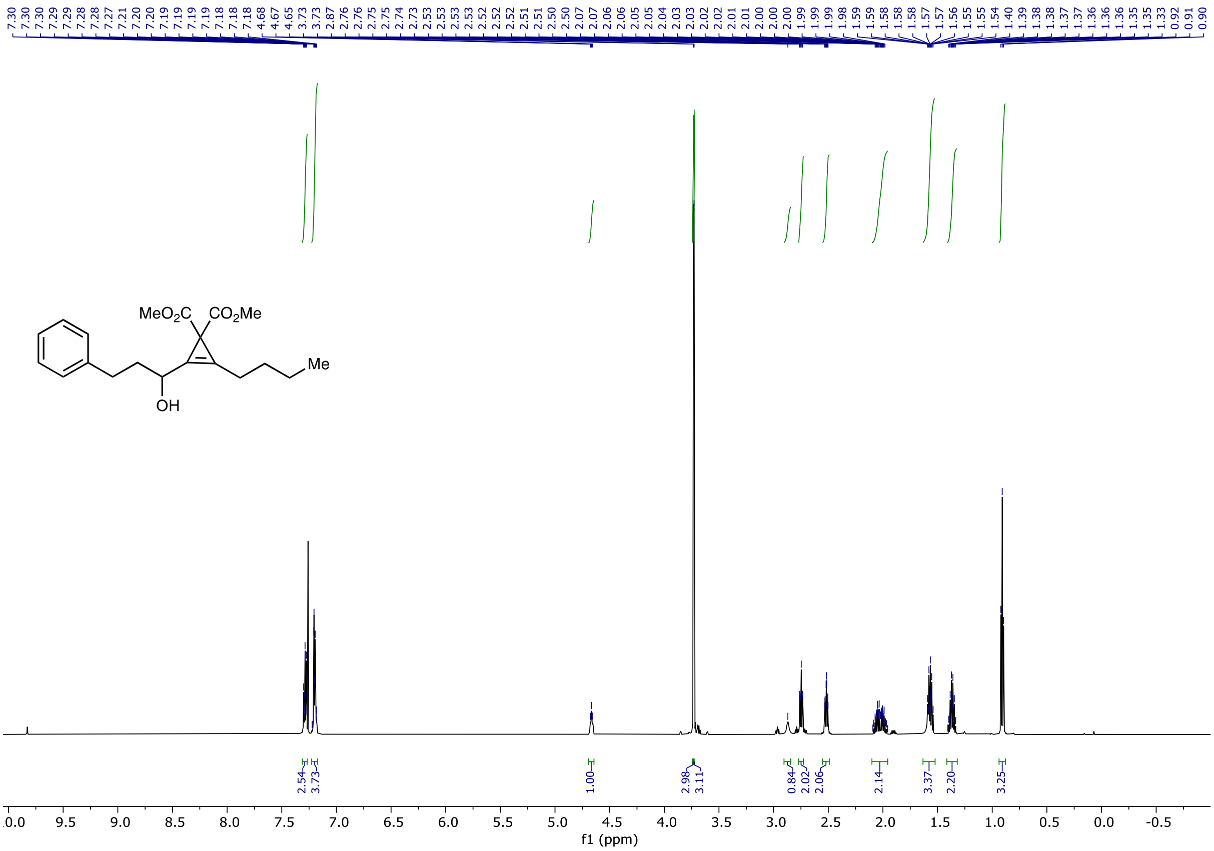

^{13}C NMR (151 MHz, $CDCl_3$) of **S27**

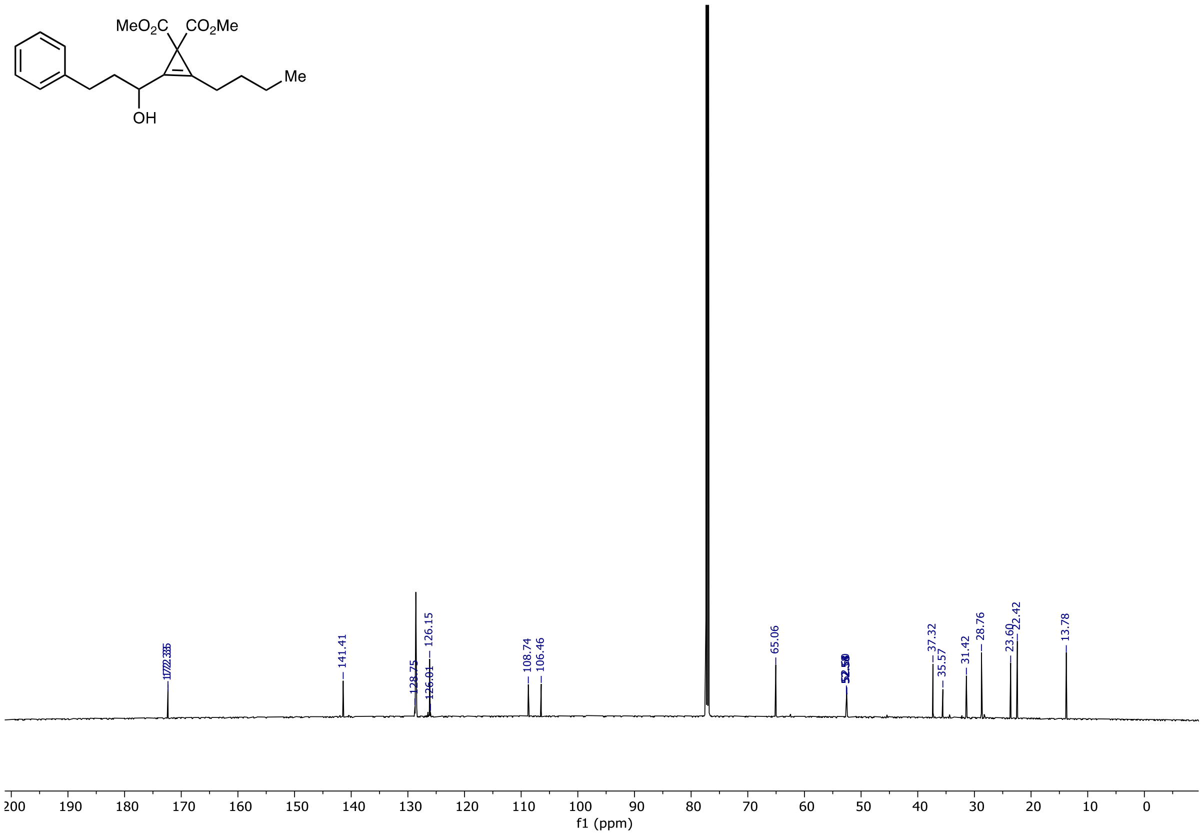

^{1}H NMR (400 MHz, $CDCl_3$) of **4a**

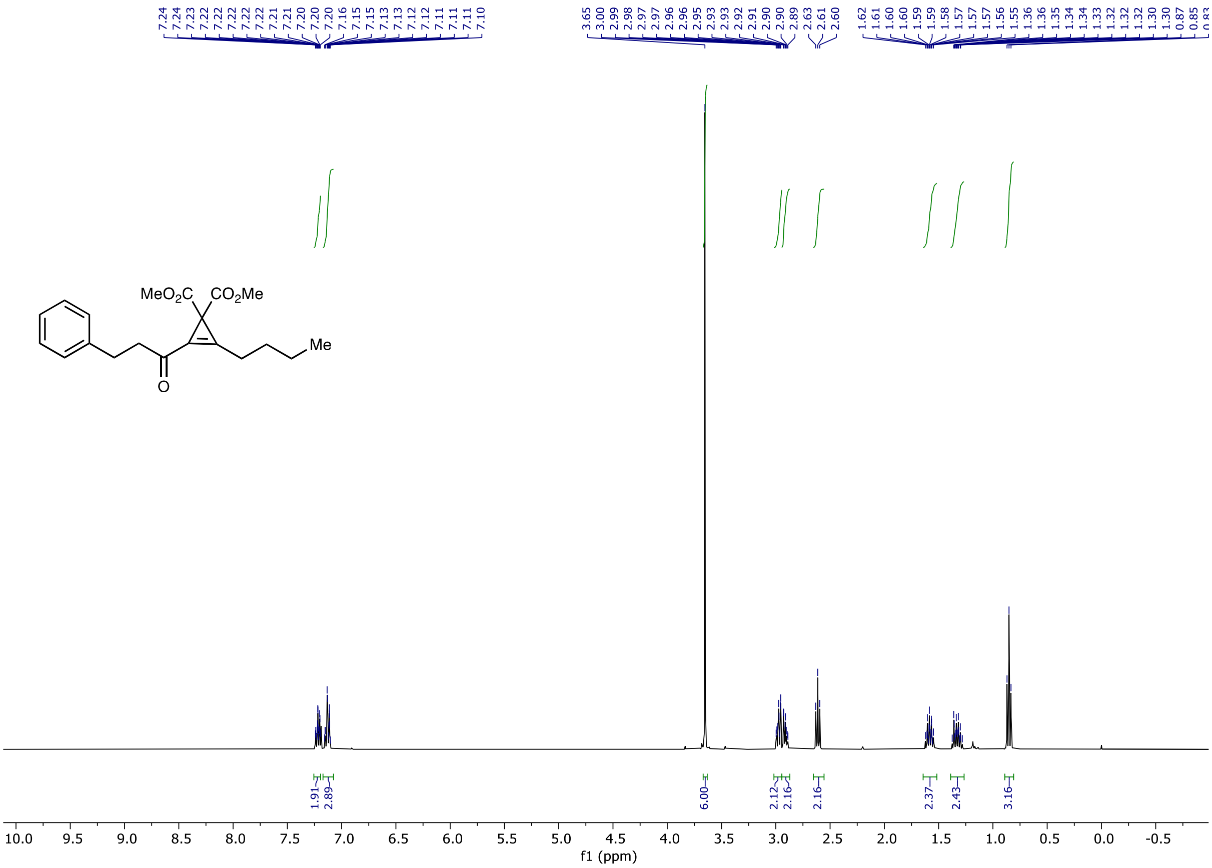

^{13}C NMR (101 MHz, $CDCl_3$) of **4a**

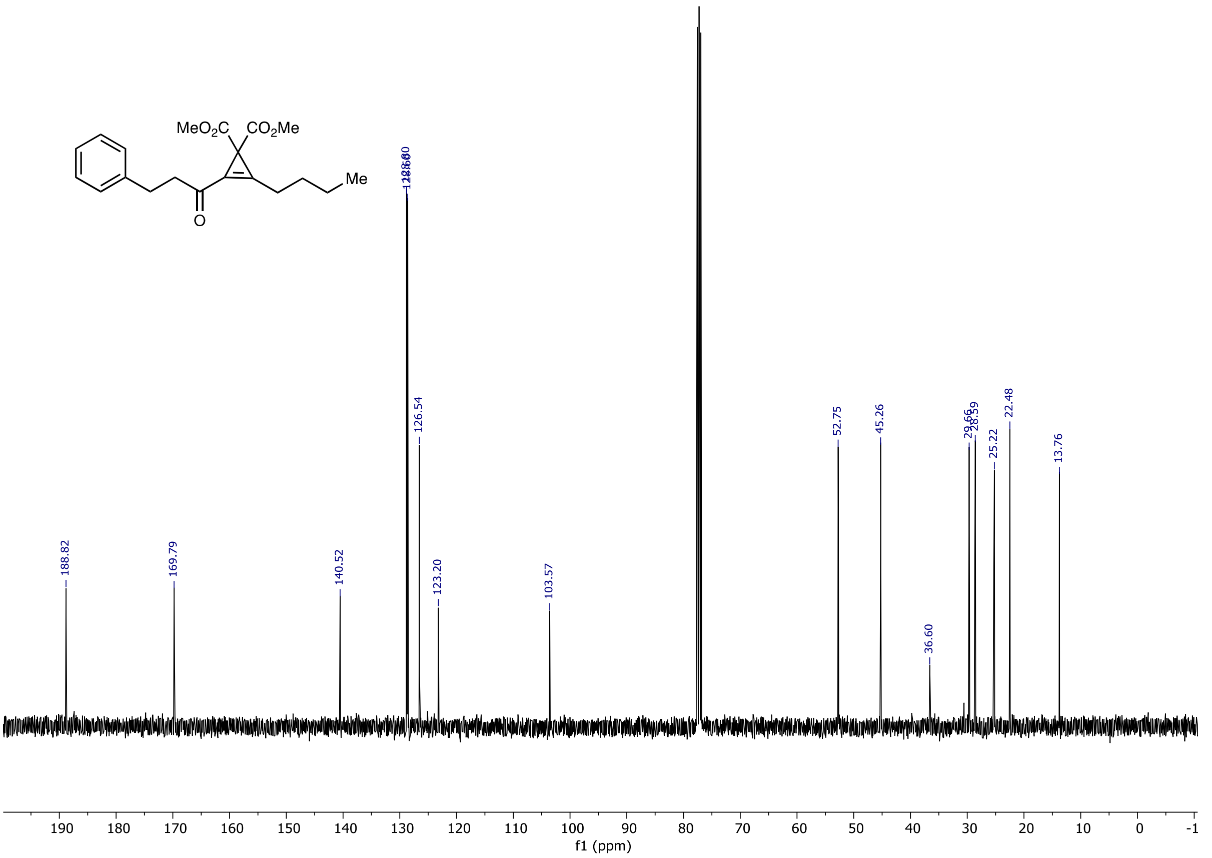

^{1}H NMR (500 MHz, $CDCl_3$) of **S28**

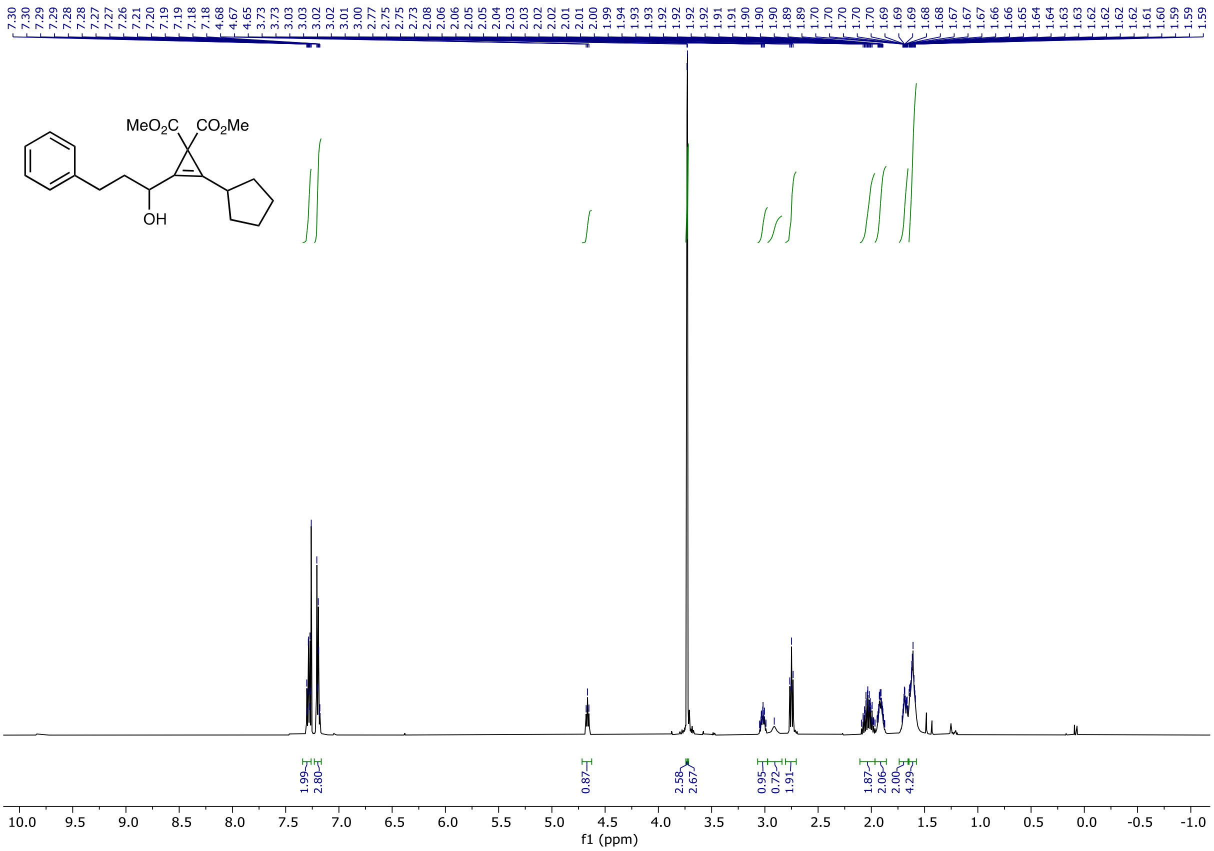

^{13}C NMR (126 MHz, $CDCl_3$) of **S28**

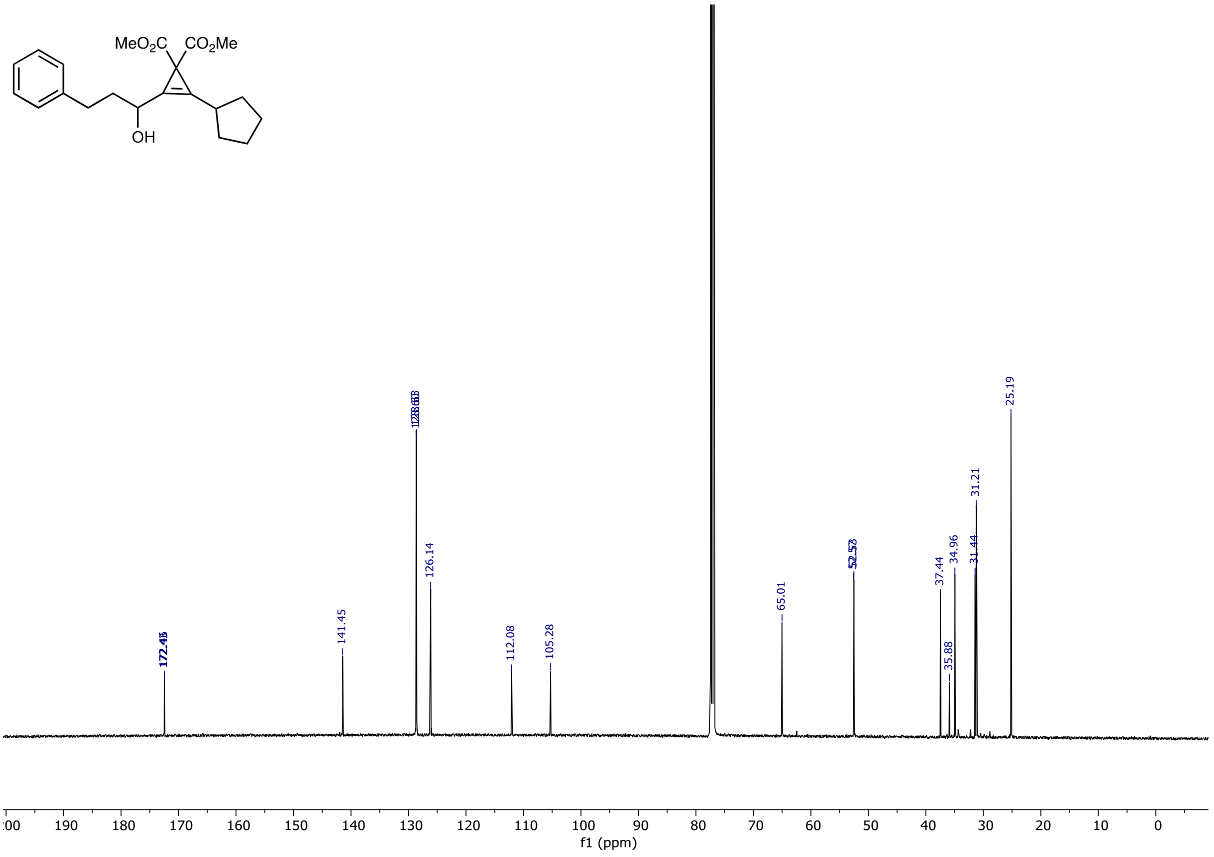

^{1}H NMR (500 MHz, $CDCl_3$) of **31a**

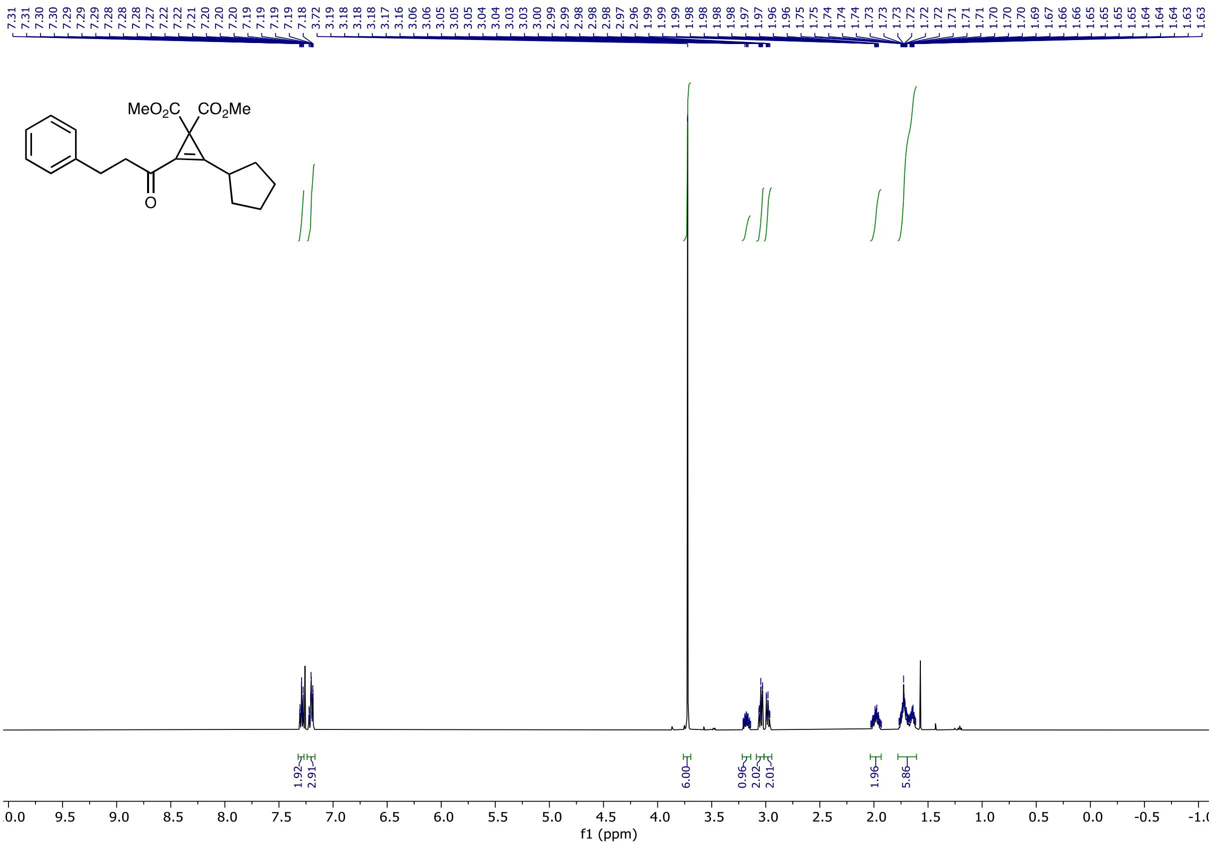

^{13}C NMR (126 MHz, $CDCl_3$) of **31a**

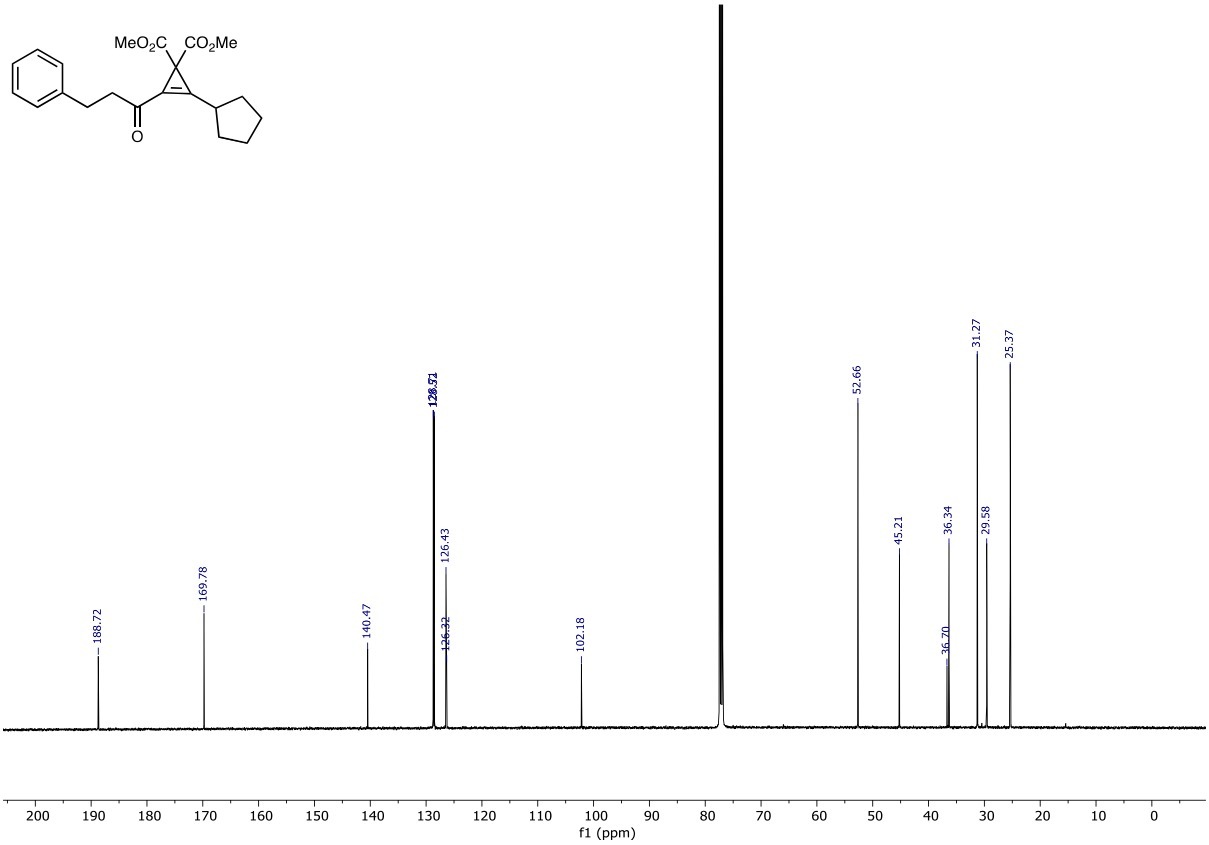

^{1}H NMR (500 MHz, $CDCl_3$) of **S29**

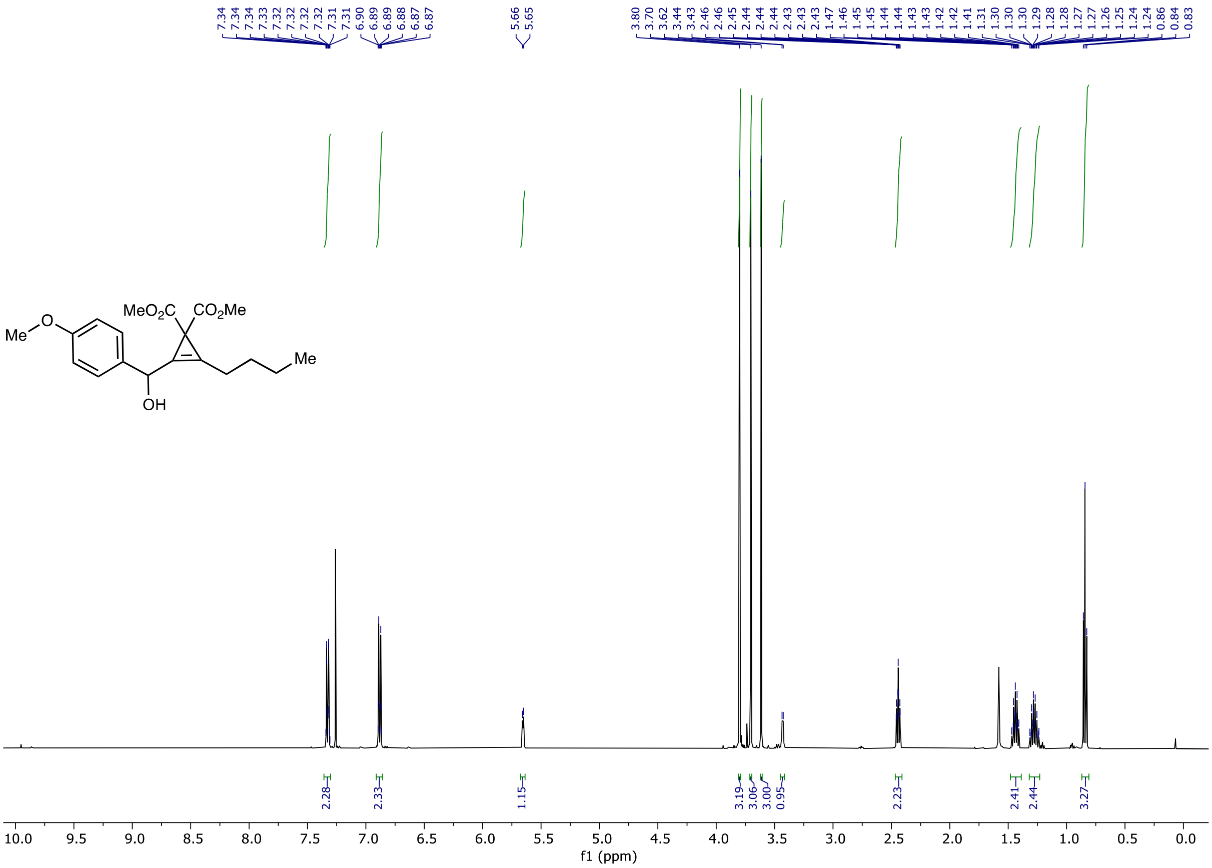

^{13}C NMR (126 MHz, $CDCl_3$) of **S29**

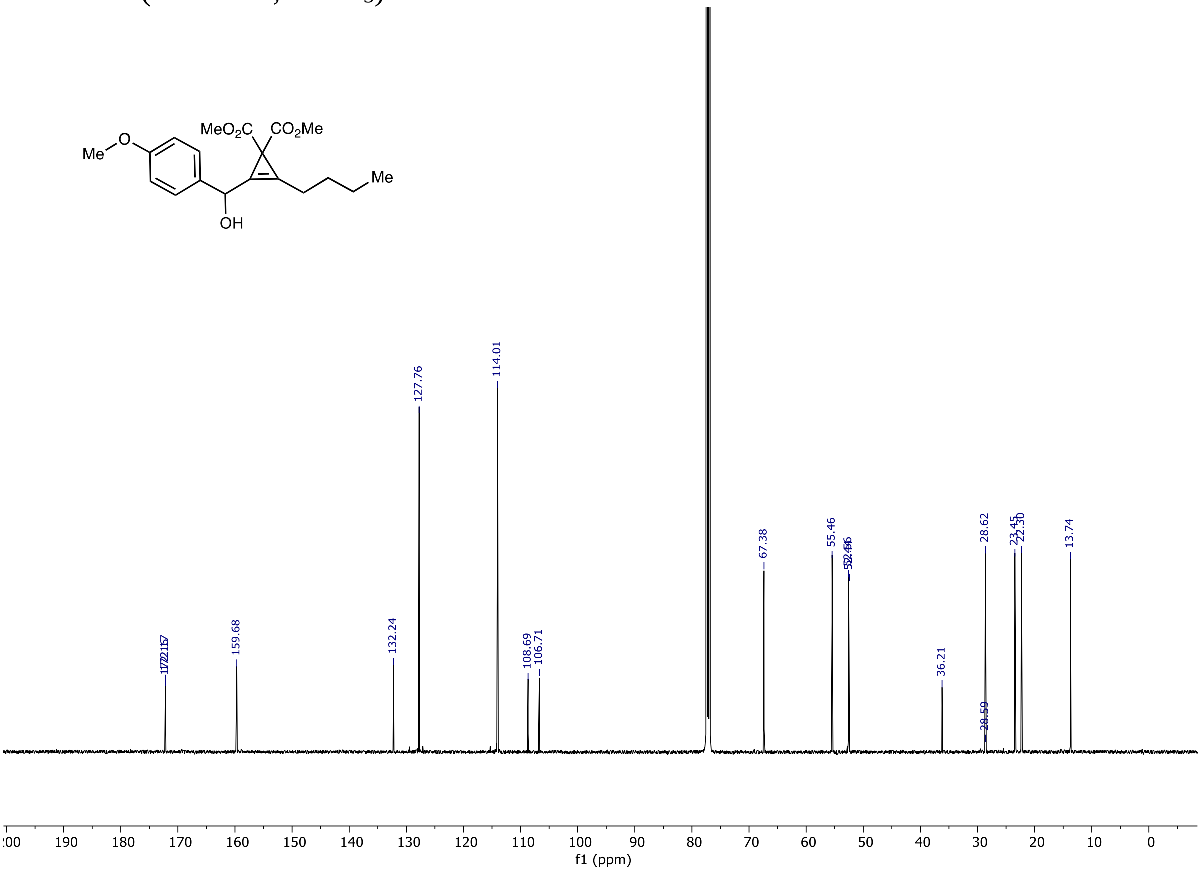

^{1}H NMR (500 MHz, $CDCl_3$) of **32a**

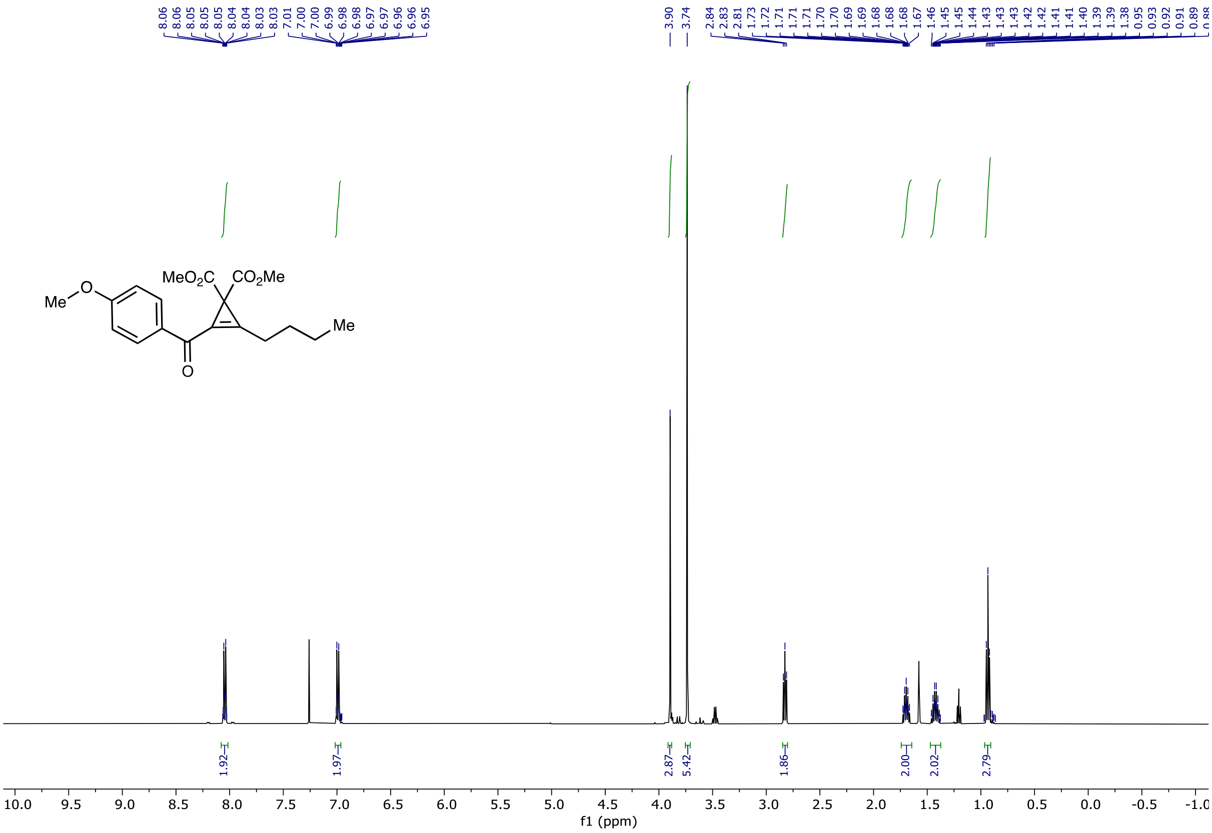

^{13}C NMR (126 MHz, $CDCl_3$) of **32a**

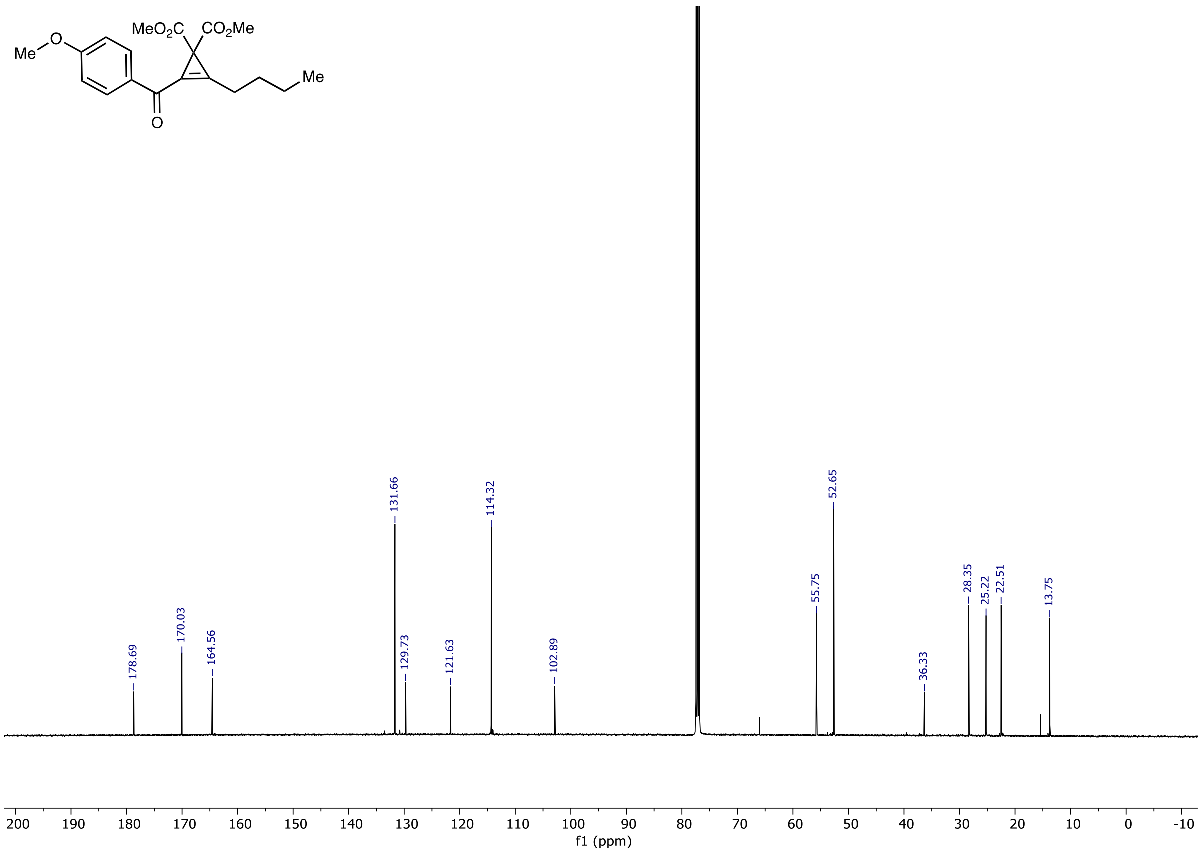

^{1}H NMR (400 MHz, $CDCl_3$) of **S31**

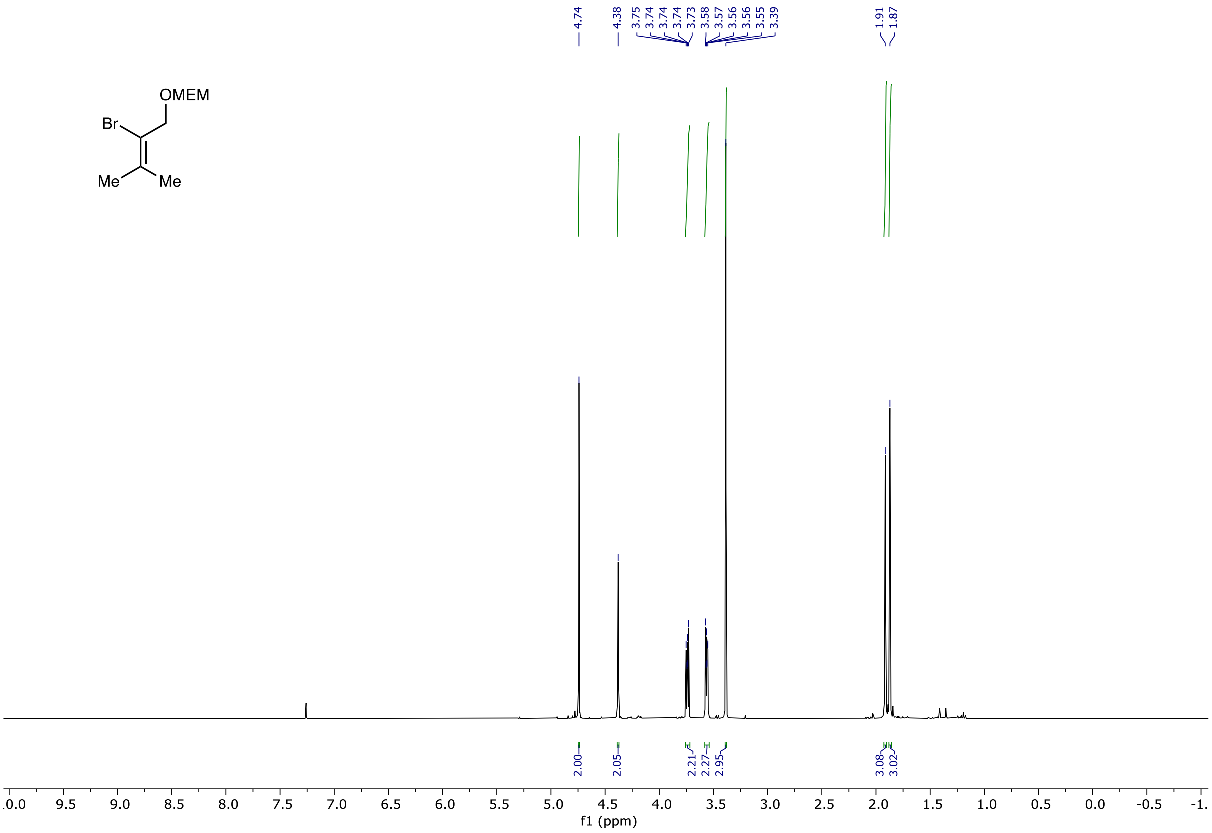

^{13}C NMR (101 MHz, $CDCl_3$) of **S31**

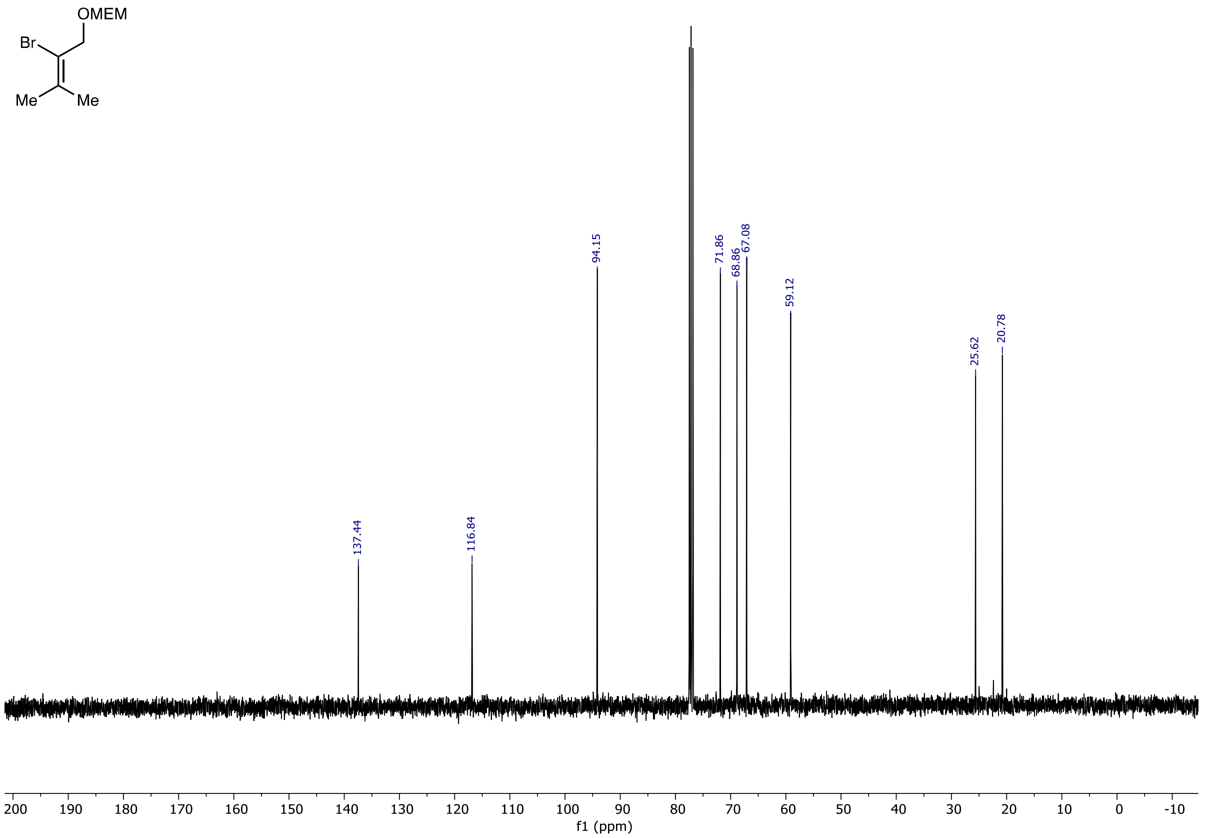

^{1}**H NMR** (500 MHz, $CDCl_3$) of **S32**

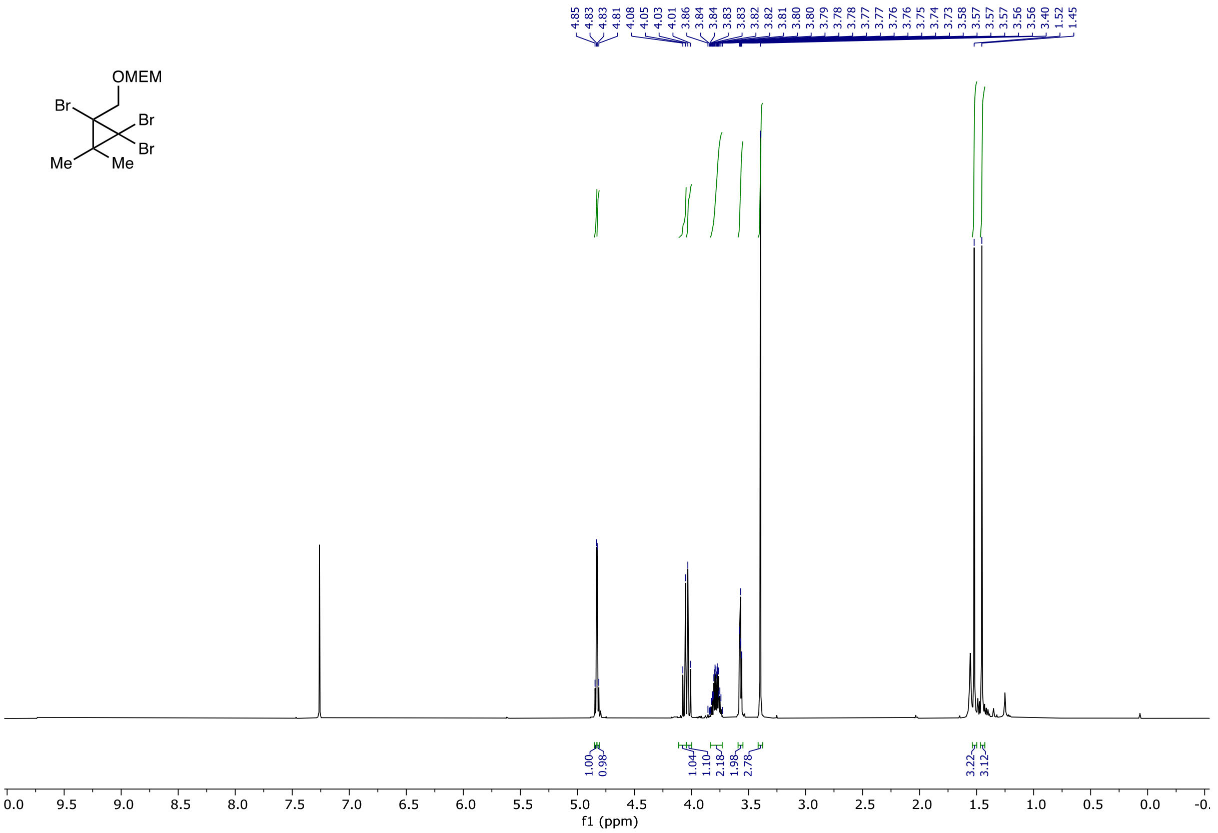

^{13}**C NMR** (126 MHz, $CDCl_3$) of **S32**

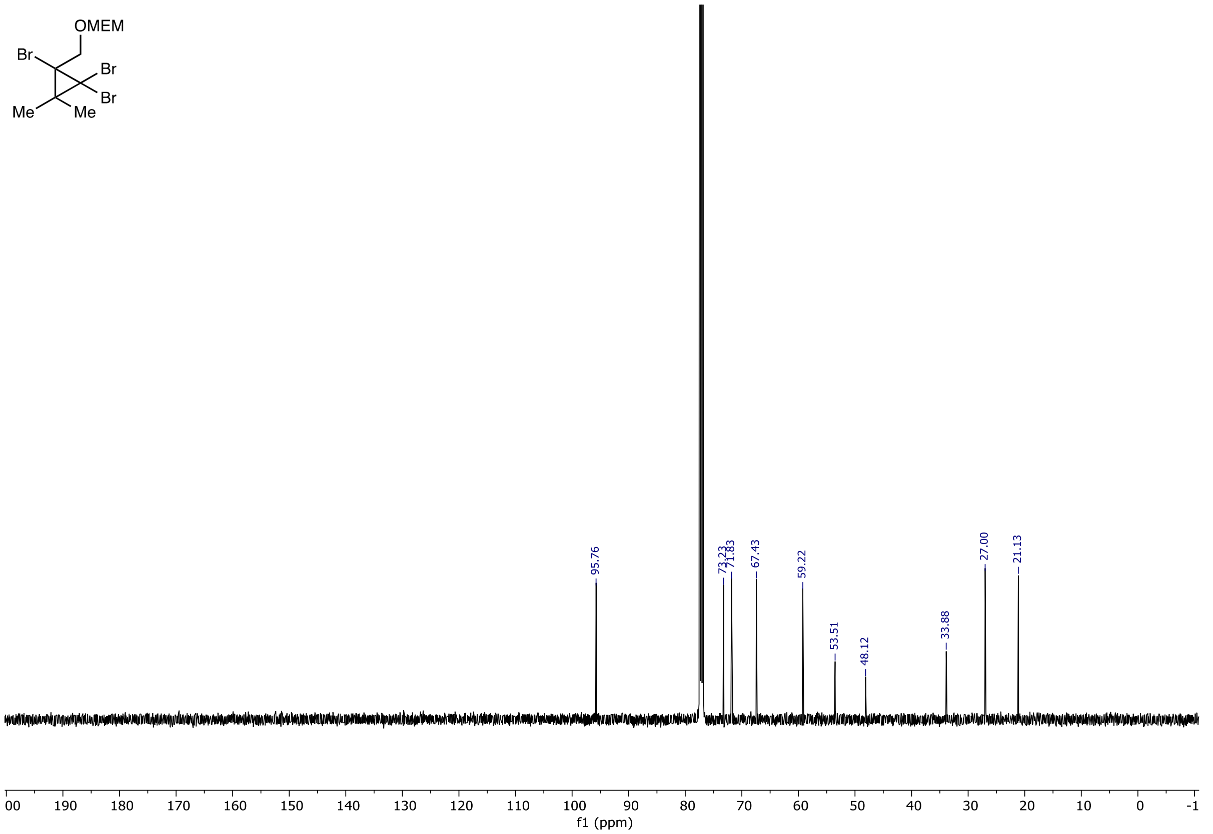

^{1}H NMR (500 MHz, $CDCl_3$) of **33a**

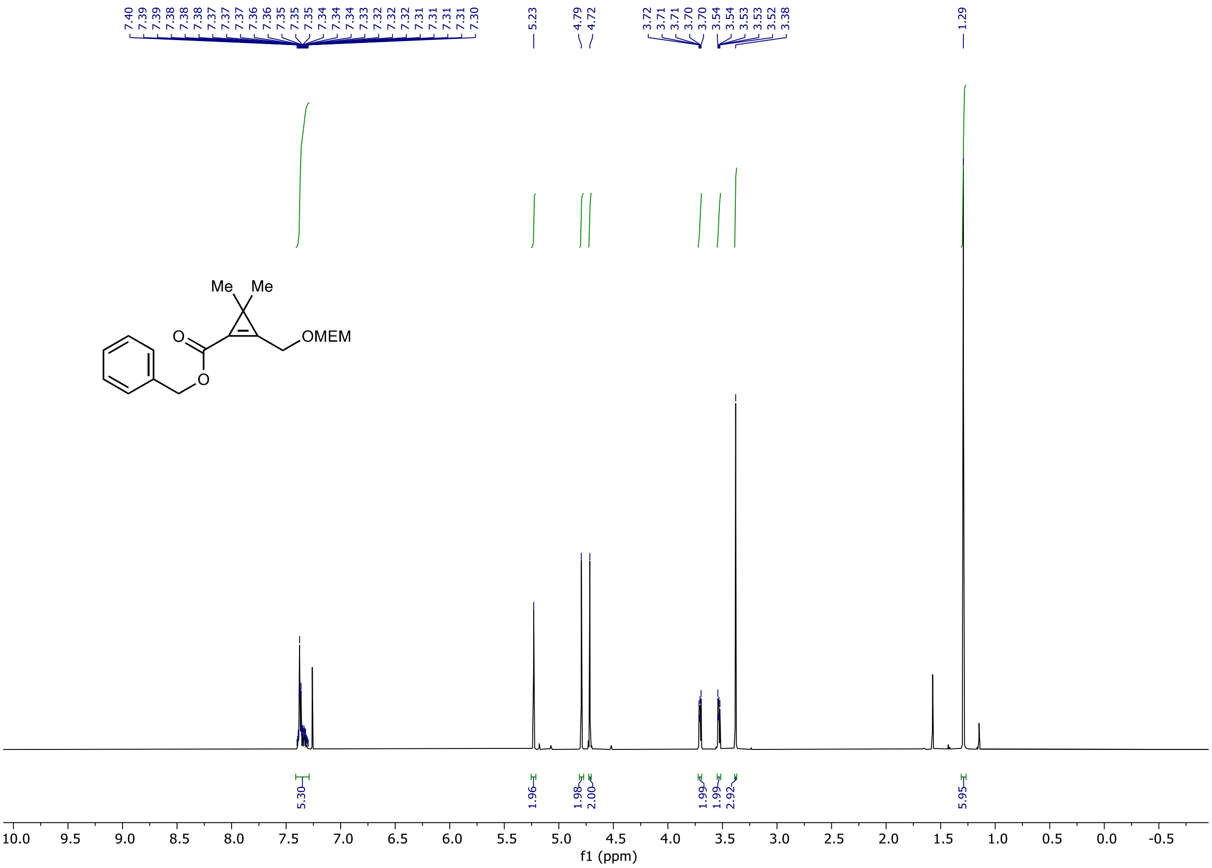

^{13}C NMR (126 MHz, $CDCl_3$) of **33a**

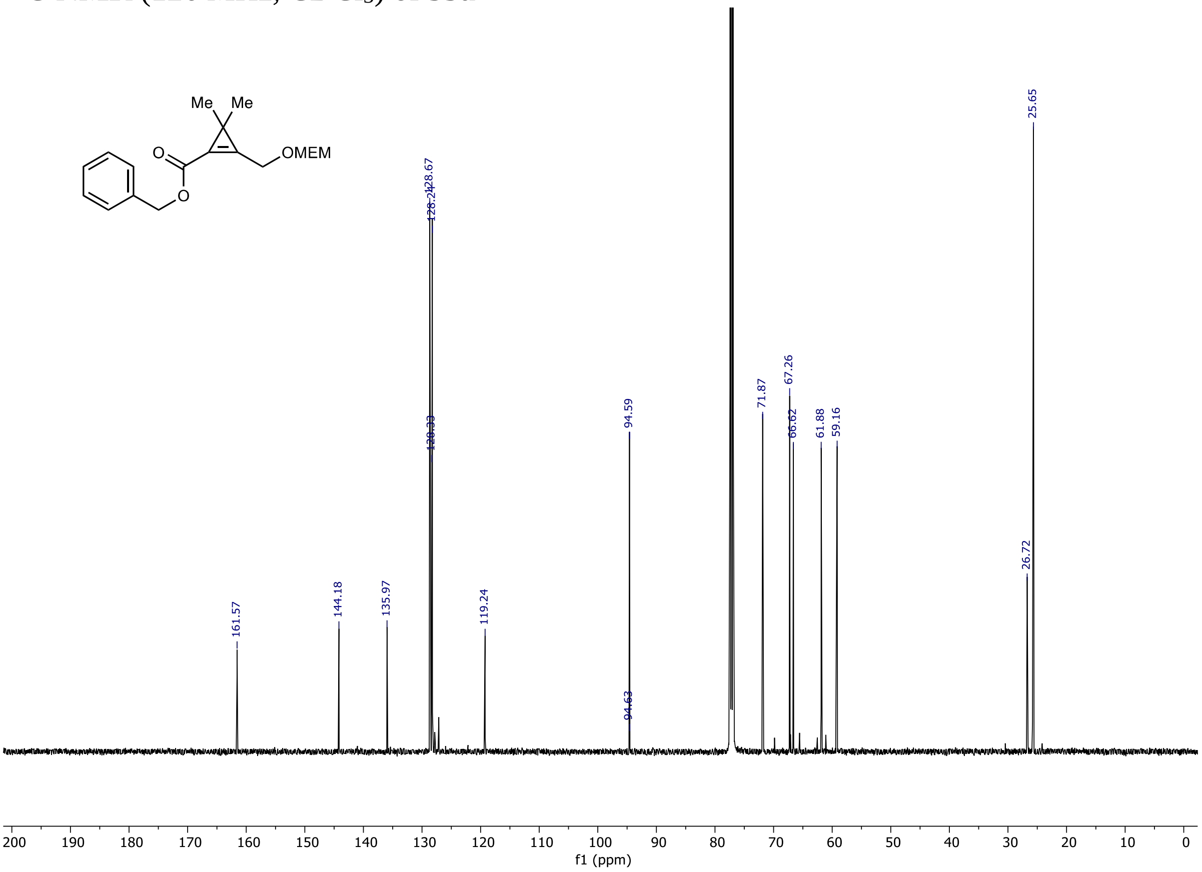

^{1}H NMR (500 MHz, $CDCl_3$) of **34a**

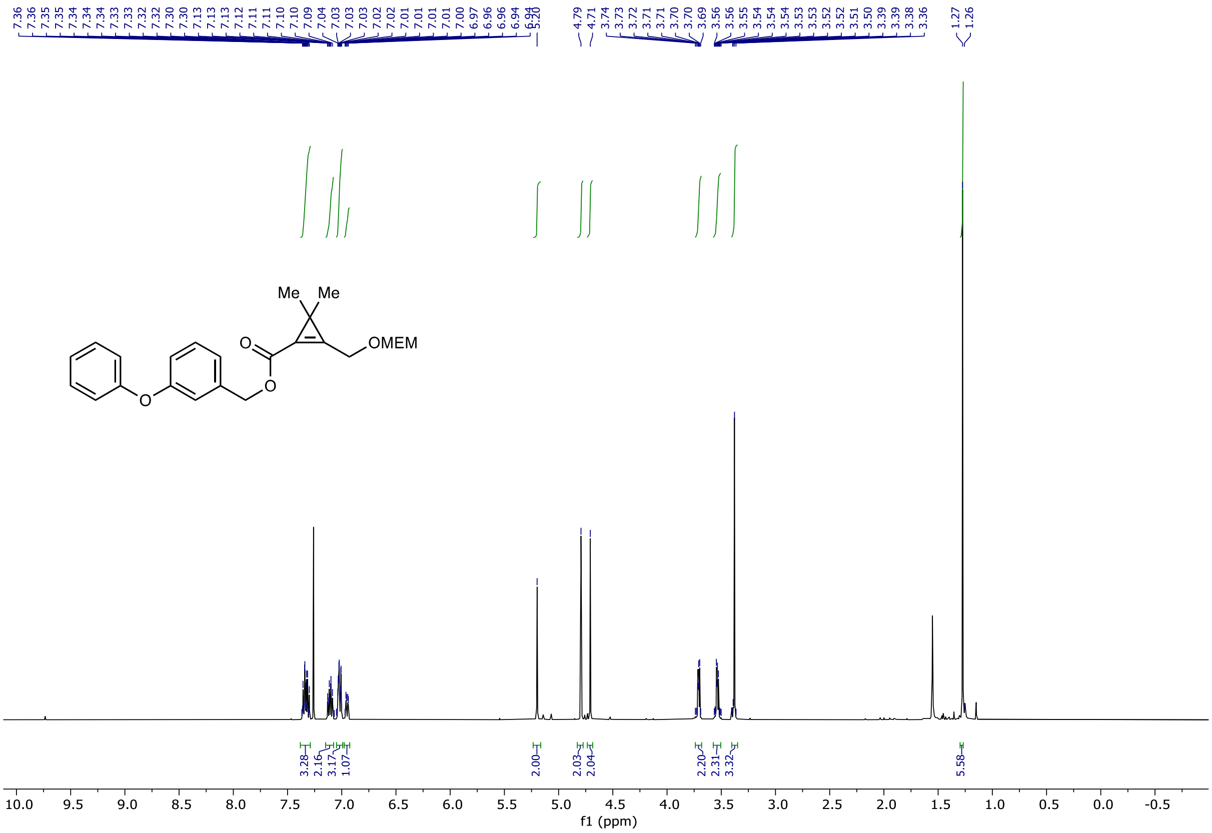

^{13}C NMR (126 MHz, $CDCl_3$) of **34a**

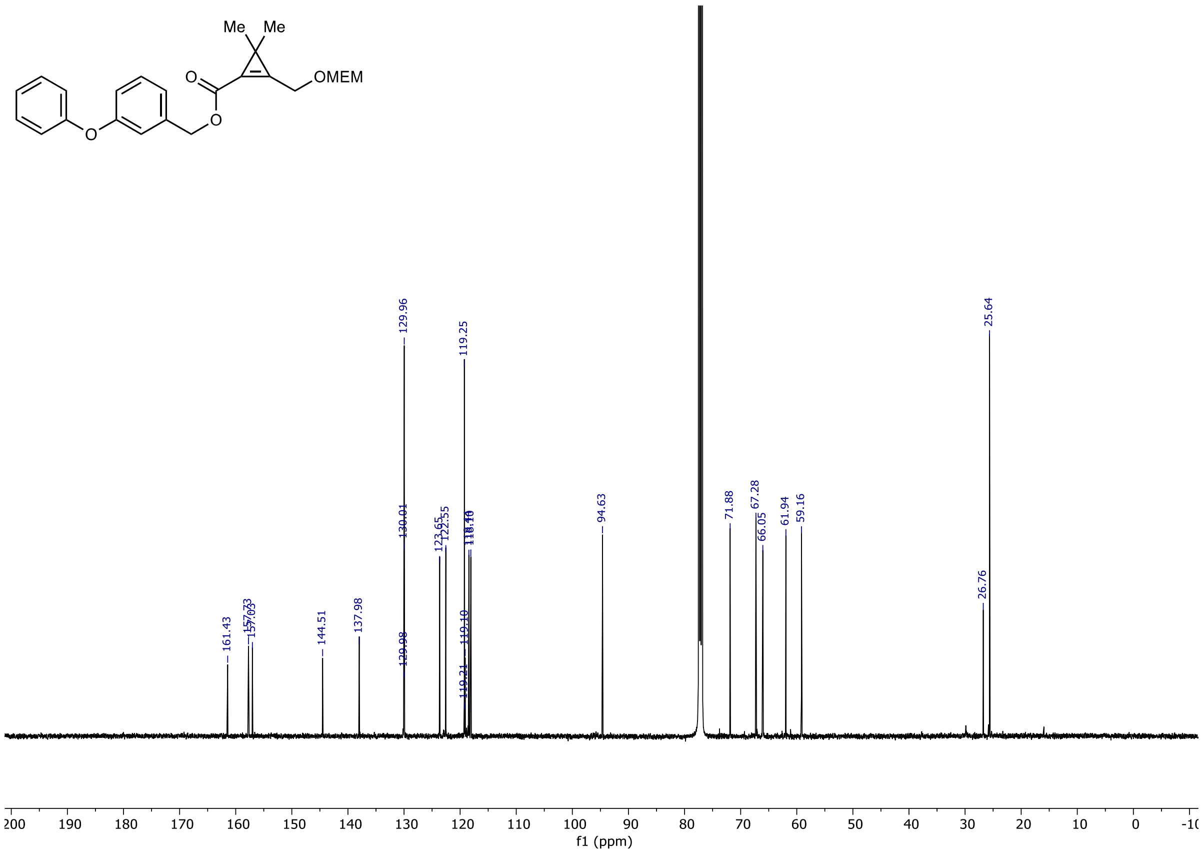

^{1}H NMR (400 MHz, $CDCl_3$) of **S34**

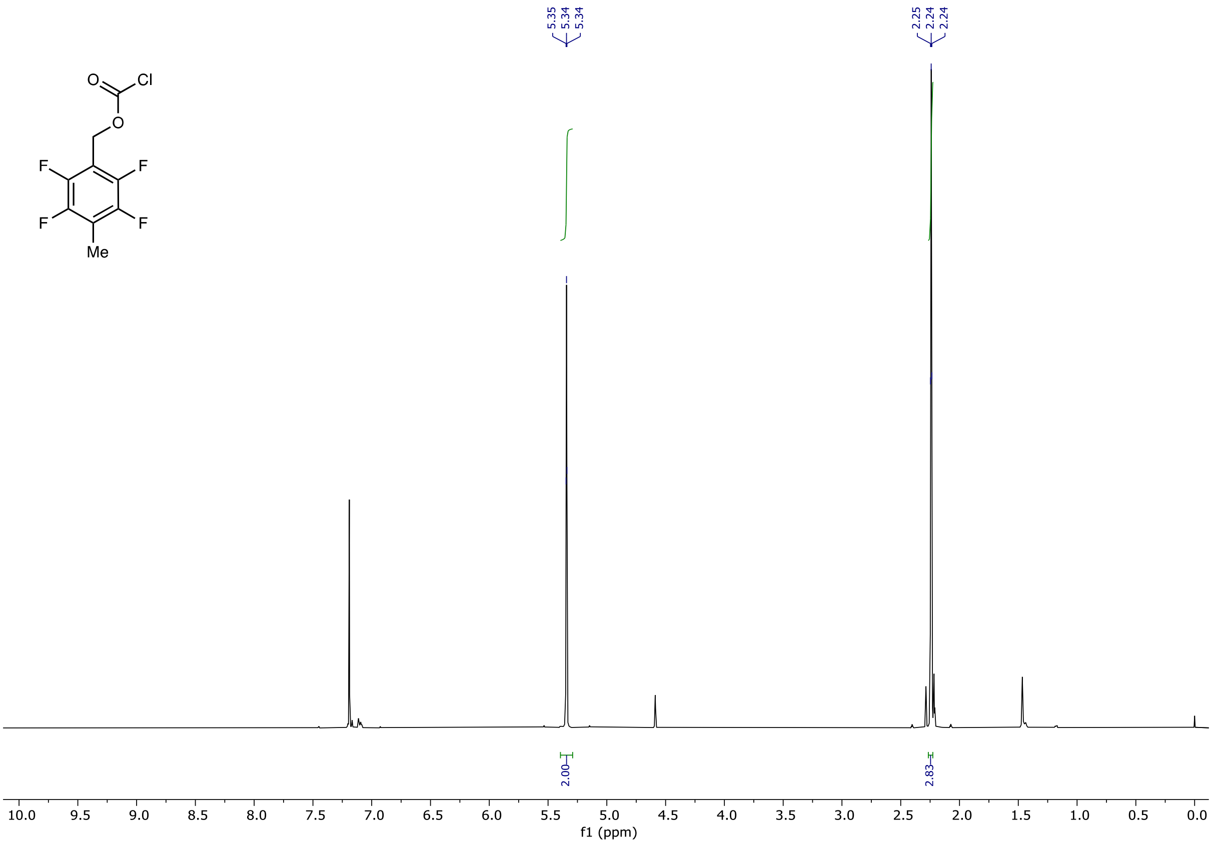

^{1}H NMR (500 MHz, $CDCl_3$) of **35a**

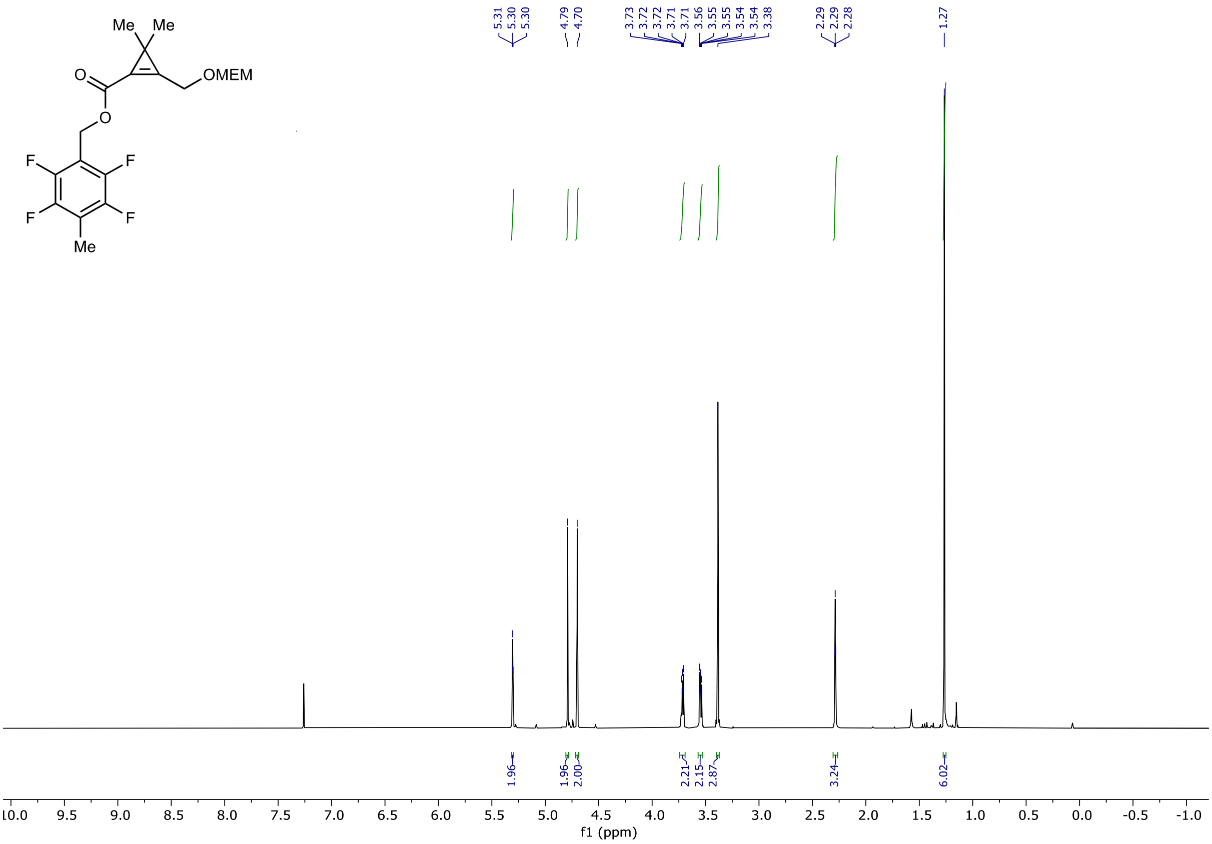

^{13}C NMR (126 MHz, $CDCl_3$) of **35a**

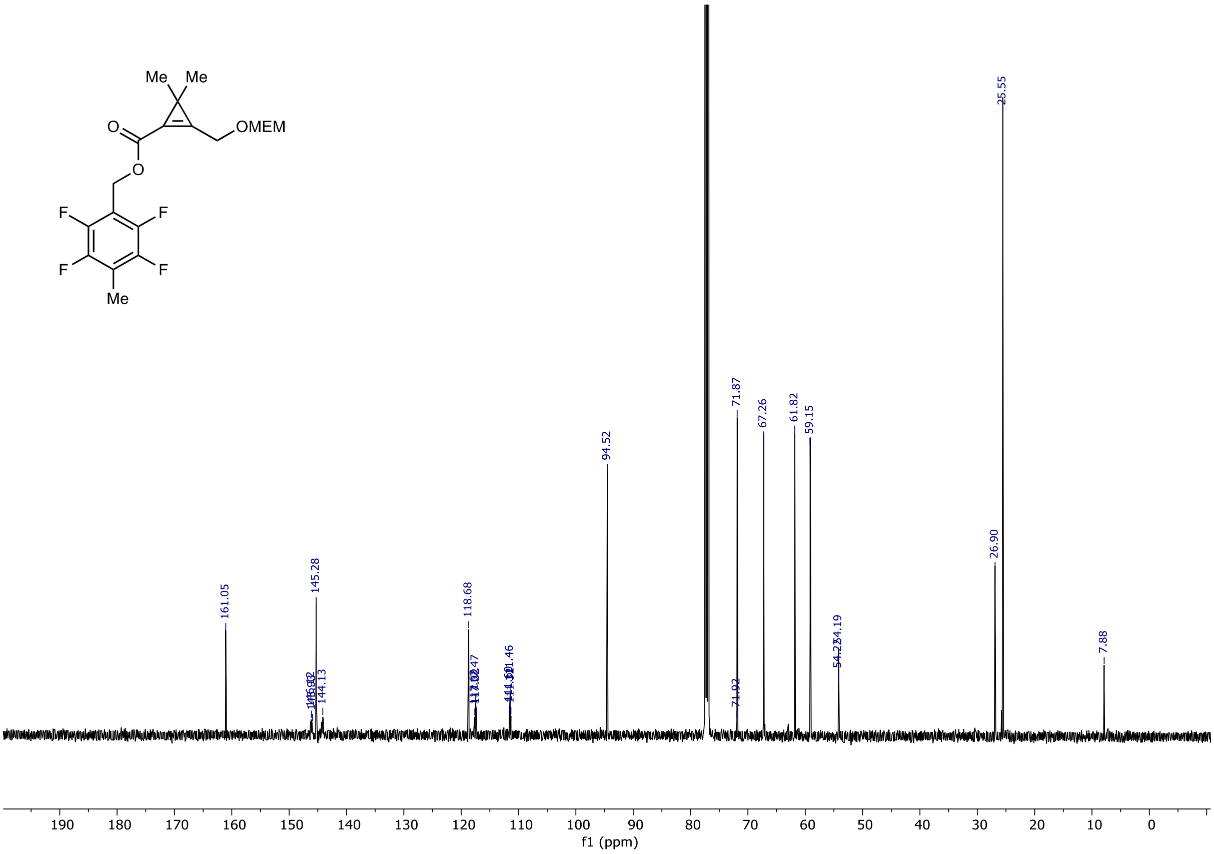

^{19}F NMR (471 MHz, $CDCl_3$) of **35a**

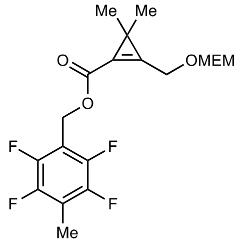

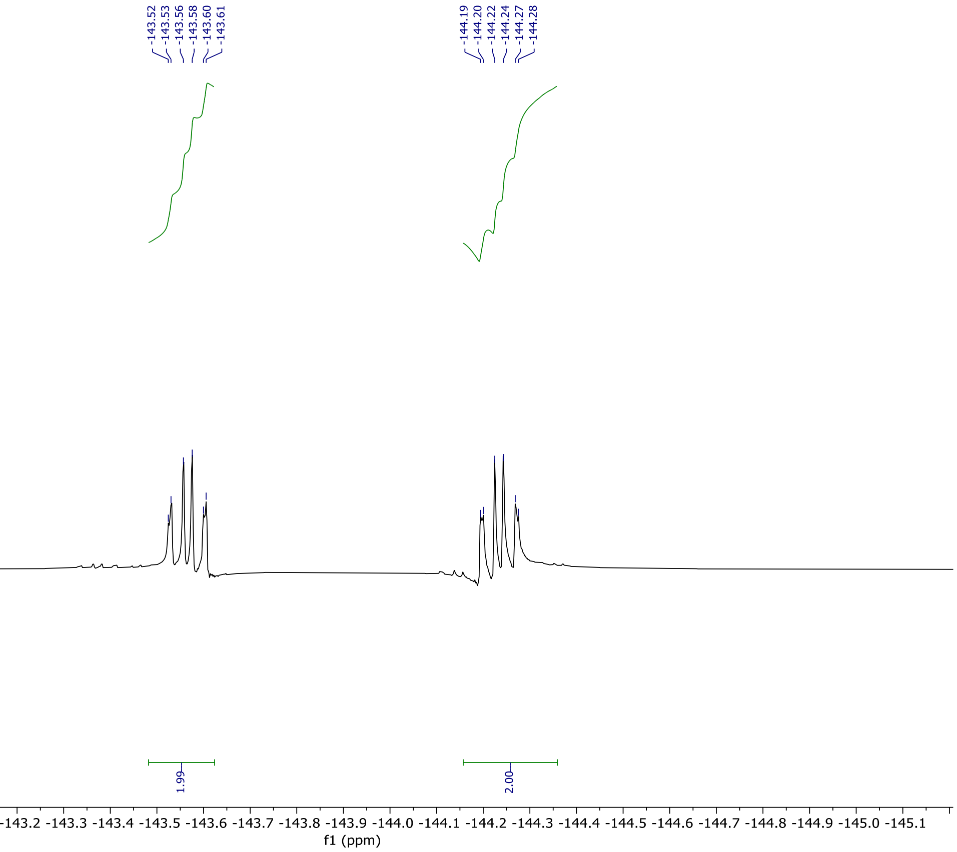

^{1}H NMR (400 MHz, $CDCl_3$) of **S35**

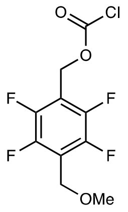

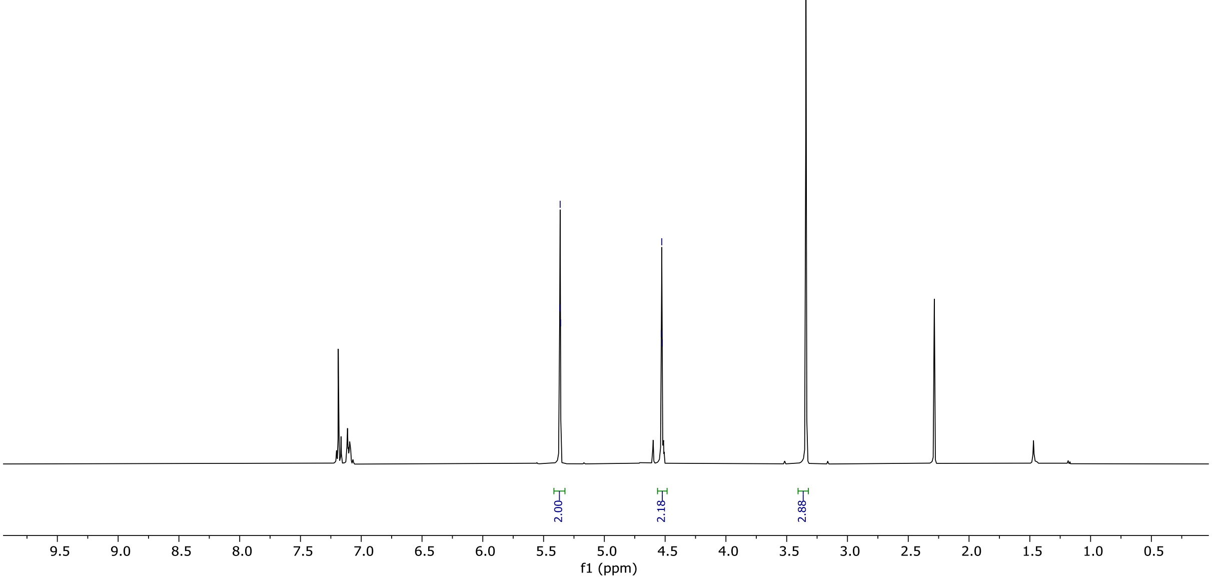

^{1}**H NMR** (500 MHz, $CDCl_3$) of **36a**

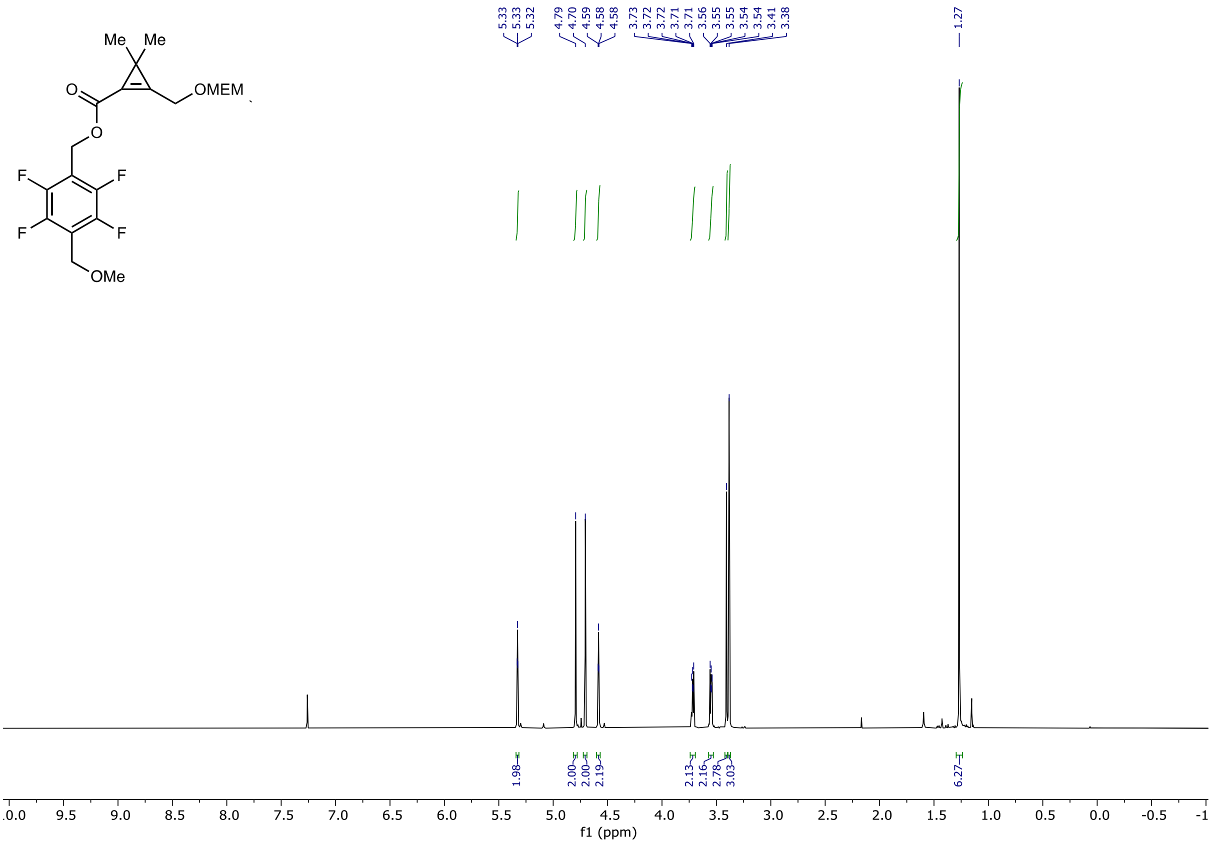

^{13}**C NMR** (126 MHz, $CDCl_3$) of **36a**

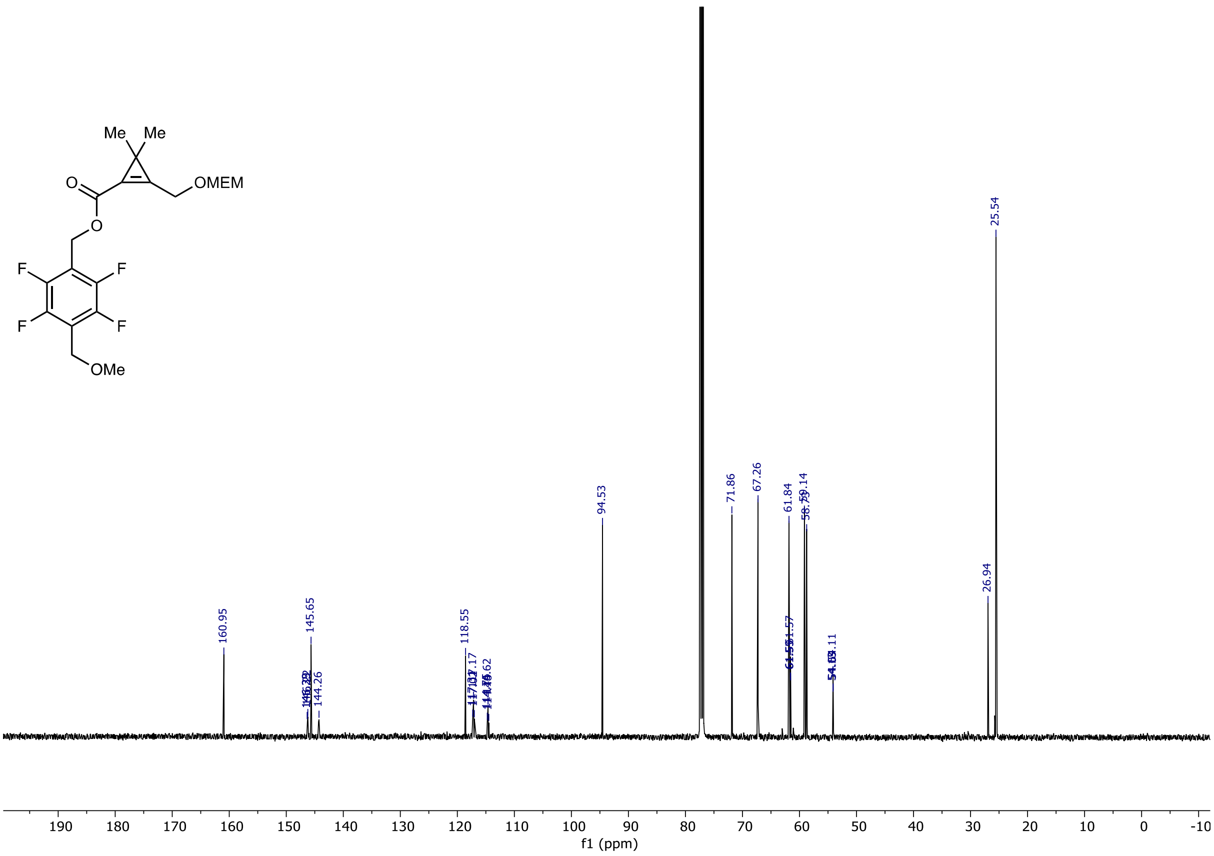

^{19}F NMR (471 MHz, $CDCl_3$) of **36a**

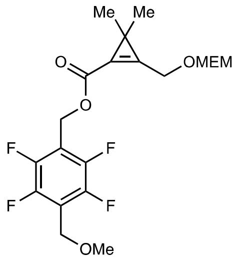

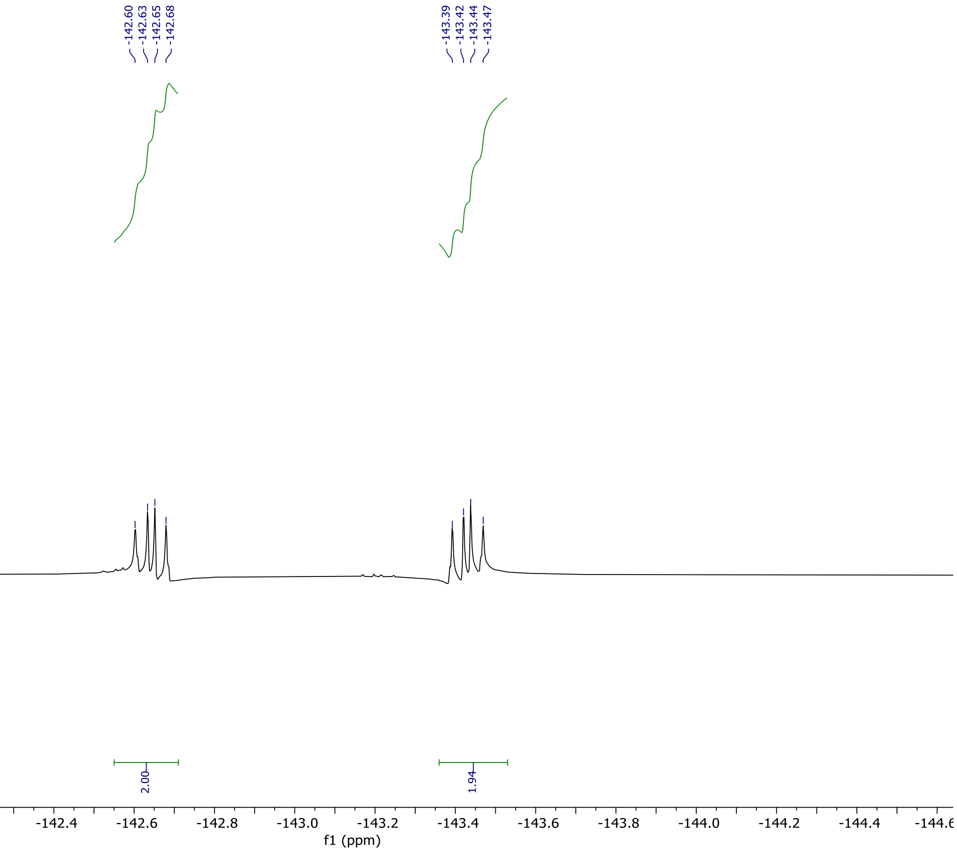

^{1}H NMR (400 MHz, $CDCl_3$) of **S36**

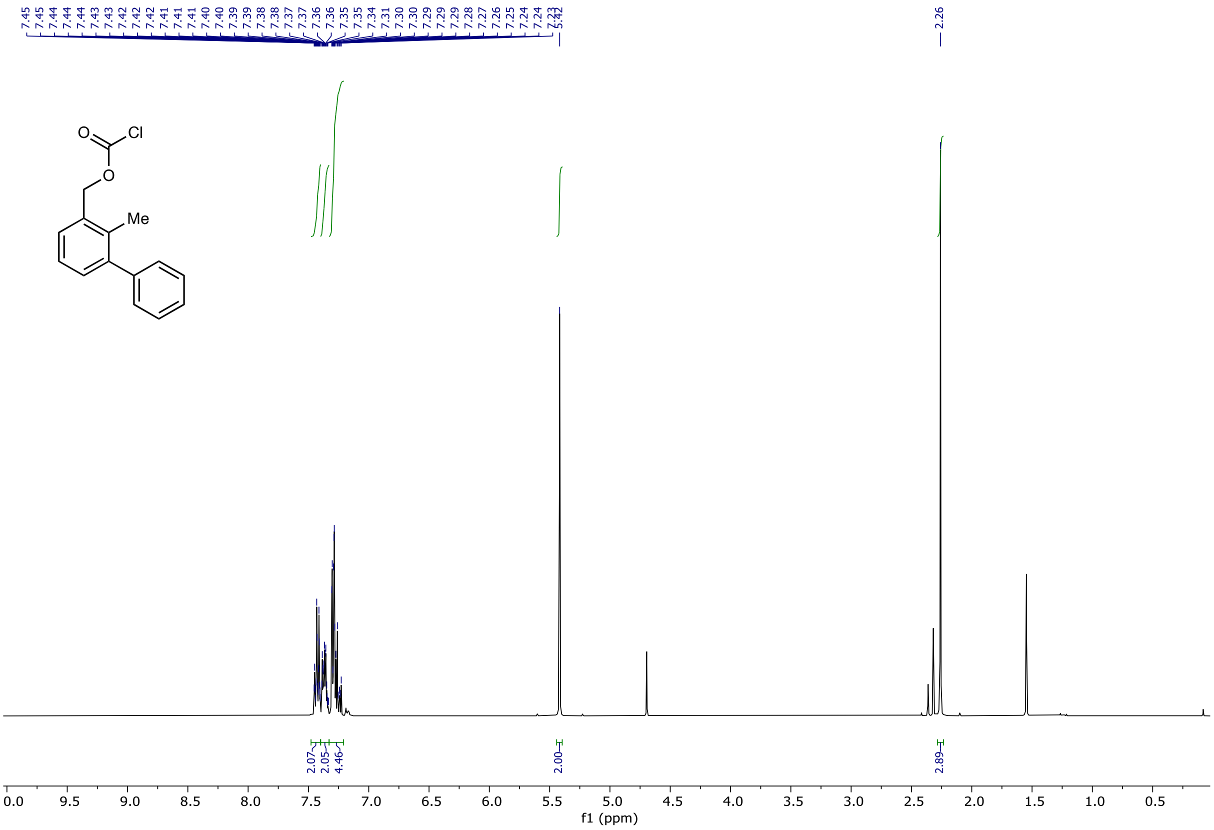

^{1}H NMR (500 MHz, $CDCl_3$) of **37a**

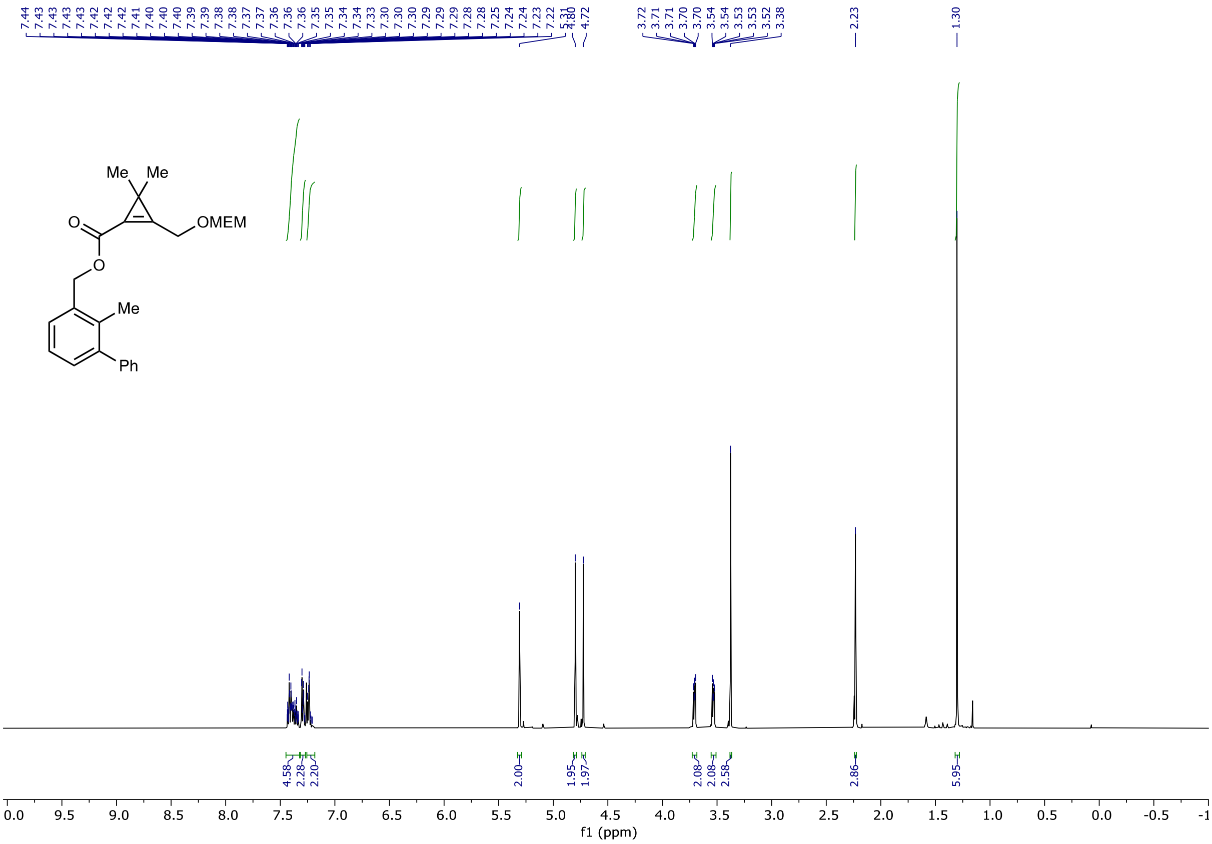

^{13}C NMR (126 MHz, $CDCl_3$) of **37a**

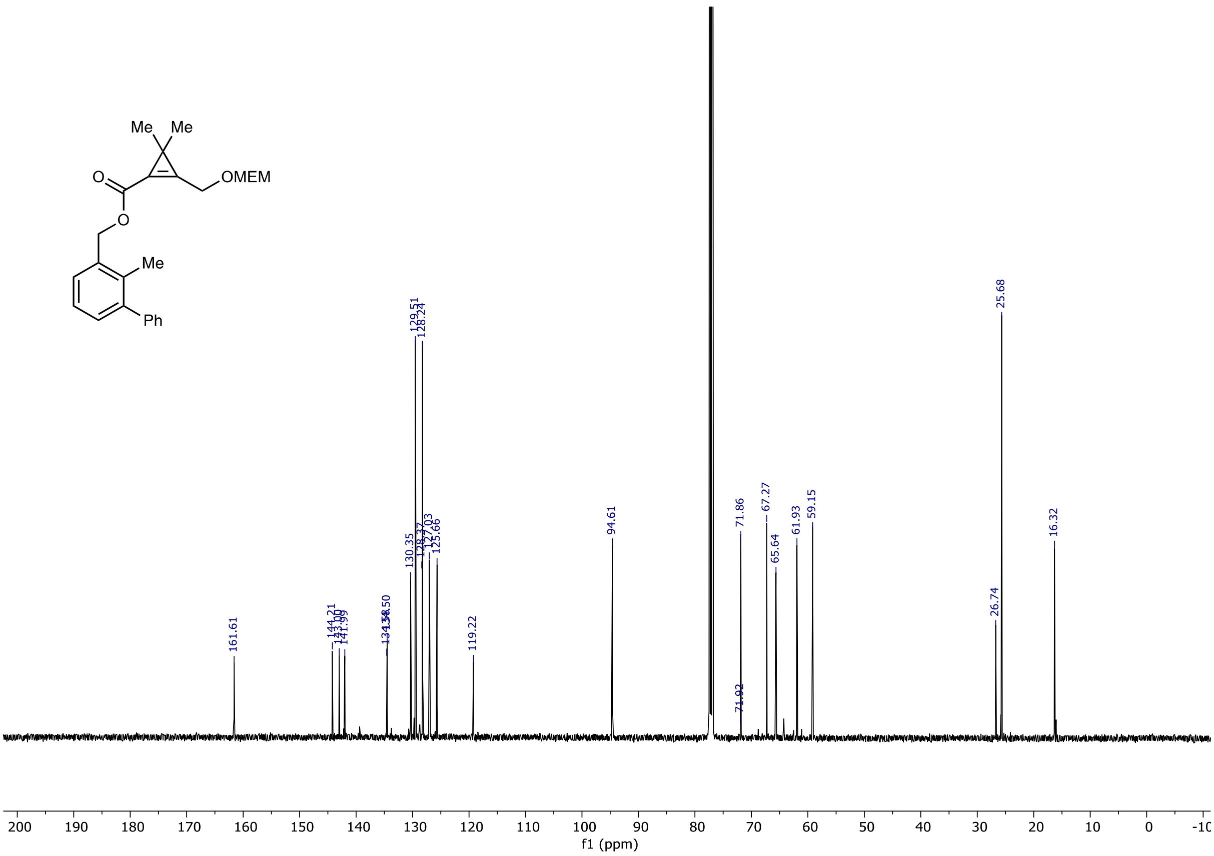

[1]**H NMR** (400 MHz, $CDCl_3$) of **39a**

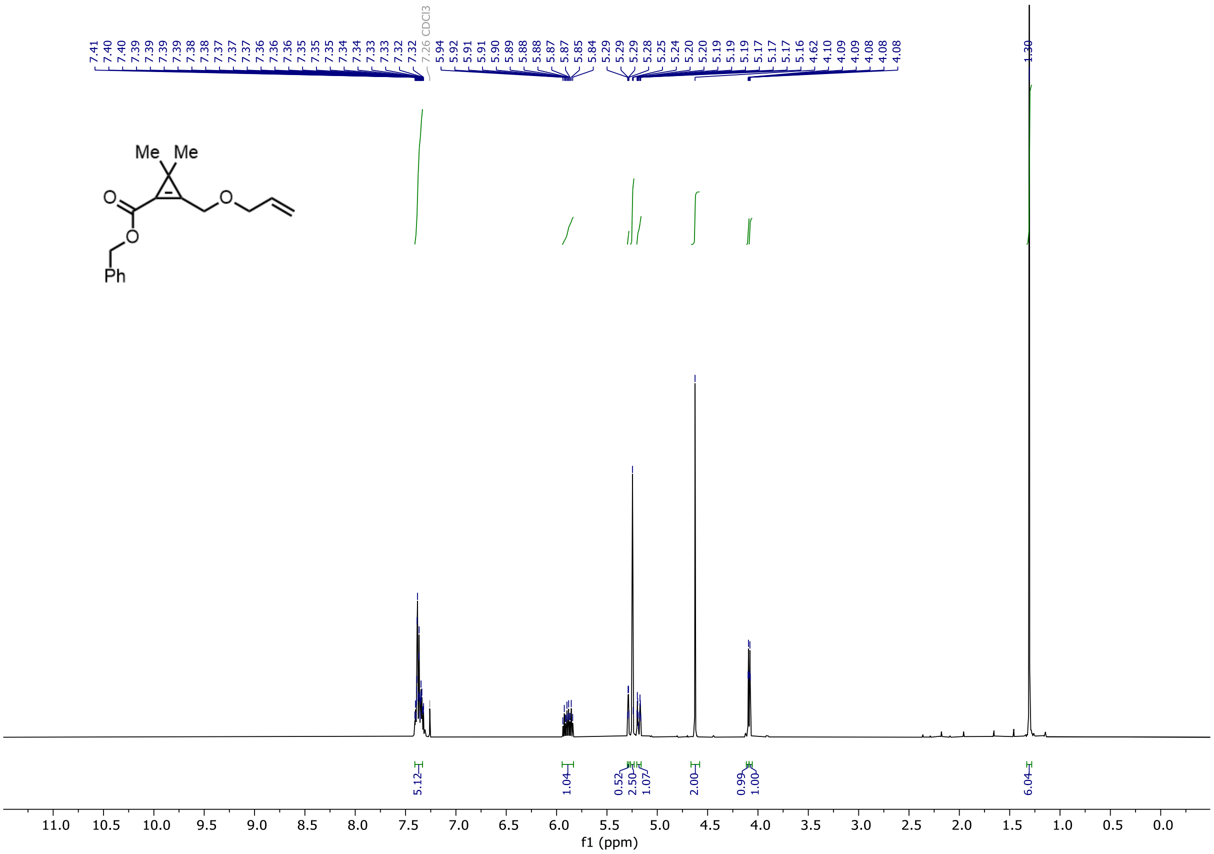

[13]**C NMR** (101 MHz, $CDCl_3$) of **39a**

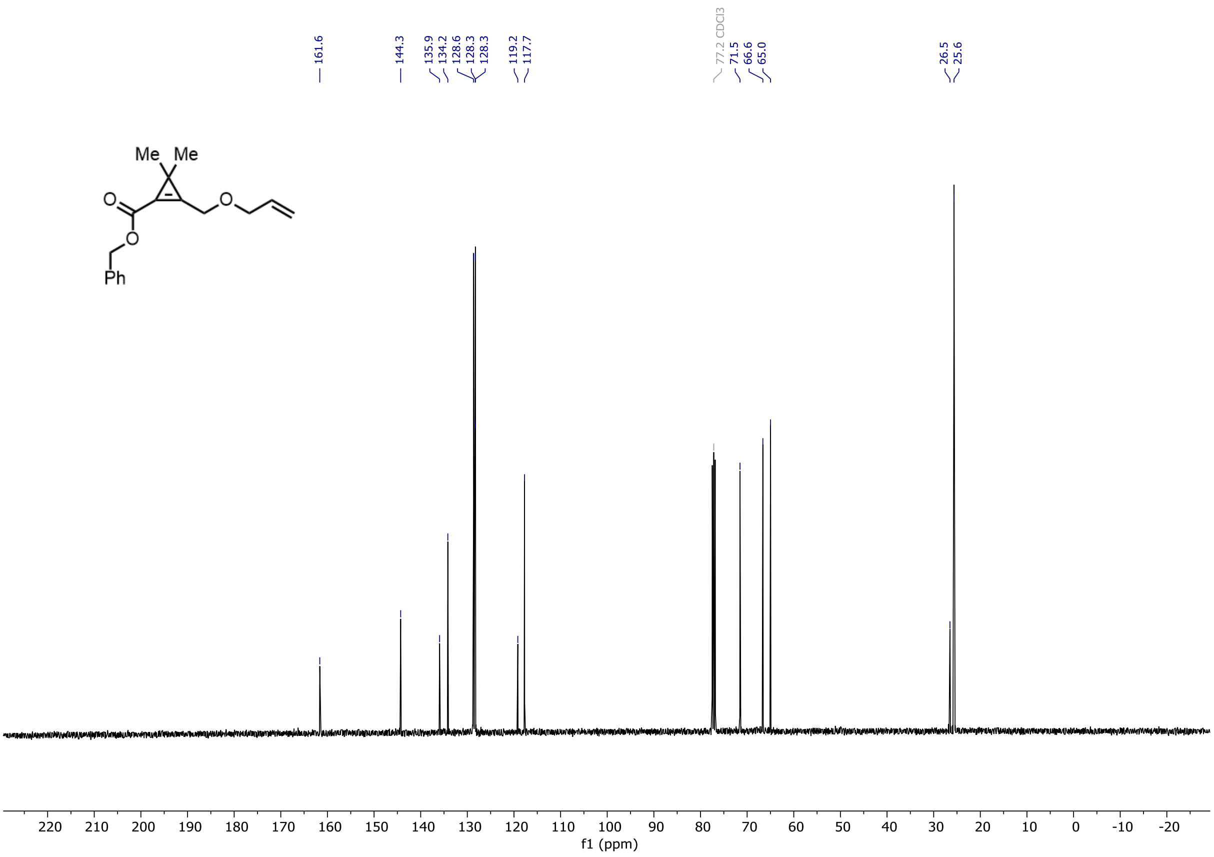

^{1}H NMR (500 MHz, $CDCl_3$) of **3b**

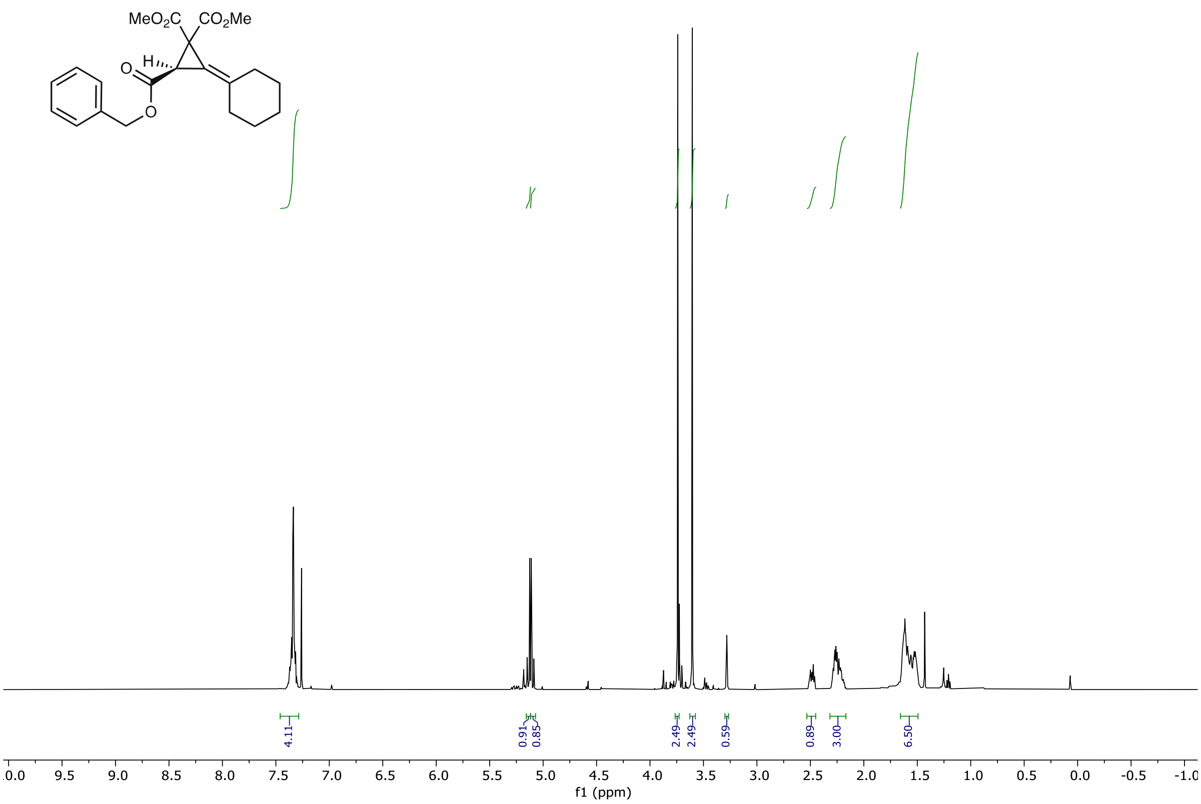

^{13}C NMR (126 MHz, $CDCl_3$) of **3b**

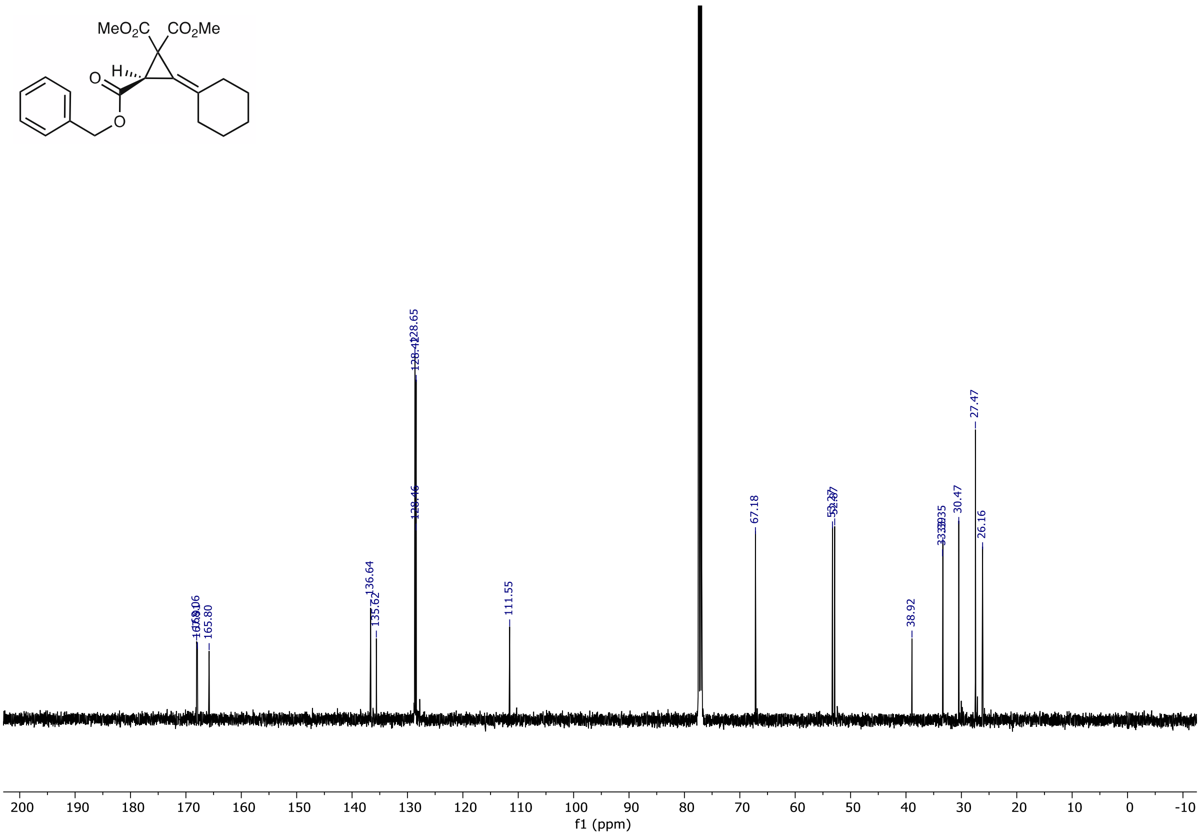

^{1}H NMR (500 MHz, $CDCl_3$) of **5b**

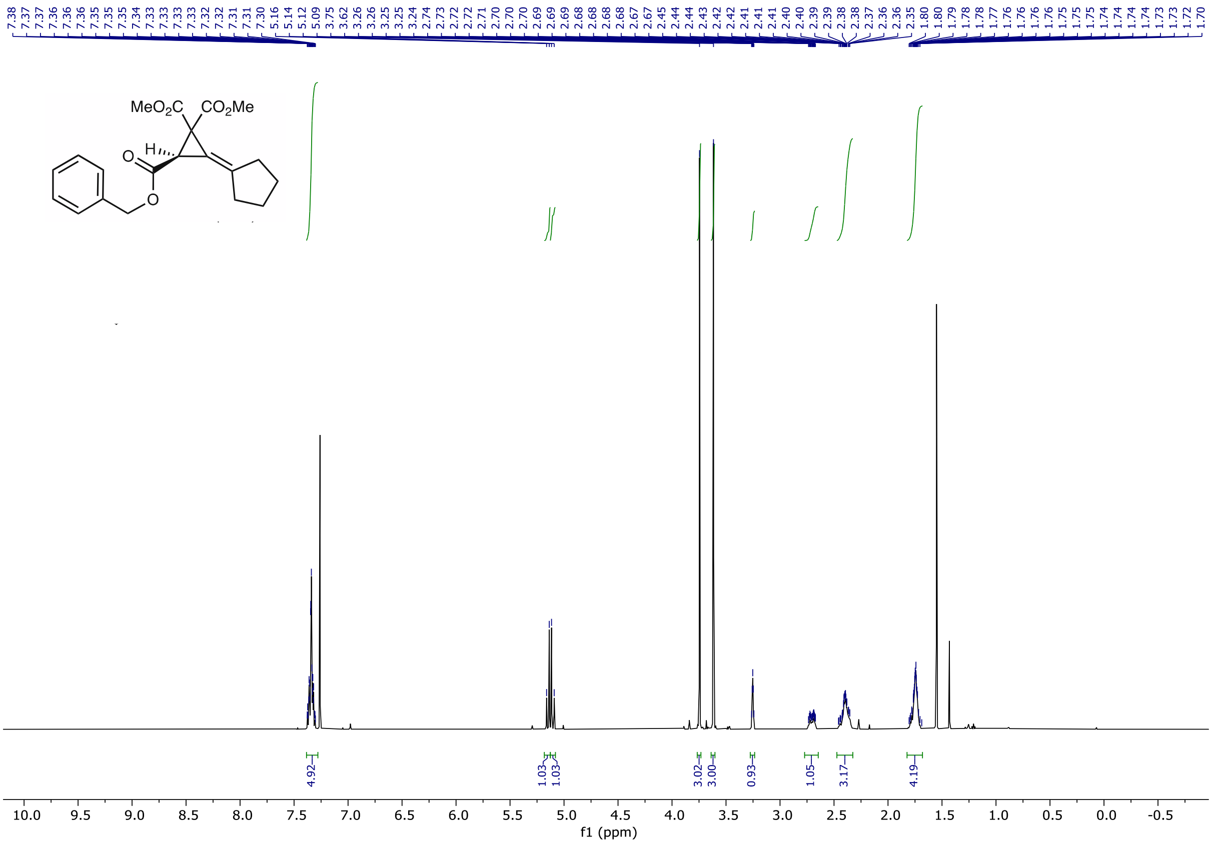

^{13}C NMR (126 MHz, $CDCl_3$) of **5b**

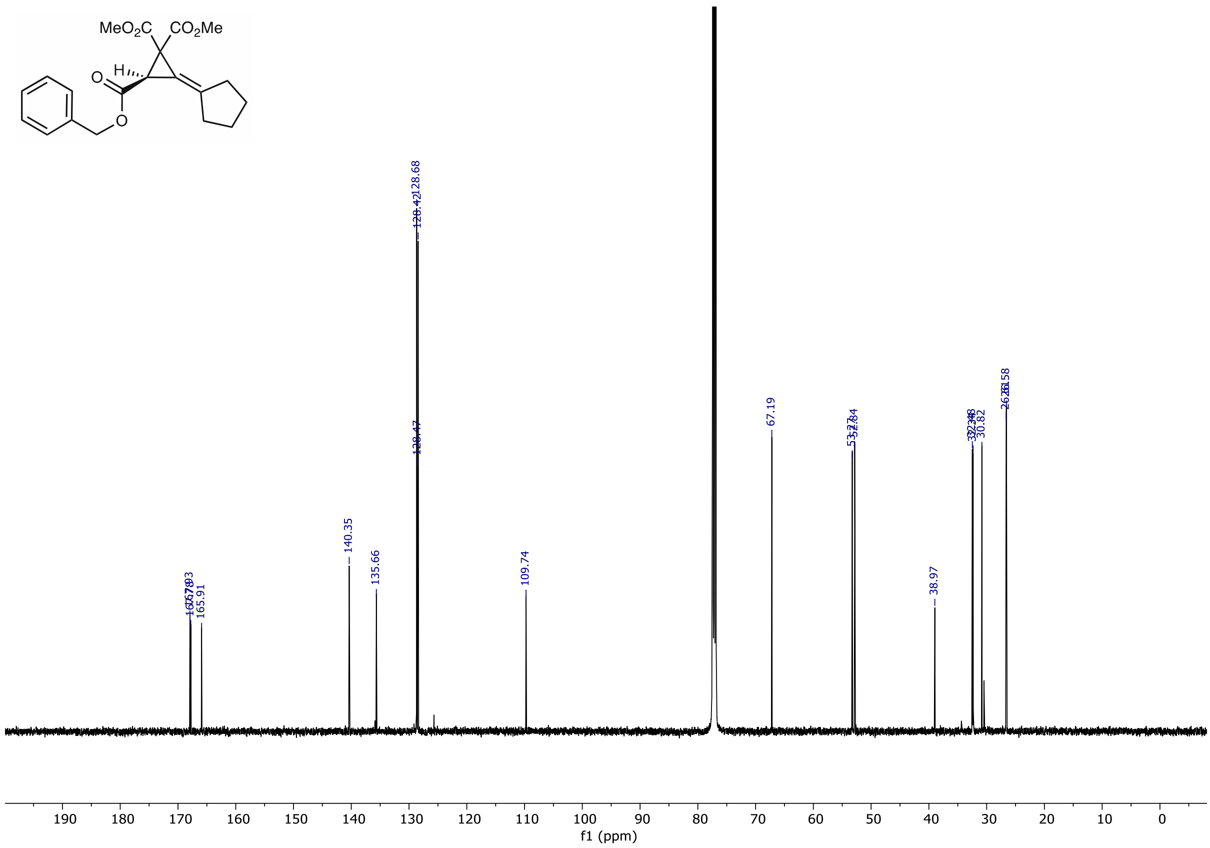

¹H NMR (400 MHz, $CDCl_3$) of **6b**

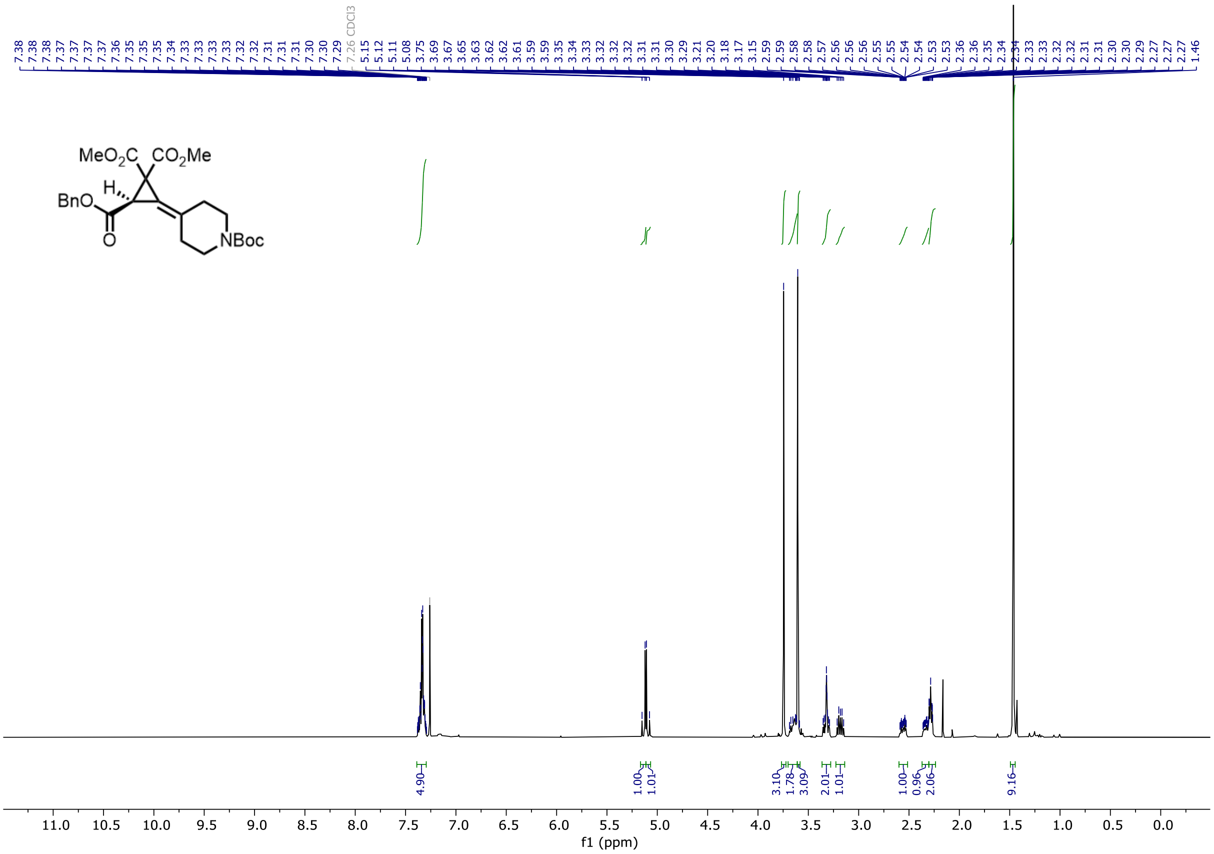

¹³C NMR (101 MHz, $CDCl_3$) of **6b**

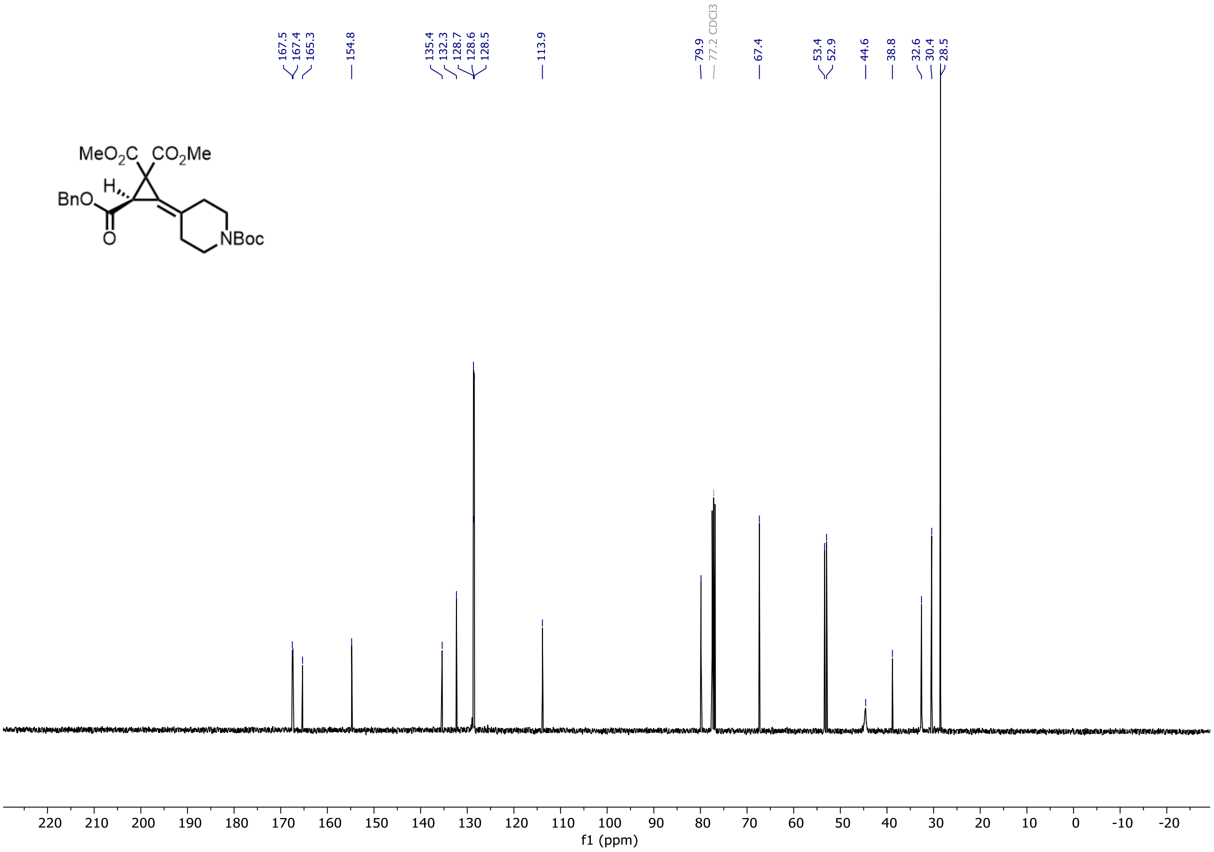

^{1}H NMR (400 MHz, $CDCl_3$) of **7b**

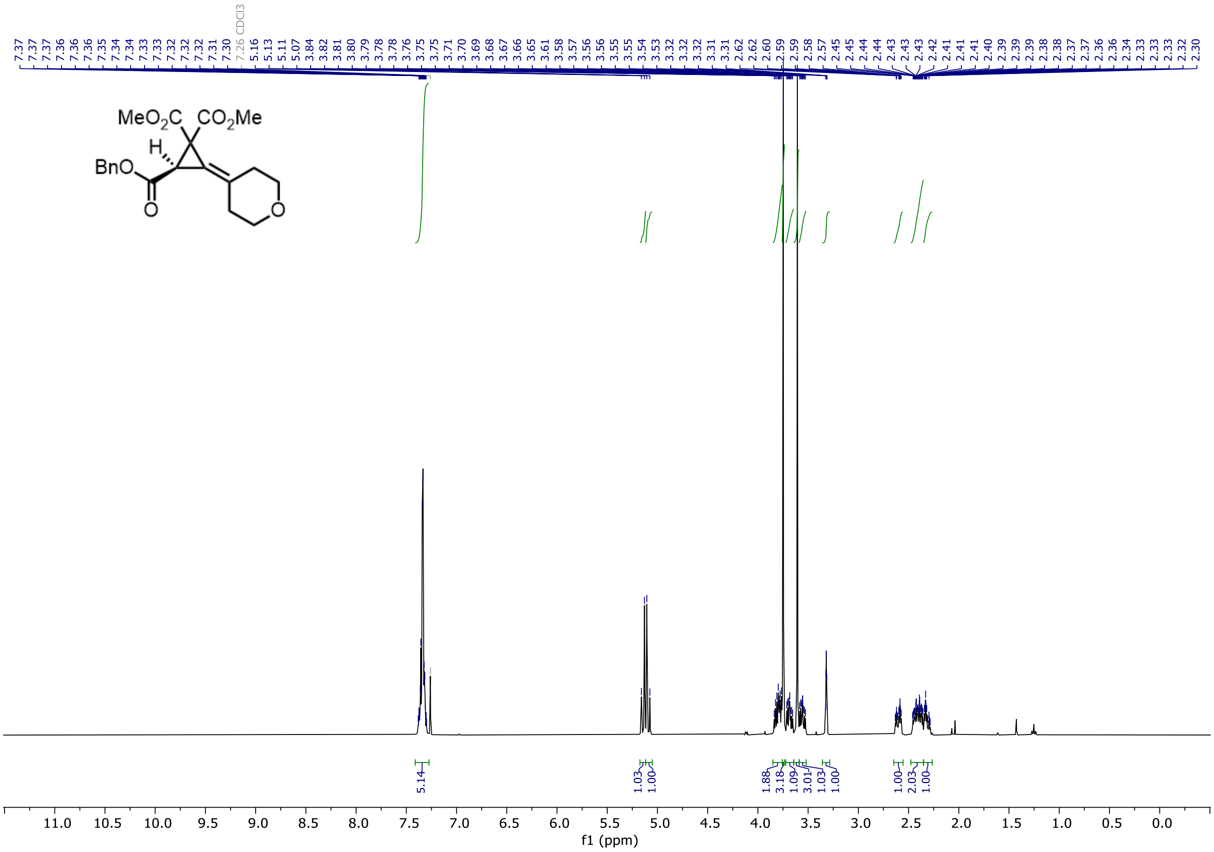

^{13}C NMR (101 MHz, $CDCl_3$) of **7b**

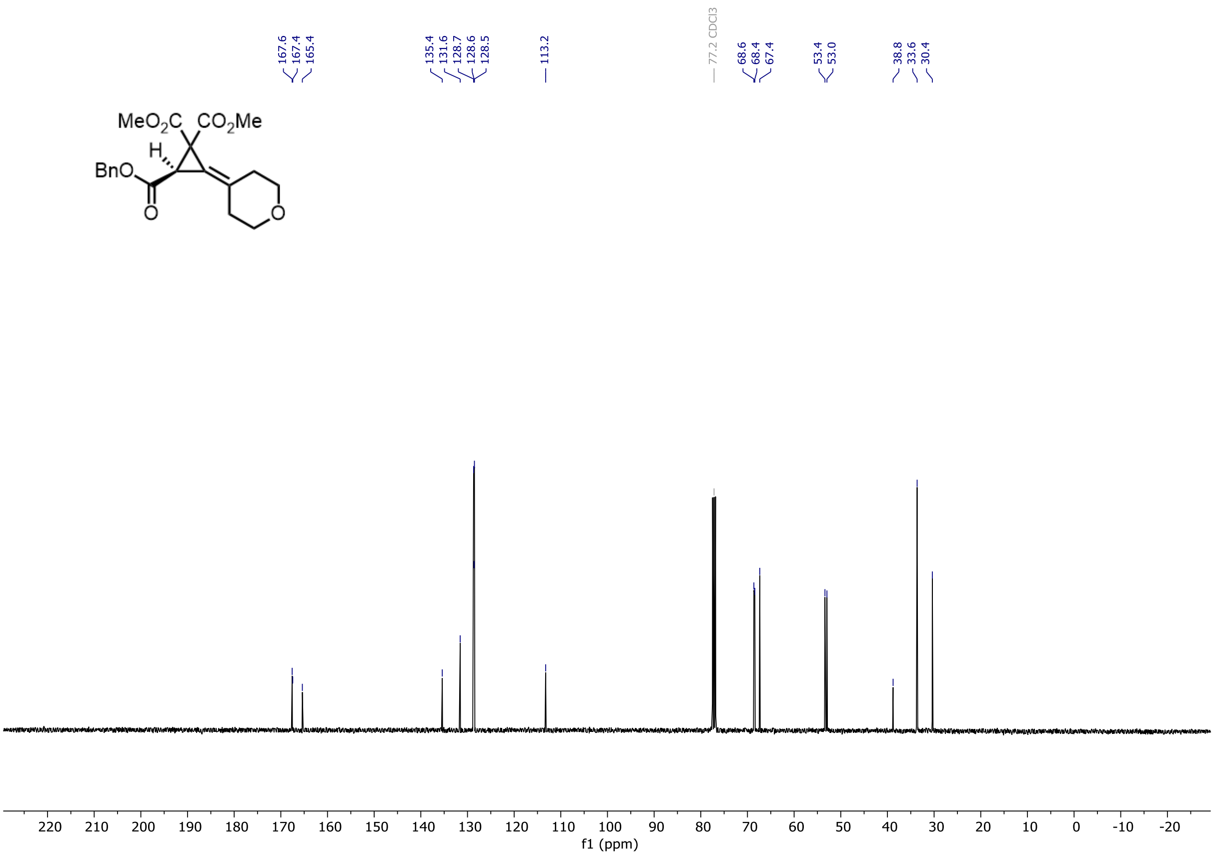

^{1}H NMR (400 MHz, $CDCl_3$) of **8b**

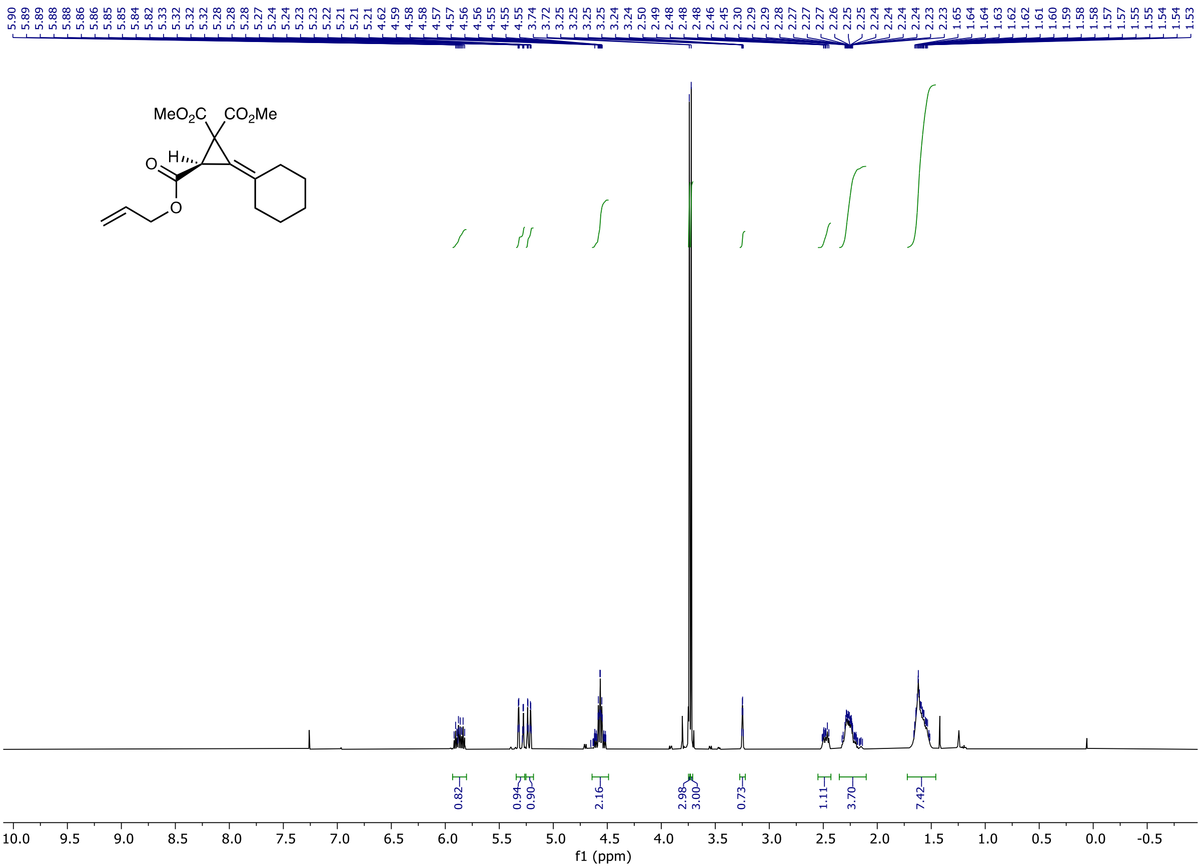

^{13}C NMR (101 MHz, $CDCl_3$) of **8b**

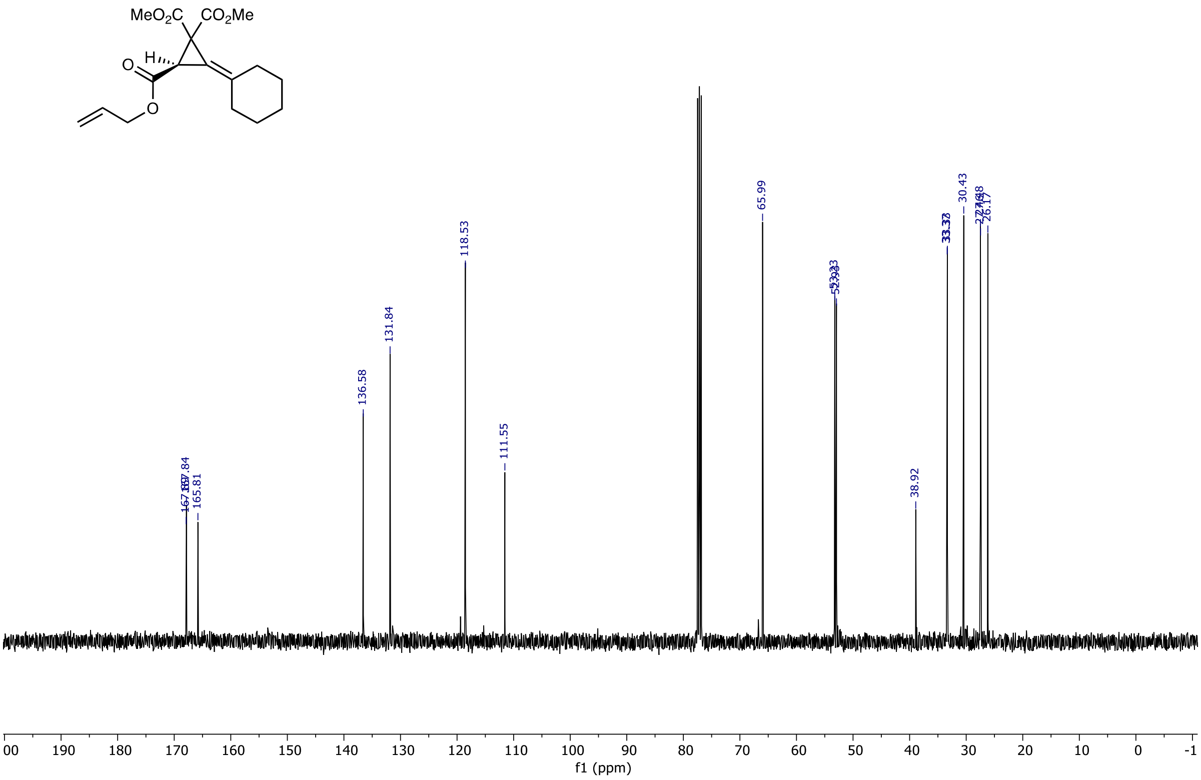

^{1}H NMR (500 MHz, $CDCl_3$) of **9b**

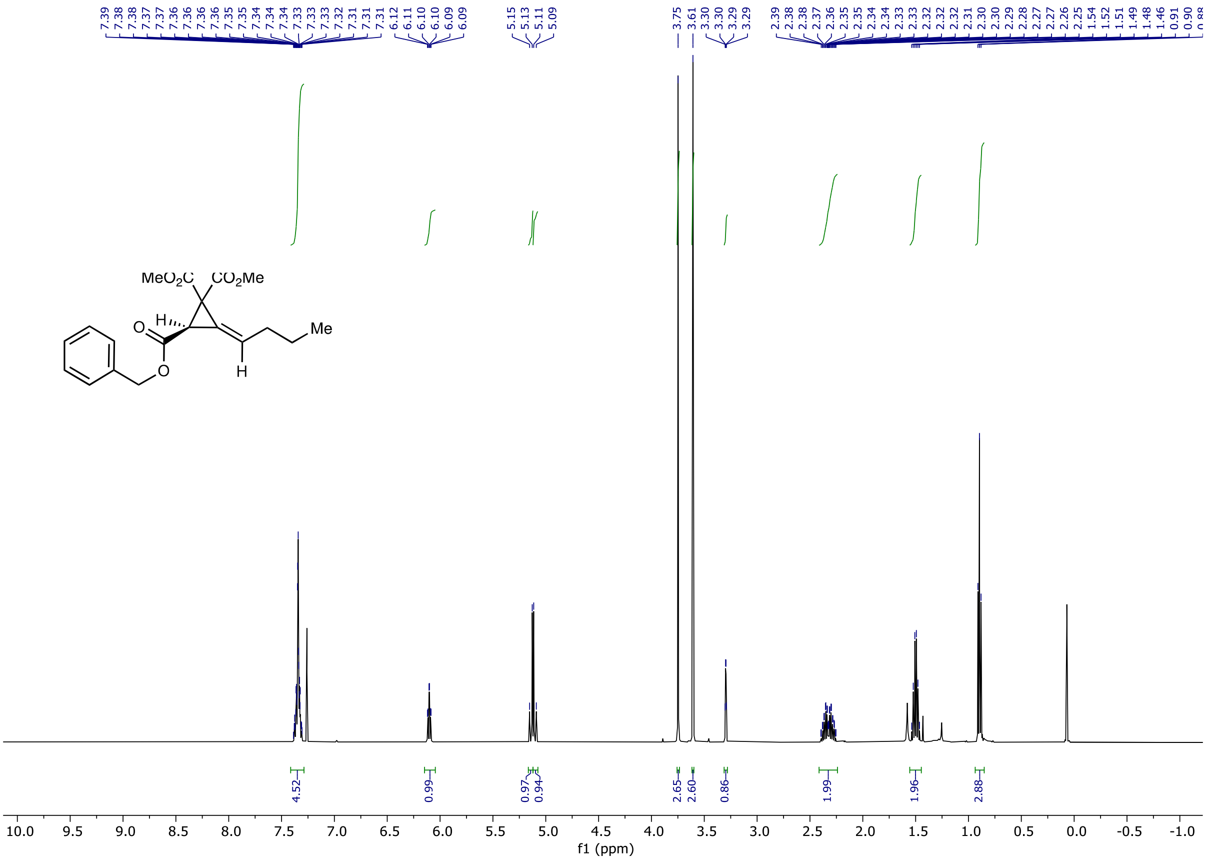

^{13}C NMR (126 MHz, $CDCl_3$) of **9b**

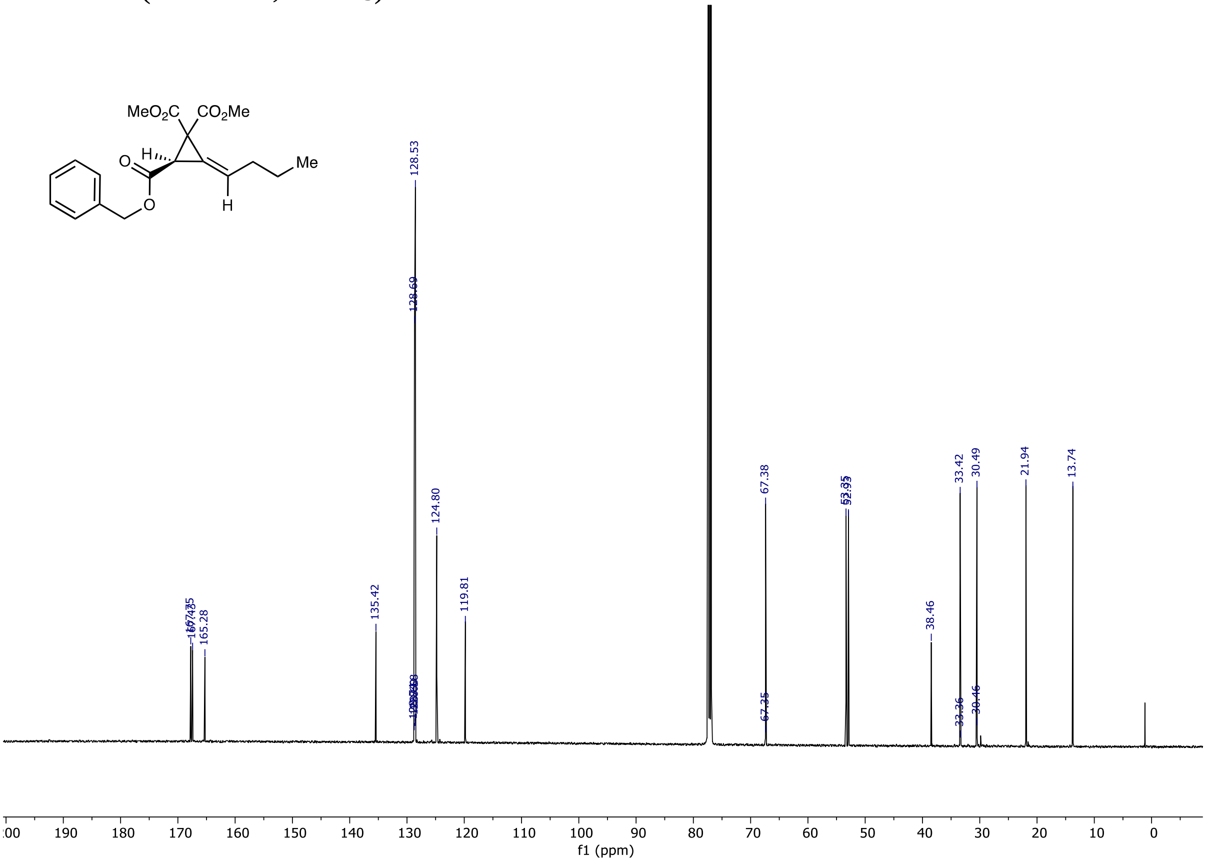

^{1}H NMR (500 MHz, $CDCl_3$) of **10b**

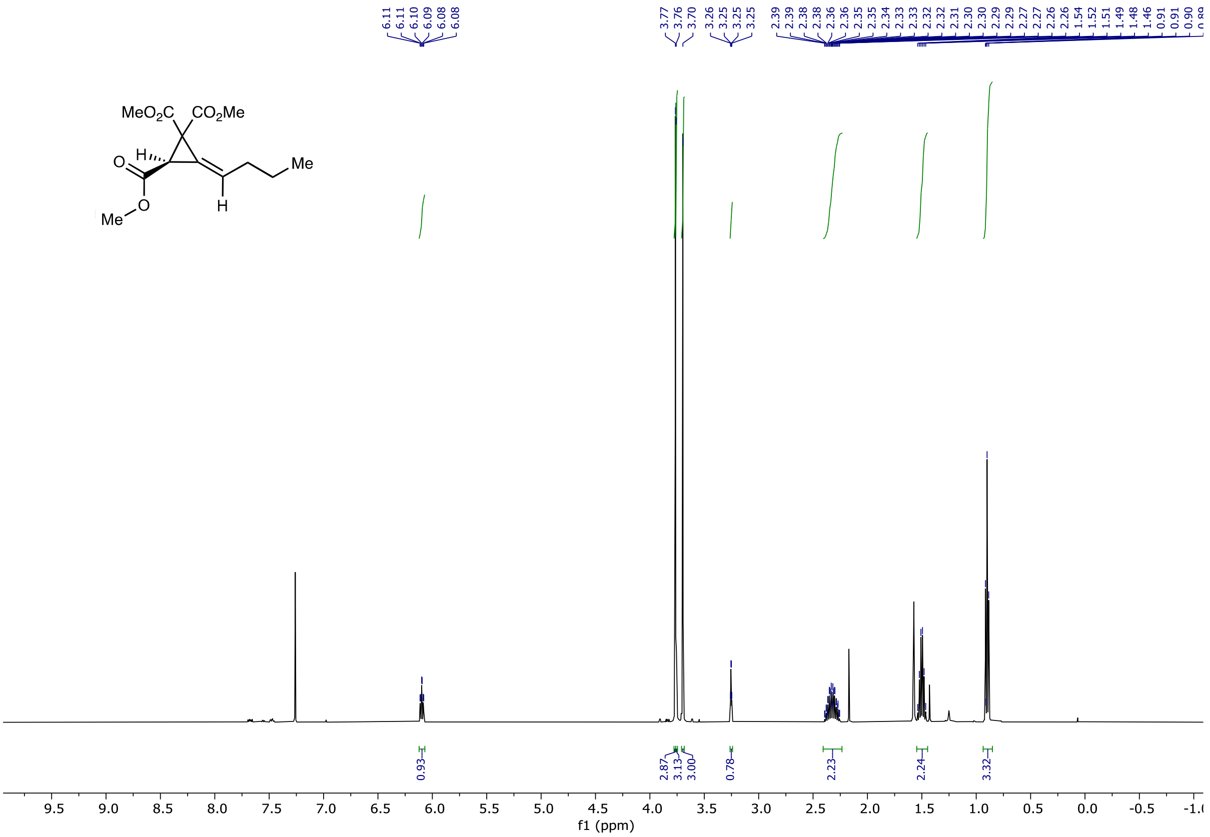

^{13}C NMR (126 MHz, $CDCl_3$) of **10b**

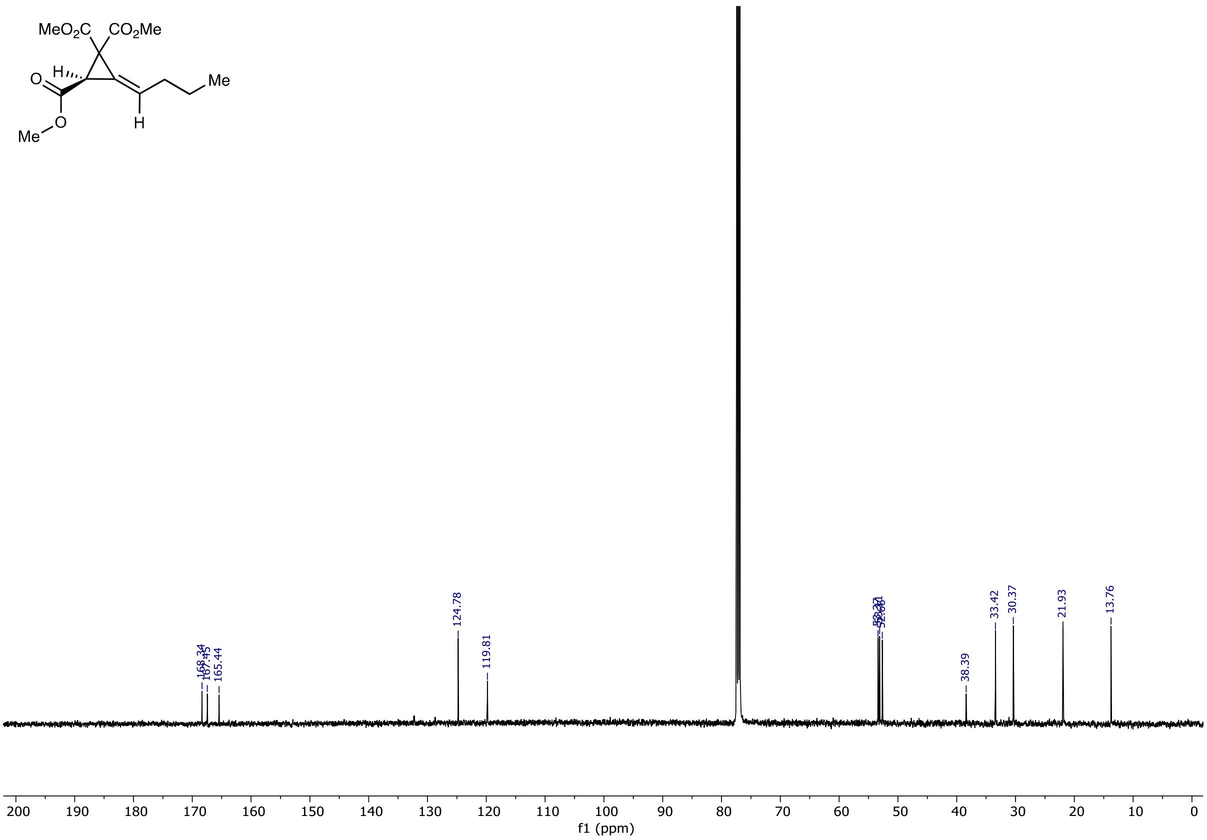

^{1}H NMR (500 MHz, $CDCl_3$) of **11b**

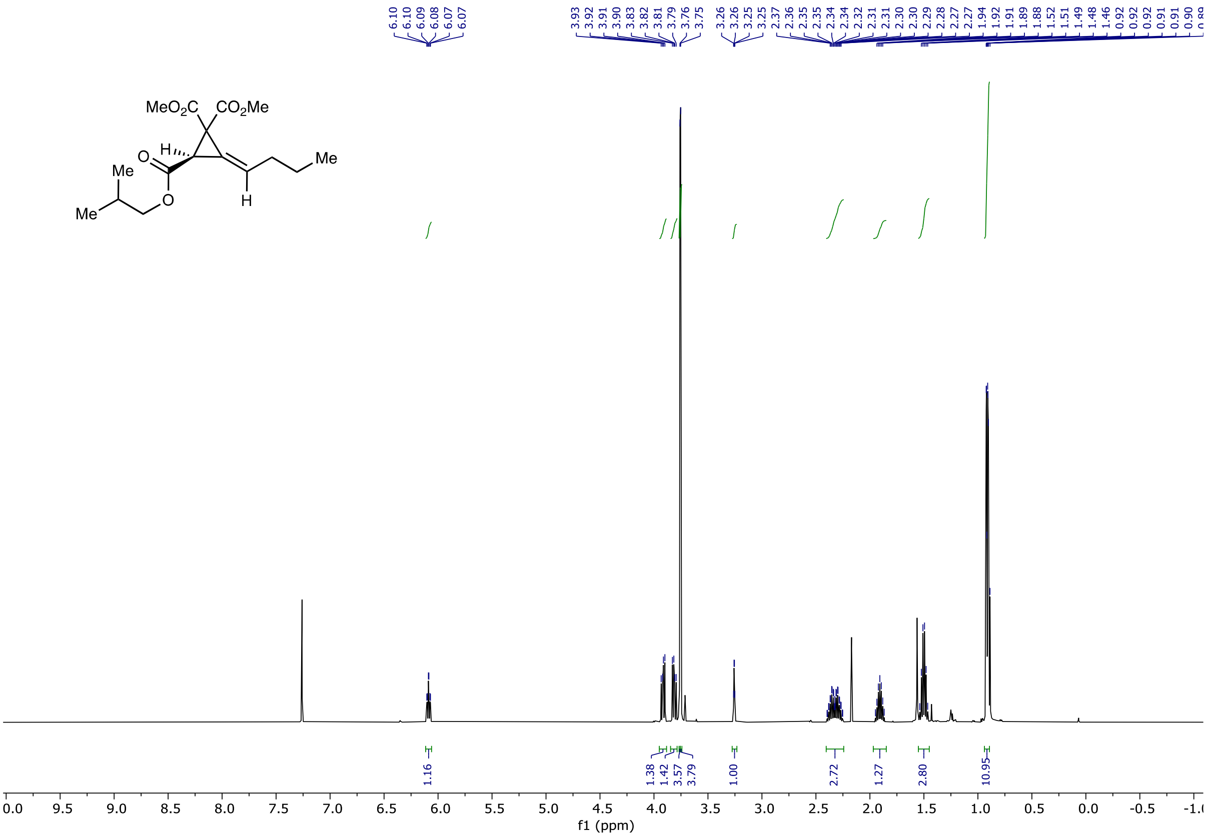

^{13}C NMR (126 MHz, $CDCl_3$) of **11b**

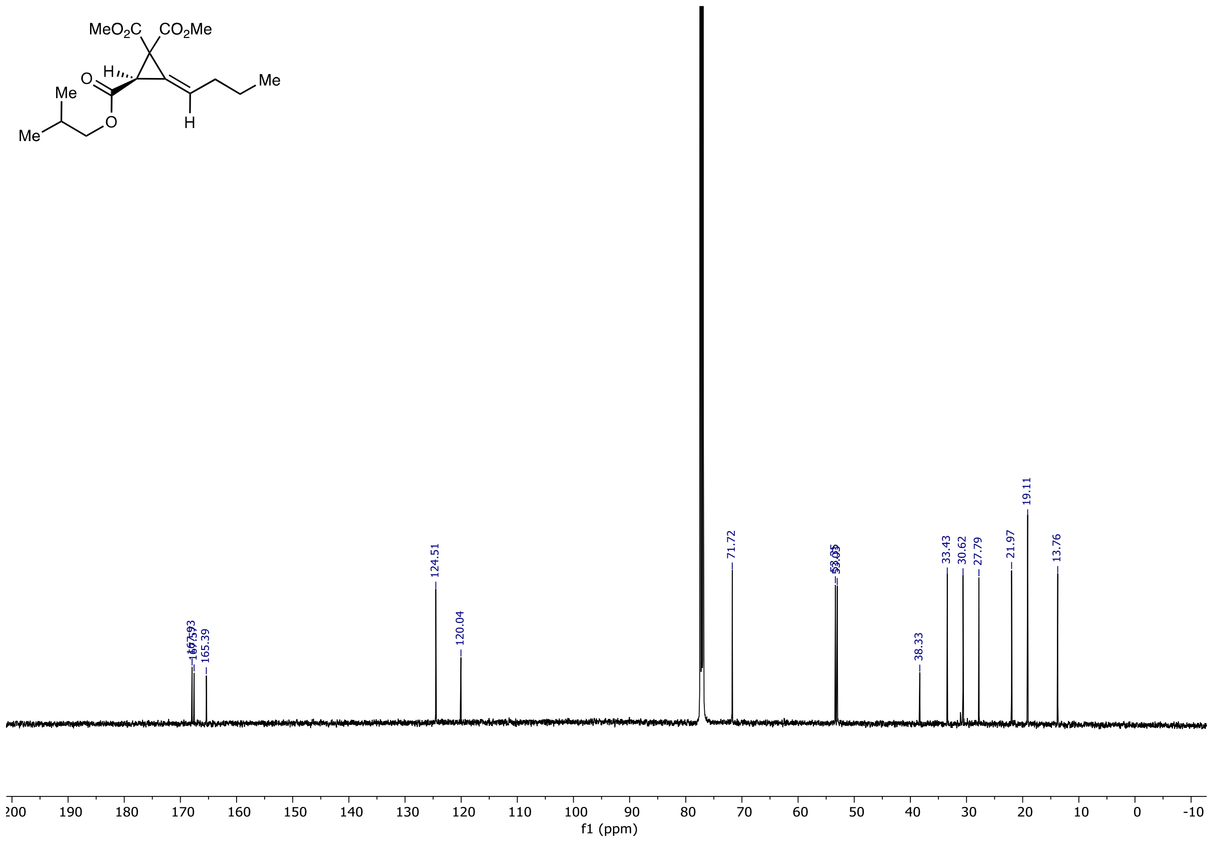

^{1}H NMR (400 MHz, $CDCl_3$) of **12b**

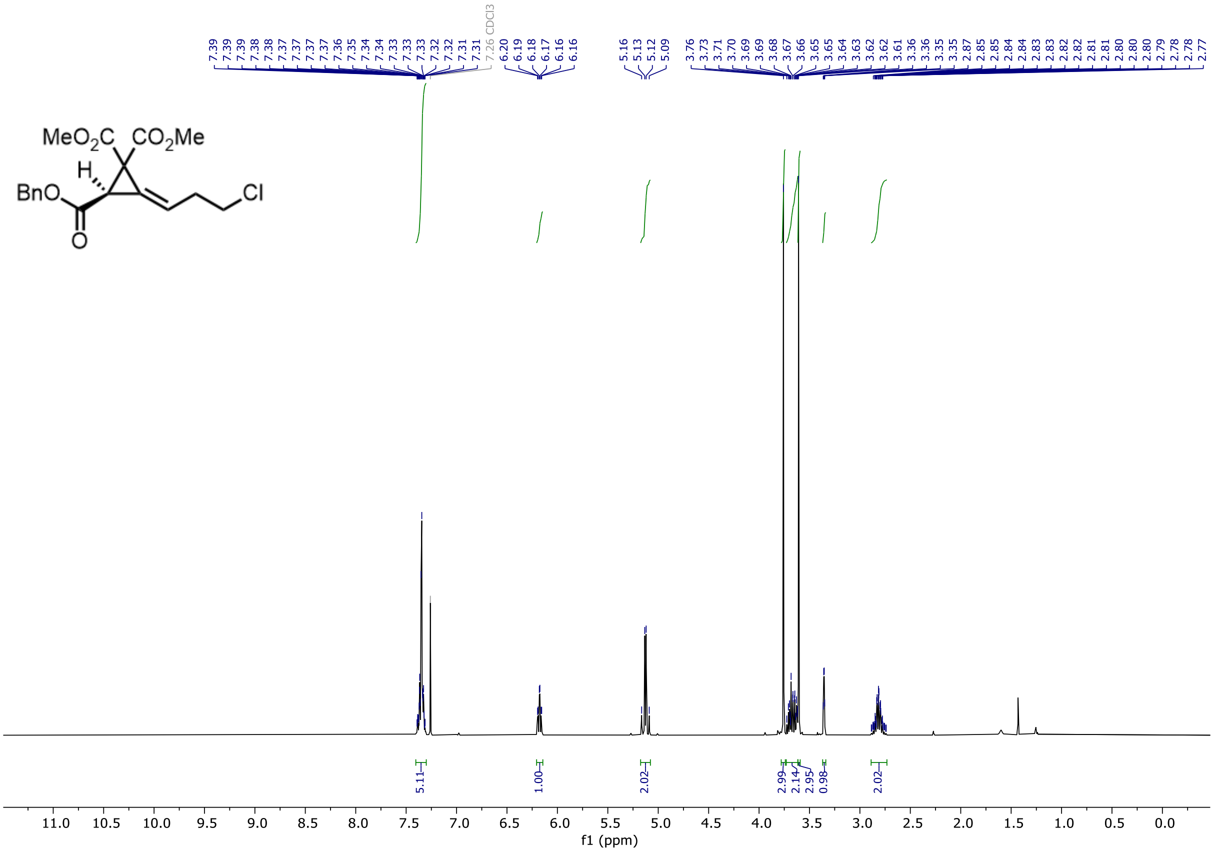

^{13}C NMR (101 MHz, $CDCl_3$) of **12b**

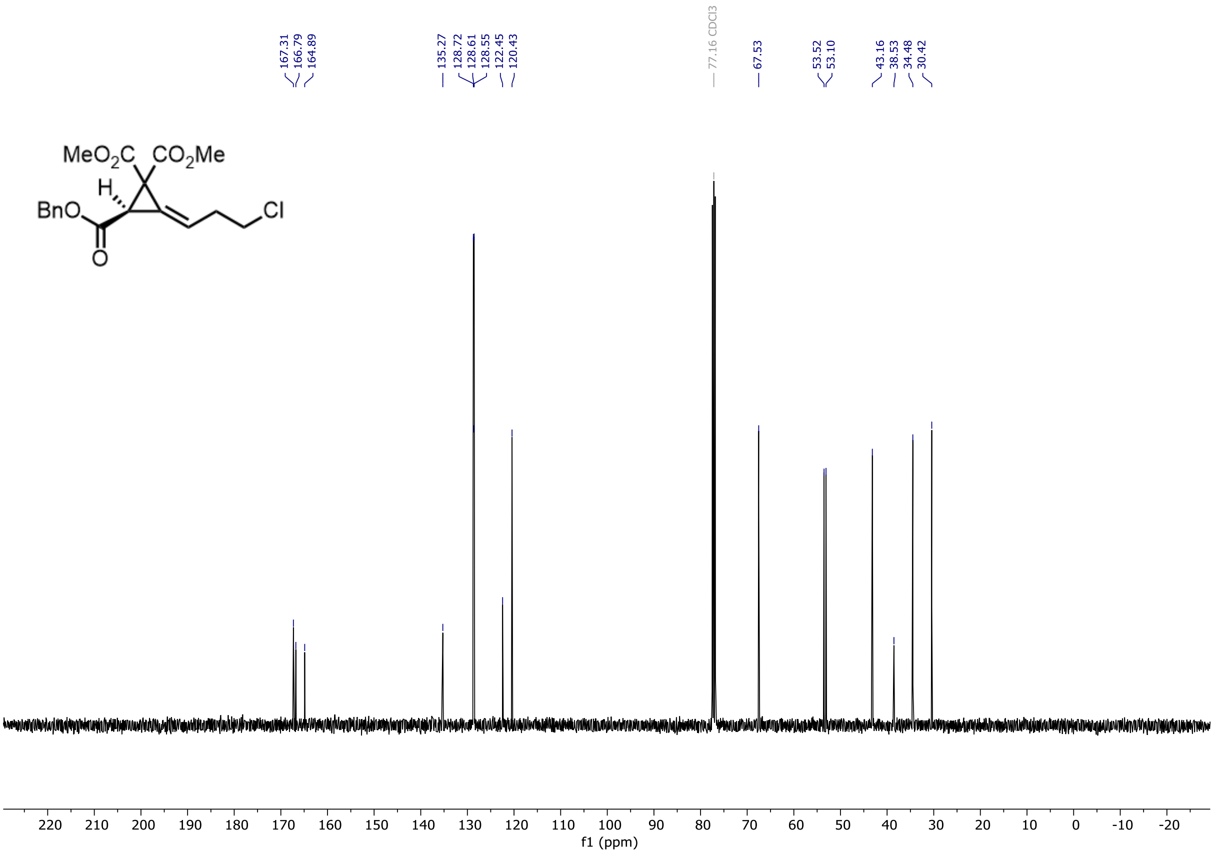

^{1}H NMR (400 MHz, $CDCl_3$) of **13b**

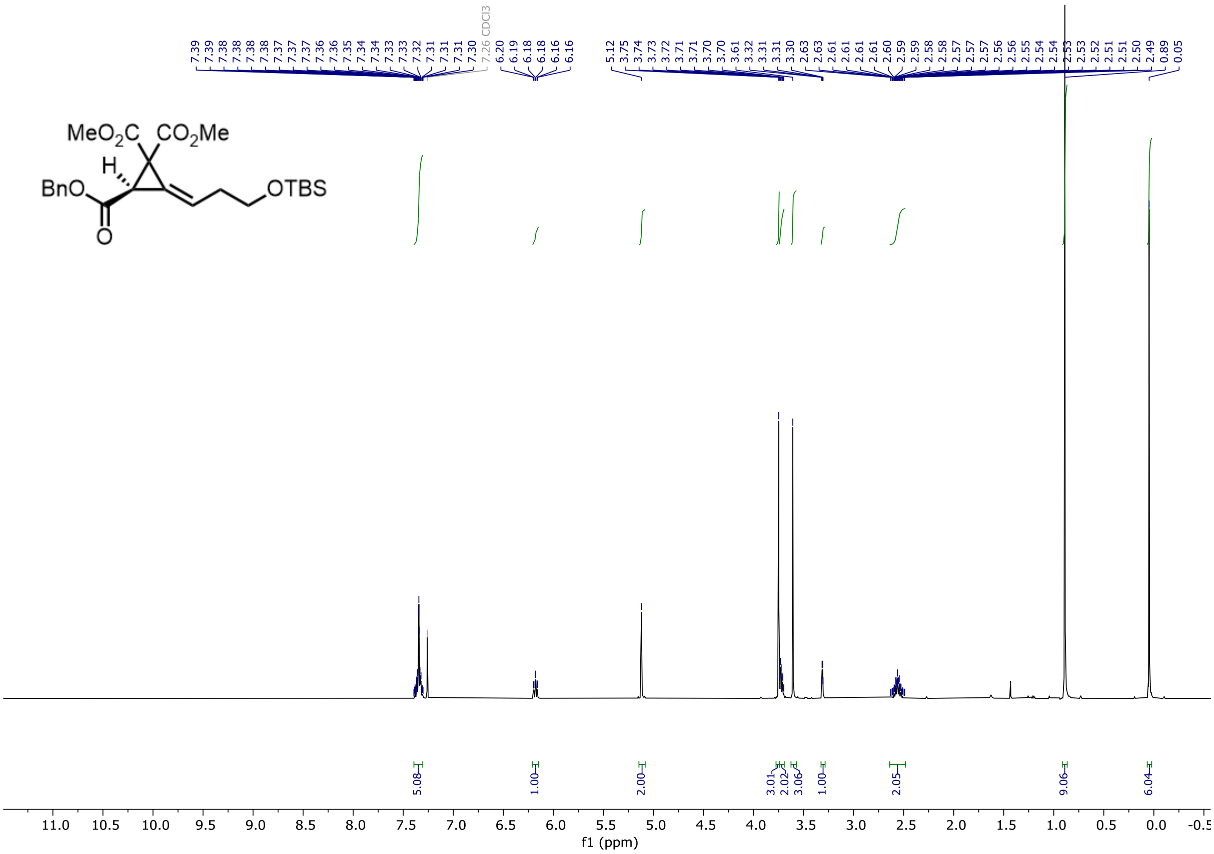

^{13}C NMR (101 MHz, $CDCl_3$) of **13b**

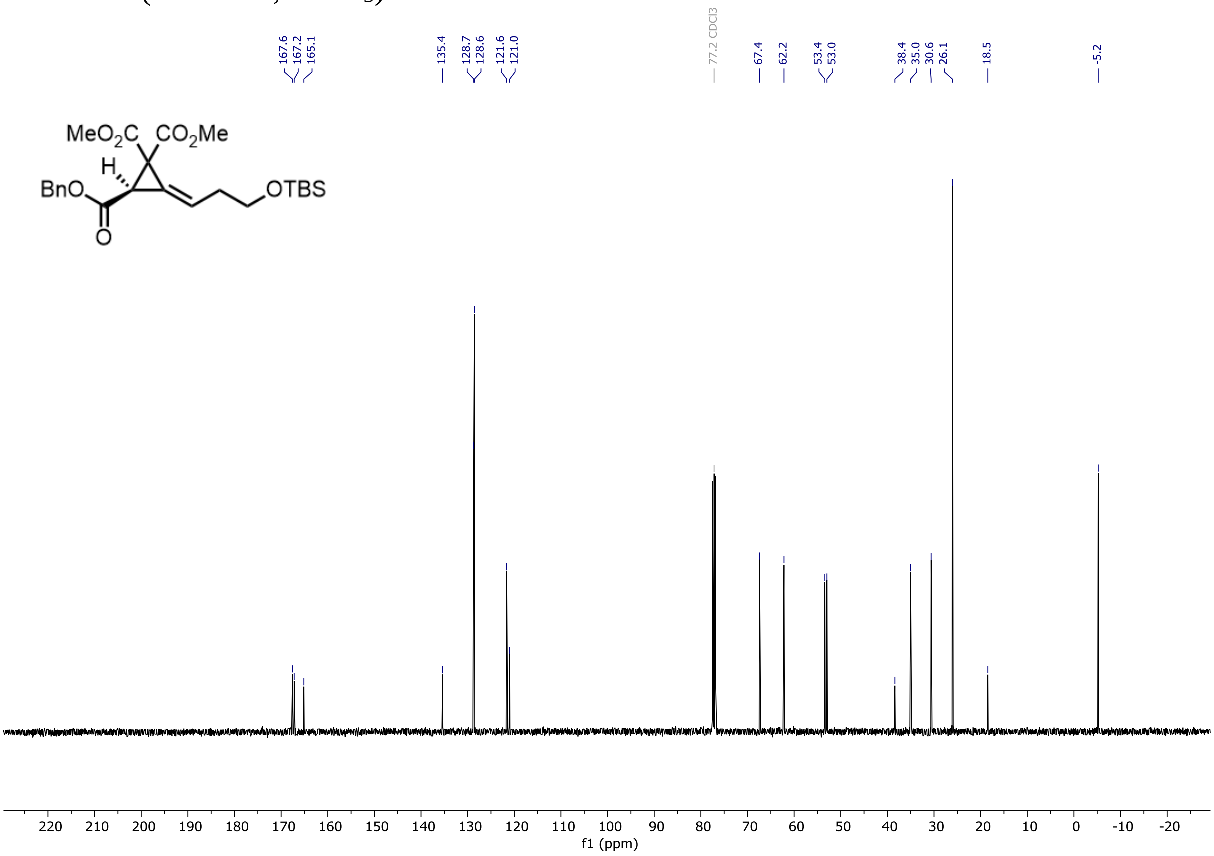

^{1}H NMR (400 MHz, $CDCl_3$) of **14b**

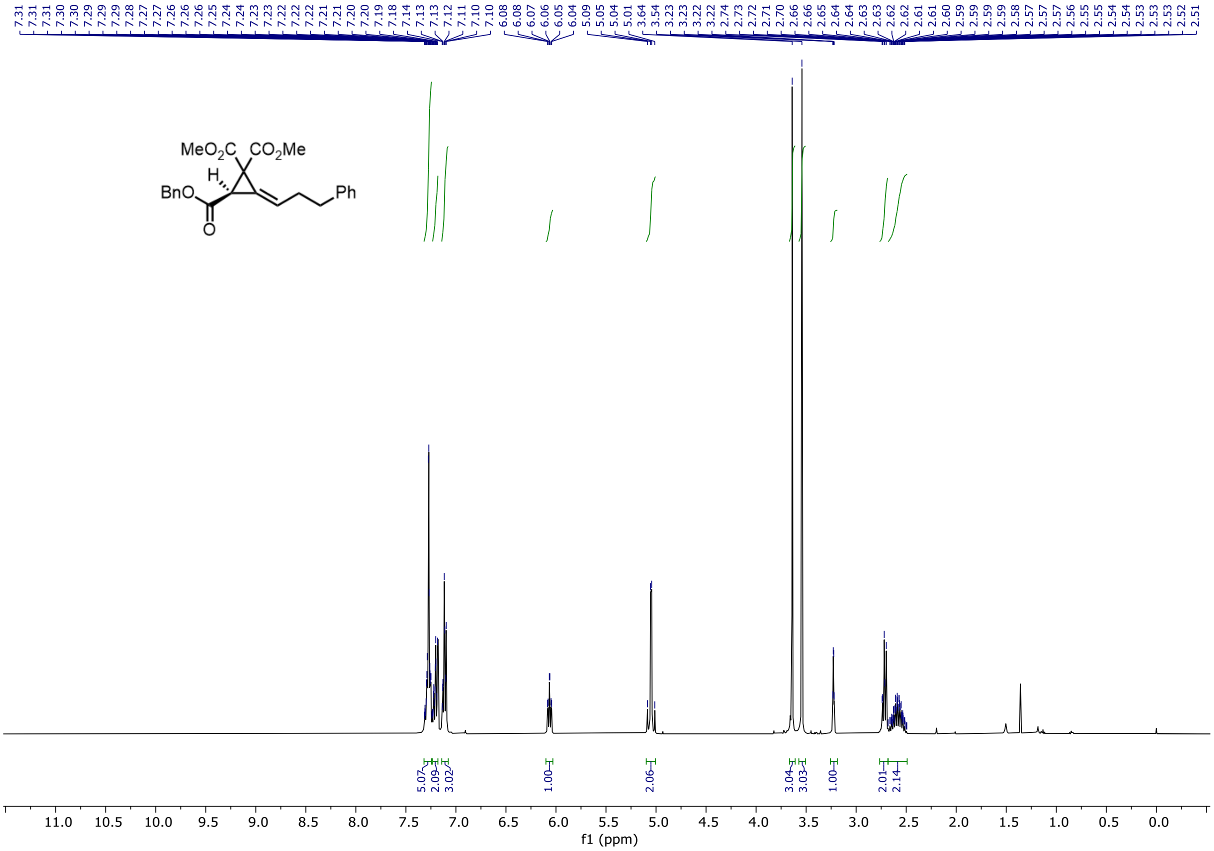

^{13}C NMR (126 MHz, $CDCl_3$) of **14b**

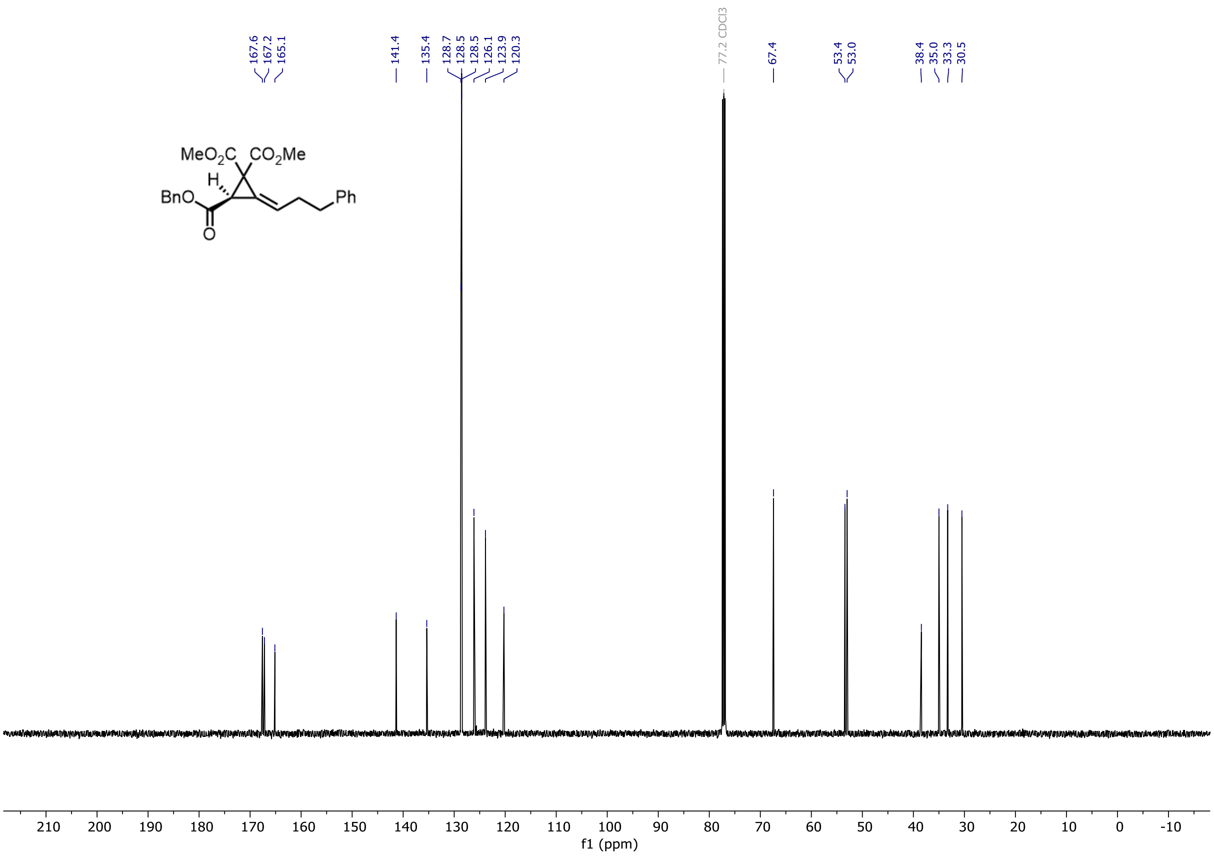

^{1}H NMR (400 MHz, $CDCl_3$) of **15b**

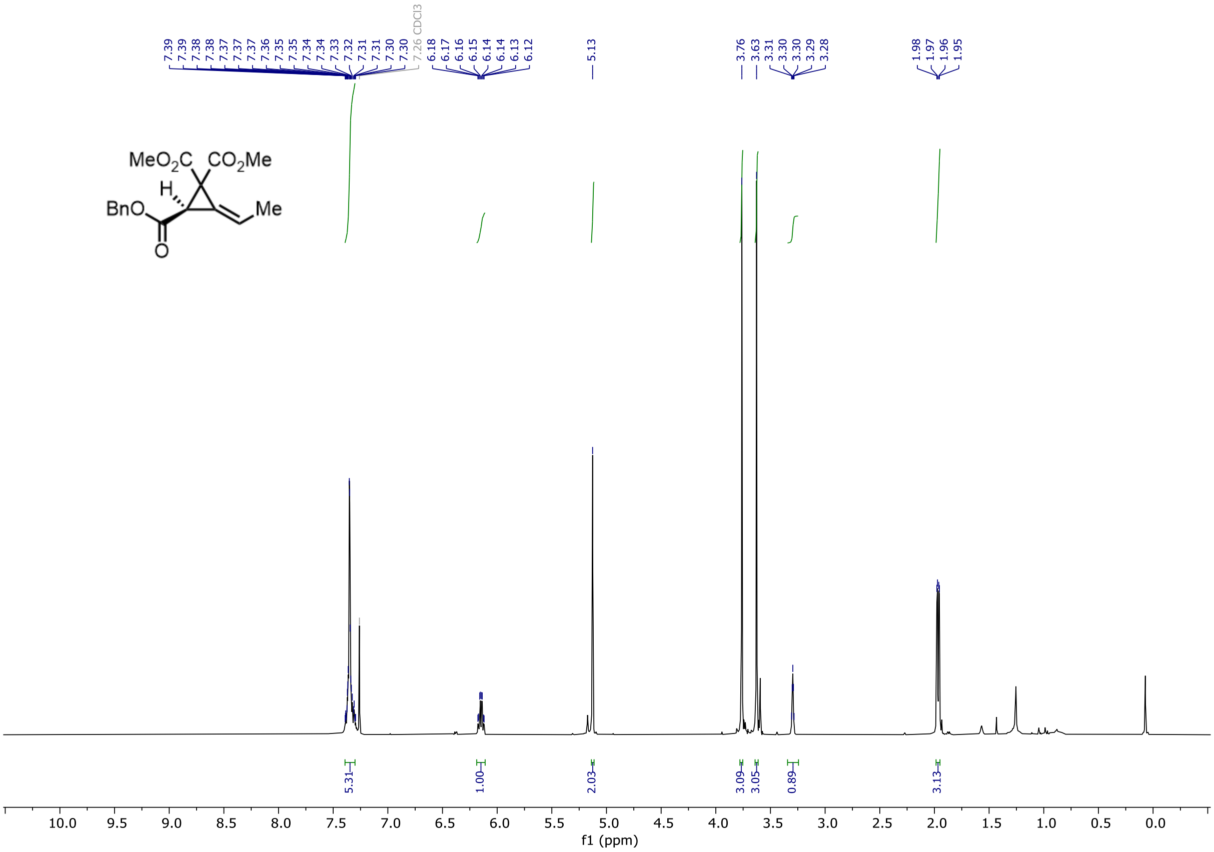

^{13}C NMR (101 MHz, $CDCl_3$) of **15b**

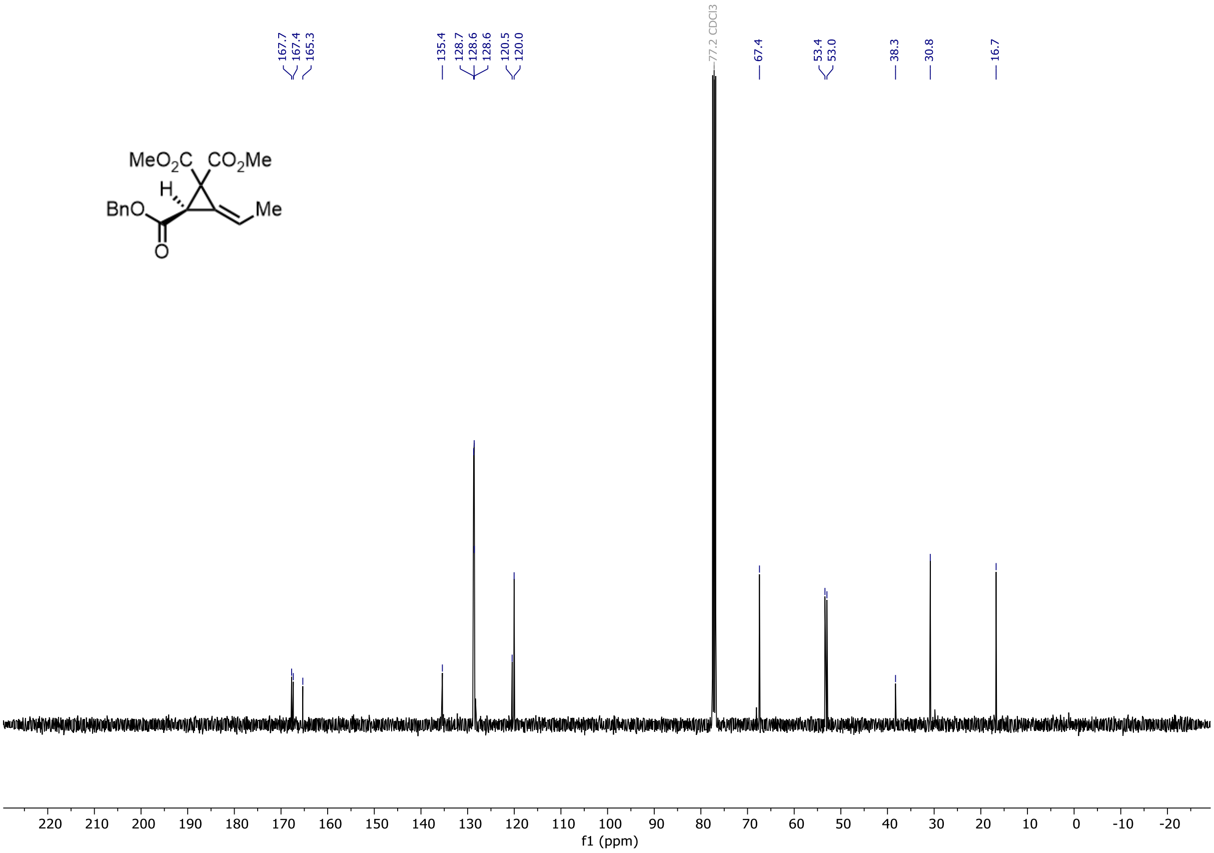

^{1}H NMR (400 MHz, $CDCl_3$) of **16b**

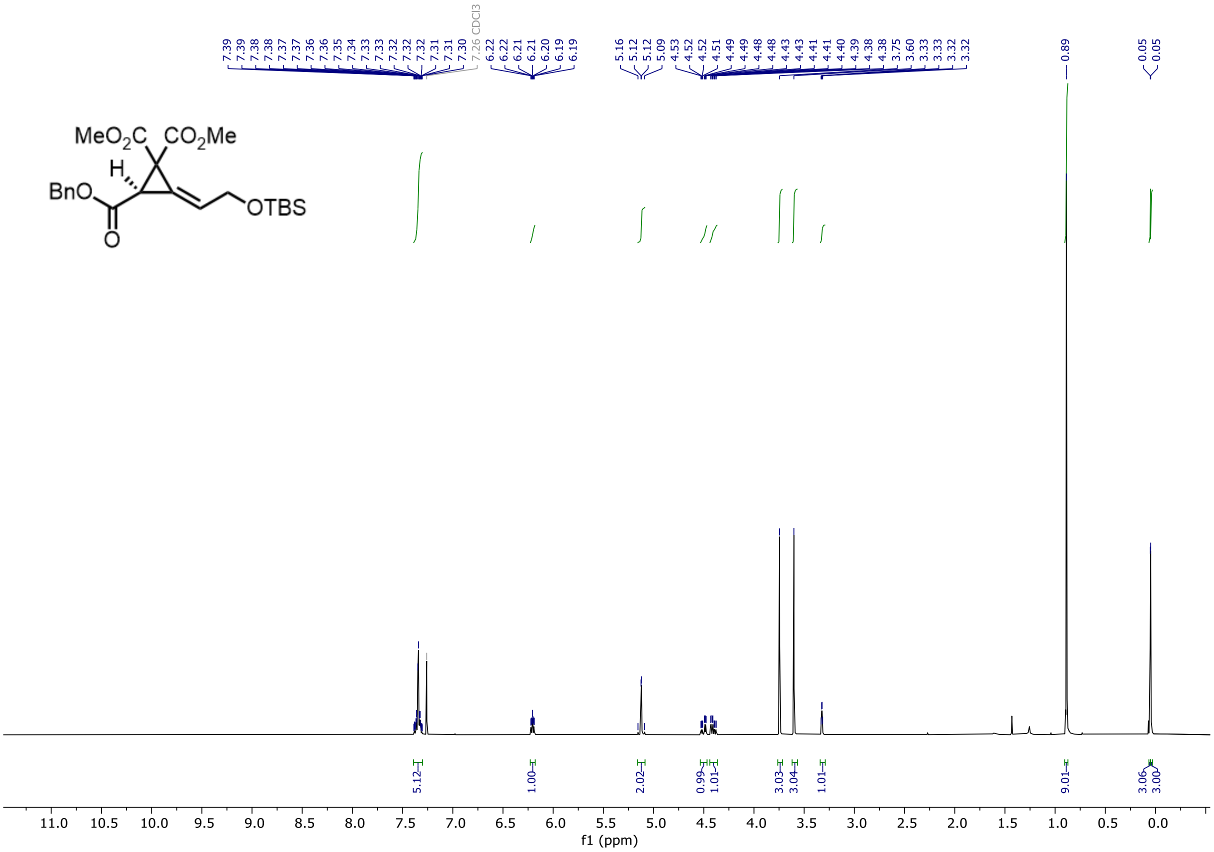

^{13}C NMR (101 MHz, $CDCl_3$) of **16b**

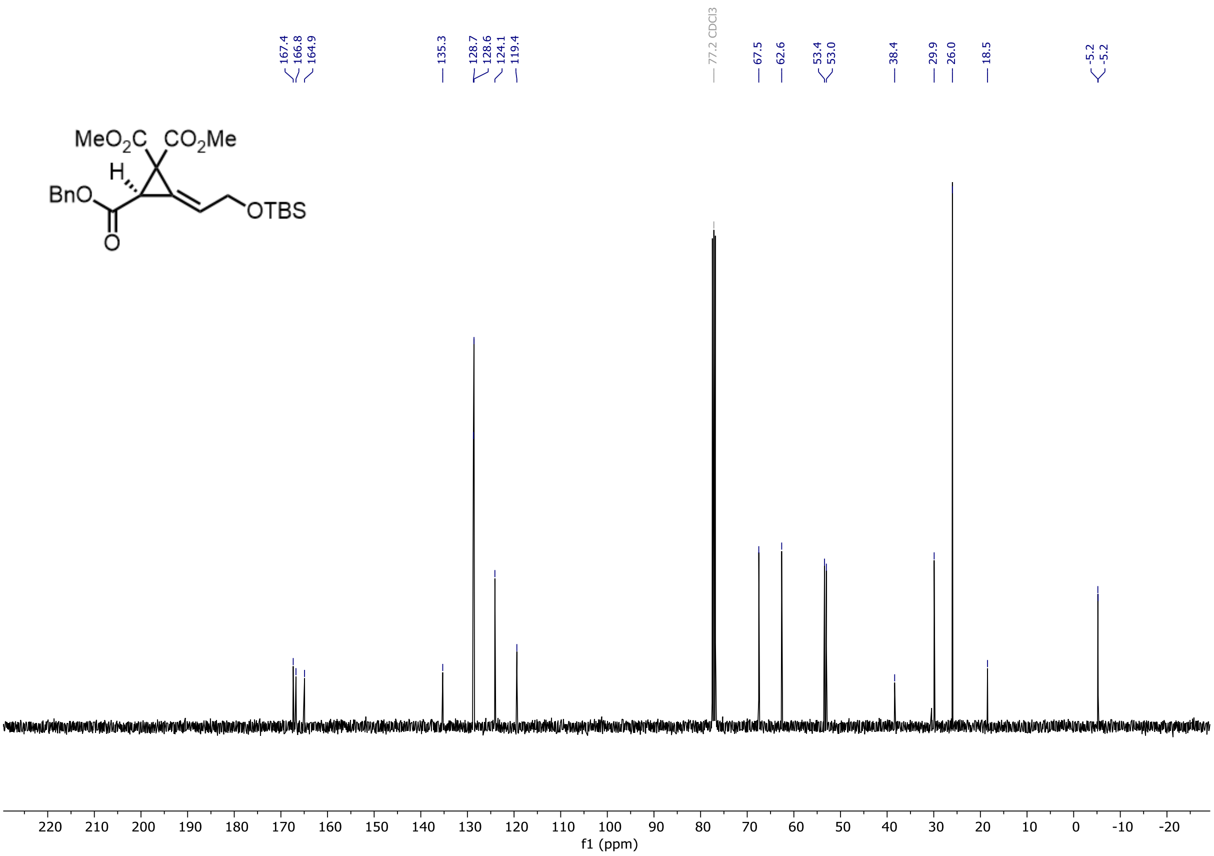

^{1}**H NMR** (400 MHz, $CDCl_3$) of **17b**

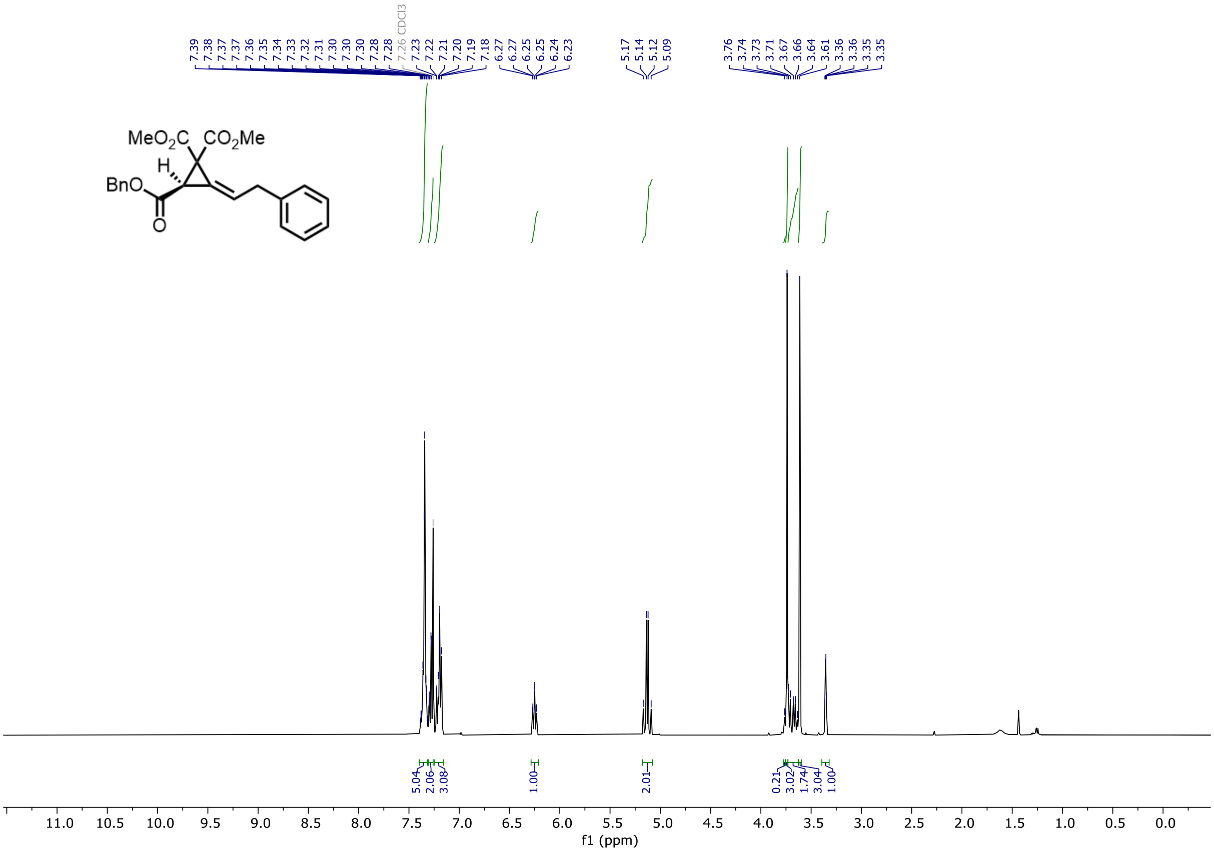

^{13}**C NMR** (101 MHz, $CDCl_3$) of **17b**

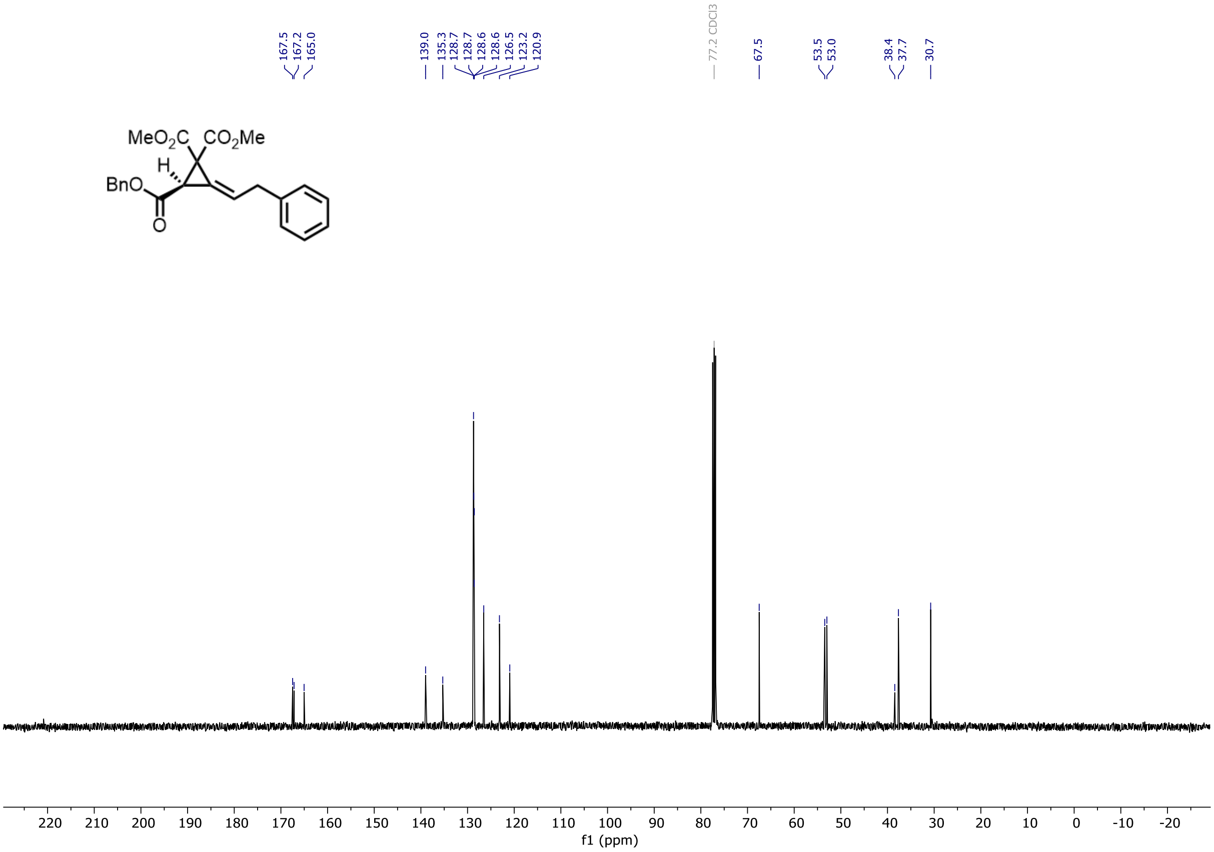

^{1}H NMR (400 MHz, $CDCl_3$) of **18b**

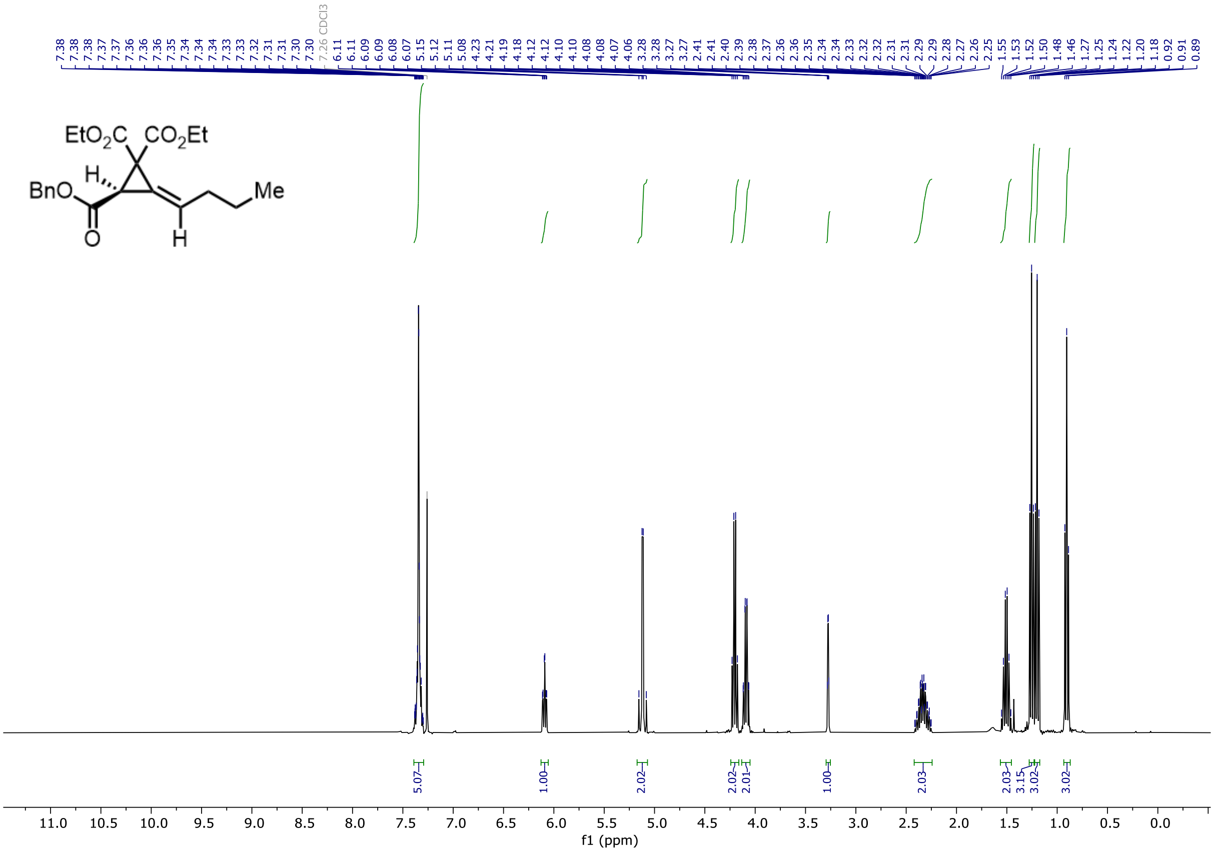

^{13}C NMR (101 MHz, $CDCl_3$) of **18b**

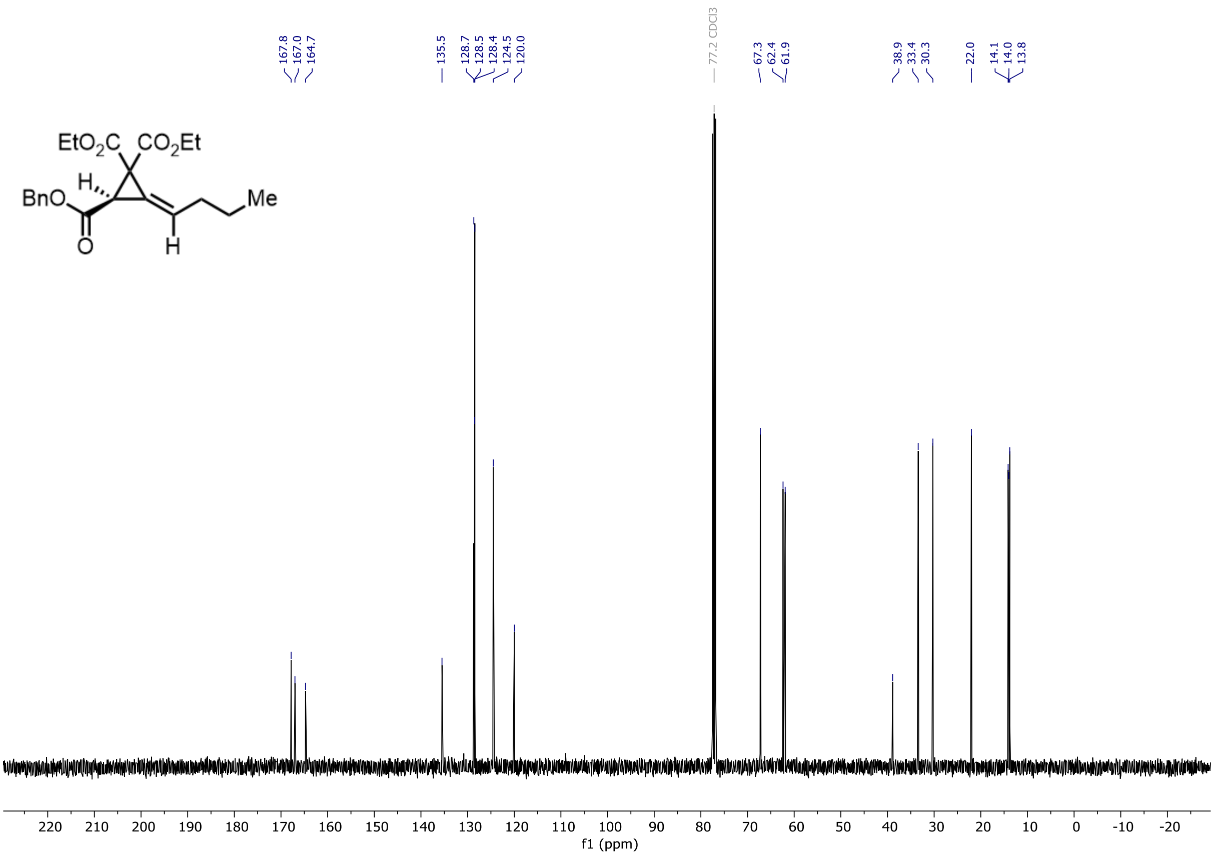

^{1}H NMR (400 MHz, $CDCl_3$) of **19b**

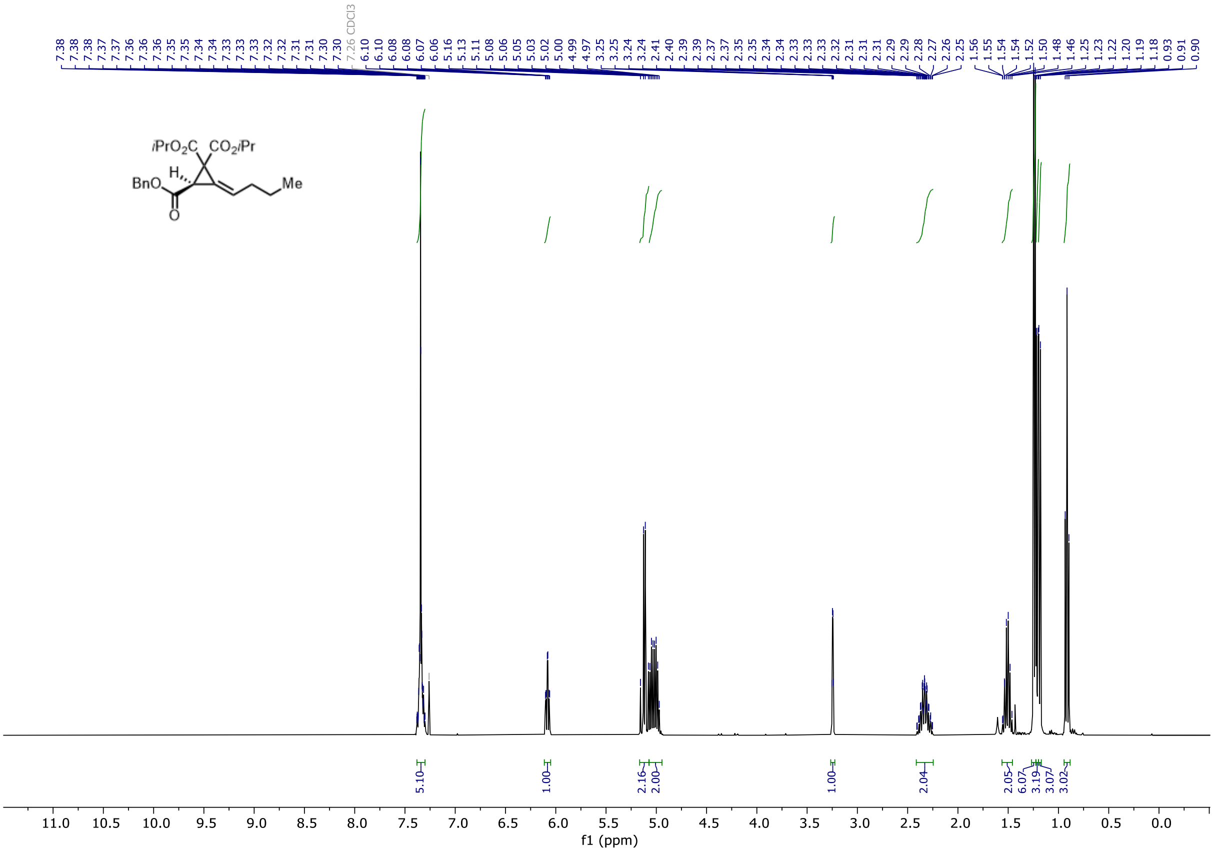

^{13}C NMR (101 MHz, $CDCl_3$) of **19b**

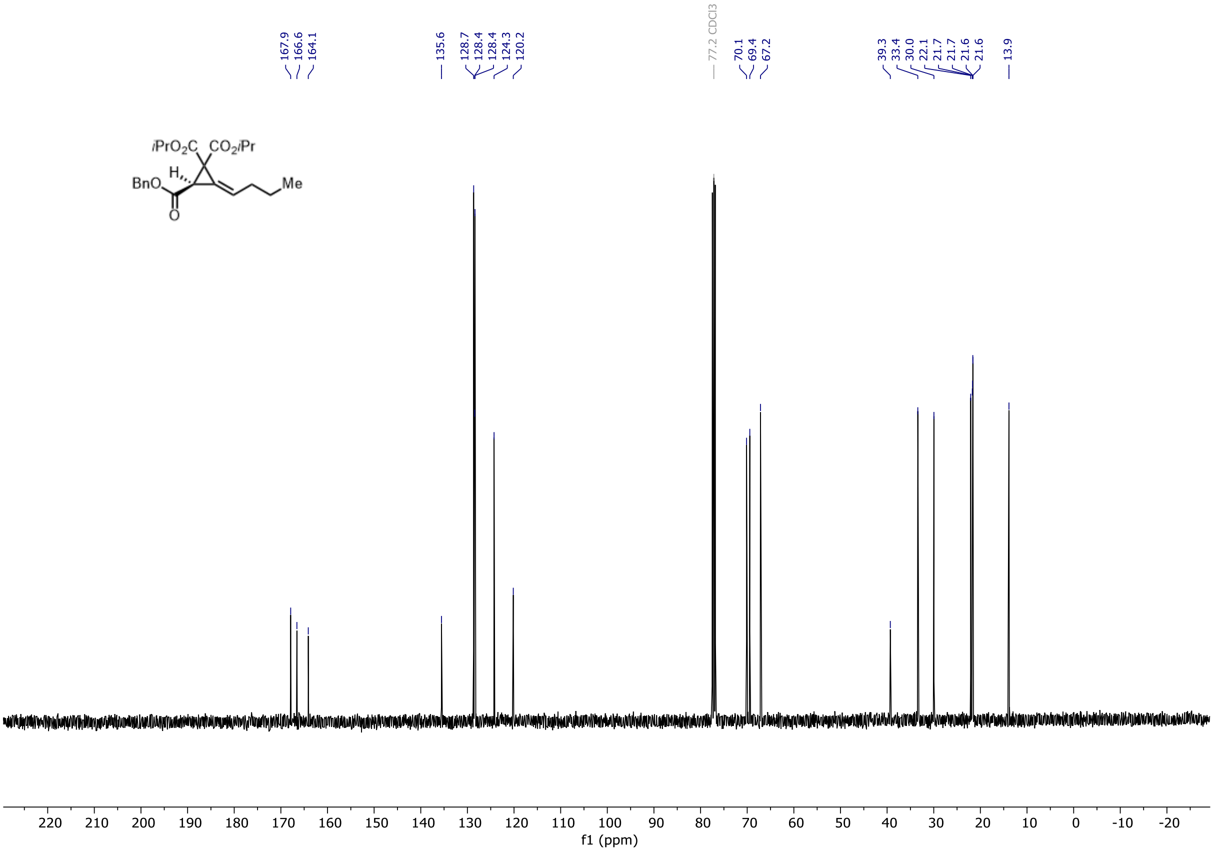

^{1}**H NMR** (400 MHz, $CDCl_3$) of **20b**

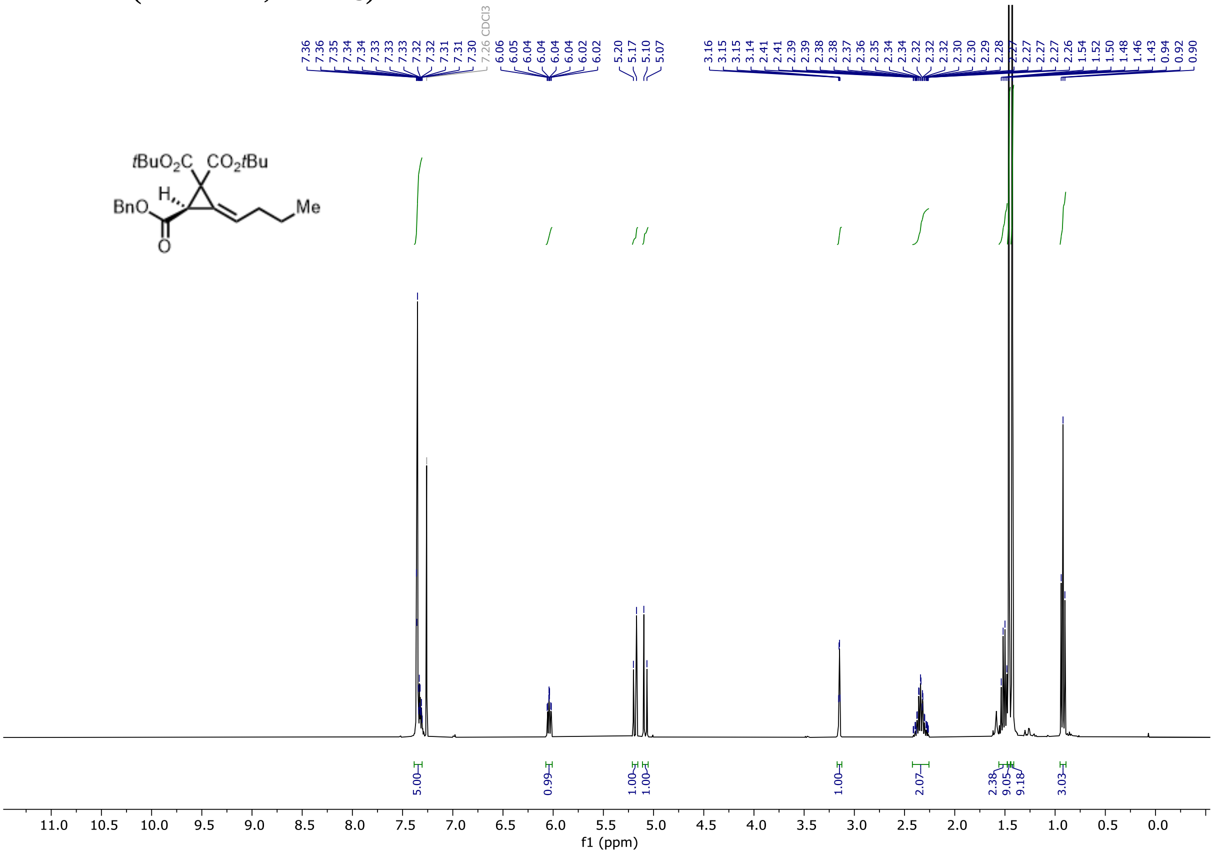

^{13}**C NMR** (101 MHz, $CDCl_3$) of **20b**

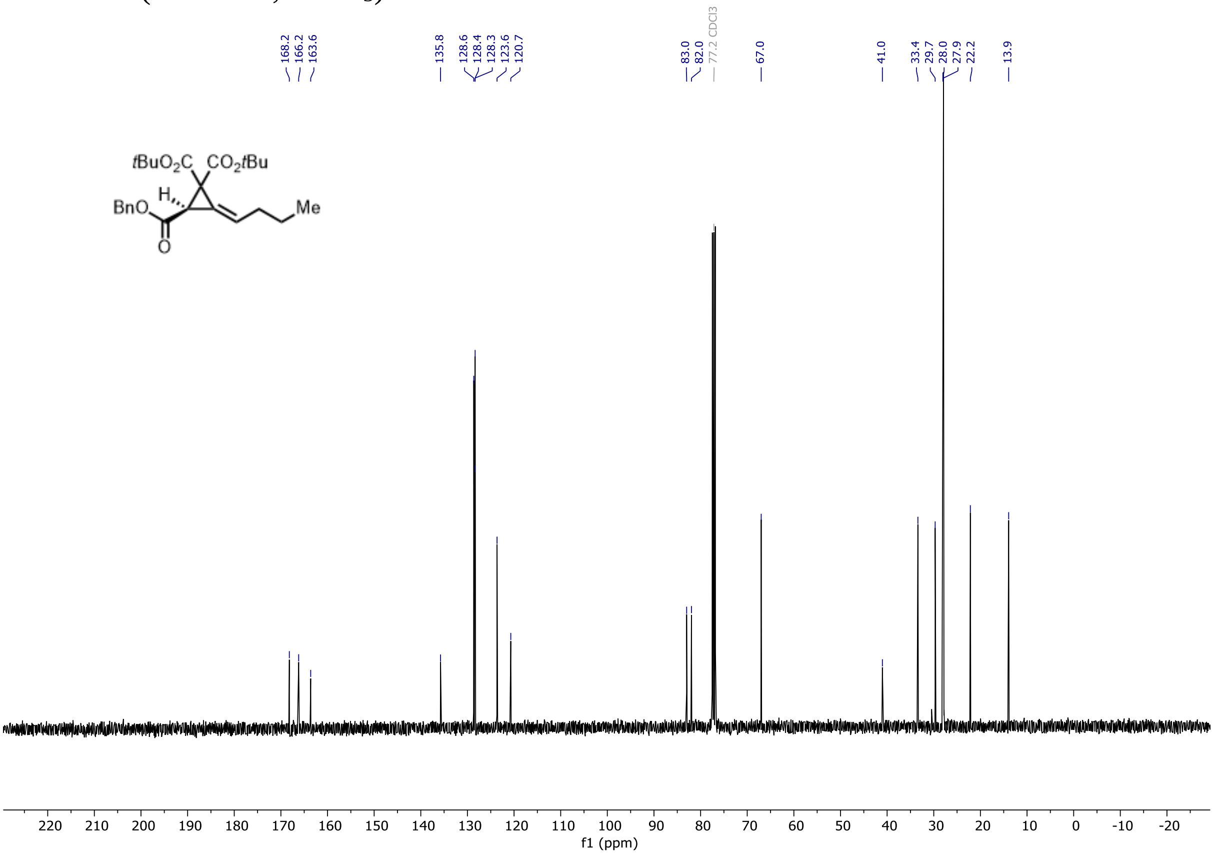

^{1}H NMR (400 MHz, $CDCl_3$) of **21b**

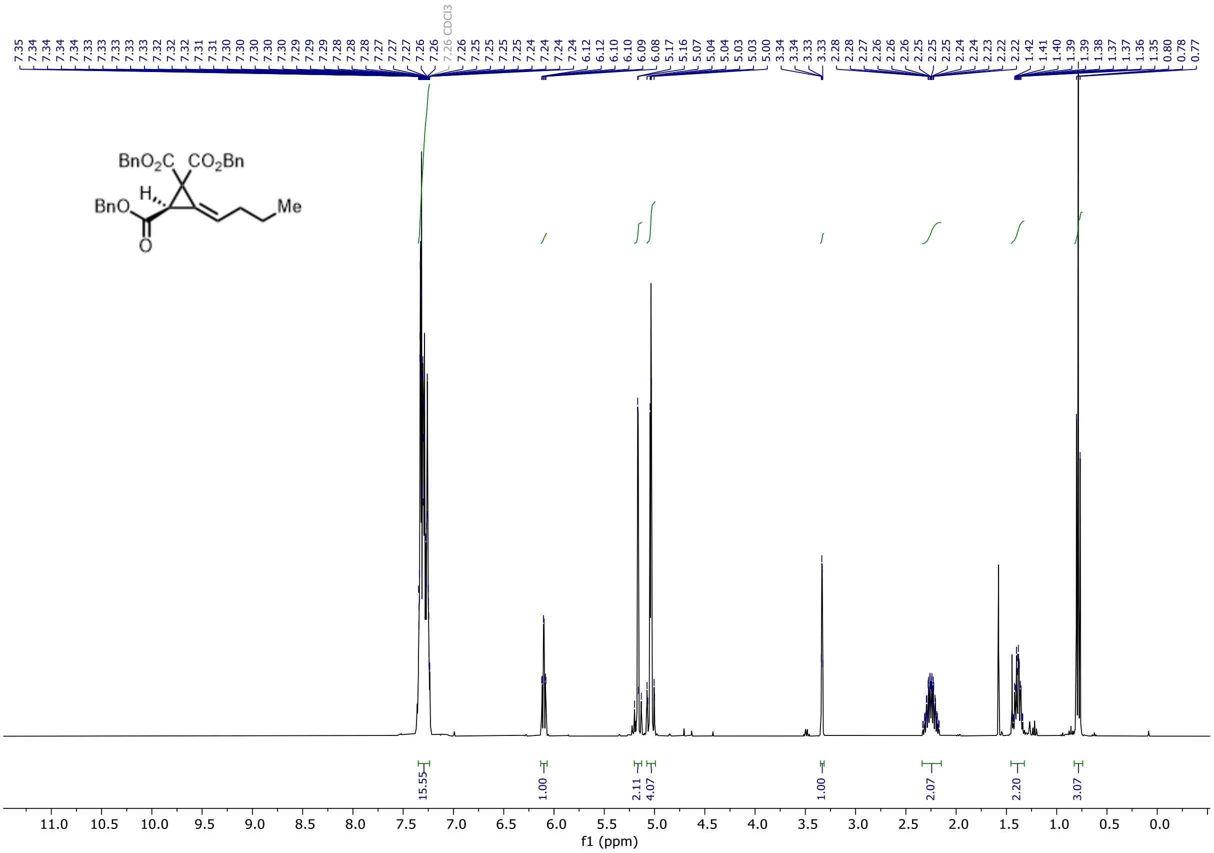

^{13}C NMR (101 MHz, $CDCl_3$) of **21b**

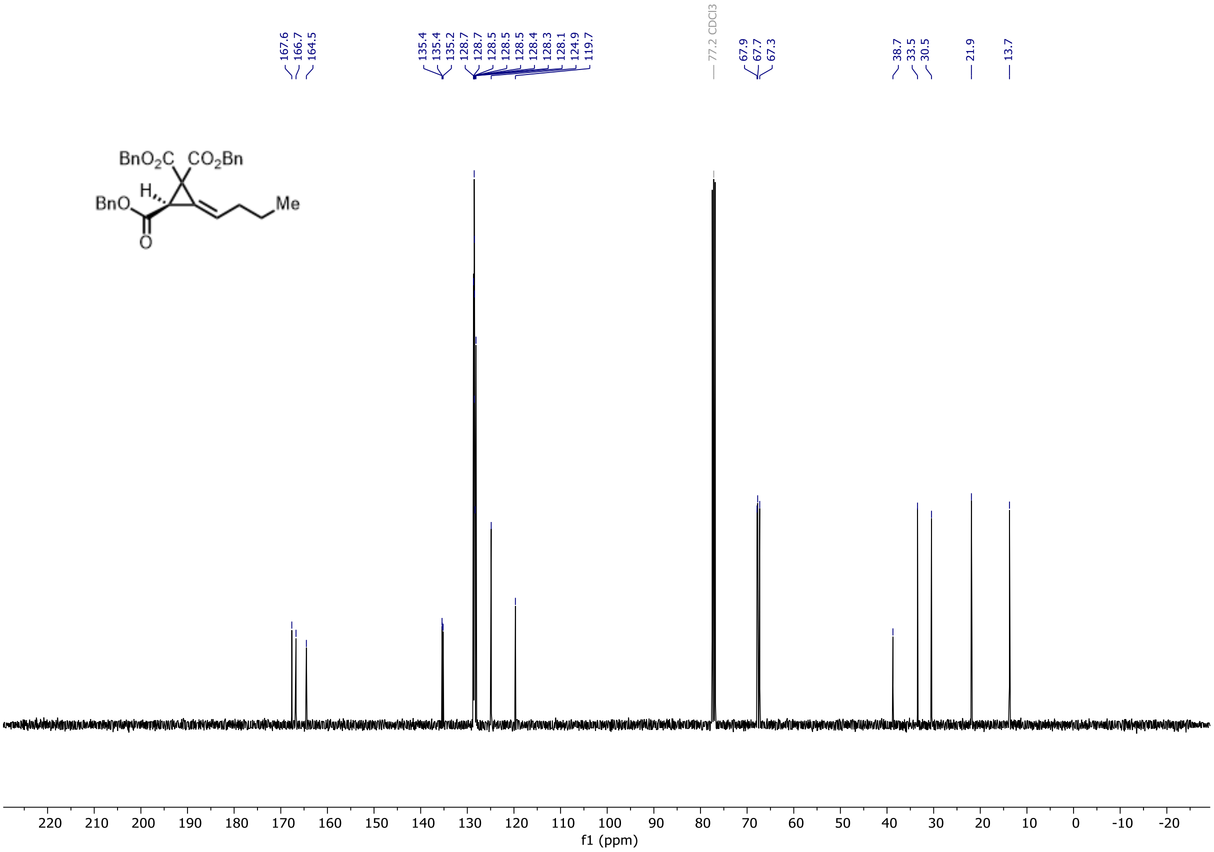

^{1}H NMR (400 MHz, $CDCl_3$) of **22b**

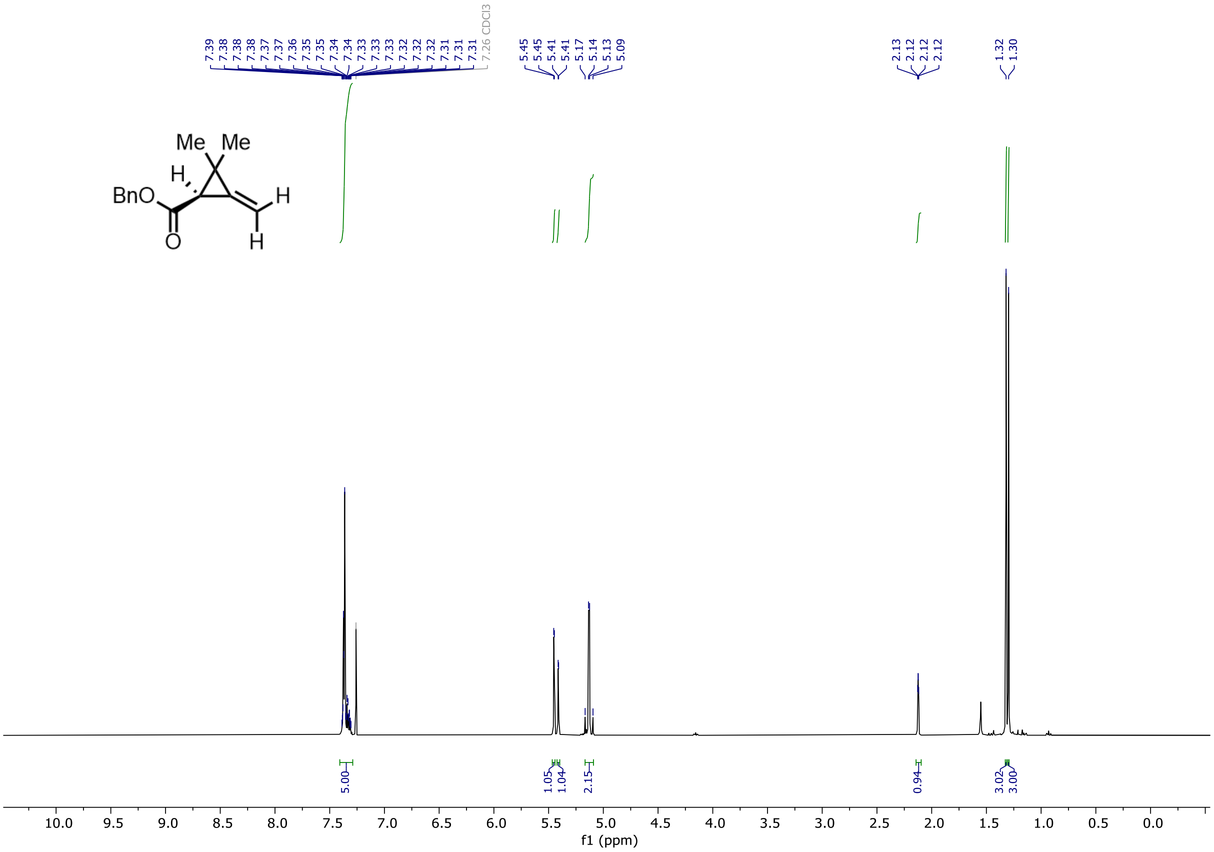

^{13}C NMR (101 MHz, $CDCl_3$) of **22b**

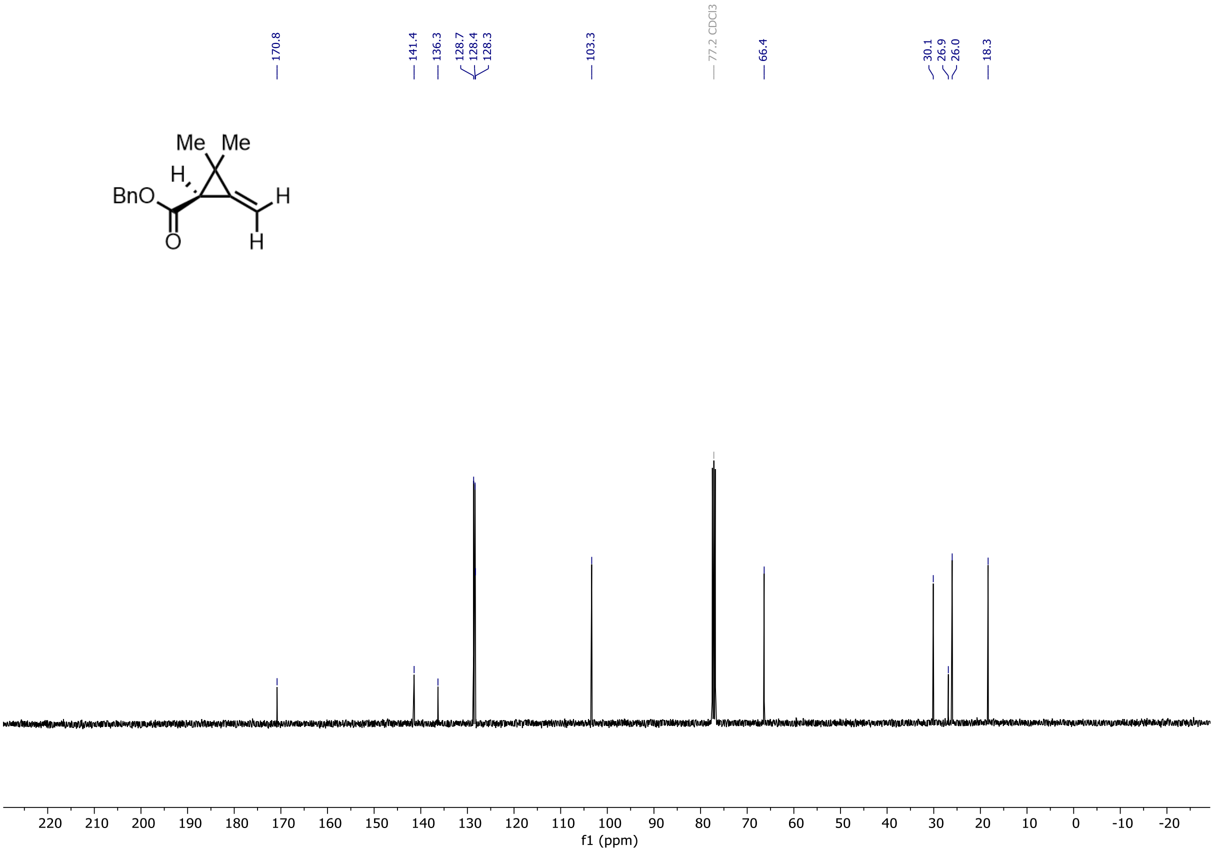

^{1}H NMR (500 MHz, $CDCl_3$) of **23b**

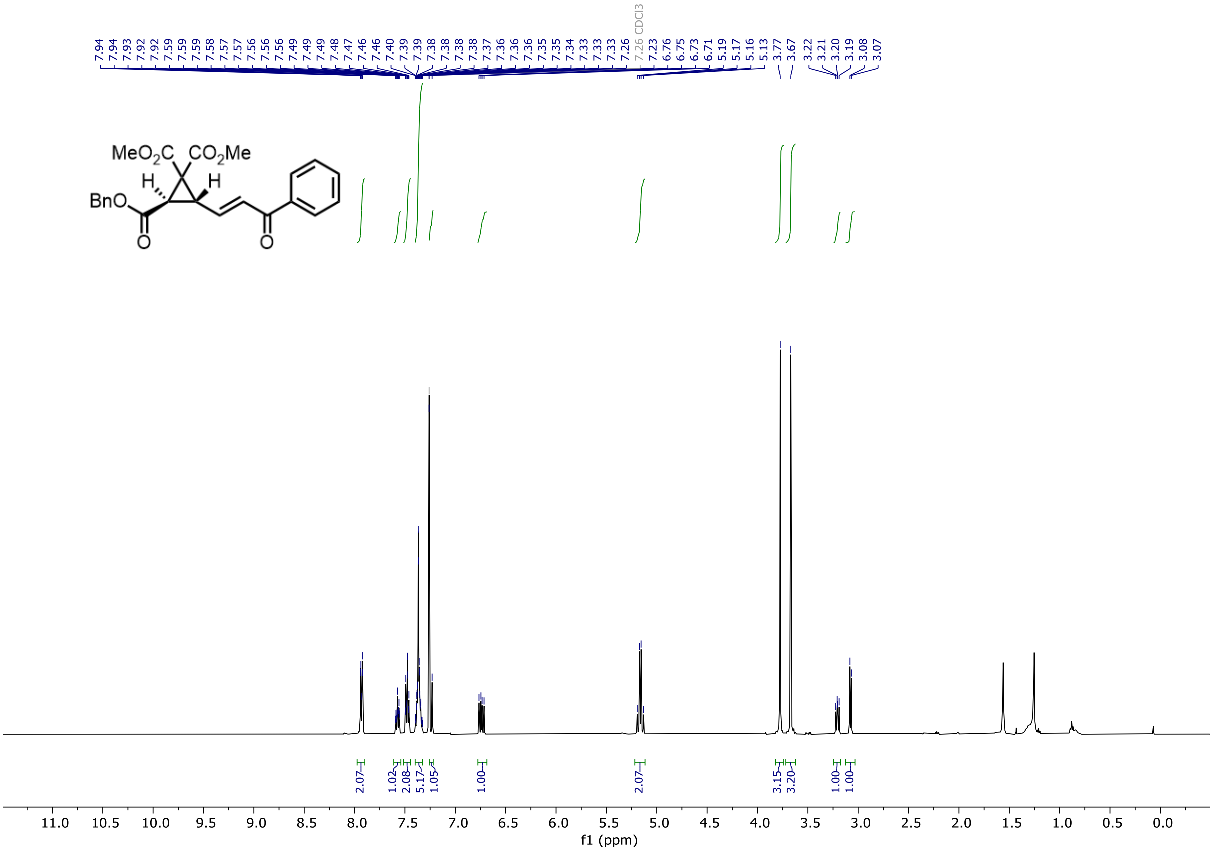

^{13}C NMR (126 MHz, $CDCl_3$) of **23b**

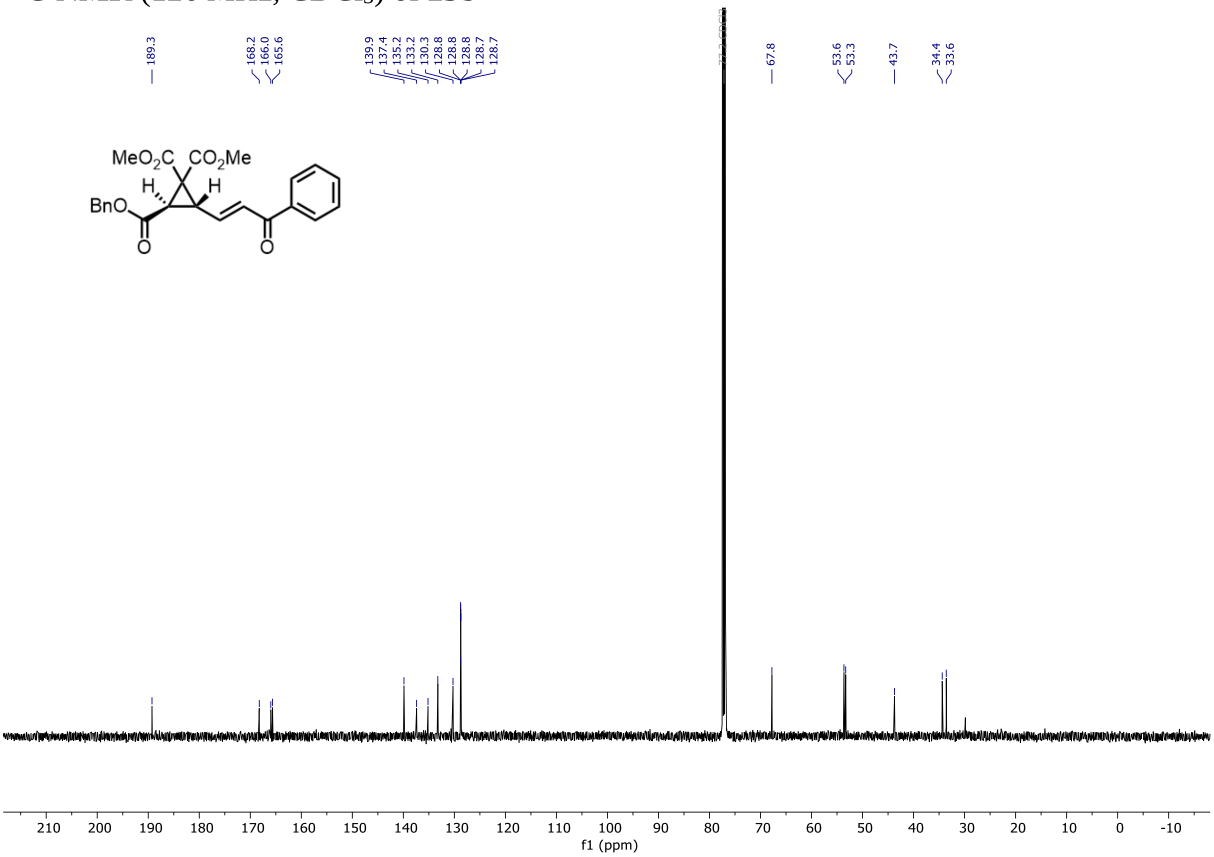

^{1}H NMR (400 MHz, $CDCl_3$) of **24b**

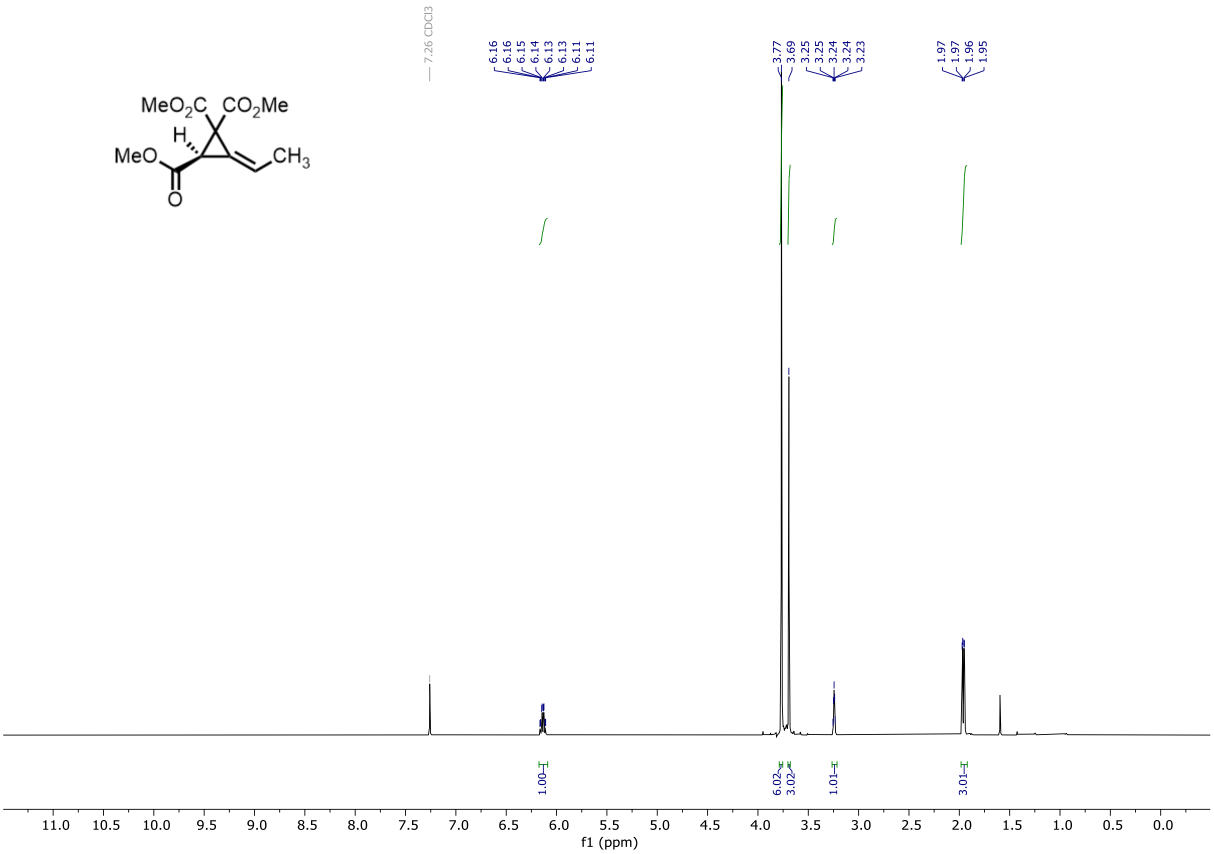

^{13}C NMR (101 MHz, $CDCl_3$) of **24b**

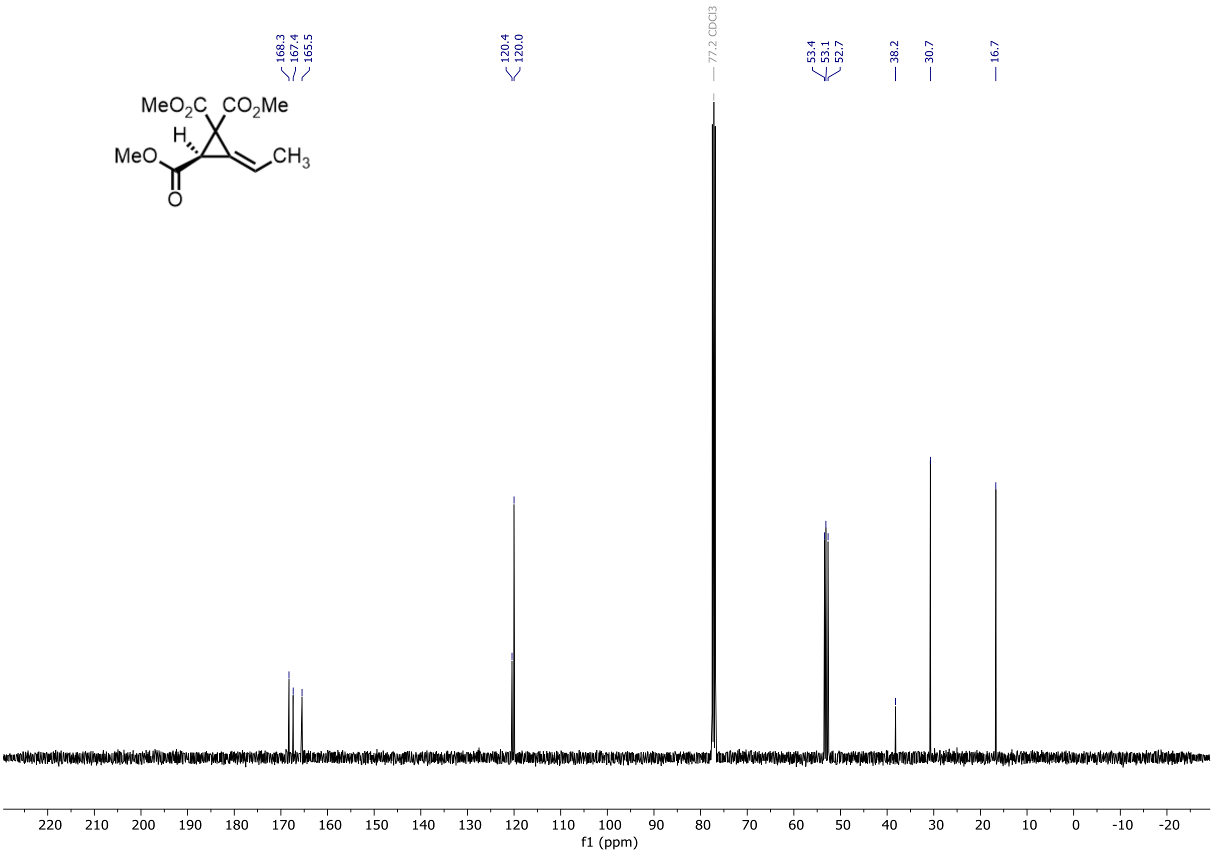

^{1}H NMR (500 MHz, $CDCl_3$) of **25b**

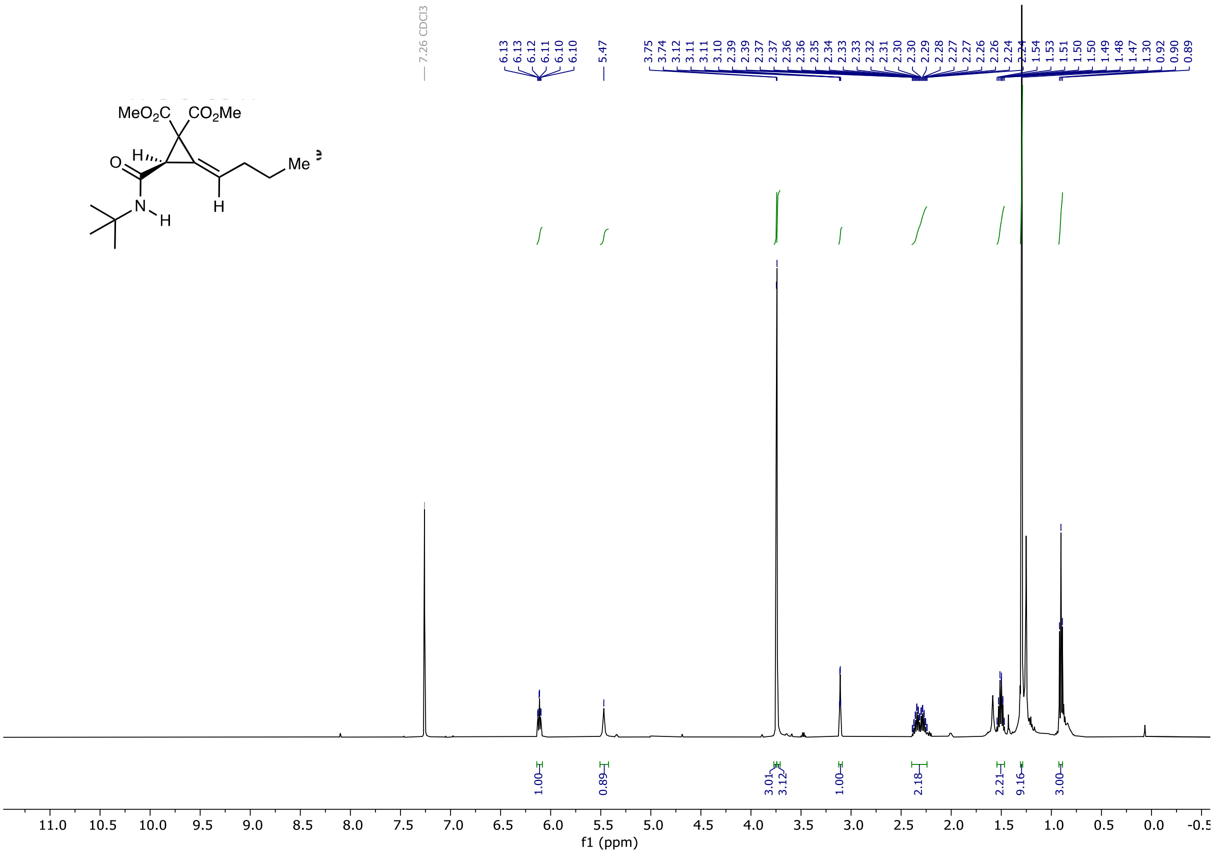

^{13}C NMR (126 MHz, $CDCl_3$) of **25b**

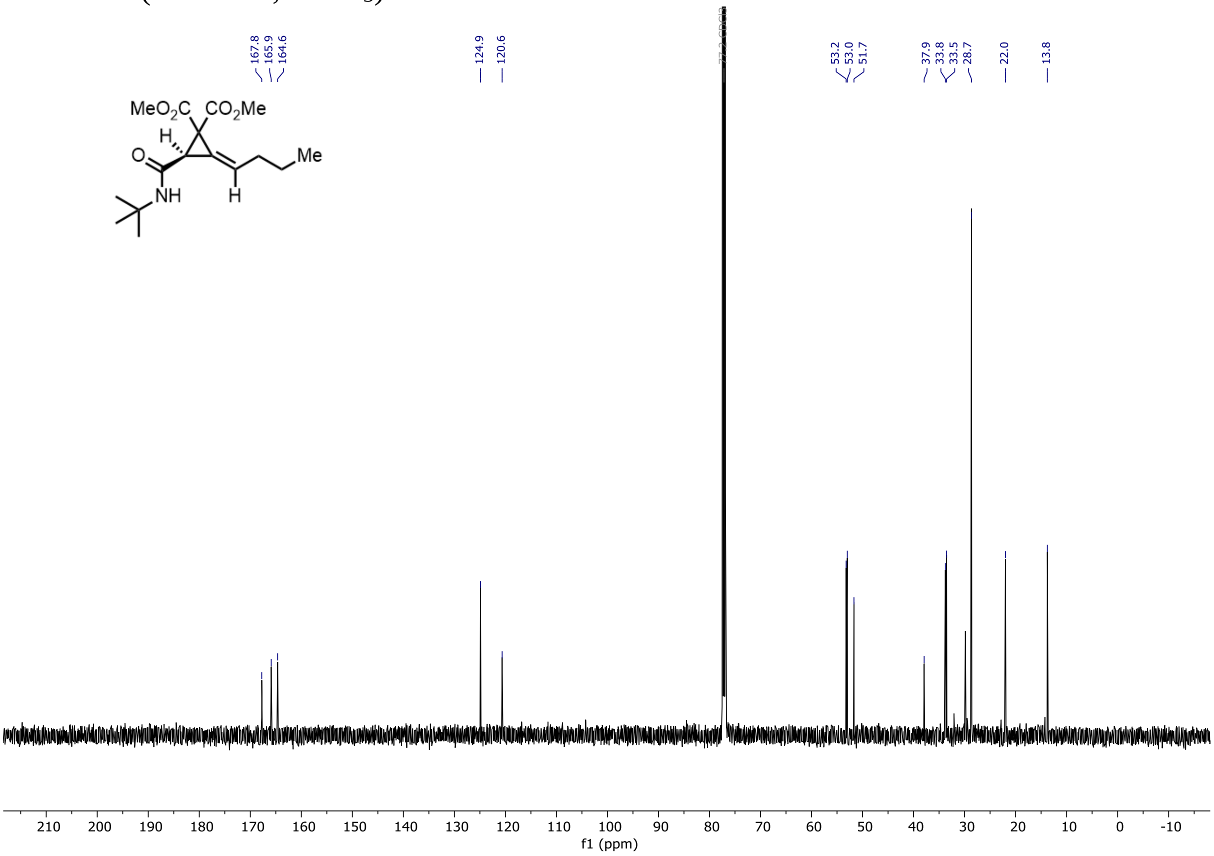

^{1}**H NMR** (500 MHz, $CDCl_3$) of **26b**

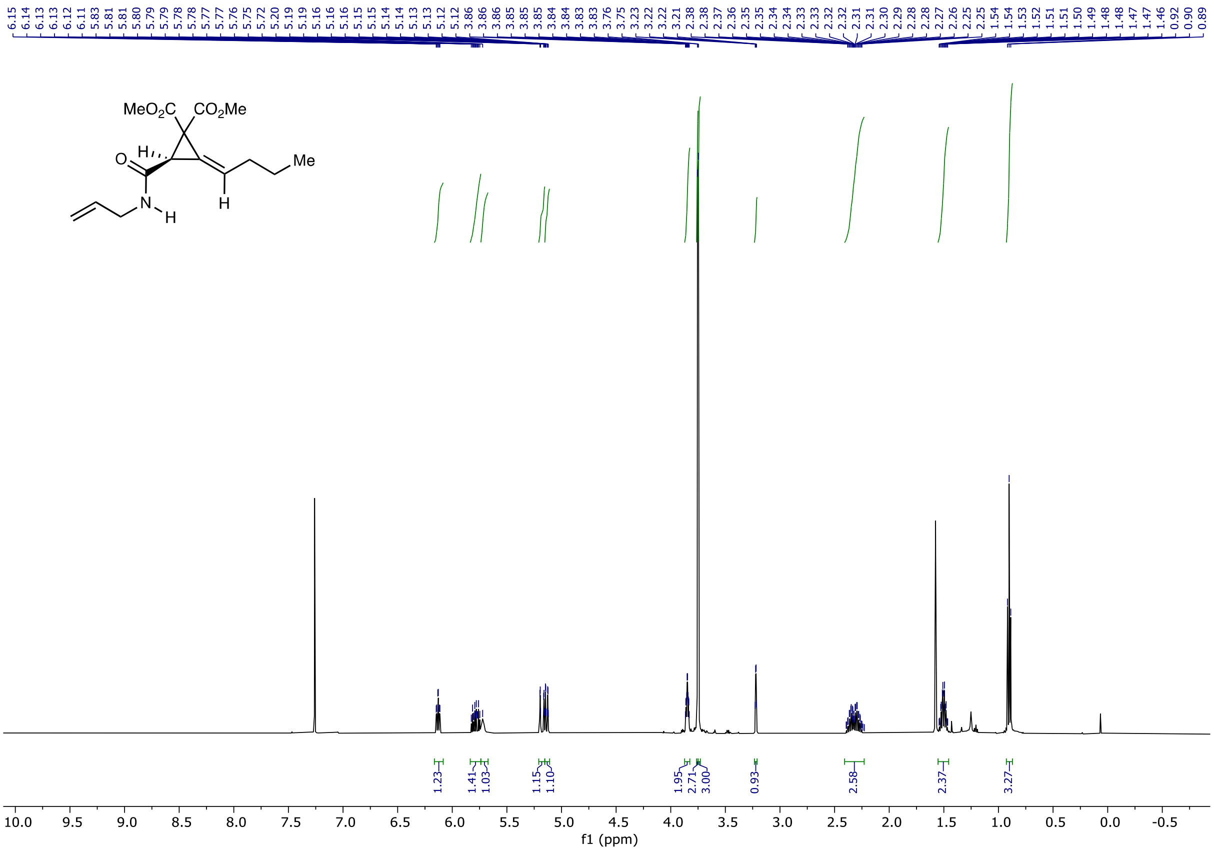

^{13}**C NMR** (126 MHz, $CDCl_3$) of **26b**

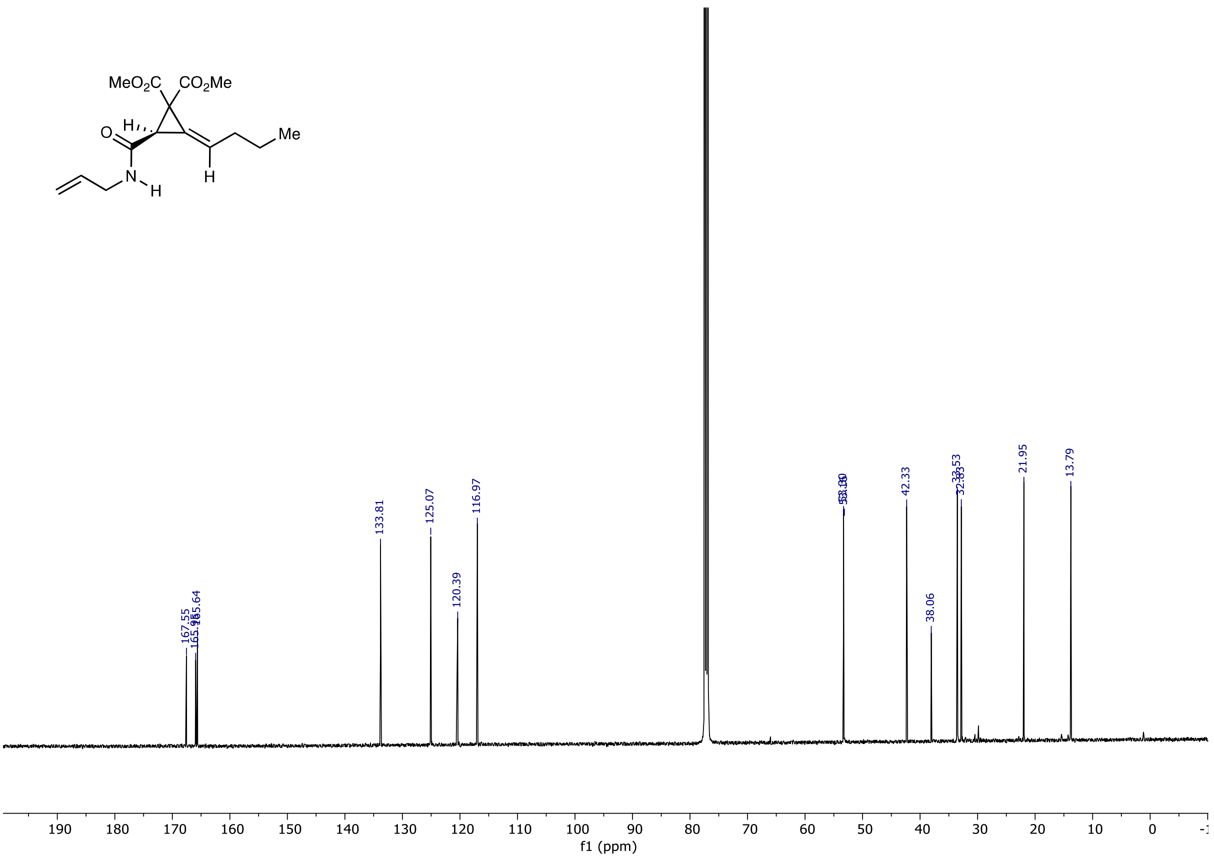

^{1}H NMR (500 MHz, $CDCl_3$) of **27b**

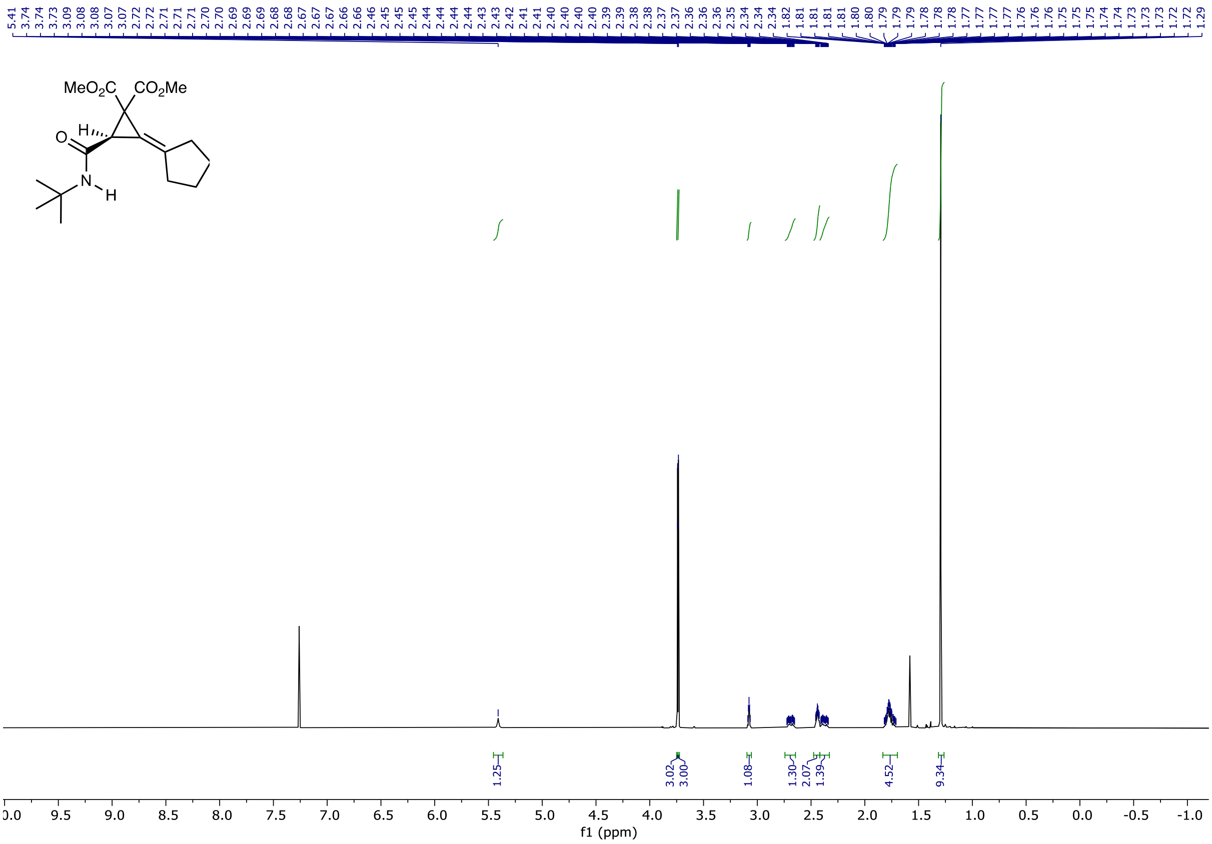

^{13}C NMR (126 MHz, $CDCl_3$) of **27b**

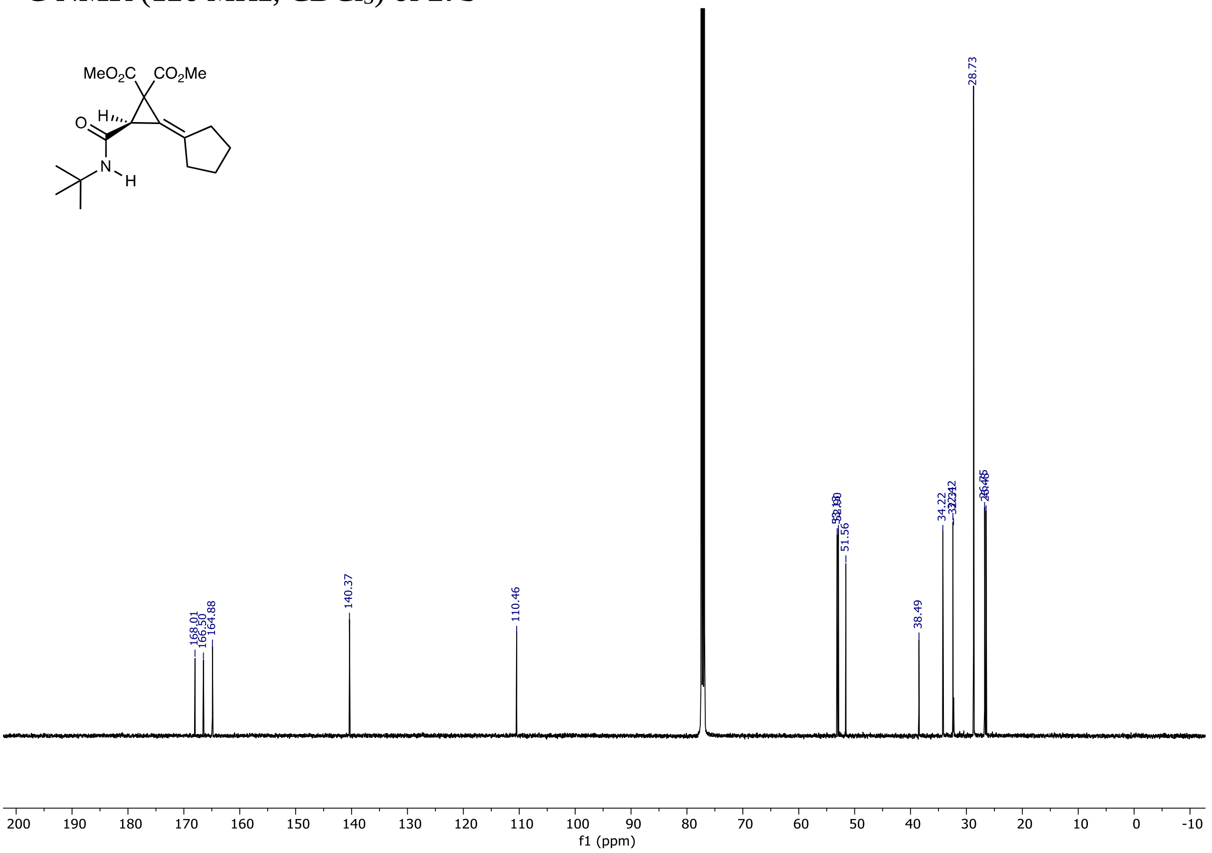

^{1}H NMR (500 MHz, $CDCl_3$) of **28b**

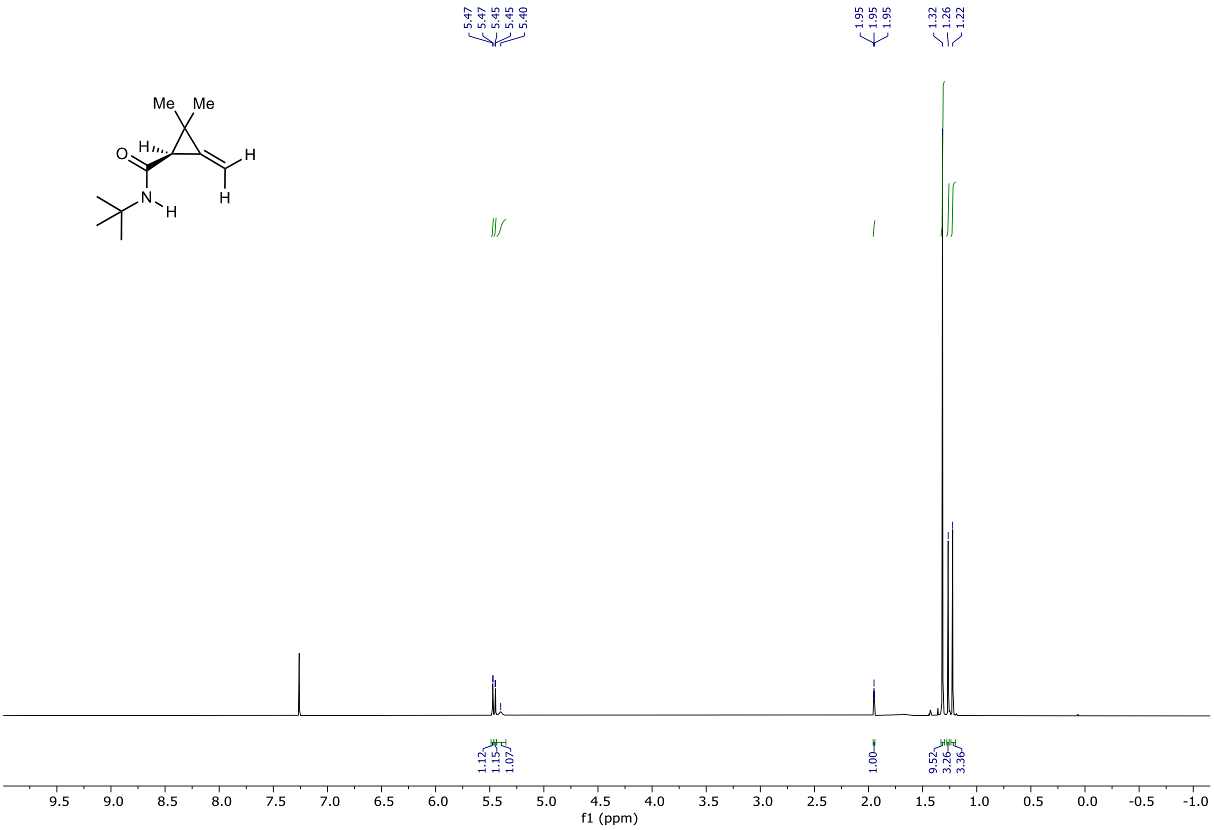

^{13}C NMR (126 MHz, $CDCl_3$) of **28b**

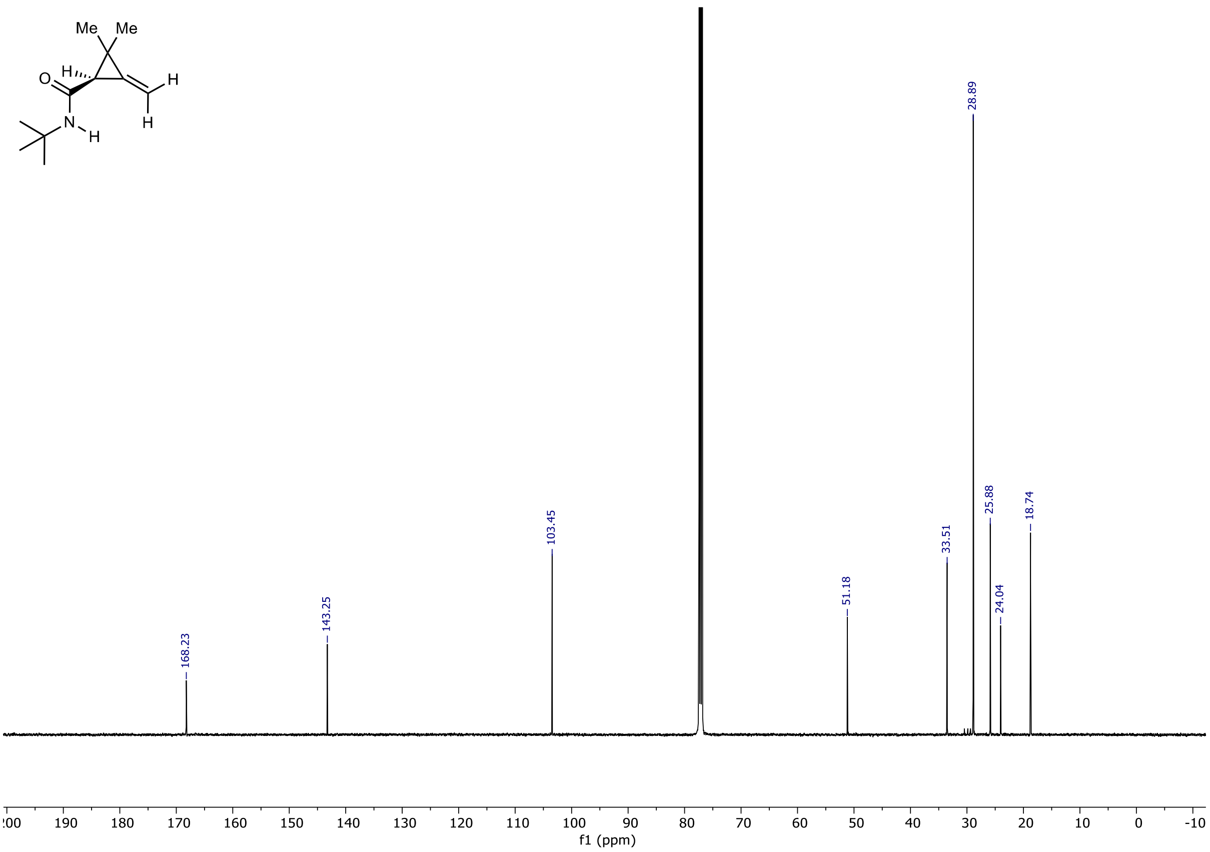

^{1}H NMR (600 MHz, $CDCl_3$) of **29b**

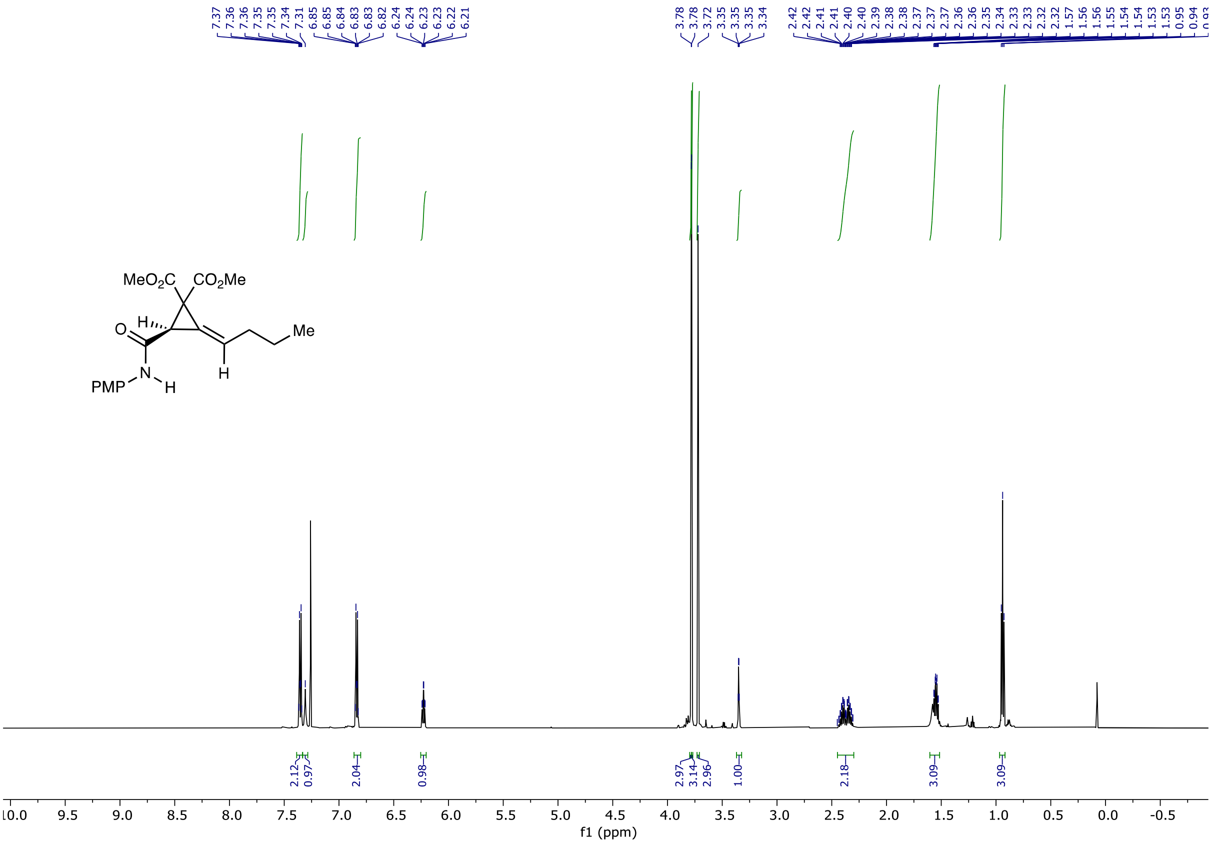

^{13}C NMR (151 MHz, $CDCl_3$) of **29b**

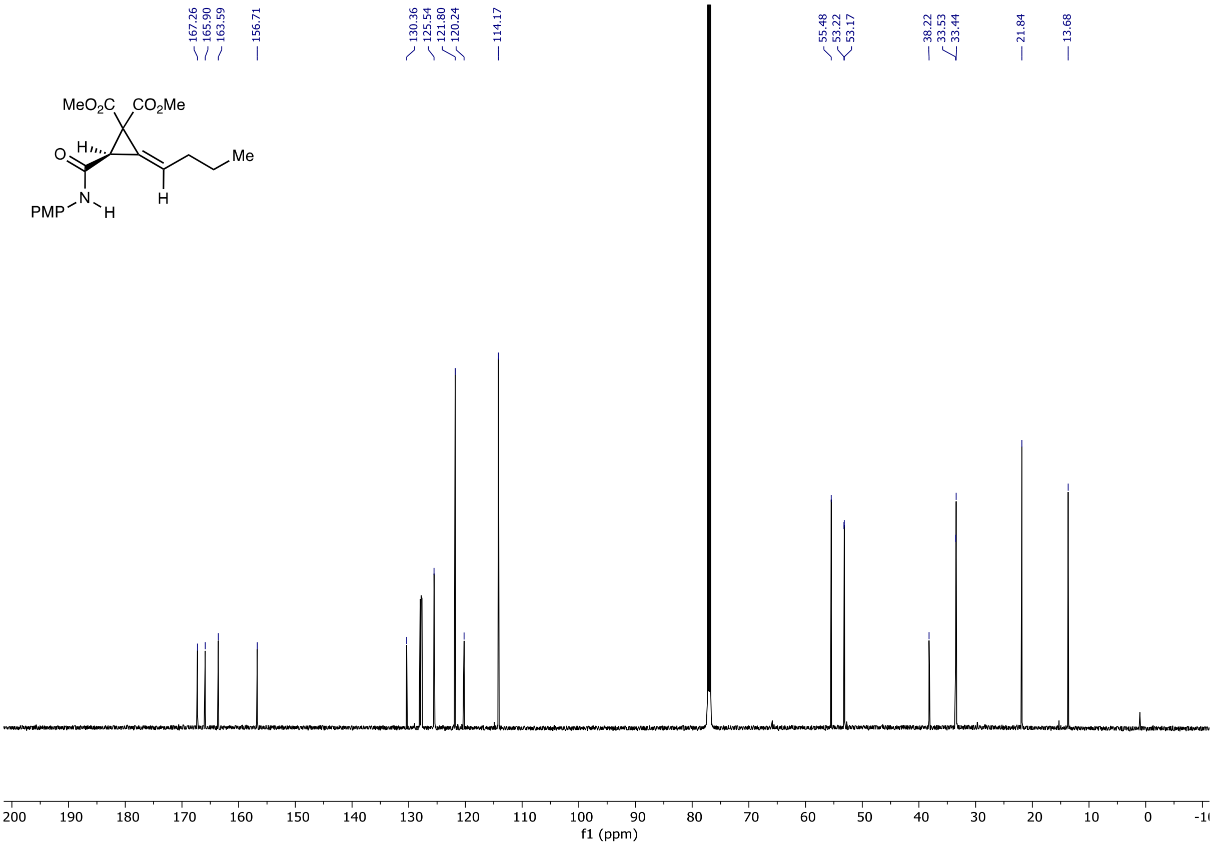

^{1}H NMR (500 MHz, $CDCl_3$) of **29c**

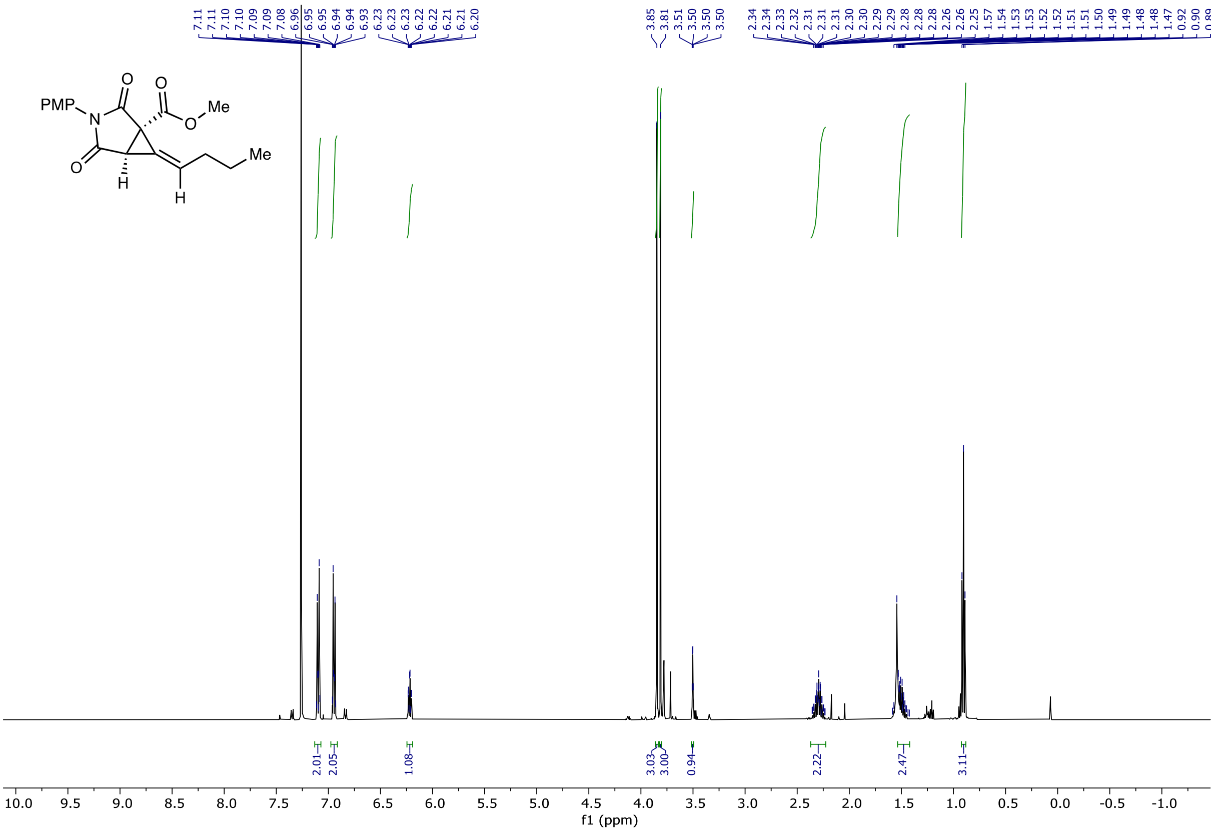

^{13}C NMR (126 MHz, $CDCl_3$) of **29c**

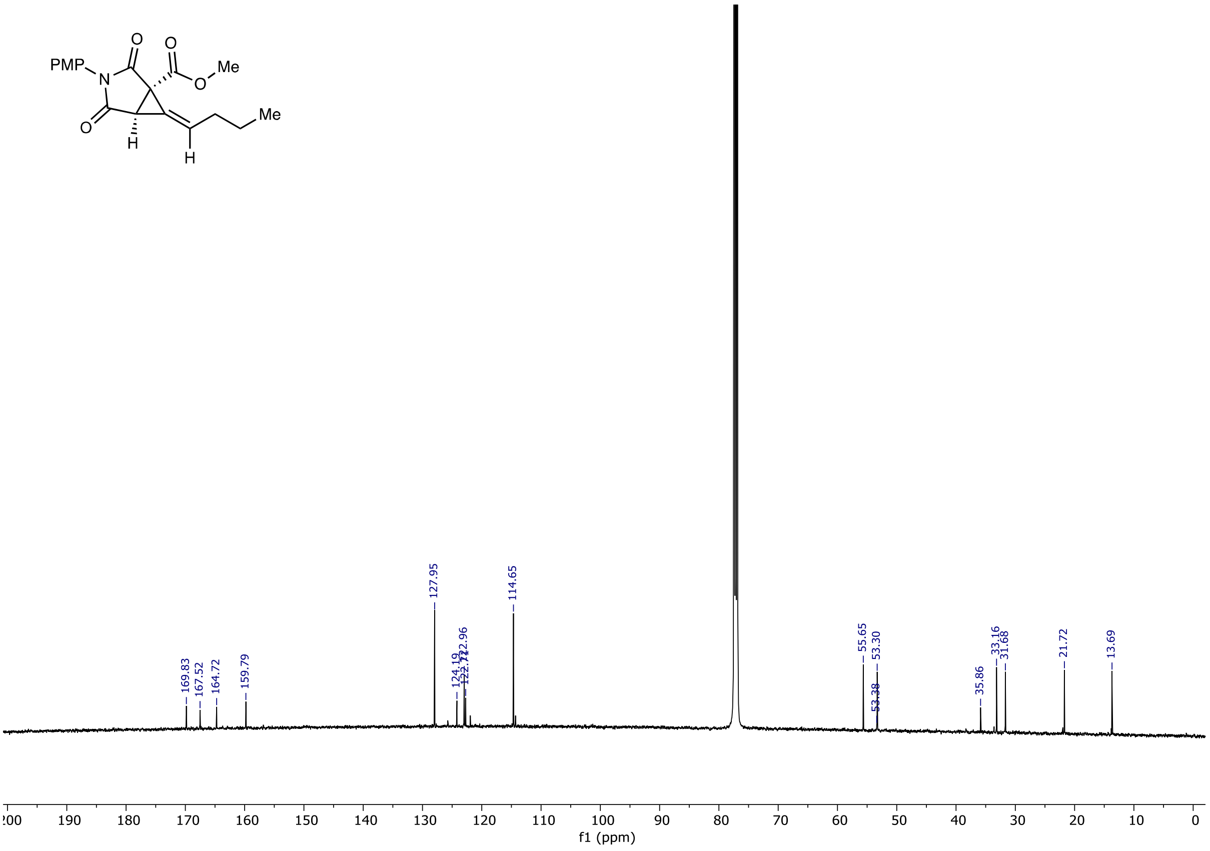

^{1}H NMR (400 MHz, $CDCl_3$) of **30b**

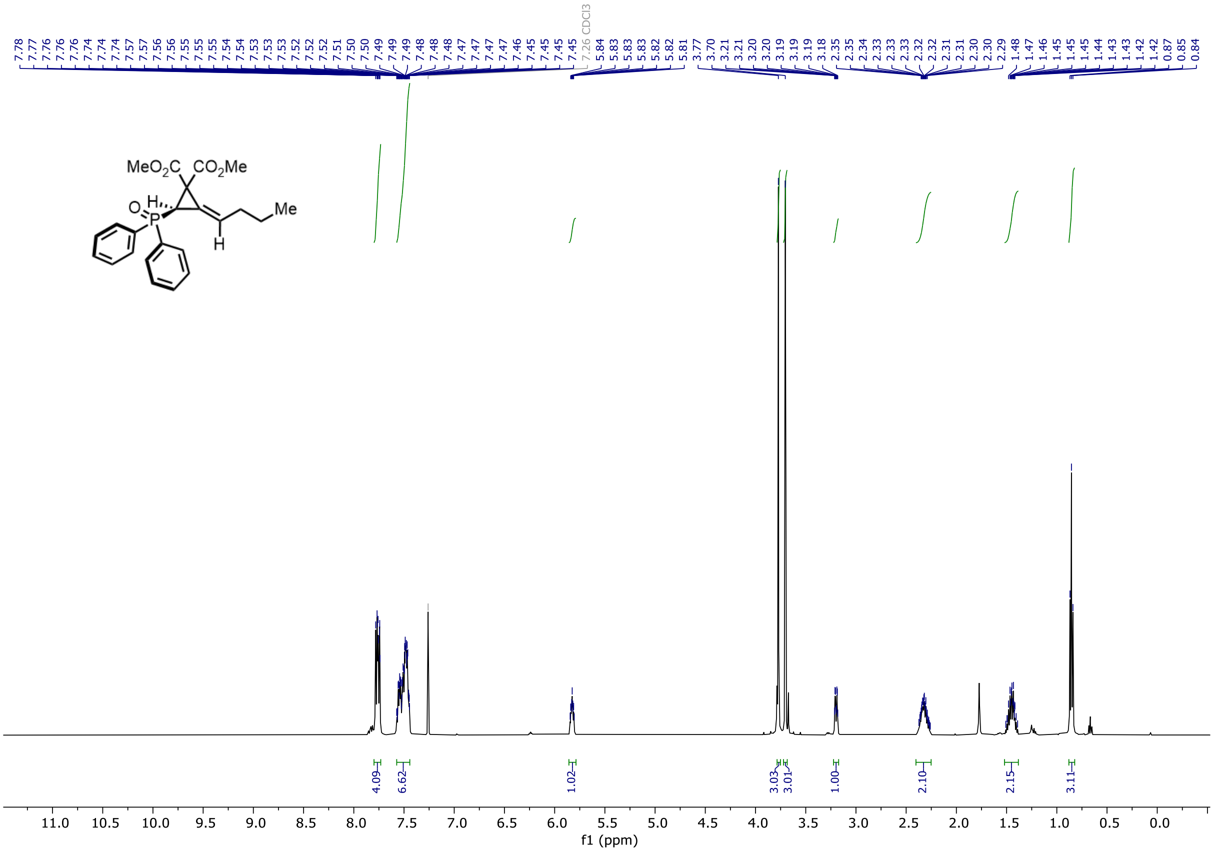

^{13}C NMR (101 MHz, $CDCl_3$) of **30b**

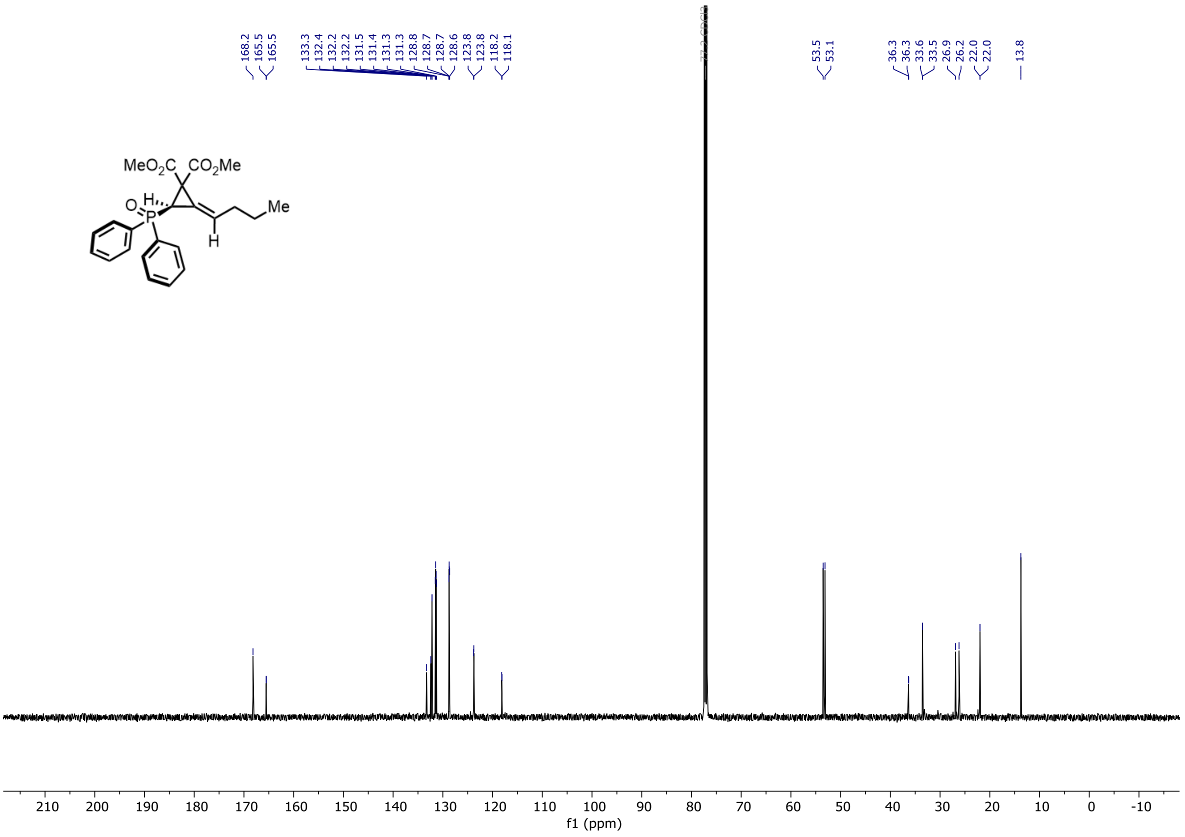

^{1}H NMR (500 MHz, $CDCl_3$) of **4b**

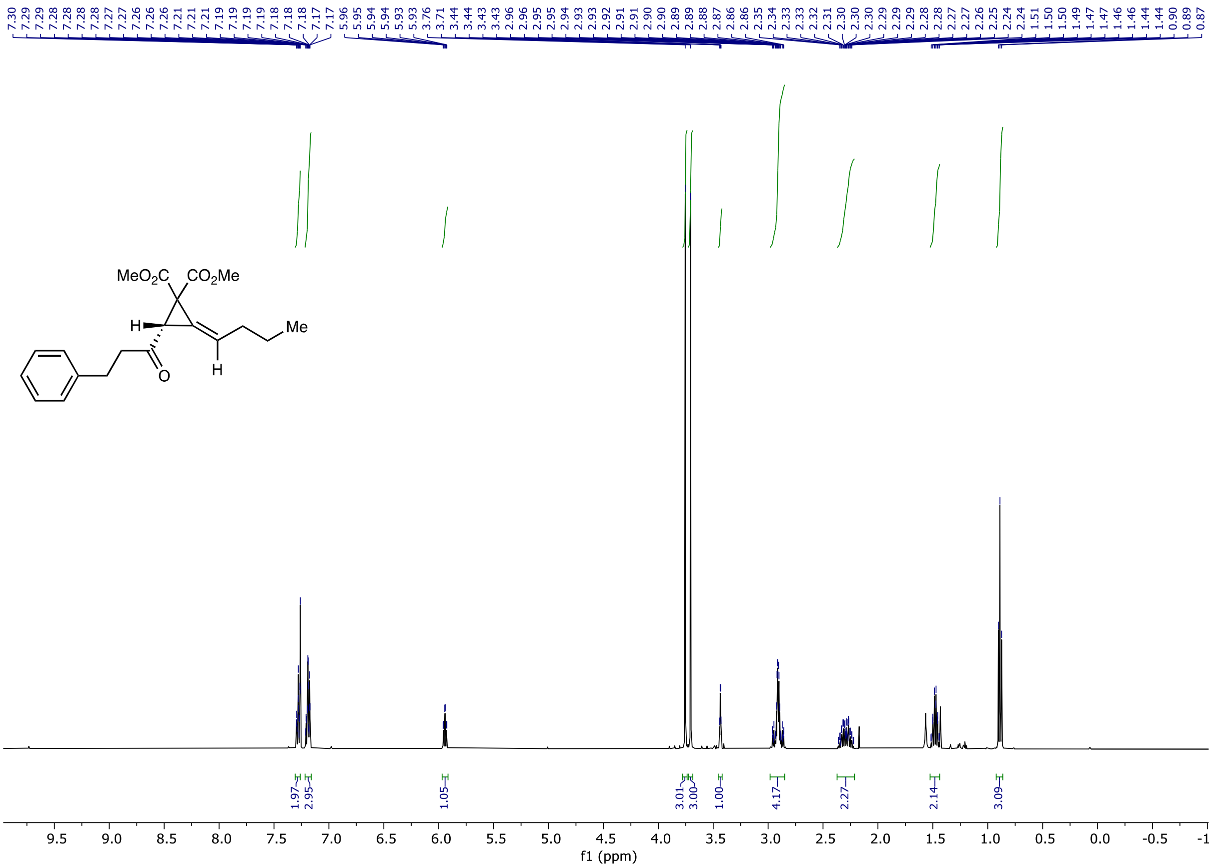

^{13}C NMR (126 MHz, $CDCl_3$) of **4b**

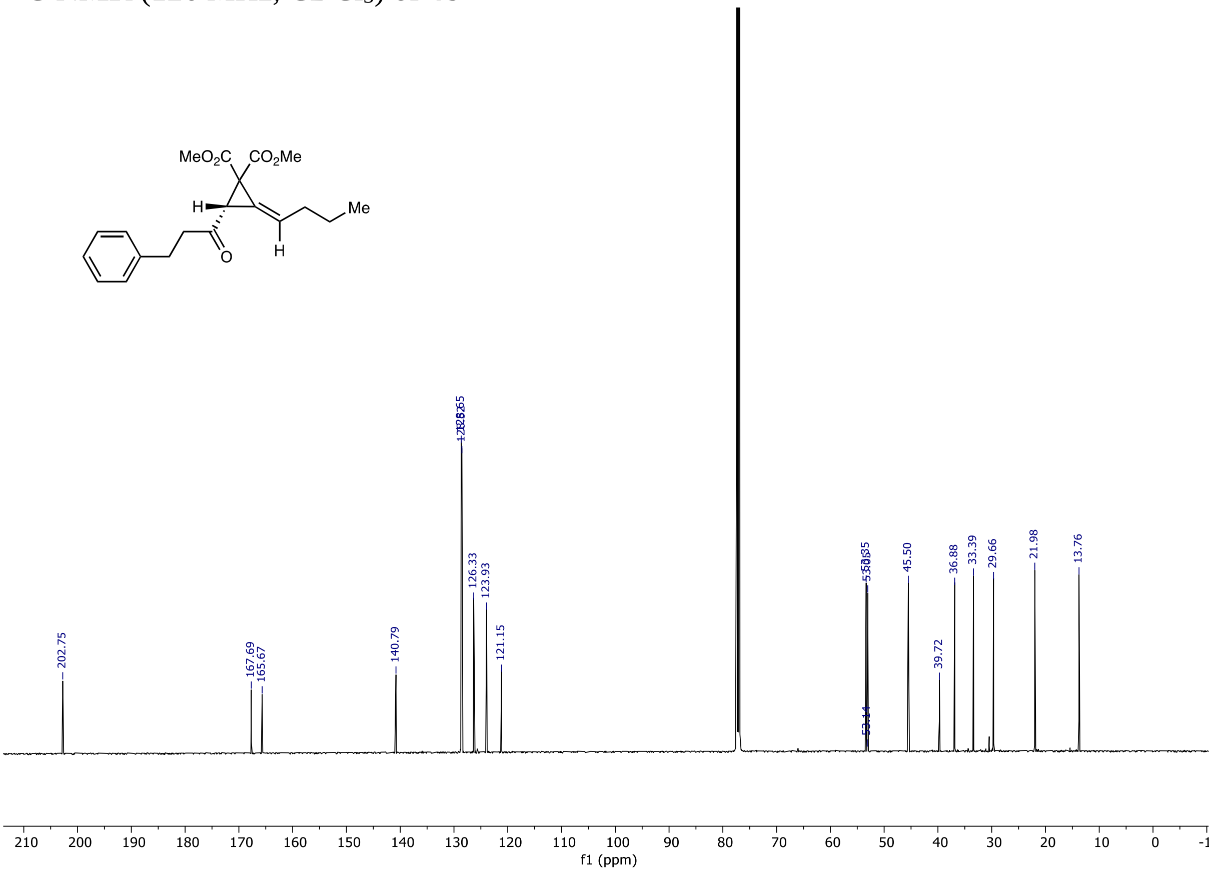

^{1}H NMR (500 MHz, Benzene-d_6) of **31b**

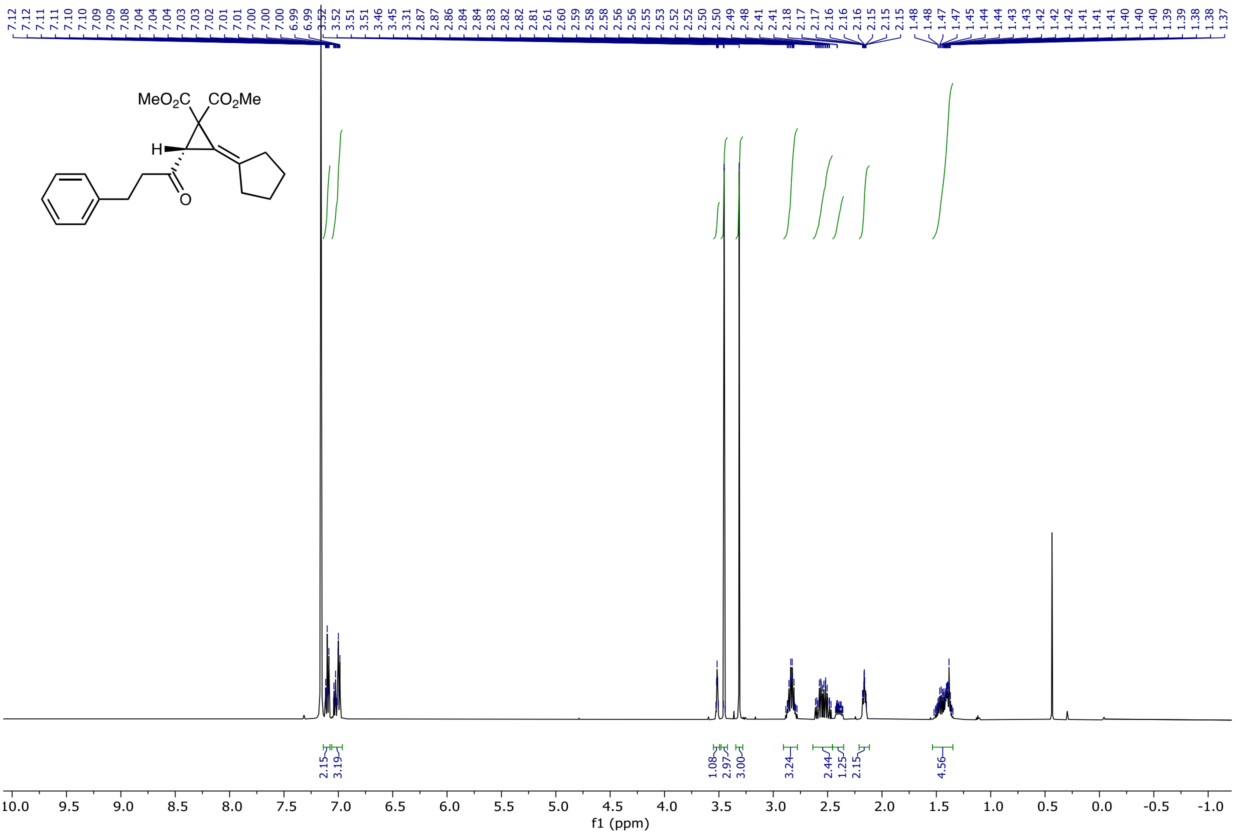

^{13}C NMR (126 MHz, Benzene-d_6) of **31b**

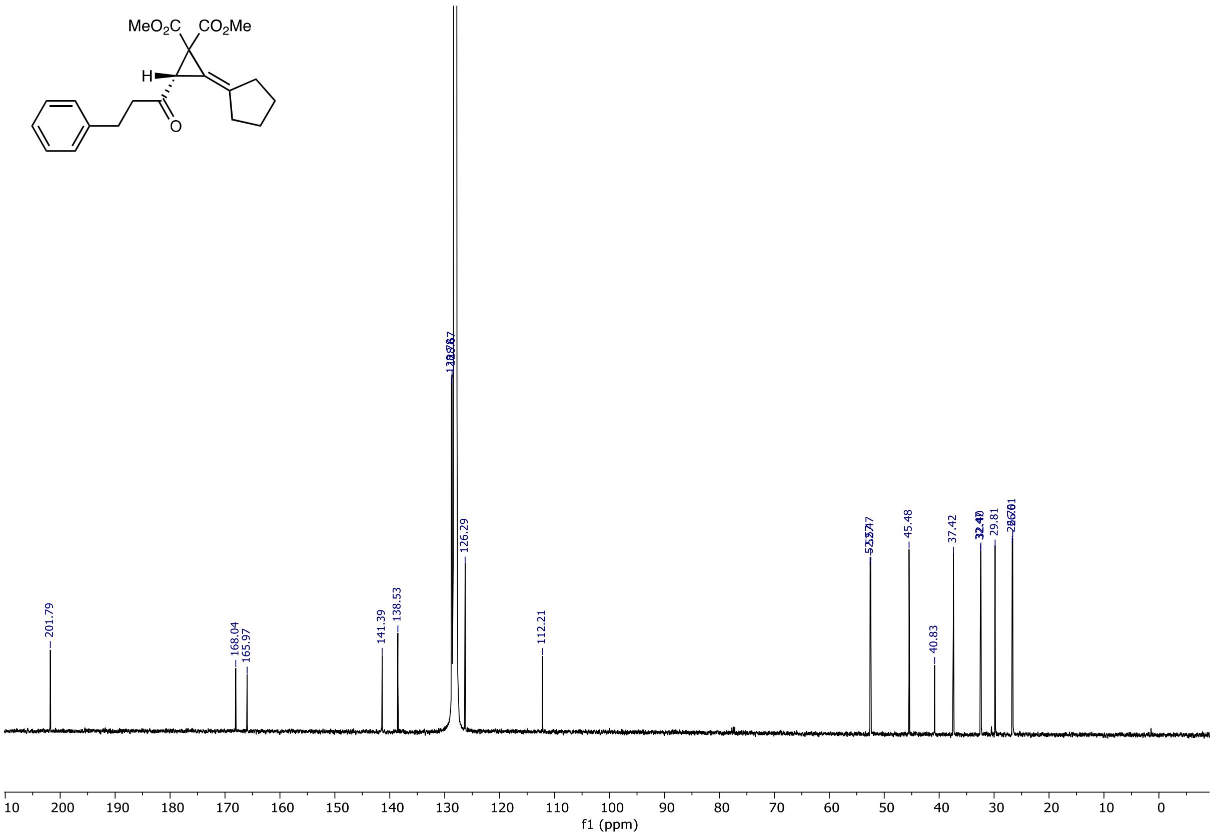

^{1}H NMR (500 MHz, $CDCl_3$) of **32b**

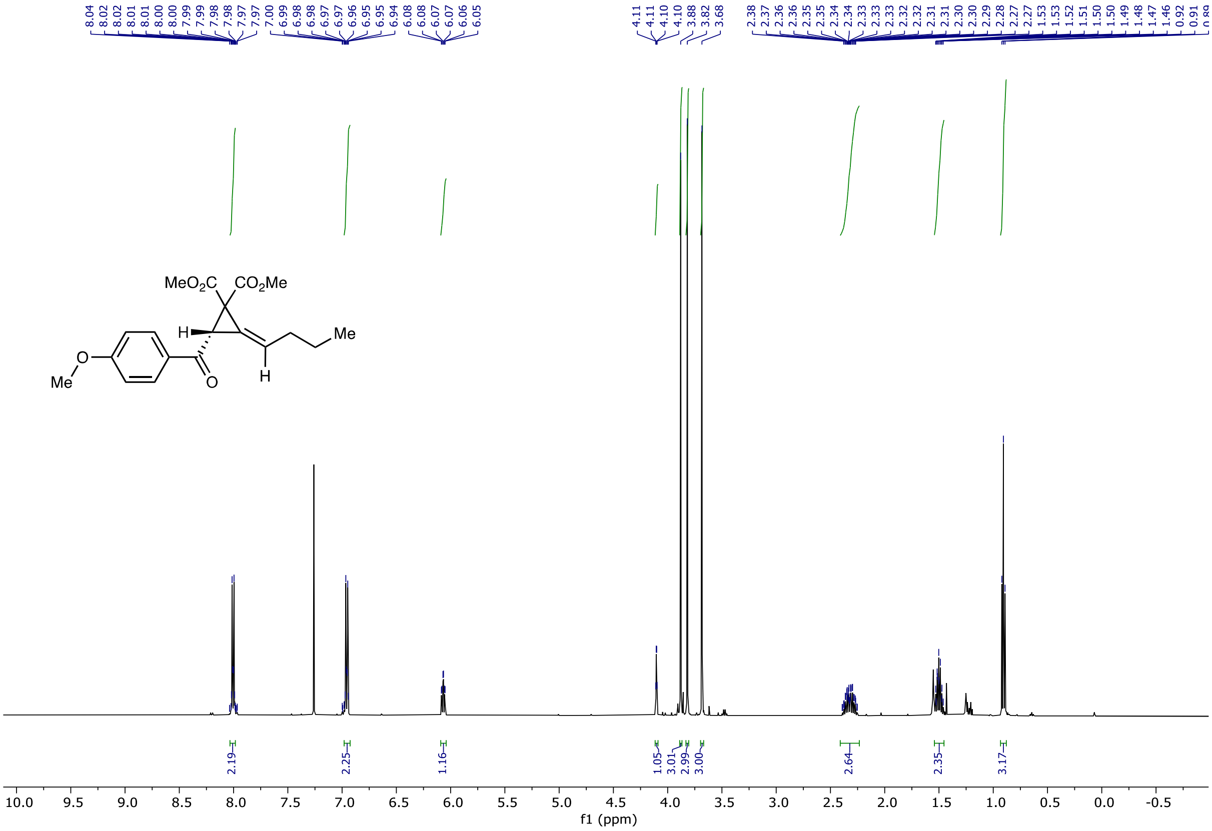

^{13}C NMR (126 MHz, $CDCl_3$) of **32b**

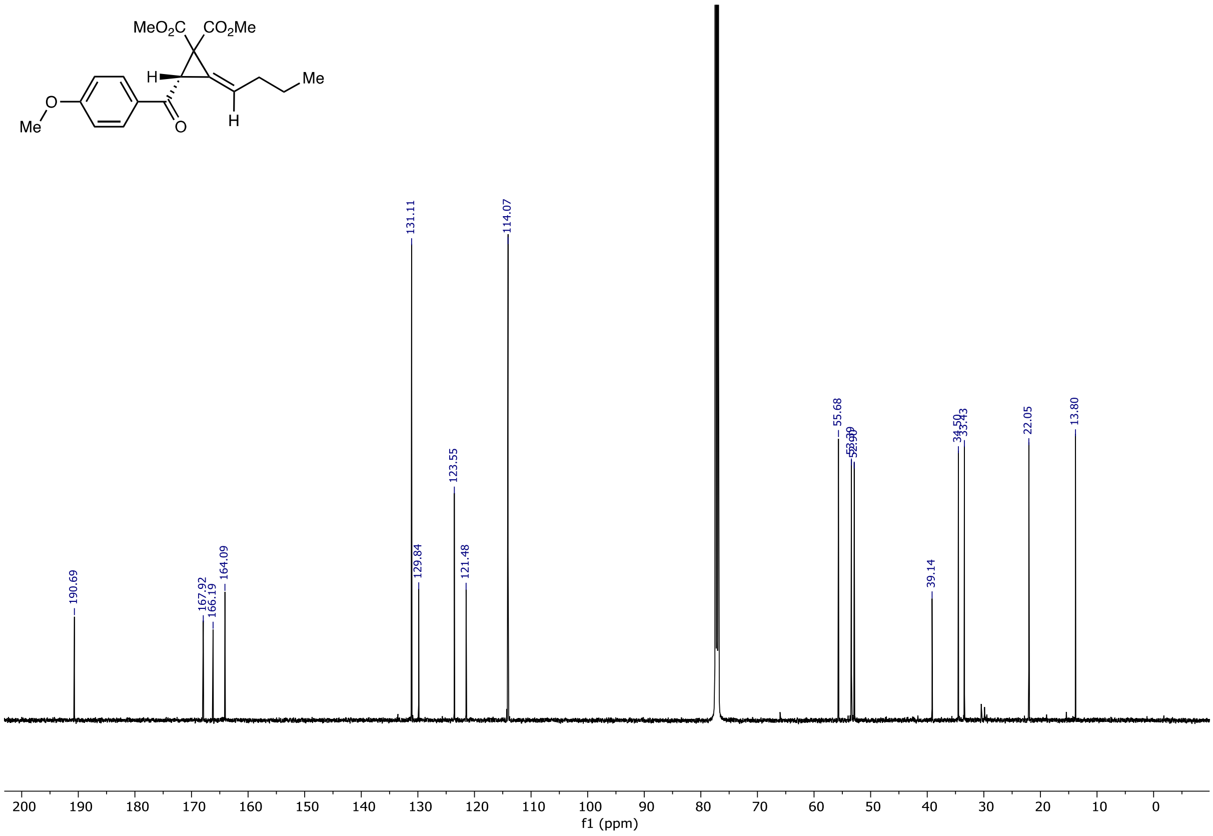

^{1}H NMR (400 MHz, Benzene-d_6) of **33b**

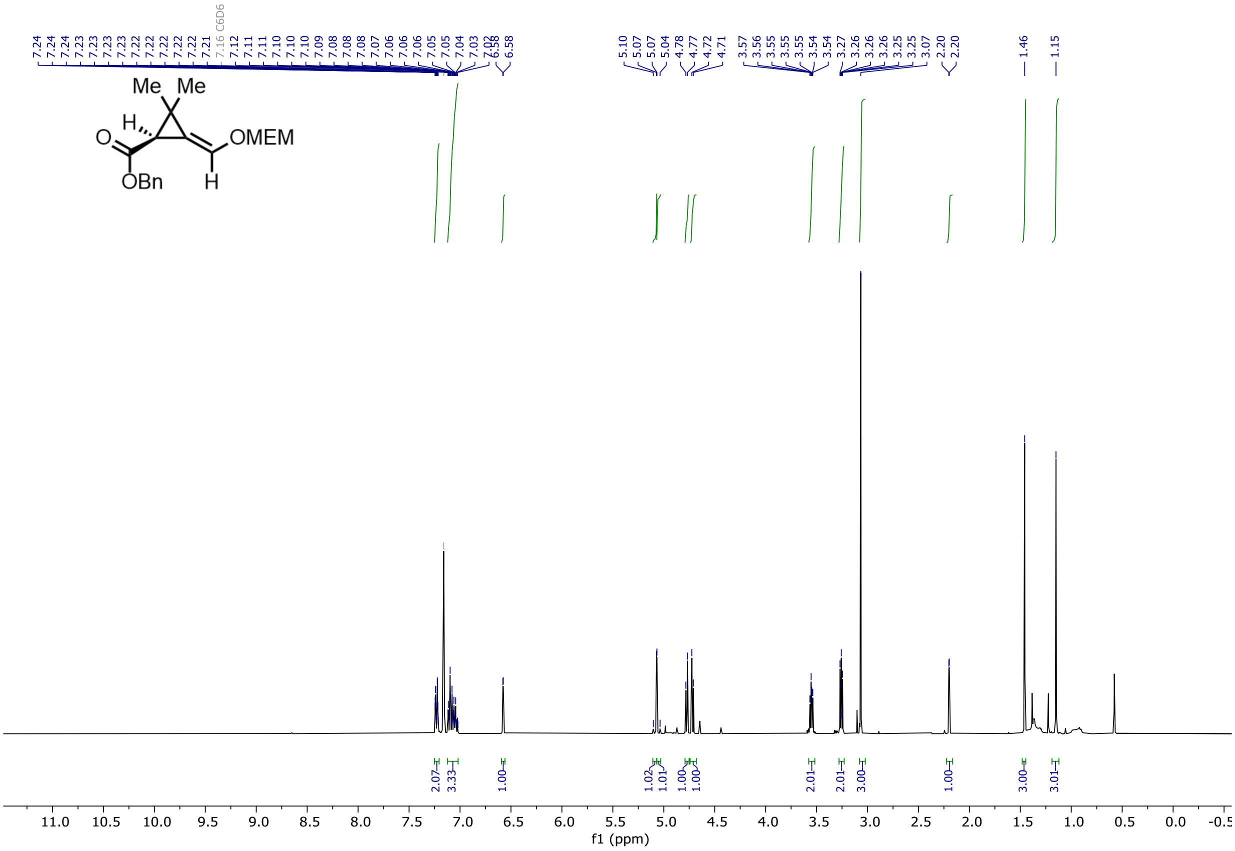

^{13}C NMR (101 MHz, Benzene-d_6) of **33b**

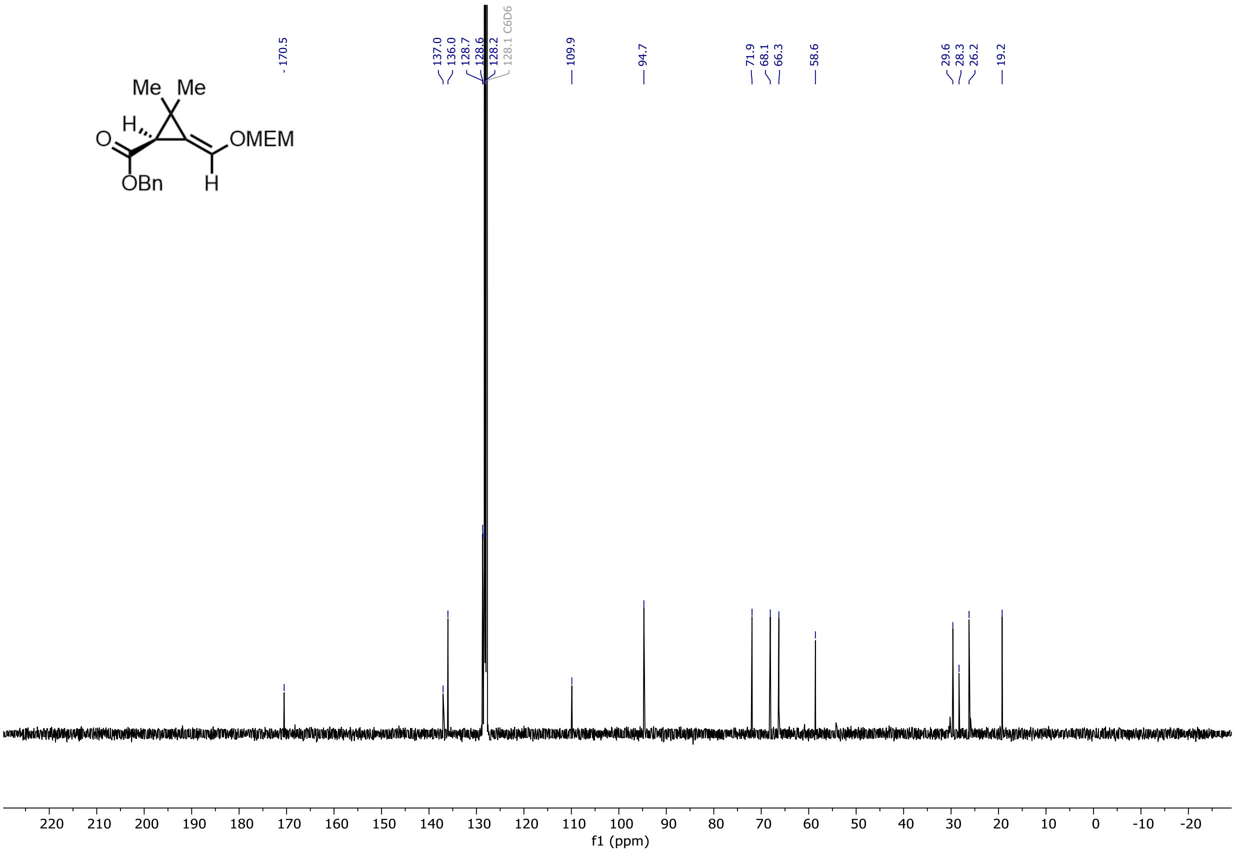

^{1}H NMR (400 MHz, $CDCl_3$) of **34b**

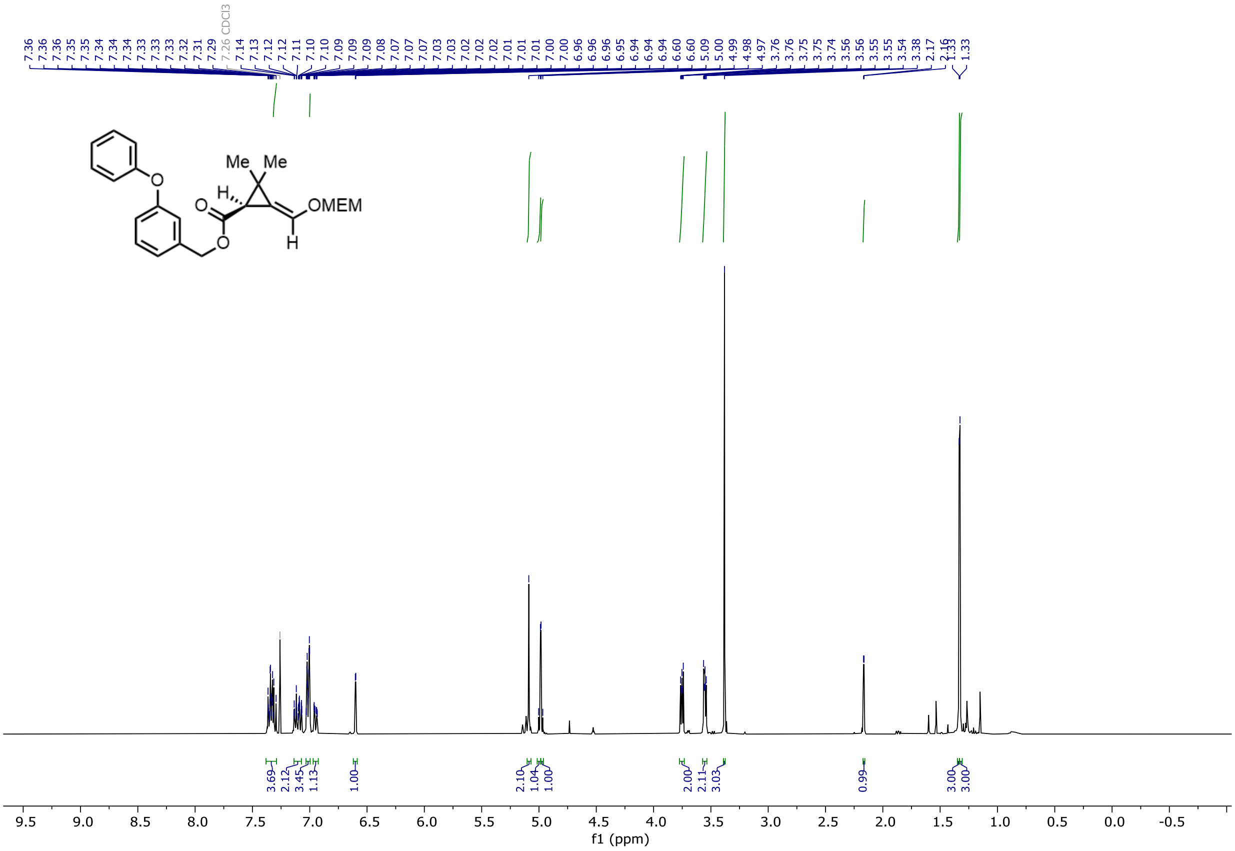

^{13}C NMR (101 MHz, $CDCl_3$) of **34b**

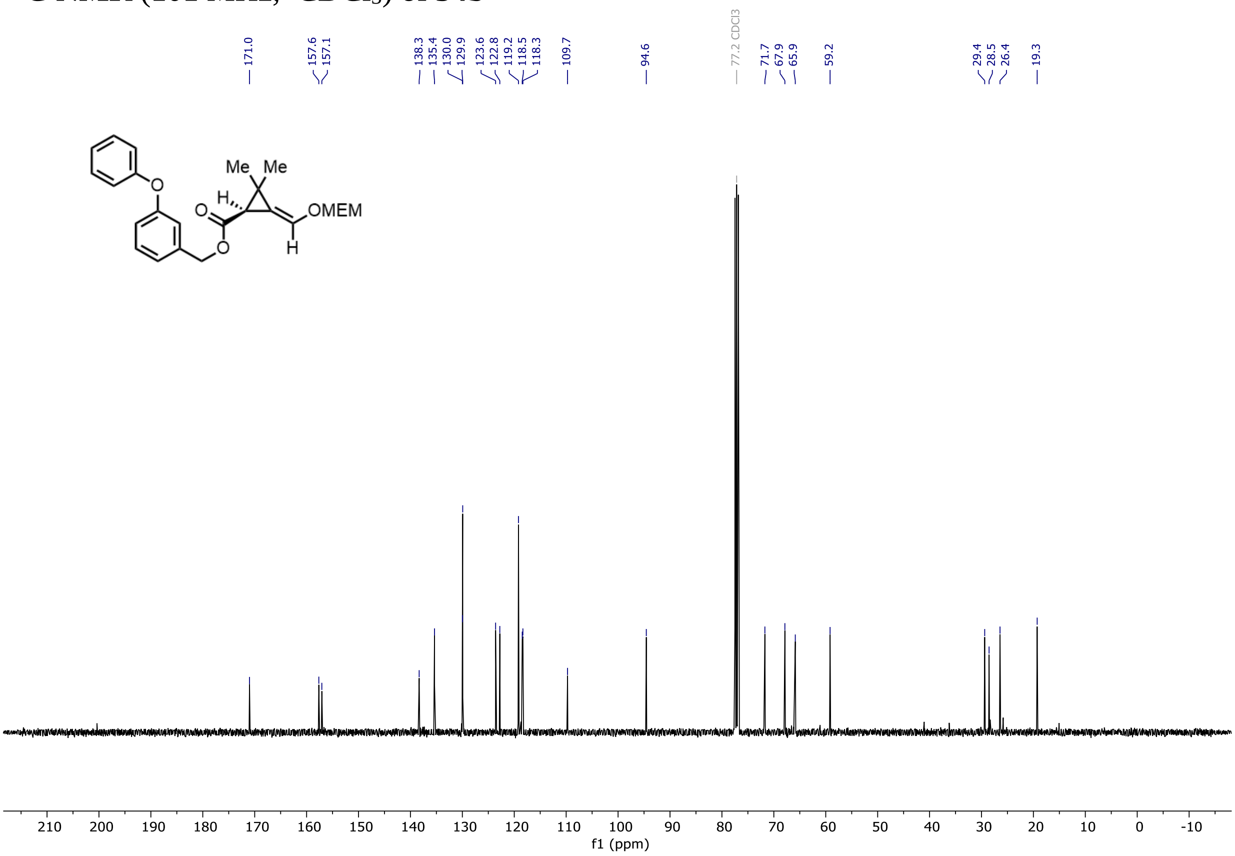

^{1}H NMR (500 MHz, $CDCl_3$) of **2**

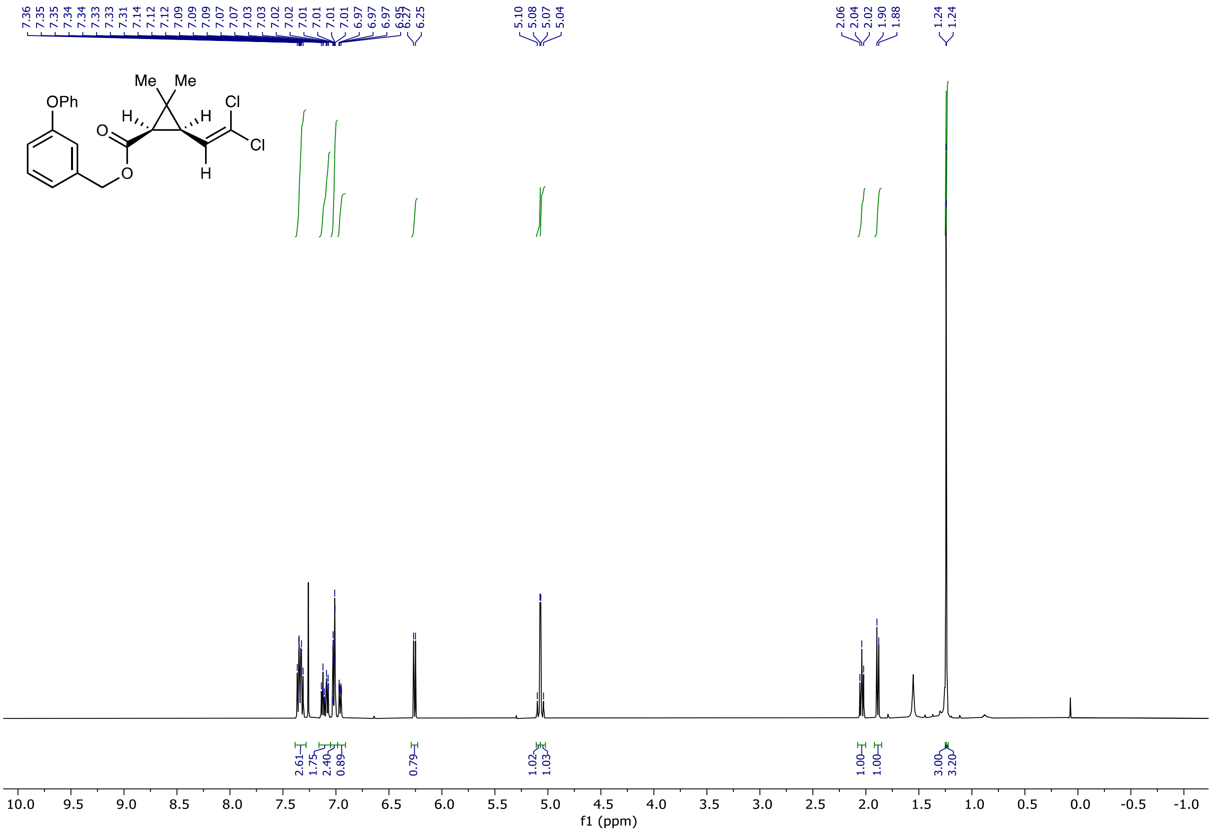

^{13}C NMR (126 MHz, $CDCl_3$) of **2**

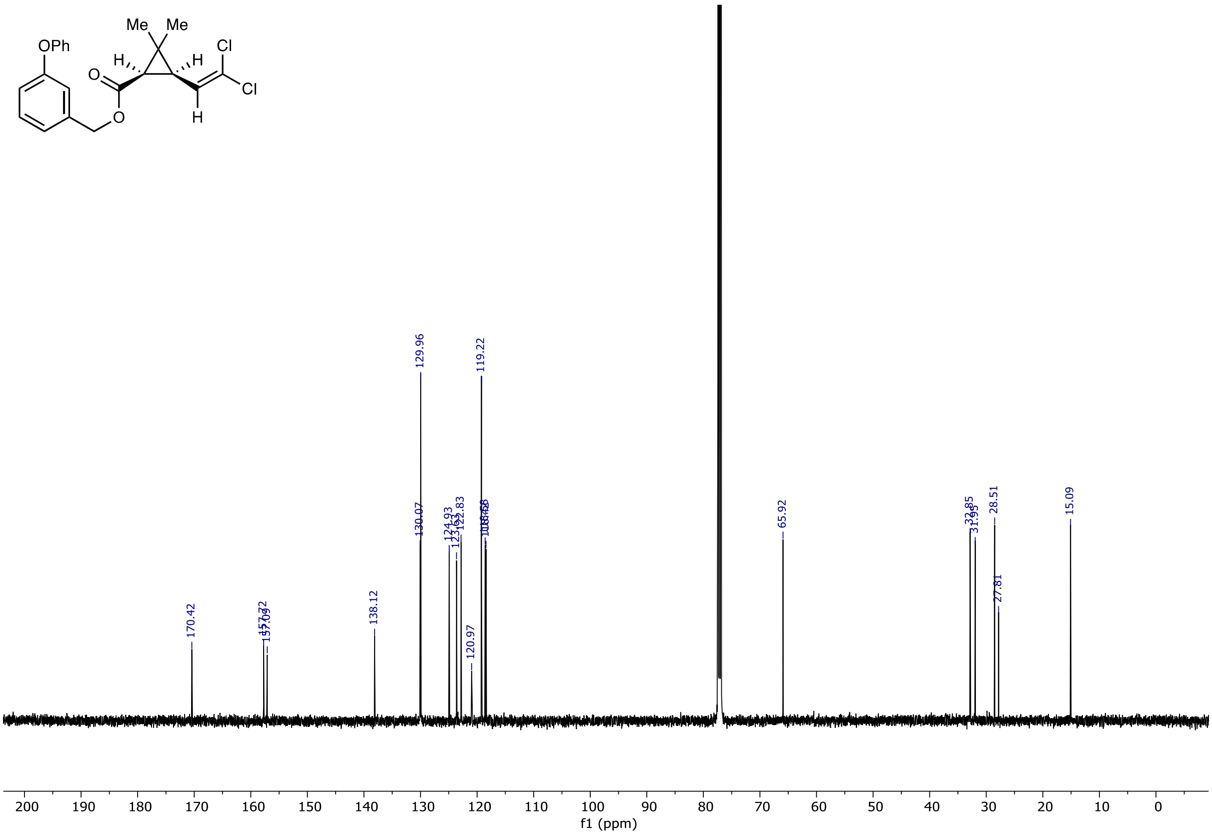

^{1}H NMR (500 MHz, $CDCl_3$) of **35b**

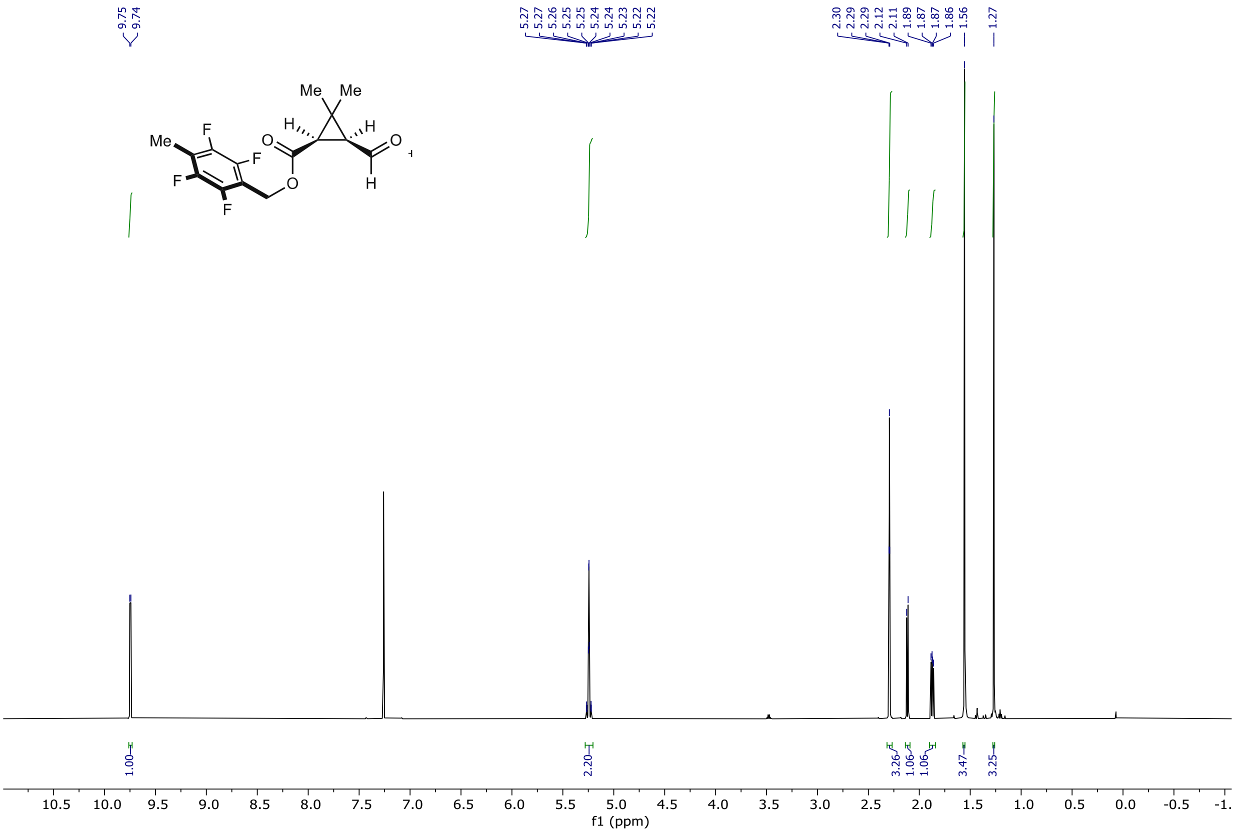

^{13}C NMR (126 MHz, $CDCl_3$) of **35b**

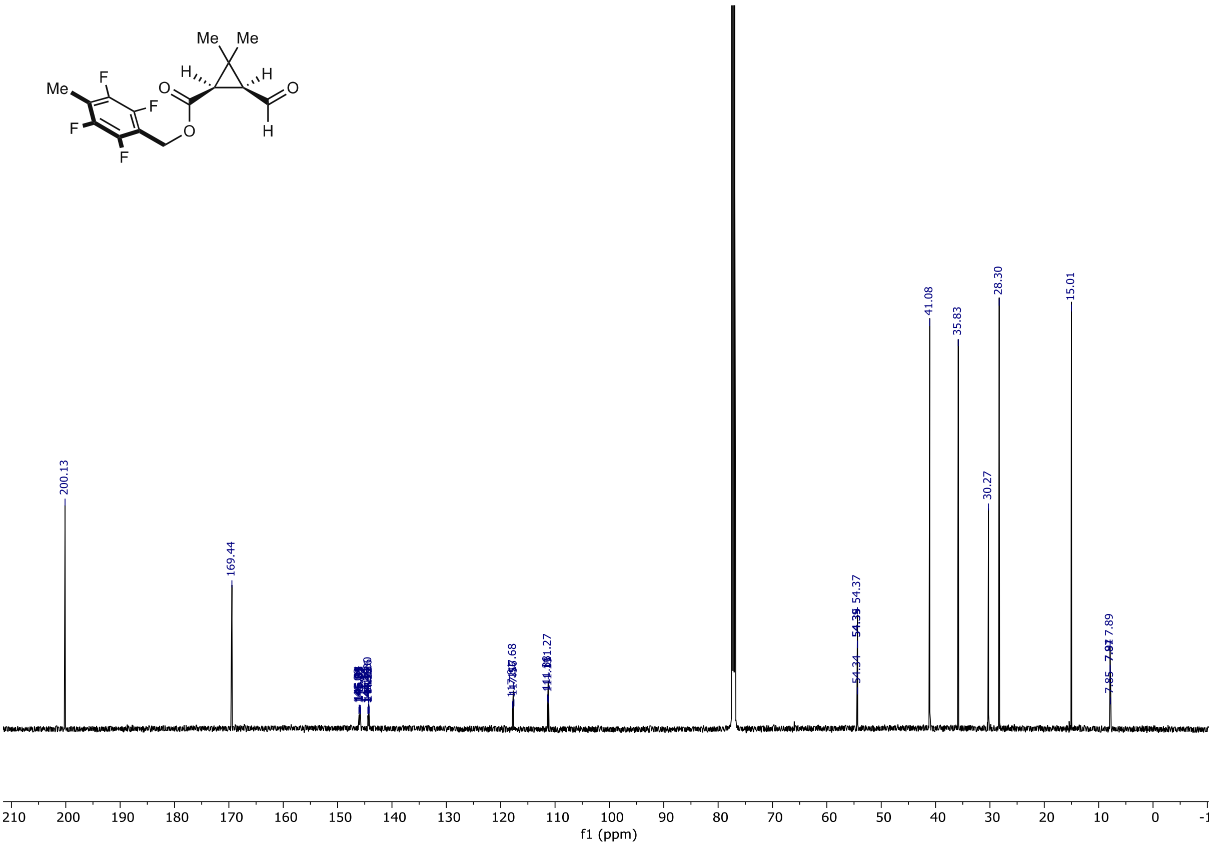

^{19}F NMR (471 MHz, $CDCl_3$) of **35b**

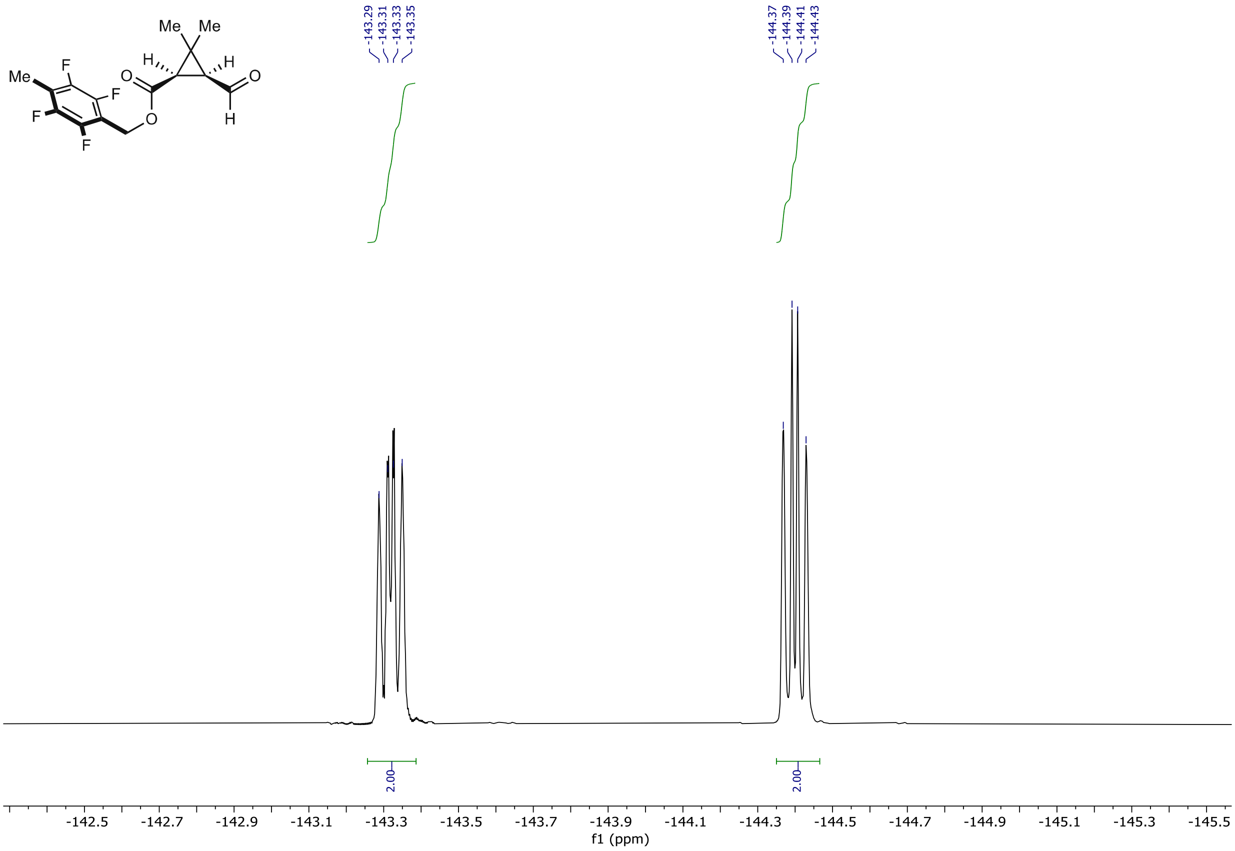

^{1}H NMR (500 MHz, $CDCl_3$) of **36b**

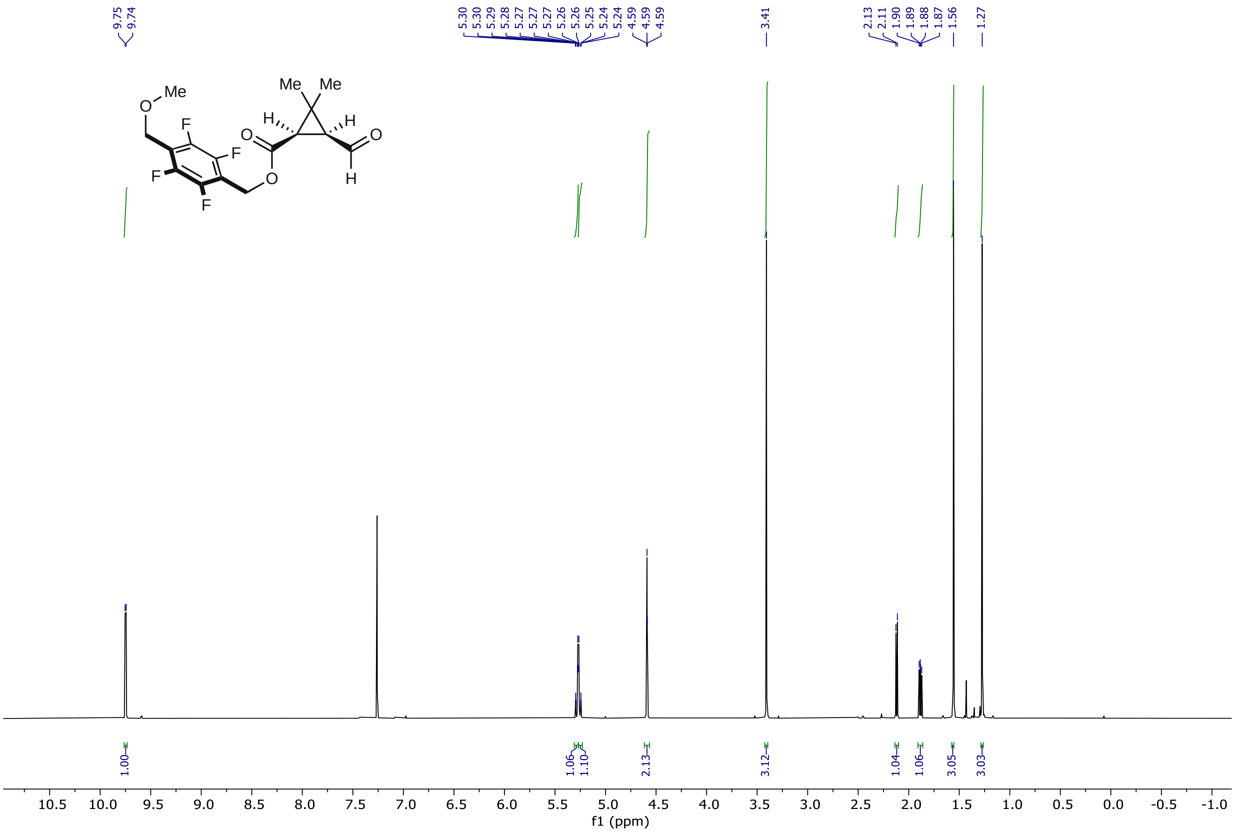

^{13}C NMR (126 MHz, $CDCl_3$) of **36b**

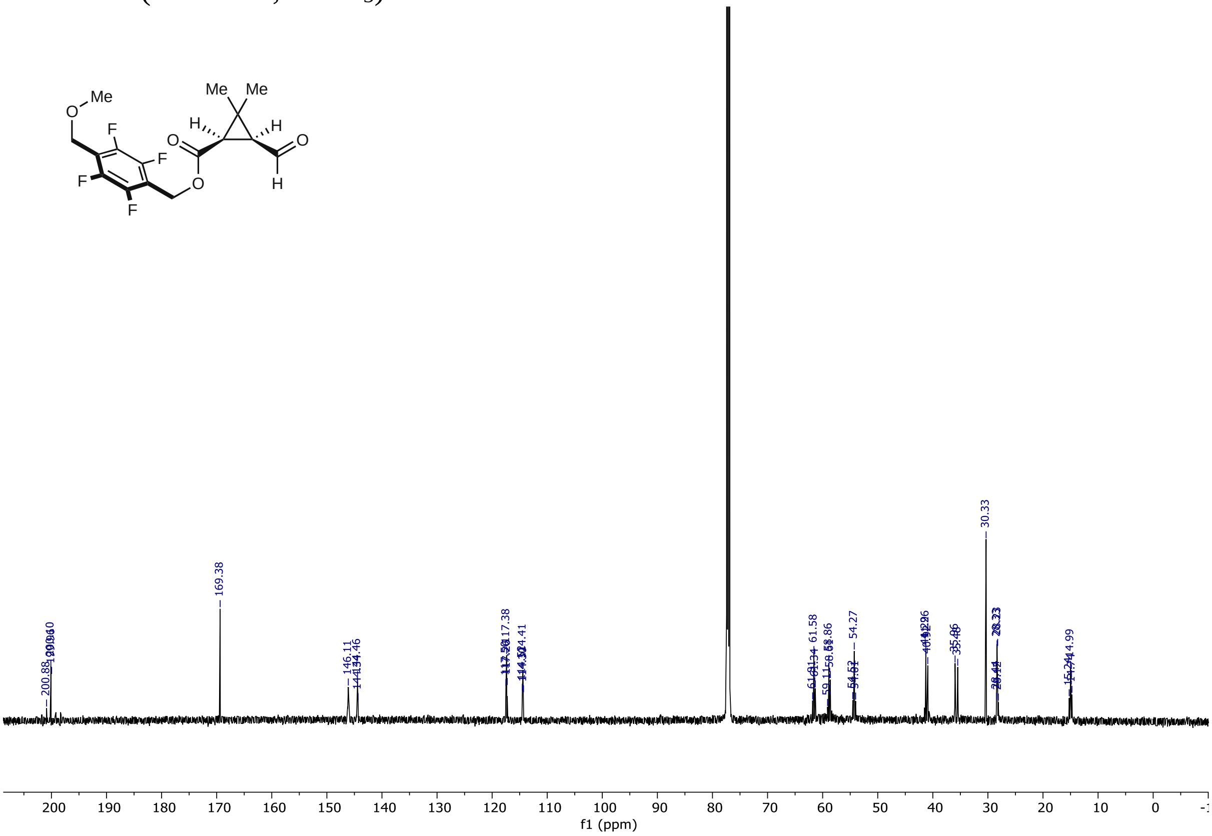

^{19}F NMR (471 MHz, $CDCl_3$) of **36b**

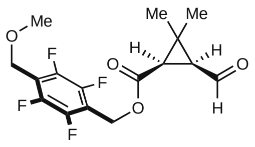

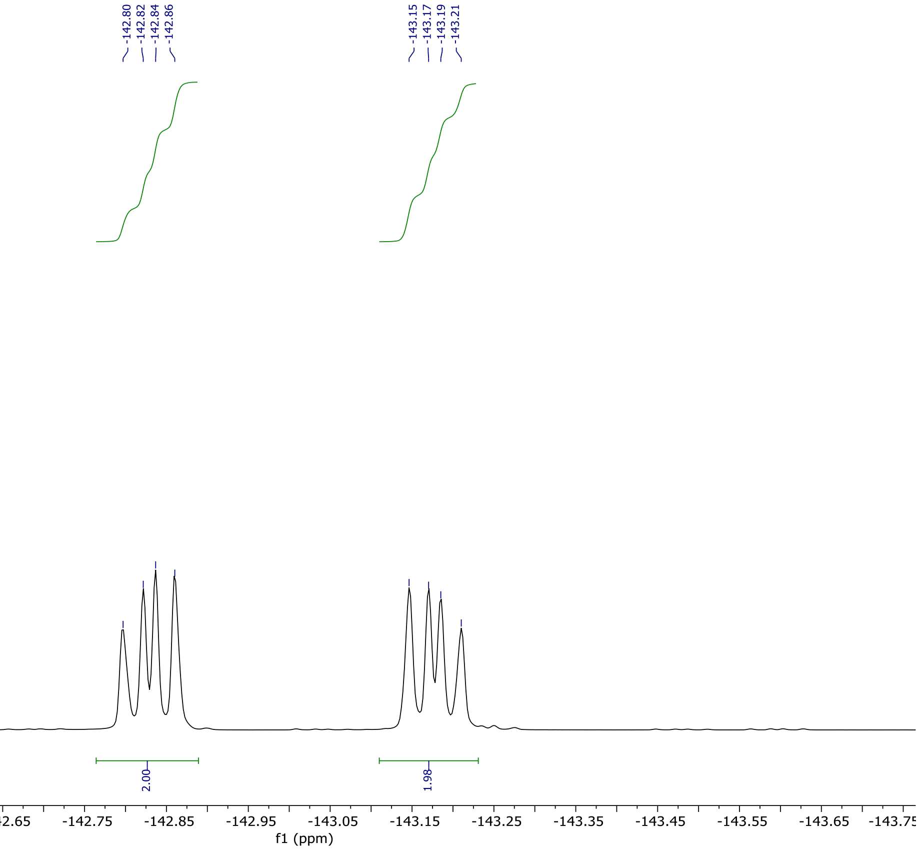

^{1}H NMR (600 MHz, $CDCl_3$) of **37b**

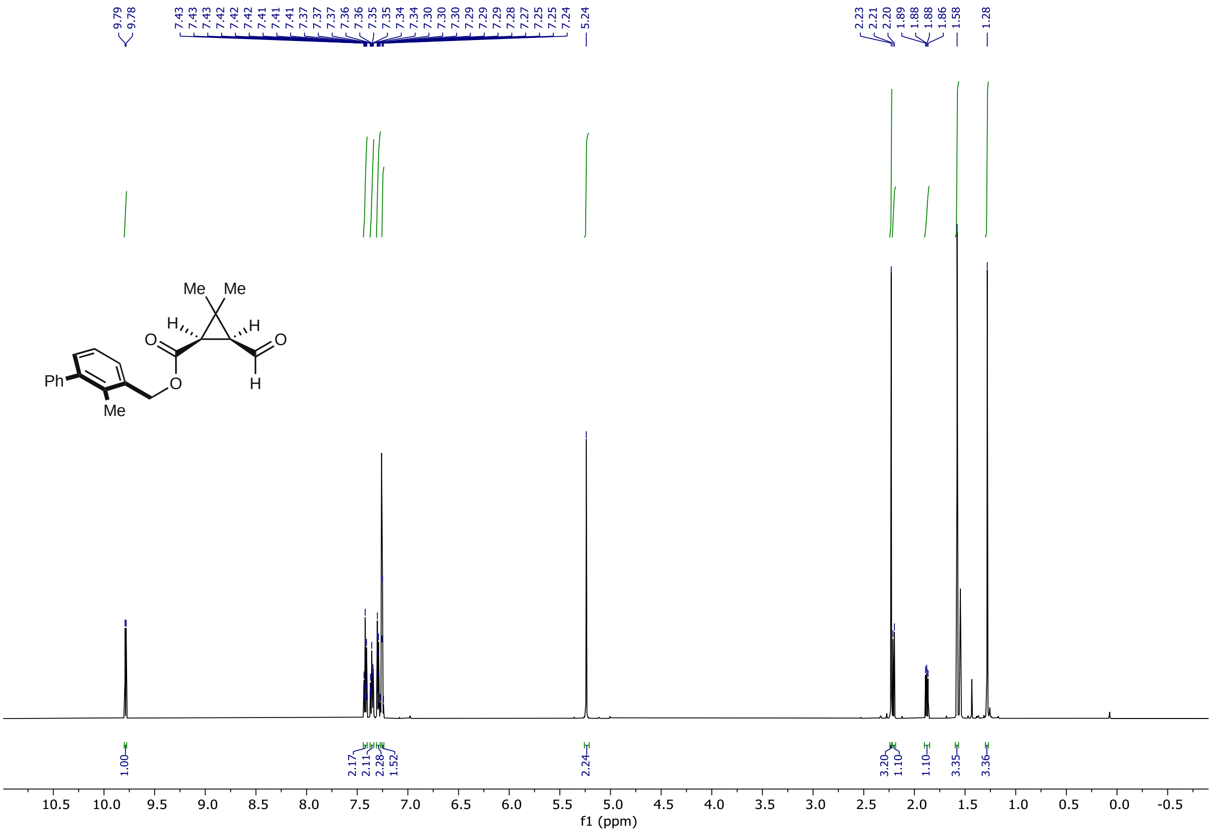

^{13}C NMR (151 MHz, $CDCl_3$) of **37b**

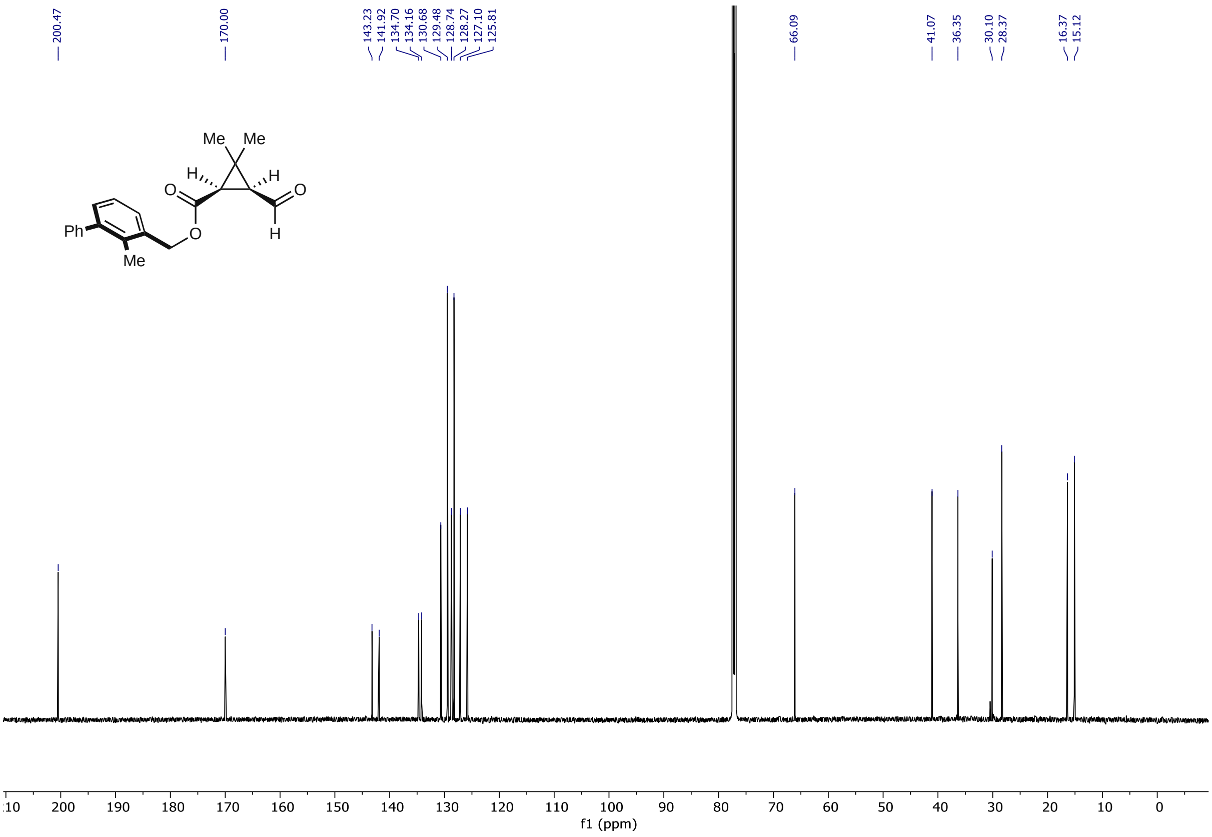

¹H NMR (400 MHz, $CDCl_3$) of **38b**

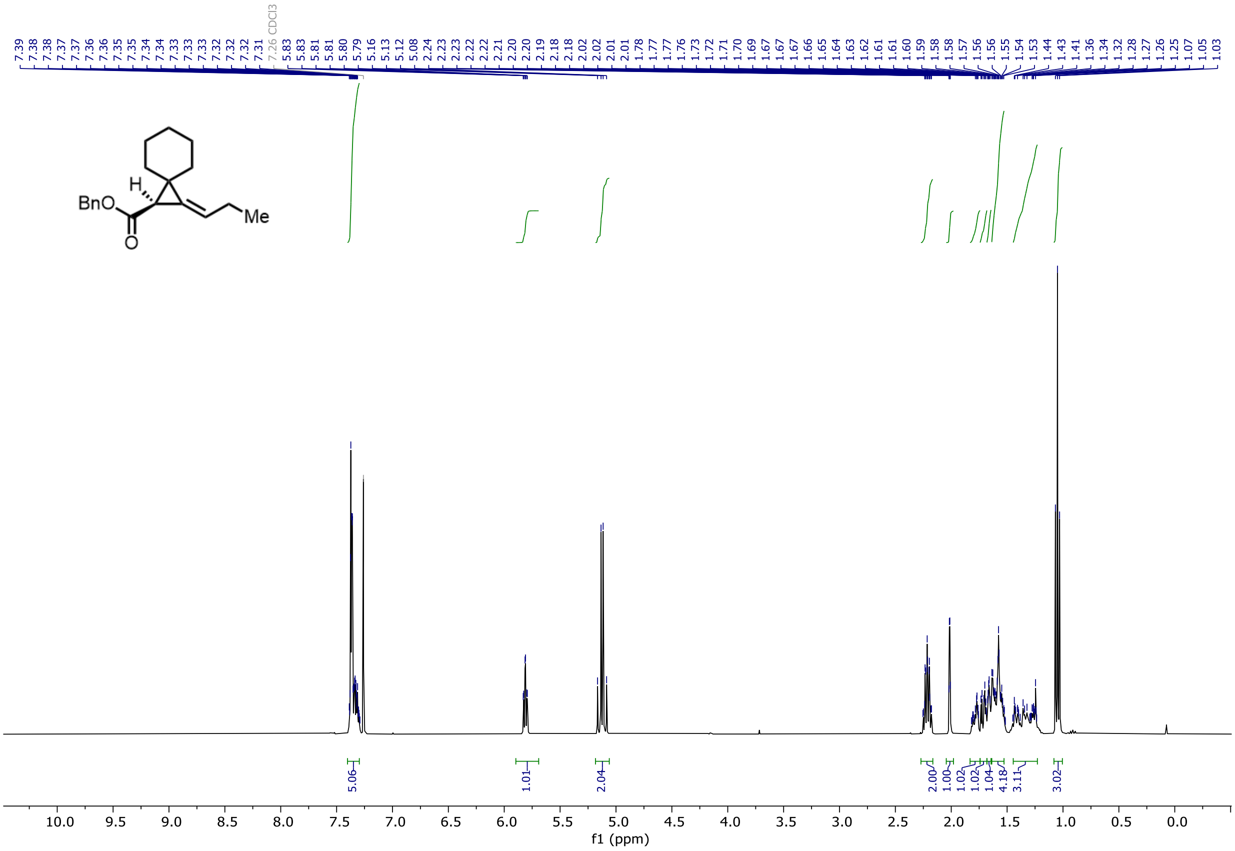

¹³C NMR (101 MHz, $CDCl_3$) of **38b**

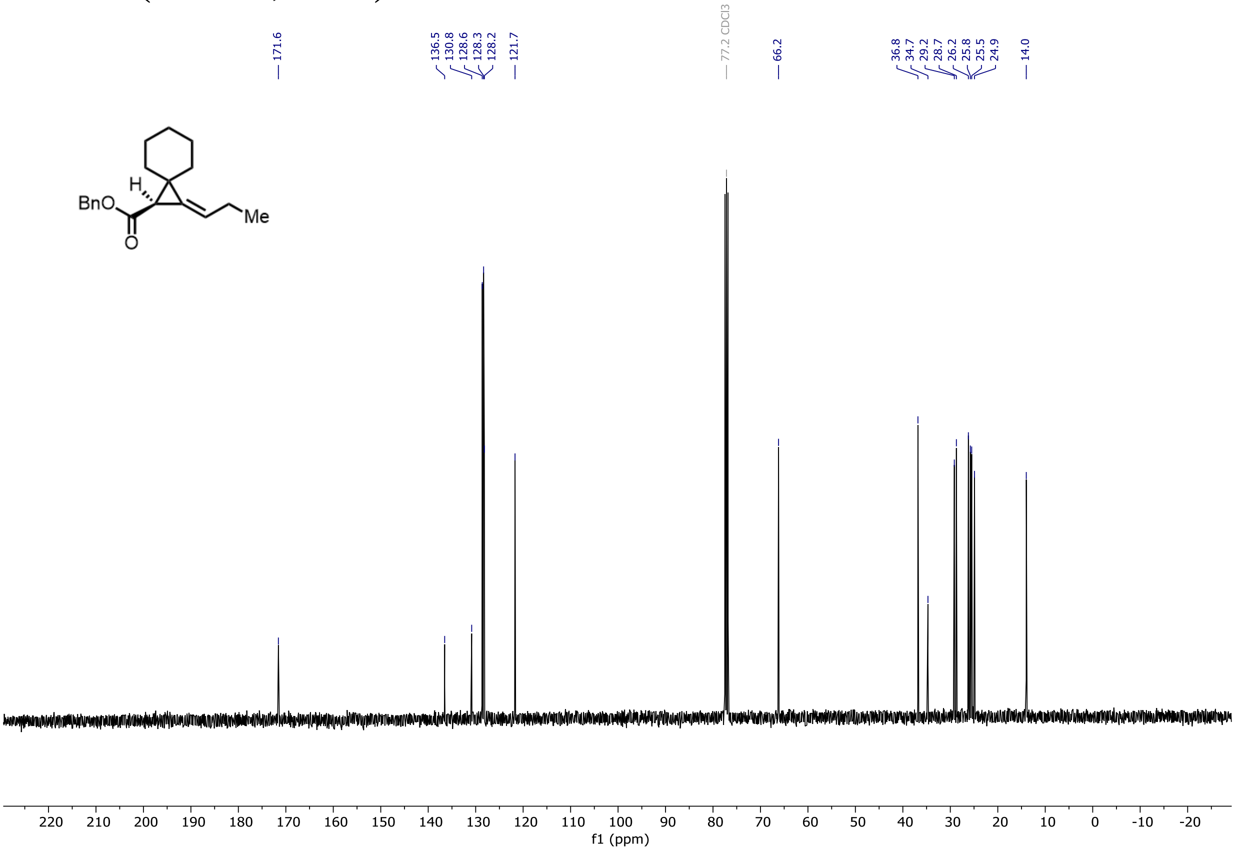

^{1}**H NMR** (400 MHz, Benzene-d_6) of **39b**

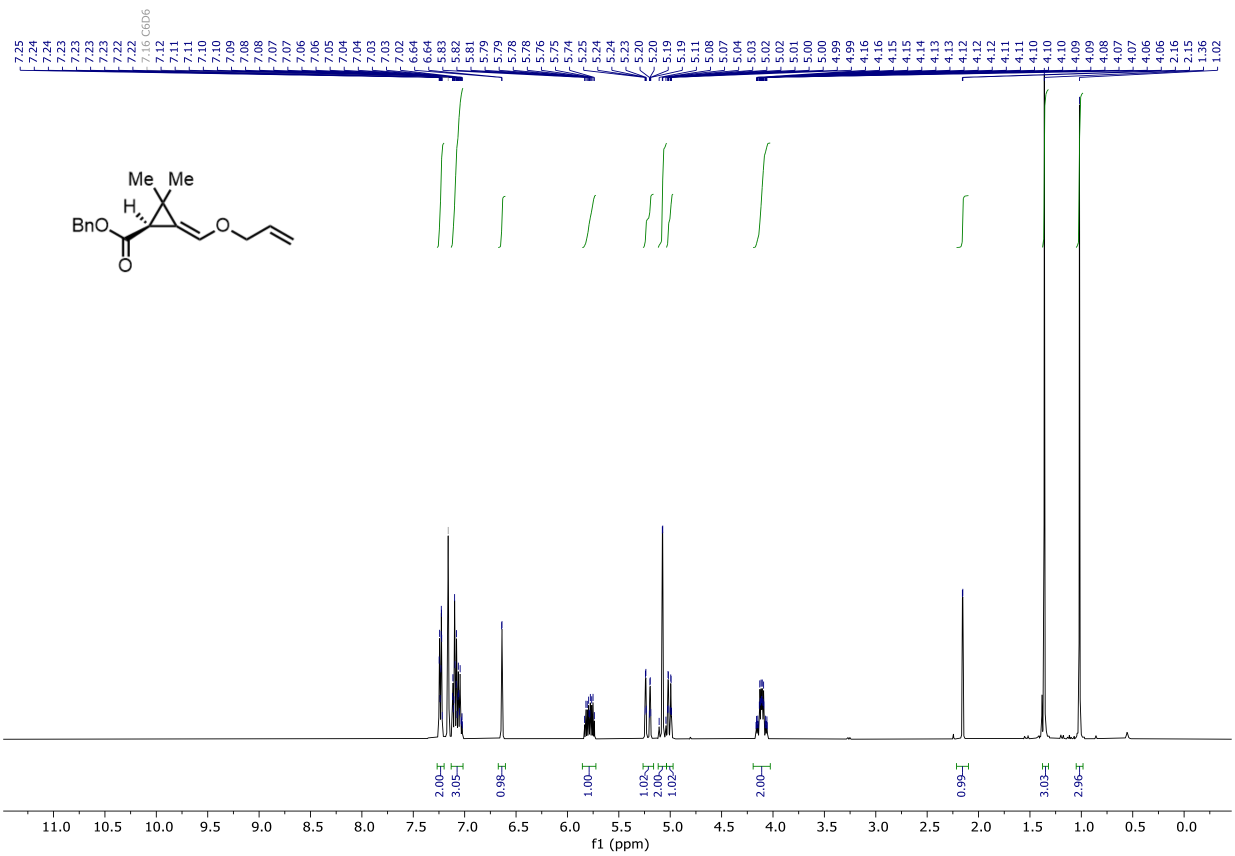

^{13}**C NMR** (101 MHz, Benzene-d_6) of **39b**

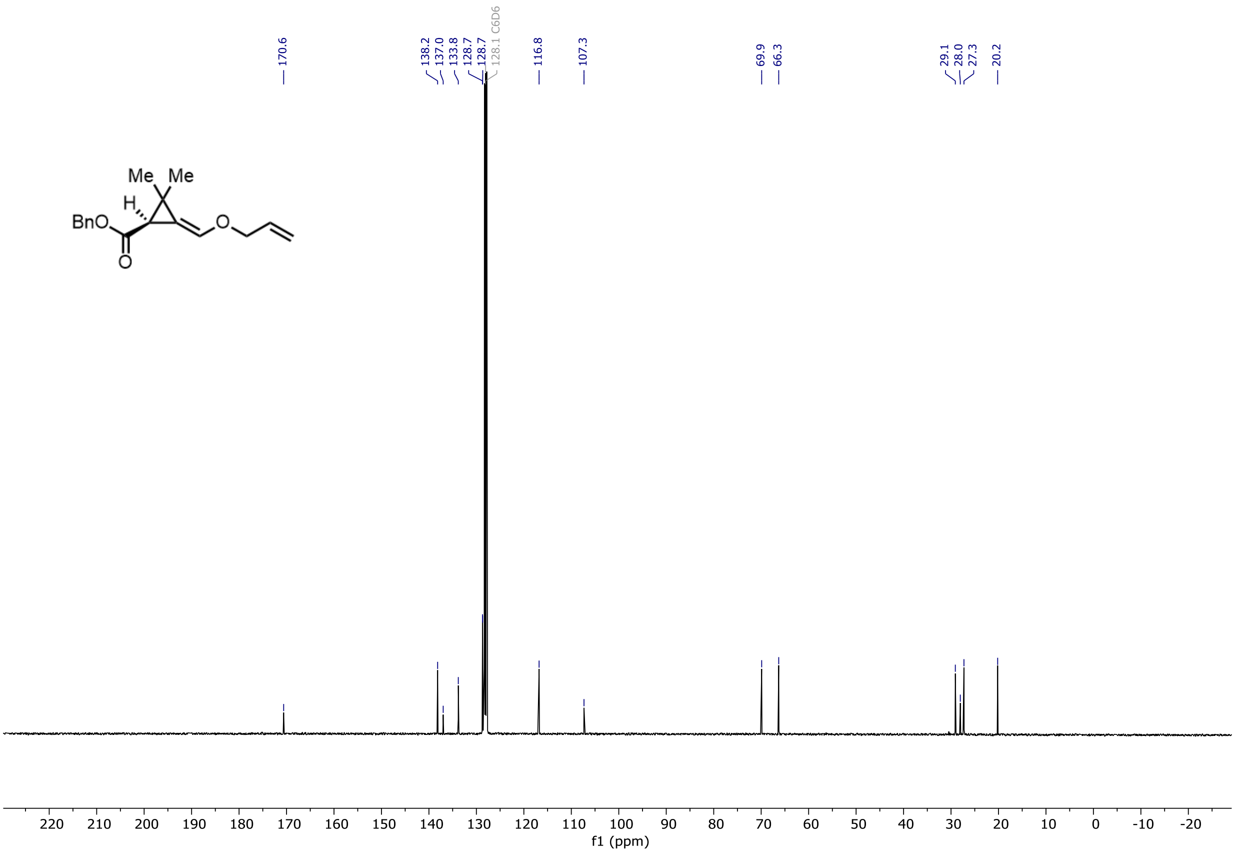

^{1}H NMR (400 MHz, Benzene-d_6) of **40**

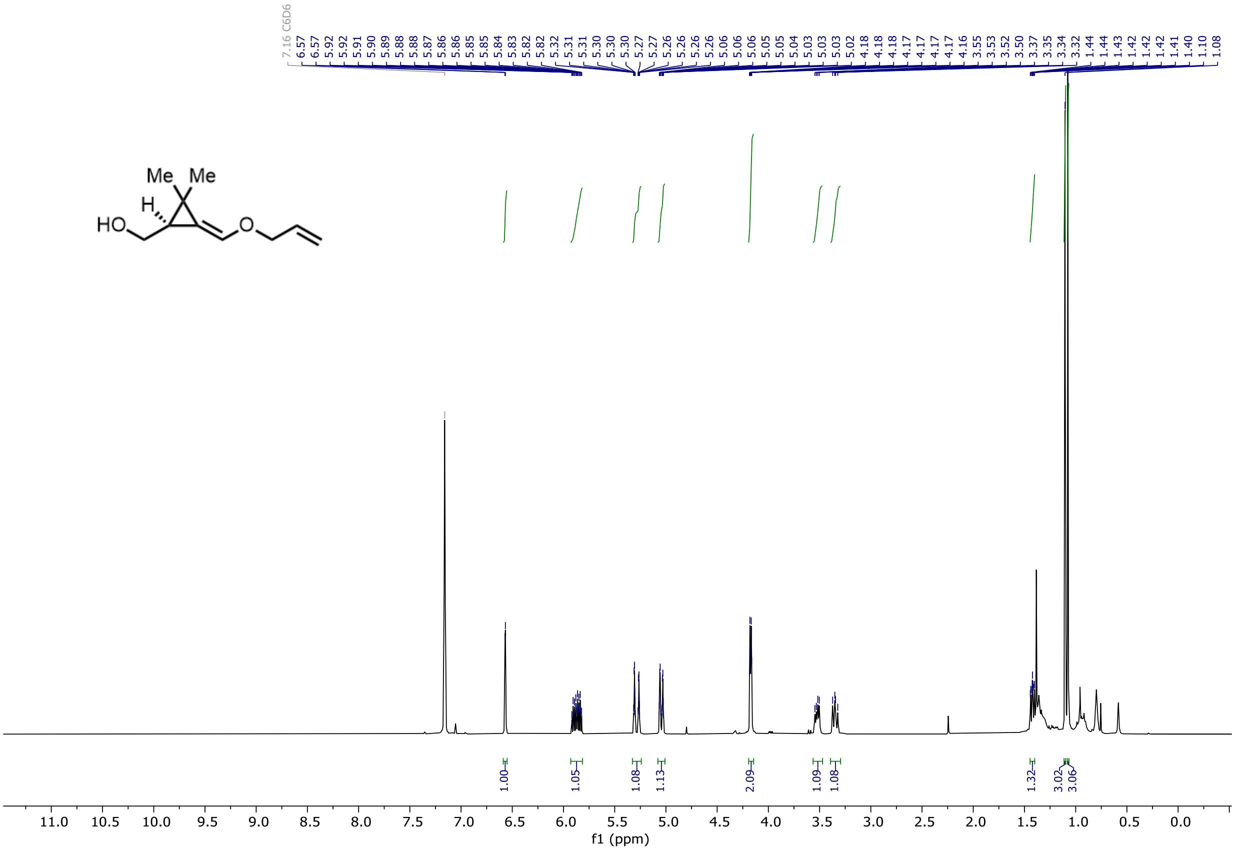

^{13}C NMR (101 MHz, Benzene-d_6) of **40**

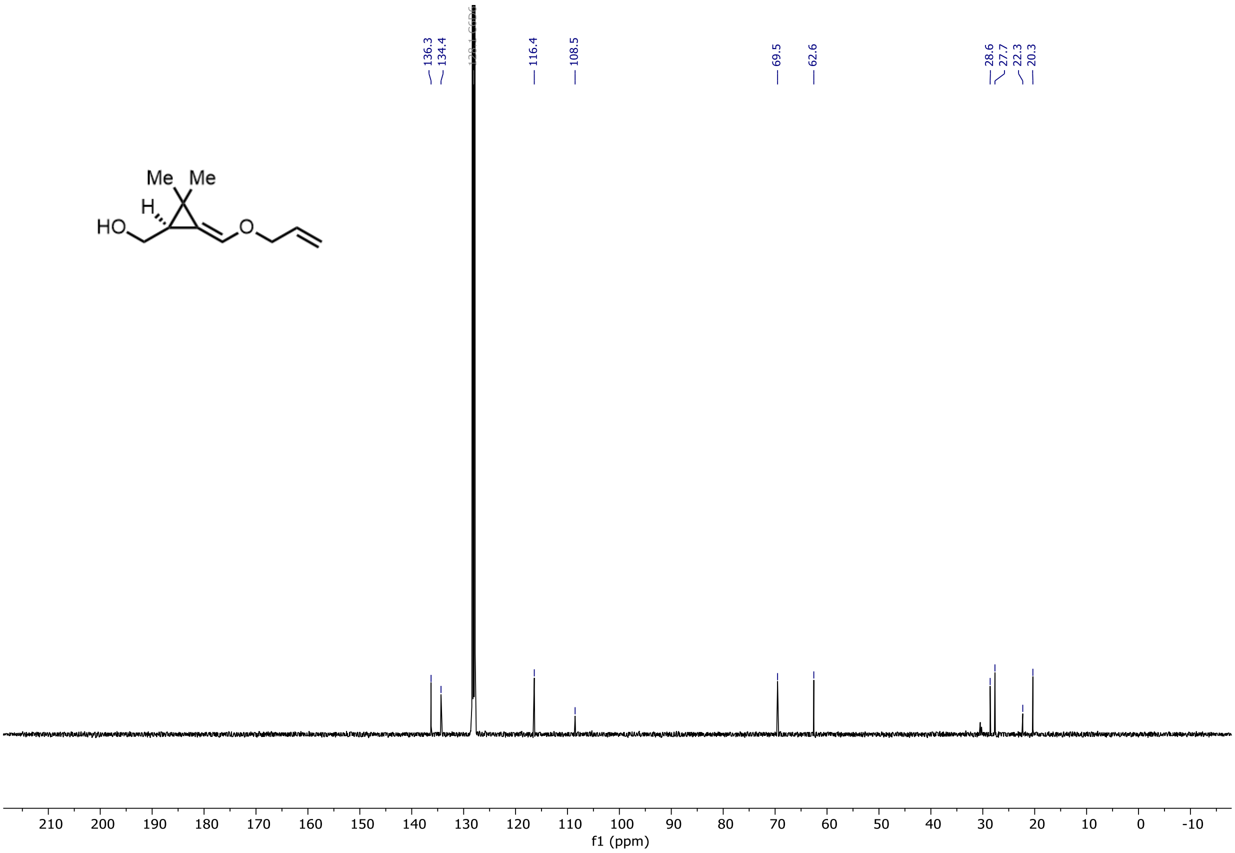

^{1}**H NMR** (400 MHz, CDCl$_3$) of **41**

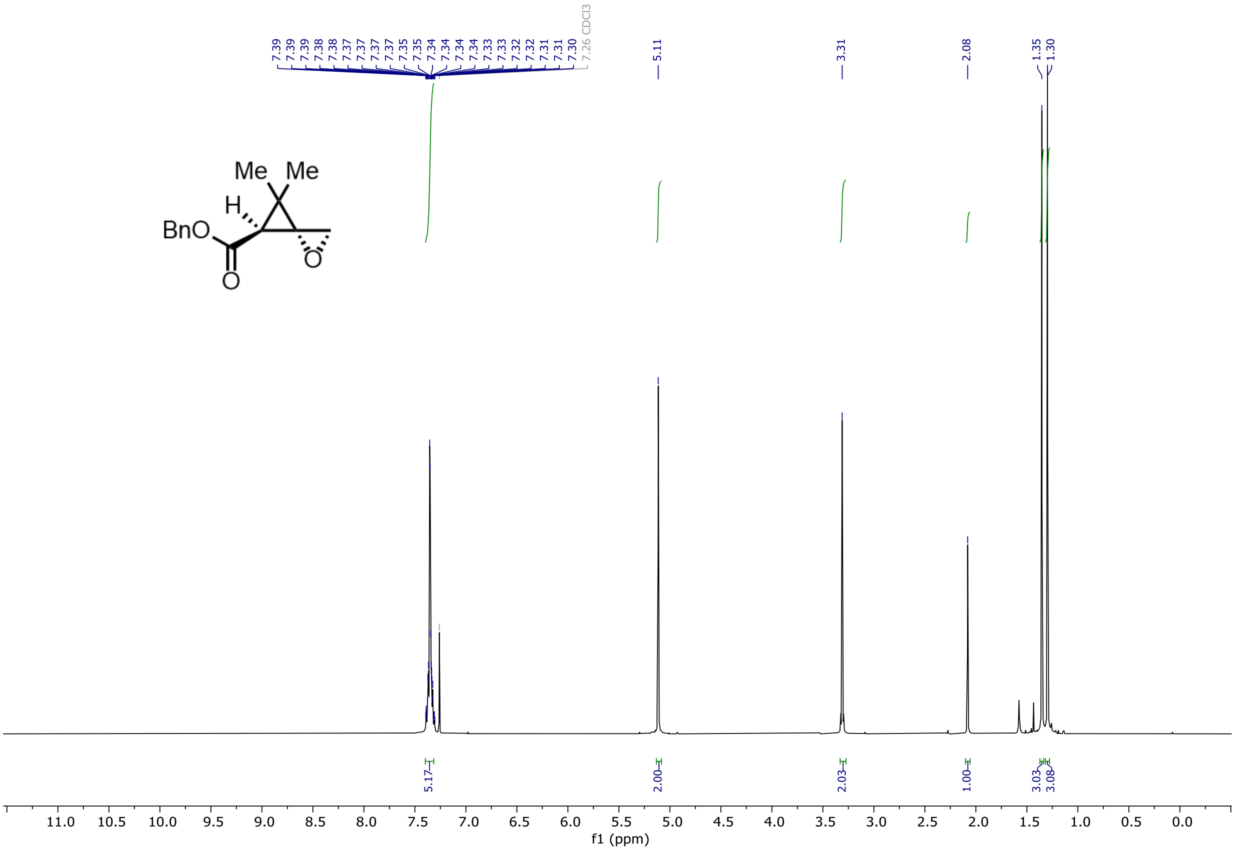

^{13}**C NMR** (101 MHz, CDCl$_3$) of **41**

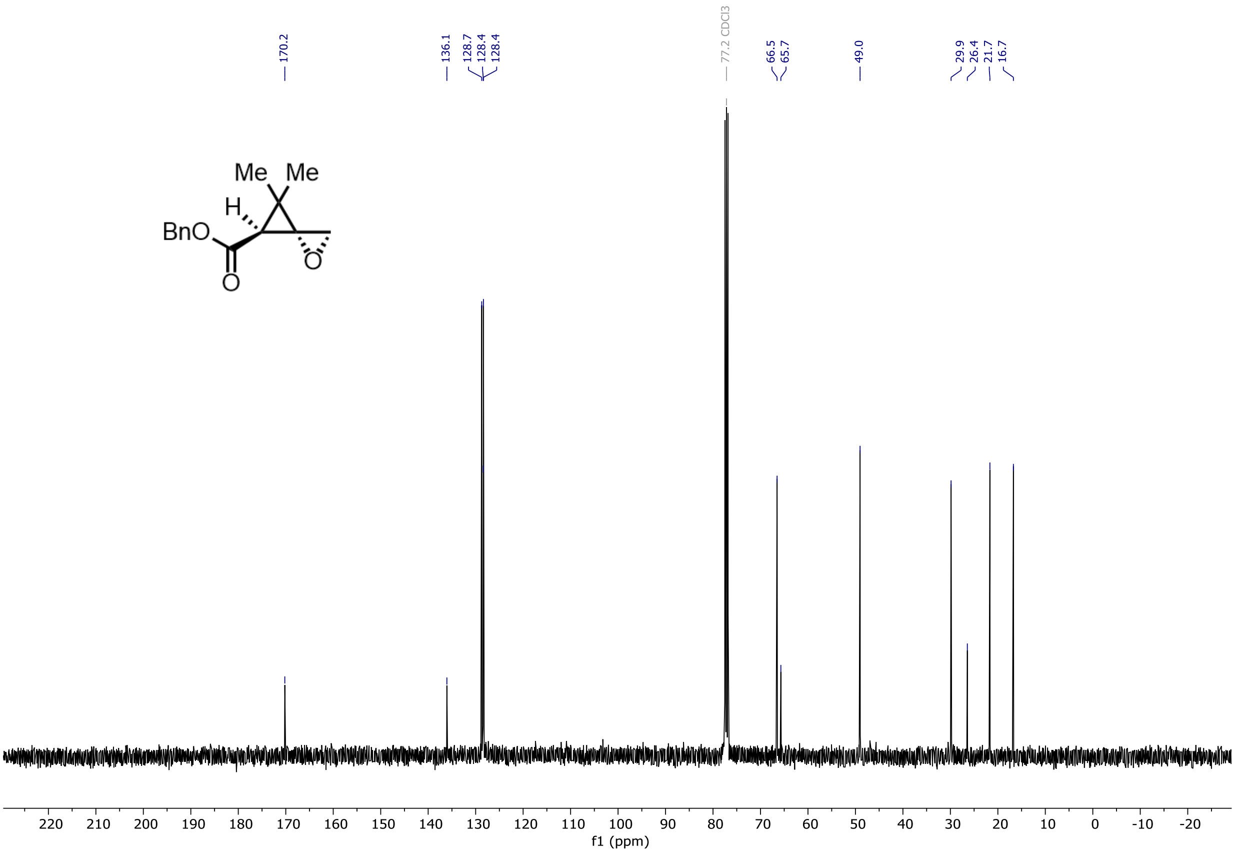

^{1}H NMR (400 MHz, $CDCl_3$) of ***S30-d*₂**

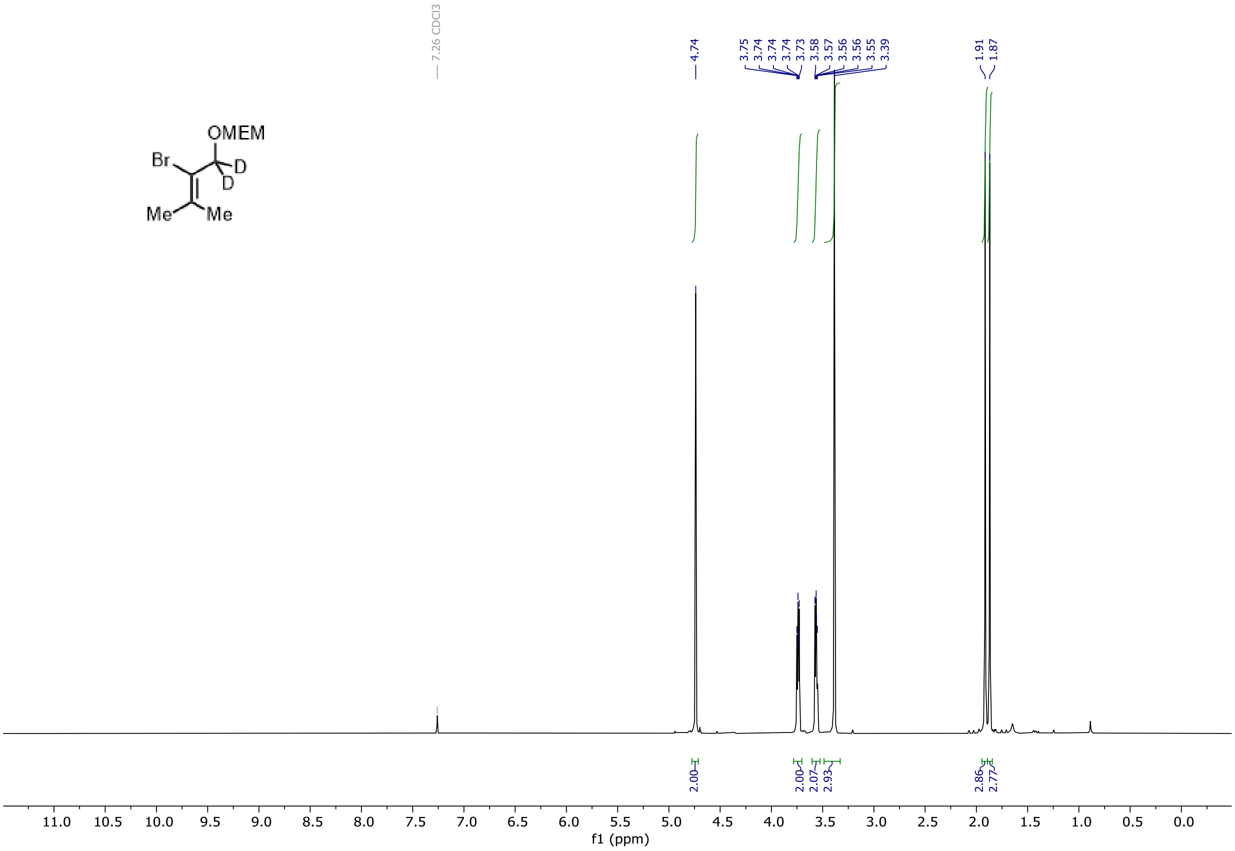

^{13}C NMR (101 MHz, $CDCl_3$) of ***S30-d*₂**

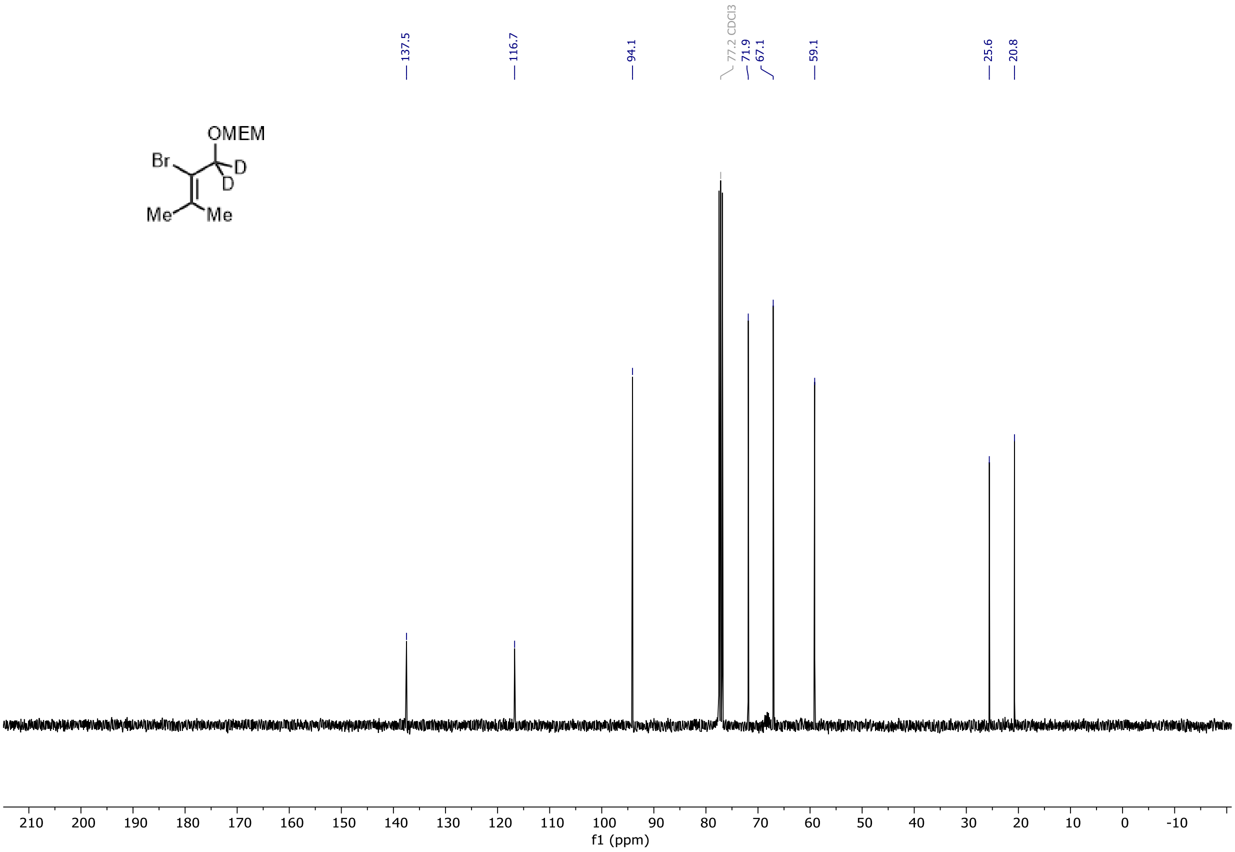

^{1}H NMR (400 MHz, $CDCl_3$) of **S31-*d*$_2$**

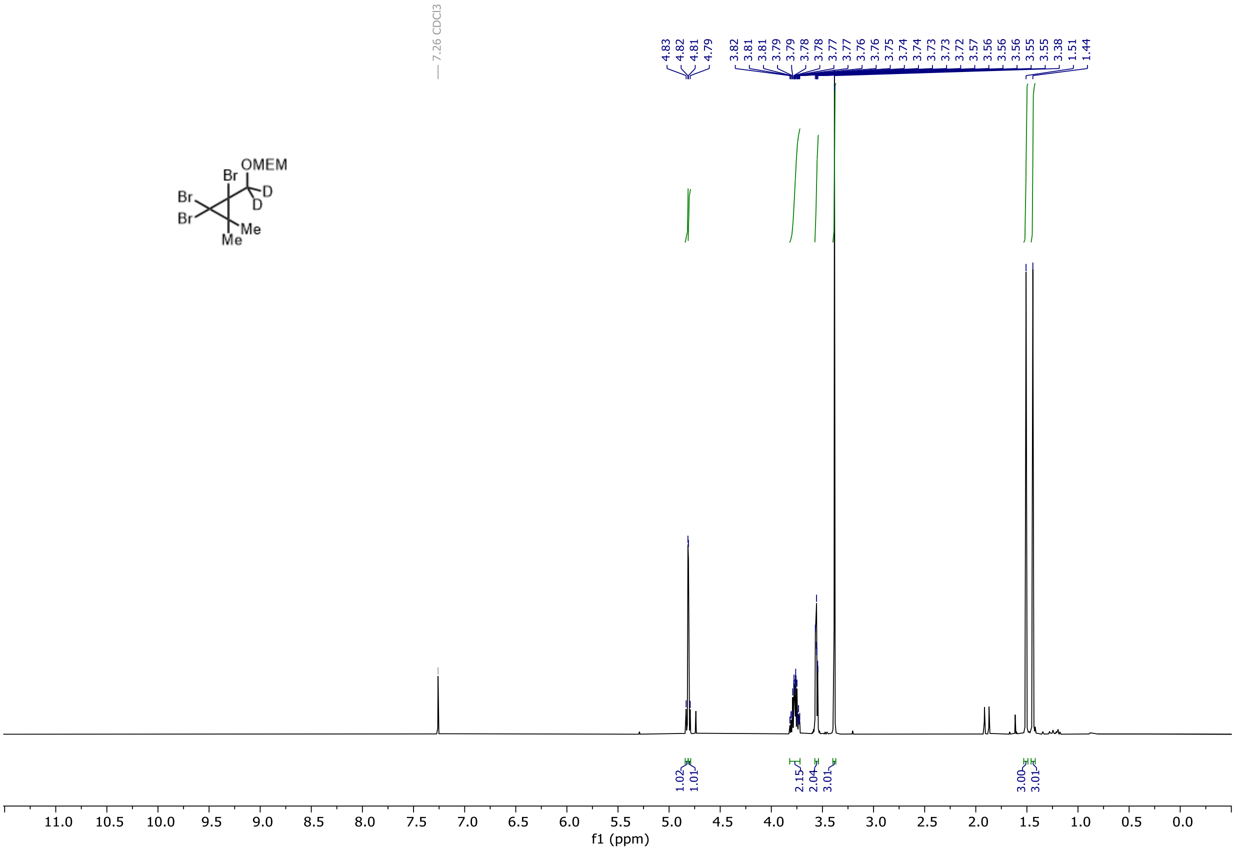

^{13}C NMR (101 MHz, $CDCl_3$) of **S31-*d*$_2$**

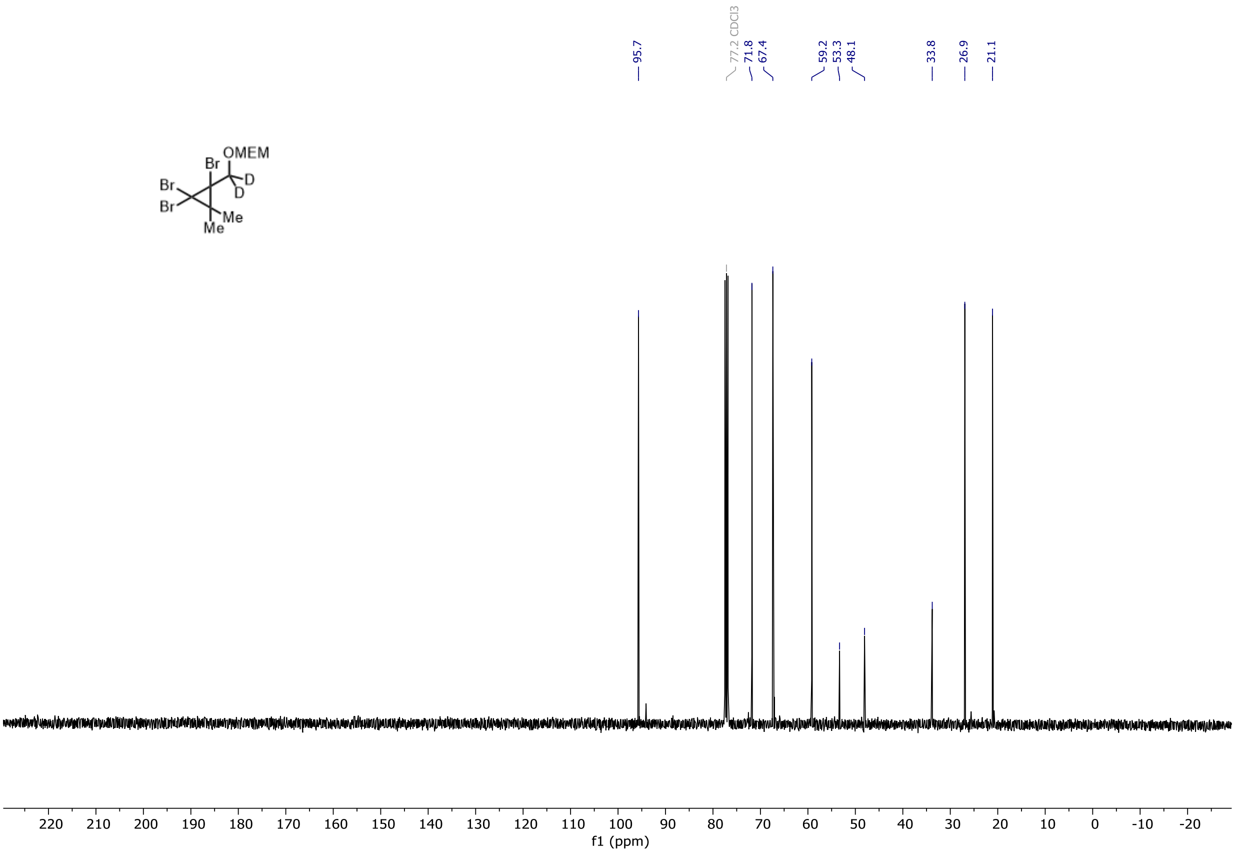

^{1}H NMR (400 MHz, $CDCl_3$) of **33a-*d*$_2$**

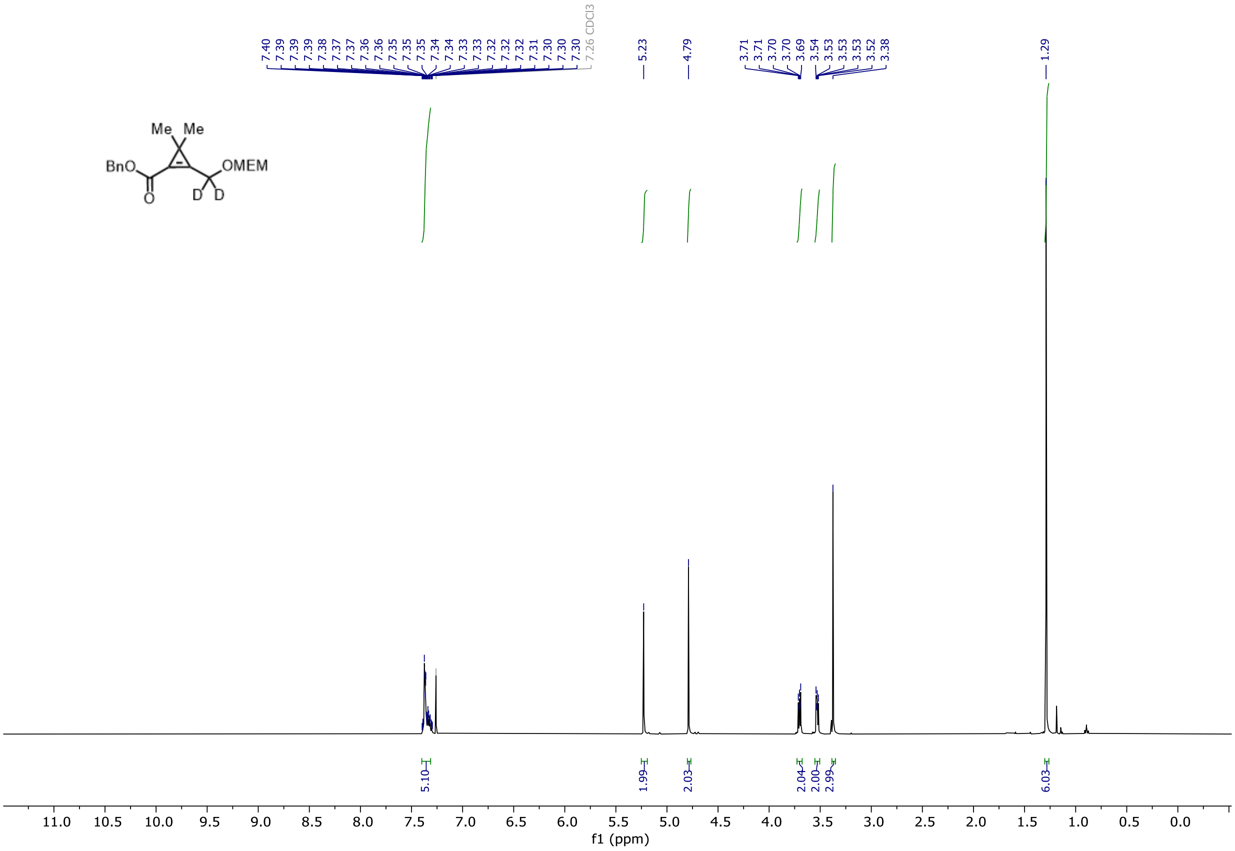

^{13}C NMR (101 MHz, $CDCl_3$) of **33a- *d*$_2$**

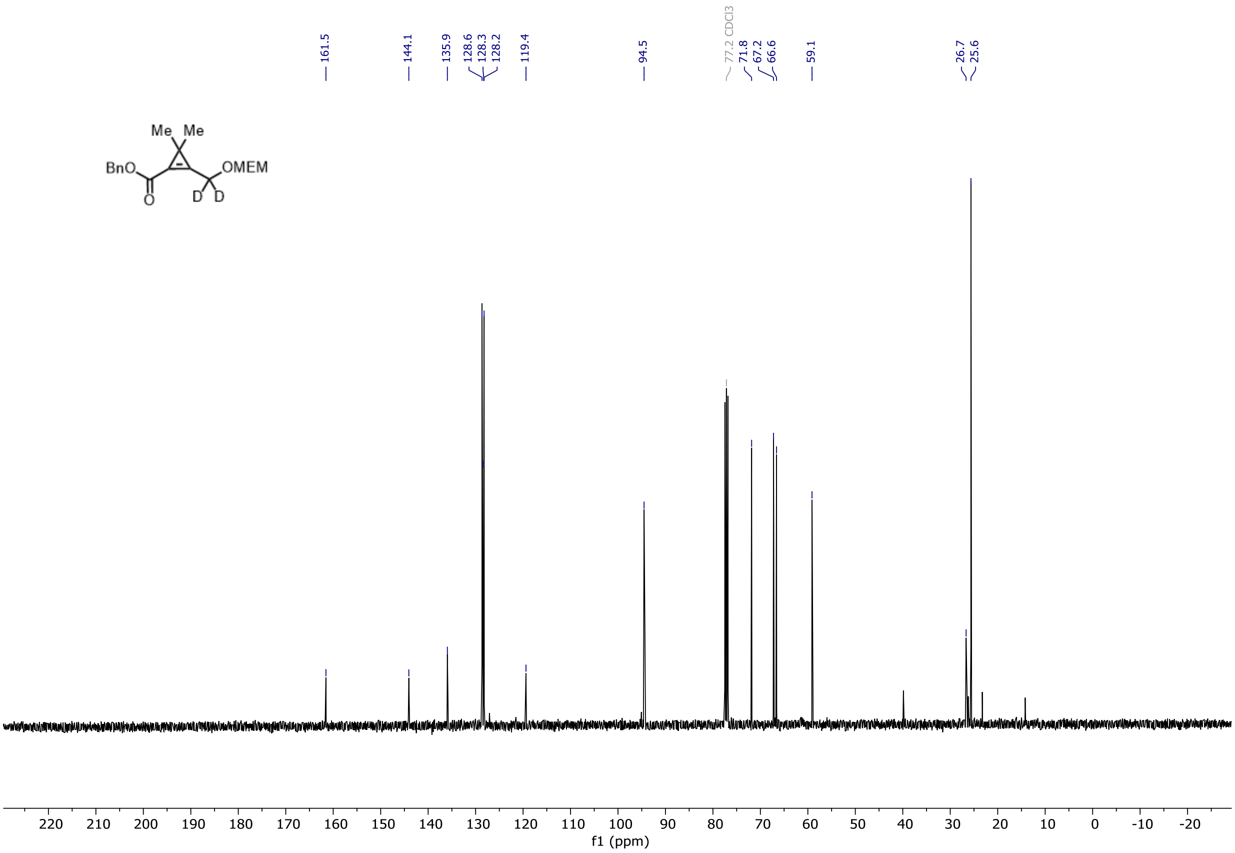

^{1}H NMR (400 MHz, $CDCl_3$) of **S30-*d***

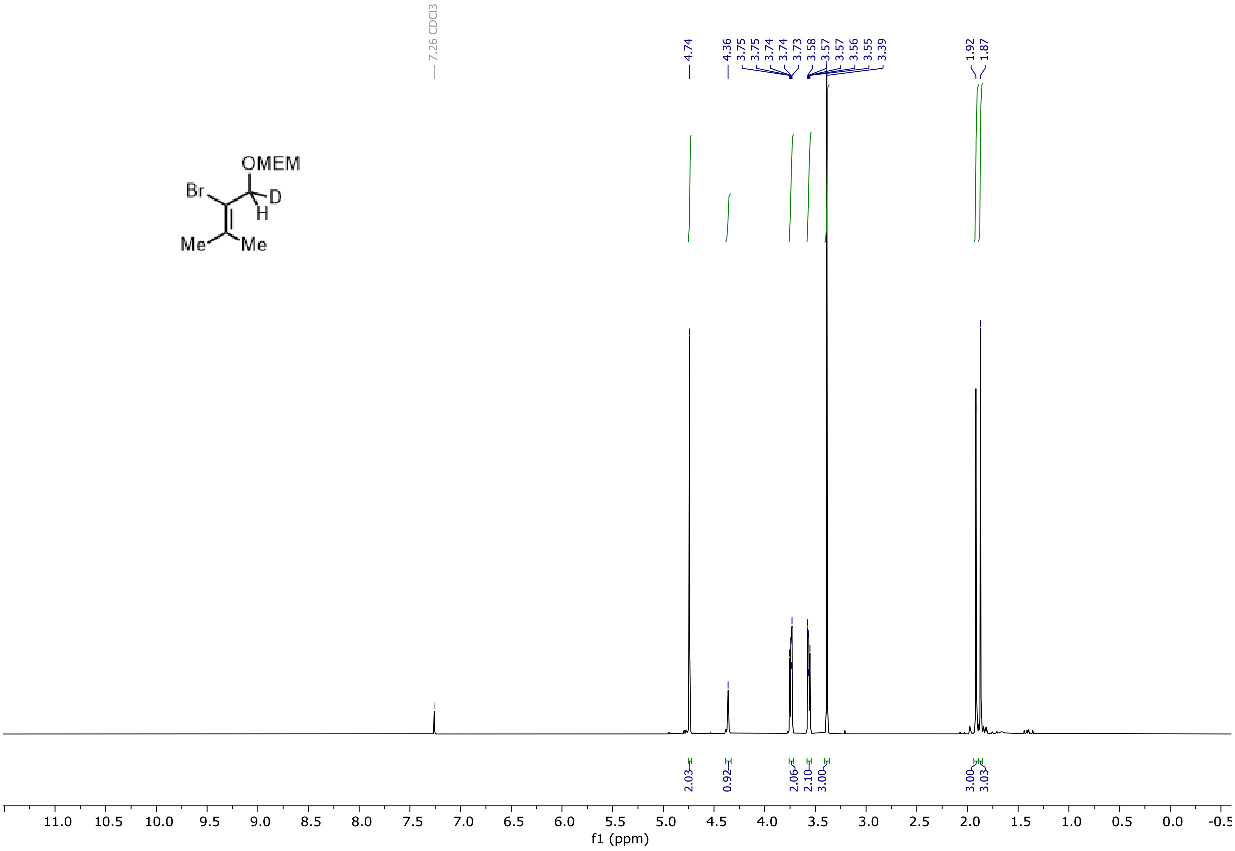

^{13}C NMR (101 MHz, $CDCl_3$) of **S30-*d***

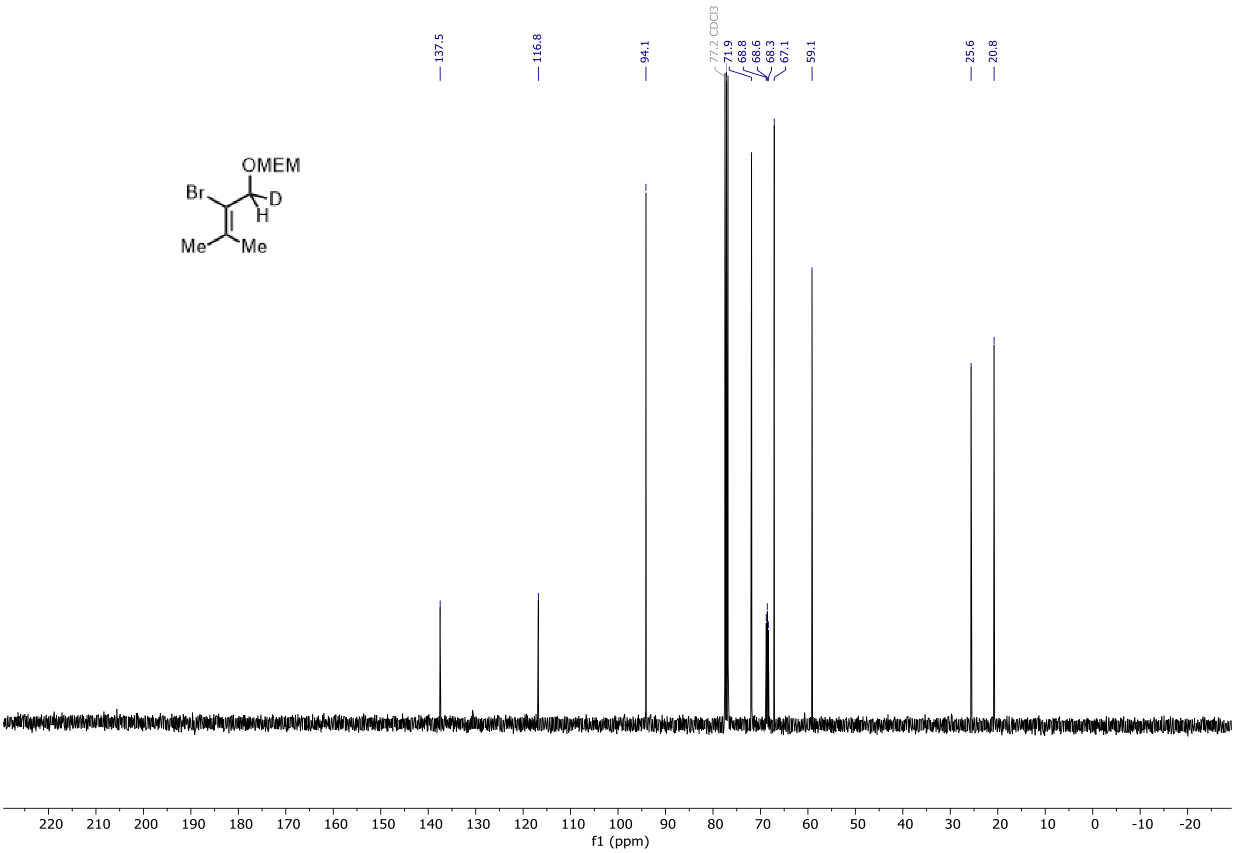

^{1}H NMR (400 MHz, $CDCl_3$) of **S31-*d***

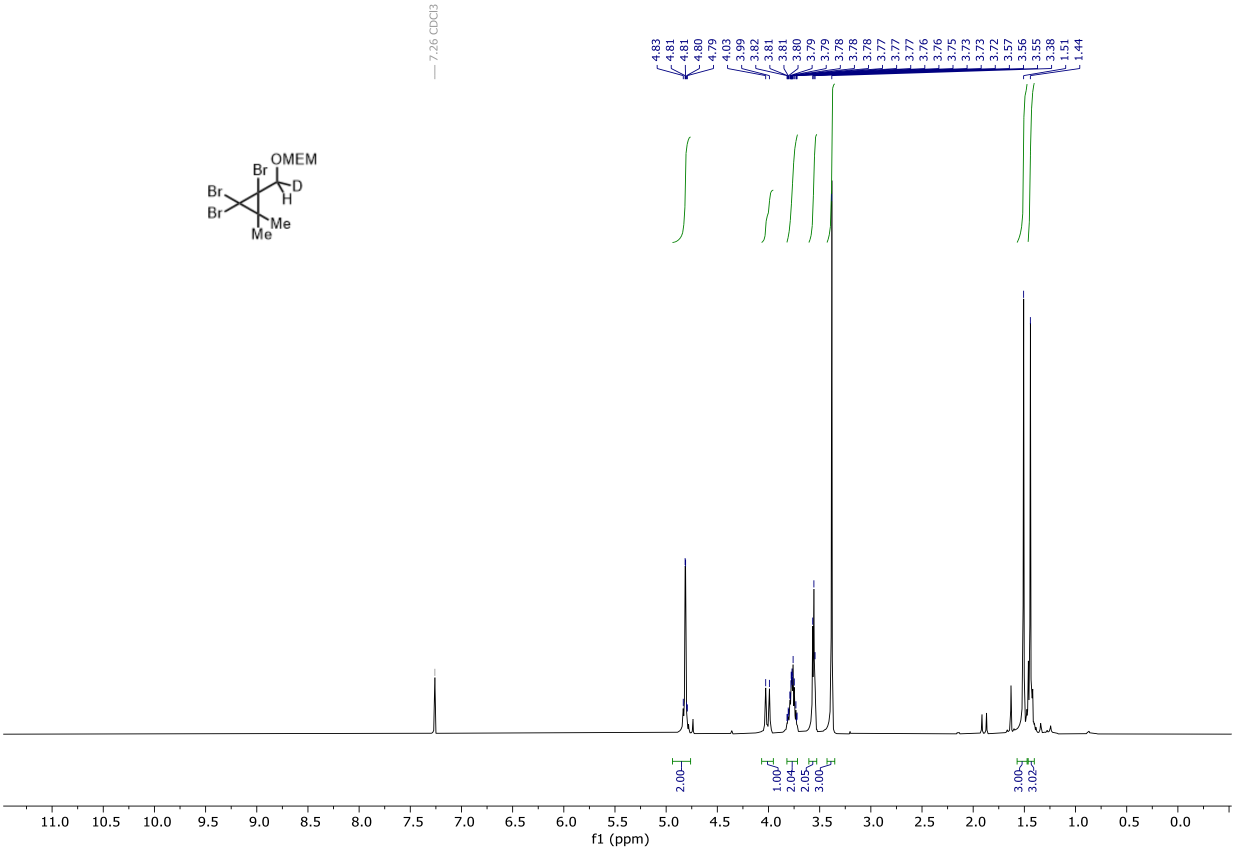

^{13}C NMR (101 MHz, $CDCl_3$) of **S31-*d***

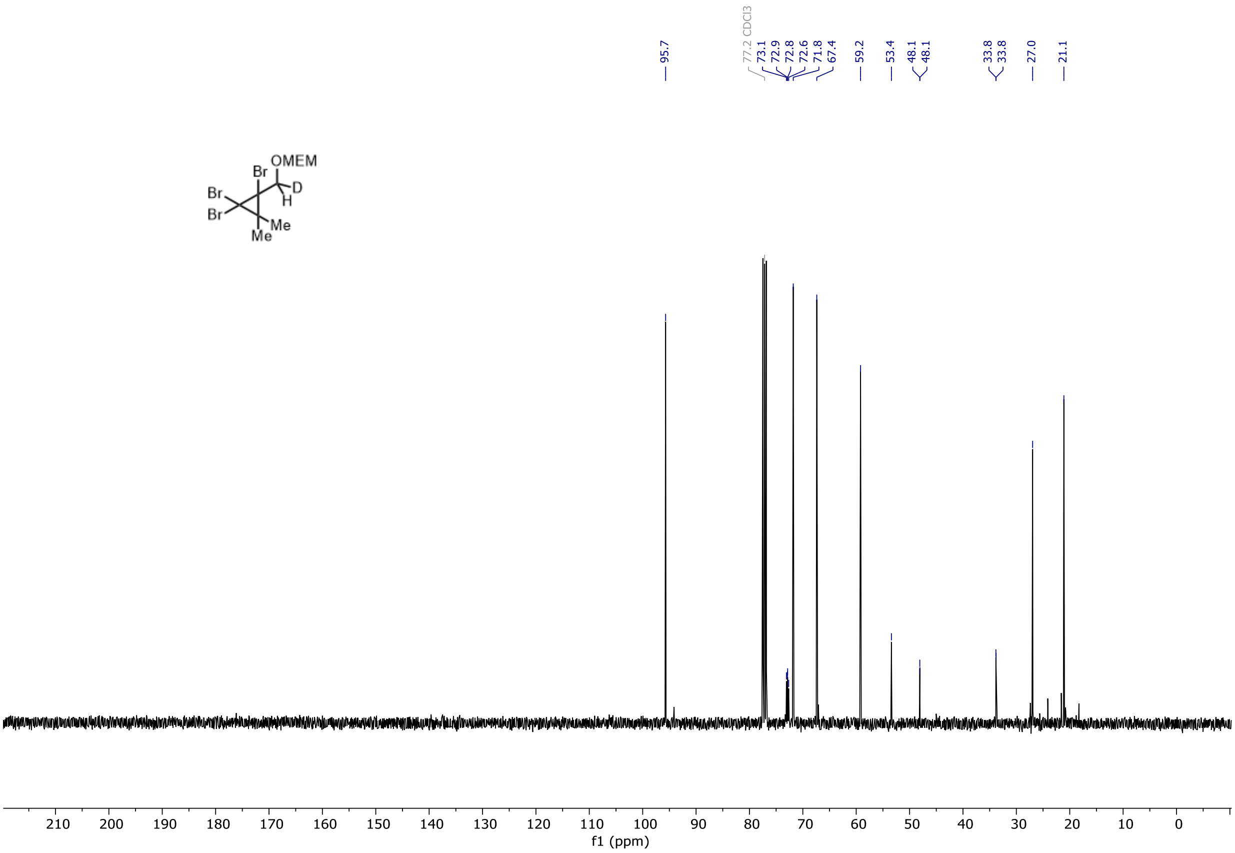

^{1}H NMR (400 MHz, $CDCl_3$) of **33a-*d***

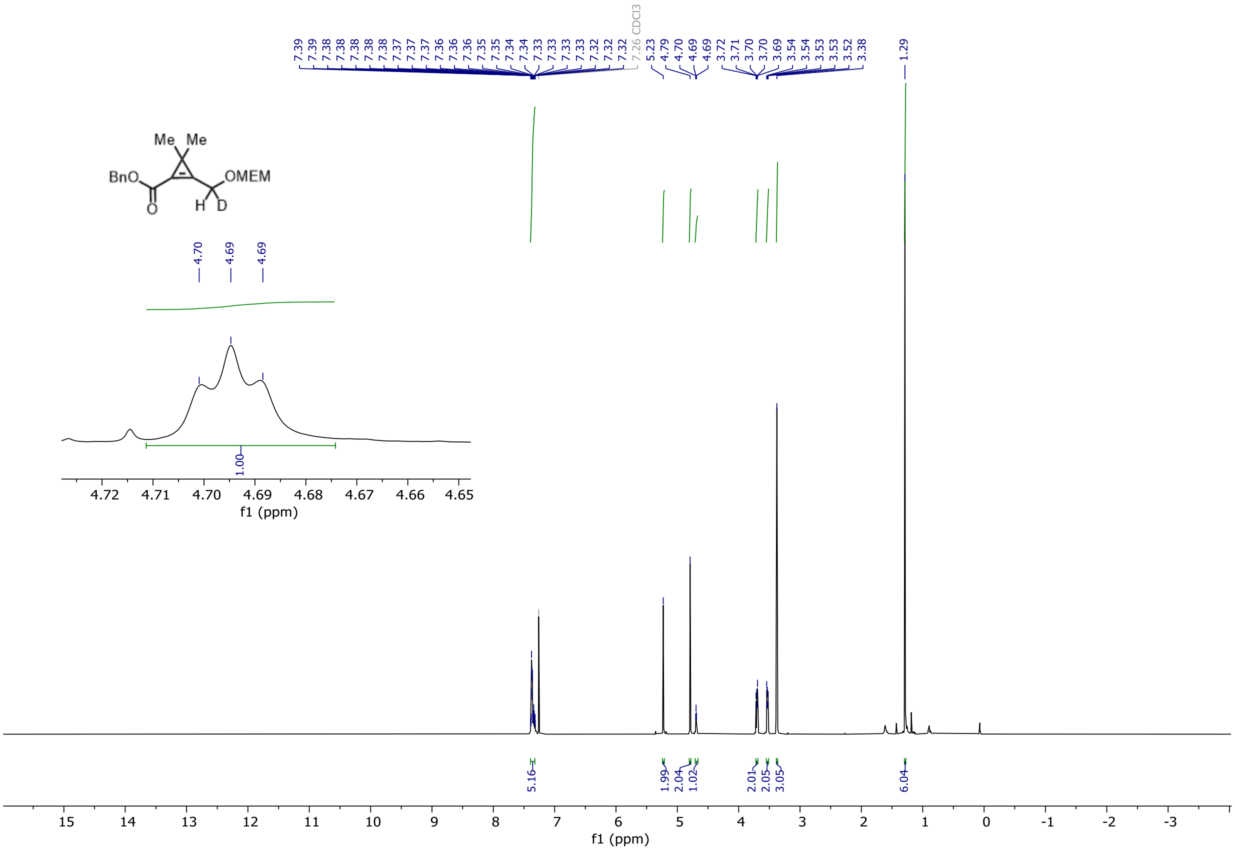

^{13}C NMR (101 MHz, $CDCl_3$) of **33a-*d***

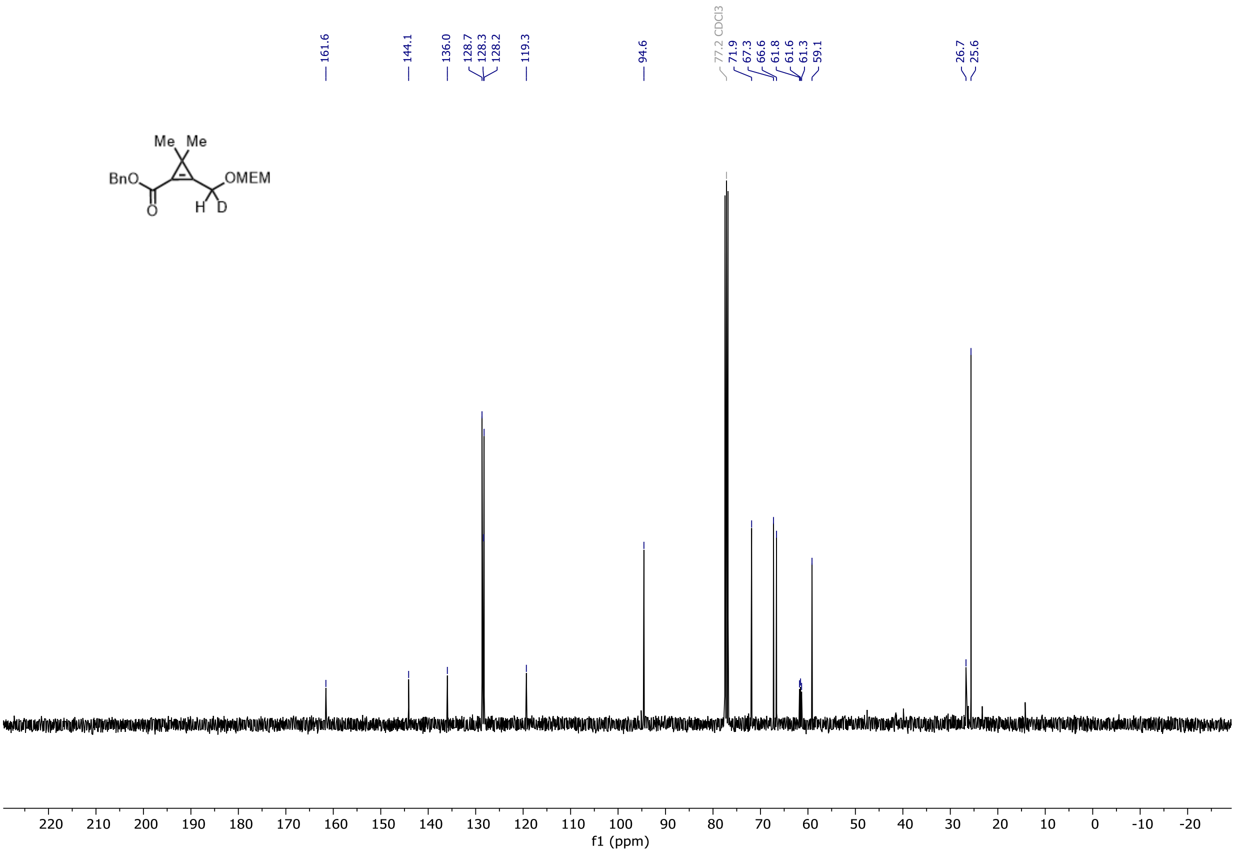

10. HPLC and SFC Traces

2-benzyl 1,1-dimethyl (R)-3-cyclohexylidenecyclopropane-1,1,2-tricarboxylate (3b)

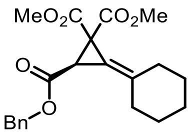

(Chiralpak OG, hexane/isopropanol = 98/2, 1 ml/min)

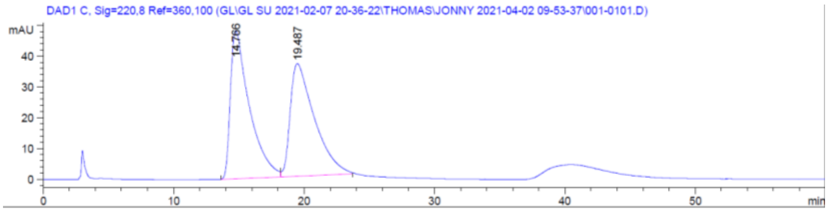

Signal 3: DAD1 C, Sig=220,8 Ref=360,100

Peak #	RetTime [min]	Type	Width [min]	Area [mAU*s]	Height [mAU]	Area %
1	14.766	BB	1.4269	4866.76611	48.16390	50.6775
2	19.487	BB	1.8396	4736.63574	36.55877	49.3225

Totals : 9603.40186 84.72267

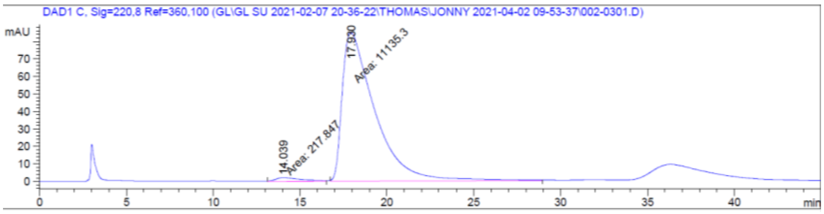

Signal 3: DAD1 C, Sig=220,8 Ref=360,100

Peak #	RetTime [min]	Type	Width [min]	Area [mAU*s]	Height [mAU]	Area %
1	14.039	MM	1.6762	217.84711	2.16602	1.9188
2	17.930	MM	2.1817	1.11353e4	85.06754	98.0812

Totals : 1.13531e4 87.23355

2-benzyl 1,1-dimethyl (R)-3-cyclopentylidenecyclopropane-1,1,2-tricarboxylate (5b)

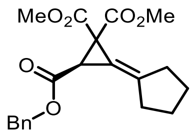

(Chiralpak AD-H, hexane/isopropanol = 98/2, 1 ml/min)

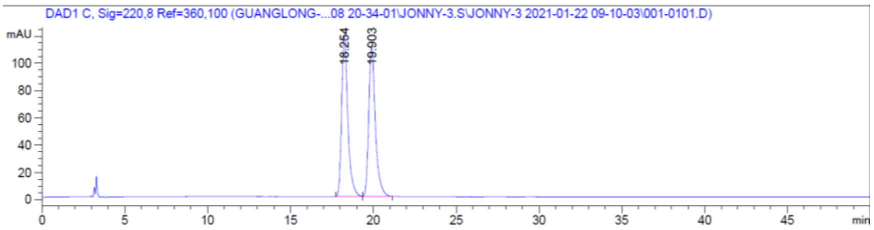

Signal 3: DAD1 C, Sig=220,8 Ref=360,100

Peak #	RetTime [min]	Type	Width [min]	Area [mAU*s]	Height [mAU]	Area %
1	18.254	BV	0.3907	3070.73340	118.36333	49.9651
2	19.903	VB	0.4226	3075.01758	109.83517	50.0349
Totals :				6145.75098	228.19851	

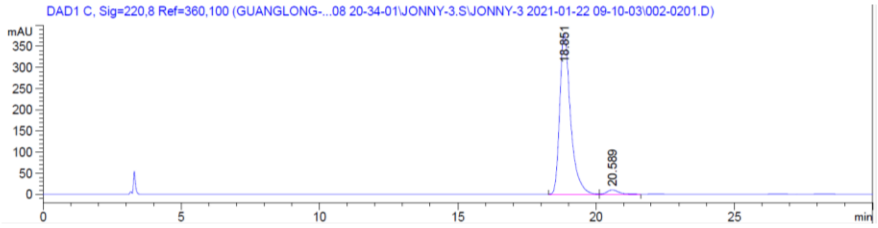

Signal 3: DAD1 C, Sig=220,8 Ref=360,100

Peak #	RetTime [min]	Type	Width [min]	Area [mAU*s]	Height [mAU]	Area %
1	18.851	BV	0.4096	1.03978e4	381.91403	96.9297
2	20.589	VB	0.4564	329.35815	10.84329	3.0703
Totals :				1.07272e4	392.75732	

2-benzyl 1,1-dimethyl (R)-3-(1-(tert-butoxycarbonyl)piperidin-4-ylidene)cyclopropane-1,1,2-tricarboxylate (6b)

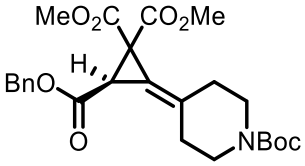

(chiralpak AD-H, hexane/isopropanol = 99/5, 1 ml/min)

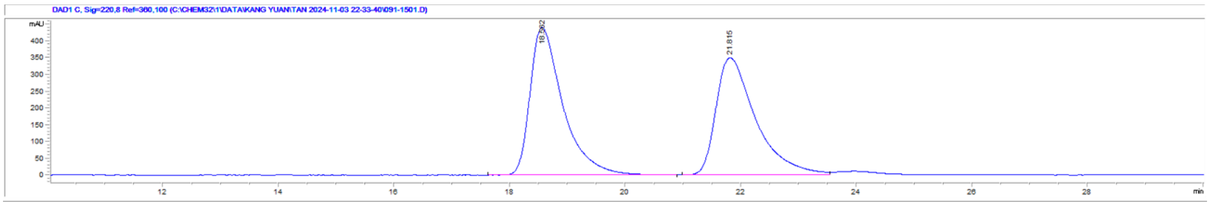

#	Time	Area	Height	Width	Area%	Symmetry
1	18.562	17435	440.4	0.5939	50.888	0.528
2	21.815	16826.7	349.7	0.7293	49.112	0.506

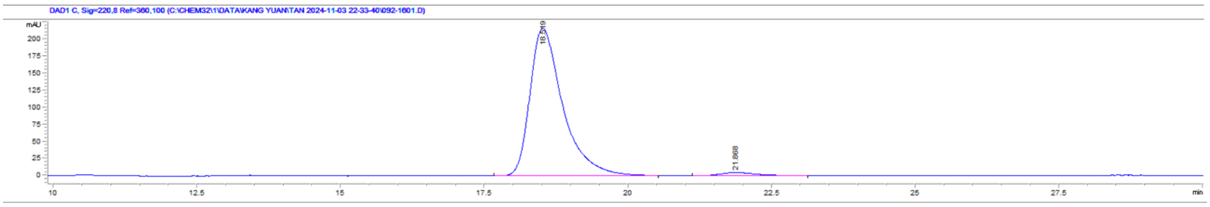

#	Time	Area	Height	Width	Area%	Symmetry
1	18.519	8658.9	216.7	0.5882	97.354	0.595
2	21.868	235.4	4.8	0.5814	2.646	0.643

2-benzyl 1,1-dimethyl (R)-3-(tetrahydro-4H-pyran-4-ylidene)cyclopropane-1,1,2-tricarboxylate (7b)

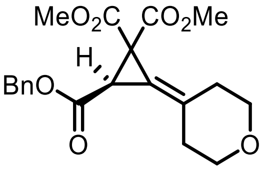

(chiralcel OD, hexane/isopropanol = 95/5, 1 ml/min)

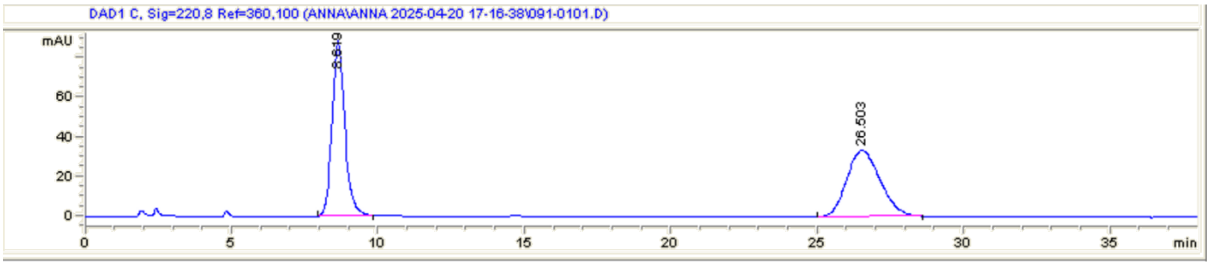

#	Time	Area	Height	Width	Area%	Symmetry
1	8.619	2683.2	88.5	0.4558	50.099	0.818
2	26.503	2672.6	33.5	1.2379	49.901	0.785

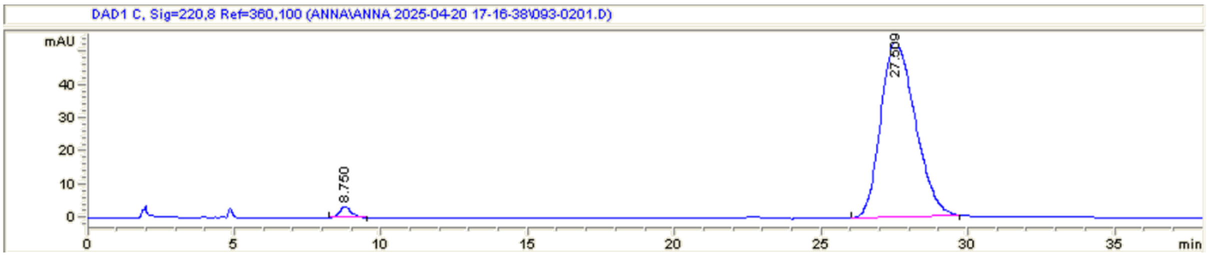

#	Time	Area	Height	Width	Area%	Symmetry
1	8.75	95.8	3.2	0.4533	2.163	0.836
2	27.509	4334.4	52.9	1.2808	97.837	0.715

2-allyl 1,1-dimethyl (R)-3-cyclohexylidenecyclopropane-1,1,2-tricarboxylate (8b)

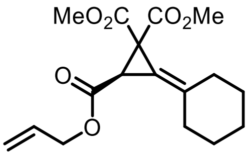

(Chiralpak OG, hexane/isopropanol = 98/2, 1 ml/min)

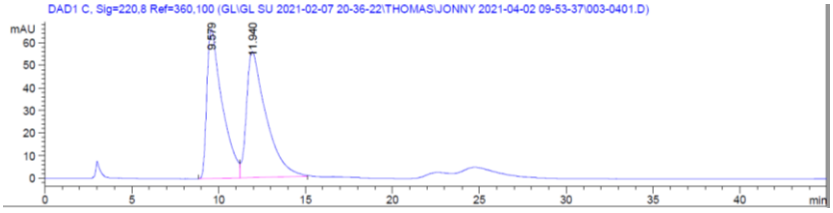

Signal 3: DAD1 C, Sig=220,8 Ref=360,100

Peak #	RetTime [min]	Type	Width [min]	Area [mAU*s]	Height [mAU]	Area %
1	9.579	BV	0.8718	4033.82617	65.93304	48.0134
2	11.940	VB	1.1068	4367.63281	55.70168	51.9866

Totals : 8401.45898 121.63472

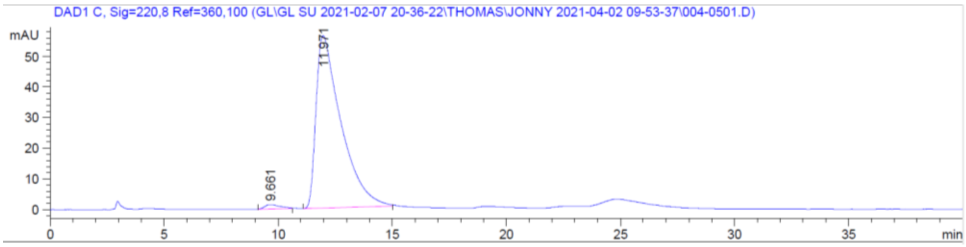

Signal 3: DAD1 C, Sig=220,8 Ref=360,100

Peak #	RetTime [min]	Type	Width [min]	Area [mAU*s]	Height [mAU]	Area %
1	9.661	BB	0.6942	68.61773	1.39911	1.5797
2	11.971	BB	1.0718	4275.02148	56.30739	98.4203

Totals : 4343.63921 57.70650

2-benzyl 1,1-dimethyl (R,Z)-3-butylidenecyclopropane-1,1,2-tricarboxylate (9b)

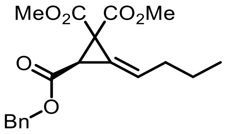

(Chiralpak AS-H, hexane/isopropanol = 90/10, 1 ml/min)

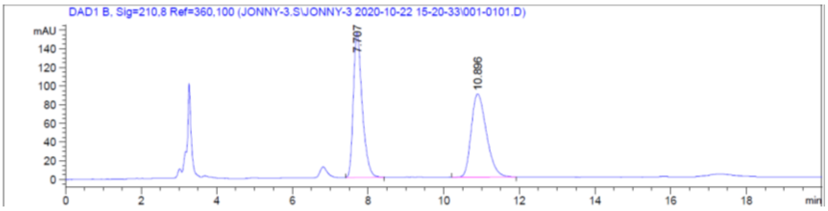

Signal 2: DAD1 B, Sig=210,8 Ref=360,100

Peak #	RetTime [min]	Type	Width [min]	Area [mAU*s]	Height [mAU]	Area %
1	7.707	BB	0.2452	2530.35547	156.02222	50.4109
2	10.896	BB	0.4335	2489.10938	89.22608	49.5891
Totals :				5019.46484	245.24830	

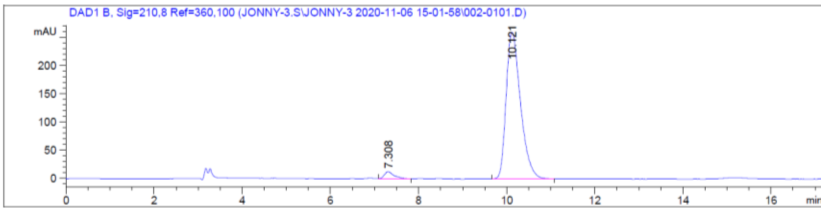

Signal 2: DAD1 B, Sig=210,8 Ref=360,100

Peak #	RetTime [min]	Type	Width [min]	Area [mAU*s]	Height [mAU]	Area %
1	7.308	VV	0.2302	204.88252	12.97821	3.3606
2	10.121	BB	0.3478	5891.71533	260.60773	96.6394
Totals :				6096.59785	273.58593	

Crystal sample (95% ee) crystallised MeOH from 88% ee:

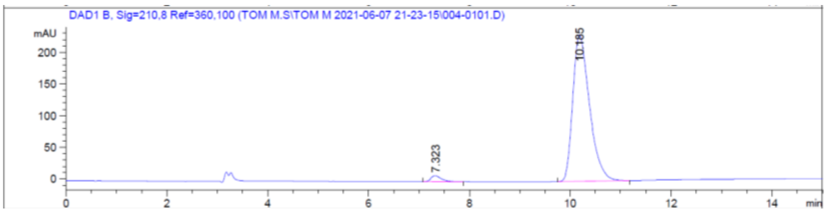

Signal 2: DAD1 B, Sig=210,8 Ref=360,100

Peak #	RetTime [min]	Type	Width [min]	Area [mAU*s]	Height [mAU]	Area %
1	7.323	BB	0.2226	144.21925	9.74714	2.6394
2	10.185	BB	0.3526	5319.88574	231.06537	97.3606
Totals :				5464.10500	240.81250	

trimethyl (R,Z)-3-butylidenecyclopropane-1,1,2-tricarboxylate (10b)

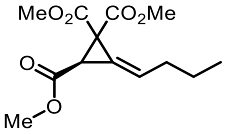

(Chiralcel IC, 1500 psi, 1% to 30% MeOH over 5 min, then from 30% to 50% MeOH in 0.5 min, then hold 50% MeOH for 1.5 min, 1.5 mL/min)

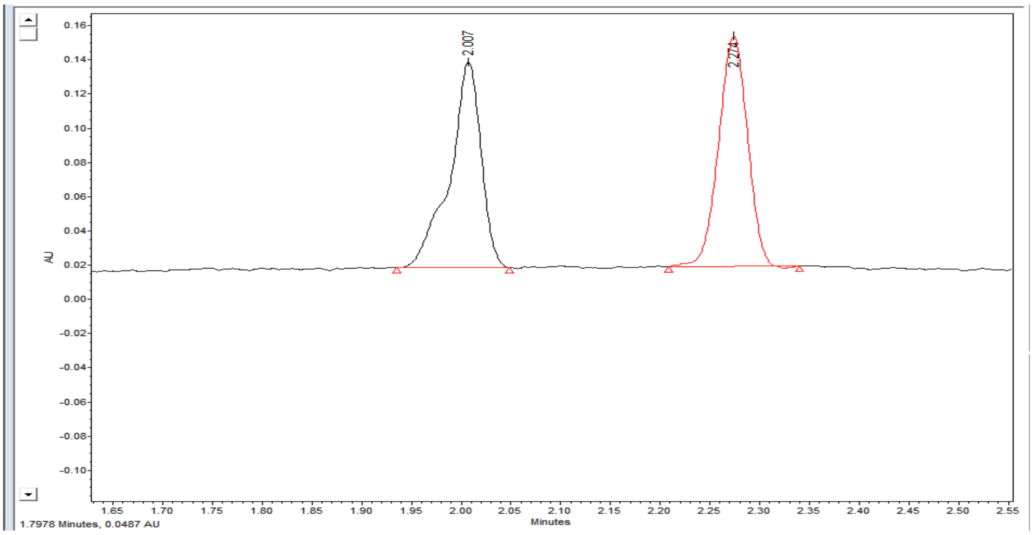

	Name	Retention Time (min)	Purity1 Angle	Purity1 Threshold	PDA/FLR Match1 Spect. Name	PDA/FLR Match1 Angle	PDA/FLR Match1 Threshold	PDA/FLR Match1 Lib. Name	Area (µV*sec)	% Area	Height (µV)	Int Type	Amount	Units	Peak Type
1		2.007							261555	48.88	120267	bb			Unknown
2		2.274							273504	51.12	134441	bb			Unknown

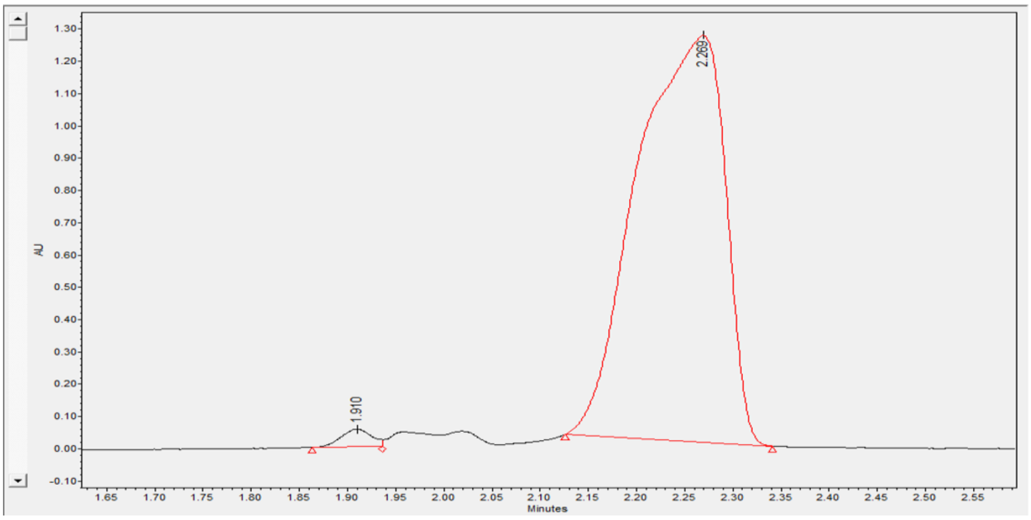

	Name	Retention Time (min)	Purity1 Angle	Purity1 Threshold	PDA/FLR Match1 Spect. Name	PDA/FLR Match1 Angle	PDA/FLR Match1 Threshold	PDA/FLR Match1 Lib. Name	Area (µV*sec)	% Area	Height (µV)	Int Type	Amount	Units	Peak Typ
1		1.910							119476	1.47	54699	bv			Unknown
2		2.269							7994373	98.53	1260145	bb			Unknown

2-isobutyl 1,1-dimethyl (R,Z)-3-butylidenecyclopropane-1,1,2-tricarboxylate (11b)

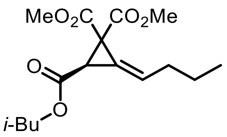

(Chiralpak AS-H, hexane/isopropanol = 99/1, 1 ml/min)

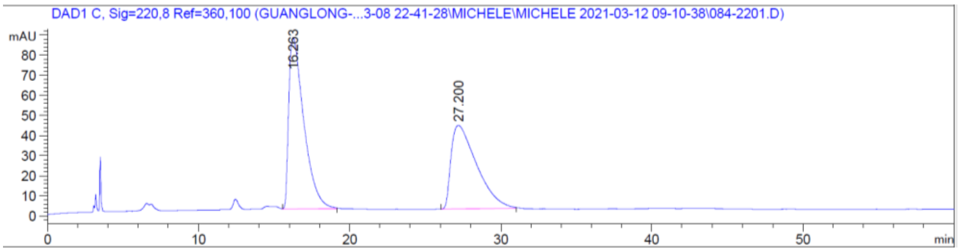

Signal 3: DAD1 C, Sig=220,8 Ref=360,100

Peak #	RetTime [min]	Type	Width [min]	Area [mAU*s]	Height [mAU]	Area %
1	16.263	VB	1.0363	5917.36816	85.20004	54.7382
2	27.200	BB	1.6026	4892.93799	41.52795	45.2618
Totals :				1.08103e4	126.72800	

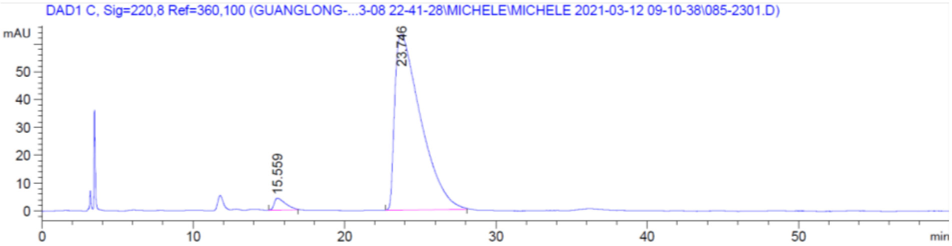

Signal 3: DAD1 C, Sig=220,8 Ref=360,100

Peak #	RetTime [min]	Type	Width [min]	Area [mAU*s]	Height [mAU]	Area %
1	15.559	BB	0.7287	224.28351	4.28443	2.9005
2	23.746	BB	1.6256	7508.24365	63.03869	97.0995
Totals :				7732.52716	67.32312	

2-benzyl 1,1-dimethyl (R,Z)-3-(3-chloropropylidene)cyclopropane-1,1,2-tricarboxylate (12b)

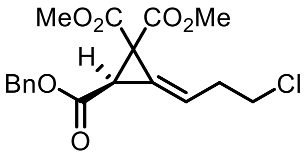

(chiralcel OD, hexane/isopropanol = 95/5, 1 ml/min)

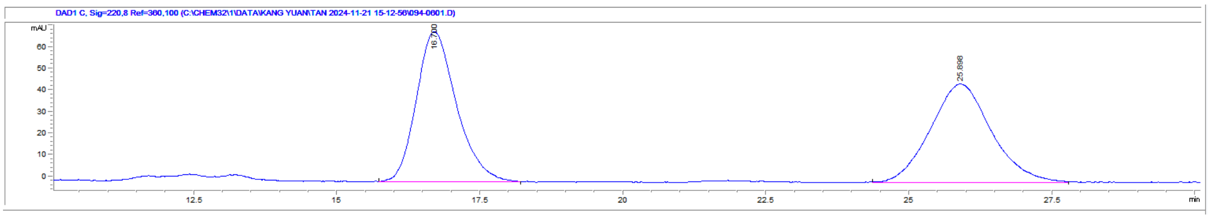

#	Time	Area	Height	Width	Area%	Symmetry
1	16.7	3401.4	69.7	0.7111	49.892	0.76
2	25.898	3416.1	45.7	0.9372	50.108	0.943

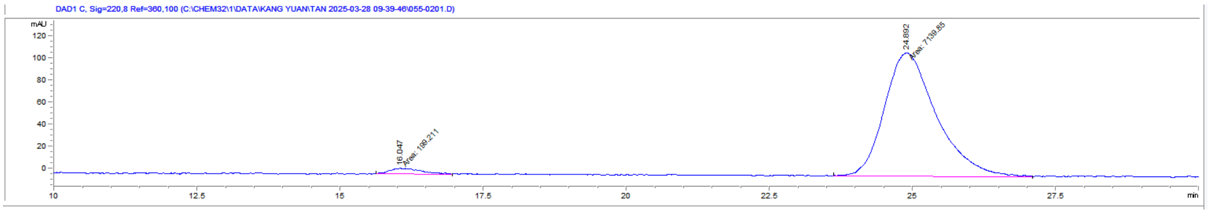

#	Time	Area	Height	Width	Area%	Symmetry
1	16.047	199.2	5.2	0.6447	2.714	0.466
2	24.892	7139.9	112.1	1.0617	97.286	0.72

2-benzyl 1,1-dimethyl (R,Z)-3-(3-((tert-butyldimethylsilyl)oxy)propylidene)cyclopropane-1,1,2-tricarboxylate (13b)

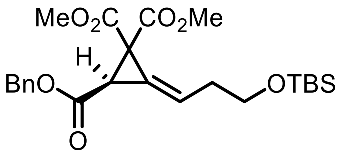

(chiralcel OD, hexane/isopropanol = 99/1, 1 ml/min)

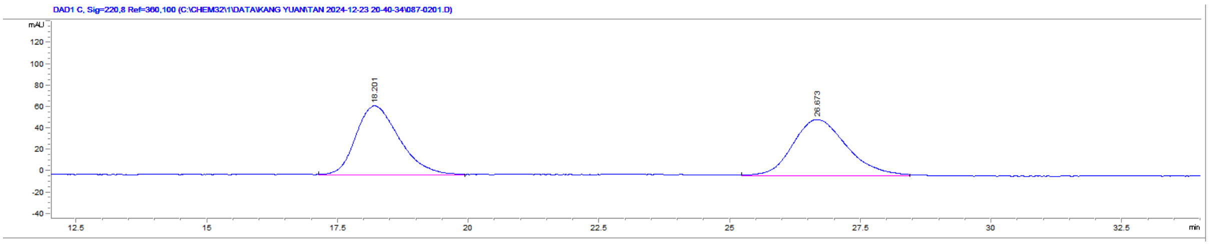

#	Time	Area	Height	Width	Area%	Symmetry
1	18.201	3782.9	64.8	0.7214	49.669	0.714
2	26.673	3833.3	52.8	0.8808	50.331	0.81

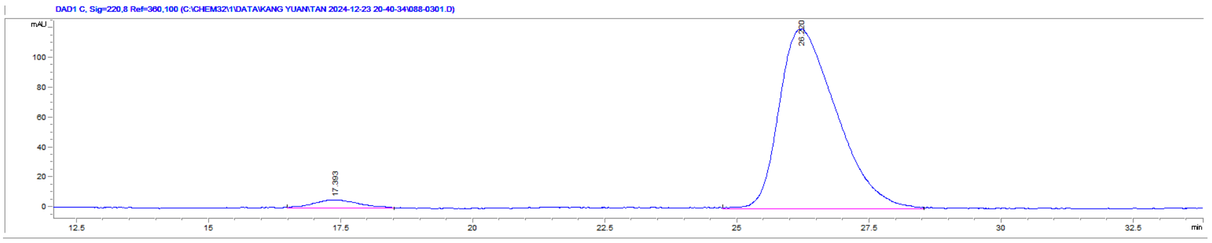

#	Time	Area	Height	Width	Area%	Symmetry
1	17.393	382.7	5.9	0.7784	4.036	0.856
2	26.22	9099.9	120.4	0.9336	95.964	0.638

2-benzyl 1,1-dimethyl (R,Z)-3-(3-phenylpropylidene)cyclopropane-1,1,2-tricarboxylate (14b)

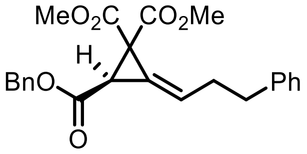

(chiralpak AD-H, hexane/isopropanol = 95/5, 1 ml/min)

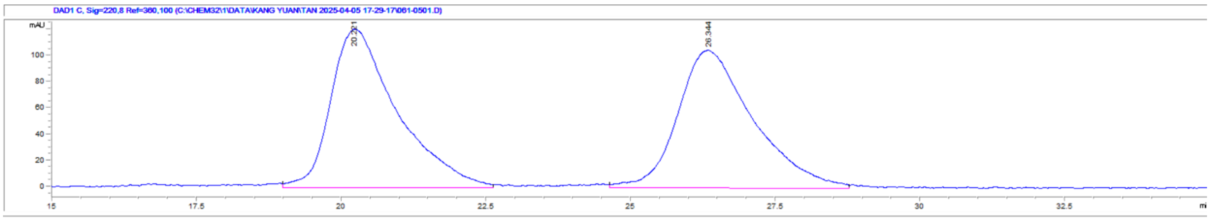

#	Time	Area	Height	Width	Area%	Symmetry
1	20.221	9739.9	121.4	0.9703	50.344	0.519
2	26.344	9606.9	105.6	1.0779	49.656	0.681

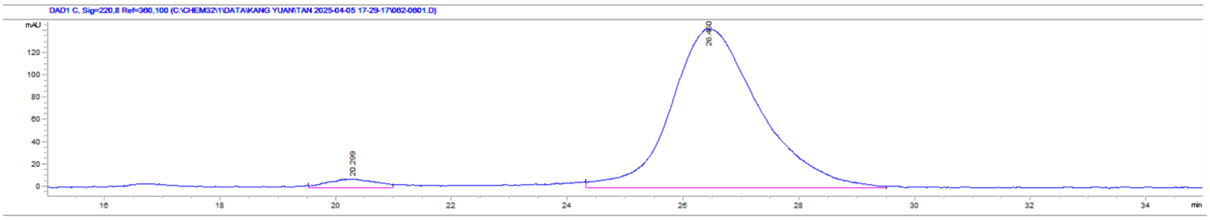

#	Time	Area	Height	Width	Area%	Symmetry
1	20.299	479.4	8	0.7082	3.063	1.051
2	26.45	15171.7	142.9	1.287	96.937	0.757

2-benzyl 1,1-dimethyl (R,Z)-3-ethylidenecyclopropane-1,1,2-tricarboxylate (15b)

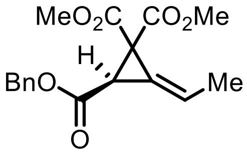

(chiralcel OD, hexane/isopropanol = 95/5, 1 ml/min)

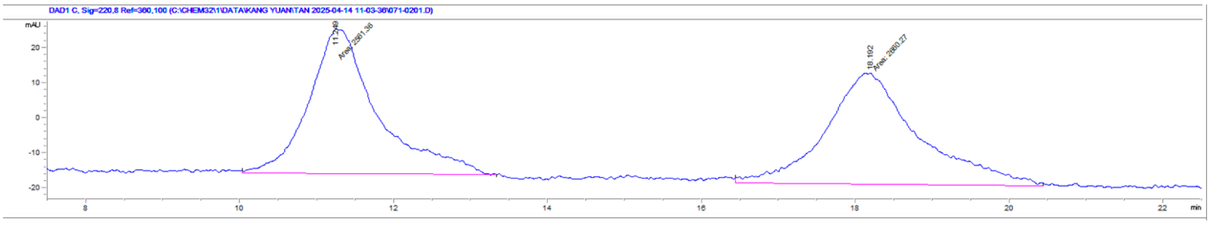

#	Time	Area	Height	Width	Area%	Symmetry
1	11.249	2561.4	41.4	1.0312	49.053	0.652
2	18.192	2660.3	31.7	1.3997	50.947	0.882

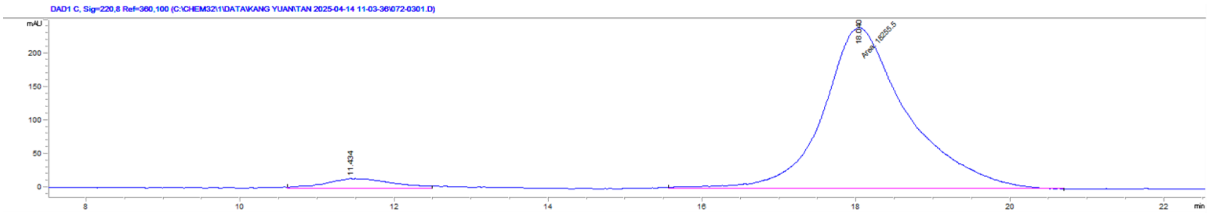

#	Time	Area	Height	Width	Area%	Symmetry
1	11.434	949.7	15.1	0.7432	4.945	0.604
2	18.04	18255.5	240.2	1.2669	95.055	0.734

2-benzyl 1,1-dimethyl (R,Z)-3-(2-((tert-butyldimethylsilyl)oxy)ethylidene)cyclopropane-1,1,2-tricarboxylate (16b)

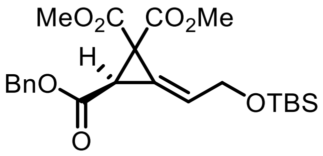

(chiralpak AD-H, hexane/isopropanol = 99/1, 1 ml/min)

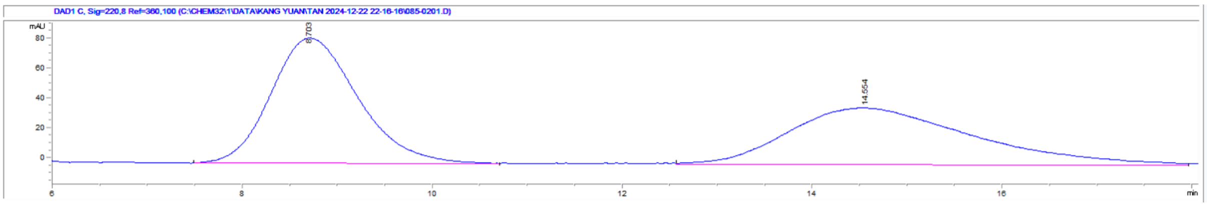

#	Time	Area	Height	Width	Area%	Symmetry
1	8.703	5359.3	83.8	0.9363	50.783	0.759
2	14.554	5194.1	38.2	1.6032	49.217	0.682

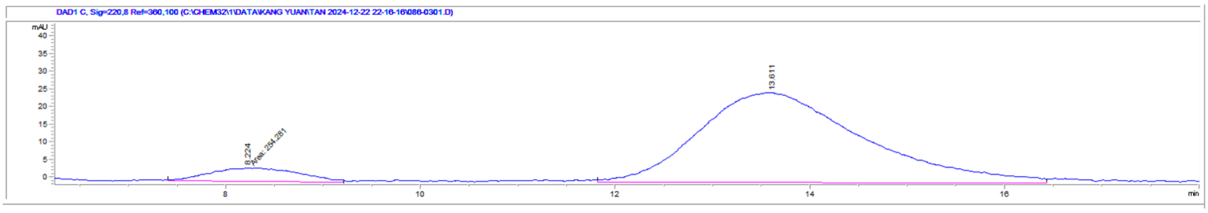

#	Time	Area	Height	Width	Area%	Symmetry
1	8.224	254.3	4	1.0681	7.821	0.755
2	13.611	2997.1	25.4	1.3869	92.179	0.776

2-benzyl 1,1-dimethyl (R,Z)-3-(2-phenylethylidene)cyclopropane-1,1,2-tricarboxylate (17b)

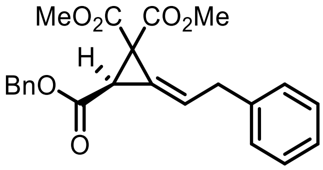

(chiralcel OD, hexane/isopropanol = 95/5, 1 ml/min)

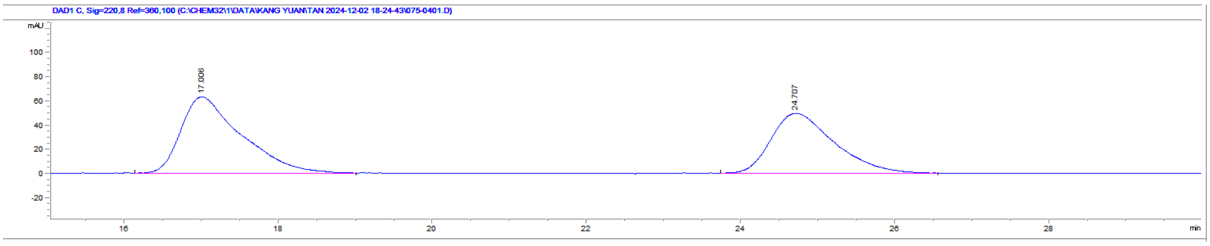

#	Time	Area	Height	Width	Area%	Symmetry
1	17.006	3495.3	63.5	0.7701	54.787	0.49
2	24.707	2884.4	49.8	0.8219	45.213	0.587

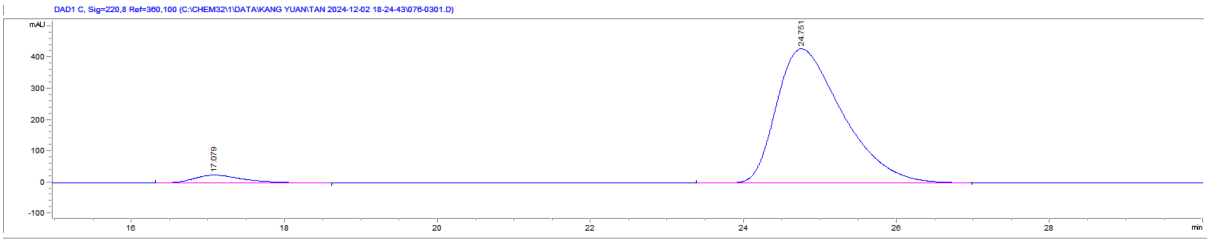

#	Time	Area	Height	Width	Area%	Symmetry
1	17.079	1143	25.4	0.6374	4.248	0.66
2	24.751	25765.5	430.1	0.8929	95.752	0.567

2-benzyl 1,1-diethyl (R,Z)-3-butylidenecyclopropane-1,1,2-tricarboxylate (18b)

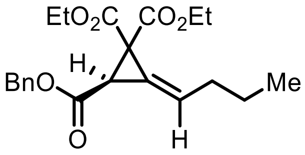

(chiralcel OD, hexane/isopropanol = 99/1, 1 ml/min)

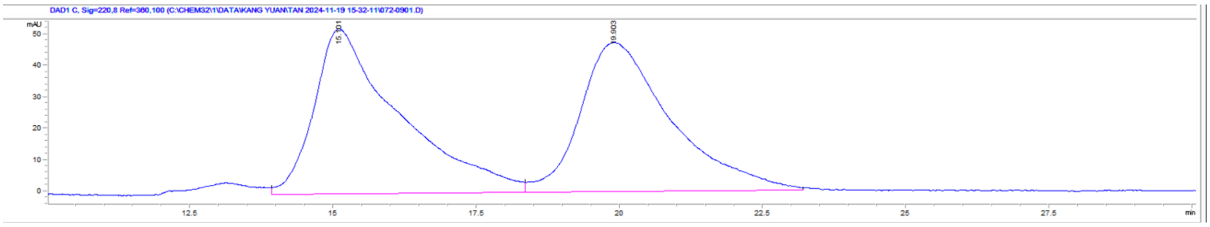

#	Time	Area	Height	Width	Area%	Symmetry
1	15.101	5492	52.6	1.3282	51.033	0.389
2	19.903	5269.7	47.5	1.3191	48.967	0.573

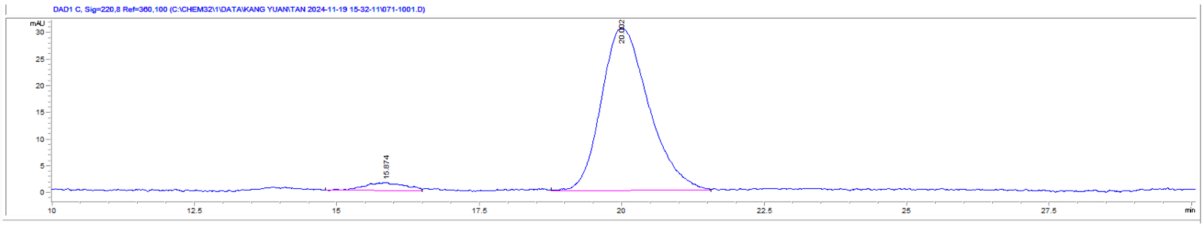

#	Time	Area	Height	Width	Area%	Symmetry
1	15.874	74.7	1.5	0.5863	4.051	1.061
2	20.002	1770.2	30.5	0.7563	95.949	0.729

2-benzyl 1,1-diisopropyl (R,Z)-3-butylidenecyclopropane-1,1,2-tricarboxylate (19b)

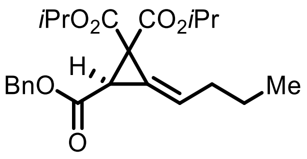

(chiralpak AD-H, hexane/isopropanol = 98/2, 1 ml/min)

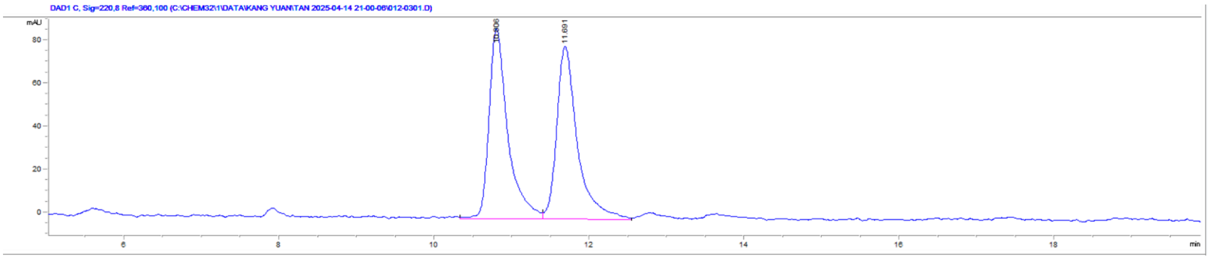

#	Time	Area	Height	Width	Area%	Symmetry
1	10.806	1503.4	88.7	0.2518	50.076	0.61
2	11.691	1498.8	80.3	0.2738	49.924	0.623

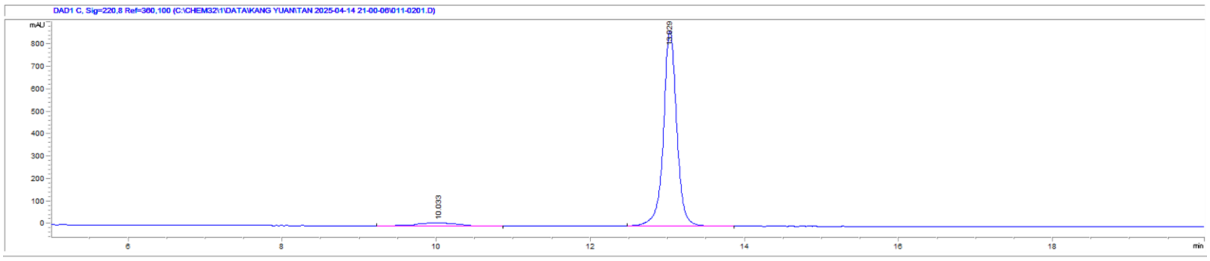

#	Time	Area	Height	Width	Area%	Symmetry
1	10.033	539.3	14.8	0.4658	4.866	1.193
2	13.029	10543.6	872.6	0.183	95.134	0.926

2-benzyl 1,1-di-tert-butyl (R,Z)-3-butylidenecyclopropane-1,1,2-tricarboxylate (20b)

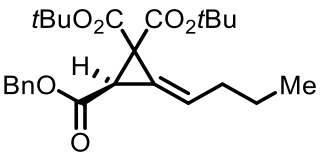

(SFC Chiralcel IC, 1500 psi, from 1% to 30% IPA in 5 min, 1.5 ml/min)

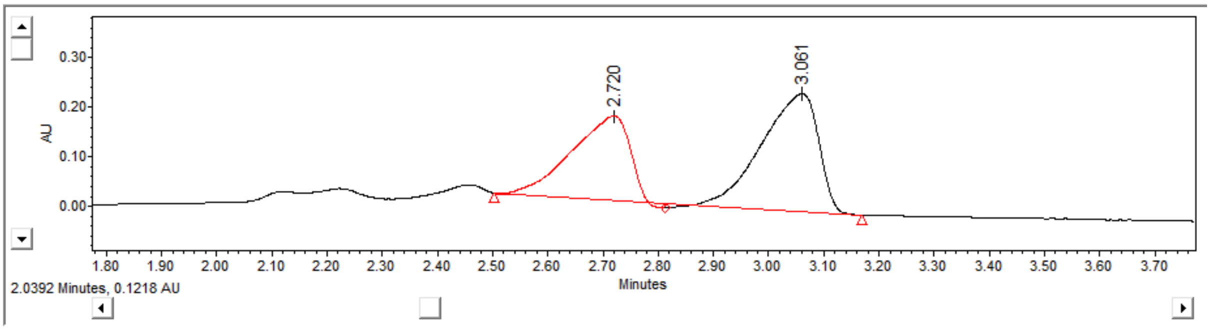

	Name	Retention Time (min)	Purity1 Angle	Purity1 Threshold	PDA/FLR Match1 Spect. Name	PDA/FLR Match1 Angle	PDA/FLR Match1 Threshold	PDA/FLR Match1 Lib. Name	Area (µV*sec)	% Area	Height (µV)	Int Type	Amount	Units	Peak Typ
1		2.720							1259944	42.32	170669	bv			Unknown
2		3.061							1717301	57.68	238039	vb			Unknown

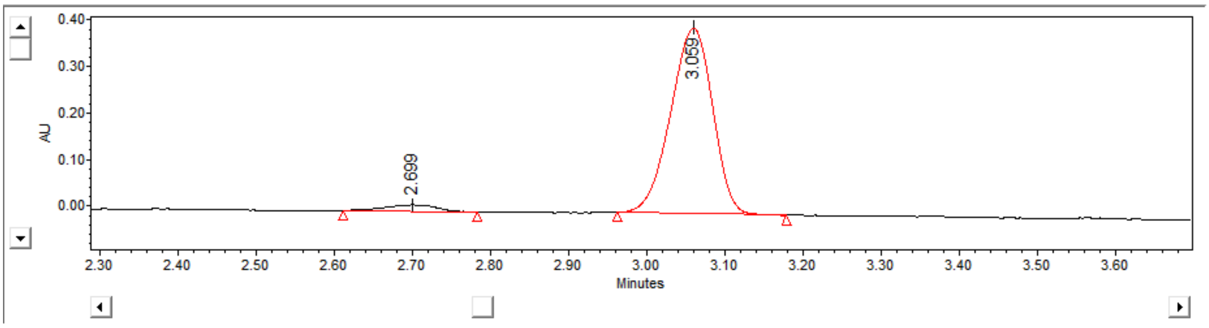

	Name	Retention Time (min)	Purity1 Angle	Purity1 Threshold	PDA/FLR Match1 Spect. Name	PDA/FLR Match1 Angle	PDA/FLR Match1 Threshold	PDA/FLR Match1 Lib. Name	Area (µV*sec)	% Area	Height (µV)	Int Type	Amount	Units	Peak Typ
1		2.699							64326	4.14	14193	bb			Unknown
2		3.059							1489533	95.86	398462	bb			Unknown

tribenzyl (R,Z)-3-butylidenecyclopropane-1,1,2-tricarboxylate (21b)

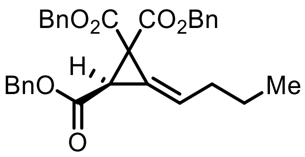

(chiralpak IA, hexane/isopropanol = 98/2, 1 ml/min)

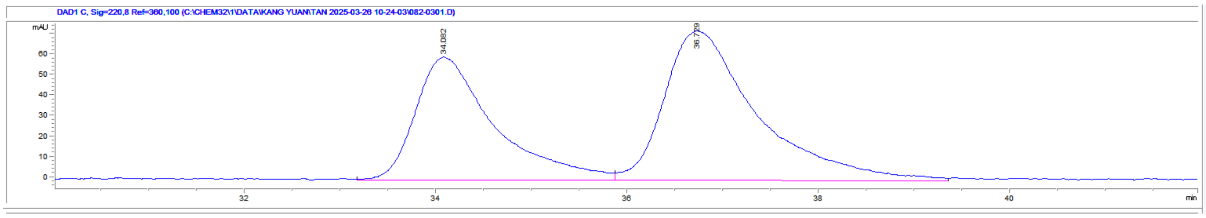

#	Time	Area	Height	Width	Area%	Symmetry
1	34.082	3495.8	60.5	0.8106	44.267	0.524
2	36.728	4401.2	70.9	1.0344	55.733	0.556

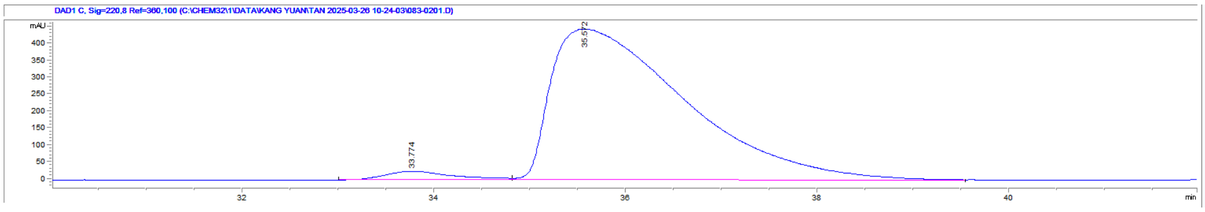

#	Time	Area	Height	Width	Area%	Symmetry
1	33.774	1358.9	26.2	0.6661	3.011	0.628
2	35.572	43774.9	447	1.1906	96.989	0.31

benzyl (S)-2,2-dimethyl-3-methylenecyclopropane-1-carboxylate (22b)

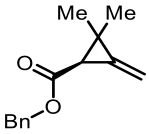

(Chiralpak IA, hexane, 1 ml/min)

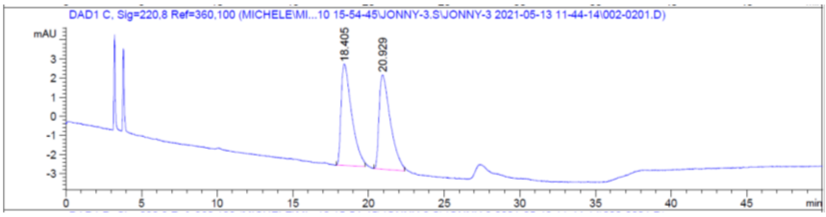

Signal 3: DAD1 C, Sig=220,8 Ref=360,100

Peak #	RetTime [min]	Type	Width [min]	Area [mAU*s]	Height [mAU]	Area %
1	18.405	BB	0.6639	256.34949	5.30362	50.2438
2	20.929	BB	0.7404	253.86140	4.91673	49.7562
Totals :				510.21089	10.22035	

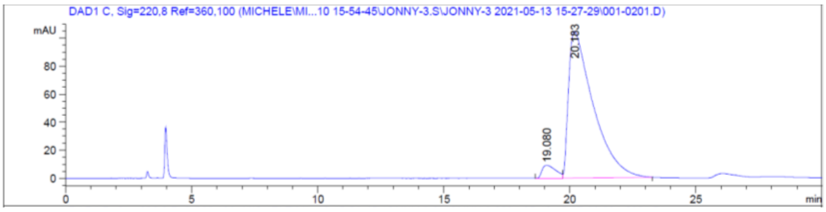

Signal 3: DAD1 C, Sig=220,8 Ref=360,100

Peak #	RetTime [min]	Type	Width [min]	Area [mAU*s]	Height [mAU]	Area %
1	19.080	BV	0.5636	345.52716	9.21251	4.6184
2	20.183	VB	0.9720	7135.96924	104.85372	95.3816
Totals :				7481.49640	114.06623	

2-benzyl 1,1-dimethyl (2R,3R)-3-((E)-3-oxo-3-phenylprop-1-en-1-yl)cyclopropane-1,1,2-tricarboxylate (23b)

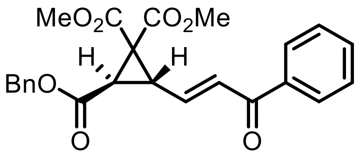

(chiralcel OD, hexane/isopropanol = 95/5, 1 ml/min)

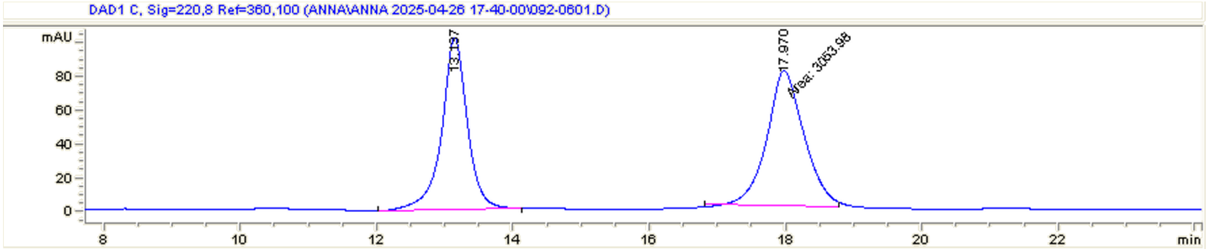

#	Time	Area	Height	Width	Area%	Symmetry
1	13.137	2714.4	102.1	0.3882	47.056	1.011
2	17.97	3054	80.2	0.6348	52.944	0.861

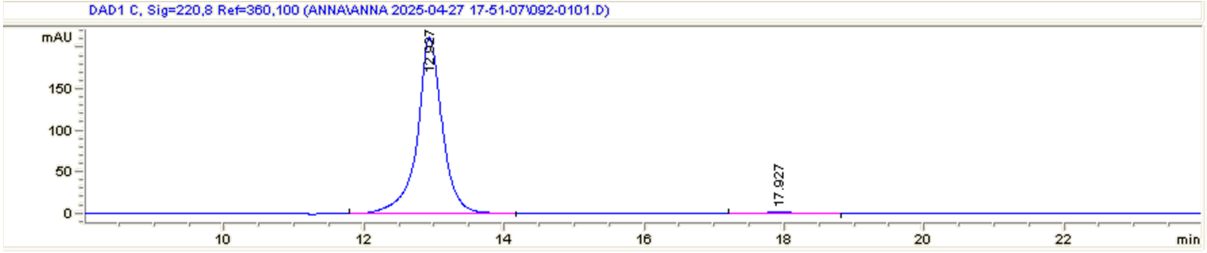

#	Time	Area	Height	Width	Area%	Symmetry
1	12.927	5338.7	212.4	0.3652	98.808	0.961
2	17.927	64.4	1.8	0.5095	1.192	1.13

trimethyl (R,Z)-3-ethylidenecyclopropane-1,1,2-tricarboxylate (24b)

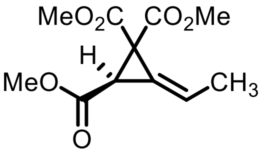

(chiralpak IB, hexane/isopropanol = 99.5/0.5-70/30, 1 ml/min)

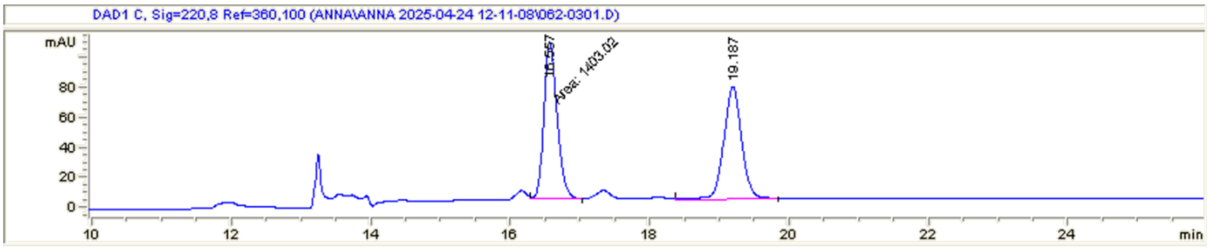

#	Time	Area	Height	Width	Area%	Symmetry
1	16.557	1403	104.8	0.223	50.570	0.725
2	19.187	1371.4	75	0.2773	49.430	1.024

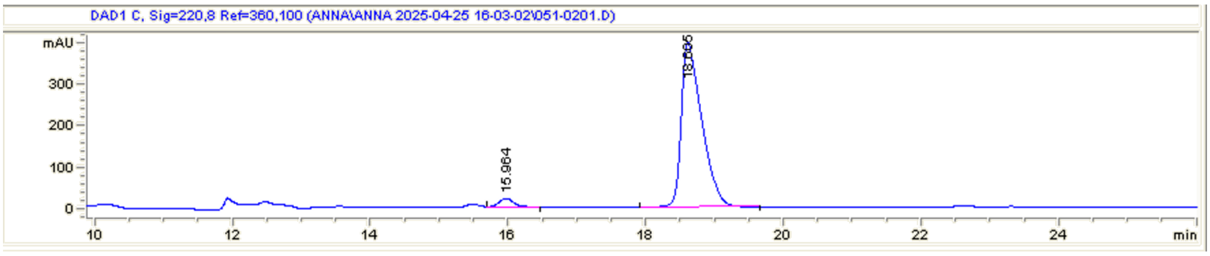

#	Time	Area	Height	Width	Area%	Symmetry
1	15.964	422.7	23.4	0.2627	4.857	0.774
2	18.605	8279.2	395.9	0.314	95.143	0.453

dimethyl (R,Z)-2-(tert-butylcarbamoyl)-3-butylidenecyclopropane-1,1-dicarboxylate (25b)

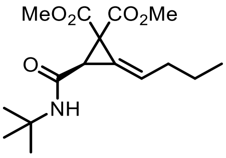

(chiralpak AD, hexane/isopropanol = 99.5/0.5-70/30, 1 ml/min)

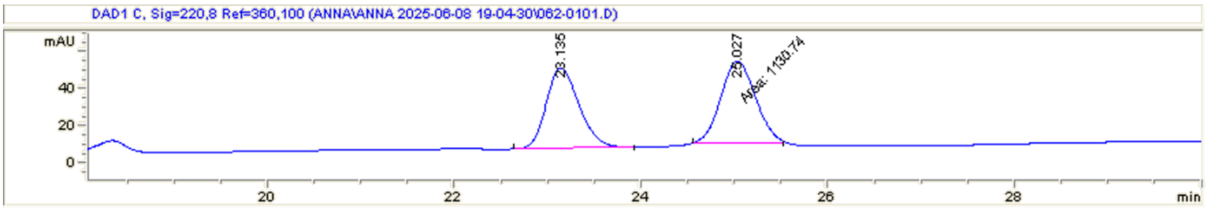

#	Time	Area	Height	Width	Area%	Symmetry
1	23.135	1069.4	43.3	0.38	48.605	0.74
2	25.027	1130.7	44.1	0.4276	51.395	0.897

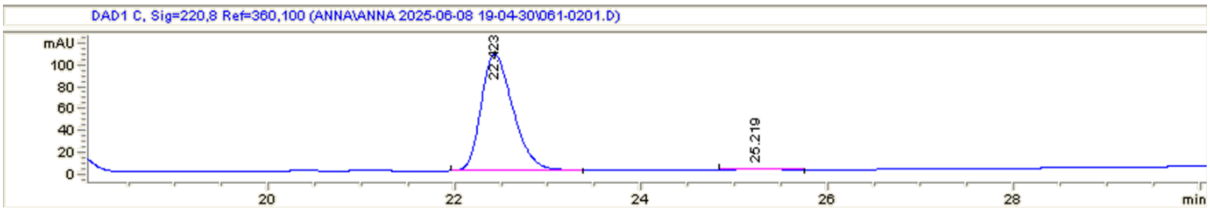

#	Time	Area	Height	Width	Area%	Symmetry
1	22.423	2520.2	106.2	0.3689	99.159	0.69
2	25.219	21.4	1	0.32	0.841	0.722

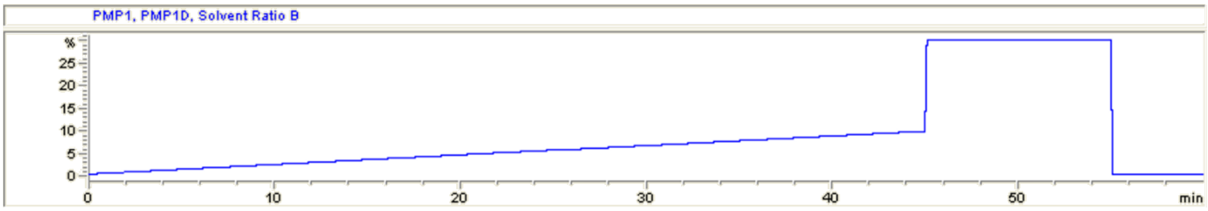

dimethyl (R,Z)-2-(allylcarbamoyl)-3-butylidenecyclopropane-1,1-dicarboxylate (26b)

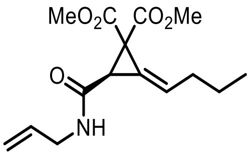

(Chiralpak AS-H, hexane/isopropanol = 90/10 to 70/30, 1 ml/min)

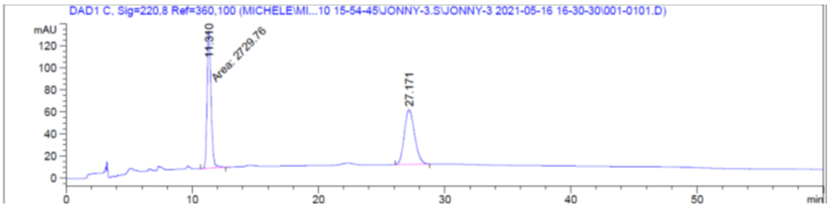

Signal 3: DAD1 C, Sig=220,8 Ref=360,100

Peak #	RetTime [min]	Type	Width [min]	Area [mAU*s]	Height [mAU]	Area %
1	11.310	MM	0.3645	2729.76245	124.81705	49.1081
2	27.171	BB	0.8867	2828.91357	49.80587	50.8919
Totals :				5558.67603	174.62292	

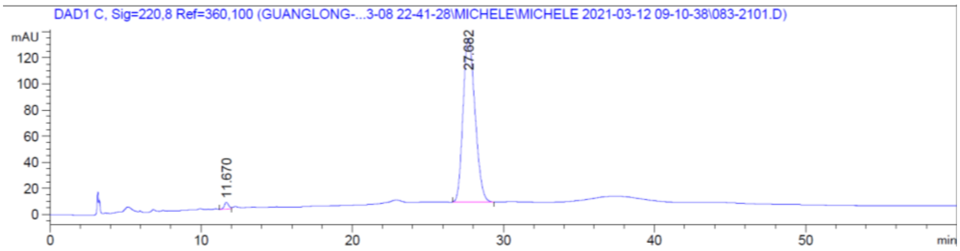

Signal 3: DAD1 C, Sig=220,8 Ref=360,100

Peak #	RetTime [min]	Type	Width [min]	Area [mAU*s]	Height [mAU]	Area %
1	11.670	VV	0.3040	97.59658	4.99405	1.3925
2	27.682	BB	0.8621	6911.01953	125.57695	98.6075
Totals :				7008.61611	130.57100	

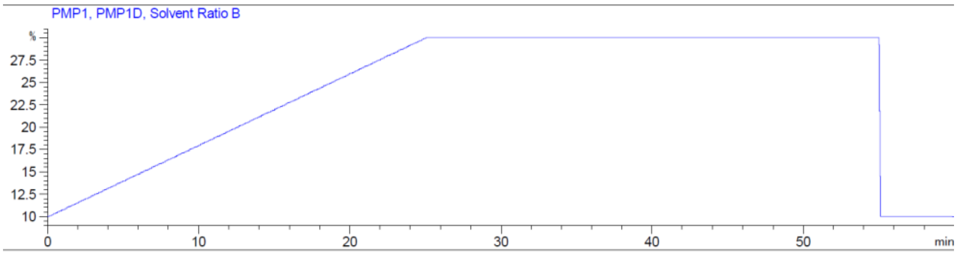

dimethyl (R)-2-(tert-butylcarbamoyl)-3-cyclopentylidenecyclopropane-1,1-dicarboxylate (27b)

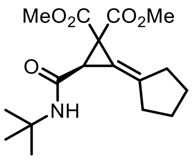

(Chiralpak AD-H, hexane/isopropanol = 98/2, 1 ml/min)

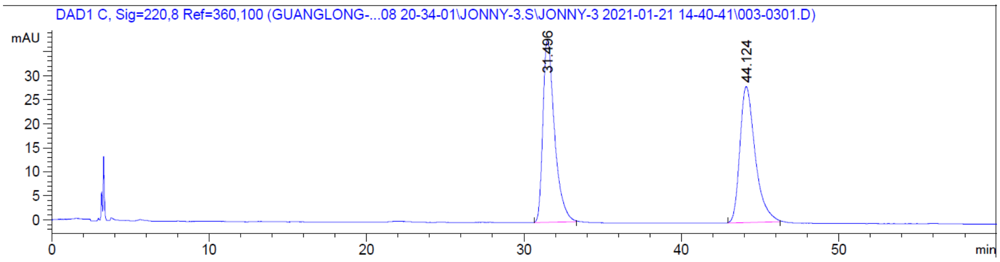

Signal 3: DAD1 C, Sig=220,8 Ref=360,100

Peak #	RetTime [min]	Type	Width [min]	Area [mAU*s]	Height [mAU]	Area %
1	31.496	BB	0.7793	1948.88782	38.06321	50.3962
2	44.124	BB	1.0248	1918.24524	28.29990	49.6038
Totals :				3867.13306	66.36311	

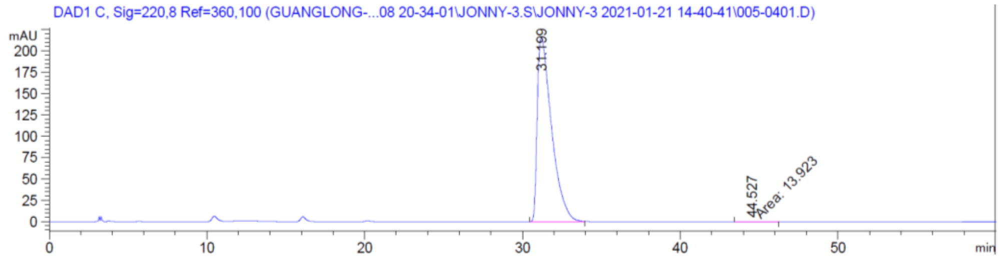

Signal 3: DAD1 C, Sig=220,8 Ref=360,100

Peak #	RetTime [min]	Type	Width [min]	Area [mAU*s]	Height [mAU]	Area %
1	31.199	BB	0.9508	1.36048e4	216.00661	99.8978
2	44.527	MM	1.1366	13.92300	2.04167e-1	0.1022
Totals :				1.36187e4	216.21077	

(S)-N-(tert-butyl)-2,2-dimethyl-3-methylenecyclopropane-1-carboxamide (28b)

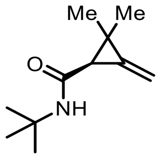

(Chiralpak AS-H, hexane/isopropanol = 98/2, 1 ml/min)

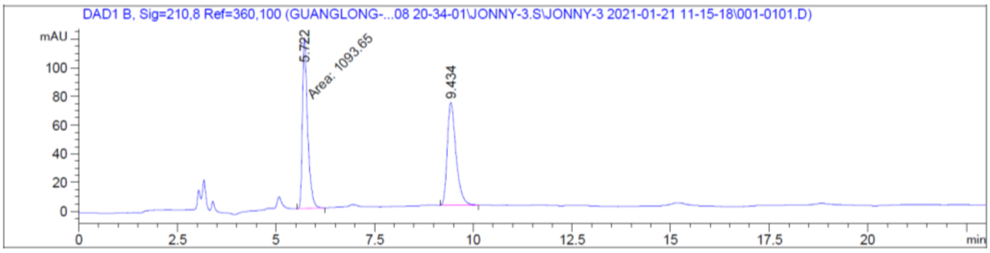

Signal 2: DAD1 B, Sig=210,8 Ref=360,100

Peak #	RetTime [min]	Type	Width [min]	Area [mAU*s]	Height [mAU]	Area %
1	5.722	MM	0.1528	1093.65137	119.27623	49.7836
2	9.434	BB	0.2336	1103.15820	71.66758	50.2164
Totals :				2196.80957	190.94381	

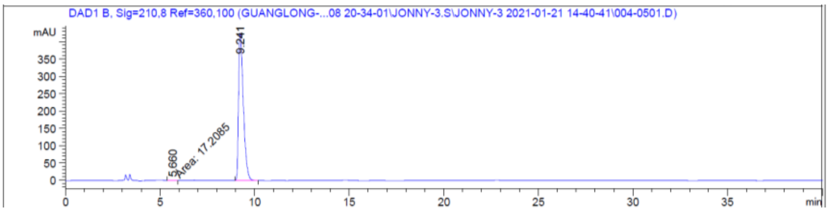

Signal 2: DAD1 B, Sig=210,8 Ref=360,100

Peak #	RetTime [min]	Type	Width [min]	Area [mAU*s]	Height [mAU]	Area %
1	5.660	MM	0.4081	17.20852	7.02781e-1	0.2292
2	9.241	BB	0.2693	7490.98730	425.66281	99.7708
Totals :				7508.19582	426.36559	

dimethyl (R,Z)-2-butylidene-3-((4-methoxyphenyl)carbamoyl)cyclopropane-1,1-dicarboxylate (29b)

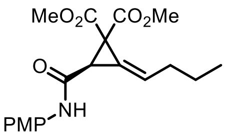

(Chiralpak AD-H, hexane/isopropanol = 90/10, 1 ml/min)

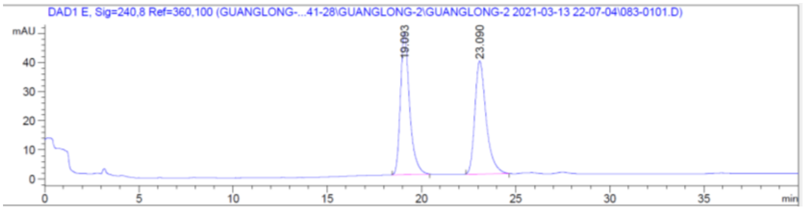

Signal 5: DAD1 E, Sig=240,8 Ref=360,100

Peak #	RetTime [min]	Type	Width [min]	Area [mAU*s]	Height [mAU]	Area %
1	19.093	BB	0.5124	1659.32935	48.83737	50.6695
2	23.090	BB	0.6305	1615.48108	38.74138	49.3305
Totals :				3274.81042	87.57875	

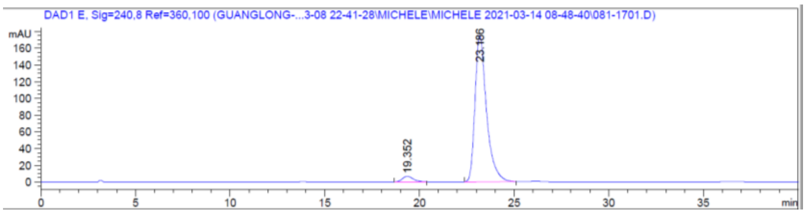

Signal 5: DAD1 E, Sig=240,8 Ref=360,100

Peak #	RetTime [min]	Type	Width [min]	Area [mAU*s]	Height [mAU]	Area %
1	19.352	BB	0.6037	260.51288	6.61185	3.3948
2	23.186	BB	0.6371	7413.30176	175.41408	96.6052
Totals :				7673.81464	182.02592	

dimethyl (R,Z)-2-butylidene-3-((4-hydroxyphenyl)carbamoyl)cyclopropane-1,1-dicarboxylate (29c)

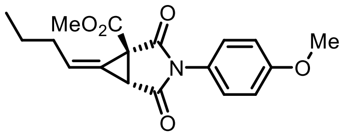

(Chiralpak AS-H, hexane/isopropanol = 90/10 to 70/30, 1 ml/min)

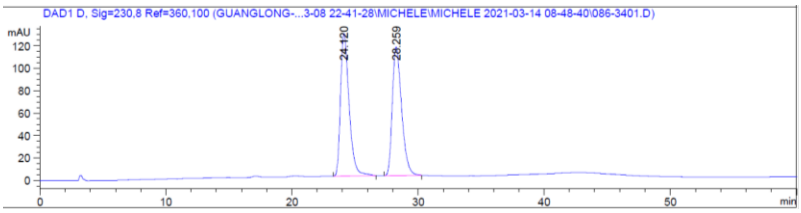

Signal 4: DAD1 D, Sig=230,8 Ref=360,100

Peak #	RetTime [min]	Type	Width [min]	Area [mAU*s]	Height [mAU]	Area %
1	24.120	BB	0.7247	6021.92334	127.11844	50.6063
2	28.259	BB	0.7888	5877.63135	114.52177	49.3937
Totals :				1.18996e4	241.64021	

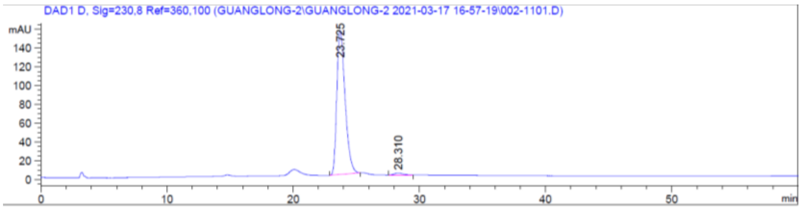

Signal 4: DAD1 D, Sig=230,8 Ref=360,100

Peak #	RetTime [min]	Type	Width [min]	Area [mAU*s]	Height [mAU]	Area %
1	23.725	BB	0.6981	6954.54492	153.05290	98.5299
2	28.310	BB	0.6539	103.76665	2.17788	1.4701
Totals :				7058.31158	155.23079	

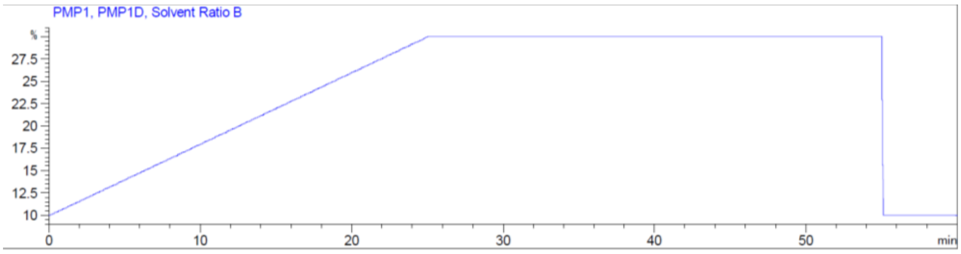

dimethyl (R,E)-2-butylidene-3-(diphenylphosphoryl)cyclopropane-1,1-dicarboxylate (30b)

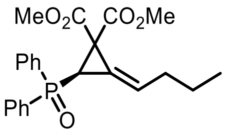

(Chiralpak AS-H, hexane/isopropanol = 80/20, 1 ml/min)

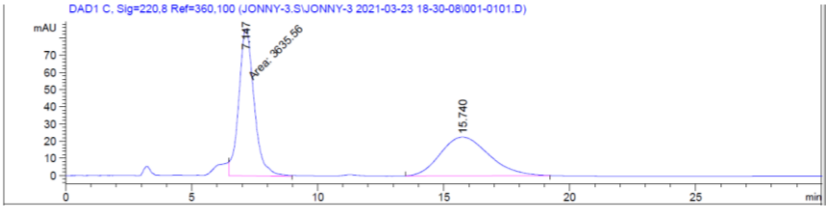

Signal 3: DAD1 C, Sig=220,8 Ref=360,100

Peak #	RetTime [min]	Type	Width [min]	Area [mAU*s]	Height [mAU]	Area %
1	7.147	MM	0.7076	3635.55615	85.63195	54.4020
2	15.740	BB	1.8518	3047.20483	22.61794	45.5980
Totals :				6682.76099	108.24989	

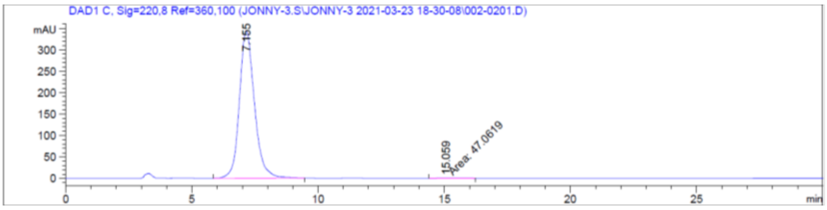

Signal 3: DAD1 C, Sig=220,8 Ref=360,100

Peak #	RetTime [min]	Type	Width [min]	Area [mAU*s]	Height [mAU]	Area %
1	7.155	BB	0.6315	1.42551e4	343.99744	99.6709
2	15.059	MM	0.9394	47.06189	8.34926e-1	0.3291
Totals :				1.43022e4	344.83236	

dimethyl (S,Z)-2-butylidene-3-(3-phenylpropanoyl)cyclopropane-1,1-dicarboxylate (4b)

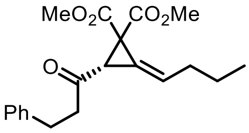

(Chiralpak AS-H, hexane/isopropanol = 98/2, 1 ml/min)

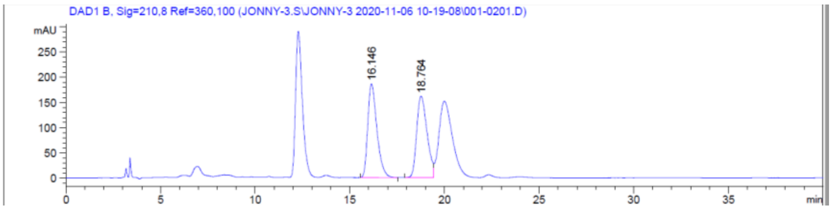

Signal 2: DAD1 B, Sig=210,8 Ref=360,100

Peak #	RetTime [min]	Type	Width [min]	Area [mAU*s]	Height [mAU]	Area %
1	16.146	BB	0.5130	6270.05615	186.13019	50.3046
2	18.764	BV	0.5902	6194.12305	161.95111	49.6954
Totals :				1.24642e4	348.08130	

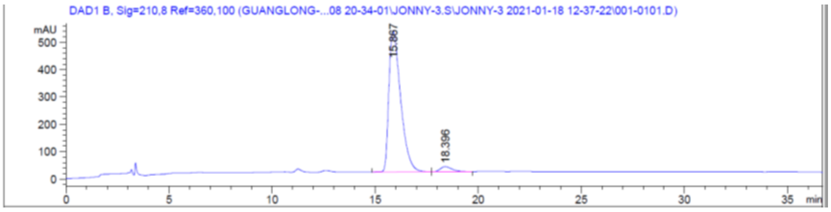

Signal 2: DAD1 B, Sig=210,8 Ref=360,100

Peak #	RetTime [min]	Type	Width [min]	Area [mAU*s]	Height [mAU]	Area %
1	15.867	BV	0.6101	2.04929e4	517.42603	96.5249
2	18.396	VB	0.5947	737.77753	18.76310	3.4751
Totals :				2.12307e4	536.18913	

Crystal sample (92% ee)

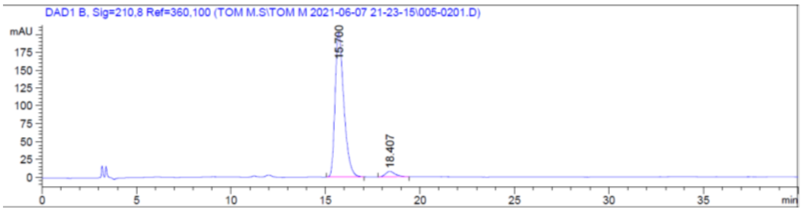

Signal 2: DAD1 B, Sig=210,8 Ref=360,100

Peak #	RetTime [min]	Type	Width [min]	Area [mAU*s]	Height [mAU]	Area %
1	15.700	BB	0.4867	6499.23877	202.35170	95.9629
2	18.407	BB	0.5326	273.42148	7.58487	4.0371
Totals :				6772.66025	209.93657	

dimethyl (S)-2-cyclopentylidene-3-(3-phenylpropanoyl)cyclopropane-1,1-dicarboxylate (31b)

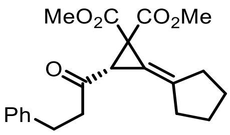

(Chiralpak AD-H, hexane/isopropanol = 98/2, 1 ml/min)

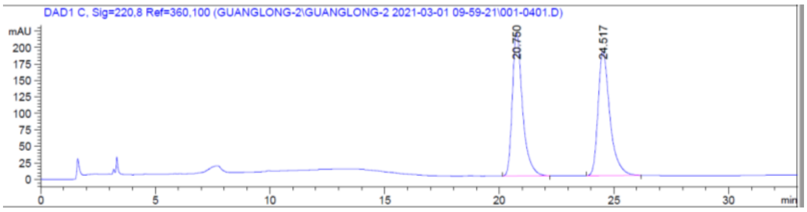

Signal 3: DAD1 C, Sig=220,8 Ref=360,100

Peak #	RetTime [min]	Type	Width [min]	Area [mAU*s]	Height [mAU]	Area %
1	20.750	BB	0.4649	6692.24756	217.52118	49.8419
2	24.517	BB	0.5390	6734.69580	188.41028	50.1581
Totals :				1.34269e4	405.93146	

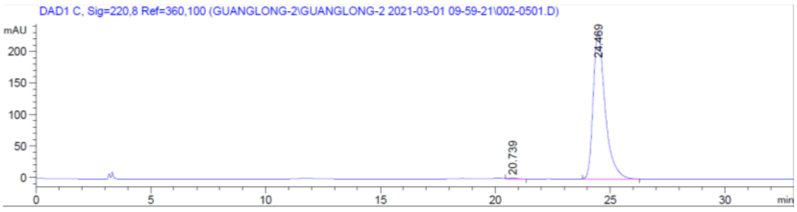

Signal 3: DAD1 C, Sig=220,8 Ref=360,100

Peak #	RetTime [min]	Type	Width [min]	Area [mAU*s]	Height [mAU]	Area %
1	20.739	VB	0.4183	44.56236	1.58361	0.5239
2	24.469	BB	0.5452	8461.42676	234.35800	99.4761
Totals :				8505.98912	235.94162	

dimethyl (S,Z)-2-butylidene-3-(4-methoxybenzoyl)cyclopropane-1,1-dicarboxylate (32b)

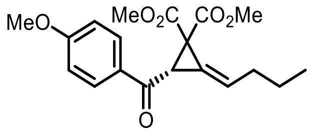

(Chiralpak AD-H, hexane/isopropanol = 95/5, 1 ml/min)

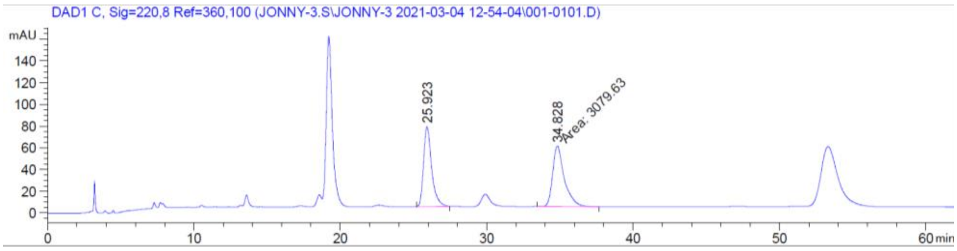

Signal 3: DAD1 C, Sig=220,8 Ref=360,100

Peak #	RetTime [min]	Type	Width [min]	Area [mAU*s]	Height [mAU]	Area %
1	25.923	BB	0.5797	2823.47705	73.57510	47.8303
2	34.828	MM	0.9231	3079.63135	55.60331	52.1697

Totals : 5903.10840 129.17841

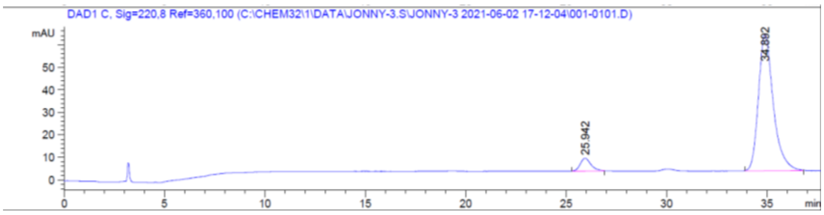

Signal 3: DAD1 C, Sig=220,8 Ref=360,100

Peak #	RetTime [min]	Type	Width [min]	Area [mAU*s]	Height [mAU]	Area %
1	25.942	BB	0.5432	212.59039	5.69861	6.3938
2	34.892	BB	0.7795	3112.37817	60.57398	93.6062

Totals : 3324.96857 66.27259

benzyl (S,Z)-3-(((2-methoxyethoxy)methoxy)methylene)-2,2-dimethylcyclopropane-1-carboxylate (33b)

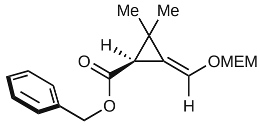

(Chiralpak AD-H, hexane/isopropanol = 98/2, 1 ml/min)

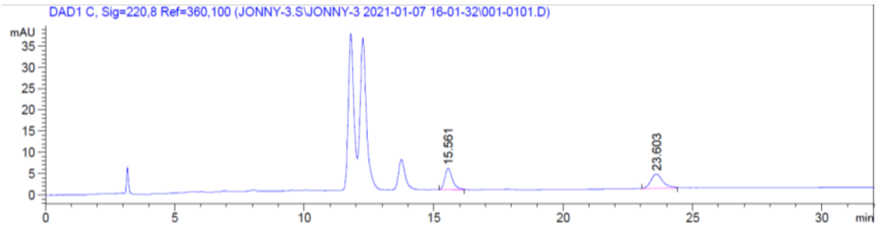

Signal 3: DAD1 C, Sig=220,8 Ref=360,100

Peak #	RetTime [min]	Type	Width [min]	Area [mAU*s]	Height [mAU]	Area %
1	15.561	BB	0.3142	105.09305	5.06254	49.3450
2	23.603	BB	0.4812	107.88289	3.37239	50.6550
Totals :				212.97594	8.43492	

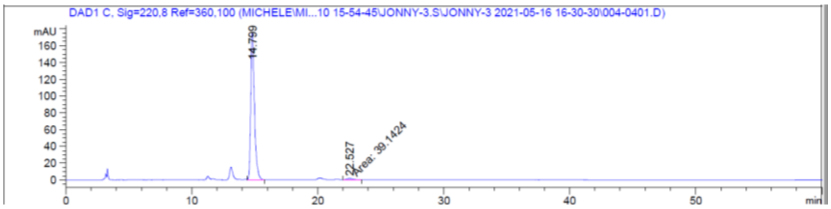

Signal 3: DAD1 C, Sig=220,8 Ref=360,100

Peak #	RetTime [min]	Type	Width [min]	Area [mAU*s]	Height [mAU]	Area %
1	14.799	VB	0.3071	3560.86963	175.17908	98.9127
2	22.527	MM	0.5211	39.14240	1.25186	1.0873
Totals :				3600.01203	176.43094	

3-phenoxybenzyl (S,Z)-3-(((2-methoxyethoxy)methoxy)methylene)-2,2-dimethylcyclopropane-1-carboxylate (34b)

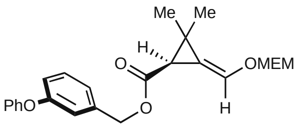

(Chiralpak AS-H, hexane/isopropanol = 95/5, 1 ml/min)

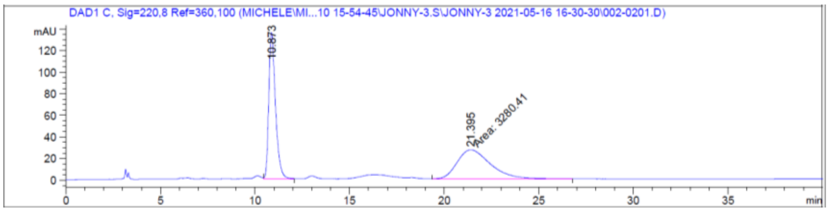

Signal 3: DAD1 C, Sig=220,8 Ref=360,100

Peak #	RetTime [min]	Type	Width [min]	Area [mAU*s]	Height [mAU]	Area %
1	10.873	VB	0.3720	3348.31665	135.62076	50.5122
2	21.395	MM	2.0360	3280.41309	26.85279	49.4878
Totals :				6628.72974	162.47355	

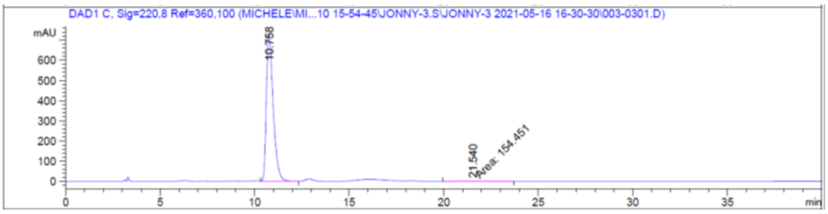

Signal 3: DAD1 C, Sig=220,8 Ref=360,100

Peak #	RetTime [min]	Type	Width [min]	Area [mAU*s]	Height [mAU]	Area %
1	10.758	VV	0.3863	1.84321e4	730.71564	99.1690
2	21.540	MM	1.9651	154.45111	1.30994	0.8310
Totals :				1.85865e4	732.02558	

(1R, 3R)-cis-Permethrin (2)

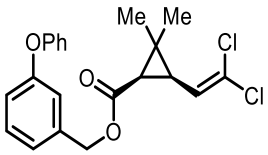

(Chiralpak IB, hexane/isopropanol = 99/1, 1 ml/min)

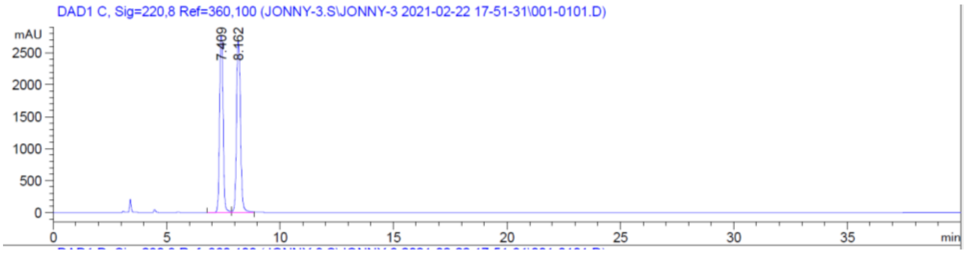

Signal 3: DAD1 C, Sig=220,8 Ref=360,100

Peak #	RetTime [min]	Type	Width [min]	Area [mAU*s]	Height [mAU]	Area %
1	7.409	VV	0.1761	3.03957e4	2769.14575	47.9883
2	8.162	VV	0.1928	3.29442e4	2690.62305	52.0117

Totals : 6.33399e4 5459.76880

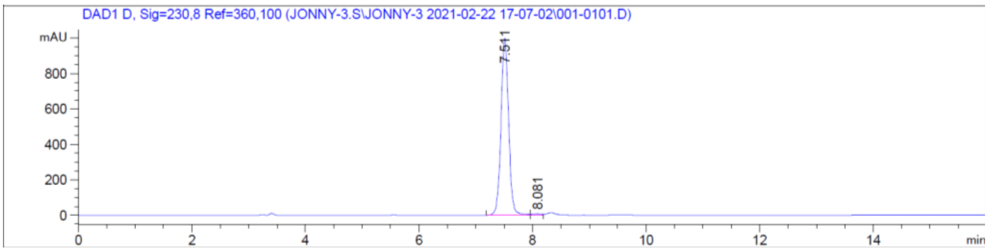

Signal 3: DAD1 C, Sig=220,8 Ref=360,100

Peak #	RetTime [min]	Type	Width [min]	Area [mAU*s]	Height [mAU]	Area %
1	7.511	BV	0.1455	1.39525e4	1486.63171	99.0789
2	8.081	VV	0.1629	129.71309	11.56458	0.9211

Totals : 1.40822e4 1498.19630

2,3,5,6-tetrafluoro-4-methylbenzyl (1R,3S)-3-formyl-2,2-dimethylcyclopropane-1-carboxylate (35b)

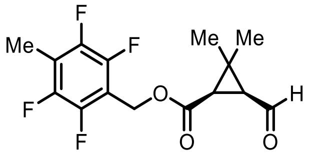

(Chiralpak AS-H, hexane/isopropanol = 98/2, 1 ml/min)

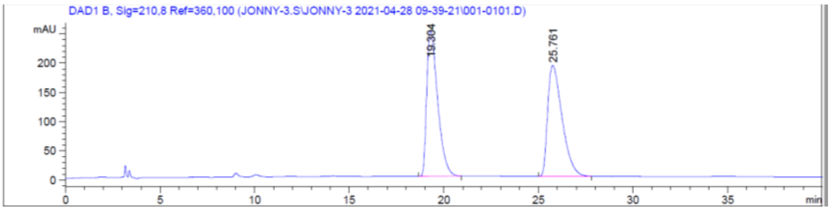

Signal 2: DAD1 B, Sig=210,8 Ref=360,100

Peak #	RetTime [min]	Type	Width [min]	Area [mAU*s]	Height [mAU]	Area %
1	19.304	BB	0.6200	9988.81738	248.98643	49.8254
2	25.761	BB	0.8226	1.00588e4	189.72003	50.1746

Totals : 2.00476e4 438.70647

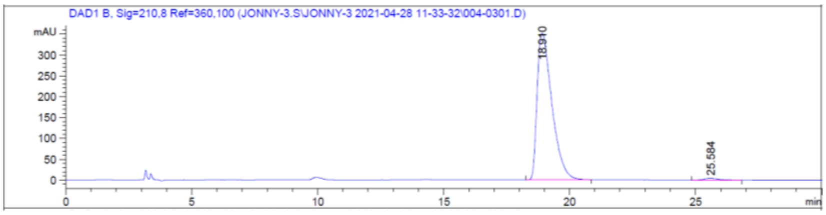

Signal 2: DAD1 B, Sig=210,8 Ref=360,100

Peak #	RetTime [min]	Type	Width [min]	Area [mAU*s]	Height [mAU]	Area %
1	18.910	BB	0.6245	1.43334e4	350.91733	98.8262
2	25.584	BB	0.5718	170.23697	3.97371	1.1738

Totals : 1.45036e4 354.89104

2,3,5,6-tetrafluoro-4-(methoxymethyl)benzyl (1R,3S)-3-formyl-2,2-dimethylcyclopropane-1-carboxylate (36b)

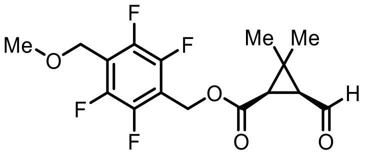

(Chiralpak AS-H, hexane/isopropanol = 97/3, 1 ml/min)

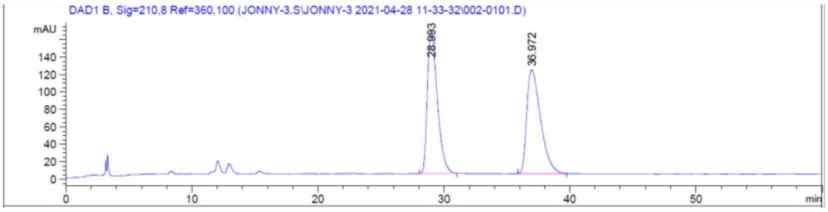

Signal 2: DAD1 B, Sig=210,8 Ref=360,100

Peak #	RetTime [min]	Type	Width [min]	Area [mAU*s]	Height [mAU]	Area %
1	28.993	BB	0.8293	9043.24902	164.50764	49.8536
2	36.972	BB	1.1619	9096.34961	119.25816	50.1464
Totals :				1.81396e4	283.76580	

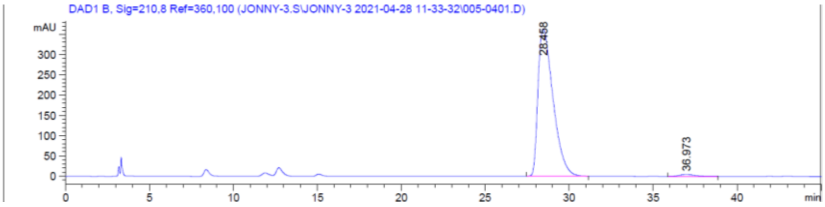

Signal 2: DAD1 B, Sig=210,8 Ref=360,100

Peak #	RetTime [min]	Type	Width [min]	Area [mAU*s]	Height [mAU]	Area %
1	28.458	BB	0.9667	2.26493e4	363.63037	98.5941
2	36.973	BB	0.8939	322.96301	4.62965	1.4059
Totals :				2.29722e4	368.26002	

(2-methyl-[1,1'-biphenyl]-3-yl)methyl (1R,3S)-3-formyl-2,2-dimethylcyclopropane-1-carboxylate (37b)

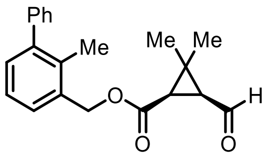

(Chiralpak AS-H, hexane/isopropanol = 95/5, 1 ml/min)

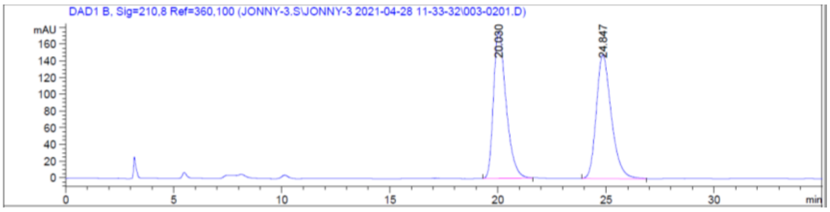

Signal 2: DAD1 B, Sig=210,8 Ref=360,100

Peak #	RetTime [min]	Type	Width [min]	Area [mAU*s]	Height [mAU]	Area %
1	20.030	BB	0.6167	7132.07666	176.03253	49.9492
2	24.847	BB	0.7397	7146.58740	147.91612	50.0508

Totals : 1.42787e4 323.94865

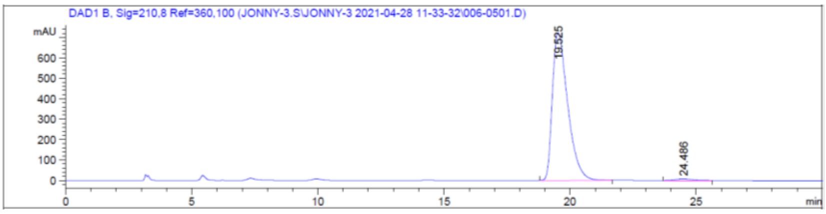

Signal 2: DAD1 B, Sig=210,8 Ref=360,100

Peak #	RetTime [min]	Type	Width [min]	Area [mAU*s]	Height [mAU]	Area %
1	19.525	BB	0.6495	3.05066e4	721.19086	98.8167
2	24.486	BB	0.6578	365.30002	8.04487	1.1833

Totals : 3.08719e4 729.23572

benzyl (S,Z)-2-propylidenespiro[2.5]octane-1-carboxylate (38b)

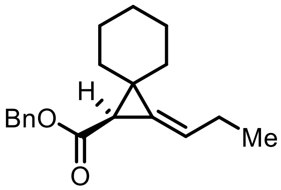

(SFC Chiralcel IG, 1500 psi, from 1% to 30% IPA in 5 min, 1.5 ml/min)

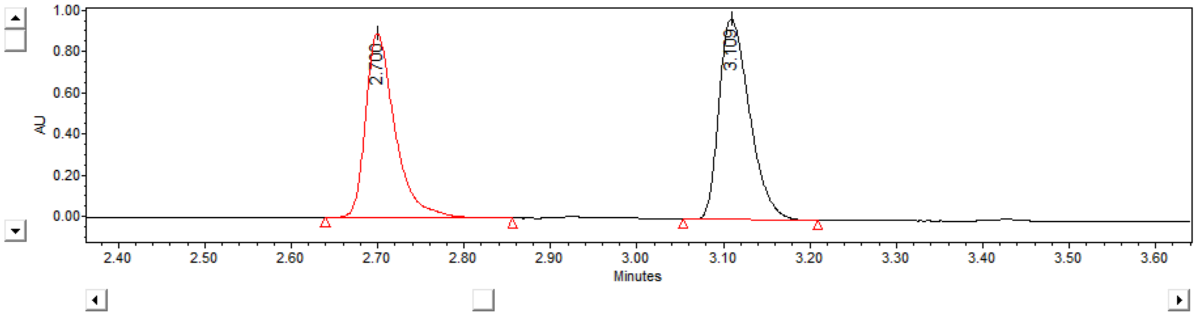

	Name	Retention Time (min)	Purity1 Angle	Purity1 Threshold	PDA/FLR Match1 Spect. Name	PDA/FLR Match1 Angle	PDA/FLR Match1 Threshold	PDA/FLR Match1 Lib. Name	Area (µV*sec)	% Area	Height (µV)	Int Type	Amount	Units	Peak Typ
1		2.700							2111719	46.94	893305	bb			Unknown
2		3.109							2386859	53.06	970908	bb			Unknown

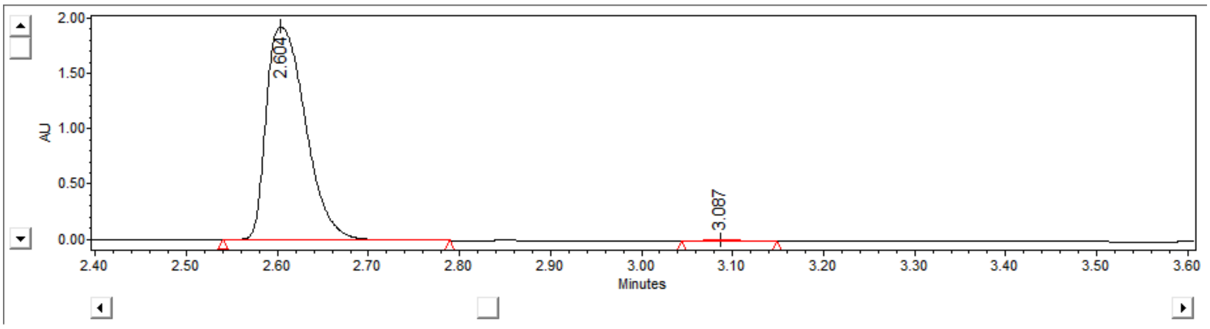

	Name	Retention Time (min)	Purity1 Angle	Purity1 Threshold	PDA/FLR Match1 Spect. Name	PDA/FLR Match1 Angle	PDA/FLR Match1 Threshold	PDA/FLR Match1 Lib. Name	Area (µV*sec)	% Area	Height (µV)	Int Type	Amount	Units	Peak Ty
1		2.604							5738078	99.49	1921337	bb			Unknowr
2		3.087							29201	0.51	13545	bb			Unknowr

benzyl (S,Z)-3-((allyloxy)methylene)-2,2-dimethylcyclopropane-1-carboxylate (39b)

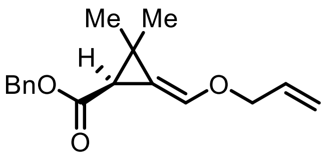

(chiralpak AD, hexane/isopropanol = 95/5, 1 ml/min)

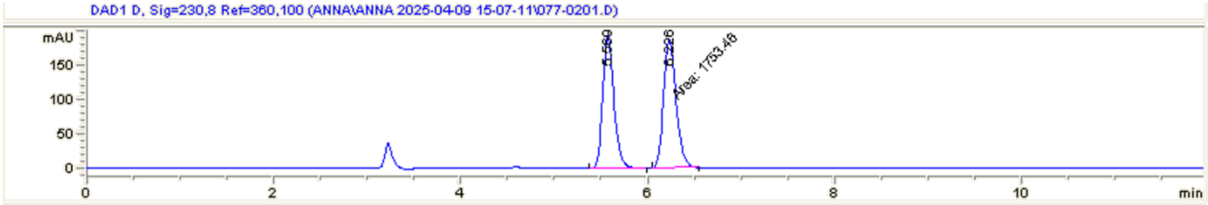

#	Time	Area	Height	Width	Area%	Symmetry
1	5.569	1620.8	194.4	0.1273	48.035	0.763
2	6.226	1753.5	189	0.1546	51.965	0.797

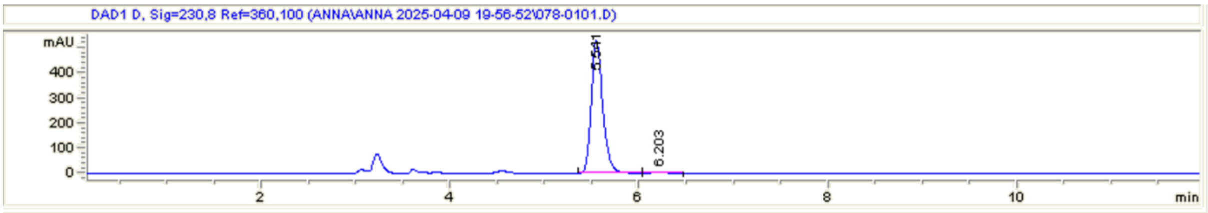

#	Time	Area	Height	Width	Area%	Symmetry
1	5.541	4530.1	532.2	0.1293	98.839	0.735
2	6.203	53.2	4.8	0.1654	1.161	0.805

(S,Z)-(3-((allyloxy)methylene)-2,2-dimethylcyclopropyl)methanol (40)

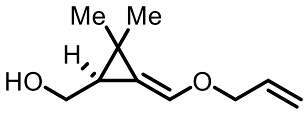

(chiralpak AD-H, hexane/isopropanol = 99/1, 1 ml/min)

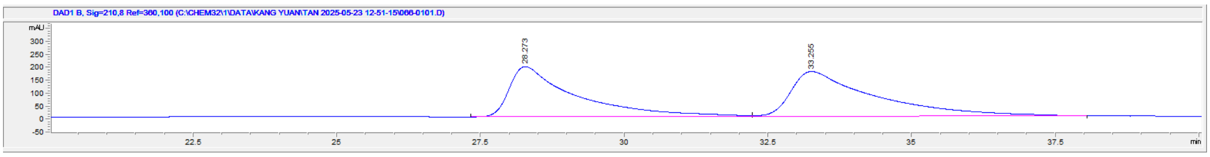

#	Time	Area	Height	Width	Area%	Symmetry
1	28.273	15557.6	193.6	1.0954	48.937	0.287
2	33.255	16233.8	170.2	1.5901	51.063	0.331

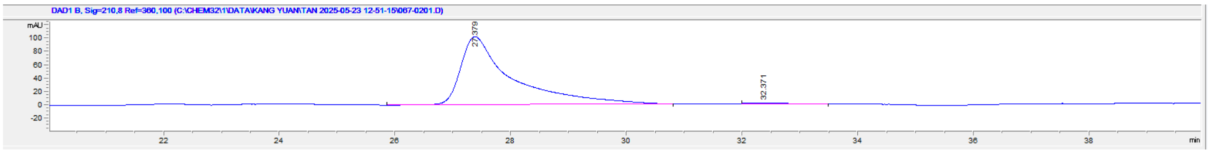

#	Time	Area	Height	Width	Area%	Symmetry
1	27.379	6467.7	102.6	0.8658	98.298	0.341
2	32.371	112	2.1	0.6255	1.702	0.56

benzyl (3R,4S)-5,5-dimethyl-1-oxaspiro[2.2]pentane-4-carboxylate (41)

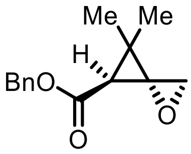

(chiralpak IA, hexane/isopropanol = 98/2, 1 ml/min)

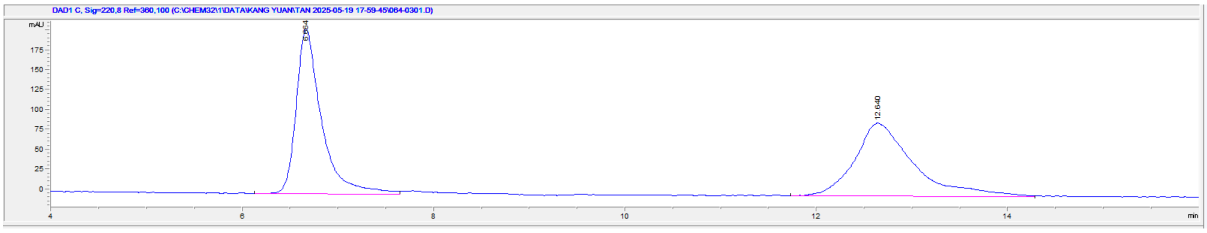

#	Time	Area	Height	Width	Area%	Symmetry
1	6.664	3875.2	207.6	0.2697	49.242	0.58
2	12.64	3994.5	92.3	0.5787	50.758	0.625

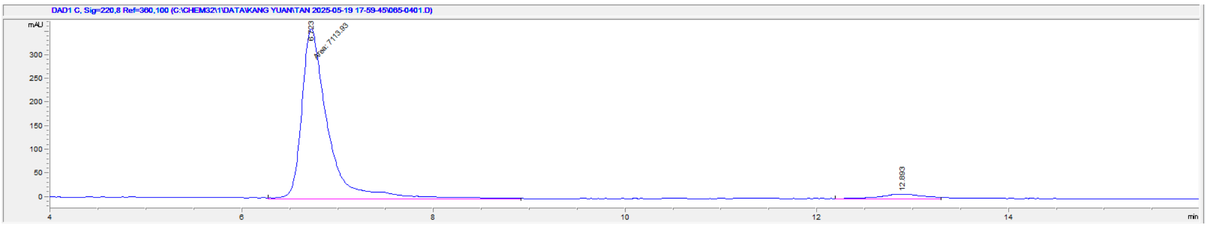

#	Time	Area	Height	Width	Area%	Symmetry
1	6.723	7113.9	361	0.3284	95.043	0.536
2	12.893	371	10.6	0.4193	4.957	1.147

11. Computational Studies

11.1 Computational Methods

All calculations reported in this study were performed using the Amsterdam Density Functional (ADF) software.[23-25] Equilibrium structures and transition structure geometries were optimized using the BLYP functional[26-29] and the DZP basis set.[30] Solvent effects of Et_2O were accounted for using the conducter-like screen model (COSMO) of solvation.[31-34] Dispersion interactions were included using Grimme's DFT-D3 correction with Becke-Johnson damping.[35,36] The zeroth-order regular approximation (ZORA) was used to account for scalar relativistic effects.[37,38] This level is referred to as COSMO(Et_2O)-ZORA-BLYP-D3(BJ)/DZP. All stationary points have been verified, through vibrational analysis, to be minima (zero imaginary frequencies) or transition structures (one imaginary frequency). The character of the normal mode associated with the imaginary frequency has been analysed to ensure it resembles the reaction under consideration. Optimized structures were illustrated using CYLview20.[39] Potential energies were refined by means of single point calculations using the M06-2X functional[36] and the TZ2P basis set.[30] This level is denoted COSMO(Et_2O)-ZORA-M06-2X/TZ2P//COSMO(Et_2O)-ZORA-BLYP-D3(BJ)/DZP.

The intramolecular interaction energies in the transition states were computed by using the energy decomposition analysis (EDA) at ZORA-M06-2X/TZ2P.[37-39] For this analysis, the transition states were fragmented such that the interaction energy between just the interacting fragments could be quantified. Disconnections were made by capping with a hydrogen atom followed by an optimization of the X–H bond length at COSMO(Et_2O)-ZORA-BLYP-D3(BJ)/TZ2P.

11.2 Computational Results

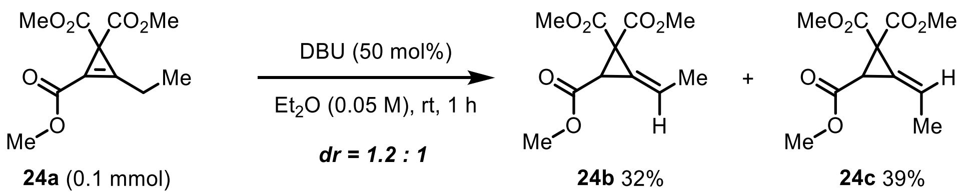

Scheme S4: Experimental observation for the racemic deconjugation of ester **24a** with DBU.

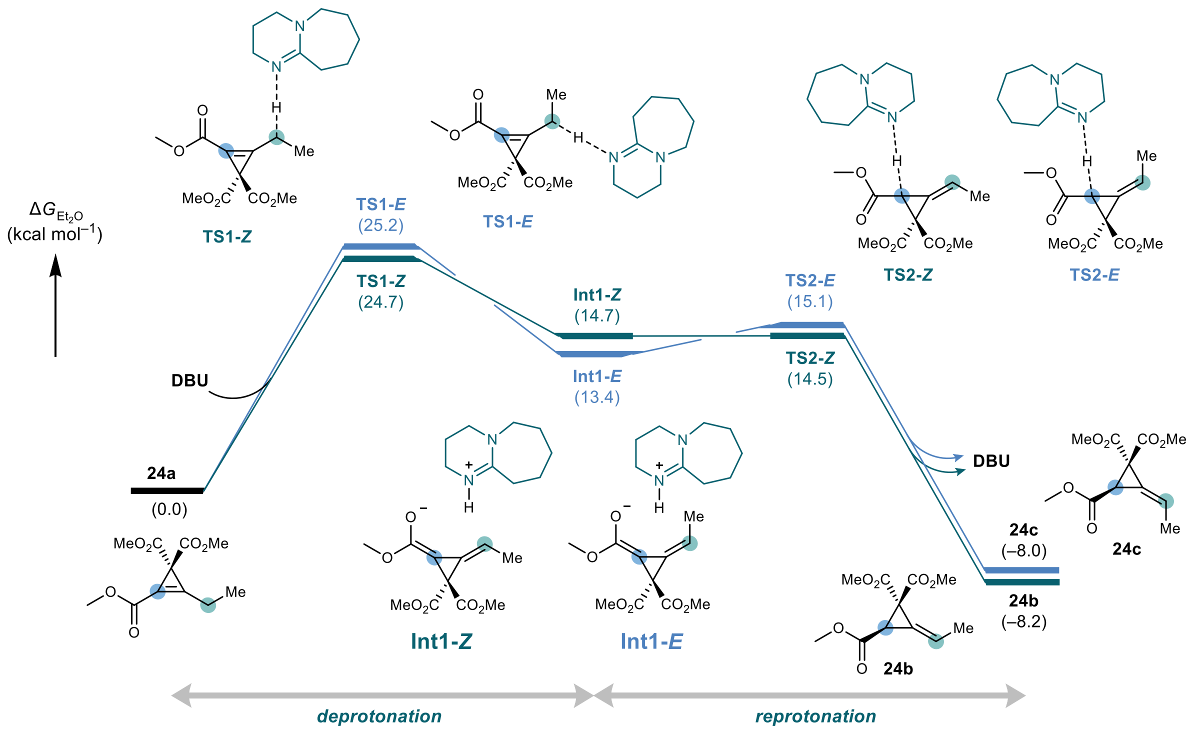

Scheme S5: Computed potential energy surfaces (ΔG [kcal mol^{-1}]) for the DBU-catalysed 1,3-prototropic shift computed at COSMO(Et_2O)-ZORA-M06-2X/TZ2P//COSMO(Et_2O)-ZORA-BLYP-D3(BJ)/DZP.

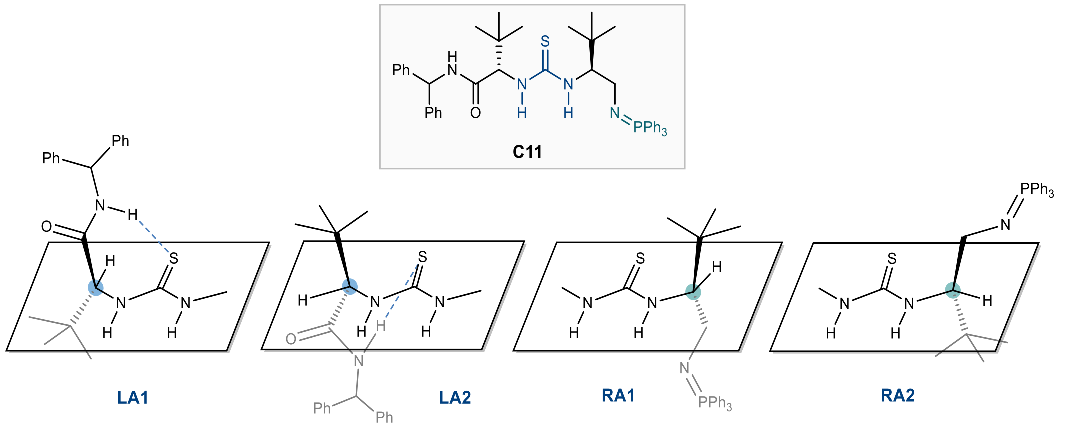

Figure S7: Catalyst conformations of the *left arm* and *right arm* of **C11**.

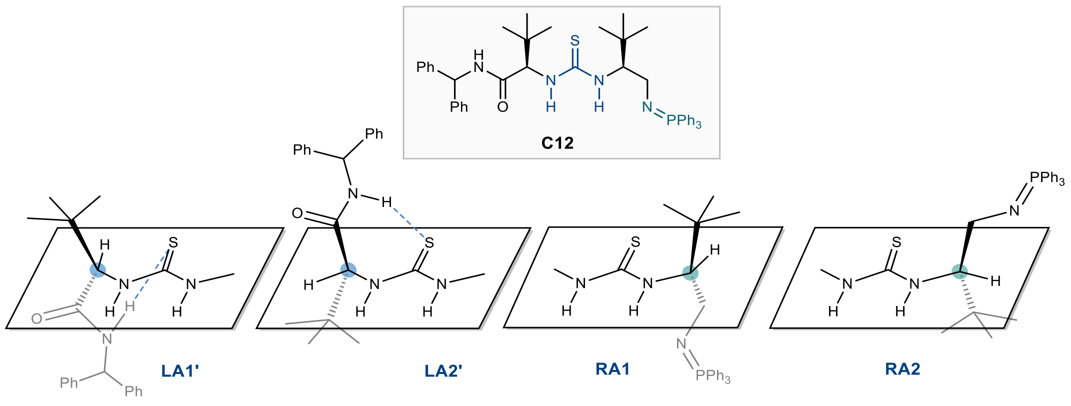

Figure S8: Catalyst conformations of the *left arm* and *right arm* of **C12**.

Since the BIMP catalyst has a rich conformational space, the potential conformations on both side arms of the molecule were considered. The conformational spaces of both the "*left arm – LA*" (side containing the amide moiety with the *tert*-butyl group) and "*right arm – RA*" (side with the iminophosphorane moiety with the *tert*-butyl group) of the BIMP catalyst were explored. Regarding the *left arm*, two conformations are possible which form a hydrogen bonding between the amide and the thiourea. On the other hand, the *right arm* of the catalyst can have the iminophosphorane located either on the bottom or top side of the thiourea plane. Various conformations of the catalysts are likely accessible, therefore every possible conformation of the BIMP catalyst were calculated for all key transition states.

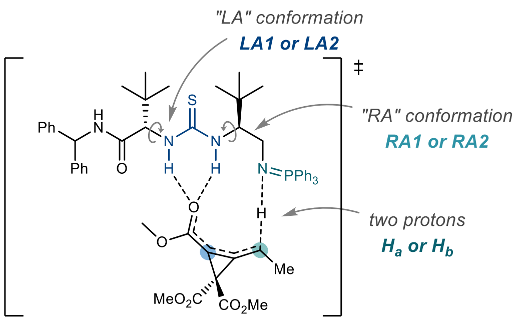

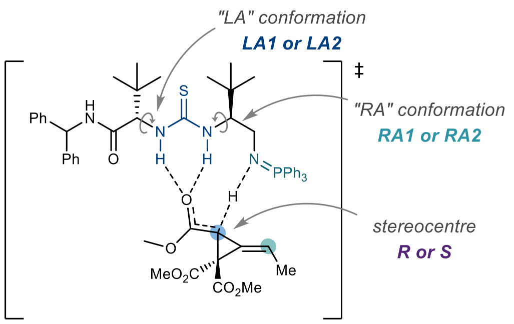

TSs for deprotonation	$\Delta G^{\ddagger}$	$\Delta E^{\ddagger}$
**TS1–depro–LA1–RA1–H_a**	**21.7**	**2.7**
**TS1–depro–LA1–RA2–H_a**	22.1	4.8
**TS1–depro–LA2–RA1–H_a**	36.2	17.9
**TS1–depro–LA2–RA2–H_a**	33.1	13.0
**TS1–depro–LA1–RA1–H_b**	22.5	4.1
**TS1–depro–LA1–RA2–H_b**	26.6	7.0
**TS1–depro–LA2–RA1–H_b**	*n.d.*	*n.d.*
**TS1–depro–LA2–RA2–H_b**	35.5	15.1

TSs for reprotonation	$\Delta G^{\ddagger}$	$\Delta E^{\ddagger}$
**TS2–repro–LA1–RA1–*R***	**10.9**	**–8.7**
**TS2–repro–LA1–RA2–*R***	26.5	7.9
**TS2–repro–LA2–RA1–*R***	13.7	–4.8
**TS2–repro–LA2–RA2–*R***	23.9	2.4
**TS2–repro–LA1–RA1–*S***	**15.1**	**–5.1**
**TS2–repro–LA1–RA2–*S***	20.2	5.0
**TS2–repro–LA2–RA1–*S***	20.1	0.8
**TS2–repro–LA2–RA2–*S***	28.2	7.6

Figure S9: Relative stability of the deprotonation and protonation transition structures of cyclopropene ester **24a** with **C11** computed at COSMO(Et_2O)-ZORA-M06-2X/TZ2P//COSMO(Et_2O)-ZORA-BLYP-D3(BJ)/DZP. Energies (kcal mol^{-1}) are provided in the insert.

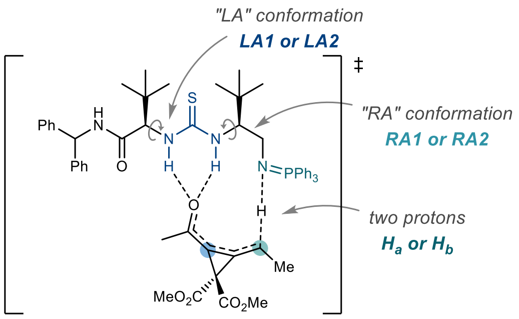

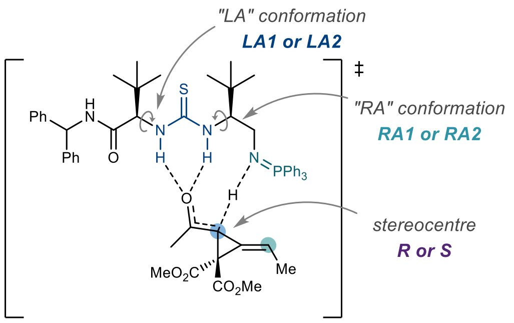

TSs for deprotonation	$\Delta G^{\ddagger}$	$\Delta E^{\ddagger}$
**TS3–depro–LA1'–RA1–H_a**	**20.0**	**4.4**
**TS3–depro–LA1'–RA2–H_a**	33.1	15.4
**TS3–depro–LA2–RA1–H_a**	28.0	12.6
**TS3–depro–LA2–RA2–H_a**	*n.d.*	*n.d.*
**TS3–depro–LA1'–RA1–H_b**	23.5	9.1
**TS3–depro–LA1'–RA2–H_b**	*n.d.*	*n.d.*
**TS3–depro–LA2–RA1–H_b**	38.3	20.7
**TS3–depro–LA2–RA2–H_b**	33.5	18.0

TSs for reprotonation	$\Delta G^{\ddagger}$	$\Delta E^{\ddagger}$
**TS4–repro–LA1'–RA1–*R***	*n.d.*	*n.d.*
**TS4–repro–LA1'–RA2–*R***	31.4	14.4
**TS4–repro–LA2–RA1–*R***	**25.3**	**9.0**
**TS4–repro–LA2–RA2–*R***	46.4	23.8
**TS4–repro–LA1'–RA1–*S***	29.8	11.2
**TS4–repro–LA1'–RA2–*S***	28.2	11.0
**TS4–repro–LA2–RA1–*S***	**23.7**	**7.0**
**TS4–repro–LA2–RA2–*S***	29.7	13.4

Figure S10: Relative stability of the deprotonation and protonation transition structures of cyclopropene ester **24a** with **C12** computed at COSMO(Et_2O)-ZORA-M06-2X/TZ2P//COSMO(Et_2O)-ZORA-BLYP-D3(BJ)/DZP. Energies (kcal mol^{-1}) are provided in the insert.

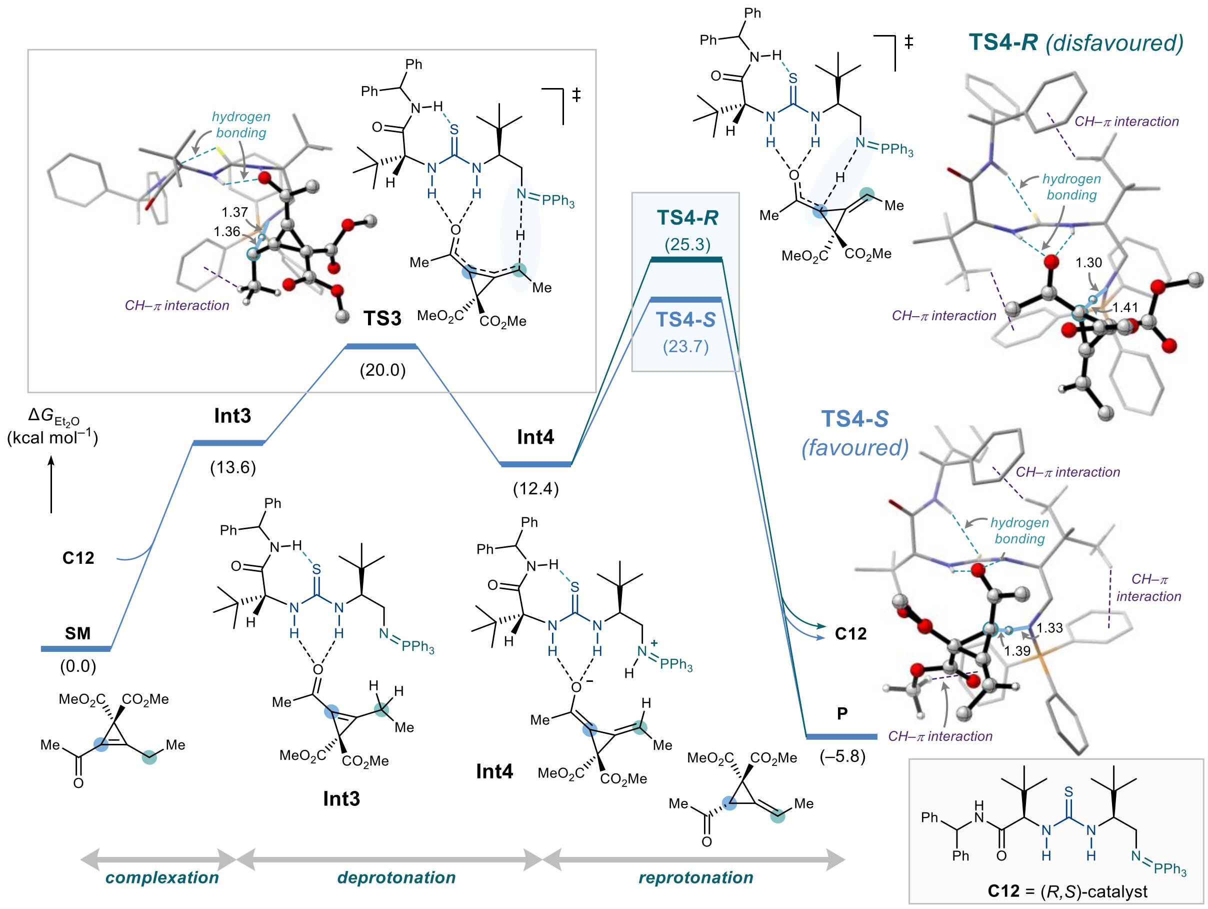

Scheme S6: Computed potential energy surfaces (Δ*G* [kcal mol^{-1}]) for the BIMP-catalysed 1,3-prototropic shift of ketone computed at COSMO(Et2O)-ZORA-M06-2X/TZ2P//COSMO(Et2O)-ZORA-BLYP-D3(BJ)/DZP.

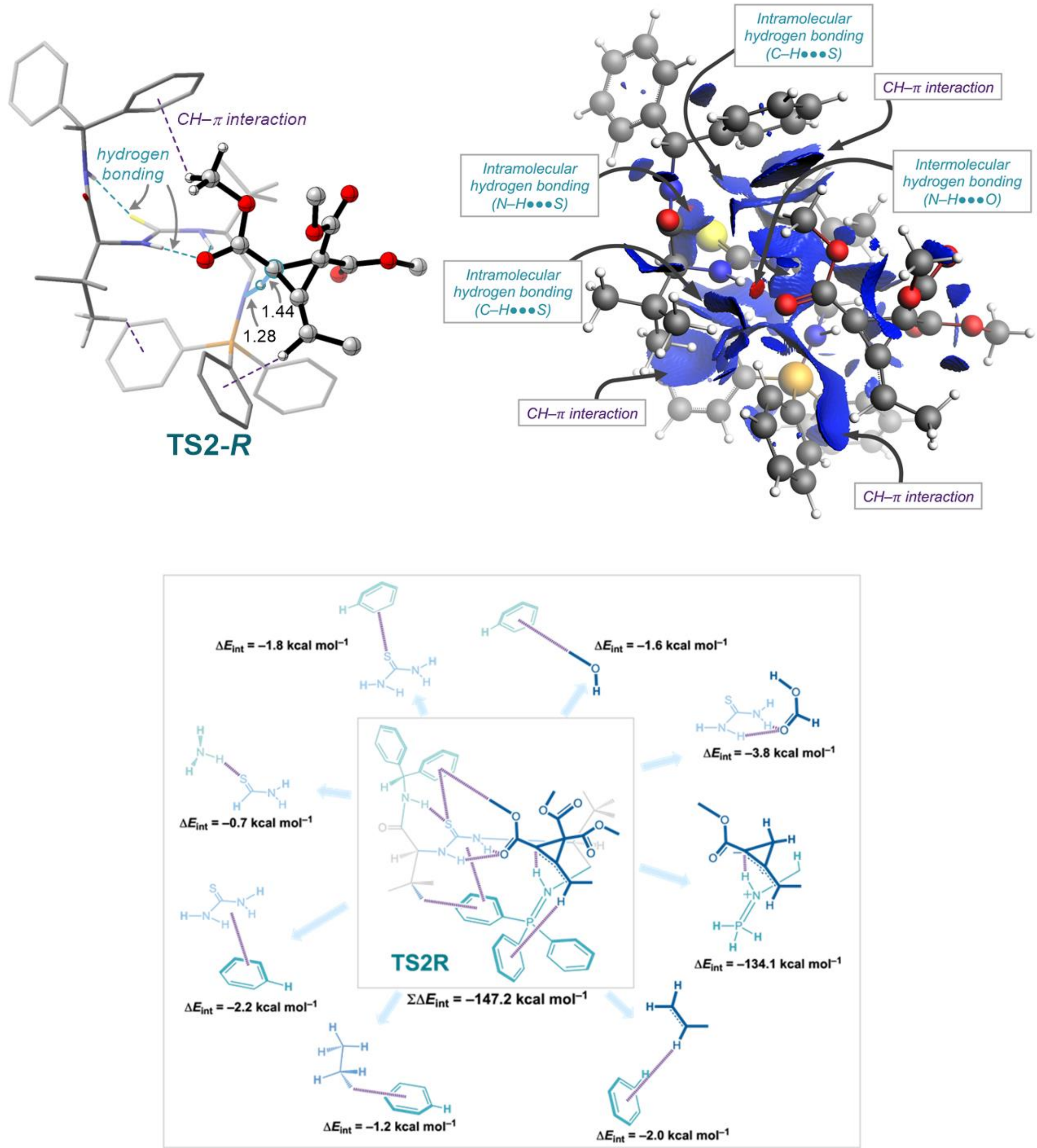

Figure S11: Transition state structure of **TS2-*R*** and the ADFview plot of non-covalent interaction (NCI) regions materialized by reduced density gradient isosurfaces (all default settings were used) colored according to the sign of the signed density *λ2ρ* (red and blue colors are associated with negatively and positively signed terms). Interaction energies in **TS2-*R*** are shown below with truncated fragments using the energy decomposition analysis (EDA). Disconnections were made by capping with a hydrogen atom with an optimized X–H bond length. Computed at ZORA-M06-2X/TZ2P//COSMO(Et_2O)-ZORA-BLYP-D3(BJ)/DZP.

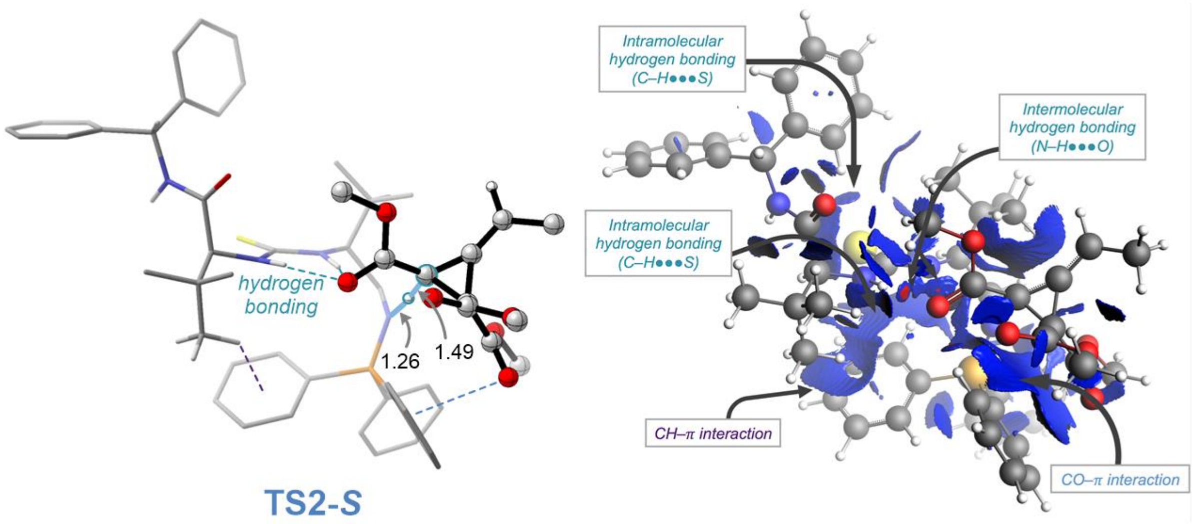

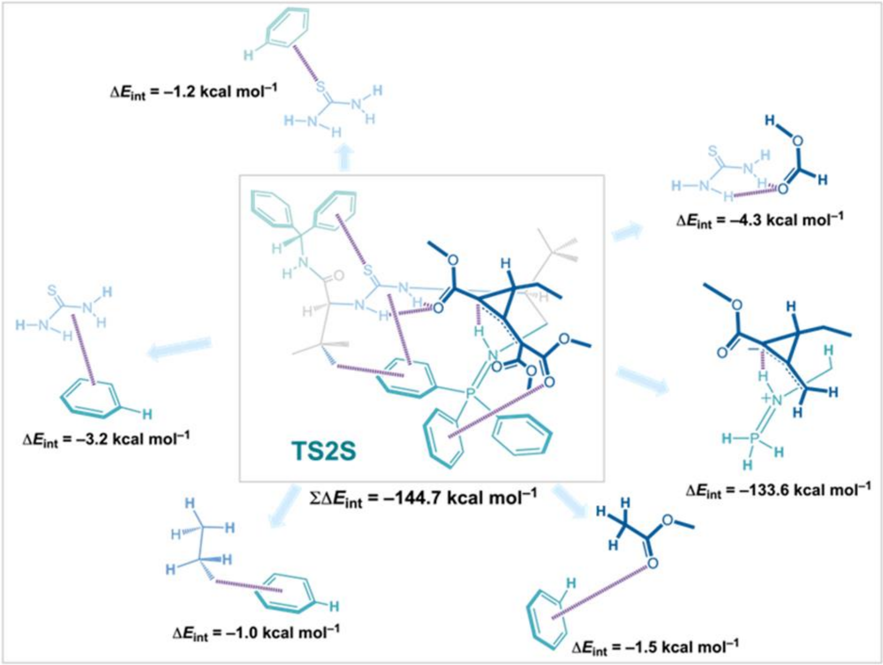

Figure S12: Transition state structure of **TS2-*S*** and the ADFview plot of non-covalent interaction (NCI) regions materialized by reduced density gradient isosurfaces (all default settings were used) colored according to the sign of the signed density *λ2ρ* (red and blue colors are associated with negatively and positively signed terms). Interaction energies in **TS2-*S*** are shown below with truncated fragments using the energy decomposition analysis (EDA). Disconnections were made by capping with a hydrogen atom with an optimized X–H bond length. Computed at ZORA-M06-2X/TZ2P//COSMO(Et_2O)-ZORA-BLYP-D3(BJ)/DZP.

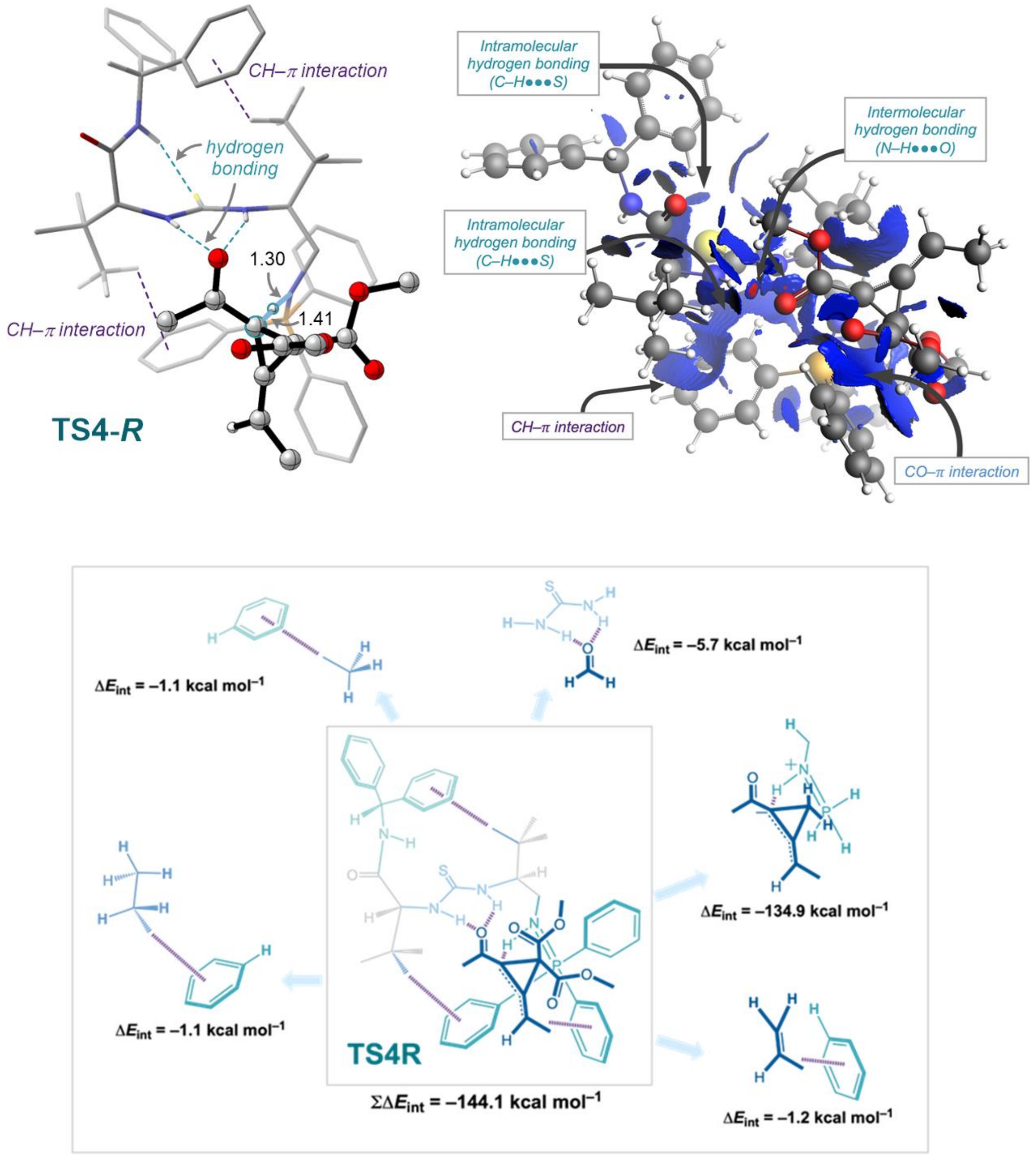

Figure S13: Transition state structure of **TS4-*R*** and the ADFview plot of non-covalent interaction (NCI) regions materialized by reduced density gradient isosurfaces (all default settings were used) colored according to the sign of the signed density $\lambda 2\rho$ (red and blue colors are associated with negatively and positively signed terms). Interaction energies in **TS4-*R*** are shown below with truncated fragments using the energy decomposition analysis (EDA). Disconnections were made by capping with a hydrogen atom with an optimized X–H bond length. Computed at ZORA-M06-2X/TZ2P//COSMO(Et_2O)-ZORA-BLYP-D3(BJ)/DZP.

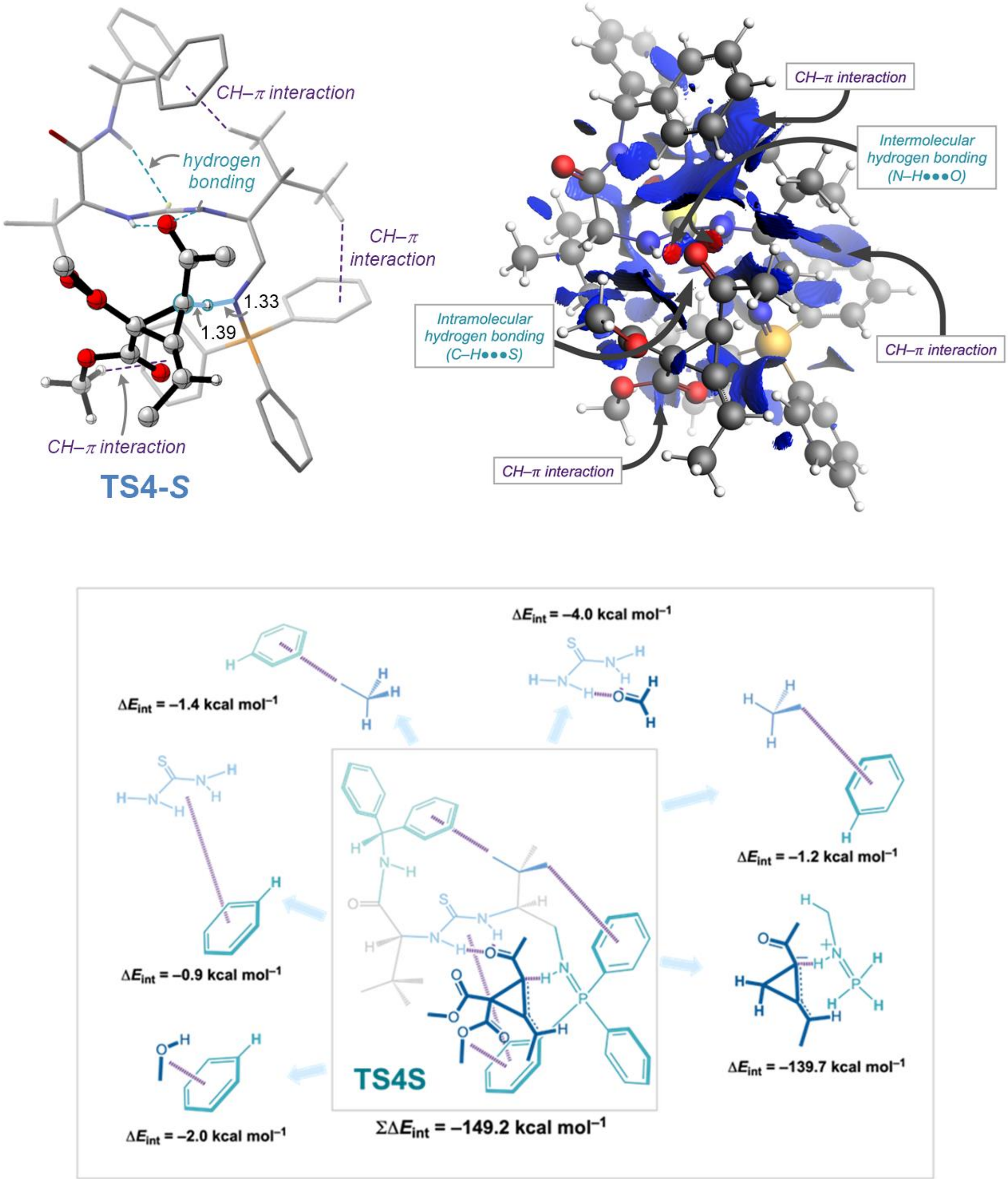

Figure S14: Transition state structure of **TS4-*S*** and the ADFview plot of non-covalent interaction (NCI) regions materialized by reduced density gradient isosurfaces (all default settings were used) colored according to the sign of the signed density *λ2ρ* (red and blue colors are associated with negatively and positively signed terms). Interaction energies in **TS4-*S*** are shown below with truncated fragments using the energy decomposition analysis (EDA). Disconnections were made by capping with a hydrogen atom with an optimized X–H bond length. Computed at ZORA-M06-2X/TZ2P//COSMO(Et_2O)-ZORA-BLYP-D3(BJ)/DZP.

11.3 Computational Details

Table S6. Cartesian coordinates (in Å), energies (in kcal mol^{-1}), and number of imaginary frequencies of all stationary points, computed at COSMO(Et_2O)-ZORA-BLYP-D3(BJ)/DZP. Energies (in kcal mol^{-1}) at COSMO(Et_2O)-ZORA-M06-2X/TZ2P//COSMO(Et_2O)-ZORA-BLYP-D3(BJ)/DZP are also provided.

24a
COSMO(Et_2O)-ZORA-BLYP-D3(BJ)/DZP
E = -4159.37
G = -4046.40
COSMO(Et_2O)-ZORA-M06-2X/TZ2P//COSMO(Et_2O)-ZORA-BLYP-D3(BJ)/DZP
E = -6218.59
G = -6105.62
N_{imag} = 0

C	-0.24706330	0.46103318	0.50853140
C	-0.04171670	1.91516773	0.16056872
C	0.98988053	0.80356164	0.33924935
H	-2.11814394	3.05889310	-3.14216978
C	-0.16568185	2.91254987	1.29165283
C	-0.35914935	2.33366059	-1.25574580
H	-2.20278839	4.72874692	-2.46651525
C	2.44875803	0.61876661	0.30652699
C	-1.25651960	-0.55555982	0.77729617
O	-0.65608645	2.65015600	2.38157421
O	0.40971556	4.09684400	0.96993591
C	0.34844723	5.12383973	2.01602409
H	0.86012732	5.98949074	1.59083588
H	0.85731212	4.76736461	2.91718964
H	-0.69706076	5.35627742	2.24268095
O	0.03334074	1.72811200	-2.24419924
O	-1.18018681	3.41128583	-1.28928232
C	-1.56795821	3.85622211	-2.63254575
H	-0.67443650	4.12175870	-3.20623348
O	-0.99549301	-1.70438593	1.11649532
O	-2.50186412	-0.05305807	0.59270412
C	-3.59274459	-1.00627249	0.83276346
H	-3.49747893	-1.85490709	0.14836335
H	-4.50556357	-0.44082302	0.63706718
H	-3.55590935	-1.35223298	1.87048795
C	3.13270305	1.50162728	-0.75475683
H	2.66766615	-0.44853785	0.15181849
H	2.83303107	0.87588081	1.31012745
H	4.21906814	1.34909058	-0.72410457
H	2.92156190	2.56231592	-0.56219684
H	2.76189214	1.25448640	-1.75693075

C11
COSMO(Et_2O)-ZORA-BLYP-D3(BJ)/DZP
E = -13929.43
G = -13459.55
COSMO(Et_2O)-ZORA-M06-2X/TZ2P//COSMO(Et_2O)-ZORA-BLYP-D3(BJ)/DZP
E = -19784.00
G = -19314.12
N_{imag} = 0

C	-6.14449954	-2.61422710	0.97532564
N	-4.88946181	-2.00739685	1.42166354
H	-4.13501896	-1.98512062	0.73505384
C	-5.45697443	-6.17622828	2.16273527
S	-5.56140268	-2.15029597	4.04613194
N	-3.31037795	-1.21709749	2.84776127
H	-2.92983236	-0.68723090	2.04344372
C	-2.66617057	-0.74663486	4.07389179
H	-3.40734057	-0.80115786	4.88087410
H	-2.85533406	-6.61037107	1.46076343
C	-2.29482245	0.75415529	3.82528116
N	-2.03400206	0.95453459	2.39732632
H	-1.43163953	1.01451361	4.45715221
P	-1.82293565	2.36352072	1.72280267
C	-0.10154263	3.00582333	1.72362072
C	0.94036915	2.06944039	1.81196639
C	0.19545624	4.37453547	1.62491420
C	2.27038261	2.49819005	1.79665885
H	0.69611547	1.01204291	1.89648668
C	1.52679046	4.79996075	1.60690172
H	-0.60966980	5.10541088	1.56656628
C	2.56380812	3.86318864	1.69285535
H	3.07754571	1.76958112	1.86815186
H	1.75579858	5.86257251	1.53029976
H	3.60082335	4.19786292	1.68270446
C	-2.79441037	3.75000279	2.42679423
C	-2.47948436	4.18590988	3.72719737
C	-3.89559747	4.30748224	1.75813628
C	-3.25704988	5.16551200	4.34745013
H	-1.61948651	3.76884882	4.24971812
C	-4.66926113	5.29207584	2.38136528
H	-4.14466551	3.98404878	0.74973977
C	-4.35308349	5.71978060	3.67510319
H	-3.00735200	5.49851873	5.35439922
H	-5.51744016	5.72708892	1.85352106
H	-4.95772764	6.48627627	4.15886009
C	-2.31056089	2.22415453	-0.02751222
C	-3.49143367	1.52089213	-0.32582934
C	-1.58448532	2.83683624	-1.05877952
C	-3.94626544	1.44513734	-1.64254183
H	-4.04559942	1.04644556	0.48075905

C	-2.03711343	2.74596049	-2.37951392
H	-0.66486231	3.37386574	-0.83665996
C	-3.21855559	2.05657853	-2.67111593
H	-1.46561598	3.21463934	-3.17995949
H	-3.57264316	1.99277320	-3.69959148
H	-3.14301170	1.37273433	4.16901628
H	-4.86642598	0.91190048	-1.86869952
H	-0.56635675	-0.22530483	5.88048515
H	-5.36297718	-8.79803425	2.84209469
H	-5.17050284	-6.49821520	1.15120298
H	-6.09681526	0.09157517	0.48616424
H	-6.84765807	-2.47219027	1.80713174
H	-2.29420171	-3.49780034	3.56819349
C	-1.46496949	-1.66841186	4.47138338
C	-0.31265303	-1.56324000	3.44976169
C	-0.96262832	-1.25018961	5.87181785
C	-1.95352767	-3.13083332	4.54445885
H	-0.65694305	-1.82679841	2.44005252
H	0.09698911	-0.54642993	3.41114290
H	0.49810277	-2.25328656	3.72877822
C	-4.53378684	-1.76834044	2.72458774
C	-6.72962215	-1.87416053	-0.26305166
C	-7.01371539	-0.41307070	0.15782868
C	-5.75750942	-1.88405681	-1.46413860
C	-8.06191353	-2.55175176	-0.65381666
H	-7.73475510	-0.37562572	0.98793337
H	-7.43353147	0.14762043	-0.68961694
H	-5.54223347	-2.91014868	-1.78155746
H	-6.20600791	-1.33569999	-2.30588405
H	-4.80873011	-1.38453171	-1.22224342
H	-8.54369400	-1.98657246	-1.46492768
H	-7.89714072	-3.58064701	-0.99482888
H	-8.75312271	-2.57067895	0.20271442
C	-5.92261124	-4.14604718	0.81971717
O	-5.80729392	-4.73354618	-0.26719845
N	-5.83960218	-4.76790721	2.02742808
H	-5.83728154	-4.17341038	2.87164550
H	-1.13586471	-3.78225373	4.88080844
H	-2.79060409	-3.23723322	5.24653691
H	-1.77574490	-1.31112794	6.61138117
H	-0.15547960	-1.92340627	6.19670808
C	-4.24610574	-6.30015953	3.07948431
C	-4.39181081	-6.18601426	4.46878939
C	-3.28270292	-6.30440167	5.30767275
C	-2.01572318	-6.54725232	4.76539880
C	-1.86212738	-6.65318920	3.38068322
C	-2.97411187	-6.52547261	2.54114638
H	-5.38056146	-6.01851618	4.89487994
H	-3.40575658	-6.20807840	6.38603278
H	-1.15045173	-6.64339374	5.42072970

H	-0.87671213	-6.83437030	2.95160848
C	-6.62227404	-7.05704278	2.61951505
C	-7.92560538	-6.56315363	2.73488173
C	-8.96995559	-7.40189384	3.14575765
C	-8.72019900	-8.74501785	3.43992182
C	-7.41729449	-9.24680845	3.32035714
C	-6.37763326	-8.40748195	2.91556930
H	-8.12535394	-5.51829650	2.50704766
H	-9.97939277	-7.00075930	3.23660729
H	-9.53171655	-9.39741334	3.76119358
H	-7.21145575	-10.29274372	3.54774838

Int1

COSMO(Et_2O)-ZORA-BLYP-D3(BJ)/DZP

E = -18118.00

G = -17515.04

COSMO(Et_2O)-ZORA-M06-2X/TZ2P//COSMO(Et_2O)-ZORA-BLYP-D3(BJ)/DZP

E = -26015.04

G = -25412.08

N_{imag} = 0

C	-5.80922779	-2.27692253	2.54933336
N	-4.56698762	-1.49178669	2.65988299
H	-3.88441549	-1.67151780	1.91905679
C	-4.36403441	-5.34175501	4.29416582
S	-4.91158772	-1.08713698	5.31615506
N	-2.68614627	-0.83338606	3.79611015
H	-2.29543058	-0.79285040	2.85139903
C	-1.94889628	-0.05603865	4.80066556
H	-2.70351878	0.29196014	5.51651841
H	-2.94324287	-3.83575478	2.46496528
C	-1.29773408	1.18044332	4.09226891
N	-2.07282628	1.67237341	2.95746216
H	-0.31654591	0.88245745	3.69622250
P	-3.21237824	2.75895481	3.06516977
C	-2.63097969	4.47762501	3.38508394
C	-1.33395463	4.80394607	2.95679027
C	-3.43336534	5.46201064	3.98303631
C	-0.84748562	6.10399096	3.11574909
H	-0.71556730	4.02951746	2.50568326
C	-2.94548111	6.76302885	4.13918292
H	-4.43686811	5.21344411	4.32401941
C	-1.65433774	7.08527189	3.70481492
H	0.15990362	6.35316232	2.78297841
H	-3.57210829	7.52538513	4.60149678
H	-1.27547150	8.09944992	3.82876211
C	-4.55723329	2.54032863	4.29103578
C	-4.24533419	2.63526542	5.65897956
C	-5.86152017	2.20669921	3.90063782
C	-5.22821764	2.39937726	6.61922038
H	-3.23646975	2.90155991	5.97289480

C	-6.84326729	1.97056234	4.86559567
H	-6.11172478	2.13346608	2.84618347
C	-6.52938884	2.06773144	6.22327641
H	-4.98029619	2.47003160	7.67803404
H	-7.85303012	1.70445410	4.55513979
H	-7.29509750	1.87769579	6.97508138
C	-4.00922476	2.81580183	1.42617370
C	-4.19013260	1.61035698	0.73030017
C	-4.42328520	4.02454167	0.84756693
C	-4.77797084	1.61439254	-0.53430214
H	-3.84640245	0.68600544	1.18341515
C	-5.01232097	4.02436592	-0.42078968
H	-4.27202739	4.96486883	1.37361029
C	-5.18994060	2.81996916	-1.10996751
H	-5.32568983	4.96543143	-0.87208084
H	-5.65289775	2.82189838	-2.09532041
H	-1.09776276	1.94020898	4.86680476
H	-4.90318578	0.68359964	-1.08080570
H	0.23181413	0.78372755	6.36729299
H	-5.64761169	-7.61181394	5.12796741
H	-4.70998977	-6.11370107	3.59108702
H	-6.25417195	0.25716719	1.66100883
H	-6.39060652	-2.02809577	3.44651367
H	-2.01732130	-2.81198148	5.38975559
C	-0.91225112	-0.93235597	5.58686270
C	0.22967611	-1.42411036	4.66770289
C	-0.33099832	-0.08326769	6.73996909
C	-1.62436337	-2.16744546	6.18297081
H	-0.17384349	-1.88799572	3.76002561
H	0.90832187	-0.61151676	4.37594745
H	0.82338161	-2.18908733	5.18863673
C	-4.00558569	-1.13375653	3.86786662
C	-6.65859795	-1.86464624	1.31583590
C	-7.11519929	-0.40496458	1.53792398
C	-5.86625643	-1.96430318	-0.00631988
C	-7.90400154	-2.77692391	1.25141371
H	-7.73790823	-0.32506772	2.44090440
H	-7.70036155	-0.05395434	0.67551529
H	-5.56234848	-2.99832402	-0.19900164
H	-6.48999328	-1.61705818	-0.84291644
H	-4.97127786	-1.33117665	0.01387077
H	-8.57626946	-2.43286060	0.45193645
H	-7.62268357	-3.81737788	1.04887235
H	-8.45997916	-2.74159624	2.20091502
C	-5.40330224	-3.77225128	2.64886183
O	-5.29877840	-4.54698100	1.68288988
N	-5.07446855	-4.10697204	3.92586705
H	-5.09854546	-3.35199413	4.62707476
H	-0.91158534	-2.75837419	6.77713381
H	-2.46260453	-1.86508258	6.82317274

H	-1.13501169	0.28269632	7.39702288
H	0.35405989	-0.69304944	7.34814469
C	-2.84082326	-5.20658703	4.13227360
C	-1.96663899	-5.91596397	4.96842404
C	-0.58109115	-5.80998367	4.80634156
C	-0.04975101	-5.00145010	3.79780364
C	-0.91597228	-4.30188376	2.95313495
C	-2.29705791	-4.40193404	3.12132073
H	-2.36634518	-6.54036803	5.76487350
H	0.08228483	-6.35570297	5.47749739
H	1.02910923	-4.90747733	3.67886995
H	-0.52157515	-3.65453652	2.17183809
C	-4.77435853	-5.72696219	5.70572271
C	-4.49184022	-4.86472446	6.77726793
C	-4.86705330	-5.20794732	8.07719563
C	-5.52080611	-6.42308192	8.32287041
C	-5.80008390	-7.28733680	7.26023214
C	-5.42933194	-6.93698716	5.95561772
H	-3.97260573	-3.92559745	6.59385996
H	-4.64695768	-4.52987728	8.90152762
H	-5.81022030	-6.69260769	9.33838668
H	-6.30656050	-8.23468762	7.44390326
C	-1.74236942	-0.41074020	-0.91276160
C	-1.47103409	0.25125506	-2.23384873
C	-1.40221142	0.83920488	-0.82537864
H	1.25944630	-1.36571385	-4.76895064
C	-2.65944566	0.63397834	-3.09159912
C	-0.16457726	-0.07501990	-2.91423027
H	0.66959439	-0.25138316	-6.05834171
C	-1.08247387	2.10232364	-0.15417393
C	-2.08835080	-1.65918441	-0.26438816
O	-3.67260230	-0.03959946	-3.21062578
O	-2.47025494	1.85289343	-3.65685920
C	-3.55082671	2.30395514	-4.53902114
H	-3.25571227	3.30482333	-4.86018279
H	-4.49407621	2.32944393	-3.98780269
H	-3.63561616	1.62418513	-5.39346825
O	0.85431151	-0.38040192	-2.31087303
O	-0.27676068	-0.01567210	-4.26454076
C	0.94632988	-0.34435457	-5.00650522
H	1.73893564	0.36289533	-4.74317273
O	-2.21274466	-1.78046557	0.95741200
O	-2.25297771	-2.65826564	-1.15050425
C	-2.58714610	-3.97025444	-0.56661025
H	-1.76753000	-4.30066953	0.07696605
H	-2.70454916	-4.63084148	-1.42757990
H	-3.51358637	-3.90159557	0.00802589
C	0.30850599	2.63717855	-0.55785154
H	-1.17611762	1.95433167	0.93321243
H	-1.85332670	2.83396221	-0.44992907

H	0.49398550	3.59038105	-0.04572672
H	0.36536080	2.80845707	-1.64134207
H	1.09509238	1.92458147	-0.27945528

Int2

COSMO(Et_2O)-ZORA-BLYP-D3(BJ)/DZP
E = -18114.42
G = -17509.86
COSMO(Et_2O)-ZORA-M06-2X/TZ2P//COSMO(Et_2O)-ZORA-BLYP-D3(BJ)/DZP
E = -26013.70
G = -25409.14
N_{imag} = 0

C	-5.91049159	-2.34336371	2.19486118
N	-4.57588332	-1.82324059	2.50091825
H	-3.87805686	-1.80636770	1.74174533
C	-5.25282893	-5.69273029	3.82566004
S	-5.12659299	-1.39444717	5.11755161
N	-2.76926148	-1.23646313	3.77515069
H	-2.30598314	-1.39962593	2.87131478
C	-2.16305982	-0.16234732	4.57367335
H	-2.96540455	0.25133683	5.19762151
H	-3.76699820	-7.38548679	2.28332861
C	-1.61171681	0.94966501	3.62549728
N	-2.39886404	1.15548642	2.39442275
H	-0.59613990	0.69017482	3.30199582
P	-3.27127878	2.47762245	1.99073825
C	-2.41608165	3.93929358	2.61744620
C	-1.05100624	4.09361158	2.30937368
C	-3.08793176	4.90607541	3.38005227
C	-0.36741742	5.22029066	2.76238885
H	-0.53298857	3.33530973	1.72493349
C	-2.39157826	6.03056943	3.83317846
H	-4.14123946	4.78048176	3.62013048
C	-1.03733835	6.18749208	3.52406974
H	0.68821930	5.34453434	2.52529244
H	-2.90830031	6.78147911	4.42892837
H	-0.49817499	7.06496009	3.87920630
C	-4.97337011	2.51987539	2.60813305
C	-5.29143928	1.90058863	3.82431351
C	-5.97123332	3.16890721	1.85913316
C	-6.60432970	1.93233880	4.29506340
H	-4.53877126	1.36081498	4.38843045
C	-7.27893127	3.20531539	2.34250936
H	-5.73203356	3.62029164	0.89836967
C	-7.59486849	2.58969236	3.55998301
H	-6.84968321	1.42079902	5.22390502
H	-8.05614157	3.69914420	1.76056431
H	-8.62105501	2.60782236	3.92610579
C	-3.35502510	2.54472419	0.18853307
C	-3.61969137	1.36899049	-0.53233367

C	-3.15110305	3.76159289	-0.48198648
C	-3.64751486	1.40797386	-1.92315633
H	-3.75221009	0.42269729	-0.02155034
C	-3.19450234	3.78920168	-1.87744655
H	-2.93976077	4.67139445	0.07636258
C	-3.43222376	2.61365136	-2.59570681
H	-3.02348466	4.72794542	-2.40271792
H	-3.43743855	2.63781179	-3.68432459
H	-1.54548650	1.89533618	4.17369890
H	-3.80778853	0.48741562	-2.47767392
H	0.10960087	1.17146970	5.69571227
H	-3.87593192	-7.07905845	5.78145193
H	-5.69080970	-6.36769803	3.07506731
H	-5.66945487	0.24772285	1.35048761
H	-6.55460781	-2.02694683	3.02638375
H	-2.19881531	-2.50751896	5.98590621
C	-1.05387927	-0.69922170	5.53821309
C	0.06095900	-1.42455813	4.75097136
C	-0.45408016	0.48011103	6.33712345
C	-1.71107459	-1.68837537	6.52398309
H	-0.35826934	-2.20093318	4.09897892
H	0.64954777	-0.73641612	4.12907868
H	0.75556707	-1.90990531	5.45185767
C	-4.12198189	-1.47211522	3.72943227
C	-6.49517949	-1.72615561	0.89172485
C	-6.63742074	-0.20665849	1.12345113
C	-5.60374713	-1.97302198	-0.34762498
C	-7.89418538	-2.33958616	0.66139735
H	-7.30716889	0.00810521	1.96728367
H	-7.04022558	0.28122090	0.22484467
H	-5.51131575	-3.04424908	-0.55562407
H	-6.04951971	-1.47444501	-1.22111534
H	-4.59648571	-1.55962205	-0.21443888
H	-8.37425638	-1.85787250	-0.20280473
H	-7.82671440	-3.41699429	0.46480019
H	-8.53852281	-2.18144945	1.53981566
C	-5.79889659	-3.88743791	2.24324307
O	-5.66901534	-4.62073944	1.25431116
N	-5.78039337	-4.35087800	3.52726621
H	-5.72788666	-3.64711252	4.27665035
H	-0.95125772	-2.10959234	7.19852631
H	-2.48058658	-1.18694373	7.12689876
H	-1.24496564	1.04759452	6.85142782
H	0.23894176	0.09849686	7.10104041
C	-3.73879098	-5.72844481	3.65942738
C	-2.93033665	-4.81491880	4.34910042
C	-1.54133518	-4.87920037	4.23647057
C	-0.94397333	-5.84627148	3.41890407
C	-1.74656923	-6.74643676	2.71290301
C	-3.13941988	-6.68525465	2.83486516

H	-3.39013612	-4.05578313	4.97957383
H	-0.92143807	-4.17732977	4.78785937
H	0.14108067	-5.88855518	3.32551652
H	-1.29071148	-7.49179034	2.06132602
C	-5.73098182	-6.13350814	5.20854684
C	-7.04651711	-5.85107299	5.61116381
C	-7.51357209	-6.26654371	6.86070676
C	-6.67280590	-6.97696528	7.72597649
C	-5.36531592	-7.27076146	7.32755993
C	-4.89746790	-6.85140052	6.07676441
H	-7.70299544	-5.29486637	4.94361905
H	-8.53546622	-6.03391884	7.15992694
H	-7.03486928	-7.29748213	8.70246388
H	-4.70181700	-7.82361451	7.99232559
C	-0.81152618	-0.72698130	-0.71698259
C	0.11220749	-0.69093989	-1.89466206
C	-0.38774111	0.51886232	-1.08964418
H	3.87508470	-2.14325873	-2.46905444
C	-0.47354378	-1.01049487	-3.24168875
C	1.56415784	-1.04342037	-1.68871978
H	4.12680019	-0.85584502	-3.70387710
C	-0.22351817	1.84711911	-1.08283841
C	-1.56500123	-1.57491074	0.04831724
O	-0.20492778	-2.01380116	-3.89370560
O	-1.39049692	-0.08025141	-3.63612265
C	-2.10955091	-0.40935237	-4.86209300
H	-2.81073735	0.41518833	-5.01113524
H	-2.64372811	-1.35907254	-4.74514227
H	-1.41252546	-0.48068640	-5.70460536
O	2.05858964	-1.41898792	-0.63516908
O	2.28641792	-0.80804624	-2.82432265
C	3.71727829	-1.08836753	-2.71808485
H	4.16843197	-0.45427735	-1.94728947
O	-2.33111327	-1.20237637	1.01649326
O	-1.47913366	-2.91407904	-0.27815474
C	-2.23346328	-3.83060972	0.56927955
H	-1.96889269	-3.70748364	1.62411538
H	-1.94260714	-4.82939295	0.23386670
H	-3.31166211	-3.69973372	0.43896659
C	0.55437315	2.59434195	-2.13024709
H	-2.35695009	0.35843615	1.71169473
H	-0.75301755	2.43315555	-0.33494881
H	1.29752989	3.27821641	-1.68715439
H	-0.11688774	3.21118125	-2.75349969
H	1.08367115	1.89794140	-2.79439889

24b
COSMO(Et_2O)-ZORA-BLYP-D3(BJ)/DZP
E = -4168.12
G = -4053.83

COSMO(Et_2O)-ZORA-M06-2X/TZ2P//COSMO(Et_2O)-ZORA-BLYP-D3(BJ)/DZP
E = -6228.12
G = -6113.83
N_{imag} = 0

C	-0.11626913	0.15285461	0.21707659
C	0.12782850	1.69540334	-0.03915171
C	1.21346424	0.71133334	-0.01026350
H	-0.02021137	2.72478259	-3.96180184
C	-0.01819034	2.66764468	1.12211894
C	-0.33545215	2.28119979	-1.34939010
H	-0.56080897	1.04869798	-4.35031558
C	2.52366349	0.54562119	-0.06823232
C	-0.53027629	-0.24128643	1.60351749
O	0.89989053	3.32071043	1.58760958
O	-1.30449866	2.71402448	1.53464826
C	-1.56067943	3.61795407	2.66221602
H	-2.63306519	3.53721087	2.84939963
H	-1.28266231	4.64003797	2.38681375
H	-0.98081529	3.28797972	3.52990029
O	-0.69615449	3.44428813	-1.46761684
O	-0.27026567	1.38336730	-2.35839897
C	-0.67514431	1.89696356	-3.67304067
H	-1.71527659	2.23454278	-3.63333075
O	0.00977425	0.13380744	2.63314982
O	-1.60827696	-1.06986992	1.55249806
C	-2.10958082	-1.50582664	2.86203025
H	-1.32755207	-2.05838150	3.39229202
H	-2.96258663	-2.14862222	2.63668790
H	-2.41574625	-0.63368548	3.44809755
C	3.49682179	1.66249357	-0.30597489
H	4.19986285	1.74056881	0.53756844
H	2.97933573	2.62268082	-0.41570187
H	4.09714164	1.46673665	-1.20795392
H	2.92582239	-0.46240874	0.06778049
H	-0.62492638	-0.36680733	-0.59560943

SM
COSMO(Et_2O)-ZORA-BLYP-D3(BJ)/DZP
E = -4011.02
G = -3900.42
COSMO(Et_2O)-ZORA-M06-2X/TZ2P//COSMO(Et_2O)-ZORA-BLYP-D3(BJ)/DZP
E = -5946.47
G = -5835.87
N_{imag} = 0

C	-0.27477435	0.45133390	0.43307219
C	-0.05709304	1.91648718	0.10481878
C	0.96681100	0.80259608	0.29859957
H	-2.03369004	3.16143402	-3.21896153
C	-0.23272650	2.89904916	1.24116856
C	-0.33782351	2.35100832	-1.31125200

H	-2.06395793	4.82894804	-2.53315853
C	2.42644664	0.62417215	0.29770088
C	-1.34121973	-0.52463340	0.67412443
O	-0.83766419	2.64197780	2.27417191
O	0.41834832	4.06186744	0.99802960
C	0.29599105	5.07744240	2.05054474
H	0.87185237	5.93065800	1.68673911
H	0.70953316	4.69222626	2.98792129
H	-0.75764075	5.34184476	2.18572029
O	0.03426795	1.73006277	-2.29826090
O	-1.10955971	3.46603880	-1.34989006
C	-1.46086363	3.93352942	-2.69572096
H	-0.54966861	4.16786976	-3.25495536
O	-1.06602352	-1.69089367	0.97227053
C	-2.74368994	0.00663259	0.53509177
H	2.78672646	0.88135016	1.31041751
H	4.21561205	1.36917330	-0.68907561
H	2.90520940	2.57208614	-0.55742459
H	2.78547675	1.26352292	-1.75720297
C	3.12917943	1.51328829	-0.74590806
H	2.65252757	-0.44199164	0.14594326
H	-2.88299666	0.84503012	1.23352593
H	-3.47436652	-0.78433738	0.73319735
H	-2.88698654	0.40743580	-0.48013671

C12

COSMO(Et_2O)-ZORA-BLYP-D3(BJ)/DZP

E = -13943.78

G = -13470.87

COSMO(Et_2O)-ZORA-M06-2X/TZ2P//COSMO(Et_2O)-ZORA-BLYP-D3(BJ)/DZP

E = -19793.29

G = -19320.38

$N_{imag} = 0$

C	-5.84722758	-2.56253422	0.50719489
N	-4.65456903	-1.95003637	1.10576656
H	-4.16925979	-1.26676617	0.52228757
C	-8.35557542	-0.01070723	1.81419284
S	-5.34285573	-2.78963531	3.58901650
N	-3.32266864	-1.17278187	2.80032237
H	-2.84647983	-0.65055059	2.06494344
C	-2.71928989	-1.08090911	4.13681501
H	-3.40000137	-1.63299776	4.79814013
H	-9.70862635	1.04967193	4.00559480
C	-2.74451962	0.38976771	4.61751024
N	-1.77336709	1.25542725	3.95412763
H	-2.52870776	0.38140485	5.69855333
P	-2.12531671	2.35004180	2.87975159
C	-0.53493642	3.01658143	2.28130551
C	0.65859164	2.58055134	2.87422305
C	-0.49654927	3.95539864	1.23858944

C	1.88413981	3.07831249	2.42191170
H	0.60449206	1.85309695	3.68074265
C	0.73036625	4.44761803	0.78700025
H	-1.41711480	4.29265933	0.76664540
C	1.92193233	4.00959527	1.37794211
H	2.81055825	2.73633723	2.88276383
H	0.75703756	5.17102629	-0.02731657
H	2.87818824	4.39423286	1.02418809
C	-3.07809766	3.81547875	3.46944254
C	-3.08551803	4.04115843	4.85461447
C	-3.70585425	4.73491654	2.61392917
C	-3.70774350	5.17739596	5.37892893
H	-2.59739891	3.32203657	5.51123686
C	-4.32659401	5.86997689	3.13991790
H	-3.72577527	4.56275369	1.53993164
C	-4.32544144	6.09470866	4.52135302
H	-3.71414575	5.34536813	6.45569143
H	-4.81743120	6.57703444	2.47178076
H	-4.81318791	6.97960437	4.92952151
C	-3.00986515	1.81973707	1.35564524
C	-4.41244136	1.86390517	1.27725732
C	-2.27719298	1.22909545	0.30853958
C	-5.06809198	1.36422266	0.15161783
H	-4.99199114	2.29110560	2.08820859
C	-2.93932840	0.72598226	-0.81553812
H	-1.19067408	1.17576940	0.36596468
C	-4.33489019	0.80700493	-0.90035634
H	-2.36583184	0.27990597	-1.62773264
H	-4.85566768	0.41921168	-1.77189277
H	-3.78910224	0.72405901	4.51483164
H	-6.15219555	1.39367646	0.09552679
H	-0.33017490	-0.44606065	5.54850033
H	-8.48225679	2.65941845	1.33502563
H	-8.61276194	0.36395083	0.81244995
H	-3.89227764	-2.14161403	-1.54760423
H	-7.44604388	4.51443523	2.62826155
H	-2.16090690	-3.59027384	3.16642886
C	-1.33421770	-1.81934426	4.16713050
C	-0.41691599	-1.41186462	2.98926937
C	-0.61938015	-1.50410662	5.49875502
C	-1.60425570	-3.33960098	4.07994028
H	-0.85544925	-1.70038969	2.02233794
H	-0.24106664	-0.33213816	2.98965316
H	0.54682917	-1.93602592	3.07907170
C	-4.38755751	-1.92481575	2.44782685
C	-5.51746705	-3.39518677	-0.76340425
C	-4.85465454	-2.55788325	-1.87741566
C	-4.55695926	-4.52981872	-0.33639605
C	-6.83544877	-4.01391545	-1.28171314
H	-5.50970216	-1.73710685	-2.18667881

H	-4.65094488	-3.19962515	-2.74751741
H	-5.01688060	-5.16494142	0.43487027
H	-4.31297182	-5.15993620	-1.20373930
H	-3.62159427	-4.12248724	0.07066474
H	-6.62388087	-4.69307573	-2.12012638
H	-7.33007467	-4.59682858	-0.48975473
H	-7.52721468	-3.23677243	-1.62928894
C	-6.92858691	-1.45648667	0.36959478
O	-7.23285934	-0.88633270	-0.68872337
N	-7.46946158	-1.15372545	1.58101796
H	-7.11914874	-1.67386835	2.39707016
H	-0.65270726	-3.89198590	4.06517072
H	-2.19567191	-3.68562547	4.93981297
H	-1.26521021	-1.73413495	6.35985200
H	0.29088797	-2.11606859	5.58546265
C	-9.64906173	-0.48527498	2.48764339
C	-10.30885271	-1.61016486	1.96599590
C	-11.49974662	-2.06443779	2.53561834
C	-12.04915169	-1.39994831	3.64004488
C	-11.39914281	-0.27896321	4.16267995
C	-10.20573488	0.17743091	3.58826037
H	-9.87889720	-2.13510536	1.11401584
H	-11.99937938	-2.93930560	2.11993781
H	-12.97587796	-1.75649717	4.08878806
H	-11.81640416	0.24426012	5.02283151
C	-7.65732551	1.10479967	2.58218203
C	-6.85836401	0.82789446	3.70020609
C	-6.29857353	1.87082841	4.44234847
C	-6.51468757	3.20005917	4.06680831
C	-7.29300630	3.48189260	2.94093533
C	-7.86651383	2.43737127	2.20750292
H	-6.66083786	-0.20218264	3.99456622
H	-5.68243018	1.64647078	5.31172278
H	-6.06168022	4.00790969	4.63447846
H	-6.20875456	-3.27087597	1.26372503

Int3
COSMO(Et_2O)-ZORA-BLYP-D3(BJ)/DZP
E = -17971.72
G = -17371.01
COSMO(Et_2O)-ZORA-M06-2X/TZ2P//COSMO(Et_2O)-ZORA-BLYP-D3(BJ)/DZP
E = -25743.35
G = -25142.64
N_{imag} = 0

C	-5.17766279	-2.03193760	2.52694621
N	-3.84558358	-1.48141802	2.78614505
H	-3.12841097	-1.62701184	2.07450825
C	-7.47228430	1.01131241	2.29056662
S	-4.51868663	-0.71091148	5.28437244
N	-2.08013845	-0.86273068	4.10823294

H	-1.56138046	-0.99516578	3.23387359
C	-1.40193357	-0.03479520	5.11722562
H	-2.19594779	0.46087154	5.68977631
H	-8.56296303	-1.45999862	2.95599395
C	-0.57543330	1.04688782	4.36868359
N	-1.37014150	1.73819588	3.35744461
H	0.26262209	0.55539856	3.84837053
P	-2.24786413	3.01492952	3.66590068
C	-1.36471110	4.63413431	3.62421866
C	-1.89995556	5.79949252	4.19690321
C	-0.13594791	4.69285431	2.95098356
C	-1.21792469	7.01361793	4.08173303
H	-2.84567544	5.75440470	4.73579354
C	0.54712434	5.90731409	2.83822241
H	0.27761176	3.77897086	2.52803198
C	0.00429843	7.06897116	3.39966974
H	-1.63683039	7.91616584	4.52652826
H	1.50320087	5.94849766	2.31670725
H	0.53618094	8.01602014	3.31188180
C	-3.08505187	3.06342744	5.28849779
C	-2.29291881	3.34287424	6.42127103
C	-4.40785891	2.63438361	5.45553384
C	-2.82136353	3.18871737	7.70146457
H	-1.26451922	3.67977972	6.29812572
C	-4.93599954	2.49556164	6.74181975
H	-5.01961875	2.40046215	4.59126313
C	-4.14834153	2.76535598	7.86175107
H	-2.20347263	3.39878778	8.57431346
H	-5.96160238	2.15788977	6.86379753
H	-4.56279765	2.64258010	8.86239154
C	-3.43277236	3.11480964	2.27558223
C	-3.89257755	1.90715160	1.72386817
C	-3.73120097	4.32777524	1.63677319
C	-4.61144049	1.90939854	0.52796409
H	-3.63455826	0.97028110	2.20542191
C	-4.45708032	4.32746137	0.44181928
H	-3.36173263	5.26799471	2.03968487
C	-4.88593765	3.11930708	-0.11819886
H	-4.67289937	5.27052976	-0.06028591
H	-5.43875648	3.12706588	-1.05548568
H	-0.12275973	1.71037945	5.12217214
H	-4.95143552	0.96513133	0.10775370
H	0.83391440	0.67409971	6.77260353
H	-6.42663834	3.50322197	1.93081684
H	-7.12390633	1.37365234	1.31425820
H	-3.26378294	-3.24796644	0.66732199
H	-0.68129194	-3.65297473	-0.30921737
H	-1.98340779	-2.57339632	6.09302108
C	-0.56555033	-0.90185399	6.11820494
C	0.54714331	-1.68819814	5.39053959

C	0.05285805	0.02479393	7.19039007
C	-1.50604503	-1.90505985	6.82170688
H	0.12346243	-2.32708139	4.60193505
H	1.29119294	-1.02139155	4.93383491
H	1.07385309	-2.33896420	6.10470129
C	-3.42137134	-1.03453815	4.00826684
C	-5.12108485	-3.41109920	1.80876592
C	-4.33915277	-3.34829170	0.47687315
C	-4.42139961	-4.40549342	2.76516698
C	-6.57108041	-3.88853313	1.56142315
H	-4.67134035	-2.51363415	-0.14878849
H	-4.47570402	-4.28939226	-0.07696009
H	-4.98992071	-4.51918071	3.70023533
H	-4.34239762	-5.39250656	2.28646482
H	-3.40804222	-4.06595381	3.02020114
H	-6.56111060	-4.91965305	1.17897966
H	-7.14879804	-3.88142900	2.49861526
H	-7.08061999	-3.24789049	0.83280456
C	-6.06041268	-0.95703475	1.83506097
O	-6.31764124	-0.92727471	0.62302381
N	-6.54380362	-0.03885622	2.71644973
H	-6.19042051	-0.06598730	3.68567270
H	-0.93325049	-2.51657808	7.53558834
H	-2.30343543	-1.37912388	7.36299383
H	-0.72005570	0.66395285	7.64317769
H	0.50972339	-0.57847458	7.98930597
C	-8.89947823	0.48656355	2.09775899
C	-9.84982496	1.33895711	1.51636914
C	-11.17108101	0.92064058	1.34591687
C	-11.56041785	-0.36075231	1.75944167
C	-10.61703684	-1.21277307	2.34081846
C	-9.29219048	-0.79055394	2.50673959
H	-9.55366303	2.34207636	1.20808929
H	-11.89877367	1.59295320	0.89161316
H	-12.59097211	-0.68935442	1.62823203
H	-10.90906866	-2.21168644	2.66480571
C	-7.45408044	2.17309244	3.27437579
C	-8.04327233	2.04428641	4.54082749
C	-8.04900762	3.12252014	5.42788422
C	-7.46356728	4.34009767	5.06061918
C	-6.87915986	4.47379574	3.79887659
C	-6.88070094	3.39523228	2.90971060
H	-8.51317269	1.10314209	4.82503246
H	-8.50721425	3.01312427	6.41065729
H	-7.45684639	5.17531669	5.76017538
H	-6.41145208	5.41333861	3.50589229
C	-1.38287697	-0.29970212	-0.41815784
C	-1.71823242	0.31769001	-1.75740139
C	-1.36591355	1.00122206	-0.44189259
H	0.32209687	-0.75043892	-5.09729876

C	-3.17315169	0.22160471	-2.17113551
C	-0.66437694	0.26889318	-2.83221422
H	-0.86323672	0.18417225	-6.08364681
C	-1.05519157	2.32762728	0.09886793
C	-1.14165968	-1.60899680	0.16288556
O	-3.86615777	-0.76256872	-1.95907857
O	-3.61081429	1.36044256	-2.76018428
C	-5.00449766	1.31319499	-3.21976883
H	-5.21854819	2.32162204	-3.57885235
H	-5.66178129	1.03510164	-2.39078254
H	-5.09822092	0.57950511	-4.02786269
O	0.53493064	0.19704777	-2.59983946
O	-1.21208505	0.26834919	-4.07388249
C	-0.25122106	0.17826486	-5.17975756
H	0.42314451	1.03970328	-5.15417400
O	-1.15552892	-1.79412885	1.39039018
C	-0.90497629	-2.71539929	-0.82802294
H	-1.93687260	2.97012156	-0.04095003
H	0.34024392	3.96347571	-0.17206438
H	-0.03941356	3.10566622	-1.68714411
H	1.05301140	2.35673874	-0.49989744
C	0.15438766	2.97591586	-0.61416231
H	-0.88608128	2.21104668	1.17890412
H	-0.07722519	-2.43880347	-1.49833258
H	-1.80285251	-2.82845747	-1.45379018
H	-5.61878121	-2.20085534	3.51901398

Int4

COSMO(Et_2O)-ZORA-BLYP-D3(BJ)/DZP
E = -17971.78
G = -17371.07
COSMO(Et_2O)-ZORA-M06-2X/TZ2P//COSMO(Et_2O)-ZORA-BLYP-D3(BJ)/DZP
E = -25744.53
G = -25143.82
N_{imag} = 0

C	-1.06205047	-2.36986707	3.97743234
N	-2.53695757	-2.20826958	3.89301400
H	-3.01741693	-3.06762623	4.22909894
C	-0.80449326	-3.17033399	0.24183853
S	-2.90964628	0.02614592	2.34111368
N	-4.51983509	-2.08971318	2.78779112
H	-4.61173774	-3.04891948	3.17011772
C	-5.74639684	-1.41318965	2.37456661
H	-5.47475537	-0.36337151	2.20462849
H	-2.43056174	-4.40333813	2.12155009
C	-6.72286033	-1.46554131	3.56649385
N	-6.15707929	-0.70528317	4.69297207
H	-6.89455064	-2.49807662	3.89548776
P	-6.82470731	0.62418396	5.37312417
C	-7.90301465	0.26739178	6.78523398

C	-7.69545050	-0.93636920	7.48051602
C	-8.86714302	1.18982487	7.22720010
C	-8.45538671	-1.21011859	8.61928872
H	-6.94550978	-1.64885755	7.14596455
C	-9.62322260	0.90346893	8.36551545
H	-9.02735468	2.12221882	6.68789747
C	-9.41670158	-0.29445171	9.06088031
H	-8.29191947	-2.14136711	9.15746430
H	-10.37335285	1.61437599	8.70950425
H	-10.00853674	-0.51409171	9.94896661
C	-7.79966352	1.42460863	4.07464177
C	-9.15142330	1.08466602	3.89290140
C	-7.15077308	2.26595437	3.15494311
C	-9.84527676	1.58241565	2.78842591
H	-9.65342108	0.42642171	4.59899238
C	-7.85398557	2.75763480	2.05466263
H	-6.10568320	2.53222113	3.29340988
C	-9.19712948	2.41360379	1.86923265
H	-10.89079331	1.31445667	2.64325422
H	-7.35044701	3.40689344	1.33995409
H	-9.74045699	2.79489629	1.00550830
C	-5.48370544	1.69398731	5.94618502
C	-4.31352381	1.77724561	5.17488246
C	-5.59539919	2.40569067	7.15102715
C	-3.26400094	2.58783087	5.60499806
H	-4.19548004	1.18687295	4.26777458
C	-4.53719020	3.21380989	7.57176749
H	-6.48438550	2.31324886	7.76985680
C	-3.37387914	3.30741077	6.79952540
H	-4.61655943	3.75892157	8.51154384
H	-2.54663655	3.93065274	7.13820075
H	-7.68971030	-1.04157790	3.28575017
H	-2.35159441	2.63490531	5.01255474
H	-8.33671022	-1.05252172	1.28076035
H	1.25085589	-3.04569940	-1.55887467
H	0.07610286	-3.82520655	0.30956161
H	-0.41369970	0.26323239	2.94724233
H	-7.15604268	-5.16277305	5.64338705
H	-4.40162440	-2.61851138	0.24668836
C	-6.33661273	-1.96899074	1.04076116
C	-6.86268447	-3.41005975	1.21735934
C	-7.48428976	-1.03526691	0.58809281
C	-5.22365901	-1.95180409	-0.02945369
H	-6.08198938	-4.06735613	1.61772354
H	-7.73129546	-3.44588392	1.89037189
H	-7.17190989	-3.82049990	0.24488608
C	-3.32952190	-1.48667900	3.04767250
C	-0.18153801	-1.19641263	4.56363830
C	0.38872564	-0.23746880	3.49774127
C	-1.02067639	-0.42445765	5.59735022

C	1.01040080	-1.87700719	5.28516064
H	1.02440195	-0.77443567	2.77919165
H	1.00840748	0.52346877	3.99649310
H	-1.37744986	-1.10960872	6.37418715
H	-0.40122657	0.35164668	6.07203581
H	-1.88396397	0.05426049	5.12477784
H	1.68089679	-1.10924245	5.69891561
H	0.65310803	-2.51219127	6.10749226
H	1.58689053	-2.50185477	4.58861554
C	-0.48096784	-3.01411493	2.69626353
O	0.26903222	-4.00447442	2.76666447
N	-0.88891271	-2.46423216	1.52555888
H	-1.49163141	-1.63350020	1.56499034
H	-5.62439509	-2.28638564	-0.99696298
H	-4.81588628	-0.93813566	-0.14512468
H	-7.13524511	0.00495275	0.50886709
H	-7.85408201	-1.34847872	-0.39942609
C	-2.04274616	-4.05971475	0.01676358
C	-2.48791898	-4.34942550	-1.28128016
C	-3.60620080	-5.16454771	-1.48630745
C	-4.29345918	-5.70582313	-0.39543063
C	-3.84964102	-5.42988507	0.90095059
C	-2.73453269	-4.61429323	1.10300368
H	-1.97337795	-3.91863066	-2.13824290
H	-3.94692607	-5.36479716	-2.50237412
H	-5.17541529	-6.32590894	-0.55515560
H	-4.37486639	-5.81835944	1.77240920
C	-0.59599749	-2.16526548	-0.87849513
C	-1.53787374	-1.14761991	-1.09483287
C	-1.35978227	-0.22849542	-2.13008217
C	-0.24287510	-0.32303254	-2.97123214
C	0.69530042	-1.33895621	-2.76454371
C	0.51866173	-2.25404364	-1.71886173
H	-2.41166092	-1.07094600	-0.45153647
H	-2.09476304	0.56137009	-2.28524526
H	-0.10642017	0.39166858	-3.78255351
H	1.56625074	-1.42001781	-3.41474930
C	-5.05421703	-3.81464821	6.30188946
C	-3.94622997	-3.29915612	7.19785754
C	-5.33096295	-3.81502348	7.64749216
H	-1.08560532	-6.03507102	8.29710614
C	-3.79004839	-1.82136041	7.37177880
C	-2.71028579	-4.13654367	7.32066446
H	-2.25729976	-7.32025971	7.82414280
C	-6.02348797	-3.91674494	8.78850527
C	-5.14808501	-4.48815889	5.09896064
O	-4.41469662	-0.97260821	6.74275598
O	-2.96306242	-1.48984975	8.39227746
C	-2.88947927	-0.04819685	8.66083149
H	-2.14163850	0.05366438	9.45039789

H	-3.86930129	0.31346285	8.98995728
H	-2.59118988	0.48608507	7.75613468
O	-1.57869178	-3.72852438	7.08319338
O	-2.99547761	-5.42784799	7.64345814
C	-1.85215709	-6.33675408	7.57549142
H	-1.43139845	-6.32983809	6.56436813
O	-4.24667716	-4.46410766	4.17631767
C	-6.41591289	-5.28921340	4.84293527
H	-7.04455163	-4.30692284	8.74146951
H	-5.44883946	-4.38728322	10.82893762
H	-6.09638817	-2.75349788	10.61571016
H	-4.45586463	-3.13514048	10.04223637
C	-5.47605824	-3.52966072	10.13597301
H	-5.41682694	-1.15112290	5.26582884
H	-6.15354234	-6.35591175	4.76371254
H	-6.86188247	-4.99294517	3.88301704
H	-0.97992998	-3.16867774	4.72235134

P
COSMO(Et_2O)-ZORA-BLYP-D3(BJ)/DZP
E = -4018.87
G = -3905.88
COSMO(Et_2O)-ZORA-M06-2X/TZ2P//COSMO(Et_2O)-ZORA-BLYP-D3(BJ)/DZP
E = -5954.64
G = -5841.65
N_{imag} = 0

C	-0.50496586	0.76249325	0.82496233
C	0.08984039	2.12436095	0.28180629
C	0.89960669	0.91401412	0.46931410
H	-1.83934553	2.44378239	-3.27348411
C	0.21182374	3.21924571	1.31612647
C	-0.25745939	2.55816835	-1.13160337
H	-3.10586991	3.42612948	-2.44650388
C	2.10142746	0.39109913	0.29842115
C	-1.46672489	0.03745206	-0.08896192
O	0.28594844	3.02813357	2.52020855
O	0.25345306	4.43359468	0.71794654
C	0.39575333	5.57634261	1.63107981
H	0.40738315	6.45244054	0.98049193
H	1.33205844	5.48566281	2.19002205
H	-0.45480899	5.60408947	2.31866017
O	0.54752610	2.65368281	-2.04231335
O	-1.58062877	2.83332917	-1.22415526
C	-2.03461936	3.24767005	-2.55690350
H	-1.51034857	4.15953730	-2.85953649
O	-1.14671593	-0.29720761	-1.22764934
C	-2.83949291	-0.20222330	0.48706975
H	-2.76191735	-0.80195782	1.40656405
H	4.10449072	1.14742546	0.49958371
H	3.00819478	2.19419073	-0.45659254

H	3.65795228	0.68636722	-1.15014340
C	3.27919907	1.15631121	-0.22930357
H	-3.47762617	-0.71192529	-0.24254230
H	-3.28499172	0.76361093	0.77150361
H	-0.78182624	0.81567246	1.88095880
H	2.24891974	-0.66267125	0.55162182

TS1-depro-LA1-RA1-Ha (TS1)
COSMO(Et_2O)-ZORA-BLYP-D3(BJ)/DZP
E = -18106.04
G = -17504.18
COSMO(Et_2O)-ZORA-M06-2X/TZ2P//COSMO(Et_2O)-ZORA-BLYP-D3(BJ)/DZP
E = -25999.87
G = -25398.01
N_{imag} = 1, 1574 i cm^{-1}

C	-6.28125149	-2.27744247	2.49276532
N	-5.09046372	-1.42342164	2.44103782
H	-4.61189622	-1.39451090	1.53055142
C	-4.61178241	-5.59770467	3.38469091
S	-4.79196138	-1.44529507	5.13594075
N	-3.07857209	-0.65763519	3.18940412
H	-2.99194174	-0.36938957	2.21392212
C	-2.08455463	-0.05883115	4.08249243
H	-2.60134419	0.18445354	5.01943414
H	-2.08380520	-5.59605332	2.34871877
C	-1.53544618	1.25423040	3.42333085
N	-2.48444013	1.93205210	2.52747636
H	-0.67010784	1.01761794	2.79523850
P	-3.66421607	2.90551795	3.04498538
C	-3.07703963	4.55276438	3.57115955
C	-1.81185067	4.97573237	3.13486405
C	-3.86063188	5.39536647	4.37780332
C	-1.33846428	6.24041002	3.49239072
H	-1.20303378	4.31361787	2.52489746
C	-3.38635380	6.66224265	4.72423792
H	-4.83049915	5.05956597	4.74064187
C	-2.12666173	7.08497096	4.28196779
H	-0.35373632	6.56463414	3.15796354
H	-3.99578980	7.31726304	5.34589123
H	-1.75648562	8.07121245	4.55987676
C	-4.64917439	2.31950898	4.45369305
C	-4.07550417	2.32743766	5.73819684
C	-5.97506723	1.89942487	4.27953312
C	-4.82842871	1.91190977	6.83466148
H	-3.05355507	2.67478247	5.88226501
C	-6.72260659	1.48689702	5.38367208
H	-6.42352269	1.90206516	3.29154033
C	-6.15335663	1.49642950	6.65882663
H	-4.38222596	1.91276382	7.82846715
H	-7.75020935	1.15545273	5.24474393

H	-6.73845890	1.17087641	7.51833446
C	-4.77892110	3.11123659	1.62872948
C	-4.98076055	2.02161390	0.76343803
C	-5.46856012	4.31577941	1.42064574
C	-5.87852979	2.14063224	-0.29781947
H	-4.42863006	1.09721763	0.90051410
C	-6.36532477	4.42461178	0.35479726
H	-5.30069741	5.16773644	2.07563896
C	-6.57332128	3.33730611	-0.50165824
H	-6.89757818	5.36082247	0.19068513
H	-7.27371293	3.42600421	-1.33156619
H	-1.16612021	1.91219191	4.22399309
H	-6.03293176	1.29519458	-0.96577329
H	0.75659480	0.37747704	4.37988404
H	-4.07251339	-8.22162186	3.78673800
H	-4.41960439	-5.84276849	2.32972394
H	-6.96353585	0.34041011	1.92120684
H	-6.65476268	-2.20097908	3.52299532
H	-2.23896445	-2.72901075	4.97820157
C	-0.92039311	-1.06581381	4.41399797
C	-0.43726311	-1.77405669	3.13189363
C	0.25312731	-0.30460836	5.07689166
C	-1.41946274	-2.13947553	5.40116702
H	-1.21039286	-2.44827760	2.74734760
H	-0.18124214	-1.06592672	2.33859797
H	0.45479480	-2.37816599	3.34959774
C	-4.29810203	-1.15728006	3.52769544
C	-7.40710152	-1.76725368	1.54900927
C	-7.80599522	-0.35089846	2.02196232
C	-6.95907334	-1.69665343	0.07148533
C	-8.62247848	-2.71017689	1.69545208
H	-8.12135032	-0.36025654	3.07549125
H	-8.63752346	0.02977729	1.41118668
H	-6.67919966	-2.68899406	-0.29785752
H	-7.78285086	-1.30189411	-0.54201032
H	-6.09986497	-1.02622663	-0.05385647
H	-9.46692623	-2.32644567	1.10451025
H	-8.38346710	-3.72156860	1.34474370
H	-8.94406579	-2.76973833	2.74693385
C	-5.82169464	-3.74569897	2.27383055
O	-5.95204539	-4.38110516	1.21696036
N	-5.21202880	-4.26089944	3.37954009
H	-5.06117781	-3.63934835	4.18760783
H	-0.59727723	-2.82604695	5.64246006
H	-1.78296630	-1.68216451	6.33211946
H	-0.09437248	0.27616734	5.94556618
H	1.00039344	-1.02813866	5.43497353
C	-3.28750379	-5.57402988	4.13638738
C	-3.26639897	-5.55930011	5.53798552
C	-2.05197947	-5.55647875	6.22668791

C	-0.84404084	-5.57482086	5.52045637
C	-0.85739765	-5.57890712	4.12362477
C	-2.07552875	-5.57905312	3.43675287
H	-4.20555026	-5.56811639	6.09055655
H	-2.04712864	-5.54210107	7.31645220
H	0.10341118	-5.57559119	6.05884952
H	0.07869654	-5.58118824	3.56593876
C	-5.56358360	-6.66571075	3.93347718
C	-6.87837885	-6.36898991	4.30427075
C	-7.72001793	-7.37275142	4.80110375
C	-7.25407047	-8.68425391	4.92591855
C	-5.93855633	-8.98838532	4.55019874
C	-5.10067891	-7.98479860	4.06024032
H	-7.24633226	-5.35006213	4.20942976
H	-8.74145197	-7.12559891	5.09061458
H	-7.90803526	-9.46529129	5.31295853
H	-5.56522091	-10.00801227	4.64419692
C	-1.34016183	-0.15469696	-0.31381546
C	0.06782299	0.31995091	-0.53602431
C	-1.18857814	1.16347856	-0.28868101
H	2.42499820	-1.00608900	2.51066078
C	0.60589599	0.32971613	-1.95000225
C	1.08410101	0.25616925	0.58217849
H	3.39532446	-1.92435429	1.30089899
C	-1.60268747	2.47059142	-0.02186123
C	-2.26503821	-1.19974655	-0.08086592
O	-0.05492729	0.07398969	-2.94599960
O	1.90253047	0.74108872	-1.97345101
C	2.50599205	0.82895014	-3.30569996
H	3.52909618	1.16817178	-3.13069167
H	1.95253597	1.54781297	-3.91839744
H	2.49481566	-0.15594989	-3.78360609
O	1.17697643	1.07573128	1.48831508
O	1.85317821	-0.85943685	0.48887746
C	2.88753886	-0.98275091	1.52014500
H	3.57931996	-0.13621405	1.45137339
O	-3.47464390	-0.98001750	0.14686525
O	-1.72854932	-2.45399239	-0.07958831
C	-2.66760470	-3.51138087	0.30342044
H	-2.94308488	-3.39640407	1.35667865
H	-2.11858116	-4.44301969	0.14968606
H	-3.56817045	-3.47635705	-0.31605328
C	-0.61093079	3.61301673	-0.20112690
H	-1.99017695	2.24415613	1.33993281
H	-2.63129151	2.67939589	-0.34379668
H	-1.02171953	4.54846232	0.20246480
H	-0.36957091	3.79196525	-1.26198471
H	0.32593626	3.38895924	0.32720708

TS1-depro-LA1-RA2-Ha

COSMO(Et_2O)-ZORA-BLYP-D3(BJ)/DZP
E = -18105.44
G = -17505.25
COSMO(Et_2O)-ZORA-M06-2X/TZ2P//COSMO(Et_2O)-ZORA-BLYP-D3(BJ)/DZP
E = -25997.80
G = -25397.61
N_{imag} = 1, 1627 *i* cm^{-1}

C	-4.92449829	-2.37021474	4.51885528
N	-4.26859868	-1.38319343	3.66534320
H	-4.34669524	-1.51648542	2.65288536
C	-2.17635152	-4.77262230	5.64357638
S	-3.01138384	-0.24783932	5.76640916
N	-2.73970605	0.17372850	3.06779138
H	-3.09883963	-0.11881309	2.15408479
C	-1.90012830	1.38155105	2.98690140
H	-1.49086444	1.32826387	1.97337137
H	0.47312972	-4.36800782	5.05745185
C	-0.69258407	1.37055773	3.93357674
N	0.27182646	0.30207633	3.56803337
H	-1.03436621	1.26376564	4.97017502
P	1.66750858	0.20606594	4.37461798
C	1.47029445	-0.11443993	6.15377086
C	0.28686593	-0.73806081	6.57504147
C	2.44232291	0.26943717	7.09343952
C	0.06195958	-0.95081670	7.93392105
H	-0.47058394	-1.01888147	5.84944021
C	2.22244670	0.02439697	8.45074523
H	3.35605064	0.76429703	6.76811899
C	1.02919436	-0.57768502	8.87024480
H	-0.86434917	-1.41960305	8.25576181
H	2.97564591	0.31508810	9.18254709
H	0.85745809	-0.75794324	9.93109447
C	2.66551069	1.73373877	4.28799169
C	2.17112800	2.89088628	4.92131790
C	3.85205531	1.80612829	3.54082793
C	2.83765765	4.10796501	4.77399919
H	1.27887396	2.83781975	5.54190196
C	4.52026751	3.02576819	3.40618675
H	4.25901381	0.91510771	3.06971955
C	4.00924596	4.17831792	4.01077451
H	2.44644277	5.00071727	5.26048575
H	5.44242851	3.07409583	2.82792771
H	4.52821317	5.12946892	3.89733811
C	2.63637836	-1.14309358	3.64983379
C	2.91027766	-1.15400766	2.27045408
C	3.10291732	-2.18226853	4.46777266
C	3.64647186	-2.20352488	1.72058761
H	2.51585564	-0.37348481	1.62560542
C	3.83040500	-3.23536411	3.90745284
H	2.88707784	-2.17124707	5.53181521

C	4.10443381	-3.24569664	2.53618201
H	4.18197147	-4.04846810	4.54115491
H	4.67157006	-4.06800245	2.10114074
H	-0.20349572	2.34596907	3.82584631
H	3.85049758	-2.21482776	0.65039783
H	-1.09926868	4.13880427	3.39683579
H	-1.20214352	-7.26974414	5.32052874
H	-1.75921376	-4.93758882	4.64033235
H	-6.71909974	-0.42609325	3.82151087
H	-4.90950909	-1.94523958	5.53015276
H	-4.54735913	1.77631922	2.16087234
C	-2.73901477	2.71296017	2.99826953
C	-3.38843824	2.99455152	4.36662533
C	-1.81774874	3.89331361	2.60297893
C	-3.84127871	2.58125829	1.91915544
H	-4.07059543	2.18757714	4.65857139
H	-2.63264265	3.09516635	5.15758557
H	-3.95991780	3.93435152	4.31680819
C	-3.34408738	-0.46914905	4.09536846
C	-6.41693093	-2.57710227	4.12572338
C	-7.15672227	-1.24822740	4.40419193
C	-6.59116635	-2.96021629	2.63857057
C	-6.99906482	-3.68690339	5.03012305
H	-7.09826883	-0.98188383	5.47006372
H	-8.21701861	-1.34279299	4.12850334
H	-6.03391340	-3.87421038	2.40527351
H	-7.65823684	-3.12608959	2.42746785
H	-6.24724402	-2.15722144	1.97060428
H	-8.08027998	-3.78423656	4.85489821
H	-6.52659871	-4.65574036	4.82473087
H	-6.84845395	-3.44299491	6.09303029
C	-4.06331190	-3.65690245	4.53866565
O	-4.16441844	-4.57942837	3.71940922
N	-3.14847389	-3.67384152	5.55422880
H	-2.95304545	-2.78472220	6.03094974
H	-4.40768367	3.52109255	1.84681344
H	-3.39748678	2.36934447	0.93531425
H	-1.26114355	3.66673125	1.68341458
H	-2.42874494	4.79064348	2.42469735
C	-1.03901441	-4.42816932	6.59796173
C	-1.27739935	-4.33406525	7.97778435
C	-0.22174987	-4.12833599	8.86759526
C	1.08975952	-4.02632567	8.39025421
C	1.33312099	-4.11282431	7.01877823
C	0.27376958	-4.30228787	6.12557116
H	-2.29148714	-4.44981945	8.35800740
H	-0.42153321	-4.05257931	9.93633591
H	1.91464585	-3.86762433	9.08348259
H	2.35129662	-4.03986595	6.64353479
C	-2.83776062	-6.08309527	6.07868804

C	-4.06261553	-6.11134967	6.75221131
C	-4.60599155	-7.32657410	7.18251702
C	-3.92848562	-8.52616776	6.94084173
C	-2.70159429	-8.50219868	6.26502093
C	-2.16093761	-7.28628435	5.83944911
H	-4.59787703	-5.18186010	6.93208494
H	-5.56348805	-7.33565491	7.70331331
H	-4.35350848	-9.47334189	7.27193628
H	-2.16759530	-9.43180189	6.06859208
C	-1.75761457	-0.83016894	0.02164327
C	-0.35452806	-0.47630019	-0.37969872
C	-0.81480496	-1.31603724	0.81962078
H	1.68639252	-3.08759466	-2.75552928
C	0.10145139	0.94236178	-0.08300275
C	0.32580044	-1.12439449	-1.55237399
H	0.23720690	-4.15912406	-2.72882042
C	-0.28075335	-1.87632297	1.98090554
C	-3.13857791	-0.58353426	-0.19232099
O	-0.64790792	1.90723344	-0.03806242
O	1.42538355	1.00152415	0.21793712
C	1.90651354	2.32356688	0.63368509
H	2.98761105	2.21420846	0.73348472
H	1.45723721	2.58593895	1.59632472
H	1.65179320	3.07196533	-0.12302797
O	1.05721500	-0.53006356	-2.33693996
O	0.01238114	-2.44266955	-1.65090285
C	0.59333406	-3.12971867	-2.80566890
H	0.24552654	-2.66198785	-3.73246872
O	-4.03478536	-0.87334838	0.62814338
O	-3.39827455	0.04688599	-1.37531187
C	-4.80077985	0.41066156	-1.57864021
H	-5.42641893	-0.48792818	-1.59238155
H	-4.82191107	0.91758472	-2.54597913
H	-5.13208896	1.07990281	-0.77789387
H	0.75572325	-2.20129106	1.86182850
C	-1.13853934	-2.81000846	2.82970466
H	-0.05285643	-0.71029764	2.77188723
H	-1.37106208	-3.73701150	2.28605947
H	-2.08808595	-2.34763777	3.11219284
H	-0.61441845	-3.08304178	3.75246791

TS1-depro-LA2-RA1-Ha
COSMO(Et_2O)-ZORA-BLYP-D3(BJ)/DZP
E = -18096.02
G = -17494.89
COSMO(Et_2O)-ZORA-M06-2X/TZ2P//COSMO(Et_2O)-ZORA-BLYP-D3(BJ)/DZP
E = -25984.68
G = -25383.55
N_{imag} = 1, 1416 i cm^{-1}

C	-4.74995684	-1.02220424	0.61455250

N	-4.38370914	-0.84182124	2.04060834
H	-3.57129112	-0.21851698	2.10829538
C	-8.08133989	0.89443533	0.63462011
S	-6.52595607	-1.90792822	3.35790986
N	-4.33250311	-0.69177728	4.32558454
H	-3.44142854	-0.22255867	4.14570601
C	-5.01404349	-0.23363955	5.54759776
H	-6.07409958	-0.47869882	5.39644104
H	-8.61327645	0.29002890	-1.96543348
C	-4.96226633	1.31221418	5.59125080
N	-3.64113943	1.93087583	5.28033956
H	-5.34865975	1.64825078	6.56221685
P	-3.72717645	3.47856236	4.77037822
C	-4.20080009	4.65688094	6.08151378
C	-4.30717635	4.17734323	7.39575393
C	-4.36962784	6.02928439	5.82293011
C	-4.61040601	5.05569589	8.43992608
H	-4.12831436	3.12503690	7.60078834
C	-4.67028540	6.90212991	6.86888690
H	-4.24523329	6.41823003	4.81388872
C	-4.79803688	6.41565892	8.17604389
H	-4.69281953	4.67818389	9.45830630
H	-4.79729173	7.96483001	6.66618243
H	-5.03340755	7.10038382	8.99000279
C	-4.91084805	3.67659361	3.39347396
C	-5.99292650	4.57063558	3.45184461
C	-4.70548343	2.91343998	2.22668385
C	-6.81793090	4.74441672	2.33876140
H	-6.20912226	5.11773745	4.36433750
C	-5.52208947	3.10690291	1.11468289
H	-3.91801066	2.16767804	2.18317815
C	-6.57091326	4.03136755	1.16553013
H	-7.66778411	5.42115819	2.40109067
H	-5.35385024	2.51977877	0.21441792
H	-7.21532313	4.16989069	0.29892431
C	-2.07425682	4.02236649	4.22936106
C	-1.49787397	3.48898018	3.06373212
C	-1.33136829	4.90465467	5.03464329
C	-0.18572506	3.81553789	2.72463306
H	-2.03086029	2.77655796	2.44587318
C	-0.02108785	5.23395274	4.68010591
H	-1.75854654	5.31424936	5.94572303
C	0.55549979	4.68588591	3.53015056
H	0.55307466	5.90904991	5.31348309
H	1.58373889	4.93106924	3.26638168
H	-5.68233548	1.65423816	4.83390075
H	0.26247382	3.37257166	1.83653844
H	-4.98283775	0.55852645	8.32962339
H	-10.14064968	2.63204378	0.65323976
H	-7.78852475	1.57592985	-0.17521135

H	-4.62745987	-3.59155460	1.91427806
H	-3.85257091	-0.63906860	0.11821099
H	-4.59396313	-2.91017952	5.73298243
C	-4.58996388	-1.01536604	6.83299500
C	-3.07865007	-0.93569948	7.08690016
C	-5.34432619	-0.44158732	8.05421883
C	-5.01724281	-2.48911041	6.64959201
H	-2.51052719	-1.29657015	6.22313553
H	-2.76900234	0.09303032	7.29327868
H	-2.80327910	-1.55446929	7.95345711
C	-5.01736756	-1.10654231	3.21137635
C	-4.95445862	-2.49224466	0.05612266
C	-4.16193933	-3.48515007	0.92800246
C	-6.43803839	-2.92123036	-0.03247011
C	-4.37622685	-2.49760613	-1.38097411
H	-3.12270607	-3.15591449	1.06815113
H	-4.15073117	-4.47240437	0.44298309
H	-7.00964141	-2.25762715	-0.69558859
H	-6.48757451	-3.93700929	-0.45259479
H	-6.91805012	-2.92170731	0.95095131
H	-4.55284473	-3.47672074	-1.84918544
H	-4.85137878	-1.72289387	-1.99741636
H	-3.29194884	-2.31629888	-1.37257336
C	-5.85129759	-0.05142169	0.12652821
O	-5.73789275	0.51779796	-0.97468882
N	-6.88326265	0.12594725	0.97671572
H	-6.97190072	-0.56003437	1.74820997
H	-4.67329157	-3.08875484	7.50510024
H	-6.11053355	-2.57289411	6.57773906
H	-6.42679074	-0.38089865	7.86331783
H	-5.19041560	-1.09822811	8.92288215
C	-9.12879154	-0.07688969	0.09512199
C	-9.90492782	-0.84451098	0.97350714
C	-10.76785782	-1.82485351	0.47844405
C	-10.86707331	-2.04136304	-0.90184000
C	-10.09973389	-1.27141982	-1.78244202
C	-9.23168995	-0.29399724	-1.28435683
H	-9.82850616	-0.67615953	2.04700856
H	-11.36432221	-2.42125027	1.16880686
H	-11.54060031	-2.80613684	-1.28815452
H	-10.17337355	-1.43494071	-2.85758894
C	-8.57536122	1.71358535	1.82128520
C	-7.97958689	1.64106582	3.08390053
C	-8.46105836	2.41747491	4.14145686
C	-9.54186209	3.28065740	3.94762908
C	-10.14307800	3.35868160	2.68512230
C	-9.66521976	2.57558374	1.63295781
H	-7.12449025	0.99373714	3.24142078
H	-7.97990690	2.35832036	5.11705252
H	-9.91069115	3.89336863	4.76987359

H	-10.98444688	4.03194943	2.52107506
C	-0.72097427	0.04786124	3.20748974
C	0.03503965	-0.85484201	4.14326810
C	-0.71478726	0.45307960	4.46855796
H	-2.44753517	-3.76507312	5.33765098
C	1.54150697	-0.74369825	4.14926203
C	-0.56314506	-2.16952659	4.56608041
H	-3.37861355	-3.59862080	3.80582027
C	-0.96930348	1.27710073	5.57171573
C	-1.11114485	0.13387029	1.84955844
O	2.14387430	0.30873434	4.31174604
O	2.14144438	-1.93396498	3.88267585
C	3.60506745	-1.89328123	3.84000906
H	3.90954123	-2.91930904	3.62265609
H	3.99630070	-1.56539000	4.80868861
H	3.93727311	-1.20884467	3.05264480
O	-0.10207069	-2.89811535	5.43563067
O	-1.71142534	-2.43150990	3.88186355
C	-2.38684757	-3.67379327	4.25015951
H	-1.83479923	-4.52459528	3.83368705
O	-2.05769345	0.82018619	1.41150564
O	-0.35766768	-0.68151315	1.04674552
C	-0.73855813	-0.68421708	-0.36301003
H	-1.74475718	-1.09479390	-0.47892883
H	-0.00447442	-1.32729804	-0.85385121
H	-0.70784692	0.33179738	-0.76993972
C	-0.27678276	0.92344351	6.88428256
H	-2.43746463	1.47508425	5.55329483
H	-0.84972644	2.33080929	5.30882664
H	-0.74513302	1.44925602	7.72910348
H	0.78827569	1.20569563	6.85627620
H	-0.32880974	-0.15382709	7.08695887

TS1-depro-LA2-RA2-Ha
COSMO(Et_2O)-ZORA-BLYP-D3(BJ)/DZP
E = -18095.87
G = -17492.93
COSMO(Et_2O)-ZORA-M06-2X/TZ2P//COSMO(Et_2O)-ZORA-BLYP-D3(BJ)/DZP
E = -25989.54
G = -25386.60
N_{imag} = 1, 1624 i cm^{-1}

C	-4.93037947	-3.07290425	4.90874835
N	-4.05755583	-2.15236406	4.13966050
H	-4.25419733	-2.20393060	3.13591142
C	-7.07054543	-0.36303313	6.61267265
S	-2.88335844	-0.69264936	6.11164978
N	-2.82606761	-0.40539184	3.40237107
H	-3.19347992	-0.76587697	2.51595219
C	-2.14954882	0.89725655	3.25312625
H	-1.80985723	0.87922326	2.21255705

H	-6.30134294	-1.11408857	9.08992910
C	-0.88407971	1.03633570	4.11065972
N	0.16870601	0.08573334	3.66705438
H	-1.14212557	0.88787568	5.16534907
P	1.52779397	0.00595283	4.53407429
C	1.33646091	-0.72622932	6.18950925
C	0.33473476	-1.69500954	6.35330771
C	2.17584606	-0.38845338	7.26457561
C	0.17309369	-2.32739444	7.58689417
H	-0.32875970	-1.92681378	5.52737133
C	2.01304962	-1.02883524	8.49508513
H	2.94911050	0.36781341	7.14045823
C	1.01347653	-1.99795077	8.65536245
H	-0.61778868	-3.06430698	7.71456432
H	2.66204315	-0.76974817	9.33085091
H	0.88658622	-2.49086095	9.61889312
C	2.26586341	1.65192054	4.79882549
C	1.62058176	2.54719012	5.67287902
C	3.37239120	2.08537006	4.05025817
C	2.07111135	3.86348008	5.78209960
H	0.77451764	2.21492807	6.27172669
C	3.82031749	3.40358141	4.16750460
H	3.88758732	1.39629115	3.38535813
C	3.16788070	4.29444305	5.02625616
H	1.56660616	4.55288593	6.45800569
H	4.68238834	3.73400575	3.58897943
H	3.51749973	5.32260370	5.11246561
C	2.71788445	-1.01439256	3.62255665
C	2.89700120	-0.80263822	2.24406690
C	3.48226868	-1.98445047	4.28946301
C	3.83501317	-1.55926889	1.54200808
H	2.28003562	-0.08152977	1.71850021
C	4.41959257	-2.73893244	3.57755916
H	3.34063112	-2.16019450	5.35321943
C	4.59787565	-2.52661070	2.20680339
H	5.00618934	-3.49682384	4.09517542
H	5.32660569	-3.11926272	1.65478643
H	-0.51440215	2.06125751	3.98407646
H	3.96267463	-1.40039788	0.47179186
H	-1.70520515	3.73328874	3.75204430
H	-9.43911737	0.93415893	6.81605212
H	-7.81233715	-1.02568951	7.08633629
H	-5.39617748	-3.49646803	7.72951199
H	-5.32610658	-3.71025094	4.10935484
H	-4.78703662	0.95785124	2.41840225
C	-3.14505088	2.11781623	3.31349707
C	-3.82071107	2.28906091	4.68690769
C	-2.38278140	3.41508112	2.94815267
C	-4.22968773	1.88316218	2.23346829
H	-4.42618669	1.41878119	4.95221260

H	-3.08678799	2.43382302	5.49007613
H	-4.48414340	3.16685542	4.65982189
C	-3.27598713	-1.07852433	4.49284845
C	-4.26719160	-4.08265022	5.94067847
C	-4.35088192	-3.61537026	7.41149278
C	-5.06482543	-5.40592047	5.81709014
C	-2.79918588	-4.33696189	5.54984655
H	-3.82945114	-2.66501768	7.55829491
H	-3.88936512	-4.37975985	8.05473634
H	-4.95976077	-5.83390661	4.80876712
H	-4.68863907	-6.13930488	6.54519418
H	-6.13308815	-5.23743037	6.01090093
H	-2.38128814	-5.12559556	6.19330846
H	-2.71600788	-4.66531958	4.50508883
H	-2.20124955	-3.42881651	5.67642234
C	-6.20326955	-2.39085941	5.47084566
O	-7.31836391	-2.92899376	5.35041704
N	-5.99880528	-1.20256517	6.07568446
H	-5.02546746	-0.89424141	6.23474489
H	-4.94721214	2.71560399	2.23742496
H	-3.77477514	1.81835103	1.23456514
H	-1.80296803	3.28586822	2.02426680
H	-3.10661231	4.22789845	2.78718564
C	-6.42653038	0.51838633	7.68352427
C	-6.08973337	1.85346092	7.43733230
C	-5.38839160	2.59372679	8.39495564
C	-5.02236385	2.00500503	9.60866796
C	-5.36268271	0.66995459	9.86289654
C	-6.05813501	-0.06808393	8.90347620
H	-6.36416148	2.31458784	6.49233373
H	-5.12332481	3.63026695	8.18771823
H	-4.47515372	2.58111683	10.35462509
H	-5.08125788	0.20430928	10.80728150
C	-7.78051970	0.43856001	5.52588183
C	-7.21816743	0.61588169	4.25850830
C	-7.85666320	1.41394987	3.30391898
C	-9.06961217	2.03902705	3.60874998
C	-9.64204100	1.85859870	4.87496867
C	-8.99941410	1.06271256	5.82620859
H	-6.27555452	0.13226303	4.02038973
H	-7.40385004	1.55003961	2.32310686
H	-9.56866376	2.66064190	2.86575435
H	-10.58905472	2.33923160	5.12002334
C	-1.98682247	-1.06156291	0.20756751
C	-0.69298684	-0.50676255	-0.30836595
C	-0.90839581	-1.46644761	0.86852622
H	0.47369804	-0.98605842	-4.18060270
C	-0.36979962	0.93391464	0.04599030
C	-0.08477168	-1.08299903	-1.55964092
H	1.74313666	0.26352299	-3.90244680

C	-0.18145761	-2.01857607	1.92461948
C	-3.40426418	-1.00364326	0.14546086
O	-1.19052606	1.83955714	0.08239754
O	0.93260151	1.07994395	0.41378962
C	1.29388963	2.40328079	0.93009130
H	2.37975754	2.37912678	1.04532866
H	0.81092426	2.55428215	1.90016220
H	0.98842303	3.18299107	0.22577839
O	-0.20152051	-2.24821333	-1.91178570
O	0.60335320	-0.13743247	-2.25683766
C	1.23669611	-0.61180831	-3.49066739
H	1.95216691	-1.40733094	-3.25861359
O	-4.15969963	-1.46814808	1.02548331
O	-3.86969556	-0.35074957	-0.95898821
C	-5.32298653	-0.19401291	-1.01427113
H	-5.80824470	-1.17514898	-1.04829658
H	-5.51029601	0.36714224	-1.93262890
H	-5.67149919	0.36228647	-0.13840525
H	0.87515163	-2.17468728	1.68567293
C	-0.80900680	-3.13480699	2.75335002
H	-0.06488564	-0.89246543	2.79709748
H	-0.24011688	-3.28678170	3.68075507
H	-0.81836334	-4.09177070	2.20684894
H	-1.83879171	-2.89548937	3.03147898

TS1-depro-LA1-RA1-Hb
COSMO(Et_2O)-ZORA-BLYP-D3(BJ)/DZP
E = -18107.49
G = -17506.18
COSMO(Et_2O)-ZORA-M06-2X/TZ2P//COSMO(Et_2O)-ZORA-BLYP-D3(BJ)/DZP
E = -25998.51
G = -25397.20
N_{imag} = 1, 1680 i cm^{-1}

C	-6.11807436	-2.31349277	2.14153568
N	-4.93066190	-1.52811439	2.51820815
H	-4.07103809	-1.77518058	2.01249323
C	-5.20355367	-5.35297275	4.25978477
S	-5.95947357	-0.99664378	4.97101685
N	-3.42761073	-0.73167057	4.05197433
H	-2.74716275	-0.93550089	3.31236691
C	-2.97406876	-0.01085620	5.23717520
H	-3.89053089	0.21456263	5.79532258
H	-3.27164502	-4.16288189	2.71773482
C	-2.28070504	1.34020901	4.84240424
N	-2.66287194	1.84779269	3.52049539
H	-1.19506404	1.18858019	4.79694756
P	-3.84777316	2.90514339	3.26953226
C	-3.41631844	4.63623860	3.66854998
C	-2.07542626	5.02475210	3.51285370
C	-4.37765606	5.57943047	4.06696049

C	-1.69950449	6.34832320	3.75024429
H	-1.33389898	4.28950350	3.21125845
C	-3.99630953	6.90358729	4.30124375
H	-5.41616166	5.28166795	4.19662805
C	-2.66007295	7.28828389	4.14343413
H	-0.65856692	6.64657943	3.63074304
H	-4.74251168	7.63455717	4.61073917
H	-2.36606761	8.32081528	4.32928235
C	-5.39453684	2.59277154	4.17443813
C	-5.41665605	2.75238611	5.57183047
C	-6.55321856	2.18186273	3.49967562
C	-6.59068519	2.50330956	6.28020852
H	-4.52618083	3.08463086	6.10283303
C	-7.72726650	1.94312846	4.21529783
H	-6.54210780	2.04998700	2.42240266
C	-7.74876405	2.10494076	5.60216842
H	-6.60435239	2.62340381	7.36298427
H	-8.62295497	1.61968133	3.68714538
H	-8.66504158	1.91172849	6.15924518
C	-4.24618026	2.84190204	1.50289655
C	-4.05329483	1.64347868	0.79845875
C	-4.87770980	3.93347619	0.88430337
C	-4.50477542	1.53657684	-0.51634426
H	-3.57148832	0.80434938	1.28925327
C	-5.34227732	3.81008153	-0.42634865
H	-5.01456601	4.86841624	1.42421933
C	-5.16170844	2.60968123	-1.12272640
H	-5.84056591	4.65250666	-0.90509988
H	-5.52332370	2.51512166	-2.14630739
H	-2.45069897	2.06777681	5.64843409
H	-4.34047298	0.61701764	-1.06958137
H	-1.05737305	0.75601176	7.18368217
H	-6.50609778	-7.69854591	4.79396600
H	-5.35989011	-6.11299587	3.48041251
H	-6.32645513	0.19362783	1.17227112
H	-6.88873445	-2.03691864	2.87264267
H	-3.13893739	-2.76534585	5.68295328
C	-2.06824581	-0.90992979	6.14567642
C	-0.79016459	-1.35716906	5.39969239
C	-1.68913654	-0.11309733	7.41418899
C	-2.86591988	-2.16733598	6.55824927
H	-1.03635414	-1.87095632	4.46247743
H	-0.12240040	-0.51482780	5.17330210
H	-0.22811123	-2.06829465	6.02186966
C	-4.71104647	-1.08341674	3.80183415
C	-6.65748786	-1.92323573	0.73807236
C	-7.13737816	-0.45623609	0.83187060
C	-5.58085261	-2.05277701	-0.36043312
C	-7.86378553	-2.82798176	0.40225400
H	-7.97133423	-0.36533780	1.54316789

H	-7.47394843	-0.09911078	-0.15205891
H	-5.28031433	-3.09854058	-0.48235616
H	-5.97659184	-1.68403262	-1.31825940
H	-4.69013087	-1.45790378	-0.12337808
H	-8.33134492	-2.49257552	-0.53510198
H	-7.55356298	-3.87312459	0.28514531
H	-8.62275013	-2.77387023	1.19803341
C	-5.76959834	-3.80763007	2.37410986
O	-5.48047042	-4.61794840	1.47870529
N	-5.73389396	-4.09800404	3.70332379
H	-5.89507045	-3.31752840	4.35471246
H	-2.25564500	-2.79964442	7.21860046
H	-3.78963228	-1.88921693	7.08284657
H	-2.59149426	0.24299414	7.93481731
H	-1.12947809	-0.75808769	8.10766428
C	-3.69286832	-5.25976256	4.53507144
C	-3.11894798	-5.82297131	5.68321986
C	-1.74010444	-5.73233486	5.90715806
C	-0.91796046	-5.08016925	4.98532339
C	-1.48364842	-4.52317601	3.83387371
C	-2.85741162	-4.61579245	3.60994194
H	-3.74744677	-6.32303515	6.41662276
H	-1.31349477	-6.16342595	6.81307898
H	0.15300397	-4.99474497	5.16790276
H	-0.86483113	-3.99278597	3.11264803
C	-6.00523531	-5.72278591	5.49621765
C	-6.12343669	-4.81044307	6.55702796
C	-6.83591747	-5.15192798	7.70809442
C	-7.43130163	-6.41548718	7.81690409
C	-7.31083142	-7.33068666	6.76693259
C	-6.60094253	-6.98286619	5.61065946
H	-5.64835433	-3.83228252	6.48987307
H	-6.92342151	-4.43456991	8.52390678
H	-7.98464568	-6.68370030	8.71659520
H	-7.77003296	-8.31596569	6.84491958
C	-1.17231902	-0.46913347	0.44569658
C	-0.46972076	0.51259015	-0.44138788
C	-0.68007684	0.59172469	1.06915907
H	2.75512990	-0.45607585	-2.71806022
C	-1.27761667	1.40177214	-1.36660229
C	0.94113560	0.20804472	-0.88689279
H	2.64580758	1.16625886	-3.49722736
C	-0.35965607	1.37242773	2.18784730
C	-1.76033114	-1.74463408	0.62988046
O	-2.02371077	1.00627515	-2.24956540
O	-1.06590683	2.71105579	-1.06635913
C	-1.76908576	3.67482917	-1.91590741
H	-1.46789057	3.53835519	-2.96007713
H	-1.46244681	4.65523338	-1.54439399
H	-2.84702340	3.53852350	-1.81267154

O	1.71456711	-0.51231330	-0.27296346
O	1.24187052	0.84714607	-2.04948120
C	2.59793910	0.61479531	-2.55607079
H	3.33148458	0.99686253	-1.83869476
O	-2.22854530	-2.12162659	1.72517940
O	-1.78692080	-2.52416019	-0.48592110
C	-2.30617979	-3.88070365	-0.27094728
H	-1.68718591	-4.40061028	0.46749239
H	-2.23681475	-4.36368528	-1.24825707
H	-3.34147025	-3.84689089	0.07686336
H	-0.10219953	0.74239869	3.05141444
H	-1.61337789	1.74015050	2.71270245
C	0.55925116	2.57554446	1.99228846
H	1.58454143	2.27543292	1.71940607
H	0.62439465	3.16543900	2.91751900
H	0.17485987	3.22450577	1.19436436

TS1-depro-LA1-RA2-Hb
COSMO(Et_2O)-ZORA-BLYP-D3(BJ)/DZP
E = -18104.39
G = -17501.96
COSMO(Et_2O)-ZORA-M06-2X/TZ2P//COSMO(Et_2O)-ZORA-BLYP-D3(BJ)/DZP
E = -25995.55
G = -25393.12
N_{imag} = 1, 1408 i cm^{-1}

C	-4.82508125	-2.25784509	4.40341937
N	-4.31275252	-1.11111080	3.65554387
H	-4.37617912	-1.16300752	2.63434118
C	-1.80428577	-4.43832519	5.25662758
S	-3.11053748	-0.10475912	5.85370680
N	-2.92139406	0.61701535	3.21146159
H	-3.33301303	0.45720276	2.28411508
C	-1.89257965	1.67458241	3.19864784
H	-1.47029150	1.60020533	2.18701399
H	0.88554576	-3.97981021	5.10257526
C	-0.72854505	1.46014403	4.17427285
N	0.14541703	0.32124568	3.78250248
H	-1.11140975	1.33420687	5.19239231
P	1.55519622	0.17512033	4.56491120
C	1.42918396	-0.23442462	6.33247626
C	0.19976418	-0.71148541	6.80851375
C	2.50040627	-0.02858603	7.21883389
C	0.03004676	-0.94850391	8.17209313
H	-0.62975275	-0.86043264	6.12263271
C	2.33288197	-0.30243699	8.57755543
H	3.45154532	0.35608857	6.85263054
C	1.09429011	-0.74948985	9.05454268
H	-0.92997021	-1.30376698	8.53981795
H	3.16335709	-0.15251792	9.26673542
H	0.96426923	-0.94996217	10.11762715

C	2.48884039	1.74513239	4.52088436
C	2.12039607	2.77451940	5.40765678
C	3.43854905	2.00044812	3.51766474
C	2.68292655	4.04550408	5.27676563
H	1.40024952	2.58087425	6.20063863
C	4.00128348	3.27293104	3.39528018
H	3.74859319	1.20807962	2.84180587
C	3.61881902	4.29805520	4.26752617
H	2.38930484	4.83922031	5.96275804
H	4.74148567	3.46333090	2.61884956
H	4.05514449	5.29112799	4.16577075
C	2.52907889	-1.09800351	3.72925487
C	2.62455796	-1.05895878	2.33034535
C	3.19430613	-2.09955236	4.45201945
C	3.37731573	-2.01597643	1.65272457
H	2.07562228	-0.31361136	1.77112872
C	3.93727369	-3.06441580	3.76788332
H	3.11471446	-2.13464398	5.53437225
C	4.03185726	-3.02215017	2.37132522
H	4.43931598	-3.85464577	4.32486454
H	4.60877905	-3.78100225	1.84355606
H	-0.13632672	2.38157605	4.13993479
H	3.41975269	-1.98937866	0.56443318
H	-0.66081135	4.25137776	3.62479632
H	-0.55691260	-6.62051017	4.26129057
H	-1.27629859	-4.30678746	4.30464645
H	-6.88367319	-0.51470246	3.99393013
H	-4.84499383	-1.94797226	5.45510884
H	-4.51152740	2.50684661	2.56170982
C	-2.52757688	3.11079044	3.29676859
C	-3.05249166	3.40752195	4.71592280
C	-1.46819910	4.16149726	2.88582693
C	-3.69949694	3.19358453	2.29013481
H	-3.78717195	2.65396906	5.02714854
H	-2.23973124	3.40756213	5.45531048
H	-3.53217415	4.39807751	4.73811259
C	-3.44060342	-0.18185609	4.16660798
C	-6.28435333	-2.62295021	4.00283450
C	-7.19378933	-1.45986767	4.46062578
C	-6.44682588	-2.83523154	2.48090008
C	-6.67577781	-3.91144903	4.76174191
H	-7.15009627	-1.33425549	5.55287837
H	-8.23707162	-1.66195001	4.17757593
H	-5.76668255	-3.61592615	2.12257925
H	-7.48347409	-3.13267407	2.26284199
H	-6.24756093	-1.90945401	1.92159154
H	-7.73879800	-4.13749175	4.59412581
H	-6.08193859	-4.76951078	4.42145322
H	-6.52182873	-3.78855521	5.84488386
C	-3.80224588	-3.41117113	4.26272805

O	-3.75577543	-4.18427899	3.29805671
N	-2.92043441	-3.48160334	5.30779991
H	-2.83501962	-2.64695382	5.90105247
H	-4.10629647	4.21520046	2.27507731
H	-3.36273576	2.94157615	1.27346591
H	-1.01935329	3.90424234	1.91506502
H	-1.94354320	5.14928783	2.79532889
C	-0.82268538	-4.20805151	6.39874631
C	-1.24969099	-4.28020369	7.73316107
C	-0.32697737	-4.19431309	8.77700182
C	1.03649879	-4.04053219	8.50053365
C	1.46687001	-3.95784315	7.17576832
C	0.54146228	-4.03885198	6.13144137
H	-2.30586430	-4.42556384	7.95552355
H	-0.67159949	-4.24874206	9.80946551
H	1.75593942	-3.96872542	9.31496013
H	2.52479303	-3.82776455	6.95542272
C	-2.29799918	-5.88625505	5.30340270
C	-3.49549415	-6.23983091	5.93328296
C	-3.88421815	-7.58054149	6.01120892
C	-3.07697287	-8.58173313	5.45938617
C	-1.87777126	-8.23124966	4.82709087
C	-1.49217495	-6.88977914	4.75238833
H	-4.13376072	-5.46327357	6.34840416
H	-4.82289055	-7.84356898	6.49884799
H	-3.38191986	-9.62631493	5.51613704
H	-1.24507617	-9.00259749	4.38836319
C	-2.05736643	-0.72459918	0.29964112
C	-0.62015029	-0.88280517	-0.10114572
C	-1.32627489	-1.29421647	1.23339493
H	0.22953952	-4.63243250	-1.28645063
C	0.17281334	0.40817206	-0.05956472
C	-0.16588044	-2.02029636	-0.97657619
H	-1.52904624	-4.92347528	-1.55923991
C	-0.95496575	-1.83938022	2.46475136
C	-3.30393678	-0.17021154	-0.08216224
O	-0.34060205	1.48728102	0.20914417
O	1.50183721	0.25562800	-0.29749391
C	2.28648531	1.49092562	-0.21173344
H	3.32499130	1.16846259	-0.31812382
H	2.11994214	1.97737369	0.75479747
H	2.00251379	2.17069422	-1.02214034
O	0.89043264	-2.08552616	-1.59291921
O	-1.09160423	-3.02548895	-0.96398679
C	-0.70057798	-4.22426218	-1.70023598
H	-0.56085932	-3.99025716	-2.76224510
O	-4.27413016	-0.04571340	0.69321634
O	-3.32738433	0.24063286	-1.38386383
C	-4.56942037	0.88359961	-1.80958348
H	-5.40665027	0.18323254	-1.72354370

H	-4.39835316	1.16077480	-2.85241404
H	-4.76111162	1.77047585	-1.19647857
C	0.05588181	-2.98636251	2.42608029
H	-1.81808440	-2.04063465	3.10655440
H	-0.36771031	-0.69814753	3.13966420
H	0.62538967	-3.05336539	3.35849633
H	0.77810328	-2.85286397	1.61433491
H	-0.45812383	-3.94610949	2.25993974

TS1-depro-LA2-RA2-Hb
COSMO(Et_2O)-ZORA-BLYP-D3(BJ)/DZP
E = -18093.14
G = -17489.97
COSMO(Et_2O)-ZORA-M06-2X/TZ2P//COSMO(Et_2O)-ZORA-BLYP-D3(BJ)/DZP
E = -25987.45
G = -25384.28
N_{imag} = 1, 1738 i cm^{-1}

C	-5.84728316	0.93137987	2.20026931
N	-4.54819893	1.08507876	2.89396477
H	-4.36991270	2.06705018	3.13940488
C	-7.75180003	-1.22375918	4.74765124
S	-3.77287696	-1.49229890	3.29881727
N	-2.80679807	0.86092408	4.30847484
H	-2.99130056	1.86572928	4.38476039
C	-1.69263572	0.43144992	5.17733244
H	-1.02889232	1.30848965	5.18066940
H	-10.06669772	-1.88669739	3.46497343
C	-0.87165855	-0.73513757	4.61304217
N	-0.06877257	-0.32442031	3.43464552
H	-1.53852199	-1.56186247	4.35825202
P	1.40043768	-0.95115798	3.25517279
C	1.44277906	-2.68616340	2.69147800
C	0.21526913	-3.34600570	2.52315416
C	2.64619257	-3.35577551	2.40661712
C	0.19402698	-4.67494879	2.08822576
H	-0.71526741	-2.81323482	2.70974110
C	2.61775270	-4.68164410	1.97178340
H	3.59997380	-2.84002139	2.50855764
C	1.39248364	-5.34271052	1.81703599
H	-0.75935423	-5.18491737	1.95492792
H	3.55020104	-5.19826087	1.74717573
H	1.37380940	-6.37763144	1.47678011
C	2.30750986	-0.88207610	4.84846709
C	2.98027473	-1.98269375	5.40209277
C	2.23048173	0.32246991	5.57455349
C	3.59784952	-1.86805569	6.65187352
H	3.00710188	-2.93456983	4.87942872
C	2.85418805	0.43037921	6.81680500
H	1.65437507	1.15962680	5.19113814
C	3.54408657	-0.66133775	7.35616630

H	4.11509589	-2.72760973	7.07692360
H	2.78499638	1.36433802	7.37345936
H	4.02553551	-0.57654359	8.32991755
C	2.30051646	0.00644361	1.99842117
C	2.67246584	1.32850967	2.29217200
C	2.54983972	-0.51905793	0.72123261
C	3.26713264	2.12528142	1.31630604
H	2.47171621	1.73684088	3.27355138
C	3.16490493	0.27706923	-0.24848100
H	2.25035571	-1.53456783	0.47460235
C	3.51774550	1.59752996	0.04495988
H	3.35085723	-0.13186909	-1.24099415
H	3.97580463	2.22222248	-0.72120846
H	-0.20571865	-1.07523253	5.41508202
H	3.49784481	3.16434860	1.53704374
H	-0.22258381	-0.59199255	7.38410416
H	-8.20266500	-3.38139466	6.41776565
H	-8.64439097	-0.58189009	4.73418703
H	-7.35666580	-1.35190297	1.27129673
H	-6.10082732	1.97623793	1.98255855
H	-4.02254220	1.36673499	6.52590455
C	-2.15403771	0.21713584	6.66694714
C	-2.91583768	-1.11139509	6.85272853
C	-0.90366434	0.24627530	7.57834988
C	-3.07305609	1.39308694	7.07381749
H	-3.77951829	-1.17107285	6.18163793
H	-2.26841690	-1.97633673	6.65147543
H	-3.28262904	-1.19204683	7.88625088
C	-3.70440517	0.20883291	3.53032451
C	-5.87111017	0.19944190	0.78636213
C	-6.33875617	-1.27211028	0.86608223
C	-6.87860444	0.97844029	-0.09563386
C	-4.47291019	0.27831199	0.14338242
H	-5.67052306	-1.86683657	1.49543930
H	-6.34722963	-1.69992567	-0.14803009
H	-6.53868740	2.01300657	-0.25691727
H	-6.97196549	0.48884280	-1.07602962
H	-7.86906762	1.00894197	0.37742843
H	-4.53135422	-0.06910786	-0.89914298
H	-4.09388834	1.31005116	0.14114295
H	-3.75874035	-0.35411924	0.68354043
C	-7.01953032	0.46237735	3.09293140
O	-8.15682456	0.94075529	2.93061486
N	-6.71742739	-0.48495368	4.00992906
H	-5.80222506	-0.95536040	3.91459073
H	-3.29802909	1.33606205	8.14821522
H	-2.58949121	2.36224028	6.87621578
H	-0.33806381	1.17922521	7.43868107
H	-1.21239319	0.18700508	8.63255789
C	-8.06789119	-2.50250094	3.98354966

C	-7.08577744	-3.49065395	3.82041512
C	-7.33856913	-4.61566322	3.03568342
C	-8.58020960	-4.76748649	2.40398605
C	-9.56469299	-3.78875763	2.56676182
C	-9.30619929	-2.65933391	3.35306791
H	-6.11452186	-3.37032869	4.29863917
H	-6.56608135	-5.37429512	2.90989553
H	-8.77637197	-5.64445195	1.78715414
H	-10.53181962	-3.89869815	2.07622590
C	-7.31484405	-1.42516296	6.19738543
C	-6.59639580	-0.41170071	6.84992613
C	-6.21249925	-0.55581368	8.18396492
C	-6.53670843	-1.72122843	8.88685044
C	-7.25641343	-2.73456272	8.24552289
C	-7.64689339	-2.58544898	6.90955585
H	-6.32431329	0.48538120	6.29783239
H	-5.64544841	0.23622035	8.67037281
H	-6.22750270	-1.84084162	9.92493652
H	-7.51436683	-3.64691056	8.78335691
C	-1.60714129	3.69375012	2.96428460
C	-0.19110167	3.87550673	2.49853799
C	-1.15875426	2.68570602	2.23921485
H	-0.48793085	5.41064504	-1.20389281
C	0.83773095	3.70375826	3.58542684
C	0.14799580	4.69675349	1.27922538
H	-1.69629800	6.60873593	-0.60895499
C	-1.15171195	1.42867581	1.62763562
C	-2.60480993	4.25834512	3.79394864
O	0.65097888	3.01199803	4.58103203
O	1.96306955	4.43879239	3.39034680
C	2.94426236	4.36804307	4.47588770
H	3.74766391	5.04250787	4.17152814
H	3.31922244	3.34536635	4.58271360
H	2.48807736	4.69329333	5.41647426
O	1.22493005	4.68683929	0.69964954
O	-0.93639910	5.40569284	0.85308975
C	-0.74019213	6.12021921	-0.40855694
H	0.06236553	6.85756228	-0.30238195
O	-3.67339806	3.68860057	4.10656605
O	-2.26807827	5.49895308	4.25545795
C	-3.24594516	6.11022020	5.15462569
H	-4.19708605	6.25891304	4.63241452
H	-2.80452171	7.06769912	5.44033209
H	-3.40014810	5.47337777	6.03204924
C	-0.33274934	1.23495374	0.35179469
H	-2.15535962	0.99628881	1.57451108
H	-0.57917928	0.57854672	2.58648272
H	-0.03999914	0.18420464	0.23446448
H	0.57877335	1.84320562	0.36510640
H	-0.91877982	1.51869895	-0.53739420

TS2-repro-LA1-RA1-R (TS2-R)
COSMO(Et_2O)-ZORA-BLYP-D3(BJ)/DZP
E = -18114.4
G = -17511.94
COSMO(Et_2O)-ZORA-M06-2X/TZ2P//COSMO(Et_2O)-ZORA-BLYP-D3(BJ)/DZP
E = -26011.26
G = -25408.80
N_{imag} = 1, 1436 i cm^{-1}

C	-6.04006263	-2.26943344	2.21916818
N	-4.71659815	-1.66673329	2.39309618
H	-4.10291439	-1.66125670	1.56669533
C	-5.29859469	-5.72350487	3.63870129
S	-4.97353342	-1.51893784	5.08967625
N	-2.85672387	-0.93342020	3.48851332
H	-2.63668470	-0.63421171	2.53907474
C	-2.14458078	-0.10414042	4.47296987
H	-2.87208409	0.14750130	5.25615740
H	-3.85687338	-7.21621085	1.86105702
C	-1.64615638	1.21510895	3.76529072
N	-2.16781071	1.39110152	2.39415328
H	-0.55867238	1.19383305	3.66914176
P	-3.06884198	2.66074282	1.97700475
C	-2.29537184	4.23863270	2.44444246
C	-0.90072751	4.33762838	2.28366281
C	-3.03043175	5.32325495	2.94603572
C	-0.25381550	5.53114231	2.60784475
H	-0.33141261	3.47968899	1.92938135
C	-2.37280204	6.51441376	3.26774464
H	-4.10547160	5.23868942	3.08934774
C	-0.98833624	6.61973997	3.09559545
H	0.82580629	5.61147844	2.48646931
H	-2.94119798	7.35743688	3.65887224
H	-0.47893154	7.54890617	3.34933549
C	-4.76799870	2.66754226	2.64450033
C	-5.06637320	1.96821357	3.82259060
C	-5.78990132	3.34386117	1.95429935
C	-6.37304097	1.95124399	4.31275064
H	-4.30086951	1.41048984	4.34804480
C	-7.09211488	3.33252135	2.45587328
H	-5.57351686	3.85841396	1.01973645
C	-7.38367397	2.63931981	3.63615413
H	-6.59676350	1.37600445	5.20951580
H	-7.88331560	3.85110474	1.91526578
H	-8.40498155	2.61862544	4.01560215
C	-3.29327860	2.66120022	0.17896341
C	-3.85585616	1.52535470	-0.42308771
C	-3.02286283	3.80726020	-0.58314752
C	-4.13701378	1.53370733	-1.78811349
H	-4.04586393	0.63338678	0.16139735

C	-3.31949345	3.81231102	-1.94905017
H	-2.58838533	4.68853830	-0.11625944
C	-3.87689512	2.67897514	-2.55054220
H	-3.11032140	4.70092931	-2.54355851
H	-4.10342340	2.68536320	-3.61654880
H	-1.89656964	2.07204291	4.40437387
H	-4.55747285	0.64473860	-2.25593750
H	0.23066926	0.92038516	5.65227832
H	-4.32483146	-7.74839252	5.20645045
H	-5.74652333	-6.29827159	2.81419075
H	-6.08217996	0.38136077	1.49677737
H	-6.59007958	-2.03427281	3.13975887
H	-2.08674767	-2.73752603	5.27484877
C	-0.96817232	-0.87121884	5.16413012
C	0.04302788	-1.39367147	4.12098506
C	-0.26916323	0.08399687	6.15909623
C	-1.54708369	-2.06560779	5.94796293
H	-0.44777242	-2.03970134	3.38516991
H	0.53566615	-0.57956861	3.57493567
H	0.82373819	-1.98222479	4.62554986
C	-4.15195095	-1.36150212	3.59548252
C	-6.83391342	-1.63398656	1.04086120
C	-7.03846892	-0.13826901	1.37079399
C	-6.11429790	-1.76749642	-0.31980032
C	-8.21143412	-2.33195460	0.97409418
H	-7.60808377	-0.01373312	2.30172427
H	-7.59049946	0.35559689	0.55843239
H	-5.97101791	-2.82160697	-0.58182019
H	-6.72031201	-1.27997393	-1.09808085
H	-5.13266425	-1.28187009	-0.31151874
H	-8.84285111	-1.83883803	0.22079535
H	-8.10607344	-3.38920655	0.69946517
H	-8.72889619	-2.27059790	1.94384854
C	-5.84762536	-3.80901693	2.17361907
O	-5.78682433	-4.48728512	1.13879511
N	-5.71620546	-4.33308455	3.42623628
H	-5.60116500	-3.66550662	4.20299093
H	-0.73366977	-2.62844586	6.42993297
H	-2.25189031	-1.72988967	6.72053607
H	-0.99232827	0.49737816	6.87943451
H	0.49587567	-0.46701040	6.72566433
C	-3.78460152	-5.87672281	3.54704380
C	-2.95190149	-5.21101415	4.45690369
C	-1.56887768	-5.39134546	4.41165692
C	-1.00288741	-6.24655948	3.45807044
C	-1.82662452	-6.90024519	2.53758321
C	-3.21305706	-6.70976159	2.58009481
H	-3.39139890	-4.55443379	5.20676659
H	-0.92980542	-4.86468192	5.11766469
H	0.07716084	-6.39006571	3.42606703

H	-1.39167875	-7.55335176	1.78091678
C	-5.86655825	-6.25553741	4.95634654
C	-7.04226087	-5.72121473	5.50365508
C	-7.57750462	-6.24014996	6.68756600
C	-6.94970660	-7.30861569	7.33589536
C	-5.78107927	-7.85267349	6.79094345
C	-5.24261822	-7.32777453	5.61249771
H	-7.54197852	-4.89434013	5.00299987
H	-8.48780727	-5.80716842	7.10208397
H	-7.36499925	-7.71198568	8.25904687
H	-5.28145968	-8.68413365	7.28795114
C	-0.82357283	-0.09510586	0.56378890
C	0.70812881	-0.00903988	0.34641347
C	-0.32449705	0.64689683	-0.53906943
H	3.66955632	1.66418457	2.56659437
C	1.40691749	-1.25743626	-0.11038422
C	1.50045377	0.93236028	1.21143456
H	4.60560724	1.90578861	1.04551307
C	-0.39909601	1.47683859	-1.57086809
C	-1.60268623	-1.32457415	0.52307203
O	2.29874634	-1.81593099	0.51448612
O	0.87388249	-1.73409103	-1.26907196
C	1.31008262	-3.08030262	-1.63236378
H	0.76990460	-3.31482504	-2.55241448
H	1.04391322	-3.77985115	-0.83277598
H	2.39236675	-3.09810646	-1.79792082
O	1.05730308	1.59827965	2.13732140
O	2.78214205	1.03543064	0.76422448
C	3.62193560	1.96855585	1.51580119
H	3.21400171	2.98200923	1.44146575
O	-2.80020164	-1.36067139	0.18906828
O	-0.96139834	-2.41274270	1.03295595
C	-1.78530499	-3.61564167	1.18161498
H	-2.45953171	-3.50047272	2.03619034
H	-1.07684879	-4.42055652	1.37575637
H	-2.36250897	-3.80071196	0.27126505
C	0.80482184	2.05035773	-2.26722790
H	-1.46912724	0.76375348	1.52953102
H	-1.38289770	1.76265852	-1.94041458
H	0.82100232	3.14954601	-2.17937829
H	0.79391486	1.81659264	-3.34380055
H	1.73507425	1.65756034	-1.83688561

TS2-repro-LA1-RA2-R
COSMO(Et_2O)-ZORA-BLYP-D3(BJ)/DZP
E = -18099.17
G = -17497.66
COSMO(Et_2O)-ZORA-M06-2X/TZ2P//COSMO(Et_2O)-ZORA-BLYP-D3(BJ)/DZP
E = -25994.70
G = -25393.19

N_{imag} = 1, 1560 i cm^{-1}

C	-4.81136140	-1.76481305	4.79007141
N	-4.27332592	-0.69922939	3.95211852
H	-4.45472664	-0.74542932	2.94463729
C	-2.16779133	-4.47530885	4.31110883
S	-2.53911730	0.10610833	5.85663434
N	-3.24862965	1.26256214	3.47111792
H	-3.84452619	1.13853530	2.65202142
C	-2.15191678	2.22981465	3.23268244
H	-2.23612708	2.44011095	2.15792305
H	0.10680874	-4.57467115	2.82158695
C	-0.76103217	1.60123036	3.43293115
N	-0.11149653	1.23730317	2.14397379
H	-0.85414203	0.73078868	4.08764983
P	1.50244695	1.34665203	2.15897476
C	2.03249821	3.04864884	2.57505978
C	2.14300888	3.43839829	3.92309896
C	2.14418342	4.01830326	1.56354663
C	2.33221156	4.78251273	4.25153567
H	2.08525645	2.69472229	4.71505289
C	2.34087534	5.35918305	1.89873582
H	2.07793161	3.72996084	0.51710700
C	2.42543423	5.74465223	3.24152128
H	2.40504612	5.07672488	5.29786053
H	2.42820951	6.10481670	1.10915137
H	2.57019765	6.79328321	3.49911970
C	2.15513265	0.96138809	0.51853133
C	1.47019509	1.42342114	-0.61673993
C	3.37726754	0.28432398	0.37613653
C	2.02109084	1.22493184	-1.88329851
H	0.50848684	1.91891679	-0.50479376
C	3.90972032	0.07323635	-0.89604323
H	3.90886202	-0.08309039	1.24957432
C	3.23612931	0.55075575	-2.02606140
H	1.49206988	1.58547510	-2.76359032
H	4.85124596	-0.46371860	-1.00469270
H	3.65588691	0.39001567	-3.01850839
C	2.28559216	0.30581205	3.43491059
C	1.48536057	-0.66856785	4.05424040
C	3.61202305	0.49535139	3.85845056
C	2.00068966	-1.43536399	5.09705769
H	0.47177369	-0.83816301	3.70597497
C	4.13437266	-0.30191471	4.87977205
H	4.23014935	1.26728976	3.40220190
C	3.32659853	-1.26159808	5.50367518
H	5.16670343	-0.16389700	5.19936538
H	3.73161802	-1.87089849	6.31098099
H	-0.13516903	2.31504818	3.97638240
H	1.36891142	-2.17241092	5.58180123
H	-0.48196625	4.35592960	2.99823601

H	-1.79517003	-6.91306759	3.22133453
H	-2.17436022	-4.47030442	3.21119454
H	-6.39939632	0.46616665	4.92961904
H	-4.43858002	-1.55987979	5.80240729
H	-4.44193895	3.32099321	4.48932472
C	-2.34584357	3.62665343	3.92605908
C	-1.86145949	3.65612749	5.39324627
C	-1.53395761	4.65202153	3.09671130
C	-3.84084182	4.00692064	3.87792426
H	-2.23111429	2.78402458	5.94572849
H	-0.76435026	3.66300259	5.45819128
H	-2.22582934	4.57218129	5.88182508
C	-3.37655097	0.24442426	4.36562764
C	-6.37025633	-1.72758488	4.85430018
C	-6.76930835	-0.38728133	5.51429881
C	-7.01387123	-1.82070443	3.45310634
C	-6.84995776	-2.89449096	5.74694805
H	-6.35564673	-0.31067415	6.53074993
H	-7.86451265	-0.31257010	5.57964565
H	-6.76064742	-2.77117016	2.97187066
H	-8.10749166	-1.74048753	3.54347516
H	-6.67716905	-0.99811897	2.80528327
H	-7.94079106	-2.84071107	5.87798929
H	-6.59857151	-3.86405959	5.30169037
H	-6.38678058	-2.83524322	6.74423425
C	-4.19889361	-3.10386673	4.32835091
O	-4.79279178	-3.93231708	3.62928503
N	-2.92370443	-3.30360523	4.77243949
H	-2.44827051	-2.54401700	5.27155316
H	-3.98041392	5.02903060	4.25845888
H	-4.22640460	3.97355832	2.84715406
H	-1.95321395	4.75940531	2.08489529
H	-1.55802137	5.63669483	3.58594930
C	-0.72694706	-4.44968121	4.80309077
C	-0.44255138	-4.45203288	6.17749852
C	0.87111291	-4.60454172	6.62761091
C	1.91374251	-4.77236246	5.70775370
C	1.63744125	-4.74922154	4.33856039
C	0.32375898	-4.58061722	3.88794677
H	-1.25468201	-4.36686044	6.89847652
H	1.07969456	-4.60923785	7.69739934
H	2.93707639	-4.90281048	6.05806569
H	2.44818211	-4.85865303	3.61980581
C	-2.80033268	-5.79524573	4.76946974
C	-3.64636705	-5.87462575	5.87931623
C	-4.14761946	-7.11084814	6.29924807
C	-3.80686233	-8.28096550	5.61147988
C	-2.95934117	-8.20518387	4.49915937
C	-2.45968846	-6.96794375	4.08379118
H	-3.92404569	-4.96727639	6.41120309

H	-4.81064526	-7.15927065	7.16307299
H	-4.20070271	-9.24369253	5.93637412
H	-2.68965308	-9.10971970	3.95409504
C	-1.61190572	-0.33627833	0.45041545
C	-1.43515607	-1.86353057	0.20918609
C	-1.05784283	-0.79536238	-0.78244491
H	2.44994739	-2.55343170	0.42659910
C	-2.68400993	-2.65353858	-0.11597701
C	-0.27908189	-2.66954728	0.71125932
H	2.56181110	-1.99469882	2.13283293
C	-0.59862829	-0.67729392	-2.01841940
C	-2.96022019	0.23773470	0.46053752
O	-3.36098997	-2.49388947	-1.12245895
O	-2.99730516	-3.50559988	0.88981191
C	-4.19757674	-4.31540110	0.66041786
H	-4.35123214	-4.85071579	1.59617495
H	-4.02253924	-4.99361463	-0.18255480
H	-5.04821479	-3.66105042	0.44649587
O	-0.14803742	-3.86357213	0.46451574
O	0.67660104	-1.92487634	1.33780041
C	1.98495548	-2.57914284	1.41850746
H	1.88109520	-3.60884825	1.76010132
O	-3.96169356	-0.17910623	1.05503533
O	-2.96291512	1.42918472	-0.22799860
C	-4.23045995	2.15449068	-0.24021235
H	-5.06816450	1.46244599	-0.35716721
H	-4.16353761	2.84147549	-1.08718287
H	-4.34554098	2.71997566	0.69199148
C	-0.12220017	-1.83266669	-2.85264336
H	-0.54102496	0.32043834	-2.45687903
H	-0.77052086	0.42512034	1.35369995
H	-0.25088441	-2.78377710	-2.32054983
H	0.94589182	-1.71631301	-3.10134932
H	-0.67197690	-1.89300329	-3.80530885

TS2-repro-LA2-RA1-R
COSMO(Et_2O)-ZORA-BLYP-D3(BJ)/DZP
E = -18115.39
G = -17513.95
COSMO(Et_2O)-ZORA-M06-2X/TZ2P//COSMO(Et_2O)-ZORA-BLYP-D3(BJ)/DZP
E = -26007.43
G = -25405.99
N_{imag} = 1, 1446 i cm^{-1}

C	-3.98185535	-3.29927460	0.93000074
N	-4.46548873	-3.03009481	2.29820126
H	-3.88251431	-3.50530190	3.00192221
C	-3.51898478	0.28239189	-0.45421972
S	-6.57980931	-1.34024490	1.92291977
N	-5.77382475	-2.65834784	4.12287336
H	-5.07456074	-3.25422703	4.57569536

C	-6.55110407	-1.78937022	5.01536986
H	-6.90861018	-0.96872578	4.38309246
H	-1.03434678	1.23871401	-1.01600787
C	-5.58606069	-1.21085239	6.07774869
N	-4.39937522	-0.60887544	5.46030752
H	-5.21953322	-2.02581132	6.71474599
P	-4.24711531	0.94310209	5.06142703
C	-4.00667674	2.09874206	6.45056040
C	-3.35712757	1.62628627	7.60172523
C	-4.39150707	3.44737229	6.35794528
C	-3.09332825	2.50339227	8.65585809
H	-3.06129234	0.58529194	7.68065411
C	-4.11859496	4.31952557	7.41491030
H	-4.89835046	3.81310060	5.46678220
C	-3.46921073	3.84856610	8.56256813
H	-2.59470709	2.13267489	9.55058757
H	-4.41512076	5.36549977	7.34406189
H	-3.26068526	4.53090770	9.38619003
C	-5.66726558	1.60196975	4.13728098
C	-6.89595883	1.75408918	4.80678914
C	-5.58572641	1.85125062	2.76021551
C	-8.03363551	2.13217481	4.09601069
H	-6.96286796	1.57849326	5.87916690
C	-6.72386645	2.25390572	2.05900599
H	-4.64250172	1.73013720	2.23515285
C	-7.94664383	2.38320529	2.72034965
H	-8.98608254	2.23823053	4.61404260
H	-6.65779375	2.45019433	0.99471383
H	-8.83566997	2.68178526	2.16525712
C	-2.76496972	1.03286537	4.02130272
C	-2.52084220	0.01002788	3.09011005
C	-1.86717501	2.10583761	4.14395577
C	-1.37696067	0.05928931	2.29235911
H	-3.21910590	-0.81592696	2.99626354
C	-0.72968536	2.15132145	3.33552017
H	-2.04249877	2.88831828	4.87877509
C	-0.48454034	1.12818843	2.41277411
H	-0.02915073	2.97964764	3.43559958
H	0.40921994	1.16089693	1.79002365
H	-6.12283309	-0.51380897	6.73353658
H	-1.19197142	-0.72979258	1.56834968
H	-8.08364162	-1.18995618	7.35054760
H	-3.45550623	2.98291034	-0.41983594
H	-2.86418693	0.76801225	0.28460840
H	-6.43315809	-4.65738476	1.45525040
H	-3.13585827	-3.96504175	1.13071892
H	-8.13578283	-3.71185602	3.79995653
C	-7.80600632	-2.51145453	5.60248554
C	-7.40566481	-3.72025621	6.47502991
C	-8.61895307	-1.49936102	6.44267748

C	-8.68523223	-3.00065854	4.43033963
H	-6.80280846	-4.44051914	5.90301468
H	-6.83221272	-3.41306955	7.35696435
H	-8.30642155	-4.24339869	6.82891421
C	-5.56134714	-2.39808585	2.79732353
C	-4.90598646	-4.14685758	-0.03326005
C	-5.69268890	-5.16239835	0.82179720
C	-5.88702079	-3.32742172	-0.89883426
C	-3.94638534	-4.90289354	-0.98592895
H	-5.01924240	-5.74061960	1.47172258
H	-6.22315868	-5.86549479	0.16339298
H	-5.34700188	-2.64629310	-1.56714407
H	-6.47223026	-4.01838468	-1.52447751
H	-6.57257266	-2.74017467	-0.28062491
H	-4.52652287	-5.49342796	-1.70941284
H	-3.31000074	-4.20111290	-1.54289370
H	-3.29209265	-5.58774486	-0.42613165
C	-3.28153329	-2.08277547	0.27013982
O	-2.09177770	-2.17902035	-0.08090224
N	-4.03404866	-0.97277730	0.10388459
H	-4.95907810	-0.95585460	0.58047163
H	-9.58492635	-3.49983139	4.81993273
H	-8.99283966	-2.15877464	3.79577712
H	-8.84828311	-0.59987233	5.85234774
H	-9.57119786	-1.95199790	6.75616623
C	-2.69951610	0.06934504	-1.72569656
C	-3.21856150	-0.66606841	-2.79724104
C	-2.48240177	-0.81512519	-3.97384341
C	-1.22290239	-0.21358149	-4.09625191
C	-0.70722350	0.53393701	-3.03197193
C	-1.44456136	0.67172554	-1.85110978
H	-4.19761274	-1.13065913	-2.70940901
H	-2.89130696	-1.39860800	-4.79866652
H	-0.64834105	-0.32793856	-5.01511989
H	0.27269375	1.00330612	-3.11761216
C	-4.66998209	1.23314200	-0.76991739
C	-5.92488598	0.76970706	-1.18673827
C	-6.91706652	1.67206029	-1.58211811
C	-6.66551313	3.04781955	-1.56749320
C	-5.41706513	3.51684390	-1.14147995
C	-4.42814034	2.61314830	-0.74460718
H	-6.13565434	-0.29612393	-1.20215364
H	-7.89037186	1.29724419	-1.89825656
H	-7.43936939	3.75069029	-1.87483293
H	-5.21575318	4.58743686	-1.11225836
C	-2.19795744	-2.14120894	5.71704460
C	-1.91742166	-2.51874803	7.19598392
C	-1.25535177	-1.38739673	6.46077848
H	-5.45814961	-2.22019791	9.21647038
C	-1.03104212	-3.70982809	7.45322024

C	-2.96898463	-2.24740184	8.24175412
H	-5.51290723	-4.02256108	9.24779008
C	-0.47106276	-0.33318092	6.65053256
C	-1.95855768	-3.07184481	4.61958562
O	-0.33341857	-4.26137563	6.61465670
O	-1.08202583	-4.07650723	8.76557735
C	-0.24872987	-5.22894973	9.11834732
H	-0.42816334	-5.39138085	10.18316277
H	0.80467420	-4.99995263	8.92745758
H	-0.55413146	-6.10066956	8.53055408
O	-3.03058740	-1.25152359	8.94813033
O	-3.87640780	-3.26481463	8.29162474
C	-4.87918475	-3.13963751	9.35118710
H	-4.38395554	-3.12354733	10.32841898
O	-2.71326683	-4.02078093	4.33550436
O	-0.88046128	-2.72710748	3.86765084
C	-0.57436430	-3.57602398	2.71619545
H	-0.80814212	-4.62016236	2.94278614
H	0.49752199	-3.44316215	2.54323460
H	-1.13610203	-3.23411190	1.84142405
C	0.10424755	0.06472534	7.98065163
H	-3.37499854	-1.34930030	5.53973833
H	-0.26242796	0.30537752	5.78849897
H	-0.24905788	1.06930759	8.26632474
H	1.20469677	0.10808497	7.95241953
H	-0.20247397	-0.63816969	8.76537585

TS2-repro-LA2-RA2-R
COSMO(Et_2O)-ZORA-BLYP-D3(BJ)/DZP
E = -18104.17
G = -17499.80
COSMO(Et_2O)-ZORA-M06-2X/TZ2P//COSMO(Et_2O)-ZORA-BLYP-D3(BJ)/DZP
E = -26000.18
G = -25395.81
N_{imag} = 1, 1673 i cm^{-1}

C	-5.16806683	0.70966516	2.92904677
N	-4.22548881	1.05500789	4.01719325
H	-4.28745711	2.04150186	4.27674679
C	-6.79930530	-2.42181257	4.41067051
S	-2.85691438	-1.27158242	4.40271209
N	-2.60749014	1.14592385	5.59710377
H	-2.86684001	2.13826873	5.57627697
C	-1.37065141	0.93154640	6.36652327
H	-1.05534922	1.95904549	6.58647250
H	-8.29955215	-3.01768447	2.20739726
C	-0.23784383	0.28981121	5.54829042
N	-0.03915866	0.97153948	4.24402376
H	-0.48420234	-0.76255303	5.37865554
P	0.85119035	0.16128462	3.16475565
C	0.87553691	1.06334896	1.59490537

C	-0.10256755	2.02199680	1.30266623
C	1.80353349	0.68048521	0.60917256
C	-0.16047281	2.58750172	0.02796597
H	-0.81619267	2.32770145	2.05558881
C	1.72952936	1.23942479	-0.66630125
H	2.57562200	-0.05266403	0.83300727
C	0.74221535	2.18902888	-0.95985408
H	-0.91541575	3.34258440	-0.18575227
H	2.44612496	0.93904617	-1.43005866
H	0.68910698	2.62649764	-1.95664728
C	0.19099872	-1.49900937	2.78850511
C	-0.63968212	-1.69355891	1.67314682
C	0.37503949	-2.54477782	3.71072307
C	-1.29678691	-2.91118846	1.49577355
H	-0.78547628	-0.89131862	0.95544498
C	-0.29243139	-3.75690772	3.53252374
H	1.03120745	-2.41185634	4.56854425
C	-1.13486195	-3.93750341	2.43054434
H	-1.95381103	-3.05119573	0.63961313
H	-0.16168698	-4.55792156	4.25925351
H	-1.66155065	-4.88090181	2.29908410
C	2.56763850	-0.14379826	3.70971127
C	3.11077666	0.72070312	4.67368516
C	3.33557730	-1.20812106	3.20667831
C	4.41676960	0.52193478	5.12663255
H	2.52181573	1.55650772	5.04141045
C	4.64444315	-1.39490169	3.65679726
H	2.91044089	-1.89596406	2.47821419
C	5.18410237	-0.53250460	4.61896732
H	5.24006168	-2.21817714	3.26403265
H	6.20283791	-0.68479294	4.97443293
H	0.67526431	0.33591002	6.16110595
H	4.83746034	1.19292514	5.87490106
H	0.55079879	0.34161420	8.30061445
H	-7.72591404	-4.74968634	5.43312160
H	-7.84548465	-2.09792390	4.31785447
H	-5.50038465	-1.65102626	1.27529959
H	-5.68171157	1.66463702	2.76670217
H	-3.77766576	0.47553177	7.81631490
C	-1.59391824	0.26680048	7.77885632
C	-1.68582362	-1.27419416	7.72895065
C	-0.41717684	0.67913558	8.69647558
C	-2.90361720	0.82484802	8.38106772
H	-2.47353497	-1.60046532	7.04115176
H	-0.73976117	-1.72728227	7.39917523
H	-1.90785224	-1.65713210	8.73690737
C	-3.23442993	0.37001861	4.67667077
C	-4.55096277	0.31951446	1.51085385
C	-4.49853622	-1.20374987	1.25869783
C	-5.46723404	0.95714695	0.43822027

C	-3.13965062	0.91721829	1.38985256
H	-3.87823442	-1.70976300	2.00195410
H	-4.06929701	-1.38373113	0.26164071
H	-5.46965252	2.05462049	0.52425616
H	-5.10489737	0.69236178	-0.56616903
H	-6.49981526	0.59928683	0.54491829
H	-2.74566332	0.73183214	0.38058516
H	-3.14553200	2.00136112	1.55301479
H	-2.45625950	0.46548905	2.11476406
C	-6.31488798	-0.24987290	3.32436711
O	-7.45504360	-0.09274176	2.85111122
N	-5.96269878	-1.24223971	4.17054754
H	-4.94086468	-1.39598228	4.28528311
H	-3.00595936	0.49008514	9.42356607
H	-2.90994461	1.92594732	8.37209323
H	-0.37562204	1.77166509	8.82043396
H	-0.54628786	0.23022755	9.69245675
C	-6.49611079	-3.43675993	3.30927225
C	-5.27730807	-4.13097856	3.31089276
C	-4.92701505	-4.94130885	2.23102002
C	-5.79498110	-5.07383237	1.13931514
C	-7.01605838	-4.39276205	1.13838011
C	-7.36317701	-3.57456620	2.22009945
H	-4.59360129	-4.01659945	4.15084331
H	-3.97446185	-5.46865103	2.23545136
H	-5.51849019	-5.70402069	0.29400581
H	-7.69506305	-4.48912989	0.29108290
C	-6.56135390	-2.97183845	5.81401947
C	-5.78445089	-2.28937703	6.75908158
C	-5.57919995	-2.82961115	8.03378272
C	-6.15810135	-4.05343688	8.38189643
C	-6.94174548	-4.73812207	7.44322928
C	-7.13574450	-4.20252452	6.16817620
H	-5.32724550	-1.33940690	6.49850132
H	-4.96087187	-2.29167667	8.75159134
H	-5.99893274	-4.47480028	9.37420738
H	-7.39584945	-5.69418052	7.70360773
C	-0.66079135	3.62992177	4.34024888
C	0.45126302	4.66959320	4.03619220
C	0.13690637	4.13925668	5.40491087
H	2.94302922	3.08447552	1.30761722
C	0.03114532	6.10982416	3.87937210
C	1.67146087	4.20334379	3.29145944
H	2.44706702	4.35793887	0.13783887
C	0.59086182	4.22094791	6.64923158
C	-2.06374836	3.93032861	4.06002154
O	-1.08350530	6.54583005	4.13211334
O	1.07933439	6.88644111	3.48950316
C	0.77554146	8.31308083	3.35821821
H	1.70742523	8.76945158	3.01783112

H	0.46927927	8.72054253	4.32724714
H	-0.02366121	8.45732817	2.62397375
O	2.59389685	3.56471337	3.77797695
O	1.59522544	4.55168138	1.98035296
C	2.74161507	4.14853752	1.16748578
H	3.62002592	4.73923269	1.45310748
O	-3.00571227	3.83896225	4.86449392
O	-2.26448267	4.21980044	2.74012019
C	-3.62595213	4.60855723	2.39061422
H	-4.32694903	3.79307223	2.59201470
H	-3.59416926	4.82787972	1.32079746
H	-3.91695330	5.49573769	2.96379590
C	1.80816860	5.01800328	7.03486418
H	0.06741635	3.68397250	7.44331828
H	-0.30596233	2.25085182	4.18811896
H	1.54416266	5.86471119	7.68899399
H	2.31400112	5.40987137	6.14352955
H	2.52487365	4.39320173	7.59090924

TS2-repro-LA1-RA1-S (TS2-S)

COSMO(Et_2O)-ZORA-BLYP-D3(BJ)/DZP

E = -18108.09

G = -17504.97

COSMO(Et_2O)-ZORA-M06-2X/TZ2P//COSMO(Et_2O)-ZORA-BLYP-D3(BJ)/DZP

E = -26007.71

G = -25404.59

N_{imag} = 1, 1024 i cm^{-1}

C	-5.61044683	-2.47058952	2.60689786
N	-4.43227815	-1.62744976	2.47758851
H	-3.99685204	-1.56691296	1.55182870
C	-5.52118285	-5.85916308	4.40870421
S	-4.21315833	-1.32883485	5.15810812
N	-2.51539088	-0.61989646	3.16215208
H	-2.40973922	-0.45056414	2.16307823
C	-1.52603665	0.00515364	4.02477129
H	-2.01500015	0.12885484	5.00027593
H	-3.60481551	-4.00564724	5.23031461
C	-1.17449964	1.43324459	3.46439431
N	-1.56374741	1.68549955	2.05814226
H	-0.09329122	1.59722222	3.54328808
P	-2.72169364	2.76445578	1.71472164
C	-2.39127144	4.33657715	2.58195417
C	-1.05313327	4.70034760	2.80270589
C	-3.42697795	5.19028316	2.99377454
C	-0.75142936	5.91630531	3.41697054
H	-0.26187764	4.02470777	2.49388067
C	-3.11937720	6.40840371	3.60607524
H	-4.46566061	4.90629322	2.84015136
C	-1.78459811	6.77318408	3.81513905
H	0.28770852	6.19549722	3.58758462

H	-3.92335488	7.07171724	3.92282772
H	-1.54924249	7.72266446	4.29484139
C	-4.45147360	2.37362417	2.16503965
C	-4.73501805	1.82688566	3.42680051
C	-5.50777166	2.74641914	1.31476686
C	-6.05782949	1.65565482	3.83343565
H	-3.93807651	1.52635343	4.09875485
C	-6.83018460	2.59177960	1.73735077
H	-5.29934990	3.16520551	0.33299213
C	-7.10557667	2.05266167	2.99718507
H	-6.26052083	1.19746878	4.79979857
H	-7.64484646	2.88407205	1.07548472
H	-8.13828517	1.92396968	3.31981278
C	-2.75267002	3.11284221	-0.06336776
C	-3.02939921	2.07509909	-0.97056155
C	-2.60635698	4.43252797	-0.52084317
C	-3.13567729	2.36310943	-2.33064335
H	-3.13404219	1.05074886	-0.62011011
C	-2.72855997	4.70894528	-1.88413401
H	-2.40664007	5.23998246	0.17952787
C	-2.99310767	3.67657519	-2.78775828
H	-2.60919685	5.73183229	-2.23904619
H	-3.07953112	3.89545306	-3.85174441
H	-1.64527313	2.17773895	4.11930990
H	-3.31935216	1.55158815	-3.03307571
H	0.95920654	0.74278847	5.11878965
H	-7.81769319	-6.96499260	3.46935357
H	-5.47978632	-6.44088520	3.47519549
H	-6.17028994	-0.19388799	1.17619888
H	-6.17375970	-2.09469362	3.47081133
H	-1.32702380	-2.79976573	4.00220385
C	-0.26566254	-0.89819059	4.28523867
C	0.60395554	-1.07250742	3.02671091
C	0.56344578	-0.23934780	5.41285338
C	-0.73072705	-2.28847875	4.76843374
H	0.05903754	-1.59879663	2.23611764
H	0.95157013	-0.11563155	2.61749312
H	1.49200852	-1.67320711	3.27518308
C	-3.68802537	-1.19540472	3.53418077
C	-6.53872089	-2.35020842	1.35263710
C	-7.01629042	-0.88566254	1.26397092
C	-5.81909606	-2.73980835	0.04170003
C	-7.75554741	-3.27743266	1.56820132
H	-7.58529976	-0.60555287	2.16071636
H	-7.66597772	-0.75262245	0.38638321
H	-5.42458717	-3.76084661	0.09601540
H	-6.53427833	-2.67594649	-0.79212551
H	-4.98613524	-2.06189346	-0.18583944
H	-8.47128904	-3.14841985	0.74382151
H	-7.45623043	-4.33436729	1.59687900

H	-8.27127506	-3.04216733	2.51111390
C	-5.18375383	-3.92983247	2.88866860
O	-4.41426532	-4.56122263	2.15005918
N	-5.74526949	-4.46709043	4.00270118
H	-6.26618730	-3.86106773	4.63394270
H	0.14443361	-2.91519722	4.99464636
H	-1.34957389	-2.20530850	5.67032551
H	-0.04603753	-0.10681133	6.31993280
H	1.42092629	-0.87918041	5.66776383
C	-4.19908490	-6.08192475	5.14168684
C	-3.84380639	-7.39656161	5.48178562
C	-2.65184599	-7.65771251	6.16018902
C	-1.79880226	-6.60143986	6.50810538
C	-2.14928673	-5.29211110	6.16783856
C	-3.34464599	-5.03078549	5.48582590
H	-4.51260080	-8.21819080	5.22295734
H	-2.38732640	-8.68316730	6.41831118
H	-0.86752067	-6.80119836	7.03812082
H	-1.49078022	-4.46650931	6.42970934
C	-6.73371074	-6.30863358	5.21565751
C	-6.78008281	-6.13958650	6.60569734
C	-7.93003678	-6.48902781	7.32164846
C	-9.03991694	-7.01836504	6.65397257
C	-8.99641788	-7.19390949	5.26544554
C	-7.84923113	-6.83634389	4.55136864
H	-5.91163335	-5.74322287	7.12979943
H	-7.95657071	-6.35263627	8.40259363
H	-9.93324021	-7.29654224	7.21260446
H	-9.85526080	-7.61067083	4.73982838
C	-0.53995245	0.05036815	0.10491670
C	-0.05967329	0.80738820	-1.17771253
C	0.79265581	-0.11464058	-0.33710706
H	-0.88758199	0.39339573	-5.14687435
C	0.22586868	2.28365766	-1.14919968
C	-0.42725439	0.21698858	-2.51870587
H	0.87533958	0.55158728	-5.48421863
C	2.01308505	-0.63903789	-0.33231473
C	-1.54109174	-1.02338376	0.06332027
O	0.07132876	3.04919889	-2.08821806
O	0.72883859	2.67484603	0.05997582
C	1.10631011	4.08287071	0.11564510
H	1.52914923	4.22548777	1.11254788
H	0.22671112	4.71744804	-0.03120798
H	1.85313490	4.30438475	-0.65526974
O	-1.32187271	-0.59979773	-2.69226434
O	0.40179057	0.65189654	-3.50337304
C	0.12119253	0.10655560	-4.83127487
H	0.20919644	-0.98487830	-4.81522005
O	-2.77150170	-0.84969648	0.13515820
O	-0.98629146	-2.26531703	0.15083363

C	-1.93268298	-3.36783006	0.33496673
H	-2.45886107	-3.27928564	1.28857119
H	-1.31734171	-4.27116552	0.32302112
H	-2.65980993	-3.37862230	-0.48273240
C	3.08989518	-0.27765863	-1.31990717
H	-1.02118353	0.98649335	1.15848381
H	2.25780831	-1.36659951	0.44638459
H	3.45239147	-1.16862351	-1.85813883
H	3.96319923	0.16399951	-0.81198144
H	2.71830400	0.44029538	-2.06201825

TS2-repro-LA1-RA2-S
COSMO(Et_2O)-ZORA-BLYP-D3(BJ)/DZP
E = -18100.88
G = -17502.76
COSMO(Et_2O)-ZORA-M06-2X/TZ2P//COSMO(Et_2O)-ZORA-BLYP-D3(BJ)/DZP
E = -25997.64
G = -25399.52
N_{imag} = 1, 1190 i cm^{-1}

C	-4.48111545	-2.25344797	4.60458358
N	-4.09059311	-1.00032857	3.96437686
H	-4.70298730	-0.65424256	3.22660839
C	-2.04026020	-4.93256523	3.40434408
S	-1.90230110	-0.76839515	5.51591845
N	-2.95614841	0.93853519	3.64801264
H	-3.63707552	1.05562563	2.88591382
C	-2.19101596	2.18087977	3.86638004
H	-2.42243158	2.76173708	2.96512937
H	0.02997815	-4.44550852	1.67195050
C	-0.67510024	1.96123590	3.87143401
N	-0.16732079	1.73139402	2.49535237
H	-0.44201008	1.12488043	4.54208678
P	1.43951079	1.66700019	2.33147403
C	1.87610354	1.79798755	0.58210354
C	0.98392682	2.39999353	-0.31764783
C	3.14482840	1.37938168	0.15392141
C	1.36508375	2.56710420	-1.65087692
H	-0.00468760	2.71087068	0.01015437
C	3.52420421	1.57423832	-1.17440819
H	3.82646648	0.89041535	0.84675528
C	2.63468322	2.16790385	-2.07728192
H	0.65796262	2.99733987	-2.35635302
H	4.50810870	1.24689324	-1.50915975
H	2.92758664	2.30417639	-3.11811292
C	2.17270744	0.21069888	3.14600924
C	1.30060915	-0.83840326	3.48337510
C	3.51560920	0.17183180	3.55675796
C	1.76515744	-1.90491007	4.25255303
H	0.26388837	-0.80769656	3.16107852
C	3.98189090	-0.91771119	4.29812841

H	4.18620630	0.99956333	3.32920761
C	3.10308943	-1.94920334	4.65521068
H	1.07926225	-2.69754089	4.53863110
H	5.02357504	-0.95079913	4.61639153
H	3.46214876	-2.78705968	5.25201710
C	2.25652460	3.08526717	3.15049646
C	2.38900323	3.10023277	4.55160053
C	2.63516862	4.21508727	2.40568606
C	2.89321717	4.23173735	5.19518038
H	2.11215022	2.22546055	5.13673644
C	3.14814432	5.34128331	3.05502467
H	2.54014776	4.21153133	1.32191764
C	3.27401312	5.35293680	4.44860329
H	3.45229900	6.20917150	2.47063745
H	3.67183124	6.23321234	4.95260389
H	-0.20729148	2.86423737	4.28290847
H	2.99079266	4.23762689	6.28032310
H	-1.25542640	4.65855637	4.59666961
H	-1.80945305	-7.26032496	2.05906217
H	-2.18900642	-4.78952472	2.32587217
H	-6.03414446	-0.17210453	5.53243042
H	-3.94295826	-2.26485571	5.56093940
H	-4.56070286	1.95120223	5.45449382
C	-2.71333266	3.06736541	5.05921762
C	-2.10400110	2.67736088	6.42327128
C	-2.33896574	4.53384796	4.73295052
C	-4.25177900	2.95448010	5.13534026
H	-2.35416148	1.64356523	6.68581988
H	-1.00897912	2.76897791	6.41974417
H	-2.49394900	3.34974624	7.20260846
C	-3.02442599	-0.22269300	4.33402928
C	-6.00430515	-2.28567244	4.93299647
C	-6.28587550	-1.15580186	5.95077403
C	-6.88441531	-2.07908738	3.67989968
C	-6.32417674	-3.64983611	5.58533064
H	-5.69714113	-1.29701199	6.86915934
H	-7.35216731	-1.15073913	6.21959604
H	-6.66784966	-2.83988799	2.92227078
H	-7.94569974	-2.14134822	3.96265821
H	-6.73267880	-1.08314106	3.23564142
H	-7.37024066	-3.66540414	5.92393437
H	-6.17569289	-4.47405043	4.87725881
H	-5.68175995	-3.82127849	6.46257020
C	-3.96163192	-3.45328188	3.77519382
O	-4.61501169	-4.03611112	2.89979243
N	-2.68793010	-3.81448474	4.10217926
H	-2.13219228	-3.16833553	4.67675166
H	-4.64371678	3.68087626	5.86223033
H	-4.71368646	3.16178010	4.15826259
H	-2.83993689	4.87119011	3.81320792

H	-2.65134394	5.19444350	5.55456234
C	-0.54489803	-4.97364221	3.69120419
C	-0.07822442	-5.32696623	4.97009440
C	1.29175833	-5.46798791	5.20628693
C	2.20886023	-5.26958614	4.16196074
C	1.75181920	-4.89485292	2.89241949
C	0.38066757	-4.74441083	2.66087863
H	-0.79094917	-5.51971827	5.77550126
H	1.64265812	-5.74555901	6.20317443
H	3.27640132	-5.39054514	4.34321606
H	2.46166597	-4.70536476	2.08357217
C	-2.67270079	-6.27812309	3.77692682
C	-3.44151998	-6.44870955	4.93200181
C	-3.94927873	-7.70910043	5.26545762
C	-3.69154659	-8.81186773	4.44463595
C	-2.92283048	-8.64506028	3.28522154
C	-2.41652116	-7.38507064	2.95647963
H	-3.65074144	-5.59394994	5.57056261
H	-4.55097761	-7.82724791	6.16684847
H	-4.08906153	-9.79320926	4.70234255
H	-2.71900437	-9.49753745	2.63736182
C	-1.93809946	0.66537804	0.70415192
C	-1.40499267	-0.10601506	-0.55388909
C	-2.25538253	-0.70821635	0.53439031
H	-3.00015482	2.07670526	-3.54001796
C	0.00986179	-0.60201366	-0.55079964
C	-2.08978917	0.23738816	-1.85167167
H	-1.34753508	2.36908899	-4.19724232
C	-2.64547953	-1.92294603	0.89775028
C	-2.93725476	1.72119494	0.56319659
O	0.64759204	-0.85184727	0.46010080
O	0.45867224	-0.82954370	-1.82115738
C	1.81013064	-1.37336921	-1.94014563
H	2.25674724	-0.87614471	-2.80805168
H	1.73827708	-2.45692678	-2.10948341
H	2.37820646	-1.16372563	-1.03033770
O	-3.17767349	-0.20522229	-2.20464761
O	-1.38458045	1.15822082	-2.55769524
C	-2.03087232	1.62518276	-3.77965465
H	-2.17298315	0.79201539	-4.47707594
O	-4.02625785	1.75850399	1.16089723
O	-2.49700839	2.76504634	-0.20682020
C	-3.41041699	3.90051088	-0.29203883
H	-4.35970346	3.59007504	-0.74231205
H	-2.89890407	4.62888163	-0.92579414
H	-3.59578365	4.31452565	0.70521398
C	-2.37930157	-3.15049702	0.06443348
H	-3.17842411	-2.05220999	1.83517874
H	-0.94713367	1.14906569	1.67870015
H	-1.43350587	-3.62915370	0.36773601

H	-2.29196135	-2.89602118	-1.00079564
H	-3.17920103	-3.89307099	0.18723528

TS2-repro-LA2-RA1-S
COSMO(Et_2O)-ZORA-BLYP-D3(BJ)/DZP
E = -18106.00
G = -17503.88
COSMO(Et_2O)-ZORA-M06-2X/TZ2P//COSMO(Et_2O)-ZORA-BLYP-D3(BJ)/DZP
E = -26001.76
G = -25399.64
N_{imag} = 1, 1521 i cm^{-1}

C	-3.05230310	-2.74191325	1.23302962
N	-3.84293305	-2.67105234	2.47816540
H	-3.31474374	-3.11288591	3.24125733
C	-3.30176348	0.61054046	-0.64565473
S	-6.13389178	-1.26846833	1.89075670
N	-5.45695801	-2.65565673	4.07884022
H	-4.74877099	-3.18439083	4.58350421
C	-6.35782962	-1.90974305	4.97018605
H	-6.74183243	-1.06602268	4.38564570
H	-0.72957437	0.68868316	-1.51521555
C	-5.50186240	-1.36128806	6.14343566
N	-4.27917109	-0.69954152	5.66206611
H	-5.17684175	-2.19696349	6.78258287
P	-4.24188569	0.88999909	5.37952823
C	-4.31645873	1.89858043	6.90197072
C	-3.98031008	1.28857309	8.12049315
C	-4.71135403	3.24613899	6.87517356
C	-4.03017353	2.02793856	9.30343692
H	-3.66563321	0.24824556	8.13232331
C	-4.74883873	3.98427994	8.06101460
H	-5.00319675	3.71349944	5.93629608
C	-4.40847030	3.37559345	9.27472071
H	-3.76958562	1.55405828	10.24927824
H	-5.05149268	5.03070524	8.03903756
H	-4.44281776	3.95115602	10.19944693
C	-5.62709291	1.51665951	4.37782231
C	-6.90843203	1.62175860	4.94924190
C	-5.43679940	1.84049054	3.02618416
C	-7.98682289	2.03710180	4.16916540
H	-7.06112348	1.38733684	6.00126024
C	-6.51278268	2.29293863	2.26154537
H	-4.45401845	1.74545332	2.57165896
C	-7.78665406	2.38211420	2.82562007
H	-8.98023068	2.10730167	4.61123187
H	-6.35617066	2.56103108	1.22339143
H	-8.62664857	2.72170027	2.21988698
C	-2.73104388	1.24560764	4.44833292
C	-2.14112244	0.23090889	3.68123428
C	-2.16175091	2.52596541	4.49225251

C	-0.96681159	0.49632808	2.97586592
H	-2.58523092	-0.75889207	3.65330514
C	-0.99517475	2.78716692	3.77254570
H	-2.60390818	3.30568466	5.10809197
C	-0.39551310	1.77159230	3.01861689
H	-0.54675439	3.77969229	3.81170574
H	0.52014049	1.97564449	2.46331345
H	-6.11480913	-0.70624920	6.77745034
H	-0.51768586	-0.29086434	2.37664325
H	-7.97073209	-1.78200698	7.34454678
H	-3.36327694	3.30835402	-0.73718952
H	-2.50625265	1.18548339	-0.14801690
H	-4.48668137	-5.11692271	1.67638760
H	-2.08931104	-3.11355810	1.60590471
H	-7.78975776	-3.67000060	3.42605162
C	-7.57860048	-2.77641163	5.41300361
C	-7.12761715	-4.08508423	6.09779118
C	-8.46287638	-1.95237861	6.37733187
C	-8.40383336	-3.12471283	4.15478170
H	-6.54903927	-4.70861683	5.40323833
H	-6.51275011	-3.89786078	6.98872217
H	-8.00848462	-4.66369905	6.41330981
C	-5.08354777	-2.23671901	2.82377899
C	-3.51894832	-3.82666194	0.18931354
C	-3.66107667	-5.16347715	0.95290888
C	-4.85102667	-3.49896309	-0.51518952
C	-2.41102220	-3.95682793	-0.88258146
H	-2.73810361	-5.40800471	1.49980947
H	-3.87011254	-5.97878637	0.24557387
H	-4.76235104	-2.59225801	-1.12593622
H	-5.11589366	-4.33359000	-1.18253158
H	-5.66356347	-3.35172910	0.20351186
H	-2.67140652	-4.76737570	-1.57870020
H	-2.30133572	-3.03009878	-1.46164345
H	-1.43842296	-4.19056546	-0.42547938
C	-2.62584443	-1.40321955	0.58706614
O	-1.41640735	-1.19685252	0.38136150
N	-3.60332443	-0.56968916	0.17754557
H	-4.56069006	-0.72759732	0.54968933
H	-9.26610853	-3.75039007	4.43011243
H	-8.77118630	-2.21224108	3.66509352
H	-8.71274442	-0.97459022	5.93826351
H	-9.40419980	-2.48745029	6.57104636
C	-2.75621013	0.16399978	-2.01139283
C	-3.59254212	-0.41032814	-2.97747150
C	-3.06505781	-0.88645606	-4.18083995
C	-1.69258975	-0.78311986	-4.43673050
C	-0.85442948	-0.19859280	-3.48154419
C	-1.38529550	0.26687333	-2.27444257
H	-4.66165796	-0.49015698	-2.79743716

H	-3.72720539	-1.33590808	-4.92087963
H	-1.28036182	-1.15462529	-5.37480801
H	0.21606759	-0.11604534	-3.67023556
C	-4.52863140	1.49382668	-0.80204996
C	-5.81565043	0.96226567	-0.97342706
C	-6.90385454	1.80094613	-1.22807095
C	-6.72295863	3.18543150	-1.30818139
C	-5.44555812	3.72581754	-1.12194124
C	-4.35810077	2.88342108	-0.87182000
H	-5.98203749	-0.10940976	-0.90040095
H	-7.89721033	1.37049341	-1.35228754
H	-7.57278972	3.84015181	-1.49896899
H	-5.29544681	4.80426606	-1.16763426
C	-2.28055373	-2.30102308	6.46862570
C	-0.83524823	-1.78131498	6.69837086
C	-1.73903992	-2.32259540	7.78115831
H	1.27613260	-3.87739946	4.01513589
C	-0.59717855	-0.30321728	6.77196069
C	0.23554454	-2.69076399	6.15676886
H	2.17197659	-2.38083588	3.55928803
C	-1.77539330	-2.45823175	9.10024427
C	-2.49674898	-3.47042404	5.62829575
O	-1.46921743	0.54780047	6.80880555
O	0.72909413	-0.01912434	6.92849693
C	1.02023598	1.41190484	7.02901726
H	2.10114961	1.47258168	7.17499826
H	0.48004131	1.84702209	7.87627295
H	0.71572370	1.91153310	6.10350653
O	0.45566088	-3.82831894	6.55409844
O	0.84672740	-2.12363782	5.08562794
C	1.79487361	-2.98705909	4.38608471
H	2.60785858	-3.28099069	5.05885422
O	-2.09410486	-3.61342762	4.46568051
O	-3.34266812	-4.38255672	6.22271960
C	-3.62854049	-5.57515757	5.42275138
H	-4.07343332	-5.30693365	4.45886567
H	-4.33518819	-6.15317401	6.02207894
H	-2.70425850	-6.13757398	5.25050311
C	-0.63950266	-2.07372371	10.00904019
H	-3.23938934	-1.34111017	6.04642202
H	-2.67473015	-2.88248227	9.55693657
H	-0.30953072	-2.92823128	10.62100081
H	-0.94562032	-1.27820865	10.70823549
H	0.22034208	-1.71192179	9.43022161

TS2-repro-LA2-RA2-S
COSMO(Et_2O)-ZORA-BLYP-D3(BJ)/DZP
E = -18099.86
G = -17496.39
COSMO(Et_2O)-ZORA-M06-2X/TZ2P//COSMO(Et_2O)-ZORA-BLYP-D3(BJ)/DZP

E = -25995.03
G = -25391.56
N_{imag} = 1, 1707 i cm^{-1}

C	-4.83493245	0.73626015	2.25521566
N	-3.75731634	0.76411813	3.27058107
H	-3.66547655	1.69338615	3.69517524
C	-7.29012290	-1.43884292	4.28313494
S	-3.08437119	-1.86144444	3.49786179
N	-2.64673801	0.21588024	5.17689668
H	-2.77664851	1.21940332	5.35272163
C	-1.66237652	-0.40781279	6.08137447
H	-1.37517441	0.44153988	6.71488692
H	-8.89949364	-2.95595316	2.66784578
C	-0.40016561	-0.86478631	5.33178923
N	0.13854605	0.23506655	4.50417578
H	-0.66319427	-1.72659432	4.71615143
P	1.12183483	-0.01667958	3.25014623
C	0.55605710	0.69093345	1.66272968
C	0.24335124	2.05983838	1.63331435
C	0.53197403	-0.05324466	0.47372215
C	-0.11076087	2.67132693	0.43195328
H	0.27830793	2.65428274	2.53871864
C	0.18037147	0.56714563	-0.72867774
H	0.79478867	-1.10794606	0.47706608
C	-0.14205712	1.92628528	-0.75226824
H	-0.35462398	3.73256103	0.41997223
H	0.15668494	-0.01751768	-1.64776021
H	-0.41970967	2.40496528	-1.69092655
C	1.42476854	-1.78477135	2.95566611
C	0.34518763	-2.60787119	2.58547103
C	2.69978157	-2.34162683	3.14350341
C	0.54526416	-3.97620774	2.40498691
H	-0.64964095	-2.18549045	2.45405013
C	2.89174814	-3.71401449	2.95684505
H	3.53731200	-1.70927418	3.42904542
C	1.81773538	-4.53089198	2.58811806
H	-0.29438174	-4.61020799	2.12182328
H	3.88270339	-4.14368278	3.09911788
H	1.97154236	-5.59989958	2.44446234
C	2.74487017	0.76657916	3.50371731
C	3.21170097	0.91979164	4.81690021
C	3.54975295	1.14124810	2.41605954
C	4.49784333	1.41633701	5.03833195
H	2.55585768	0.69753416	5.65378583
C	4.82986080	1.65065140	2.64647019
H	3.17811780	1.04294488	1.39770733
C	5.30743337	1.77781275	3.95658999
H	5.45391008	1.94931940	1.80460159
H	6.30741780	2.17405316	4.13263940
H	0.33989022	-1.19586884	6.07758971

H	4.85980969	1.53833448	6.05852940
H	-0.45087745	-1.26042306	8.34260421
H	-8.49489445	-2.82700255	6.32643528
H	-8.17608534	-1.01572221	3.78697496
H	-6.09605226	-1.36636234	0.75864952
H	-5.01281460	1.80645334	2.09382060
H	-4.29204787	-1.50878458	6.32588757
C	-2.23683258	-1.50004839	7.08261945
C	-1.96569974	-2.96062144	6.65208202
C	-1.54560761	-1.26322090	8.44533532
C	-3.75392288	-1.29440830	7.25482655
H	-2.40211197	-3.16786894	5.66999193
H	-0.88958777	-3.18195731	6.61302555
H	-2.41707108	-3.63728589	7.39365748
C	-3.14848910	-0.22522251	3.99786150
C	-4.51326303	0.15559004	0.81436235
C	-5.00789934	-1.29499614	0.61779621
C	-5.26673183	1.05843839	-0.19507618
C	-3.00058917	0.25084687	0.54527946
H	-4.52073533	-1.97797128	1.32019258
H	-4.77905173	-1.61606817	-0.40954431
H	-4.89180135	2.09258498	-0.15216852
H	-5.11807390	0.68132190	-1.21731150
H	-6.34408187	1.07338026	0.01970983
H	-2.79679550	-0.02060190	-0.50012461
H	-2.62563251	1.26994131	0.70524258
H	-2.43811524	-0.42424758	1.19851195
C	-6.19523353	0.24240511	2.80726645
O	-7.25038784	0.82594471	2.49834758
N	-6.12796895	-0.81657117	3.64189556
H	-5.23279536	-1.31984731	3.71375748
H	-4.13937076	-1.96265459	8.03873306
H	-3.98851909	-0.26189845	7.54490667
H	-1.85506896	-0.30041767	8.87216445
H	-1.81620872	-2.05794341	9.15616275
C	-7.22400023	-2.93644296	4.02434493
C	-6.21958984	-3.70665574	4.63028781
C	-6.10827412	-5.06934524	4.35110019
C	-7.00914858	-5.68095242	3.46931325
C	-8.01646663	-4.91964788	2.86800828
C	-8.11902212	-3.55048976	3.14258078
H	-5.52041458	-3.23752448	5.31987915
H	-5.32041017	-5.65684392	4.82199309
H	-6.92603118	-6.74612019	3.25442107
H	-8.72216392	-5.39004748	2.18342297
C	-7.35927000	-1.07525809	5.77105152
C	-6.77570727	0.10827344	6.24104862
C	-6.86624135	0.45986182	7.59028023
C	-7.53996211	-0.37106119	8.49015357
C	-8.13064422	-1.55240230	8.02743180

C	-8.04344569	-1.89971810	6.67578079
H	-6.22765513	0.74666253	5.55246618
H	-6.39891972	1.38002589	7.94060565
H	-7.60163159	-0.10341349	9.54483454
H	-8.65636476	-2.20892127	8.72082671
C	-0.47993753	2.84075642	5.04300524
C	-0.01439622	3.35168346	6.43546477
C	0.76019202	3.52711000	5.15946333
H	-1.55645810	6.25882491	8.82239234
C	0.44779965	2.35825773	7.46861019
C	-0.58849694	4.65884459	6.93017132
H	0.06495152	6.42284642	9.59259154
C	1.92304233	4.00264646	4.73404548
C	-1.71675823	3.33166931	4.43940007
O	1.47069195	1.69297289	7.40885216
O	-0.44508246	2.28802746	8.49846228
C	0.02945640	1.57829979	9.68817110
H	-0.84767217	1.47508400	10.33036406
H	0.79970175	2.18627048	10.17760452
H	0.43514620	0.60174614	9.41817818
O	-1.40469103	5.34099061	6.32655036
O	-0.00630565	5.02916831	8.10384963
C	-0.47588793	6.30378887	8.65134058
H	-0.24171327	7.11744295	7.95713485
O	-2.86041436	3.05590692	4.84052098
O	-1.49998948	4.01966340	3.28145230
C	-2.68633222	4.29005895	2.47247317
H	-3.07774290	3.34935823	2.07131498
H	-2.33682086	4.92931968	1.65850099
H	-3.45070641	4.79711949	3.06913108
C	2.96231954	4.59256873	5.64495867
H	2.15875031	3.94371134	3.66814468
H	-0.23572455	1.46195629	4.78318901
H	3.89059998	4.00311299	5.60086506
H	2.61094908	4.60695201	6.68451511
H	3.21802928	5.62189307	5.34689734

TS3-depro-LA1'-RA1-Ha (TS3)
COSMO(Et_2O)-ZORA-BLYP-D3(BJ)/DZP
E = -17969.52
G = -17370.43
COSMO(Et_2O)-ZORA-M06-2X/TZ2P//COSMO(Et_2O)-ZORA-BLYP-D3(BJ)/DZP
E = -25735.34
G = -25136.25
N_{imag} = 1, 1625 i cm^{-1}

C	-6.05602873	-2.05534187	2.32228086
N	-4.70720356	-1.47628079	2.31696397
H	-4.10616428	-1.71793179	1.52245737
C	-8.51533130	0.80296573	2.97742371
S	-4.86109009	-0.68637204	4.90872046

N	-2.70795380	-0.99368478	3.30615131
H	-2.33530529	-1.40050589	2.43860312
C	-1.82324835	-0.13846186	4.09741923
H	-2.44570246	0.27845484	4.89841419
H	-10.87104878	2.05525123	2.57302700
C	-1.36273036	1.02816398	3.18707611
N	-2.51557689	1.72869691	2.61125405
H	-0.78287149	0.61993253	2.35116537
P	-3.15992086	3.05801709	3.24696271
C	-2.12361136	4.54719192	3.04937058
C	-1.10192141	4.50796162	2.08801279
C	-2.32877206	5.70653323	3.81580703
C	-0.29137115	5.62887957	1.89049636
H	-0.92955292	3.60621945	1.50641224
C	-1.52273130	6.82746519	3.60521637
H	-3.10640797	5.72928679	4.57829641
C	-0.50459071	6.78871824	2.64363098
H	0.50745502	5.59135972	1.15058907
H	-1.68230693	7.72889218	4.19602011
H	0.12717537	7.66275272	2.48757854
C	-3.45938606	2.93942305	5.03221544
C	-2.35465592	3.06465268	5.89858882
C	-4.71314858	2.56985778	5.54009076
C	-2.50554574	2.81005803	7.26010299
H	-1.38267430	3.36139942	5.50836602
C	-4.85788757	2.33485506	6.90970576
H	-5.56518597	2.46258638	4.87709137
C	-3.76036125	2.44424401	7.76588782
H	-1.64916798	2.90021112	7.92739883
H	-5.82971142	2.04802025	7.30146666
H	-3.87962047	2.24564094	8.83073085
C	-4.67638657	3.30369406	2.27870932
C	-5.35145876	2.16699085	1.80154643
C	-5.06088110	4.58322271	1.84759361
C	-6.34853783	2.30856566	0.83693566
H	-5.06028433	1.18092302	2.14768113
C	-6.09105629	4.71882576	0.91419009
H	-4.53119047	5.46594235	2.19910481
C	-6.71482917	3.58155634	0.38889574
H	-6.38322275	5.71153380	0.57335127
H	-7.49103953	3.68869347	-0.36872579
H	-0.69091878	1.69826684	3.74003146
H	-6.83514476	1.42010476	0.43841283
H	0.85537746	0.63597761	4.85406362
H	-7.87069299	3.46007904	2.74105192
H	-8.44831381	1.30983223	2.00752505
H	-4.61820618	-2.86022023	-0.06289127
H	0.45036613	-3.42526142	0.99030161
H	-2.01664661	-2.61107186	5.25417162
C	-0.67123240	-0.95597440	4.75905556

C	0.13328744	-1.74867283	3.70418260
C	0.26439128	0.00333162	5.52965648
C	-1.30742641	-1.94428136	5.76222958
H	-0.49645392	-2.50598557	3.21883669
H	0.54558005	-1.09974208	2.92150590
H	0.97225970	-2.27123242	4.18756985
C	-4.05141143	-1.06893638	3.44665889
C	-6.12196268	-3.35799129	1.47077371
C	-5.68270070	-3.12839182	0.00544623
C	-5.18150027	-4.39542032	2.13154919
C	-7.57172695	-3.89307804	1.51618894
H	-6.28375512	-2.33970242	-0.46017082
H	-5.81327069	-4.06121475	-0.56334927
H	-5.47604261	-4.58186044	3.17523780
H	-5.23664486	-5.34681907	1.58263818
H	-4.13714101	-4.05722240	2.11946672
H	-7.62065213	-4.87349011	1.02072587
H	-7.90537288	-4.02398575	2.55715310
H	-8.26494664	-3.21059829	1.01088041
C	-7.11528034	-0.97061157	1.95970124
O	-7.61417949	-0.82058162	0.83316362
N	-7.45663196	-0.20808251	3.03156367
H	-6.86773301	-0.29200484	3.87804530
H	-0.52706543	-2.55650324	6.23809767
H	-1.85646114	-1.40278621	6.54562596
H	-0.31188784	0.65760599	6.20055546
H	0.96989385	-0.57681401	6.14250738
C	-9.91476906	0.18389000	3.07052233
C	-10.12060928	-1.15392628	3.41779185
C	-11.41952719	-1.66542015	3.52809760
C	-12.52320193	-0.84201804	3.28789293
C	-12.32148941	0.49953518	2.93624970
C	-11.02488677	1.00717372	2.83132030
H	-9.26610751	-1.80006007	3.60201220
H	-11.56641762	-2.71068520	3.79988294
H	-13.53402654	-1.24033772	3.37129700
H	-13.17563873	1.14909105	2.74580200
C	-8.31377053	1.83219987	4.08366599
C	-8.48002042	1.46250199	5.42669436
C	-8.33449844	2.40809311	6.44315146
C	-8.01941485	3.73577673	6.12934501
C	-7.85388264	4.10968392	4.79354247
C	-8.00296540	3.16149429	3.77617012
H	-8.74290795	0.43470371	5.67515831
H	-8.46830011	2.10981361	7.48279883
H	-7.89643900	4.47083900	6.92411293
H	-7.60002777	5.13828417	4.53840313
C	-1.17248453	-0.81977015	-0.13942824
C	-0.38469113	0.33032089	-0.71477250
C	-1.84283271	0.32359781	-0.25639437

H	1.65052029	1.67014076	2.50716570
C	-0.14649242	0.38256611	-2.20190374
C	0.57222327	1.12610757	0.15100425
H	2.73305547	0.23031532	2.42090687
C	-2.89799190	1.21096085	-0.04078530
C	-1.14311171	-2.15550459	0.32051430
O	-0.92680143	-0.04688446	-3.03944622
O	1.05930085	0.94338459	-2.49044168
C	1.37871526	1.03411541	-3.91901603
H	2.36494845	1.50092770	-3.95795522
H	0.63082283	1.65167962	-4.42632374
H	1.39877903	0.03240329	-4.35983889
O	0.61390367	2.34800471	0.19353565
O	1.34205098	0.31389383	0.92605564
C	2.23588487	1.01812223	1.85169734
H	2.95927734	1.61525258	1.28649848
O	-2.11988355	-2.63791987	0.96403728
C	0.09068955	-2.98620799	0.04976736
H	-3.86917594	0.71399977	0.06949438
H	-3.66017982	3.21671060	-0.35396448
H	-3.16652826	2.39963721	-1.85332299
H	-1.93122674	3.02237719	-0.73231957
C	-2.91584165	2.53963514	-0.79017557
H	-2.68237820	1.49928544	1.27514806
H	0.89025503	-2.39291994	-0.40790704
H	-0.17621601	-3.81508950	-0.62328005
H	-6.25538045	-2.34361779	3.36438210

TS3-depro-LA1’-RA2-Ha
COSMO(Et_2O)-ZORA-BLYP-D3(BJ)/DZP
E = -17954.83
G = -17353.67
COSMO(Et_2O)-ZORA-M06-2X/TZ2P//COSMO(Et_2O)-ZORA-BLYP-D3(BJ)/DZP
E = -25724.33
G = -25123.17
N_{imag} = 1, 1731 i cm^{-1}

C	-5.33801960	-2.37595403	4.47284124
N	-4.49841717	-1.44272407	3.71563565
H	-4.76529358	-1.26359557	2.74543080
C	-6.92911361	0.19422009	6.83398817
S	-2.86704230	-0.92820675	5.80936165
N	-2.74809625	-0.08410516	3.21300381
H	-3.14049593	-0.24806113	2.27505550
C	-1.96349388	1.16562428	3.28420798
H	-1.57644046	1.27166443	2.26600277
H	-8.61528211	0.05898594	8.96538170
C	-0.73823274	1.10597286	4.20869342
N	0.29294423	0.17486857	3.69045502
H	-1.04651579	0.83487928	5.22531398
P	1.72611631	0.09649340	4.42673513

C	1.66248367	-0.44260462	6.16348473
C	0.59696073	-1.26809507	6.55440414
C	2.65509948	-0.07924801	7.08902908
C	0.52710551	-1.73126342	7.87022291
H	-0.18907223	-1.51616501	5.84465895
C	2.58295824	-0.55302322	8.40076393
H	3.47541666	0.56979261	6.78699112
C	1.52071785	-1.37913032	8.79060122
H	-0.30665579	-2.36217363	8.17648730
H	3.35231148	-0.27543029	9.12048661
H	1.46556671	-1.74267911	9.81636775
C	2.60149514	1.69838452	4.45826992
C	2.06053794	2.74098518	5.23628787
C	3.72291646	1.94678490	3.65053234
C	2.61507576	4.01999640	5.17389430
H	1.21921253	2.55062397	5.89968061
C	4.27894226	3.22737990	3.60029839
H	4.16554050	1.14454690	3.06583973
C	3.71990045	4.26668211	4.35005646
H	2.18796049	4.82391387	5.77214065
H	5.15042830	3.41262188	2.97317631
H	4.15029131	5.26638920	4.30215269
C	2.74508783	-1.09420363	3.51263836
C	2.91724899	-0.93334416	2.12618781
C	3.33497363	-2.18365633	4.17107390
C	3.67493684	-1.85742518	1.40776939
H	2.43112969	-0.11633934	1.60289218
C	4.09015784	-3.10809862	3.44220155
H	3.19862186	-2.31856205	5.24144683
C	4.26121802	-2.94637731	2.06375523
H	4.53964879	-3.95838298	3.95353965
H	4.84697405	-3.67151852	1.49958851
H	-0.32232542	2.12027279	4.22841501
H	3.79528822	-1.73280938	0.33227299
H	-1.29664316	3.88974370	4.04161006
H	-7.31029416	0.78220100	4.21678897
H	-7.95026785	-0.19873643	6.72396169
H	-5.99389005	-2.55495224	1.68830954
H	-3.31580961	-0.29654779	-2.27819015
H	-4.69154627	1.54900370	2.63800814
C	-2.87235617	2.43268386	3.50888826
C	-3.47844146	2.49094445	4.92506355
C	-2.03091091	3.70649902	3.24548725
C	-4.01405201	2.39350492	2.46510920
H	-4.13196214	1.63537491	5.11899325
H	-2.69872906	2.50452751	5.69910727
H	-4.08773908	3.39870194	5.03074929
C	-3.39389798	-0.78251558	4.17820478
C	-5.76340050	-3.60319994	3.61376300
C	-6.57967393	-3.19540382	2.36490201

C	-4.48442067	-4.34099975	3.15599040
C	-6.59112515	-4.55442689	4.50865615
H	-7.49450824	-2.66631406	2.65176845
H	-6.85007478	-4.09783647	1.79641283
H	-3.82594637	-4.56515012	4.00799154
H	-4.75915278	-5.28929601	2.67129323
H	-3.91605145	-3.74443133	2.43385249
H	-6.87024406	-5.45076959	3.93605230
H	-5.99992618	-4.87806353	5.37922655
H	-7.50649871	-4.06862781	4.86509240
C	-6.51032089	-1.57244775	5.12800960
O	-7.69849302	-1.64524481	4.77638239
N	-6.06541944	-0.75743957	6.11755719
H	-5.04397670	-0.71620722	6.27004886
H	-4.61022703	3.31344818	2.53304615
H	-3.60997204	2.31156486	1.44558288
H	-1.49625639	3.63357216	2.28763015
H	-2.69575720	4.58200961	3.20502688
C	-6.56705122	0.21594928	8.30937061
C	-5.23197287	0.34738984	8.72210740
C	-4.91298626	0.40878583	10.08090193
C	-5.92667143	0.34485667	11.04503707
C	-7.25929230	0.21801964	10.64044987
C	-7.57541980	0.15411410	9.27821448
H	-4.43493163	0.41255577	7.98224137
H	-3.87234853	0.51021989	10.38832451
H	-5.67806422	0.39396513	12.10497742
H	-8.05417639	0.16551226	11.38428390
C	-6.89564373	1.58344373	6.17851027
C	-6.66727262	2.75386984	6.91218613
C	-6.66494161	4.00231624	6.27727291
C	-6.89353360	4.09389117	4.90265205
C	-7.12546826	2.92777118	4.16405507
C	-7.12611970	1.68442977	4.79611256
H	-6.48852595	2.69899320	7.98342053
H	-6.47776371	4.90265753	6.86280982
H	-6.88626320	5.06497350	4.40795275
H	-7.29944702	2.98649186	3.09025593
C	-1.70569799	-0.71795549	0.11702147
C	-0.28998805	-0.32152036	-0.23910619
C	-0.76118931	-1.24743646	0.88932870
H	1.98686939	-2.68621060	-2.66358046
C	0.10610581	1.09550172	0.13661986
C	0.41546915	-0.88975854	-1.43755038
H	0.69276781	-3.93619484	-2.55985600
C	-0.23986671	-1.90697229	2.00263783
C	-3.05801303	-0.38040213	-0.14727562
O	-0.69646649	1.99103103	0.35749086
O	1.45063319	1.24396211	0.26102275
C	1.88782297	2.56785327	0.71778976

H	2.97838231	2.51975605	0.71645015
H	1.50929266	2.74841990	1.72856897
H	1.52507617	3.33919656	0.03131059
O	1.01164143	-0.21870846	-2.27257144
O	0.29154846	-2.24317481	-1.49595363
C	0.90739316	-2.87068344	-2.66628588
H	0.46503741	-2.47065905	-3.58448504
O	-3.98623919	-0.69683181	0.65033168
C	-3.34056308	0.39110801	-1.41790395
H	-0.03293619	-0.82386818	2.84478788
H	-1.04015432	-3.91144842	2.31467373
H	-2.16053055	-2.60145075	2.72922975
H	-0.81230253	-2.96846770	3.80918689
H	0.80198050	-2.21648257	1.87824344
C	-1.11299388	-2.90411315	2.75416259
H	-2.57149186	1.15711567	-1.58163313
H	-4.33264043	0.85362897	-1.36217109
H	-4.70859525	-2.74348491	5.29618832

TS3-depro-LA2-RA1-Ha
COSMO(Et_2O)-ZORA-BLYP-D3(BJ)/DZP
E = -17959.63
G = -17360.72
COSMO(Et_2O)-ZORA-M06-2X/TZ2P//COSMO(Et_2O)-ZORA-BLYP-D3(BJ)/DZP
E = -25727.20
G = -25128.29
N_{imag} = 1, 1628 i cm^{-1}

C	-1.96285243	-1.92128265	0.78932574
N	-2.44226644	-1.45400061	2.11788938
H	-1.89639450	-1.90595098	2.87184827
C	-4.84893400	-4.41975414	0.41376783
S	-4.87320974	-0.29319318	1.59037993
N	-3.87195497	-1.27991979	3.87784354
H	-3.14056154	-1.82252653	4.34303430
C	-4.96682794	-0.77282549	4.70691951
H	-5.69849850	-0.35695282	4.00404021
H	-2.99014859	-4.23955513	2.48560896
C	-4.44044290	0.37865635	5.59371511
N	-3.94640473	1.50691916	4.79620980
H	-3.60107222	0.02428096	6.20286598
P	-4.85923805	2.76251279	4.37503325
C	-5.28865161	3.82658886	5.78938289
C	-4.46369794	3.77451417	6.92458551
C	-6.38492095	4.70403926	5.74748359
C	-4.73579442	4.60768042	8.01340744
H	-3.62343142	3.08377547	6.95363646
C	-6.64611791	5.53690586	6.83780022
H	-7.02860884	4.73418953	4.86978697
C	-5.82176253	5.48944567	7.96930426
H	-4.09865524	4.56767672	8.89639950

H	-7.49312822	6.22134004	6.80688912
H	-6.02987364	6.13892661	8.81915944
C	-6.46030659	2.35527091	3.59673598
C	-6.68260948	2.50258806	2.21889365
C	-7.43133881	1.71946311	4.39199834
C	-7.84062855	1.97991078	1.63850269
H	-5.94996021	3.00641609	1.59528825
C	-8.58577729	1.19982629	3.80760775
H	-7.28563350	1.63224128	5.46672170
C	-8.78374731	1.31555514	2.42636646
H	-7.99405197	2.07538500	0.56430250
H	-9.33064188	0.70278988	4.42780008
H	-9.67811866	0.89489841	1.96812558
C	-3.86360877	3.72907328	3.21235934
C	-3.49754783	5.05176515	3.50247118
C	-3.41135129	3.10868452	2.03440857
C	-2.67509294	5.75092988	2.61434661
H	-3.83885986	5.52809008	4.41890305
C	-2.59365620	3.81609884	1.15357305
H	-3.69053860	2.07705290	1.82243442
C	-2.22414364	5.13519797	1.44235203
H	-2.23868068	3.33507936	0.24420461
H	-1.57814362	5.68147774	0.75551758
H	-5.21866478	0.69722323	6.29959881
H	-2.38148425	6.77476868	2.84310846
H	-6.35569453	-0.83058174	7.26759601
H	-6.13685499	-5.21568954	-1.87268486
H	-4.39396272	-5.11780089	-0.30430562
H	-3.78697670	-0.26633834	-0.75711843
H	0.30095161	-2.79931451	7.14987221
H	-5.57298949	-3.24967100	3.73367059
C	-5.65091801	-1.94319169	5.49041582
C	-4.62289777	-2.72662871	6.34071869
C	-6.76077683	-1.39137616	6.41422952
C	-6.30585142	-2.88555179	4.45784415
H	-3.87779265	-3.23089799	5.71428216
H	-4.09660675	-2.07017760	7.04878505
H	-5.13689878	-3.50549477	6.92293248
C	-3.67309068	-1.04096268	2.55612772
C	-1.71867091	-0.86333381	-0.35777743
C	-2.92325452	-0.66012488	-1.30127940
C	-1.31540452	0.47798998	0.27998053
C	-0.53691788	-1.40640709	-1.20092217
H	-3.21517901	-1.60533406	-1.78007946
H	-2.64155880	0.04908979	-2.09424739
H	-0.45576205	0.35758658	0.95503796
H	-1.03861070	1.19520320	-0.50666008
H	-2.14872726	0.89267923	0.85392934
H	-0.32667939	-0.71822955	-2.03228928
H	0.37318200	-1.49853765	-0.58916289

H	-0.77131726	-2.39546310	-1.61709871
C	-2.67751170	-3.21145371	0.30855361
O	-2.01821847	-4.18526351	-0.09729776
N	-4.02627780	-3.20419691	0.41295103
H	-4.48318221	-2.33149660	0.71404670
H	-6.74745790	-3.75951608	4.95635582
H	-7.09996567	-2.35634806	3.91144090
H	-7.44486223	-0.73639677	5.85836228
H	-7.34985433	-2.22838513	6.81705067
C	-4.85384643	-5.10155319	1.79487104
C	-5.90694115	-5.95920504	2.14642267
C	-5.92180359	-6.59898289	3.38952699
C	-4.87979430	-6.39342979	4.30043563
C	-3.82389310	-5.54678529	3.95467863
C	-3.81302041	-4.90644294	2.71262811
H	-6.73189747	-6.11258202	1.45238114
H	-6.75563679	-7.25130410	3.64951676
H	-4.89813119	-6.88173386	5.27459833
H	-3.00788479	-5.35748722	4.65029789
C	-6.24517153	-4.04680823	-0.06321217
C	-7.03608818	-3.17684870	0.70266202
C	-8.30206738	-2.79418611	0.25766985
C	-8.80155587	-3.29259500	-0.95287963
C	-8.02189059	-4.16801073	-1.71586319
C	-6.74558825	-4.53852147	-1.27324604
H	-6.65594772	-2.78391048	1.64079367
H	-8.89892379	-2.10600045	0.85588515
H	-9.79258354	-2.99959847	-1.29910373
H	-8.40401770	-4.56067604	-2.65804186
C	-0.90723036	-1.02067523	5.56363318
C	-0.46566593	0.08920978	6.47630806
C	-0.98223622	0.20949083	5.04119997
H	-3.48780574	0.17575215	9.17374483
C	0.97777121	0.36504400	6.77535126
C	-1.39529832	0.56633162	7.57839960
H	-2.54106869	-0.93349008	10.23617083
C	-1.32759897	1.20154220	4.13043520
C	-1.00058344	-2.42322346	5.45663406
O	1.35852957	1.00190505	7.75319506
O	1.80141181	-0.16632548	5.83666978
C	3.22679053	0.10304196	6.04258148
H	3.72765413	-0.37259486	5.19685127
H	3.55545822	-0.33465093	6.99078152
H	3.40549999	1.18322141	6.04931809
O	-1.96242860	1.64868779	7.59856397
O	-1.57223935	-0.40326016	8.51626655
C	-2.50278645	-0.05098962	9.59446906
H	-2.12525494	0.81952246	10.14090959
O	-1.47772319	-2.98600304	4.42346072
C	-0.56833599	-3.24537350	6.64995620

H	-1.45059058	0.84536788	3.10297450
H	-1.02767140	3.25776236	3.50308382
H	0.39729130	2.53872868	4.28477822
H	-1.00672305	3.02482481	5.26539691
C	-0.70276094	2.58631996	4.30469667
H	-2.71392950	1.37525407	4.45128786
H	-0.34529294	-4.27121166	6.33451007
H	-1.38943660	-3.26990911	7.38345435
H	-0.95895019	-2.28387022	1.04362605

TS3-depro-LA1’-RA1-Hb
COSMO(Et_2O)-ZORA-BLYP-D3(BJ)/DZP
E = -17967.81
G = -17369.81
COSMO(Et_2O)-ZORA-M06-2X/TZ2P//COSMO(Et_2O)-ZORA-BLYP-D3(BJ)/DZP
E = -25730.71
G = -25132.71
N_{imag} = 1, 1692 i cm^{-1}

C	-5.96588536	-2.50061860	2.46997521
N	-4.69027824	-1.85399281	2.77941306
H	-3.88194512	-2.07617187	2.18702384
C	-8.49089474	0.22907763	1.63043003
S	-5.67453406	-0.85288825	5.09134245
N	-3.15910949	-0.74182828	4.06139640
H	-2.50792732	-1.04156419	3.32087831
C	-2.60609414	-0.11840311	5.25858421
H	-3.44558632	-0.05236464	5.95943977
H	-9.55790330	-1.46074452	-0.20422496
C	-2.08275865	1.34150067	4.99172214
N	-2.55187438	1.90406818	3.72607172
H	-0.98470833	1.33160779	4.95781911
P	-3.47895045	3.20238966	3.51994405
C	-2.49533064	4.69226741	3.12824384
C	-2.44923228	5.24214928	1.84105995
C	-1.64531948	5.19464630	4.13166524
C	-1.56718087	6.28849657	1.55742731
H	-3.08647478	4.84341118	1.06031926
C	-0.77351739	6.24449583	3.84730450
H	-1.66123165	4.76069680	5.13100774
C	-0.73123847	6.79124644	2.55776959
H	-1.52799521	6.70592421	0.55185460
H	-0.11871972	6.63152505	4.62737472
H	-0.04233765	7.60532661	2.33478383
C	-4.48775753	3.63793569	4.97101408
C	-4.73697703	4.97635555	5.31877693
C	-5.06499774	2.59926443	5.71920225
C	-5.55193834	5.26688550	6.41628156
H	-4.30249466	5.78736570	4.73866132
C	-5.86750356	2.89794893	6.82021489
H	-4.93090224	1.55896676	5.43144939

C	-6.11007744	4.23027563	7.17290132
H	-5.74973297	6.30521000	6.68050582
H	-6.31417707	2.08535453	7.39253113
H	-6.74179067	4.46206791	8.02992354
C	-4.58023583	2.89506352	2.11355365
C	-4.57175432	1.63150811	1.50487436
C	-5.46856710	3.89074642	1.67111382
C	-5.43289065	1.37462979	0.43801966
H	-3.89497347	0.86655167	1.87375672
C	-6.32762686	3.62364789	0.60774525
H	-5.50124563	4.86091097	2.16425655
C	-6.30624367	2.36806984	-0.00865896
H	-7.02906546	4.38589649	0.27423294
H	-6.98420019	2.16135201	-0.83633729
H	-2.35848896	1.96450256	5.85343044
H	-5.43186677	0.39522132	-0.03324359
H	-0.52942683	0.56039585	7.05359786
H	-10.03143261	2.22940466	0.78247473
H	-8.19502644	0.29971637	0.57561867
H	-3.86796648	-3.75581140	0.94007461
H	-0.17102864	3.48877848	1.04066908
H	-2.52749544	-2.91859423	5.34609672
C	-1.53382375	-1.04690731	5.92238965
C	-0.32813531	-1.29660330	4.98749375
C	-1.05447492	-0.38727232	7.23568715
C	-2.20173733	-2.40062452	6.25742594
H	-0.63632527	-1.74422920	4.03246105
H	0.22314241	-0.37204564	4.77048120
H	0.37469433	-1.99209417	5.46991988
C	-4.43938931	-1.16294462	3.92478054
C	-5.78880744	-3.96371967	1.97075547
C	-4.89707375	-4.06067357	0.71467782
C	-5.14021797	-4.77156011	3.11992768
C	-7.19461560	-4.53573668	1.67715816
H	-5.28638965	-3.42928367	-0.09139705
H	-4.86057784	-5.10499345	0.36919027
H	-5.76934096	-4.74828076	4.02203589
H	-5.01213225	-5.81986062	2.81352969
H	-4.15248781	-4.36839323	3.38082158
H	-7.11655174	-5.60267676	1.42298448
H	-7.84758608	-4.44155501	2.55831421
H	-7.66841138	-4.01151950	0.83807703
C	-6.79996031	-1.57443531	1.54708353
O	-6.82740498	-1.65271646	0.30917564
N	-7.48879895	-0.63875536	2.25515661
H	-7.39089889	-0.65454436	3.27642310
H	-1.48988772	-3.05084317	6.78671048
H	-3.08403761	-2.25386582	6.89653000
H	-1.90515968	-0.18771801	7.90495184
H	-0.35876455	-1.05884488	7.75975910

C	-9.85108228	-0.47145099	1.68717359
C	-10.69831807	-0.32268031	2.79331250
C	-11.90259412	-1.02937661	2.86121382
C	-12.27295954	-1.88897780	1.81945818
C	-11.43025223	-2.03957277	0.71233980
C	-10.22459089	-1.33382165	0.64749701
H	-10.41984332	0.35651651	3.59811735
H	-12.55553109	-0.90589212	3.72526218
H	-13.21453083	-2.43540553	1.86961598
H	-11.71333406	-2.70557648	-0.10277614
C	-8.53159211	1.63242141	2.21574454
C	-7.74888881	2.04681784	3.29673459
C	-7.85858915	3.35652691	3.78190545
C	-8.73708970	4.26313017	3.18874001
C	-9.51753552	3.85542718	2.09929688
C	-9.41694115	2.54914810	1.62446268
H	-7.02968361	1.37051627	3.75308234
H	-7.24526765	3.67118004	4.61955492
H	-8.81029624	5.28283776	3.56690091
H	-10.20230545	4.55598653	1.62099034
C	-1.23370422	-0.25739383	0.60888072
C	-0.92950030	0.85923253	-0.35286369
C	-0.73403015	0.84646462	1.16026158
H	1.58908789	0.37071142	-3.50530821
C	-2.09881675	1.59033047	-0.98044074
C	0.32432224	0.78256413	-1.18520144
H	1.04181297	1.96663641	-4.14004127
C	-0.31827130	1.67241190	2.20600282
C	-1.72124881	-1.57906406	0.66130896
O	-2.99552326	1.04611106	-1.60676296
O	-2.02402878	2.93010984	-0.75624517
C	-3.07493171	3.72025931	-1.40445777
H	-3.08154634	3.51644645	-2.48083012
H	-2.80906168	4.76142757	-1.20973596
H	-4.04567386	3.47144877	-0.97083705
O	1.34337275	0.19781252	-0.84509712
O	0.17535420	1.44029999	-2.36801335
C	1.34401365	1.40719409	-3.25236812
H	2.19716077	1.88330536	-2.75846730
O	-1.98551553	-2.15065274	1.76221719
C	-1.94575024	-2.26906866	-0.66726179
C	0.34961911	3.00121685	1.87380698
H	1.40550737	2.86564932	1.58943328
H	0.31303340	3.68093675	2.73463692
H	-6.50741111	-2.56000479	3.42239361
H	0.13871461	1.11187468	3.03323514
H	-1.52759803	1.85136498	2.85205243
H	-1.02736024	-2.22361768	-1.27152683
H	-2.72704175	-1.73774744	-1.22985184
H	-2.23833932	-3.31196658	-0.51559808

TS3-depro-LA2-RA1-Hb

COSMO(Et_2O)-ZORA-BLYP-D3(BJ)/DZP

E = -17953.88

G = -17352.76

COSMO(Et_2O)-ZORA-M06-2X/TZ2P//COSMO(Et_2O)-ZORA-BLYP-D3(BJ)/DZP

E = -25719.11

G = -25117.99

N_{imag} = 1, 1698 i cm^{-1}

C	-5.09038000	-2.99860200	2.53448100
N	-3.86589100	-2.18342600	2.69996800
H	-3.31435300	-2.14491000	1.83142600
C	-4.95613100	-5.29364700	5.59987900
S	-4.09530400	-1.21470000	5.24642300
N	-2.33109500	-0.60630900	3.30334300
H	-2.00911400	-0.73030400	2.33563900
C	-1.42365800	0.05647800	4.23530500
H	-1.99878800	0.16854600	5.16218600
H	-5.02947100	-6.67632400	7.93187400
C	-1.01813800	1.48570300	3.73443300
N	-1.86830700	2.03557200	2.67010100
H	-0.00733500	1.44832000	3.31366900
P	-3.17338700	2.93186600	2.97553800
C	-2.84073000	4.73037400	3.02954100
C	-1.86234300	5.23080400	2.15384200
C	-3.56581500	5.61127600	3.84690500
C	-1.61650800	6.60274500	2.09157200
H	-1.30228400	4.54740300	1.52291500
C	-3.31356900	6.98528300	3.78223500
H	-4.32035800	5.22880600	4.53156800
C	-2.34278900	7.48127400	2.90524700
H	-0.85835900	6.98752100	1.41017000
H	-3.87467500	7.66816300	4.41917200
H	-2.14925000	8.55241900	2.85782700
C	-4.03594200	2.53937500	4.52792000
C	-3.40271400	2.79253800	5.75910600
C	-5.31284600	1.95955100	4.50781500
C	-4.04286000	2.45573700	6.95074300
H	-2.42306600	3.26524600	5.78966000
C	-5.95170900	1.63537400	5.70535600
H	-5.80619000	1.75614400	3.56319500
C	-5.31870200	1.87900400	6.92517700
H	-3.54818400	2.64860100	7.90206300
H	-6.93667500	1.17211000	5.67963000
H	-5.81361000	1.61511400	7.85920100
C	-4.37440600	2.71730300	1.63301600
C	-4.25986700	1.62229200	0.76956700
C	-5.45477700	3.60752100	1.51007100
C	-5.20935700	1.43211200	-0.23461800
H	-3.43180400	0.93482900	0.89633800

C	-6.42533700	3.38376700	0.53324000
H	-5.53964400	4.46778200	2.17168800
C	-6.29781700	2.30029800	-0.34491000
H	-7.27070300	4.06508900	0.44369100
H	-7.04452600	2.13928600	-1.12188500
H	-0.94605900	2.14513100	4.60894500
H	-5.10560800	0.60271300	-0.92939600
H	1.11619100	0.84492700	5.22394700
H	-5.46684400	-3.34546700	7.44161200
H	-5.07489900	-6.22883800	5.03453400
H	-6.57900100	-1.73934400	4.68185800
H	-2.68915300	-2.27997100	-2.17032000
H	-1.20438400	-2.74919300	4.41036100
C	-0.16307500	-0.82805300	4.55576700
C	0.67232700	-1.10982000	3.28583100
C	0.70083400	-0.09643900	5.60891300
C	-0.62114700	-2.17908300	5.14436100
H	0.06289500	-1.57544900	2.49845500
H	1.12741400	-0.19993500	2.87352400
H	1.49218100	-1.80175800	3.52886100
C	-3.41317800	-1.35004100	3.68152200
C	-6.49386000	-2.24480800	2.54825200
C	-7.19082500	-2.24088800	3.92720600
C	-6.27557400	-0.80646500	2.05458400
C	-7.41317900	-2.98285400	1.54399300
H	-7.40324300	-3.25965600	4.27464700
H	-8.15226100	-1.71238600	3.83700100
H	-5.78483800	-0.80244200	1.07451500
H	-7.24138100	-0.29123200	1.94936200
H	-5.65124700	-0.23597400	2.74823300
H	-8.40215500	-2.50125500	1.52655400
H	-6.99462000	-2.94608500	0.52637800
H	-7.53776200	-4.03536000	1.82746500
C	-5.14972800	-4.30521000	3.35725600
O	-5.67167400	-5.32008900	2.85985400
N	-4.64341900	-4.25074100	4.61169700
H	-4.45082300	-3.29991200	4.98845500
H	0.25697600	-2.78097600	5.42077500
H	-1.24660500	-2.03620700	6.03360600
H	0.10745300	0.12960200	6.50802500
H	1.54494800	-0.73434800	5.91011800
C	-3.82639800	-5.46463000	6.60860200
C	-2.56779300	-4.88533200	6.41311100
C	-1.54748900	-5.06350000	7.35466300
C	-1.77368400	-5.83145900	8.50048300
C	-3.02946300	-6.42083500	8.69868400
C	-4.04829400	-6.23404700	7.76183700
H	-2.38651900	-4.28663600	5.52720200
H	-0.57730800	-4.59610700	7.19116300
H	-0.98108700	-5.96922400	9.23567200

H	-3.21694500	-7.02065700	9.58929800
C	-6.29174400	-4.94514700	6.25045100
C	-7.45994000	-5.61345900	5.86984600
C	-8.70178700	-5.21199600	6.37691600
C	-8.78025000	-4.13452000	7.26428100
C	-7.61090000	-3.46558600	7.65046700
C	-6.37436600	-3.87223800	7.14921700
H	-7.39822000	-6.43452700	5.15640700
H	-9.60768200	-5.73565800	6.07134700
H	-9.74686400	-3.81562200	7.65422700
H	-7.66682600	-2.62337300	8.34013800
C	-1.52463600	0.26316400	-0.80997800
C	-1.32770300	1.43912400	-1.73378000
C	-0.74065900	1.23236800	-0.34148000
H	0.26362000	1.38753600	-5.48395000
C	-2.44952300	2.43418700	-1.93777600
C	-0.36845000	1.28038200	-2.89098500
H	0.25542900	3.18999400	-5.45951700
C	-0.04153900	1.90305200	0.66431800
C	-2.24273300	-0.92947000	-0.57366900
O	-3.34069500	2.30688000	-2.76511300
O	-2.35058900	3.47405200	-1.06650200
C	-3.39526400	4.49500800	-1.18427000
H	-3.27389800	5.12908400	-0.30433400
H	-4.37862600	4.02393600	-1.19534400
H	-3.23868700	5.06677200	-2.10624400
O	0.40805900	0.34476600	-3.01824400
O	-0.44790500	2.33588700	-3.74364700
C	0.46658300	2.28344100	-4.88884000
H	1.50270000	2.27204900	-4.53574100
O	-2.11645500	-1.55636300	0.51958200
C	-3.17149100	-1.43506900	-1.65351000
C	0.70701600	3.18777200	0.33206600
H	0.94717600	3.74385800	1.24921700
H	0.09666300	3.83133500	-0.31452100
H	1.65586900	2.98277500	-0.18915200
H	0.50206000	1.21366400	1.32245300
H	-1.09679900	2.10851200	1.55369100
H	-3.39766800	-0.65861500	-2.39386700
H	-4.09825700	-1.81175600	-1.20088700
H	-4.97620100	-3.36089400	1.50505900

TS3-depro-LA2-RA2-Hb
COSMO(Et_2O)-ZORA-BLYP-D3(BJ)/DZP
E = -17956.17
G = -17357.16
COSMO(Et_2O)-ZORA-M06-2X/TZ2P//COSMO(Et_2O)-ZORA-BLYP-D3(BJ)/DZP
E = -25721.78
G = -25122.77
N_{imag} = 1, 1723 i cm^{-1}

C	-5.48436351	0.70805215	2.04321719
N	-4.30430266	0.95300673	2.89038136
H	-4.20245264	1.95019734	3.12738269
C	-3.83001805	-1.36954029	-0.75211137
S	-3.46863262	-1.56914253	3.49435242
N	-2.81664130	0.85654554	4.57384983
H	-3.08450452	1.84758674	4.59393345
C	-1.63224419	0.58040244	5.41276754
H	-1.03242246	1.49753579	5.32446764
H	-2.41485135	0.01303413	-2.62365092
C	-0.74488921	-0.56364236	4.91534326
N	-0.01702269	-0.20147964	3.67443942
H	-1.34555342	-1.46512733	4.77191289
P	1.38151377	-0.93264410	3.36859163
C	1.24783113	-2.64984648	2.76784634
C	-0.04064329	-3.20604139	2.71775757
C	2.35775657	-3.41057124	2.35956877
C	-0.21404939	-4.51990337	2.27659269
H	-0.90363077	-2.60151384	2.98954411
C	2.17473149	-4.71850529	1.90675533
H	3.35876734	-2.98201315	2.38069871
C	0.88987869	-5.27541587	1.86988310
H	-1.21495999	-4.94827514	2.23539841
H	3.03362838	-5.30285175	1.57843052
H	0.75086646	-6.29584968	1.51431106
C	2.39301360	-0.96489552	4.89872384
C	3.05948312	-2.10970234	5.36018524
C	2.41916273	0.21533500	5.66897030
C	3.77159217	-2.06628893	6.56373313
H	3.01109872	-3.04034401	4.80177811
C	3.13033482	0.24994674	6.86753871
H	1.84873567	1.08632693	5.35521442
C	3.81362909	-0.88777348	7.31441825
H	4.28552949	-2.95948669	6.91729863
H	3.13766632	1.16192644	7.46340535
H	4.36495479	-0.85927169	8.25373889
C	2.27931904	0.02708641	2.11171667
C	2.60922228	1.35971383	2.41440772
C	2.58947605	-0.49494817	0.84868480
C	3.22331036	2.16620973	1.45948349
H	2.34961945	1.76854522	3.38190669
C	3.21718393	0.31309461	-0.10243890
H	2.31658798	-1.51219923	0.59246352
C	3.53185920	1.64093542	0.19901559
H	3.44231055	-0.09163514	-1.08805851
H	4.00245223	2.27373041	-0.55283233
H	-0.02254295	-0.76510741	5.71380110
H	3.42232889	3.21126080	1.68515901
H	-0.10311306	-0.34619216	7.66466190
H	-4.43753682	-2.82878198	-2.94566035

H	-4.30430550	-0.76641410	-1.53645332
H	-8.04603930	0.64261419	1.06290881
H	-2.07058857	6.25314951	3.71314335
H	-3.90355333	1.59581109	6.76614801
C	-2.03413062	0.44519558	6.92526282
C	-2.78800544	-0.87471871	7.18497205
C	-0.75886520	0.52362719	7.79800457
C	-2.94633452	1.63709263	7.30153181
H	-3.66288480	-0.95649569	6.52640778
H	-2.14655500	-1.74825774	7.00402669
H	-3.13113576	-0.91492779	8.22985467
C	-3.53316506	0.13929448	3.67879662
C	-6.76280778	0.06120209	2.74101324
C	-8.00926882	0.76627612	2.15246963
C	-6.88962630	-1.45515438	2.47993377
C	-6.70747265	0.33171041	4.25920514
H	-7.99200379	1.84326603	2.37990214
H	-8.92211893	0.33602426	2.59043624
H	-6.04152229	-1.99725605	2.90867241
H	-7.81856833	-1.82619061	2.93919033
H	-6.93623154	-1.67225069	1.40435590
H	-7.65543043	0.01279834	4.71706865
H	-5.89246165	-0.22381046	4.73815420
H	-6.56850554	1.40230166	4.46898293
C	-5.20292992	0.10626112	0.64600208
O	-5.97980260	0.36729019	-0.29059013
N	-4.09294184	-0.66029577	0.50541055
H	-3.63985847	-1.00239895	1.36931149
H	-3.15870300	1.61873170	8.38027059
H	-2.46089275	2.59618245	7.06396593
H	-0.17762464	1.42662686	7.55934253
H	-1.03925611	0.56746982	8.86076224
C	-2.33348797	-1.44917764	-1.04125218
C	-1.50589982	-2.32650555	-0.32650399
C	-0.15037655	-2.43621044	-0.64130844
C	0.39386991	-1.67046158	-1.67863957
C	-0.42153656	-0.78530399	-2.38810497
C	-1.77869269	-0.67615187	-2.06795462
H	-1.92792607	-2.94079185	0.46488804
H	0.47788965	-3.12706476	-0.08453987
H	1.44902456	-1.76308864	-1.93130131
H	-0.00258324	-0.17781792	-3.18994681
C	-4.45724139	-2.76359998	-0.78752427
C	-4.75831551	-3.47160340	0.38093975
C	-5.27476060	-4.76996466	0.30822980
C	-5.49593772	-5.37253469	-0.93406025
C	-5.19685858	-4.66732655	-2.10719532
C	-4.67820414	-3.37226560	-2.03145018
H	-4.59948546	-3.00945825	1.35104932
H	-5.50828421	-5.30899057	1.22656528

H	-5.90203546	-6.38224314	-0.99068261
H	-5.37104865	-5.12623080	-3.08036200
C	-1.70862297	3.63872628	3.15836976
C	-0.29200146	3.88213847	2.69068187
C	-1.21212873	2.66601918	2.40767844
H	-0.70473774	5.55254242	-0.94620449
C	0.75798619	3.72479176	3.75873579
C	0.00393931	4.74737533	1.49002252
H	-1.93180319	6.68944674	-0.27492536
C	-1.15840988	1.43484302	1.75613821
C	-2.73650178	4.21150806	3.93604372
O	0.62310046	3.00296539	4.74068933
O	1.84968168	4.50999312	3.55947370
C	2.85624315	4.44270473	4.62182073
H	3.61489680	5.17475593	4.33629210
H	3.28793124	3.43778161	4.66943766
H	2.40366147	4.69522114	5.58618744
O	1.06644873	4.78205898	0.88657787
O	-1.10606852	5.44810988	1.11764355
C	-0.95821961	6.22059943	-0.11632683
H	-0.17392548	6.97506604	0.00520566
O	-3.80899752	3.58332221	4.19131699
C	-2.49353814	5.59867426	4.48733892
H	-0.58813389	0.63193031	2.75012472
H	-0.03695855	0.24152961	0.33491281
H	0.63723260	1.86651226	0.59760897
H	-0.83182328	1.64735092	-0.37742476
C	-0.28947603	1.29053210	0.51105600
H	-2.14383209	0.96888008	1.65613042
H	-1.75991192	5.54112427	5.30722080
H	-3.42873648	6.01837334	4.87469337
H	-5.79032188	1.73217541	1.79181214

TS4-repro-LA1’-RA2-R
COSMO(Et_2O)-ZORA-BLYP-D3(BJ)/DZP
E = -17950.85
G = -17350.35
COSMO(Et_2O)-ZORA-M06-2X/TZ2P//COSMO(Et_2O)-ZORA-BLYP-D3(BJ)/DZP
E = -25725.32
G = -25124.82
N_{imag} = 1, 1550 i cm^{-1}

C	-4.90803771	-1.77498326	5.28436396
N	-4.51175838	-0.68455606	4.39337604
H	-5.05165783	-0.58352300	3.53420307
C	-6.49372519	0.46823166	7.89607073
S	-2.57234064	0.17910492	6.05827931
N	-3.35499448	1.10633116	3.60744789
H	-3.83317648	0.83087574	2.73890439
C	-2.19388213	1.97195603	3.30021512
H	-2.27355705	2.09524401	2.21588004

H	-8.22110378	1.37302355	9.75539736
C	-0.83526702	1.25459969	3.51360504
N	-0.09681422	1.19106313	2.22880397
H	-1.02374926	0.24424358	3.88864528
P	1.51294788	1.28842444	2.18769604
C	2.13315025	2.78962952	3.02449941
C	2.26618216	2.76598261	4.42601598
C	2.34155639	3.99147652	2.32832658
C	2.57088870	3.93840352	5.11967000
H	2.13633141	1.83444628	4.97356529
C	2.65870403	5.15756896	3.02763294
H	2.26498525	4.02055252	1.24472629
C	2.76323519	5.13593845	4.42260241
H	2.66069239	3.91515374	6.20512415
H	2.82525838	6.08517782	2.48092403
H	3.00211693	6.04983991	4.96543036
C	1.95884008	1.37510470	0.43489955
C	1.27111840	2.30311044	-0.36831143
C	2.97733624	0.58967411	-0.11986458
C	1.62871526	2.46324862	-1.70590100
H	0.45716379	2.88547388	0.05585057
C	3.31206693	0.73737417	-1.46795195
H	3.50520163	-0.13797270	0.48840558
C	2.64916608	1.68086214	-2.25710498
H	1.09813008	3.18665346	-2.32389424
H	4.09362500	0.11381765	-1.90003151
H	2.91694980	1.79708057	-3.30673859
C	2.41841945	-0.03848681	3.04853267
C	1.68453524	-1.05817347	3.67260255
C	3.81708101	0.00197676	3.17781284
C	2.34688692	-2.03773270	4.41512715
H	0.60575757	-1.09489946	3.55907112
C	4.47629387	-0.99565945	3.89804812
H	4.38719289	0.81359217	2.72742897
C	3.74076723	-2.01286481	4.51985095
H	5.56200640	-0.97380405	3.98559306
H	4.25716781	-2.78391578	5.09079092
H	-0.24632181	1.76221405	4.28615118
H	1.77510567	-2.82668336	4.90231205
H	-0.18309411	3.95409702	3.78836640
H	-7.19110667	1.43664538	5.47910678
H	-7.51599768	0.17007164	7.62305541
H	-5.89473221	-2.24558725	2.61840739
H	-3.05285451	1.62518432	-1.43288638
H	-4.49517356	3.42986950	3.56826625
C	-2.32403837	3.43357721	3.84301637
C	-2.36971474	3.51328454	5.37937439
C	-1.12423417	4.25379771	3.31538361
C	-3.62821160	4.02610271	3.26145103
H	-3.23641517	2.97912735	5.78097819

H	-1.46690429	3.08032609	5.82962366
H	-2.44192214	4.56757839	5.68722616
C	-3.50786959	0.21290150	4.62003447
C	-5.14826843	-3.09857271	4.50653247
C	-6.21700281	-2.94884180	3.40028974
C	-3.80115303	-3.51266096	3.86842454
C	-5.58582706	-4.17693474	5.52275332
H	-7.16819625	-2.60410397	3.82139816
H	-6.37477865	-3.91932783	2.90793308
H	-3.02177206	-3.61877810	4.63794794
H	-3.91086498	-4.48063081	3.35983130
H	-3.45958357	-2.77942620	3.12707146
H	-5.65747358	-5.15297082	5.02173283
H	-4.84880780	-4.26719535	6.33552274
H	-6.56258155	-3.93645090	5.96065389
C	-6.07620159	-1.24879979	6.16086493
O	-7.27684827	-1.46855780	5.94356104
N	-5.61220950	-0.45789870	7.17265068
H	-4.61585817	-0.18918149	7.10776833
H	-3.77372936	5.05379051	3.62372042
H	-3.58987630	4.05102803	2.16137974
H	-1.00813799	4.14075647	2.22774953
H	-1.28119489	5.32011930	3.53475657
C	-6.35092126	0.35309989	9.40543749
C	-5.23387891	-0.23573199	10.00826565
C	-5.11664576	-0.27155756	11.40355044
C	-6.11735835	0.28047954	12.20836889
C	-7.23992302	0.86807878	11.61048862
C	-7.35230351	0.90439898	10.21899108
H	-4.45290570	-0.67556558	9.39154942
H	-4.24114883	-0.73371968	11.85964359
H	-6.02646765	0.25235118	13.29396673
H	-8.02768707	1.29645854	12.22984234
C	-6.24777010	1.88541985	7.36841315
C	-5.56777801	2.85693181	8.11146053
C	-5.32876838	4.12446278	7.56749228
C	-5.76971470	4.43152927	6.27875200
C	-6.44391692	3.46141334	5.52820256
C	-6.67623152	2.19578510	6.06764021
H	-5.22456765	2.62760305	9.11834779
H	-4.79053792	4.86946130	8.15324936
H	-5.57777832	5.41579524	5.85363857
H	-6.78584946	3.69133439	4.51970591
C	-1.48631229	-0.14360181	0.27573322
C	-1.37212691	-1.66023100	-0.06892514
C	-0.89403606	-0.53023417	-0.95551432
H	2.21780107	-3.23288394	0.06502782
C	-2.68250996	-2.30333791	-0.45969271
C	-0.30394890	-2.58171968	0.42933588
H	2.77597585	-2.12149890	1.36659002

C	-0.29237379	-0.35511742	-2.12318125
C	-2.79197922	0.50640278	0.36253479
O	-3.31067510	-2.02570910	-1.47362188
O	-3.11494501	-3.16780068	0.48986331
C	-4.41251912	-3.78055839	0.21587242
H	-4.58371545	-4.46379286	1.04862489
H	-4.38108168	-4.32138978	-0.73639993
H	-5.18696001	-3.00730016	0.18201701
O	-0.34531586	-3.80076719	0.30739282
O	0.79956528	-1.92054015	0.88862265
C	1.97544162	-2.77977031	1.03267634
H	1.79099035	-3.56110757	1.77604990
O	-3.75470963	0.06065596	1.02322431
C	-2.88708845	1.83675134	-0.36363031
H	-0.68655877	0.48452304	1.30564836
H	-0.08503595	-2.45508361	-2.57662665
H	1.25607892	-1.43444444	-3.16220842
H	-0.30125409	-1.41890461	-4.00784792
C	0.16501542	-1.47847371	-3.01114681
H	-0.09542257	0.66263701	-2.46004091
H	-1.94946524	2.40260548	-0.29061373
H	-3.72619085	2.42918070	0.01933011
H	-4.05025780	-1.93831073	5.95026029

TS4-repro-LA2-RA1-R (TS4-R)

COSMO(Et_2O)-ZORA-BLYP-D3(BJ)/DZP

E = -17961.08

G = -17361.27

COSMO(Et_2O)-ZORA-M06-2X/TZ2P//COSMO(Et_2O)-ZORA-BLYP-D3(BJ)/DZP

E = -25730.78

G = -25130.97

N_{imag} = 1, 1630 i cm^{-1}

C	-3.96729462	-3.34810220	0.66117807
N	-4.29402677	-2.88291994	2.03231293
H	-3.89355664	-3.51498002	2.73842850
C	-7.49233988	-4.67287548	-0.15408875
S	-6.14953492	-0.93500428	1.59184430
N	-5.62848213	-2.37285823	3.81403139
H	-5.02937798	-3.04441417	4.30753532
C	-6.54604530	-1.58560351	4.63705664
H	-6.85509610	-0.74529906	4.01111432
H	-6.15662414	-5.67850104	2.01132459
C	-5.78285698	-1.04706000	5.86468308
N	-4.50943550	-0.39546473	5.52912812
H	-5.52092154	-1.88248195	6.52323456
P	-4.30838702	1.15753897	5.15614317
C	-5.88566900	2.05497958	5.09732714
C	-6.79473721	1.81379080	4.05051763
C	-6.24464550	2.90676468	6.15635869
C	-8.05025107	2.42101265	4.06948969

H	-6.53096371	1.14543469	3.23222414
C	-7.50716868	3.50629879	6.16819982
H	-5.54340471	3.10027209	6.96519532
C	-8.40979879	3.26422689	5.12735269
H	-8.75099321	2.23116850	3.25705105
H	-7.78347511	4.16401976	6.99145484
H	-9.39302559	3.73355420	5.13997096
C	-3.38810420	1.42494988	3.60608085
C	-2.10885857	0.84609886	3.52732600
C	-3.84207970	2.25449880	2.57250048
C	-1.29417809	1.09522500	2.42591618
H	-1.74867867	0.22277425	4.33960091
C	-3.02081864	2.49653685	1.46647982
H	-4.81931867	2.72573610	2.62967616
C	-1.74935660	1.92380527	1.39386202
H	-0.30453198	0.64347780	2.36963190
H	-3.37827140	3.13902568	0.66258186
H	-1.11300607	2.11723523	0.53125436
C	-3.24999776	1.97479679	6.39572113
C	-3.17995676	1.42189535	7.68010183
C	-2.50098485	3.12004711	6.07978607
C	-2.37047102	2.01072295	8.65246800
H	-3.73858836	0.52186795	7.91040534
C	-1.69841678	3.71104990	7.05771184
H	-2.53318101	3.53829541	5.07526435
C	-1.63311651	3.15691541	8.34292186
H	-1.11474360	4.59850571	6.81502956
H	-0.99445530	3.61447835	9.09820999
H	-6.43810942	-0.37395074	6.43151349
H	-2.30315632	1.55660186	9.63948542
H	-8.37247442	-1.33002866	6.86425307
H	-8.46829686	-5.04132154	-2.67938848
H	-7.12512143	-5.46782793	-0.81921201
H	-1.92947612	-1.50708283	0.98127199
H	-0.33785097	-2.73712456	4.06212435
H	-7.94060071	-3.36164115	3.03919917
C	-7.84254648	-2.38743245	4.99854677
C	-7.50679843	-3.68336130	5.76852568
C	-8.76764801	-1.48761731	5.85147706
C	-8.57491894	-2.75141092	3.68858922
H	-6.83834205	-4.32856683	5.18340812
H	-7.02989399	-3.47429274	6.73567591
H	-8.42688760	-4.25418686	5.96020632
C	-5.31635787	-2.11618889	2.51682548
C	-3.41630262	-2.31265715	-0.40405499
C	-2.72895609	-1.14386893	0.32346982
C	-2.36365630	-3.08419519	-1.24108307
C	-4.49126202	-1.78033278	-1.37800088
H	-3.44208163	-0.57107481	0.92562123
H	-2.27673088	-0.46713839	-0.41572308

H	-2.80976235	-3.97034038	-1.71382235
H	-1.96372671	-2.43208667	-2.03102515
H	-1.52538040	-3.41554403	-0.60971392
H	-4.01280816	-1.09583165	-2.09449516
H	-4.95332443	-2.59826996	-1.94847256
H	-5.27776266	-1.23897903	-0.84425027
C	-5.04490746	-4.29956752	0.08575588
O	-4.72181734	-5.39346938	-0.41390341
N	-6.31833861	-3.86393339	0.19505638
H	-6.48159870	-2.91494464	0.55755031
H	-9.48208586	-3.33115769	3.90853328
H	-8.85709221	-1.84442607	3.13678617
H	-8.90276069	-0.50300215	5.37852401
H	-9.75732055	-1.95777134	5.94910299
C	-8.08576024	-5.33425505	1.09966217
C	-9.46802461	-5.50127466	1.25286718
C	-9.98910988	-6.10118173	2.40490988
C	-9.13297400	-6.54828373	3.41441719
C	-7.75048019	-6.40279740	3.25903752
C	-7.23203177	-5.80085676	2.11123364
H	-10.14563470	-5.14505307	0.47977561
H	-11.06820803	-6.20804452	2.51485865
H	-9.53884573	-7.00369886	4.31739442
H	-7.07339134	-6.74818774	4.03958830
C	-8.48545988	-3.80727765	-0.91131421
C	-9.02059600	-2.65629232	-0.31226481
C	-9.92874854	-1.85549508	-1.00653245
C	-10.32112367	-2.20153217	-2.30640903
C	-9.79489554	-3.34952993	-2.90643903
C	-8.87855955	-4.14719673	-2.20978134
H	-8.72606490	-2.38369608	0.69907147
H	-10.33514986	-0.96161781	-0.53337273
H	-11.03292510	-1.57834127	-2.84728361
H	-10.09521725	-3.62526299	-3.91717799
C	-2.42454973	-2.09708692	5.78906613
C	-2.16606125	-2.49828487	7.25922111
C	-1.36092781	-1.47876566	6.50400794
H	-5.47043521	-1.25392491	9.29840716
C	-1.46849721	-3.81642198	7.47674315
C	-3.09267511	-2.06808047	8.37256743
H	-6.16115284	-2.90443413	9.08712082
C	-0.39975645	-0.57816684	6.67400910
C	-2.37352618	-3.02714316	4.67107706
O	-0.72653875	-4.35467213	6.66794573
O	-1.76162822	-4.32516041	8.70530497
C	-1.11151516	-5.59983088	9.02359159
H	-1.45915188	-5.84950137	10.02809037
H	-0.02398355	-5.47679377	9.00314947
H	-1.41670385	-6.36203942	8.29981910
O	-2.80185674	-1.28588393	9.26589552

O	-4.30721840	-2.67327967	8.26379721
C	-5.26254371	-2.32889849	9.31858708
H	-4.85674647	-2.61004398	10.29622306
O	-3.21458298	-3.94952818	4.53416448
C	-1.33761236	-2.75644023	3.60409100
H	-0.10138808	0.03476426	5.81857770
H	0.25797782	0.75004248	8.22921128
H	1.36954009	-0.59629248	7.91960992
H	-0.16891030	-0.88376202	8.79012279
C	0.30363036	-0.32021883	7.97666004
H	-3.49995926	-1.20736782	5.61680722
H	-1.51704371	-1.77221828	3.15498104
H	-1.38015373	-3.52159930	2.82096946
H	-3.12506186	-4.02178198	0.85776300

TS4-repro-LA2-RA2-R
COSMO(Et_2O)-ZORA-BLYP-D3(BJ)/DZP
E = -17950.03
G = -17343.91
COSMO(Et_2O)-ZORA-M06-2X/TZ2P//COSMO(Et_2O)-ZORA-BLYP-D3(BJ)/DZP
E = -25716.01
G = -25109.89
N_{imag} = 1, 1502 *i* cm^{-1}

C	-4.76837087	0.45268992	3.19048228
N	-3.95345581	0.87364732	4.34923974
H	-3.90103335	1.89906538	4.40099890
C	-2.11056387	-0.95374210	0.78374004
S	-2.73465973	-1.44004643	5.08391213
N	-2.47431431	1.10466397	6.03857293
H	-2.75367176	2.08100562	5.87967415
C	-1.19031683	1.06642324	6.76903182
H	-0.90392366	2.12432409	6.80131423
H	0.54811515	-1.26715718	0.34301764
C	-0.06187532	0.33923271	6.03579457
N	0.11450977	0.83401567	4.63639510
H	-0.27467970	-0.73314507	6.02179645
P	1.47899482	0.31328216	3.91836622
C	1.51526362	1.03495321	2.25718054
C	0.31139582	1.45466206	1.67324541
C	2.69353320	0.99975142	1.49559211
C	0.28750378	1.83956046	0.33300825
H	-0.59718860	1.45846427	2.26470426
C	2.65331483	1.34956558	0.14545271
H	3.63686332	0.70519042	1.95202260
C	1.45135057	1.76823556	-0.43663841
H	-0.64729209	2.17657124	-0.11238067
H	3.56564462	1.31456333	-0.44887455
H	1.42636996	2.04792775	-1.48950020
C	1.59089067	-1.49317999	3.66223239
C	0.50830139	-2.30422706	4.02913936

C	2.74935225	-2.08423995	3.12446727
C	0.61746680	-3.69289845	3.94612078
H	-0.42382253	-1.85674869	4.36211160
C	2.85683215	-3.47304528	3.05933242
H	3.56782382	-1.47436652	2.75244763
C	1.80010369	-4.27976932	3.49326269
H	-0.23360098	-4.31286122	4.22334360
H	3.76059732	-3.92467561	2.65151489
H	1.88436770	-5.36408900	3.43463628
C	2.95369434	0.78451133	4.91229025
C	2.86195608	2.03192054	5.55427515
C	4.06582035	-0.03612257	5.17017153
C	3.87728235	2.47032615	6.40263593
H	1.98872986	2.65628418	5.41906739
C	5.08200908	0.40739565	6.02129566
H	4.13906662	-1.02906318	4.74191100
C	4.99392530	1.66182552	6.63451043
H	5.93877485	-0.23755468	6.21368607
H	5.78537042	1.99941704	7.30303291
H	0.85237336	0.49580611	6.62526111
H	3.78661519	3.44174657	6.88456724
H	0.80183205	0.36498585	8.72375247
H	-1.17059030	-0.01018193	-1.56950668
H	-1.42163526	-0.10300328	0.76808498
H	-6.61633471	-0.22403249	1.34421913
H	-2.75877659	2.84143106	2.08036075
H	-3.45677851	1.21436024	8.41048881
C	-1.34292580	0.64615772	8.27432044
C	-1.65043276	-0.85452032	8.44405318
C	-0.03935775	1.00046616	9.03040234
C	-2.49957000	1.47336189	8.88135945
H	-2.54860562	-1.13596188	7.88166194
H	-0.82144862	-1.48110824	8.08700062
H	-1.81102497	-1.07923306	9.50964836
C	-3.04249198	0.24231869	5.14752975
C	-5.93487419	-0.59088438	3.40316200
C	-7.02225816	-0.20473231	2.36639870
C	-5.58181192	-2.07627650	3.16997934
C	-6.48948789	-0.40760800	4.83066318
H	-7.41288868	0.80533989	2.55970695
H	-7.85934777	-0.91552472	2.41581391
H	-4.78815271	-2.41164116	3.84281983
H	-6.48271592	-2.68331224	3.34669768
H	-5.26020982	-2.25036365	2.13644961
H	-7.40471511	-1.00550469	4.95130927
H	-5.75595963	-0.73536685	5.57874331
H	-6.73614265	0.64616428	5.02675684
C	-3.89382357	0.27445953	1.92753628
O	-3.97175041	1.10051188	1.00030193
N	-3.05130389	-0.78753755	1.89738984

H	-2.87756418	-1.28434630	2.78732413
H	-2.57592111	1.27612397	9.96030962
H	-2.33040545	2.55252951	8.74245158
H	0.24617652	2.04997650	8.86469886
H	-0.18788664	0.85698763	10.11062453
C	-1.29648806	-2.22964210	0.92101897
C	-1.91112328	-3.44880274	1.23423154
C	-1.19054954	-4.64273157	1.14977459
C	0.14621553	-4.62665617	0.74039887
C	0.77151435	-3.40897299	0.45789403
C	0.05641970	-2.21463241	0.55692745
H	-2.95943727	-3.46909590	1.52594637
H	-1.67596612	-5.58678174	1.39684261
H	0.70715427	-5.55800962	0.67004777
H	1.82268598	-3.38743075	0.17564181
C	-2.81094111	-0.99330888	-0.57673618
C	-4.05298590	-1.61367330	-0.74730824
C	-4.63291649	-1.70023688	-2.01511402
C	-3.96977994	-1.17401458	-3.13076181
C	-2.72158973	-0.56261516	-2.96602892
C	-2.14669816	-0.47620007	-1.69430753
H	-4.57697463	-2.01620880	0.11524070
H	-5.60616804	-2.17677613	-2.13306979
H	-4.42330776	-1.23824844	-4.11943656
H	-2.19643965	-0.15071988	-3.82776957
C	-0.62091684	3.53197106	4.36340839
C	0.35511828	4.69912288	4.02878516
C	0.00407233	4.25899141	5.42126897
H	3.59731925	3.69216533	1.72406005
C	-0.27662339	6.03347817	3.68385444
C	1.72696548	4.51882117	3.42676873
H	2.66293174	4.31207414	0.31904334
C	0.28833345	4.55488626	6.68438615
C	-2.01499487	3.64032048	3.93252070
O	-1.43903375	6.33905427	3.90655412
O	0.63701268	6.87712108	3.13060021
C	0.12806000	8.20993151	2.79491259
H	0.97958159	8.73331097	2.35547145
H	-0.21871511	8.71464765	3.70227341
H	-0.69419195	8.12271929	2.07774090
O	2.78281470	4.56986798	4.04009936
O	1.63473735	4.36982876	2.07877171
C	2.91005448	4.46486133	1.37027667
H	3.34254767	5.45837169	1.53676008
O	-2.98125873	3.52751015	4.72646441
C	-2.27459844	3.75906748	2.44706582
H	-0.20267022	2.11774267	4.40597277
H	0.81454240	6.41598433	7.64965459
H	1.80747955	6.00791721	6.21763115
H	2.05539481	5.16025914	7.76410579

C	1.29560220	5.59414722	7.09477601
H	-0.22923803	4.01803131	7.48243900
H	-1.35525897	3.93214763	1.87859916
H	-2.97695961	4.58622136	2.27696574
H	-5.29622566	1.38631320	2.96194609

TS4-repro-LA1’-RA1-S
COSMO(Et_2O)-ZORA-BLYP-D3(BJ)/DZP
E = -17958.47
G = -17356.32
COSMO(Et_2O)-ZORA-M06-2X/TZ2P//COSMO(Et_2O)-ZORA-BLYP-D3(BJ)/DZP
E = -25728.57
G = -25126.42
N_{imag} = 1, 1335 i cm^{-1}

C	-5.66508587	-2.36436096	2.06711613
N	-4.32258847	-1.78079013	2.10455497
H	-3.93561403	-1.40437850	1.22799094
C	-7.62468845	0.05328474	4.29988542
S	-3.99152435	-2.33738760	4.74093783
N	-2.45732774	-0.87124802	3.04879139
H	-2.46251493	-0.32519081	2.18555994
C	-1.68703736	-0.21748658	4.10882527
H	-2.28881990	-0.31166231	5.02425373
H	-7.73275854	-1.16846244	6.75412535
C	-1.62374318	1.29424219	3.75348477
N	-1.51542440	1.59973303	2.29183844
H	-0.79154317	1.74808801	4.30470884
P	-2.15469661	3.04073101	1.87906727
C	-1.23491275	4.44248688	2.60030012
C	0.00515913	4.16834298	3.19741841
C	-1.72143497	5.76244972	2.58303872
C	0.74935701	5.19848033	3.77542013
H	0.39305614	3.15384154	3.17804650
C	-0.96715105	6.79123111	3.14921902
H	-2.68391341	5.98934382	2.12953171
C	0.26562898	6.50988286	3.75020811
H	1.71137774	4.97819155	4.23667366
H	-1.34412118	7.81292829	3.12531489
H	0.84881249	7.31452775	4.19660573
C	-3.88593847	3.19258239	2.45738162
C	-4.28841225	4.07865687	3.46783333
C	-4.84540520	2.36411051	1.84183689
C	-5.63984965	4.18202538	3.80995702
H	-3.55651150	4.68374604	3.99555870
C	-6.18898684	2.46888813	2.19041227
H	-4.54930884	1.64003831	1.08784857
C	-6.59012876	3.39385099	3.16145093
H	-5.94272751	4.86542624	4.60061315
H	-6.90976347	1.80614689	1.71635262
H	-7.64079224	3.47705420	3.43356439

C	-2.22622318	3.26930900	0.07869131
C	-2.71557325	2.23318837	-0.73452725
C	-1.95122014	4.52685081	-0.48465775
C	-2.93147786	2.46271371	-2.09232032
H	-2.88544028	1.23745567	-0.33477301
C	-2.15881843	4.73952193	-1.84864132
H	-1.56734754	5.33802476	0.12501092
C	-2.65790745	3.71234510	-2.65293189
H	-1.92576854	5.71179733	-2.28081908
H	-2.82020048	3.88281319	-3.71650868
H	-2.54566909	1.75216294	4.13818325
H	-3.30178703	1.65078048	-2.71469443
H	0.38286304	0.82006845	5.71017587
H	-4.87616170	0.31756373	4.57095681
H	-7.99139705	0.63917803	3.44626279
H	-4.71083047	-1.97662926	-0.63007727
H	-1.10311228	-2.61674237	1.58134105
H	-0.81800892	-2.86032726	3.70656476
C	-0.29441291	-0.86126685	4.44270568
C	0.75783718	-0.59519246	3.35112901
C	0.18813985	-0.25898752	5.78429934
C	-0.45265375	-2.38578027	4.62591958
H	0.48661919	-1.08986705	2.41659304
H	0.88289957	0.47400274	3.13540482
H	1.73236913	-0.99255914	3.67107550
C	-3.57715794	-1.63207726	3.23301468
C	-5.92003298	-3.17618734	0.76708897
C	-5.74431477	-2.33030756	-0.51560868
C	-4.91243669	-4.34983140	0.74693740
C	-7.35675759	-3.74078347	0.83762266
H	-6.41469266	-1.46407587	-0.50204630
H	-5.97924860	-2.95248521	-1.39243942
H	-5.02274866	-4.98036264	1.64195109
H	-5.08443293	-4.97554479	-0.14080423
H	-3.87871987	-3.98265310	0.71330036
H	-7.55135678	-4.37381200	-0.03996493
H	-7.49165065	-4.35702881	1.73968483
H	-8.09887184	-2.93243065	0.85244893
C	-6.68617782	-1.22589310	2.37532786
O	-7.28681847	-0.56573090	1.51437585
N	-6.80271592	-1.00952103	3.71376434
H	-6.20329873	-1.56745849	4.33704456
H	0.52203832	-2.82965367	4.87781937
H	-1.16774362	-2.61676692	5.42461681
H	-0.55672934	-0.42066511	6.57804842
H	1.12569892	-0.74365886	6.09240262
C	-8.83113473	-0.57305193	4.99254417
C	-10.07664042	-0.58245557	4.35472868
C	-11.17899865	-1.19660725	4.96060859
C	-11.03856587	-1.81152895	6.20884838

C	-9.79262718	-1.80668343	6.85010429
C	-8.69619096	-1.18795011	6.24515867
H	-10.18289624	-0.10992765	3.37827991
H	-12.14598459	-1.19433548	4.45788340
H	-11.89503827	-2.29092030	6.68243878
H	-9.67853800	-2.28151748	7.82443500
C	-6.82238602	0.97847154	5.21526313
C	-7.50929311	1.86724243	6.05655456
C	-6.81168421	2.75396438	6.87915976
C	-5.41252073	2.75496556	6.88332207
C	-4.72453412	1.87229646	6.04769173
C	-5.42362704	0.99613861	5.21379532
H	-8.59872422	1.86286990	6.06933465
H	-7.36152850	3.44110692	7.52231582
H	-4.86476280	3.44243714	7.52744359
H	-3.63583194	1.86856971	6.04351275
C	-0.41318236	0.01286938	0.30601214
C	0.12218777	0.74422701	-0.99418690
C	0.93299653	-0.15692395	-0.07578074
H	-2.00802729	-0.28392694	-4.26230793
C	0.53836693	2.18741628	-0.99663153
C	-0.30751136	0.07709299	-2.28040999
H	-1.36941098	1.14948575	-5.15351924
C	2.18516818	-0.58125765	0.03247798
C	-1.43155741	-1.04312529	0.17048148
O	0.71516988	2.87663102	-1.98906076
O	0.83703989	2.62571057	0.26811517
C	1.50638841	3.92202914	0.29547677
H	1.87918615	4.03234824	1.31378522
H	0.79433046	4.72013959	0.06102618
H	2.32753146	3.93699592	-0.42870617
O	-0.41993531	-1.14039723	-2.38864559
O	-0.60501611	0.94005845	-3.27831031
C	-1.12070730	0.31399481	-4.49533654
H	-0.35335543	-0.32444733	-4.94632496
O	-2.63236877	-0.76894844	-0.06192070
C	-1.03402087	-2.46548697	0.49564274
H	2.44195683	-1.23939212	0.86702079
H	3.72808439	-1.09770730	-1.39330297
H	4.10069222	0.32157257	-0.40289197
H	2.90803889	0.44864593	-1.72114601
C	3.28431187	-0.20534758	-0.92338120
H	-0.88336330	0.88403957	1.41098664
H	-0.00608054	-2.67491814	0.17859289
H	-1.73489960	-3.15507181	0.01137372
H	-5.70882391	-3.07228781	2.90579178

TS4-repro-LA1’-RA2-S
COSMO(Et_2O)-ZORA-BLYP-D3(BJ)/DZP
E = -17958.75

G = -17358.04
COSMO(Et_2O)-ZORA-M06-2X/TZ2P//COSMO(Et_2O)-ZORA-BLYP-D3(BJ)/DZP
E = -25728.76
G = -25128.05
N_{imag} = 1, 1772 i cm^{-1}

C	-5.02307631	-1.96274553	4.31097114
N	-4.27703786	-0.79320394	3.81672563
H	-4.78559985	-0.14223366	3.21028376
C	-5.66548532	-0.26735836	7.67402943
S	-2.05963190	-1.28206608	5.28092955
N	-2.97446963	1.03975925	4.17009260
H	-3.71499724	1.42985888	3.57104254
C	-1.77866198	1.91493392	4.20178279
H	-2.12555836	2.78004758	3.61951310
H	-6.19524150	-1.44124923	10.09917607
C	-0.59852522	1.33457006	3.38331590
N	-0.61220739	1.89662209	2.00693575
H	-0.66785782	0.24114371	3.35656988
P	0.77824365	2.36599147	1.33977221
C	0.36776804	3.34757734	-0.13089379
C	-0.93358971	3.84656459	-0.29174213
C	1.37988653	3.72661402	-1.02827131
C	-1.22049531	4.71193027	-1.34928900
H	-1.70878086	3.56760790	0.41396592
C	1.09048009	4.60845645	-2.07034540
H	2.38946265	3.33830018	-0.91993767
C	-0.20956315	5.10152975	-2.23185050
H	-2.23538893	5.08564563	-1.47838041
H	1.87843177	4.90286762	-2.76257163
H	-0.43410176	5.78500380	-3.05012164
C	1.94856166	1.02442425	0.94533603
C	1.47604376	-0.29447883	1.01459038
C	3.29558896	1.27427743	0.62854968
C	2.34519480	-1.35695831	0.75904923
H	0.43261875	-0.48075483	1.24555827
C	4.15524479	0.20829822	0.35928053
H	3.68129183	2.29184038	0.61749474
C	3.68042077	-1.10808408	0.42583960
H	1.97829199	-2.38048469	0.81347683
H	5.19781969	0.40258369	0.11000896
H	4.35519863	-1.93931795	0.22346548
C	1.73299057	3.47867084	2.42944269
C	2.64576334	2.95378334	3.36078769
C	1.44529024	4.85402748	2.43329767
C	3.25880287	3.79845403	4.28936483
H	2.87825843	1.89062375	3.36182521
C	2.06601398	5.69314889	3.35934380
H	0.73965279	5.26642462	1.71441340
C	2.96912210	5.16651594	4.29014135
H	1.84339048	6.75965600	3.35679199

H	3.44762242	5.82352877	5.01565844
H	0.34525531	1.57610250	3.88539398
H	3.95986241	3.38602322	5.01418904
H	0.42981965	3.52648877	4.86037906
H	-5.83983680	1.90238244	9.31779711
H	-6.65707873	-0.67405229	7.92242084
H	-6.28674815	-0.89200921	1.97620135
H	-3.73890217	2.61597265	-0.89283789
H	-3.36855328	2.15714836	6.49311172
C	-1.40414812	2.53957550	5.59544275
C	-0.61725215	1.59741881	6.52877722
C	-0.53351362	3.78873910	5.30933879
C	-2.70049152	2.99773340	6.29049418
H	-1.19636426	0.70375737	6.77493717
H	0.32255138	1.26110656	6.06732473
H	-0.36408994	2.13542198	7.45528462
C	-3.13398514	-0.28793085	4.38517685
C	-5.80968798	-2.67518420	3.17218320
C	-6.80819734	-1.72213791	2.47340976
C	-4.80216462	-3.19217107	2.12255636
C	-6.55567857	-3.88676546	3.77841401
H	-7.53161330	-1.31480623	3.18816131
H	-7.35214556	-2.27573672	1.69412595
H	-4.03908825	-3.83240247	2.58946874
H	-5.33336566	-3.78722448	1.36489260
H	-4.29509885	-2.36511681	1.61837663
H	-7.04622869	-4.45501095	2.97471733
H	-5.84788618	-4.56085616	4.28547021
H	-7.31671673	-3.56918603	4.49946291
C	-5.89540661	-1.49104803	5.52166014
O	-7.13177238	-1.40649129	5.51669865
N	-5.13388751	-1.08943624	6.57668927
H	-4.11221526	-1.16740432	6.49473083
H	-2.46546141	3.49158099	7.24413108
H	-3.25528804	3.70989796	5.66315145
H	-1.04656607	4.48624734	4.63102846
H	-0.32643261	4.31668718	6.25153407
C	-4.74226040	-0.42357625	8.87253394
C	-3.42938691	0.06593117	8.80592845
C	-2.55891908	-0.08672909	9.88539380
C	-2.99865445	-0.72148952	11.05466142
C	-4.30869120	-1.20513168	11.13062831
C	-5.17580118	-1.05989898	10.04001496
H	-3.08789396	0.57032306	7.90767747
H	-1.53956305	0.29207982	9.81660289
H	-2.32292545	-0.83714670	11.90174415
H	-4.65728521	-1.69817115	12.03784336
C	-5.85492268	1.20727494	7.27232472
C	-5.97086201	1.60390758	5.93400770
C	-6.15919658	2.94672172	5.59715182

C	-6.23768453	3.91791555	6.59819462
C	-6.12800903	3.53265891	7.93876170
C	-5.94160384	2.18810020	8.27192431
H	-5.89895157	0.87353412	5.13870132
H	-6.22762482	3.22874905	4.54657792
H	-6.37316622	4.96751760	6.33801777
H	-6.17945499	4.28144017	8.72937558
C	-2.83488677	0.88413918	0.80997507
C	-2.58747238	0.10764741	-0.50947733
C	-2.42107722	-0.47965533	0.86554260
H	-5.73453442	-2.29378868	-1.32189171
C	-1.41403772	0.48211947	-1.37585840
C	-3.73405917	-0.48741133	-1.28170100
H	-6.82709209	-1.12891470	-0.49304363
C	-1.93829481	-1.57483818	1.43605589
C	-4.06349062	1.63215960	1.03466266
O	-0.27454745	0.05826558	-1.26871750
O	-1.80270601	1.40556123	-2.29820723
C	-0.77390141	1.77463065	-3.27058248
H	-0.58038609	0.92278564	-3.93298386
H	0.14221245	2.06459650	-2.75424057
H	-1.19404577	2.61455148	-3.82652968
O	-3.62511711	-0.93597312	-2.41727978
O	-4.87970687	-0.53509933	-0.55223253
C	-5.99367749	-1.23801258	-1.18788448
H	-6.22257821	-0.78167069	-2.15646653
O	-4.73958264	1.62254097	2.09256952
C	-4.47132911	2.58117531	-0.07997178
H	-1.70347910	1.53360806	1.30423523
H	-0.39667309	-3.01531415	1.00324046
H	-1.35672653	-2.52149675	-0.41483355
H	-2.03946057	-3.63475090	0.80743680
C	-1.40670311	-2.74487716	0.65777002
H	-1.94811028	-1.65966970	2.52322303
H	-4.62153110	3.58603601	0.33950521
H	-5.43683093	2.24366267	-0.48471613
H	-4.26963704	-2.66280185	4.69847062

TS4-repro-LA2-RA1-S (TS4-S)
COSMO(Et_2O)-ZORA-BLYP-D3(BJ)/DZP
E = -17962.37
G = -17362.15
COSMO(Et_2O)-ZORA-M06-2X/TZ2P//COSMO(Et_2O)-ZORA-BLYP-D3(BJ)/DZP
E = -25732.77
G = -25132.55
N_{imag} = 1, 1714 *i* cm^{-1}

C	-1.09136374	-2.02136065	3.93576938
N	-2.56834780	-1.87508712	3.86016777
H	-3.03376094	-2.64814280	4.35642487
C	-0.85665127	-3.09941991	0.26862068

S	-2.99862055	0.19936620	2.12503664
N	-4.57244954	-1.88353646	2.78050789
H	-4.64103038	-2.80011495	3.22695873
C	-5.84178110	-1.27672143	2.37230207
H	-5.63657434	-0.21115783	2.21299415
H	-2.41121271	-4.23126270	2.25672991
C	-6.82805510	-1.43342752	3.55410236
N	-6.35033330	-0.75469710	4.76933551
H	-6.92007858	-2.50245911	3.79933402
P	-6.92368275	0.69117166	5.20705065
C	-8.16348509	0.66177765	6.54798116
C	-8.20861704	-0.44911617	7.40186024
C	-9.05021638	1.73485000	6.74312970
C	-9.13050645	-0.48429296	8.45025502
H	-7.51372386	-1.26871161	7.26312116
C	-9.97064415	1.69379075	7.79328949
H	-9.02783952	2.59505252	6.07615252
C	-10.01080325	0.58552659	8.64825825
H	-9.15803211	-1.34781640	9.11379133
H	-10.65795887	2.52575009	7.94289078
H	-10.72982409	0.55636049	9.46662409
C	-7.77522907	1.47586051	3.79227745
C	-9.10932877	1.13045859	3.50981929
C	-7.07303363	2.31191534	2.90892320
C	-9.72862676	1.61544231	2.35695927
H	-9.66310132	0.48584106	4.19031328
C	-7.69971358	2.79487056	1.75771928
H	-6.04285888	2.58921502	3.11769905
C	-9.02465679	2.44603954	1.47873072
H	-10.76107236	1.34134367	2.14284307
H	-7.14887474	3.44339096	1.07731565
H	-9.50866557	2.82051559	0.57733013
C	-5.60848146	1.84159087	5.71106584
C	-4.41145521	1.83457337	4.98300797
C	-5.79478305	2.76626161	6.74954678
C	-3.41860253	2.77328895	5.26779208
H	-4.24591429	1.10204526	4.19761178
C	-4.79020774	3.69152947	7.04058248
H	-6.70348763	2.75162804	7.34526621
C	-3.60562653	3.70383883	6.29447733
H	-4.93042907	4.40053576	7.85603198
H	-2.82549200	4.42983266	6.52303374
H	-7.82809071	-1.10963838	3.24797982
H	-2.49475580	2.76355833	4.69195950
H	-8.41744546	-1.01427834	1.20929952
H	1.23421593	-3.05515437	-1.49138324
H	0.02805373	-3.74207447	0.38319861
H	-0.49368207	0.56534720	2.75354561
H	-7.44728305	-4.41003161	5.43672631
H	-4.42829340	-2.48924389	0.26641467

C	-6.38530955	-1.86445376	1.02705274
C	-6.87420542	-3.31919281	1.20871797
C	-7.54882831	-0.97023406	0.53961575
C	-5.25757003	-1.83796511	-0.02737617
H	-6.08831866	-3.94733064	1.64592447
H	-7.76676829	-3.37561933	1.84739340
H	-7.12945754	-3.75370329	0.23108005
C	-3.38691211	-1.24575286	2.96619765
C	-0.22169675	-0.80340750	4.43946802
C	0.32328261	0.09395588	3.30921033
C	-1.06974363	0.01921099	5.42428711
C	0.98340873	-1.42153941	5.19263899
H	0.93821505	-0.48214903	2.60289526
H	0.95697347	0.87937780	3.74868843
H	-1.45545484	-0.61863093	6.22729077
H	-0.45740740	0.81970201	5.86634797
H	-1.91890393	0.47512857	4.90814938
H	1.64901873	-0.62168810	5.54893959
H	0.64072785	-2.00262693	6.06112416
H	1.56077195	-2.08784575	4.53643941
C	-0.51393734	-2.74784139	2.69954512
O	0.25017907	-3.72034245	2.83877031
N	-0.93874145	-2.29517762	1.49428378
H	-1.55029687	-1.46942048	1.47337082
H	-5.64085316	-2.19099985	-0.99578827
H	-4.86422448	-0.81995099	-0.14830173
H	-7.23251827	0.08026372	0.47115407
H	-7.87868359	-1.29506430	-0.45850923
C	-2.08930596	-4.01203418	0.12682797
C	-2.56009425	-4.39024579	-1.13834272
C	-3.66985494	-5.23300868	-1.26385602
C	-4.32013640	-5.71688774	-0.12510228
C	-3.84888900	-5.35435631	1.14030132
C	-2.74529955	-4.50852472	1.26322155
H	-2.07129611	-4.00917848	-2.03300086
H	-4.03116601	-5.50255082	-2.25633493
H	-5.19316662	-6.36159728	-0.22324354
H	-4.34354846	-5.71172405	2.04282952
C	-0.65526223	-2.18303992	-0.92635427
C	-1.61942119	-1.21365432	-1.24244636
C	-1.43998113	-0.37059081	-2.34053278
C	-0.29845849	-0.49433059	-3.14389215
C	0.66298004	-1.46195145	-2.83668124
C	0.48378464	-2.30114257	-1.72974222
H	-2.51119373	-1.11506936	-0.62768220
H	-2.19294157	0.38225617	-2.57392457
H	-0.16083342	0.16071346	-4.00392817
H	1.55330029	-1.56504986	-3.45687664
C	-5.46545658	-2.85533154	6.23286430
C	-4.36210800	-2.52908646	7.30314057

C	-5.74297837	-3.05645525	7.61182406
H	-1.57498930	-5.45058860	8.08958437
C	-4.18831088	-1.07879051	7.65082610
C	-3.17700717	-3.45285560	7.28280890
H	-2.91526844	-6.64012991	7.89163284
C	-6.49615079	-3.39754687	8.64819139
C	-5.32064268	-3.97309088	5.30233240
O	-5.09613583	-0.26292092	7.57758311
O	-2.95745760	-0.78508373	8.13924854
C	-2.77698352	0.62775925	8.49147621
H	-1.72954737	0.70966018	8.78962420
H	-3.44701320	0.89424911	9.31600740
H	-2.99171104	1.25458575	7.62338896
O	-2.07383426	-3.16242357	6.84382569
O	-3.51067654	-4.69410439	7.73481981
C	-2.47631218	-5.70973515	7.52472807
H	-2.24066983	-5.77823767	6.45763843
O	-4.31970738	-4.14930309	4.57058724
C	-6.49876295	-4.92101401	5.22918357
H	-7.51437051	-3.75194426	8.46149876
H	-6.01533774	-4.31959157	10.54362499
H	-6.70754035	-2.69464777	10.67914566
H	-5.02028394	-2.90055254	10.13676727
C	-6.03215521	-3.32192221	10.07584837
H	-5.87391753	-1.70096488	5.57725520
H	-6.36099242	-5.68273935	6.01458082
H	-6.53274221	-5.42033562	4.25399580
H	-0.99834645	-2.76747104	4.73053739

TS4-repro-LA2-RA2-S
COSMO(Et_2O)-ZORA-BLYP-D3(BJ)/DZP
E = -17959.16
G = -17359.30
COSMO(Et_2O)-ZORA-M06-2X/TZ2P//COSMO(Et_2O)-ZORA-BLYP-D3(BJ)/DZP
E = -25726.40
G = -25126.54
N_{imag} = 1, 1756 i cm^{-1}

C	-4.53458207	0.57561643	2.54758952
N	-3.45465311	0.60528687	3.56192318
H	-3.41268804	1.53248069	4.00320477
C	-3.09287913	-1.67795613	-0.23125385
S	-2.54761973	-1.97272188	3.82404213
N	-2.46060561	0.10771126	5.53801768
H	-2.65743945	1.10445717	5.69515107
C	-1.47794484	-0.42430879	6.50022054
H	-1.29432100	0.45131088	7.13688799
H	-0.36806761	-1.71439052	-0.21508779
C	-0.13311551	-0.78446060	5.84614881
N	0.36474231	0.31174581	4.98783728
H	-0.25195129	-1.70260459	5.26812214

P	1.40876699	0.05846713	3.78178184
C	0.75286468	0.27626520	2.09490502
C	-0.52218472	0.82188867	1.91310510
C	1.56439530	-0.00641961	0.98053751
C	-0.96756295	1.14066530	0.62799248
H	-1.16456827	0.98538171	2.77028874
C	1.12194516	0.32750619	-0.29963544
H	2.53688493	-0.47841162	1.11501250
C	-0.13863410	0.91492700	-0.47378890
H	-1.96305354	1.55430126	0.48324539
H	1.75309697	0.11905541	-1.16299348
H	-0.48374007	1.17289451	-1.47477152
C	2.07486054	-1.63078822	3.82612036
C	1.24179890	-2.67585746	3.38658991
C	3.32884332	-1.91568386	4.38974355
C	1.65906279	-3.99834258	3.53357876
H	0.27137907	-2.45701927	2.94536629
C	3.74325728	-3.24417276	4.51944021
H	3.98029902	-1.10815454	4.71617148
C	2.90623670	-4.28471783	4.10017028
H	1.00825407	-4.80451324	3.20283515
H	4.71977460	-3.46564495	4.94873906
H	3.22885909	-5.31958586	4.21040495
C	2.78417848	1.23986168	3.95328446
C	3.26932452	1.45392746	5.25436052
C	3.36306055	1.92328493	2.87314406
C	4.35076611	2.30653405	5.46681167
H	2.78006515	0.97886220	6.09986866
C	4.44170097	2.78307386	3.09396822
H	2.96785508	1.80808109	1.86824633
C	4.94558011	2.96261997	4.38600448
H	4.88207595	3.32037339	2.25458385
H	5.78572640	3.63632012	4.55241036
H	0.58122341	-0.99072087	6.66006695
H	4.70429615	2.48050980	6.48143043
H	-0.35717003	-1.24302930	8.87081092
H	-2.81945756	-2.18149001	-2.86546620
H	-2.50524737	-0.88797914	-0.72215067
H	-6.78838732	-0.12067486	1.03312983
H	-2.39554056	3.64016032	2.65875128
H	-4.01280203	-1.75958038	6.57659310
C	-2.02471005	-1.55477818	7.47383938
C	-1.60101046	-2.98828145	7.07611647
C	-1.45574639	-1.26071431	8.88086180
C	-3.56524522	-1.48330505	7.53929350
H	-1.94118938	-3.23452511	6.06518935
H	-0.51042836	-3.11765113	7.12026989
H	-2.04797944	-3.70119969	7.78578198
C	-2.85434965	-0.36719605	4.33144915
C	-5.92630361	0.03565267	3.05436918

C	-6.98298469	0.39355758	1.98309772
C	-5.96487636	-1.48386502	3.31583550
C	-6.26245867	0.77908010	4.36779770
H	-6.99488227	1.47477267	1.78269782
H	-7.98002948	0.09511995	2.33764257
H	-5.29313610	-1.77134563	4.13066530
H	-6.99070615	-1.77217237	3.59182466
H	-5.67597867	-2.05744966	2.42750508
H	-7.27088617	0.49984386	4.70559752
H	-5.55324232	0.51988918	5.16489472
H	-6.23839080	1.86979125	4.22338887
C	-4.12963003	0.09628468	1.13044874
O	-4.32478550	0.84346623	0.15241987
N	-3.61363966	-1.14375229	1.03073718
H	-3.29918201	-1.60175623	1.91289496
H	-3.93266479	-2.18033057	8.30714230
H	-3.90687349	-0.47134458	7.80087491
H	-1.81494606	-0.29087040	9.25151117
H	-1.77309680	-2.03938478	9.59021932
C	-2.16934367	-2.84501632	0.10505915
C	-2.69933295	-4.08108567	0.49933370
C	-1.84869122	-5.13384311	0.84602901
C	-0.46008148	-4.96398060	0.78854695
C	0.07175105	-3.73173884	0.39854580
C	-0.78203322	-2.67671218	0.06534537
H	-3.77869643	-4.22227527	0.52850149
H	-2.26909571	-6.09103947	1.15424560
H	0.20146671	-5.79024836	1.04653142
H	1.15060759	-3.58423552	0.36963351
C	-4.19258921	-2.10217389	-1.20138451
C	-5.51117727	-2.31609828	-0.79068604
C	-6.47723596	-2.75607530	-1.70262552
C	-6.13038856	-2.98600478	-3.03765257
C	-4.80966445	-2.77408441	-3.45480491
C	-3.84884885	-2.33622798	-2.54034928
H	-5.78412949	-2.13711326	0.24462071
H	-7.50220738	-2.91689952	-1.36838339
H	-6.88186995	-3.32623180	-3.74964556
H	-4.53001170	-2.94979348	-4.49349173
C	-0.29252661	2.95624700	5.27026267
C	0.12606854	3.60584121	6.61668705
C	0.81799527	3.84430773	5.30322896
H	-2.29384935	6.00051566	8.84417714
C	0.83548125	2.78441138	7.66562141
C	-0.68946859	4.77236011	7.10413692
H	-0.95967764	6.15380500	10.04689607
C	1.80141041	4.56906499	4.78848387
C	-1.56304146	3.22953945	4.61382467
O	2.04773393	2.72829615	7.80795078
O	-0.04816269	2.11522360	8.46005483

C	0.56668633	1.46082800	9.61747847
H	-0.26632176	1.07243331	10.20382969
H	1.14426063	2.19509755	10.18941015
H	1.22234237	0.64882057	9.28782121
O	-1.40543672	5.46503167	6.39481109
O	-0.49026333	4.99454068	8.43446619
C	-1.21749033	6.14038789	8.98574159
H	-0.89254176	7.06146219	8.49107799
O	-2.67292515	2.91687495	5.11229625
C	-1.48750780	3.84870204	3.23659151
H	0.01727179	1.57208068	5.17447271
H	3.74314396	5.22279973	5.46097249
H	2.44887127	5.38783774	6.67039982
H	2.55690388	6.52835840	5.29875555
C	2.68170739	5.47559969	5.60167957
H	1.99736575	4.49903606	3.71576798
H	-0.59974396	3.51453842	2.68808846
H	-1.39324848	4.93801051	3.37634001
H	-4.70639918	1.64530313	2.37069360

DBU

COSMO(Et_2O)-ZORA-BLYP-D3(BJ)/DZP

E = -3442.79

G = -3315.91

COSMO(Et_2O)-ZORA-M06-2X/TZ2P//COSMO(Et_2O)-ZORA-BLYP-D3(BJ)/DZP

E = -4900.72

G = -4773.84

N_{imag} = 0

C	1.64703963	-0.52856348	-0.75758136
C	-0.44418033	-0.81792912	0.34552667
C	0.56919166	1.42992424	0.34109566
N	-0.49302821	0.49942632	0.76177994
N	0.51260101	-1.37541204	-0.34032794
C	1.88385485	0.66981888	0.16876272
H	1.46319141	-0.17101946	-1.78722122
H	2.54680136	-1.16145167	-0.80225092
C	-1.63323975	-1.69393739	0.69726229
H	0.66698683	2.20566915	1.11387276
H	0.28896174	1.93066650	-0.60382668
C	-1.71739055	1.10962336	1.30757056
H	2.65752484	1.34040268	-0.23380817
H	2.22292355	0.30930856	1.15344523
C	-2.91010361	1.14340894	0.33035853
C	-3.69654267	-0.17731241	0.27356181
C	-2.87840143	-1.40030474	-0.17509433
H	-1.89358370	-1.60042474	1.76378871
H	-1.30199819	-2.72528991	0.52525269
H	-1.44523539	2.13490580	1.58870435
H	-2.01114154	0.59582859	2.23733067
H	-2.53712139	1.41427272	-0.67177529

H	-3.59411257	1.94731176	0.64868481
H	-4.56051982	-0.05579535	-0.39989328
H	-4.10696858	-0.37869597	1.27934718
H	-2.54877828	-1.27314106	-1.21998164
H	-3.53457250	-2.28473989	-0.15354127

TS1-E
COSMO(Et_2O)-ZORA-BLYP-D3(BJ)/DZP
E = -7603.95
G = -7350.28
COSMO(Et_2O)-ZORA-M06-2X/TZ2P//COSMO(Et_2O)-ZORA-BLYP-D3(BJ)/DZP
E = -11107.91
G = -10854.24
N_{imag} = 1, 1568 i cm^{-1}

C	-0.38942116	-7.13811302	-5.75930602
C	-2.69329036	-7.86265884	-5.83664126
C	-1.21164531	-8.83334994	-4.12483643
N	-2.54454034	-8.64984684	-4.74705120
N	-1.74254906	-7.10405439	-6.33755273
C	-0.10167317	-8.49697950	-5.11882171
H	-0.29765022	-6.32668002	-5.01765634
H	0.32931129	-6.93946066	-6.56447829
C	-4.03636986	-7.80567718	-6.52662811
H	-1.13980568	-9.88392362	-3.81362799
H	-1.14533150	-8.20321753	-3.22207653
C	-3.67594019	-9.37198712	-4.13206817
H	0.87015578	-8.49010653	-4.60669648
H	-0.08340173	-9.26206765	-5.90480051
C	-4.77273488	-8.45797512	-3.55798487
C	-5.75666185	-7.91028571	-4.60484001
C	-5.11527867	-7.05567906	-5.70914924
H	-4.38633629	-8.82184981	-6.75402815
H	-3.87959416	-7.29469439	-7.48315551
H	-3.24327232	-9.96980910	-3.32203863
H	-4.10359013	-10.08312336	-4.85710776
H	-4.28761956	-7.63008572	-3.01463417
H	-5.33965825	-9.04288933	-2.81609370
H	-6.53054892	-7.31619352	-4.09323593
H	-6.27562324	-8.76327403	-5.07711030
H	-4.66582351	-6.14602720	-5.27830138
H	-5.90324806	-6.72610014	-6.40331191
O	-3.13516677	-10.58020278	-8.90894317
C	-1.90834555	-10.27677129	-8.38846477
H	-0.47371425	-5.20016289	-10.08830732
C	-0.77483112	-8.07073798	-9.18316116
C	-1.12679954	-10.04094805	-10.83551122
O	-1.19611696	-11.40006479	-10.88865178
H	0.38222560	-5.74594601	-8.62994252
C	-1.24726130	-11.96752719	-12.23772742
H	-2.13295548	-11.59642872	-12.76409253

H	-0.34318377	-11.69507167	-12.79202276
O	2.19829243	-8.23660055	-7.84862367
H	-3.53813487	-12.39604322	-7.93311395
C	0.22420103	-8.90814848	-8.95185612
C	-1.44978181	-6.85078476	-9.02457613
O	1.80328127	-10.46766827	-8.26082332
C	3.04657414	-10.75876936	-7.55514367
O	-1.06610815	-9.31974664	-11.82240363
C	1.48274087	-9.12949230	-8.31924867
H	-4.10108668	-10.93763093	-7.06830216
H	3.89328076	-10.27671269	-8.05690416
H	3.14475263	-11.84672721	-7.58795337
H	2.98524663	-10.40583011	-6.51952075
C	-1.05469171	-9.55794084	-9.40586614
C	-0.61296815	-5.56782140	-9.05937164
H	-1.73953807	-6.96537411	-7.66728098
H	-2.40951004	-6.79236660	-9.55189967
O	-1.58014660	-10.53001347	-7.23899283
C	-3.98608916	-11.40264513	-8.05079638
H	-4.94476177	-11.46527284	-8.57030970
H	-1.09763534	-4.77079663	-8.47543385
H	-1.30478816	-13.04791578	-12.08872014

TS1-Z

COSMO(Et_2O)-ZORA-BLYP-D3(BJ)/DZP

E = -7604.59

G = -7350.75

COSMO(Et_2O)-ZORA-M06-2X/TZ2P//COSMO(Et_2O)-ZORA-BLYP-D3(BJ)/DZP

E = -11108.64

G = -10854.80

N_{imag} = 1, 1552 i cm^{-1}

C	-0.41387912	-7.06588444	-5.80726998
C	-2.70481518	-7.83358273	-5.81090568
C	-1.15335393	-8.75648150	-4.13541293
N	-2.50916514	-8.60016309	-4.71558893
N	-1.78361365	-7.05958368	-6.34851672
C	-0.08544820	-8.41797216	-5.17282941
H	-0.31622455	-6.25319498	-5.06815152
H	0.27682684	-6.85252600	-6.63446116
C	-4.06773649	-7.81759231	-6.46194906
H	-1.05619511	-9.80175897	-3.81413777
H	-1.06589902	-8.11413215	-3.24331793
C	-3.60265299	-9.35963484	-4.07570872
H	0.90662479	-8.40076948	-4.70191617
H	-0.09450621	-9.18791113	-5.95433205
C	-4.71423713	-8.48168297	-3.47544031
C	-5.73891364	-7.96602353	-4.49882749
C	-5.14811343	-7.09700826	-5.61981081
H	-4.39047867	-8.84574181	-6.67483983
H	-3.95455987	-7.30690388	-7.42265114

H	-3.13247973	-9.94295786	-3.27624029
H	-4.02054382	-10.08352274	-4.79396738
H	-4.24335431	-7.63852069	-2.94328439
H	-5.24436556	-9.08435045	-2.72067562
H	-6.51702493	-7.39269588	-3.97025319
H	-6.24414755	-8.83487100	-4.95675636
H	-4.71567137	-6.17309806	-5.20208482
H	-5.96122020	-6.79283459	-6.29629002
O	-3.15122823	-10.63398123	-9.03220844
C	-1.97897168	-10.27188522	-8.43108207
H	-3.34700747	-7.69046356	-9.84599050
C	-0.77392575	-8.09221425	-9.16606986
C	-1.07318094	-10.04030045	-10.84603595
O	-1.13104298	-11.39837145	-10.92860555
H	-2.60760230	-6.45767760	-10.88649717
C	-1.12271090	-11.93753923	-12.29004359
H	-1.98714661	-11.55921338	-12.84583514
H	-0.19764643	-11.64993687	-12.80022988
O	2.18763530	-8.31685261	-7.78619065
H	-3.59717929	-12.34349774	-7.89286655
C	0.19999299	-8.94478760	-8.89227198
C	-1.49171680	-6.89278370	-9.05083498
O	1.73392463	-10.53943969	-8.18618483
C	2.97526311	-10.85836690	-7.48948118
O	-0.97827223	-9.29945868	-11.81590846
C	1.44883349	-9.19298483	-8.25201193
H	-4.33953352	-10.82863752	-7.30220332
H	3.82907938	-10.39708940	-7.99855539
H	3.04774560	-11.94840876	-7.52106827
H	2.93048630	-10.50255544	-6.45395660
C	-1.06167604	-9.57937789	-9.40838615
H	-0.84947621	-6.00046406	-9.03687650
H	-1.77308940	-6.97521266	-7.67690690
C	-2.79075892	-6.74243110	-9.83804957
O	-1.74482970	-10.44746258	-7.24399962
C	-4.06870043	-11.40129502	-8.19331776
H	-4.94516564	-11.58380311	-8.81915081
H	-3.43464435	-5.97210019	-9.38843683
H	-1.18117368	-13.02107190	-12.16624206

Int1-E
COSMO(Et_2O)-ZORA-BLYP-D3(BJ)/DZP
E = -7611.15
G = -7355.73
COSMO(Et_2O)-ZORA-M06-2X/TZ2P//COSMO(Et_2O)-ZORA-BLYP-D3(BJ)/DZP
E = -11119.83
G = -10864.41
N_{imag} = 0

C	-0.24562891	-7.33384802	-5.76925014
C	-2.60804302	-8.01092895	-5.76448438

C	-1.10478744	-8.90222814	-4.03332532
N	-2.45413902	-8.67443939	-4.61978990
N	-1.61218255	-7.32699323	-6.31629724
C	-0.01074512	-8.67417641	-5.07385751
H	-0.14200577	-6.49495911	-5.06322367
H	0.45690263	-7.19562886	-6.59748259
C	-3.94044655	-7.95927674	-6.45808349
H	-1.08818371	-9.93995527	-3.67627183
H	-0.98457894	-8.23547804	-3.16547293
C	-3.60695016	-9.26942280	-3.90180828
H	0.97189843	-8.68787883	-4.58609214
H	-0.04863975	-9.46988983	-5.82892236
C	-4.64429046	-8.23724497	-3.43089626
C	-5.62527483	-7.76880682	-4.51873028
C	-4.97255523	-7.07142440	-5.72246386
H	-4.32723802	-8.98082736	-6.55949426
H	-3.76456365	-7.57510069	-7.47098758
H	-3.17906033	-9.78027980	-3.03289693
H	-4.07009934	-10.04169643	-4.53586091
H	-4.10867734	-7.37674473	-2.99709472
H	-5.22099565	-8.70107053	-2.61545693
H	-6.36101251	-7.08663102	-4.06498825
H	-6.18977644	-8.64503974	-4.88272932
H	-4.48267378	-6.13592001	-5.40792572
H	-5.75617695	-6.79665290	-6.44384722
O	-3.20161053	-10.64499407	-9.20598945
C	-2.08747307	-10.18971776	-8.54968711
H	-0.88427579	-4.84778848	-9.95481852
C	-0.92429679	-7.99303252	-9.29602700
C	-1.11198070	-10.01632573	-10.91979200
O	-0.96010514	-11.37438205	-10.92107778
H	0.14102997	-5.65787563	-8.74713312
C	-0.87214004	-11.98559479	-12.24648968
H	-1.78410908	-11.77765863	-12.81670731
H	-0.00239694	-11.59332929	-12.78420836
O	2.04834899	-8.20699609	-7.82565385
H	-3.60248545	-12.25560918	-7.91234718
C	0.09350258	-8.83819299	-8.99298746
C	-1.58783389	-6.81213804	-9.33775723
O	1.56874638	-10.44300553	-8.18081339
C	2.78268802	-10.73536984	-7.44179078
O	-1.13657174	-9.33800389	-11.93896381
C	1.28939310	-9.07772936	-8.30552976
H	-4.59497551	-10.79341514	-7.63279074
H	3.65853321	-10.29229075	-7.93216225
H	2.86122776	-11.82683647	-7.43553619
H	2.71591097	-10.35260163	-6.41569042
C	-1.15579581	-9.49163698	-9.50471121
C	-0.89963921	-5.49133266	-9.05893243
H	-1.72159423	-7.11157593	-7.32807874

H	-2.59808524	-6.79457080	-9.75086994
O	-1.92024528	-10.32241879	-7.34471618
C	-4.13041473	-11.42639877	-8.39499991
H	-4.88388199	-11.79483014	-9.09528163
H	-1.40089856	-4.91328874	-8.26257624
H	-0.76454443	-13.05670198	-12.06016599

Int1-Z
COSMO(Et_2O)-ZORA-BLYP-D3(BJ)/DZP
E = -7611.87
G = -7356.13
COSMO(Et_2O)-ZORA-M06-2X/TZ2P//COSMO(Et_2O)-ZORA-BLYP-D3(BJ)/DZP
E = -11120.66
G = -10864.92
N_{imag} = 0

C	-0.21671162	-7.32325136	-5.69885345
C	-2.58319737	-7.98847859	-5.74312544
C	-1.10118816	-8.95133499	-4.03222322
N	-2.44381282	-8.69403999	-4.62207745
N	-1.57814140	-7.29046359	-6.25914979
C	0.00354240	-8.69047155	-5.05320254
H	-0.11685765	-6.51311782	-4.95958636
H	0.49364403	-7.15560604	-6.51561767
C	-3.90955794	-7.90708102	-6.44640126
H	-1.09294688	-10.00196316	-3.71541834
H	-0.98589762	-8.31883725	-3.13843285
C	-3.60264681	-9.33190866	-3.95239701
H	0.98155993	-8.72860574	-4.55746439
H	-0.03264432	-9.45725993	-5.83774755
C	-4.65675048	-8.33357750	-3.44742654
C	-5.62363481	-7.81796463	-4.52600196
C	-4.95459233	-7.05928295	-5.68248892
H	-4.28993735	-8.92423492	-6.60112293
H	-3.72687299	-7.47624375	-7.43623620
H	-3.18403973	-9.88052291	-3.10222641
H	-4.04731016	-10.07652334	-4.63124730
H	-4.13594956	-7.49199870	-2.96142095
H	-5.24229014	-8.84314263	-2.66630398
H	-6.37103983	-7.16327431	-4.05145056
H	-6.17648332	-8.67816770	-4.94231556
H	-4.47250352	-6.13951339	-5.31397846
H	-5.72767989	-6.75052436	-6.40144631
O	-3.23419182	-10.59693791	-9.17713039
C	-2.11577171	-10.17660363	-8.50551270
H	-3.48283166	-7.81135980	-10.04739383
C	-0.96295820	-7.96911280	-9.24431692
C	-1.08278758	-10.00190201	-10.85415575
O	-1.00542327	-11.36663918	-10.86063503
H	-3.03789581	-6.38571626	-11.00489771
C	-0.88878346	-11.97272276	-12.18600155

H	-1.76030608	-11.71150951	-12.79617332
H	0.02514857	-11.62493218	-12.67919207
O	2.04739971	-8.13482663	-7.81664978
H	-3.68159640	-12.21748384	-7.91152985
C	0.05380631	-8.79651596	-8.89985382
C	-1.69114314	-6.83142541	-9.35022631
O	1.58728288	-10.37787043	-8.15870026
C	2.83507420	-10.65347979	-7.47258085
O	-1.02308935	-9.31679953	-11.86720013
C	1.28279297	-9.01571646	-8.26885271
H	-4.64744755	-10.73985078	-7.62128470
H	3.68355092	-10.19890091	-7.99943357
H	2.92904944	-11.74376876	-7.47015936
H	2.80760263	-10.27176099	-6.44411493
C	-1.16515824	-9.47905617	-9.44145227
H	-1.19258830	-5.87731223	-9.15112029
H	-1.68514657	-7.01026576	-7.25456679
C	-3.06351763	-6.79772680	-9.98020979
O	-1.96404807	-10.32704797	-7.30013166
C	-4.18669514	-11.37051770	-8.38796700
H	-4.94000950	-11.71329451	-9.10143589
H	-3.76941994	-6.17355866	-9.40739175
H	-0.84836356	-13.04941083	-12.00503108

TS2-E

COSMO(Et_2O)-ZORA-BLYP-D3(BJ)/DZP

E = -7609.53

G = -7356.26

COSMO(Et_2O)-ZORA-M06-2X/TZ2P//COSMO(Et_2O)-ZORA-BLYP-D3(BJ)/DZP

E = -11119.33

G = -10866.06

N_{imag} = 1, 1513 i cm^{-1}

C	9.85023891	3.82256837	4.86607162
C	7.97895171	3.94226960	3.32922628
C	7.64437908	4.67589850	5.65369805
N	7.14717076	4.44120307	4.28016064
N	9.24149436	3.65774609	3.54029826
C	8.79467033	3.72002578	5.96713975
H	10.36239479	4.79644623	4.90528965
H	10.62486891	3.05292431	4.96946233
C	7.43722394	3.73206529	1.93356749
H	6.80493032	4.51333888	6.34209148
H	7.96984577	5.72513612	5.75390817
C	5.79769683	4.95324271	3.95845548
H	9.22626188	3.96571052	6.94733228
H	8.40740124	2.68996809	6.01090965
C	5.79488951	6.16749782	3.01189240
C	5.93890626	5.80175070	1.52507825
C	7.23363705	5.05724055	1.16168597
H	6.48954923	3.17378574	1.98316413

H	8.14787557	3.09582148	1.40120780
H	5.34986014	5.23418046	4.91851542
H	5.18142477	4.14034139	3.54343252
H	6.59930520	6.85573402	3.32083633
H	4.84149417	6.70177305	3.15268863
H	5.86975263	6.72001204	0.92030914
H	5.07947402	5.17002454	1.23848952
H	8.11117174	5.70257777	1.33078630
H	7.21450138	4.82559105	0.08735080
C	12.85154199	-1.06190528	0.07506322
H	9.92004231	2.98593590	2.65245132
H	11.84477813	3.71615731	-1.14560410
C	10.54618793	2.14198084	1.70295534
H	9.62791383	3.23668497	-2.36331664
H	9.40247290	4.70913250	-1.40522281
H	12.30167607	-1.49612352	-0.76300441
H	12.66902935	-1.62454389	0.99696850
H	13.92584859	-1.03107599	-0.13519859
C	12.07904553	2.26963990	1.50399738
O	12.41112583	3.92258945	3.26330995
C	9.84554191	0.85988740	1.74547254
C	11.04907899	2.74598244	0.51853236
H	15.06764185	4.29298621	3.35830165
O	13.79407047	0.63068066	1.92638334
O	14.01595288	3.59484001	1.67420729
C	14.82924656	4.61344946	2.33846689
H	14.28958237	5.56599941	2.36464546
H	8.79544757	3.14656896	-0.79464632
O	8.80208664	0.60561456	1.14061927
O	10.42025938	-0.01351988	2.64326134
C	9.73182338	-1.29144193	2.77826824
H	8.70882046	-1.13864737	3.14105958
H	10.31992515	-1.85836204	3.50453466
H	9.70286155	-1.81099589	1.81367217
C	9.61789181	3.63057242	-1.33388966
O	12.32193874	0.29284196	0.21294926
C	12.82277669	3.32714265	2.27906302
C	12.84862057	1.00916689	1.24373617
H	15.73503752	4.69323137	1.73322412
C	10.93405240	3.37520716	-0.64607676

TS2-Z
COSMO(Et_2O)-ZORA-BLYP-D3(BJ)/DZP
E = -7609.55
G = -7355.95
COSMO(Et_2O)-ZORA-M06-2X/TZ2P//COSMO(Et_2O)-ZORA-BLYP-D3(BJ)/DZP
E = -11118.35
G = -10864.75
N_{imag} = 1, 1490 i cm^{-1}

C	9.99553720	4.07286449	4.87691395

C	7.99349308	3.87382593	3.52363802
C	7.99738011	5.47028397	5.40126294
N	7.33561617	4.81056330	4.25345141
N	9.22202898	3.48335566	3.77502909
C	9.06927339	4.55600731	5.99199442
H	10.59483857	4.91107428	4.48393124
H	10.70053269	3.31388536	5.23170501
C	7.28971991	3.26984768	2.33100639
H	7.22341571	5.69270380	6.14727738
H	8.43659533	6.42649525	5.07078907
C	6.00928867	5.33359014	3.85801252
H	9.63577107	5.09630426	6.76290973
H	8.58536697	3.68847036	6.46703585
C	6.01163381	6.12278005	2.53631103
C	5.98004548	5.23861615	1.27857293
C	7.15970637	4.26269773	1.15010483
H	6.29336583	2.91040937	2.63244951
H	7.85562905	2.39425097	2.00420341
H	5.69299127	5.98965732	4.67696267
H	5.28404391	4.50644014	3.81212221
H	6.89592302	6.78161800	2.52289517
H	5.12353838	6.77517336	2.53288101
H	5.94473510	5.88385252	0.38634204
H	5.04137843	4.65677554	1.28486373
H	8.10684034	4.81555496	1.05035429
H	7.03777218	3.67445209	0.22815330
C	12.78939316	-0.73299382	-0.54858268
H	9.83253203	2.67582870	2.95376644
H	9.70826512	3.86366135	-0.45645333
C	10.44966722	1.91401102	1.91350615
H	11.73935580	5.42561755	-0.82635273
H	11.89842496	4.02537851	-1.89744665
H	12.15095031	-1.04772528	-1.37709603
H	12.83908132	-1.50828086	0.22366946
H	13.79826150	-0.49306135	-0.90051052
C	11.96438582	2.11661524	1.68519233
O	12.66082804	2.76808201	3.92854073
C	9.77926949	0.63237600	1.71793608
C	10.89059931	2.78201953	0.87417718
H	15.13255720	3.76172562	3.58806975
O	13.85592949	0.63999910	1.49452652
O	13.63819315	3.72938990	2.10707501
C	14.52006653	4.46565725	3.01459881
H	13.92496321	5.08203624	3.69631708
H	12.82049146	4.08443880	-0.37022337
O	8.71485521	0.47103777	1.11410368
O	10.42181067	-0.39304089	2.37687975
C	9.78800963	-1.69884518	2.23731033
H	8.76889072	-1.67613184	2.63995502
H	10.41635899	-2.38477389	2.81118548

H	9.75718098	-1.99319635	1.18193590
C	11.85937889	4.32991771	-0.83844749
O	12.14444203	0.46140374	-0.00842661
C	12.76431019	2.88229373	2.71696107
C	12.76967644	1.01590968	1.06265767
H	15.14149297	5.08695830	2.36583629
C	10.72532263	3.64799804	-0.11994209

24c

COSMO(Et_2O)-ZORA-BLYP-D3(BJ)/DZP

E = -4167.83

G = -4053.67

COSMO(Et_2O)-ZORA-M06-2X/TZ2P//COSMO(Et_2O)-ZORA-BLYP-D3(BJ)/DZP

E = -6227.83

G = -6113.67

N_{imag} = 0

C	-0.20725364	0.15804770	0.25812153
C	0.14573422	1.68274141	0.03005344
C	1.16230862	0.63446333	0.05774806
H	0.05930357	2.79847496	-3.87465187
C	0.04826360	2.64586276	1.20603424
C	-0.28154008	2.31256852	-1.27429476
H	-0.51825101	1.14379013	-4.29968003
C	2.45090314	0.33998191	0.07991316
C	-0.64802991	-0.24471443	1.63257268
O	1.00016048	3.23594832	1.68739897
O	-1.23572459	2.76212092	1.60980168
C	-1.44838818	3.66446181	2.74751627
H	-2.52520676	3.64411956	2.92523297
H	-1.10874636	4.67204271	2.48851531
H	-0.89649188	3.28961195	3.61518102
O	-0.60799842	3.48704064	-1.37200969
O	-0.23387578	1.43246773	-2.29973666
C	-0.61705454	1.98110928	-3.60652008
H	-1.64928057	2.34246927	-3.56725245
O	-0.10256813	0.09030428	2.67333733
O	-1.74753656	-1.04380622	1.55803817
C	-2.26487338	-1.49847781	2.85603114
H	-1.50059802	-2.08793363	3.37197359
H	-3.13602006	-2.10943021	2.61218652
H	-2.54582287	-0.63301955	3.46380883
C	2.98936444	-1.04333254	0.29566660
H	3.61425567	-1.35492342	-0.55562770
H	2.18008368	-1.77275881	0.42547407
H	3.63125372	-1.06777496	1.18996498
H	3.16879516	1.15251961	-0.05867507
H	-0.73833808	-0.31314622	-0.56953176

11. Single Crystal X-Ray Diffraction Data

Single Crystal X-Ray Diffraction Data for Compound 4b (CCDC 2342557)

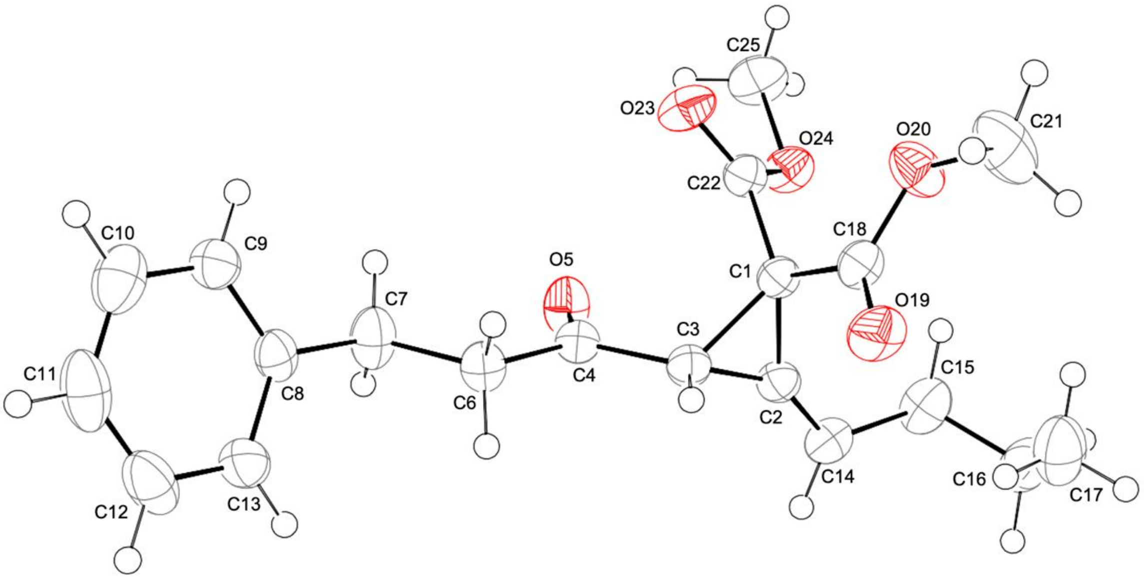

Table S7. Crystal data and structure refinement for **4b**.

CCDC	2342557	
Empirical formula	C20 H24 O5	
Formula weight	344.41	
Temperature	150 K	
Wavelength	1.54184 Å	
Crystal system / Space group	Monoclinic / $P\ 2_1$	
Unit cell dimensions	a = 7.83500(10) Å	
		α= 90°.
	b = 8.68370(10) Å	β= 102.2435(17)°.
	c = 14.0099(2) Å	γ = 90°.
Volume	931.51(2) Å^3	
Z	2	
Density (calculated)	1.228 Mg/m^3	
Absorption coefficient	0.716 mm^{-1}	
F(000)	368	
Crystal size	0.19 x 0.11 x 0.06 mm^3	
Theta range for data collection	3.228 to 76.269°.	

Index ranges	-9<=h<=9, -10<=k<=10, -17<=l<=17
Reflections collected	16967
Independent reflections	3842 [R(int) = 0.034]
Completeness to theta = 74.744°	99.7 %
Absorption correction	Semi-empirical from equivalents
Max. and min. transmission	0.96 and 0.84
Refinement method	Full-matrix least-squares on F^2
Data / restraints / parameters	3842 / 1 / 228
Goodness-of-fit on F^2	0.9959
Final R indices [I>2sigma(I)]	R1 = 0.0312, wR2 = 0.0800
R indices (all data)	R1 = 0.0333, wR2 = 0.0823
Absolute structure parameter	0.01(7)
Extinction coefficient	19(3)
Largest diff. peak and hole	0.04 and -0.05 e.Å^{-3}

Single Crystal X-Ray Diffraction Data for Compound 9b
(CCDC 2342558)

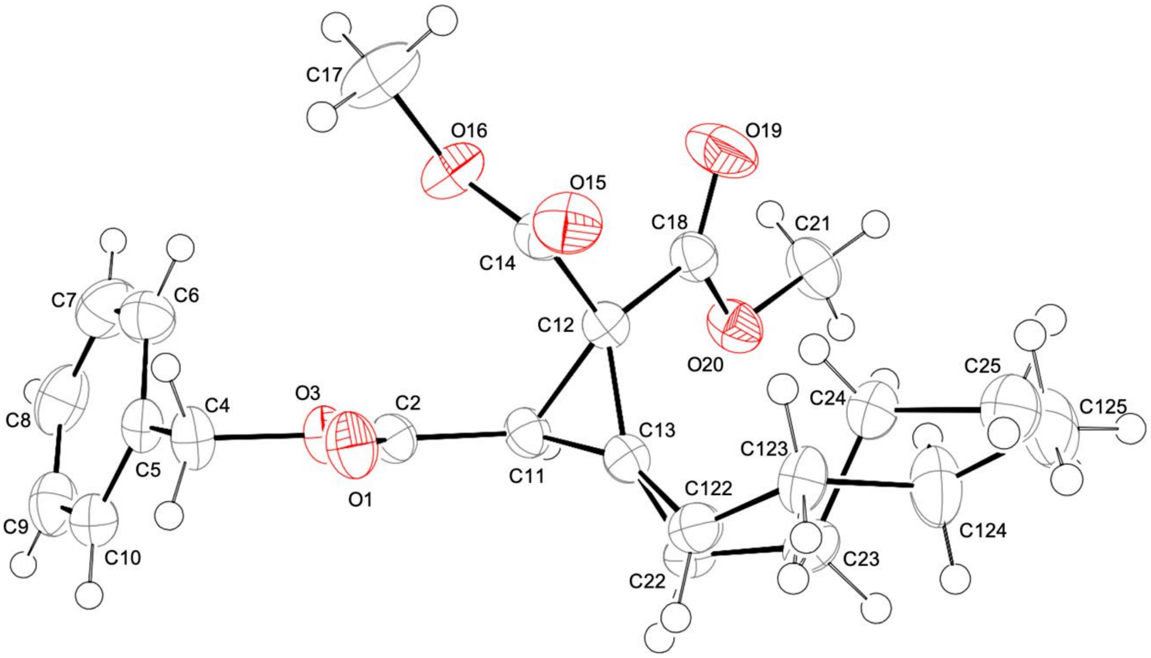

Table S8. Crystal data and structure refinement for **8b**.

CCDC	2342558	
Empirical formula	C19 H22 O6	
Formula weight	346.38	
Temperature	150 K	
Wavelength	1.54184 Å	
Crystal system / Space Group	Monoclinic / $P\ 2_1$	
Unit cell dimensions	a = 9.2248(2) Å	
		α= 90°.
	b = 7.94430(10) Å	β= 99.1678(16)°.
	c = 12.6771(2) Å	γ = 90°.
Volume	917.17(3) Å^3	
Z	2	
Density (calculated)	1.254 Mg/m^3	
Absorption coefficient	0.773 mm^{-1}	
F(000)	368	
Crystal size	0.28 x 0.09 x 0.06 mm^3	
Theta range for data collection	3.532 to 75.993°.	

Index ranges	-9<=h<=11, -9<=k<=9, -15<=l<=14
Reflections collected	11542
Independent reflections	3789 [R(int) = 0.022]
Completeness to theta = 75.993°	99.8 %
Absorption correction	Semi-empirical from equivalents
Max. and min. transmission	0.95 and 0.86
Refinement method	Full-matrix least-squares on F^2
Data / restraints / parameters	3789 / 9 / 265
Goodness-of-fit on F^2	1.0019
Final R indices [I>2sigma(I)]	R1 = 0.0277, wR2 = 0.0730
R indices (all data)	R1 = 0.0287, wR2 = 0.0744
Absolute structure parameter	0.08(5)
Extinction coefficient	26(3)
Largest diff. peak and hole	0.05 and -0.05 e.Å^{-3}

Single Crystal X-Ray Diffraction Data for Compound 27b

(CCDC 2456302)

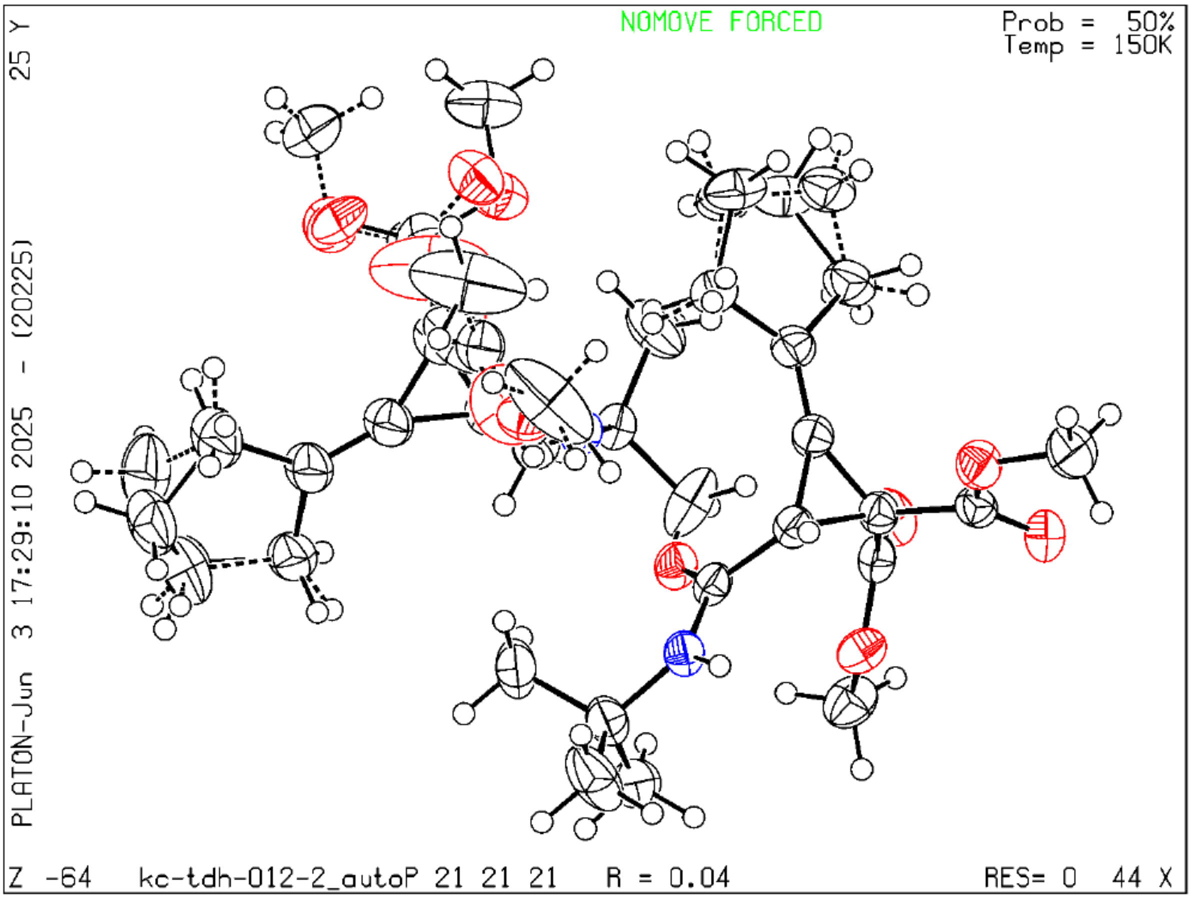

Table S9. Crystal data and structure refinement for **27b**.

Bond precision:	C-C = 0.0032 A	Wavelength=1.54184	
Cell:	a=9.2750(1)	b=9.5932(1)	c=40.8132(4)
	alpha=90	beta=90	gamma=90
Temperature: 150 K			

	Calculated	Reported
Volume	3631.43(7)	3631.43(7)
Space group	P 21 21 21	P 21 21 21
Hall group	P 2ac 2ab	P 2ac 2ab
Moiety formula	C17 H25 N O5	C17 H25 N O5
Sum formula	C17 H25 N O5	C17 H25 N O5
Mr	323.38	323.38
Dx,g	1.183	1.183

Z	8	8
Mu (mm-1)	0.713	0.713
F000	1392.0	1392.0
F000'	1396.50	
h,k,lmax	11,12,51	11,12,51
Nref	7655[4345]	7571
Tmin,Tmax	0.880,0.965	0.855,1.000
Tmin'	0.752	

Correction method=# Reported T Limits: Tmin=0.855 Tmax=1.000

AbsCorr = MULTI-SCAN

Data completeness= 1.74/0.99 Theta(max)= 76.803

R(reflections)= 0.0352(6962) wR2(reflections)= 0.0937(7571)

S = 1.029 Npar= 547

12. References

1. L. Palatinus, G. Chapuis, SUPERFLIP - A Computer Program for the Solution of Crystal Structures by Charge Flipping in Arbitrary Dimensions. *J. Appl. Cryst.* **40**, 786-790 (2007).

2. P. Parois, R. I. Cooper, A. L. Thompson, Crystal Structures of Increasingly Large Molecules: Meeting the Challenges with CRYSTALS Software. *Chem. Cent. J.* 9:30. (2015).

3. R. I. Cooper, A. L. Thompson, D. J. Watkin, CRYSTALS Enhancements: Dealing with Hydrogen Atoms in Refinement. *J. Appl. Cryst.* **43**, 1100-1107 (2010)

4. F. Zhang, J. M. Fox, Synthesis of Cyclopropene α-Amino Acids via Enantioselective Desymmetrization. *Org. Lett.* **8**, 2965–2968 (2006).

5. A. Basheer, M. Mishima, I. Marek, Regioselective Carbon-Carbon Bond Cleavage in the Oxidation of Cyclopropenylcarbinols. *Org. Lett.* **13**, 4076–4079 (2011).

6. E. Zohar, I. Marek, Diastereoselective Reduction of Cyclopropenylcarbinol: New Access to Anti-Cyclopropylcarbinol Derivatives. *Org. Lett.* **6**, 341–343 (2004).

7. G. Tojo, M. Fernández, Oxidation of Alcohols to Aldehydes and Ketones: A Guide to Current Common Practice. (Springer, 2006).

8. D. Rozsar, M. Formica, K. Yamazaki, T. A. Hamlin, D. J. Dixon, Bifunctional Iminophosphorane-Catalyzed Enantioselective Sulfa-Michael Addition to Unactivated α,β-Unsaturated Amides. *J. Am. Chem. Soc.* **144**, 1006–1015 (2022).

9. S. Duan, S. Li, X. Ye, N. Du, C. Tan, Z. Jiang, Enantioselective Synthesis of Dialkylated α-Hydroxy Carboxylic Acids Through Asymmetric Phase-Transfer Catalysis. *J. Org. Chem.* **80**, 7770–7778 (2015).

10. A. J. M. Farley, Development and Application of a New Class of Potent Bifunctional Organocatalysts. (University of Oxford, 2015).

11. A. J. M. Farley, C. Sandford, D. J. Dixon, Bifunctional Iminophosphorane Catalyzed Enantioselective Sulfa-Michael Addition to Unactivated α-Substituted Acrylate Esters. *J. Am. Chem. Soc.* **137**, 15992–15995 (2015).

12. J. Yang, A. J. M. Farley, D. J. Dixon, Enantioselective Bifunctional Iminophosphorane Catalyzed Sulfa-Michael Addition of Alkyl Thiols to Unactivated β-Substituted-α,β-Unsaturated Esters. *Chem. Sci.* **8**, 606–610 (2016).

13. B. Muriel, J. Waser, Azide Radical Initiated Ring Opening of Cyclopropenes Leading to Alkenyl Nitriles and Polycyclic Aromatic Compounds. *Angew. Chem. Int. Ed.* **60**, 4075–4079 (2021).

14. Y. Yang, P. Antoni, M. Zimmer, K. Sekine, F. F. Mulks, L. Hu, L. Zhang, M. Rudolph, F. Rominger, A. S. K. Hashmi, Dual Gold/Silver Catalysis Involving Alkynylgold(iii) Intermediates Formed by Oxidative Addition and Silver-Catalyzed C−H Activation for the Direct Alkynylation of Cyclopropenes. *Angew. Chem. Int. Ed.* **58**, 5129–5133 (2019).

15. T. Yamakawa, H. Kinoshita, K. Miura, Synthetic utility of tribenzyltin hydride and its derivatives as easily accessible, removable, and decomposable organotin reagents. *J. Organomet. Chem.* **724**, 29–134 (2013).

16. A. N. Baumann, A. Music, K. Karaghiosoff, D. Didier, Highly Diastereoselective Approach to Methylenecyclopropanes via Boron-Homologation/Allylboration Sequences. *Chem. Commun.* **52**, 2529–2532 (2016).

17. Y. Yang, H. Ma, Room-Temperature Direct Benzylic Oxidation Catalyzed by Cobalt(II) Perchlorate. *Tetrahedron Lett*. **57**, 5278–5280 (2016)

18. B. S. Schreib, J. Margarini, E. M. Carreira, Ritter Reaction for the Synthesis of Picolinamides, Tetrahedron, 122, 132937 (2022).

19. J. T. R. Liddon, P. J. Lindsay-Scott, J. Robertson, Secondary Products from Intramolecular Cycloadditions of Azidoalkyl Enol Ethers and Azidoalkyl Vinyl Bromides: 1 Azadienes, Their Reactions with Diphenylketene, and Radical Cyclizations To Form Bi- and Tricyclic Lactams, *J. Org. Chem.* **84**, 13780–13793 (2019).

20. P. A. Jacobi, Y. Li, Enantioselective Syntheses of Ring-C Precursors of vit. B12. Reagent Control. *Org. Lett.* **5**, 701–704 (2003).

21. A. R. A. S. Deshmukh, V. K. Gumaste, Process for Preparing Alkyl/Aryl Chloroformates. (U.S. Patent No. 6919471B2) (2005).

22. W. Bicker, K. Kacprzak, M. Kwit, M. Lämmerhofer, J. Gawronski, W. Lidner, Assignment of Absolute Configurations of Permethrin and its Synthon 3-(2,2-dichlorovinyl)-2,2-dimethylcyclopropanecarboxylic acid by Electronic Circular Dichroism, Optical Rotation, and X-Ray Crystallography. *Tetrahedron Asymmetry* **20**, 1027–1035 (2009).

23. G. te Velde, F. M. Bickelhaupt, E. J. Baerends, C. Fonseca Guerra, S. J. A. van Gisbergen, J. G. Snijders, T. Ziegler, Chemistry with ADF. *J. Comput. Chem.* **22**, 931−967 (2001).

24. C. Fonseca Guerra, J. G. Snijders, G. te Velde, E. J. Baerends, Towards an Order-N DFT Method. *Theor. Chem. Acc.* **99**, 391−403 (1998).

25. ADF2018.105, SCM Theoretical Chemistry; Vrije Universiteit: Amsterdam, The Netherlands. https://www.scm.com (accessed 2021-12-08).

26. J. C. Slater, Quantum Theory of Molecules and Solids. (McGraw-Hill, New York, 1974).

27. A. D. Becke, Density Functional Calculations of Molecular Bond Energies. *J. Chem. Phys.* **84**, 4524–4529 (1986).

28. A. D. Becke, Density-Functional Exchange-Energy Approximation with Correct Asymptotic Behavior. *Phys. Rev. A* **38**, 3098–3100 (1988).

29. C. Lee, W. Yang, R. G. Parr, Development of the Colle-Salvetti Correlation-Energy Formula into a Functional of the Electron Density. *Phys. Rev. B* **37**, 785–789 (1988).

30. E. van Lenthe, E. J. Baerends, Optimized Slater-Type Basis Sets for the Elements 1-118. *J. Comput. Chem.* **24**, 1142–1156 (2003).

31. A. Klamt, G. Schüürmann, COSMO: A New Approach to Dielectric Screening in Solvents with Explicit Expressions for the Screening Energy and its Gradient. *J. Chem. Soc. Perkin Trans. 2* 799–805 (1993).

32. A. Klamt, Conductor-like Screening Model for Real Solvents: A New Approach to the Quantitative Calculation of Solvation Phenomena. *J. Phys. Chem.* **99**, 2224–2235 (1995).

33. A. Klamt, V. Jonas, Treatment of the Outlying Charge in Continuum Solvation Models. *J. Chem. Phys.* **105**, 9972–9981 (1996).

34. C. C. Pye, T. Ziegler, An Implementation of the Conductor-like Screening Model of Solvation within the Amsterdam Density Functional Package. *Theor. Chem. Acc.* **101**, 396–408 (1999).

35. A. D. Becke, E. R. Johnson, A Density-Functional Model of the Dispersion Interaction. *J. Chem. Phys.* **123**, 154101 (2005).

36. S. Grimme, J. Antony, S. Ehrlich, H. Krieg, A Consistent and Accurate ab initio Parametrization of Density Functional Dispersion Correction (DFT-D) for the 94 Elements H-Pu. *J. Chem. Phys.* **132**, 154104 (2010).

37. E. van Lenthe, E. J. Baerends, J. G. Snijders, Relativistic Regular Two-Component Hamiltonians. *J. Chem. Phys.* **99**, 4597–4610 (1993).

38. E. van Lenthe, E. J. Baerends, J. G. Snijders, Relativistic Total Energy using Regular Approximations. *J. Chem. Phys.* **101**, 9783–9792 (1994).

39. CYLview20; Legault, C. Y., Unversité de Sherbrooke: Sherbrooke, www.cylview.org (2020).

40. Y. Zhao, D. G. Truhlar, The M06 suite of density functionals for main group thermochemistry, thermochemical kinetics, noncovalent interactions, excited states, and transition elements: two new functionals and systematic testing of four M06-class functionals and 12 other functionals. *Theor. Chem. Acc.*, **120**, 215– 241 (2008).

41. F. M. Bickelhaupt, E. J. Baerends, in Reviews in Computational Chemistry; K. B. Lipkowitz D. B. Boyd, Wiley, Hoboken, **15**, 1–86 (2000).

42. T. A. Hamlin, P. Vermeeren, C. Fonseca Guerra, F. M. Bickelhaupt, Complementary Bonding Analysis, (Ed. S. Grabowsky), De Gruyter, Berlin, 199–212 (2021).

43. R. van Meer, O. V. Gritsenko, E. J. Baerends, *J. Chem. Theory Comput.* **10**, 4432–4441 (2014).
